# Visible-Light-Induced Radical Cascade [4 + 2]/[4 + 2] Cycloaddition of Underexplored *N*‑Acryloyl Indoles To Access Dihydropyrido[1,2‑*a*]‑indolones

**DOI:** 10.1021/acs.orglett.5c05421

**Published:** 2026-02-23

**Authors:** Cody Bishir, Samuel Milton, Abbey Hubbard, Michael Indalsingh, Zainah Abufouz, Liangyong Mei

**Affiliations:** Department of Chemistry and Biochemistry, 4127 University of North Florida, Jacksonville, Florida 32224, United States

## Abstract

We report a visible-light-mediated radical cascade [4 + 2]/[4 + 2] cycloaddition of simple *N*-acryloyl indoles and *N*-hydroxyphthalimide esters, which provides a streamlined route to structurally complex dihydropyrido­[1,2-*a*]-indolones. The reaction features a sequence of four consecutive radical additions involving two *N*-acryloyl indole molecules, which forges four new C–C bonds and induces dearomatization of one indole ring. The proposed mechanism is supported by observations of [4 + 2] cycloaddition byproducts and additional control experiments. The synthetic utility of this method is demonstrated by the scale-up reaction and downstream derivatizations.

## Graphical abstract

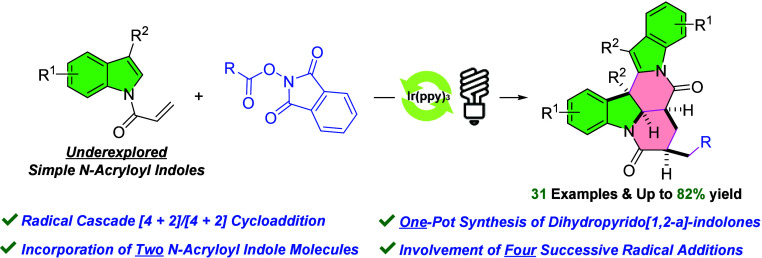

Nitrogen-fused polycyclic indoles, such as dihydropyrido­[1,2-*a*]-indolones (DHPIs), are widespread structural motifs in natural products and pharmaceutical compounds, many of which display notable pharmacological properties and biological activities (Figure 1).^1^ Consequently, substantial efforts have been devoted to their construction by both synthetic and medicinal chemists, leading to the development of numerous elegant methods.^2^ Among these, radical cascade cyclization has distinguished itself as one of the most powerful and well-studied strategies, due to its ability to streamline synthetic sequences, reduce costs, and minimize waste generation.^3^ To access N-fused polycyclic indoles via radical cascade cyclization, two general approaches are typically employed:^3,4^ (i) reactions of N-alkene/alkyne-tethered indoles with external radical precursors and (ii) couplings of indoles bearing N-tethered radical precursors, either through self-coupling or with external alkenes. Both approaches have been successfully realized under various reaction conditions, including photocatalysis, electrocatalysis, and oxidant-promoted processes, all of which proceed through a fundamentally similar mechanism.^3,4^ Typically, an initial single-electron transfer (SET) event generates a reactive radical species from a radical precursor. This radical intermediate then undergoes a series of inter- and/or intramolecular additions to π bonds, driving the cascade cyclization. A final SET step followed by deprotonation ultimately furnishes the desired N-fused polycyclic indole products.

**Figure 1:**
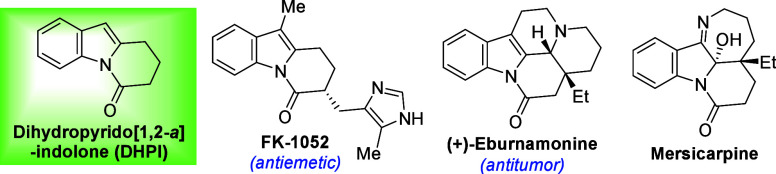
Representative bioactive DHPIs.

Specifically, 2-aryl-*N*-acryloyl indoles and 1-acryloyl-2-cyanoindoles are particularly compelling members of the N-alkene-tethered indole family,^2a,3c^ owing to their dual reactive sites that serve as excellent radical acceptors for radical cascade cyclizations. 2-Aryl-*N*-acryloyl indoles readily engage with a wide range of carbon-,^5^ nitrogen-,^6^ sulfur-,^7^ silicon-,^8^ phosphorus-,^9^ and germanium-centered^10^ radical precursors through transition-metal (TM)-driven, oxidant-mediated, visible-light-induced, or electro-promoted radical cascade cyclizations, enabling efficient access to a large variety of indolo­[2,1-*a*]­isoquinolines.^2a,3c,5–10^ Similarly, pyrrolo­[1,2-*a*]­indolediones can be obtained by treating 1-acryloyl-2-cyanoindoles with different radical precursors in the presence of stoichiometric persulfate under blue light irradiation.^11^ Although impressive progress has been achieved in the study of these 2-substituted-*N*-acryloyl indoles, their structural analogues—simple *N*-acryloyl indoles—are still largely underexplored, despite being more easily synthesized and accessible (Scheme 1a). Only a handful of studies on their reactivity toward N-fused polycyclic indoles have been documented to date.^12^ The initial work was introduced by the Kerr group in 2008,^12a^ who assembled DHPIs by reacting simple *N*-acryloyl indoles with Michael donors in the presence of base and Mn­(OAc)_3_. However, further advances did not appear for over a decade. In 2020, Paton, Smith, and co-workers disclosed a blue-light-mediated Ir­(III)-catalyzed [2 + 2] cycloaddition of simple *N*-acryloyl indoles, offering a facile route to pyrrolo­[1,2-*a*]­indol-3-ones.^12c^ More recently, the Liu and Guo groups showed both photo- and electro-induced radical cascade [3 + 2]/[4 + 2] cyclizations by treating simple *N*-acryloyl indoles with 2-(iodomethyl)­cyclopropanes or α-allyl-activated methylenes, respectively.^12d,12e^ These newly developed transformations are particularly intriguing because they forge three new C–C bonds through sequential radical additions, whereas previous work only generates one or two σ bonds. Despite these notable advances, the field remains underdeveloped, as reported examples are scarce to date. Additionally, these existing studies rely on uncommon radical precursors and are restricted to C2–functionalization of the indole cores. Furthermore, to the best of our knowledge, there are no prior literature reports describing the dimerization of 2-substituted or simple *N*-acryloyl indoles for the synthesis of N-fused polycyclic indoles. Taken together, the reactivity study of simple *N*-acryloyl indoles is still in its infancy. Therefore, exploring their additional reactivity (e.g., dearomatization or dimerization) with commonly used radical precursors is of considerable importance, as it would not only fill the current research gap but also overcome existing limitations, thereby opening new avenues for accessing structurally distinct N-fused polycyclic indoles (e.g., DHPIs).

**Scheme 1:**
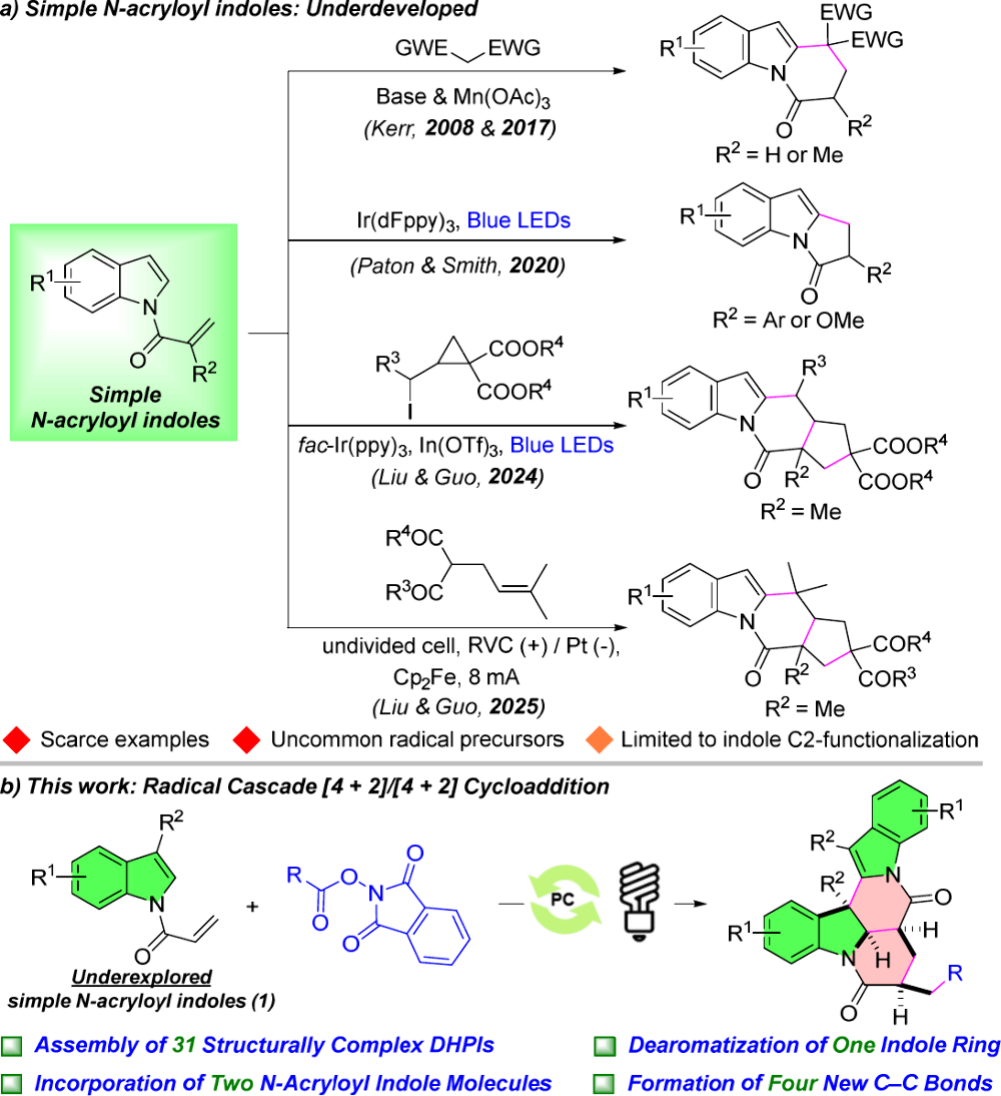
a) Current Reactivity Study of Simple N-Acryloyl Indoles toward N-Fused Polycyclic Indoles and b) This Work

In this work, we report a visible-light-promoted radical cascade [4 + 2]/[4 + 2] cycloaddition of simple *N*-acryloyl indoles (**1**) with *N*-hydroxyphthalimide (NHPI) esters, which provides a straightforward and efficient route to structurally complex DHPIs (Scheme 1b). The proposed mechanism is shown in Scheme 2. The excited state of the photocatalyst (PC*) reduces the radical precursor via oxidative quenching, generating the radical species **I** (R^•^) and PC^+^. Radical **I** then triggers a cascade of three consecutive radical additions: two intermolecular additions to the alkene moieties of two simple *N*-acryloyl indole molecules, followed by an intramolecular addition to the C2=C3 bond of the indole ring, producing the key benzyl radical intermediate **IV**. Intermediate **IV** can diverge into two possible pathways. In Path A, it engages in a fourth radical addition to form another benzyl radical species **V**, which subsequently undergoes SET coupled with deprotonation to yield the desired [4 + 2]/[4 + 2] product. In Path B, an alternative [4 + 2] cycloaddition byproduct may form via direct SET and deprotonation from intermediate **IV**. We envisioned that the intramolecular radical addition in Path A is favored over the competing SET in Path B due to its inherent spatial proximity, thereby selectively delivering the [4 + 2]/[4 + 2] adduct as the major product. Given the broad bioactivity of DHPIs (Figure 1),^1^ developing efficient methods for their synthesis is highly desirable. The methodology described herein constitutes a major advance over existing approaches. On one hand, it accommodates two common types of radical precursors (e.g., NHPI esters and α-bromocarbonyl compounds) while enabling the formation of four new C–C bonds and inducing dearomatization of one indole ring. More importantly, the dimerization of *N*-acryloyl indole molecules is entailed during the formation of final products—an outcome not realized using 2-aryl-*N*-acryloyl indoles, 1-acryloyl-2-cyanoindoles, or simple *N*-acryloyl indoles in previous reports.

**Scheme 2:**
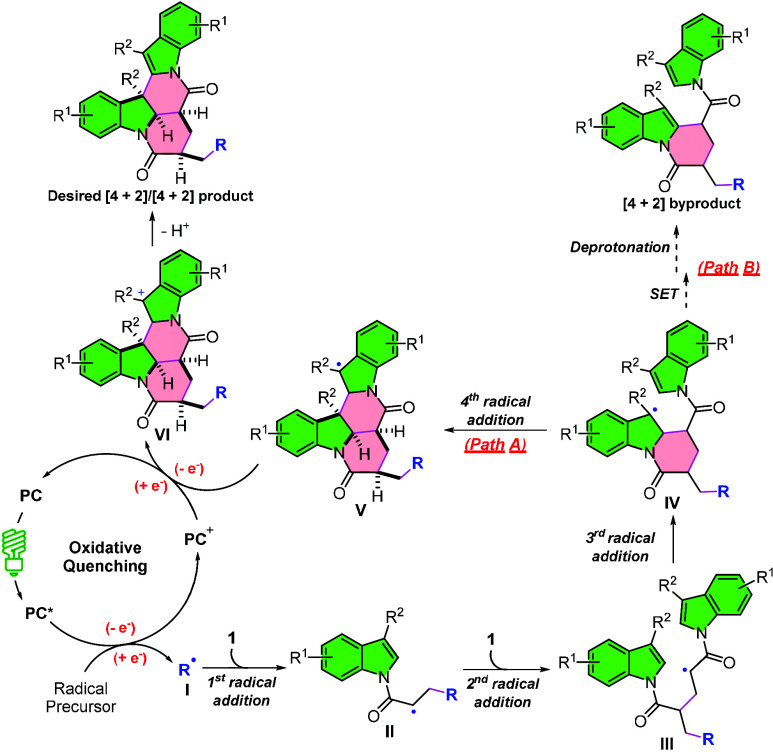
Proposed Reaction Mechanism

As part of our ongoing investigation of redox-active NHPI esters in photocatalysis,^13^ simple *N*-acryloyl indole **1a** and 1,3-dioxoisoindolin-2-yl isobutyrate **2a** were selected as model substrates to optimize reaction conditions (Table 1). Given the reduction potentials of alkyl NHPI esters (*E*_red_ = −1.20 to −1.37 V vs SCE in MeCN),^14^ we initiated the study using *fac*-Ir­(ppy)_3_ (*E*_IV_/_*III_ = −1.73 V vs SCE in MeCN)^15^ under purple LEDs (λ_max_ = 390 nm) in DMSO at room temperature under Ar for 18 h. To our delight, the proposed [4 + 2]/[4 + 2] product **3a** was obtained in 60% isolated yield (entry 1). Solvent screening confirmed DMSO as optimal (entries 1–5), while alternative PCs—including [Ru­(bpy)_3_Cl_2_]·6H_2_O, Eosin Y, or tetraphenylporphyrin (TPP)—did not improve yields (entries 6–8). Considering the deprotonation step in the proposed mechanism presented in Scheme 2, we evaluated the effect of external bases: NaHCO_3_ led to a messy mixture, whereas 2,4,6-collidine afforded **3a** in 57% yield (entries 9–10). Reducing PC loading from 3 to 1 mol % had minimal impact (entry 11). White light irradiation (23 W CFL) increased the yield to 62% and was adopted as the optimal condition (entry 12). Control experiments highlighted key features of the reaction: exposure to air led to messy outcomes (entry 13), no product formed in the dark (entry 14), and only trace **3a** was observed in the absence of PC under 23 W CFL (entry 15). Notably, **3a** was obtained in 49% yield under purple LEDs without PC (entry 16), likely due to the formation of photoactive electron donor–acceptor (EDA) complexes.^16^ More optimization studies are summarized in Tables S1–S2.

**Table 1:**
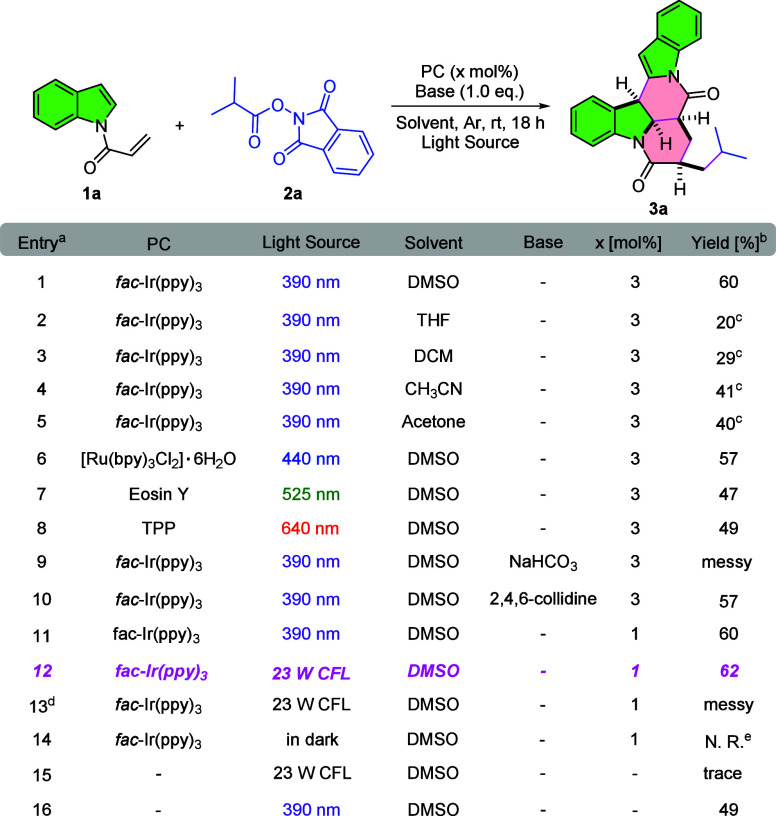
Selected Optimization of Reaction Conditions

a The reaction was conducted with **1a** (0.3 mmol), **2a** (0.1 mmol) and PC (x mol %) in solvent (1.0 mL).

b Isolated yield.

c NMR yield by using trimethoxybenzene (0.1 mmol) as the internal standard.

d Open to air.

e No reaction.

With the optimized conditions in hand, we next evaluated the generality of the reaction (Table 2). We first explored the scope of NHPI esters **2** using **1a** as a model substrate (Table 2a). The reaction proceeded smoothly, affording DHPIs **3a**–**3p** in 14–71% yield. A broad spectrum of functional groups is well-tolerated, including aryl (**3h**, **3o**), 2-thienyl (**3i**), chloride (**3j**), ketone (**3k**), ester (**3l**), ether (**3m**–**3n**), and Boc-protected amine ester (**3p**). All NHPI ester substrates **2** produced a single diastereomer, except the chiral aspartic acid-derived ester **2p**, which gave the corresponding DHPI **3p** in 26% yield with a 1.3:1 d.r. value. The relative configurations of **3a** and **3j** were confirmed by X-ray crystallography (CCDC 2465195 and 2465294; see Supporting Information), showing the same stereochemical arrangement. Notably, several low-yield substrates exhibited drastic improvement under purple LEDs (λ_max_ = 390 nm): **3b** (14 → 48%), **3l** (43 → 54%), and **3p** (26 → 51%). Next, we examined the scope of simple N-acryloyl indoles **1** utilizing **2e** as a model substrate (Table 2b). Substituents (R^1^ or R^2^) at C3–C7 positions reacted efficiently with **2e**, furnishing an array of functionalized DHPIs **3q**–**3ae** in 23–82% yield. Both electron-donating and electron-withdrawing groups (EWGs) were compatible. Nevertheless, products **3** with strong EWGs, such as CF_3_ (**3aa**), CO_2_Me (**3ab**), and F (**3ad**), were formed in slightly lower yields, presumably due to the less favored second SET process associated with formation of the less stable benzylic carbocation intermediate **VI**. To our delight, these yields also improved under purple light irradiation (**3aa**: 37 → 43%; **3ab**: 23 → 49%; and **3ad**: 44 → 48%). Interestingly, when substrate **1p** bearing 5-methoxy and 7-methyl groups was tested, the desired product **3ae** was isolated in 43% yield, along with the [4 + 2] byproduct **3ae′** in 10% yield (Table 2c). The isolation of **3ae′** further supports the mechanism proposed in Scheme 2, and its yield increased to 25% under purple light irradiation. It is worth noting that although [4 + 2] byproducts were observed for most substrates, their low formation levels and the presence of *cis*/*trans* isomers allowed isolation only of **3ae′**. These observations help explain the generally moderate yields of **3**. Finally, the protocol was ineffective for certain substrates (Table 2d). Product **3af** was isolated but could not be characterized by NMR due to poor solubility, although HRMS confirmed its structure (Figure S1). Products **3ag**–**3ah** and **3aj**–**3ak** were not detected, and only trace **3ai** was observed. The failure to form **3ag**–**3ah** is presumably due either to direct quenching of *fac*-Ir­(ppy)_3_ by the NO_2_ group, as evidenced by the observation of unreacted **2e**, or to an unfavored second SET process. Additional substrate scope investigations are summarized in Tables S3–S4 and Schemes S1–S3.

**Table 2:**
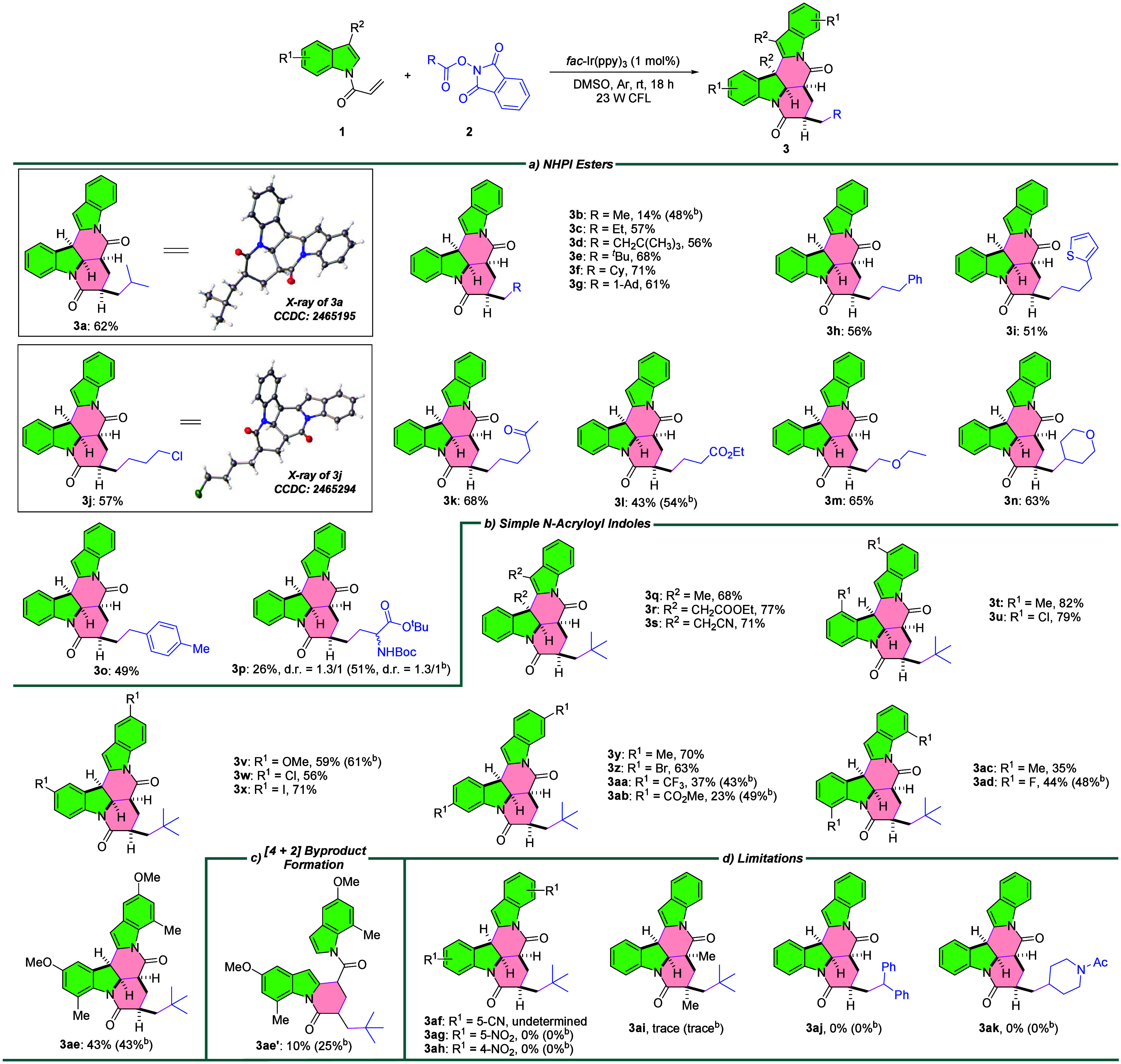
Scope of Simple N-Acryloyl Indoles and NHPI Esters^a^

a Reactions were conducted on a 0.1 mmol scale. Isolated yields.

b Purple LEDs (λ_max_ = 390 nm) were used.

To showcase the synthetic utility of this approach, several additional experiments were performed (Scheme 3). First, diethyl bromomalonate **4a** and 2-bromoacetophenone **4b** were investigated as radical precursors to react with **1a** under the standard white light conditions, respectively. Unfortunately, both reactions provided messy results (Scheme S4). Given that deprotonation is required for product formation, 1.0 equiv of NaHCO_3_ was added under the above optimal conditions. Delightfully, **5a** and **5b** were obtained smoothly, with yields increased to 50% and 41% under blue light (λ_max_ = 440 nm) irradiation (Scheme 3a). Next, the reaction was successfully scaled up to 4.2 mmol, albeit with slightly reduced efficiency, affording **3e** in 46% yield (Scheme 3b). Finally, several derivatizations of product **3a** were explored (Scheme 3c). Reduction of both amide groups with BH_3_·Me_2_S furnished **6a** in 53% yield,^17^ whereas treatment with LiAlH_4_ delivered the selectively reduced product **6b** in 78% yield as a single diastereomer (see mechanism in Scheme S5).^18^ Although the relative configuration of **6b** was not determined at this stage, it could be further converted to **6c** via reaction with TsCl in the presence of triethylamine. Collectively, these results underscore the versatility, practicality, and robustness of this approach.

**Scheme 3:**
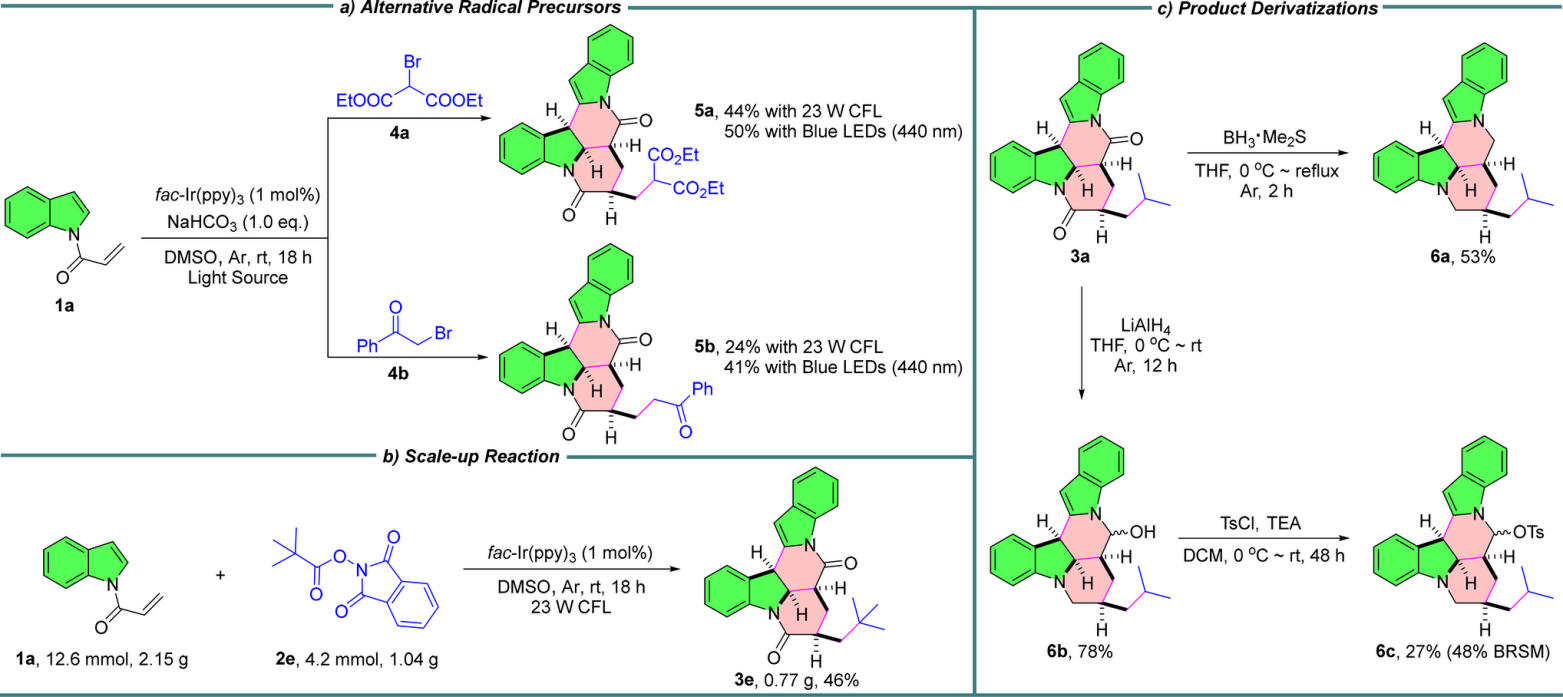
Synthetic Applications

To gain further insight into the reaction mechanism, several control experiments were conducted. Radical trapping and light/dark interval experiments support the proposed mechanism in Scheme 2 (Schemes S6–S7). Notably, the formation of **3a**, **3b**, **3p**, and **3ab** in the absence of PC under purple light suggests that an EDA-complex-driven pathway is also possible (Table S5). This alternative pathway could account for the increased yields observed for certain products (e.g., **3b**, **3p**, **3aa**, and **3ab**) indicated in Table 2. A detailed EDA-complex-driven mechanism is proposed in Scheme S8. The observed diastereoselectivity can also be rationalized by two proposed mechanistic models involving the key intermediate **IV** (Scheme S9).

In summary, we have developed a novel visible-light-mediated radical cascade [4 + 2]/[4 + 2] cycloaddition that uncovers previously unobserved reactivity of simple *N*-acryloyl indoles. This approach enables the efficient one-pot construction of structurally complex DHPIs with diverse functional groups, representing a significant advance over previously reported methods in three key aspects. First, it is compatible with two common classes of radical precursors: NHPI esters and α-bromocarbonyl compounds. Second, it forges four C–C bonds and induces dearomatization of one indole ring during the radical addition sequence. Third and most importantly, two *N*-acryloyl indole molecules are incorporated into the DHPI products. In addition, the observed [4 + 2] byproducts and mechanistic studies provide insight into the reaction pathways. The generally moderate yields are likely attributable to the formation of multiple reactive radical intermediates, which result in undetermined byproducts. The scale-up reaction and downstream transformations further highlight the synthetic utility of this methodology. Ongoing efforts aim to engage simple *N*-acryloyl indoles with other radical precursors under photocatalytic, electrocatalytic, or oxidant-induced conditions.

## Supplementary Material



## Data Availability

The data underlying this study are available in the published article and its Supporting Information.

## Notes

Experimental procedures, characterization, X-ray crystallographic data, and NMR spectra (PDF)
