# Supplementary material for: Visible-Light-Induced Radical Cascade [4 + 2]/[4 + 2] Cycloaddition of Underexplored N‑Acryloyl Indoles To Access Dihydropyrido[1,2‑a]‑indolones
Source: Org Lett. 2026 Feb 23;28(9):2852–8. doi: 10.1021/acs.orglett.5c05421 (PMC12973294; doi:10.1021/acs.orglett.5c05421)
Supplement: Supplementary file 1 [file ol5c05421_si_001.pdf]

Supporting Information

Visible-Light-Induced Radical Cascade [4 + 2]/[4 + 2] Cycloaddition of Underexplored N-Acryloyl Indoles to Access Dihydropyrido[1,2-*a*]-indolones

Cody Bishir[†]*, Samuel Milton*[†]*, Abbey Hubbard, Michael Indalsingh, Zainah Abufouz, and Liangyong Mei**

Department of Chemistry and Biochemistry, University of North Florida,
Jacksonville, Florida 32224, United States
l.mei@unf.edu

CONTENTS

1. General remarks……………………………………………………………………..S2
2. Spectrum graphs for KSPR160L–LEDs……………………………………………..S3
3. Reaction setup for photocatalysis..…………………………………………………..S4
4. General procedure for the synthesis of simple N-acryloyl indoles **1**…………………....S5
5. General procedure for the synthesis of N-hydroxyphthalimide (NHPI) esters **2**………...S7
6. Typical procedure for the synthesis of dihydropyrido[1,2-*a*]-indolones (DHPIs) **3** and **5** via visible-light-mediated radical cascade [4 + 2]/[4 + 2] cycloaddition…………………..S8
7. Optimization of visible-light-mediated radical cascade [4 + 2]/[4 + 2] cycloaddition of simple N-acryloyl indoles and NHPI esters…………………………………………….......S10
8. Substrate scope of purple-light-mediated radical cascade [4 + 2]/[4 + 2] cycloaddition of simple N-acryloyl indoles and NHPI esters……………………………………………..S12
9. Unsuccessful substrates for photo-mediated radical cascade [4 + 2]/[4 + 2] cycloaddition of simple N-acryloyl indoles and NHPI esters...S13
10. Examination of α-bromocarbonyl compounds under the standard photo-mediated radical cascade [4 + 2]/[4 + 2] cycloaddition conditions………………………………… S16
11. Experimental procedure for the scale-up reaction and transformations…………… S17
12. Mechanistic studies……………………………………………………………….S20
13. Characterization for simple N-acryloyl indoles **1** and NHPI esters **2**………………..S24
14. Characterization for DHPI products **3**, **5** and compounds **6-7**……………………....S36
15. X-ray crystallographic information of compounds **3a** and **3j**…………………….......S55
16. NMR spectra charts for compounds **1-3** and **5-7**……………………………………..S58
17. References ………………………………………………………………………S138

1. General remarks

Unless otherwise specified, all reactions were started in oven-overnight-dried Schlenk flasks or tubes with a magnetic stir bar in an Ar filled glove box (MBRAUN LABstar pro), which were then stirred on the bench outside under an Ar atmosphere. ^{1}H, ^{13}C, and ^{19}F NMR spectra were recorded on a Bruker Ascend 500 MHz NMR spectrometers in $CDCl_3$ using TMS as the internal standard or the solvent signals as the standards. The chemical shifts are shown in δ scales (ppm). Multiplicities of ^{1}H NMR signals are designated as s (singlet), d (doublet), t (triplet), q (quartet), p (pentet), dd (doublet of doublets), ddd (doublet of doublets of doublets), m (multiplet), etc. Compounds were drawn by using ChemDraw 21.0.0 and NMR spectra were processed by using MestReNova. HRMS spectra were recorded with a Bruker Impact II mass spectrometer or Agilent 6230 time-of-flight TOF mass spectrometer. All chemicals and solvents were purchased from Sigma-Aldrich, Fisher Scientific, or Avantor. Anhydrous organic solvents purchased from Fisher Scientific were under N_2 atmosphere, which were opened and used in an Ar filled glove box only. Commercially obtained reagents were used without further purification. All reactions were monitored by TLC silica gel 60 F254 (EMD Millipore). R_f values are estimated. Flash column chromatography was carried out using SiliaFlash Irregular Silica Gels F60, 40-63 μm , 60 Å (SiliCycle) at increased pressure. An oil bath is used as the heat source when heating is required for the reaction.

Compounds **1a**,[1] **1t**,[2] **1u**,[3] **2**,[4-11] and **1′**[12] have been reported in the literature. Speficially, **2s** and **2t** were described in our previous work.[4]

2. Spectrum graphs for KSPR160L–LEDs

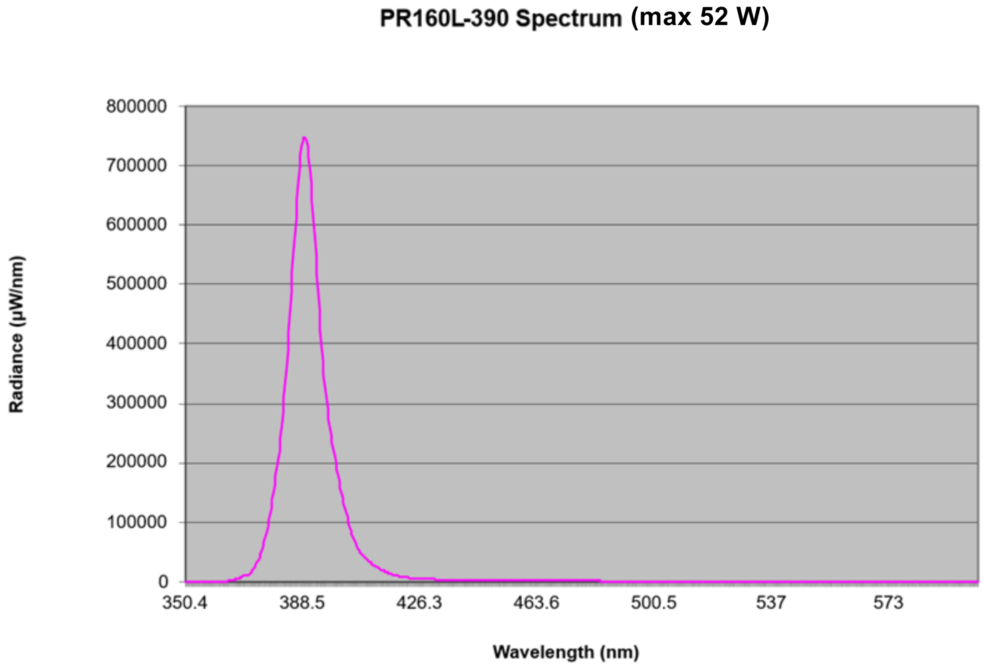

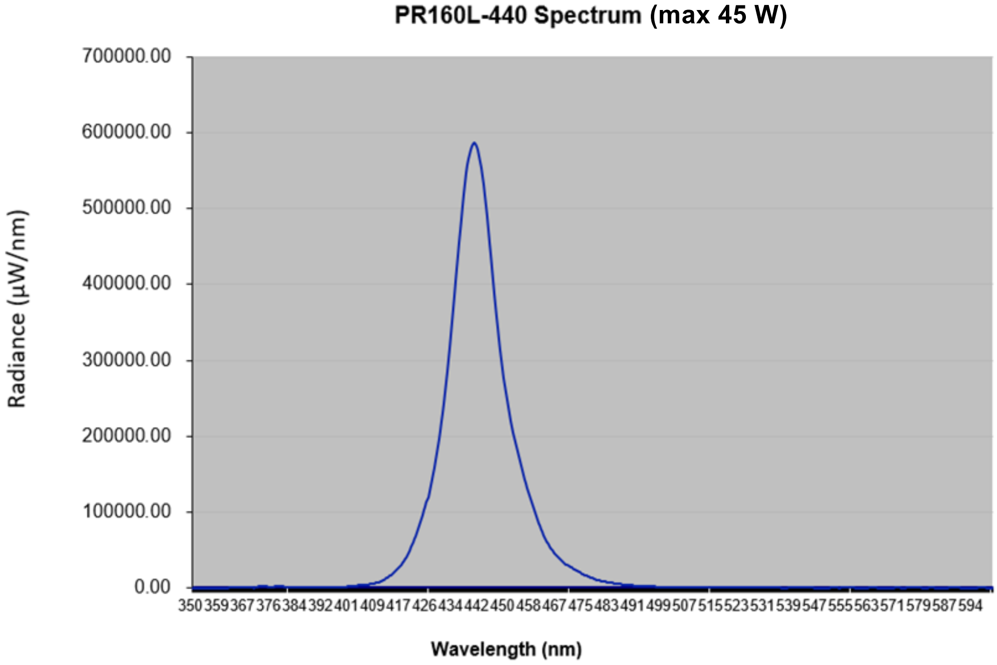

3. Reaction setup for photocatalysis

The reaction was set up as described by the the MacMillan group[13] and our previous work.[4] Two 25 mL Schlenk tubes are placed in a water bath (Kemtech crystallizing dish) at the center of a magnetic stirrer. Two parallel 23 W CFL (Philips EL/mdTQS T2), Purple LED lamps (KSPR160L-390 nm, Purple LED, Kessil LED Lights), or Blue LED lamps (KSPR160L-440 nm, Blue LED, Kessil LED Lights) are placed perpendicular to the side of the two Schlenk tubes, so that they are equally exposed to the lights. The magnetic stirrer/water bath/LED lamps are surrounded by a cardboard box covered with aluminum foil to increase the light reflections. A small fan near the water bath is always on when the reaction is running. The combination of the water bath and fan is to offset the heat generated from the LED lamps and keep the reaction around room temperature (21-23 °C). Distance of fan to water bath ≈ 20 cm. Distance of LED lamps to Schlenk tubes ≈ 7 cm.

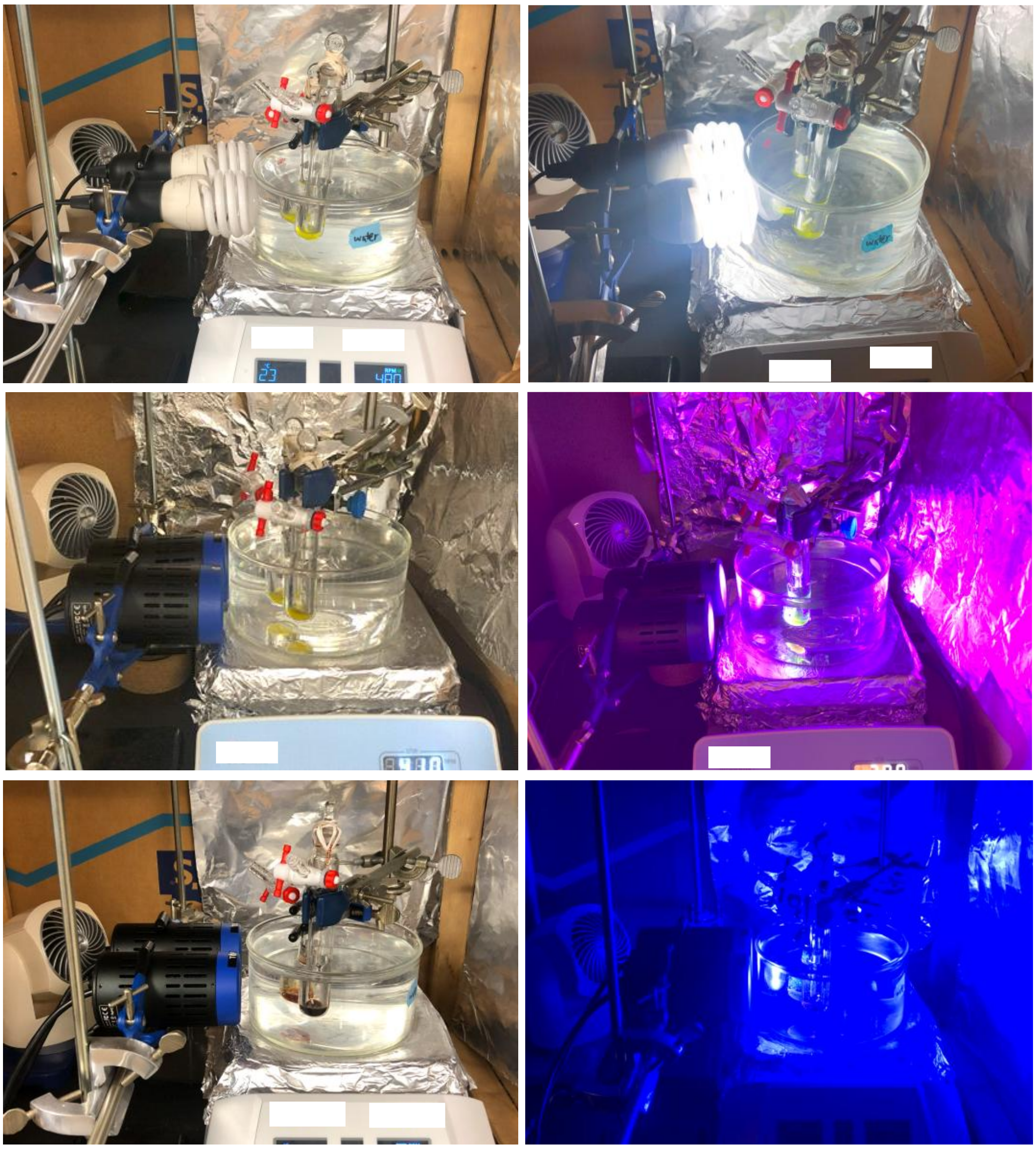

4. General procedure for the synthesis of simple N-acryloyl indoles 1

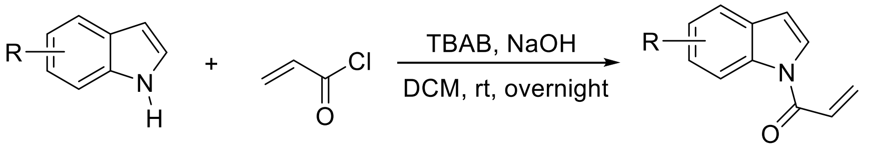

1a, R = H, 62% yield
1b, R = 3-Me, 18% yield
1c, R = 3-CH_2COOEt, 51% yield
1d, R = 3-CH_2CN, 50% yield
1e, R = 4-Me, 31% yield
1f, R = 4-Cl, 71% yield
1g, R = 5-MeO, 46% yield
1h, R = 5-Cl, 47% yield
1i, R = 5-I, 34% yield
1j, R = 6-Me, 11% yield
1k, R = 6-Br, 70% yield
1l, R = 6-CF_3, 43% yield
1m, R = 6-COOMe, 67% yield
1n, R = 7-Me, 5% yield
1o, R = 7-F, 37% yield
1p, R = 5-MeO & 7-Me, 7% yield
1q, R = 5-CN, 21% yield
1r, R = 5-NO_2, 8% yield
1s, R = 4-NO_2, 39% yield
1t, R = 2-Me, 8% yield

To a solution of indole (1.0 eq.) in dry DCM (0.33 M) was added $^{n}Bu_4NBr$ (TBAB, 0.1 eq.) and NaOH (3.0 eq.) at room temperature. After stirring for 1 h, a solution of acryloyl chloride (2.5 eq.) in dry DCM (2.5 M) was added dropwisely. The reaction mixture was stirred at rt overnight and then quenched with H_2O. The layers were separated and the aqueous layer was extracted with DCM (2x). The combined organic layer was washed with 1 M NaOH (2x), brine, dried over Na_2SO_4, and concentrated under reduced pressure on a RotaVap. The crude product was purified by flash chromatography (FC) on silica gel (eluent: Hexanes/EtOAc or Hexanes/EtOAc/DCM) to yield the desired N-acryloyl indole products **1** as a solid or oil. *For some of them, an additional recrystallization in Et_2O/Hexanes was conducted for further purification.*

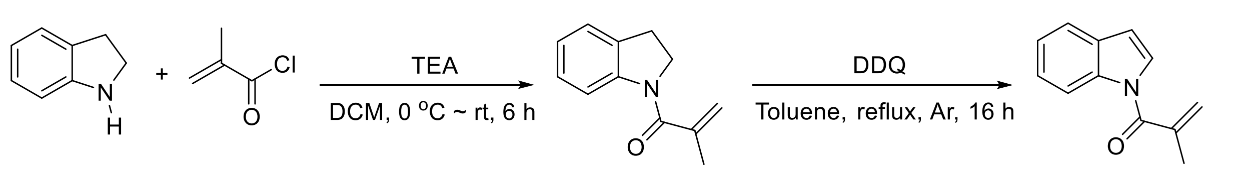

1u, 52% for 2 steps

Following a modified procedure from the literature.[3]

To a solution of indoline (1.0 eq.) in dry DCM (0.33 M) was added triethylamine (1.0 eq.) in one portion at 0 °C, followed by addition of methylacryloyl chloride (1.0 eq.) dropwisely. The reaction mixture was warmed to room temperature and stirred for 6 hours, which was then quenched with H_2O. The layers were separated and the aqueous layer was extracted with DCM (3x). The combined organic layer was dried over Na_2SO_4 and concentrated under reduced pressure on a RotaVap. The

crude product was purified by flash chromatography (FC) on silica gel (eluent: Hexanes/EtOAc = 10/1) to yield the desired N-acryloyl indoline product as a white solid.

To a solution of the above N-acryloyl indoline (1.0 eq.) in dry toluene (0.25 M) in a three-neck round-bottom flask equipped with a reflux condenser was added DDQ (1.3 eq.) at room temperature under Ar. The reaction mixture was heated to reflux in an oil bath and stirred for 16 hours. The reaction was then cooled to room temperature, diluted with EtOAc, and washed with water. The layers were separated and the aqueous layer was extracted with EtOAc (2x). The combined organic layer was dried over Na_2SO_4 and concentrated under reduced pressure on a RotaVap. The crude product was purified by flash chromatography (FC) on silica gel (eluent: Hexanes/EtOAc = 30/1) to yield the desired N-acryloyl indoline product **1u** as a brown oil.

5. General procedure for the synthesis of N-hydroxyphthalimide (NHPI) esters 2

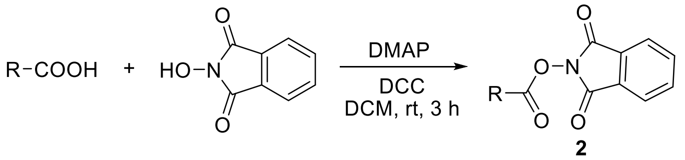

2

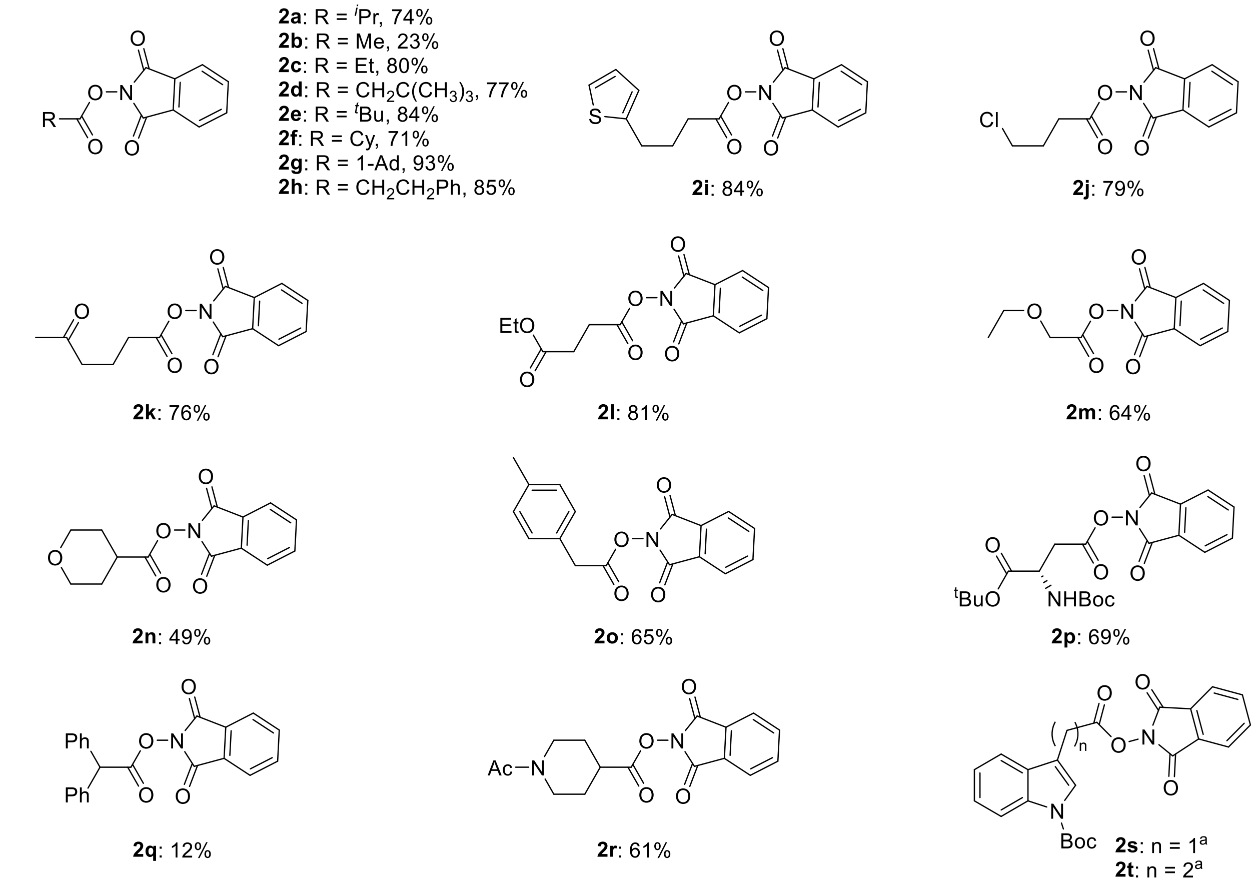

2a: R = ^{i}Pr, 74%
2b: R = Me, 23%
2c: R = Et, 80%
2d: R = $CH_2C(CH_3)_3$, 77%
2e: R = ^{t}Bu, 84%
2f: R = Cy, 71%
2g: R = 1-Ad, 93%
2h: R = CH_2CH_2Ph, 85%

2i: 84%

2j: 79%

2k: 76%

2l: 81%

2m: 64%

2n: 49%

2o: 65%

2p: 69%

2q: 12%

2r: 61%

2s: n = 1[a]
2t: n = 2[a]

[a] See our previous report in reference 4

Following our previously reported procedure.[4]

To a solution of carboxylic acid (1.0 eq.) in DCM (0.2 M) was added N-hydroxyphthalimide (NHPI) (1.2 eq.), DMAP (10 mmol%) and DCC (1.2 eq.) at room temperature. After stirring for 3 h, the precipitates in the reaction mixture were filtered through celite and the filtrate was concentrated under reduced pressure on a RotaVap. The crude product was purified by flash column chromatography (FC) on silica gel (eluent: Hexanes/EtOAc) to yield the desired redox-active NHPI esters **2** as a solid.

6. Typical procedure for the synthesis of dihydropyrido[1,2-*a*]-indolones (DHPIs) 3 and 5 via visible-light-mediated radical cascade [4 + 2]/[4 + 2] cycloaddition

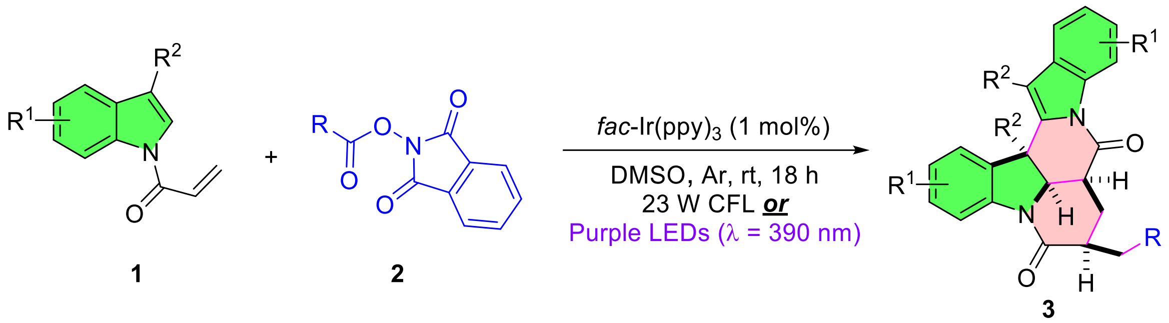

In an Ar glove box, the NHPI ester substrate **2** (0.1 mmol, 1.0 eq.) and *fac*-Ir(ppy)$_3$ (0.001 mmol, 1 mol%) were added to an oven-dried (overnight) Schlenk tube containing a stirring bar, followed by adding anhydrous DMSO (1.0 mL) and simple N-acryloyl indole substrate **1** (0.3 mmol, 3.0 eq.). The Schlenk tube was then sealed, removed from the glove box, and the mixture was stirred at room temperature under 23 W CFL or purple LEDs (λ_{max} = 390 nm) irradiation. After 18 hours, the reaction mixture was monitored by TLC. The crude product was purified by flash chromatography (FC) on silica gel (eluent: Hexanes/EtOAc) to yield the desired pure DHPI products **3**.

For some of DHPI products 3, an additional washing step was conducted after FC for their further purifications. **3** was dissolved in DCM (5.0 mL), followed by adding 1 M NaOH aq. (5.0 mL). The mixture was stirred at rt for 15 minutes. The layers were separated and the aqueous layer was extracted with DCM (5.0 mL). The combined organic layer was washed with brine and dried over Na_2SO_4. After removing DCM under reduced pressure on a RotaVap, the pure **3** was obtained.

a)

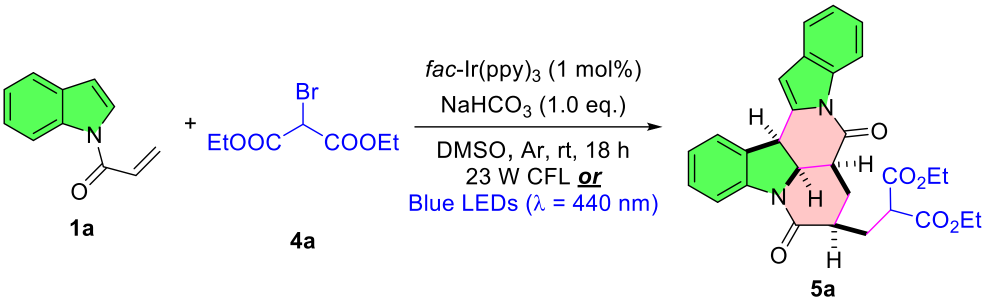

b)

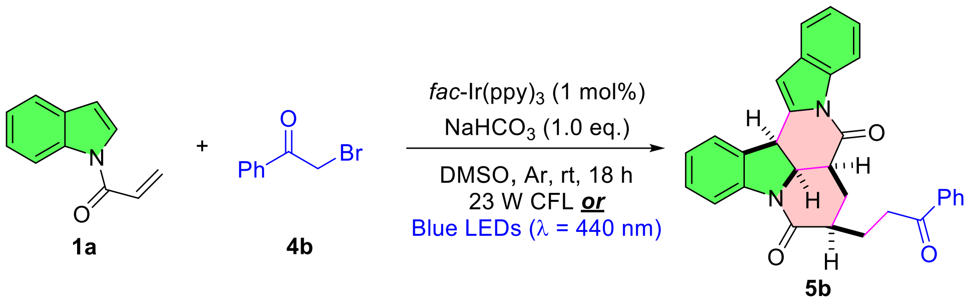

In an Ar glove box, the radical precursor **4** (0.1 mmol, 1.0 eq.) and *fac*-Ir(ppy)$_3$ (0.001 mmol, 1 mol%) were added to an oven-dried (overnight) Schlenk tube containing a stirring bar, followed by adding anhydrous DMSO (1.0 mL), $NaHCO_3$ (0.1 mmol, 1.0 eq.), and simple N-acryloyl indole substrate **1** (0.3 mmol, 3.0 eq.). The Schlenk tube was then sealed, removed from the glove box, and the mixture was stirred at room temperature under under 23 W CFL or blue LEDs (λ_{max} = 440 nm) irradiation. After 18 hours, the reaction mixture was monitored by TLC. The crude product was purified by flash chromatography (FC) on silica gel (eluent: Hexanes/EtOAc) to yield the desired product **5**.

7. Optimization of visible-light-mediated radical cascade [4 + 2]/[4 + 2] cycloaddition of simple N-acryloyl indoles and NHPI esters

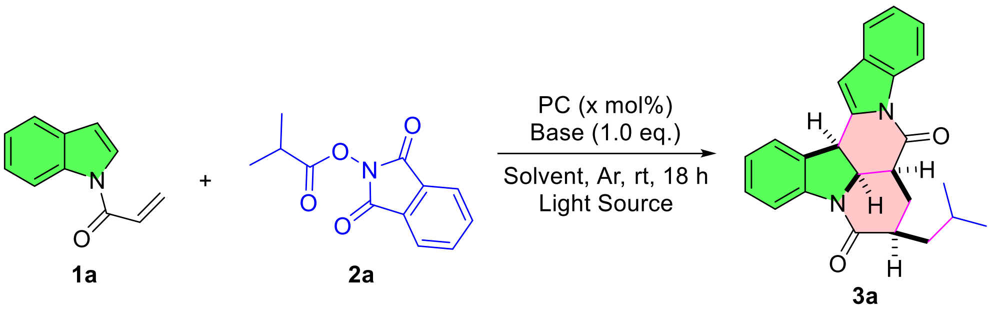

Entry[a]	PC	Light Source	Solvent	Base	x [mol%]	Yield [%][b]
1	*fac*-Ir(ppy)$_3$	390 nm	DMSO	-	3	60
2	*fac*-Ir(ppy)$_3$	390 nm	THF	-	3	20[c]
3	*fac*-Ir(ppy)$_3$	390 nm	DCM	-	3	29[c]
4	*fac*-Ir(ppy)$_3$	390 nm	EtOH	-	3	28[c]
5	*fac*-Ir(ppy)$_3$	390 nm	CH_3CN	-	3	41[c]
6	*fac*-Ir(ppy)$_3$	390 nm	DMF	-	3	44
7	*fac*-Ir(ppy)$_3$	390 nm	Acetone	-	3	40[c]
8	*fac*-Ir(ppy)$_3$	390 nm	CH_3NO_2	-	3	N. R.[d]
9	[Ru(bpy)$_3$Cl$_2$]•6H$_2$O	440 nm	DMSO	-	3	57
10	4-CzIPN	440 nm	DMSO	-	3	52
11	Rose Bengal	525 nm	DMSO	-	3	44
12	Eosin Y	525 nm	DMSO	-	3	47
13	TPP	640 nm	DMSO	-	3	49
14	[Ir(dtbbpy)(ppy)$_2$][PF$_6$]	440 nm	DMSO	-	3	49
15	*fac*-Ir(ppy)$_3$	390 nm	DMSO	$NaHCO_3$	3	messy
16	*fac*-Ir(ppy)$_3$	390 nm	DMSO	2,4,6-collidine	3	57
17	*fac*-Ir(ppy)$_3$	390 nm	DMSO	$KHCO_3$	3	messy
18	*fac*-Ir(ppy)$_3$	390 nm	DMSO	pyridine	3	57
19	*fac*-Ir(ppy)$_3$	390 nm	DMSO	Na_2CO_3	3	messy
20	*fac*-Ir(ppy)$_3$	390 nm	DMSO	DIPEA	3	messy
21	fac-Ir(ppy)$_3$	390 nm	DMSO	-	1	60
22	***fac-Ir(ppy)$_3$***	***23 W CFL***	***DMSO***	***-***	***1***	***62***

[a] The reaction was conducted with **1a** (0.3 mmol), **2a** (0.1 mmol) and PC (x mol%) in solvent (1.0 mL).
[b] Isolated yield. [c] NMR yield by using trimethoxybenzene (0.1 mmol) as the internal standard. [d] No reaction.

Table S1. Optimization of visible-light-mediated radical cascade [4 + 2]/[4 + 2] cycloaddition of simple N-acryloyl indoles and NHPI esters

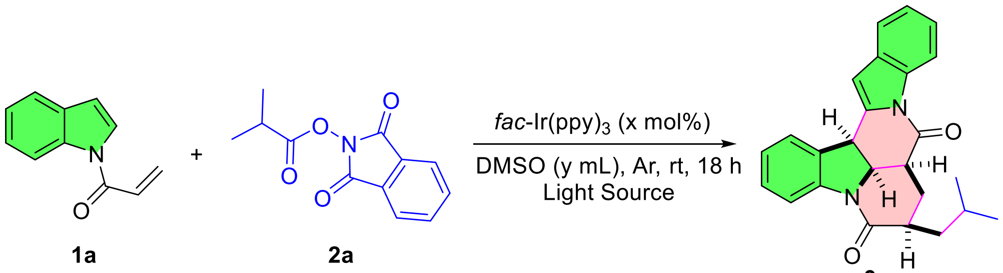

1a **2a** **3a**

Entry[a]	PC	Light Source	x [mol%]	y [mL]	Yield [%][b]
1	*fac*-Ir(ppy)$_3$	390 nm	3	1.0	60
2[c]	*fac*-Ir(ppy)$_3$	390 nm	3	1.0	44
3	*fac*-Ir(ppy)$_3$	390 nm	3	0.5	60
4	*fac*-Ir(ppy)$_3$	390 nm	3	2.0	55
5[d]	*fac*-Ir(ppy)$_3$	390 nm	3	1.0	60
6	fac-Ir(ppy)$_3$	390 nm	5	1.0	55
7	*fac*-Ir(ppy)$_3$	390 nm	1	1.0	60
8	***fac-Ir(ppy)$_3$***	***23 W CFL***	***1***	***1.0***	***62***
9	-	390 nm	-	1.0	49
10	-	440 nm	-	1.0	25
11	-	525 nm	-	1.0	trace
12	-	640 nm	-	1.0	N. R.[e]
13	-	23 W CFL	-	1.0	trace
14	*fac*-Ir(ppy)$_3$	in dark	1	1.0	N. R.[e]
15[f]	*fac*-Ir(ppy)$_3$	23 W CFL	1	1.0	messy
16[f]	*fac*-Ir(ppy)$_3$	390 nm	1	1.0	messy

[a] The reaction was conducted with **1a** (0.3 mmol), **2a** (0.1 mmol) and PC (x mol%) in DMSO (y mL). [b] Isolated yield. [c] 0.2 mmol of **1a** and 0.2 mmol of **2a** were used. [d] 0.4 mmol of **1a** was used. [e] No reaction. [f] Open to air.

Table S2. Further optimization of visible-light-mediated radical cascade [4 + 2]/[4 + 2] cycloaddition of simple N-acryloyl indoles and NHPI esters

8. Substrate scope of purple-light-mediated radical cascade [4 + 2]/[4 + 2] cycloaddition of simple N-acryloyl indoles and NHPI esters

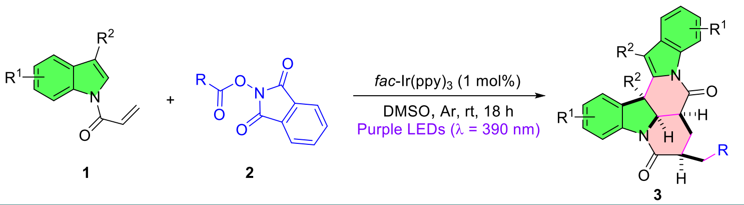

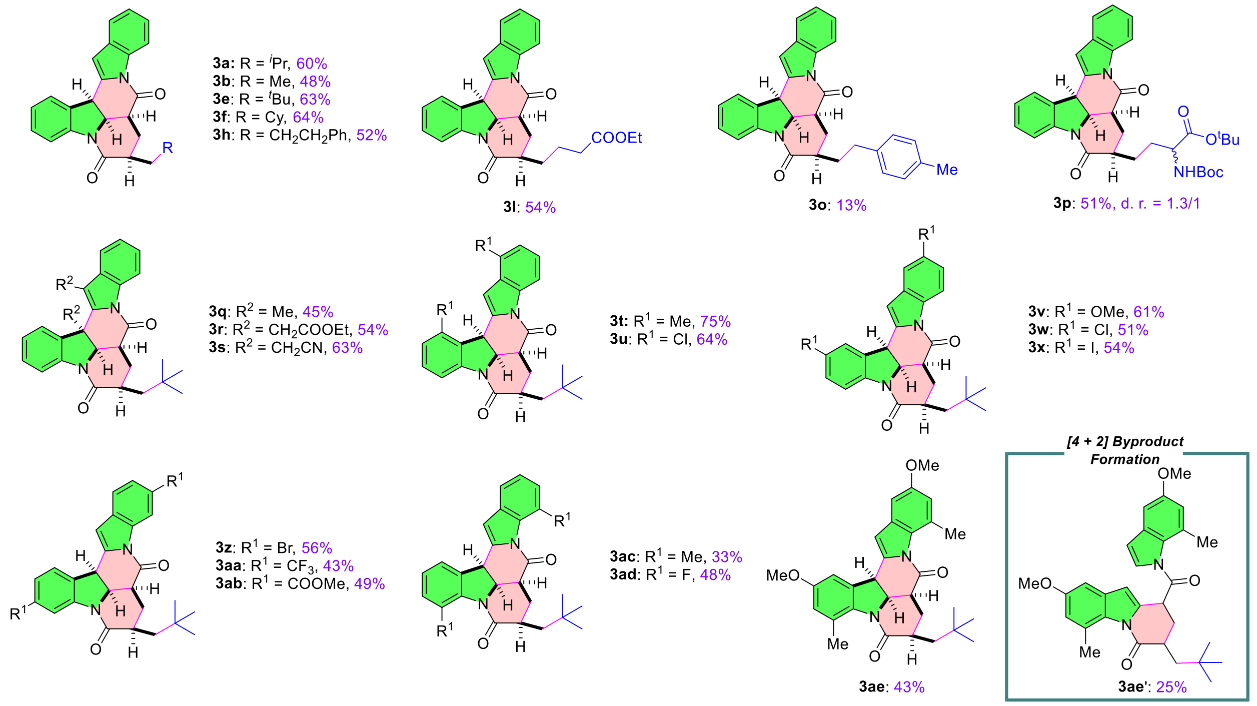

[a] The reaction was conducted with **1** (0.3 mmol), **2** (0.1 mmol) and *fac*-Ir(ppy)$_3$ (1 mol%) in DMSO (1.0 mL). Isolated yield.

Table S3. Substrate scope of purple-light-mediated radical cascade [4 + 2]/[4 + 2] cycloaddition of simple N-acryloyl indoles and NHPI esters

9. Unsuccessful substrates for photo-mediated radical cascade [4 + 2]/[4 + 2] cycloaddition of simple N-acryloyl indoles and NHPI esters

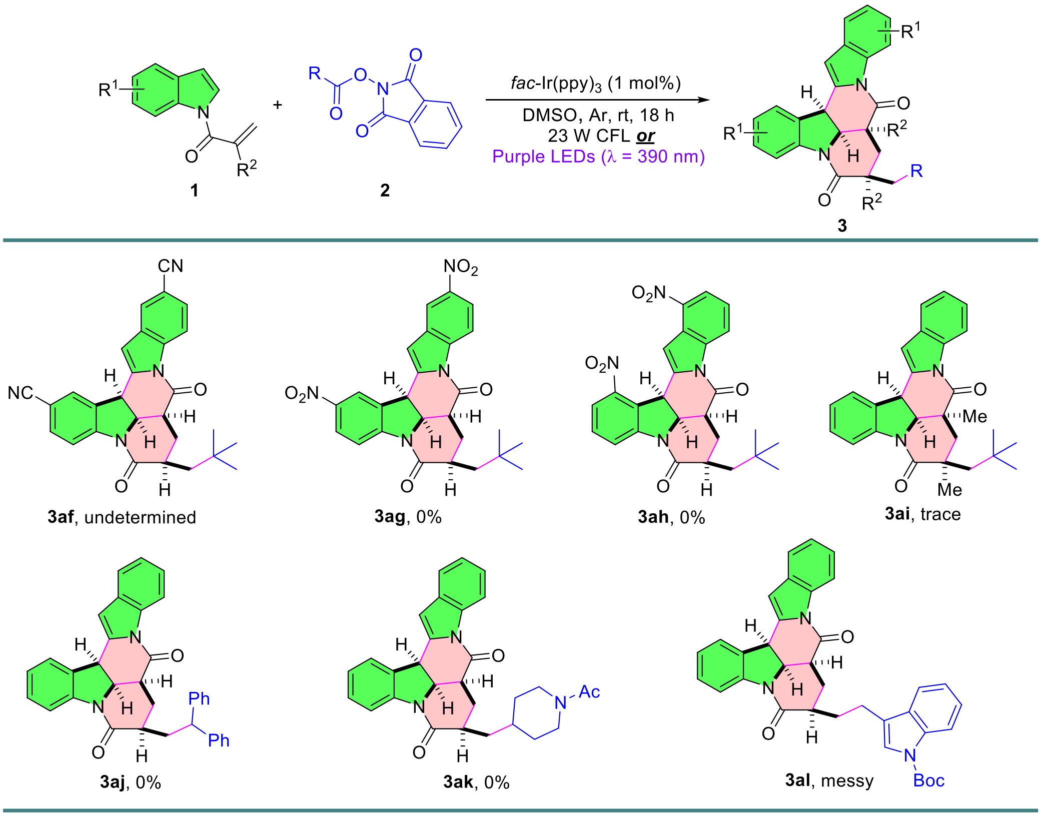

[a] The reaction was conducted with **1** (0.3 mmol), **2** (0.1 mmol) and *fac*-Ir(ppy)$_3$ (1 mol%) in DMSO (1.0 mL).

Table S4. Unsuccessful substrates for photo-mediated radical cascade [4 + 2]/[4 + 2] cycloaddition of simple N-acryloyl indoles and NHPI esters

Some substrates including **1q-1t** and **2q-2t** failed to provide the desired NHPI products **3** both under standard 23 W CFL and purple LEDs (λ_{max} = 390 nm) irradiation conditions (Table S3). Product **3af** was successfully detected and isolated when **1q** was treated with **2e** under either the standard 23 W CFL or purple light (λ_{max} = 390 nm) irradiation conditions. However, due to its poor solubility in commonly used deuterated solvents ($CDCl_3$, CD_2Cl_2, d_6-DMSO, d_6-acetone, CD_3CN, or CD_3OD), pure NMR data could not be acquired, although its HRMS data was consistent with the proposed structure (Figure S1). However, **3ag**, **3ah**, **3aj**, and **3ak** were not observed. A trace amount of **3ai** was observed. The reaction was messy when we targeted **3al** as the product. The failure to form **3ag–3ah** is presumably due either to direct quenching of *fac*-Ir(ppy)$_3$ by the NO_2 group, as evidenced by the observation of unreacted **2e**, or to an unfavored second SET process associated with formation of a very unstable benzylic carbocation intermediate **VI**.

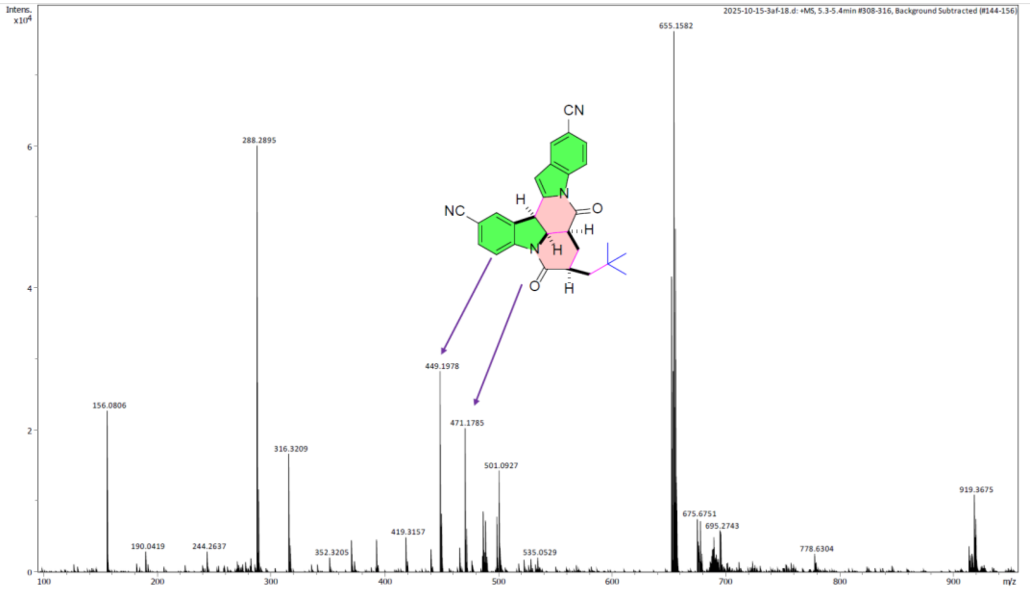

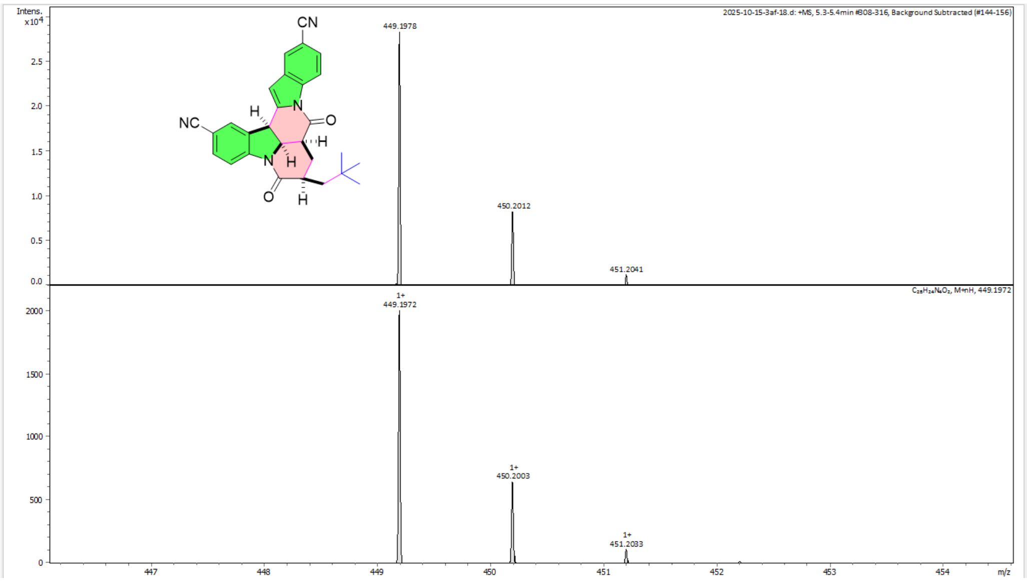

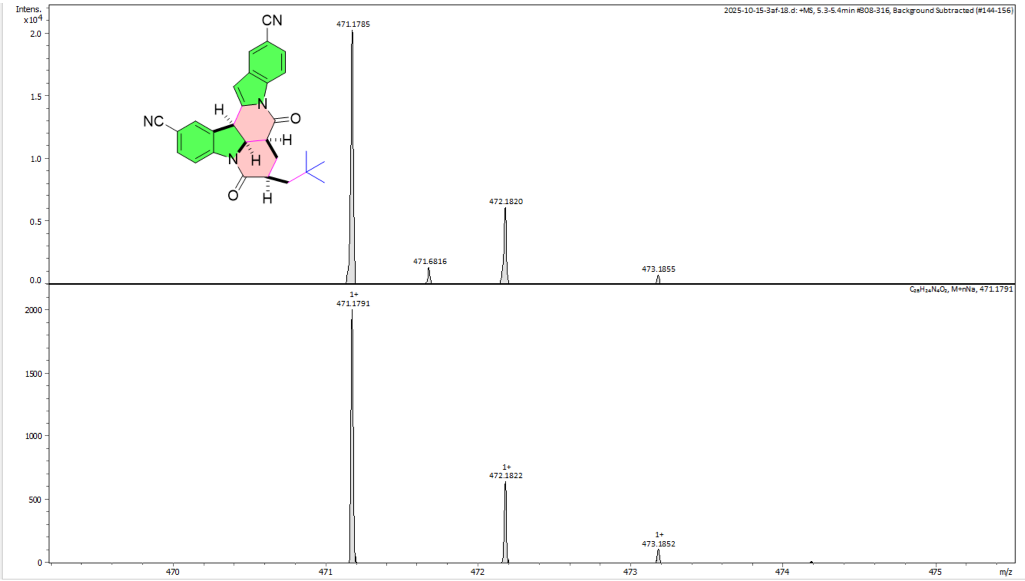

Figure S1. HRMS spectra of **3af**

When **1a** was treated with **2t** under standard 23 W CFL irradiation conditions, the desired product **3am** was not detected (Scheme S1). Instead, byproduct **7** was isolated in 51% yield, which was formed via a photo-induced radical [4+2] cycloaddition mechanism previously reported by our group.[4]

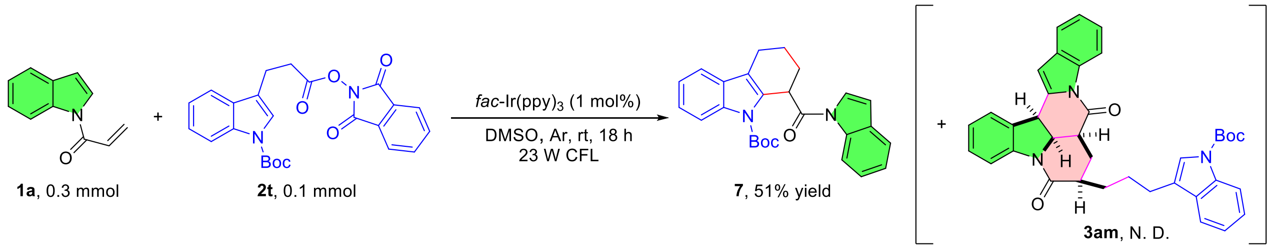

Scheme S1. Photo-mediated radical [4 + 2] cycloaddition of **1a** and **2t**

When **1t** was treated with **2e** under standard 23 W CFL or purple LEDs (λ_{max} = 390 nm) irradiation conditions, both reactions resulted in complex mixtures (Scheme S2). This outcome is expected, as the presence of a methyl group at the C-2 position of the indole ring inhibits the required deprotonation step in the proposed mechanism, thereby preventing formation of the desired [4 + 2]/[4 + 2] product or the [4 + 2] byproduct.

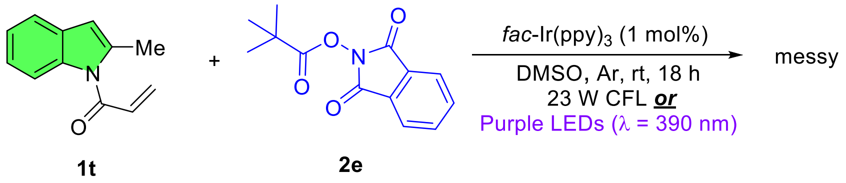

Scheme S2. Reaction between **1t** and **2e**

Additionally, N-vinylindole **1′** was synthesized by following the reported procedure.[12] Treatment of **1′** with **2e** under standard 23 W CFL or purple LEDs (λ_{max} = 390 nm) irradiation conditions led to complex reaction mixtures (Scheme S3).

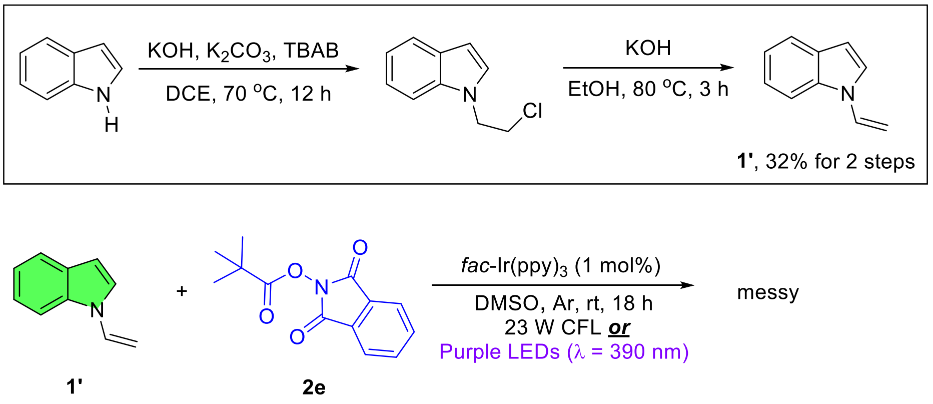

Scheme S3. Synthesis of **1′** and its reaction with **2e**

10. Examination of α-bromocarbonyl compounds under the standard photo-mediated radical cascade [4 + 2]/[4 + 2] cycloaddition conditions

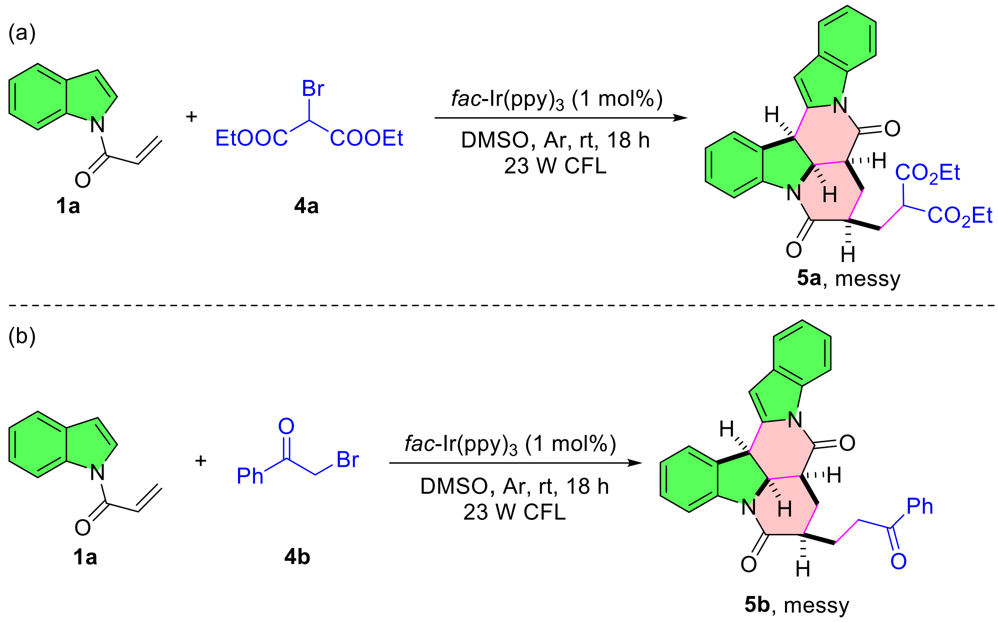

Scheme S4. Examination of α-bromocarbonyl compounds under the standard reaction conditions

11. Experimental procedure for the scale-up reaction and transformations

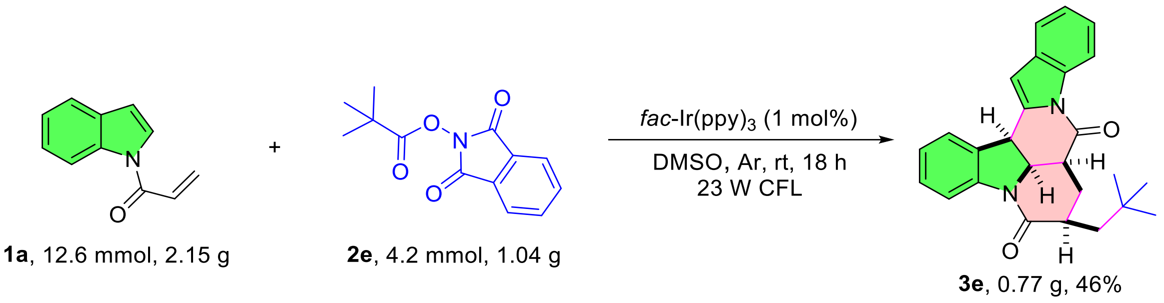

Scale-up reaction: In an Ar glove box, substrate **2e** (1.04 g, 4.2 mmol, 1.0 eq.) and *fac*-Ir(ppy)$_3$ (0.042 mmol, 1 mol%) were added to an oven-dried (overnight) Schlenk flask containing a stirring bar, followed by adding anhydrous DMSO (42.0 mL) and N-acryloyl indole substrate **1a** (2.15 g, 12.6 mmol, 3.0 eq.). The Schlenk flask was then sealed, removed from the glove box, and the mixture was stirred at room temperature under 23 W CFL irradiation for 18 hours. Water (30 mL) was added to the reaction mixture, which was then extracted with DCM (3x, 40 mL each). The combined organic layer was washed with water, 1 M NaOH aq., brine, and dried over anhydrous Na_2SO_4. After concentrating under reduced pressure on a RotaVap, the crude product was purified by flash column chromatography (FC) on silica gel (eluent: Hexanes/EtOAc = 8/1) to provide the desired DHPI product **3e** as a white solid (768 mg, 46% yield).

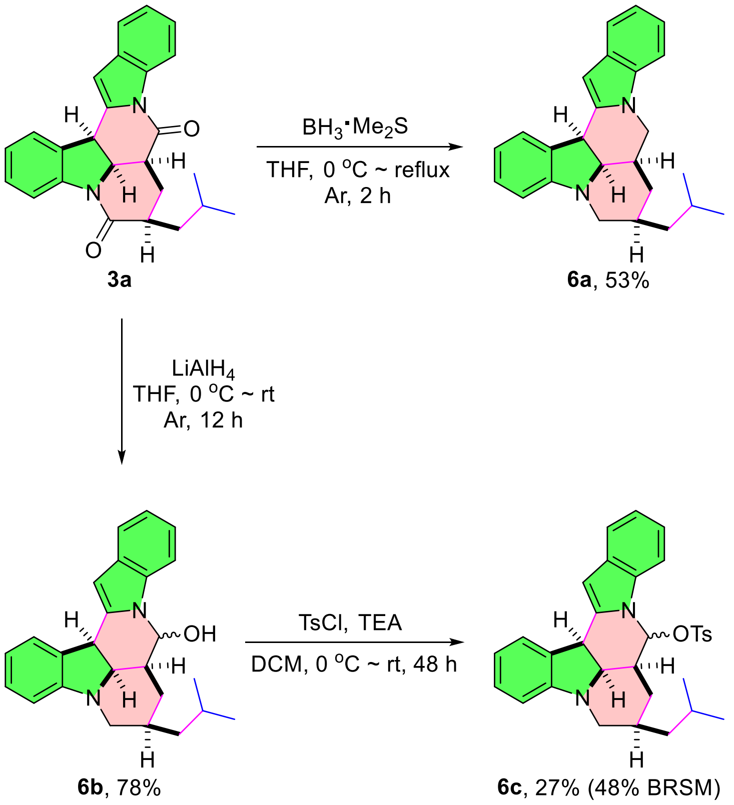

Synthesis of 6a: To a solution of **3a** (77 mg, 0.2 mmol, 1.0 eq.) in dry THF (1.5 mL) was added 2.0 M BH_3•Me_2S in THF solution (0.4 mL, 0.8 mmol, 4.0 eq.) dropwisely under Ar at 0 °C. The mixture was warmed to room temperature and heated under reflux in an oil bath for 2 hours. The reaction was quenched with MeOH slowly at 0 °C until no buddles releasing from the reaction mixture. The crude product was purified by flash column chromatography (FC) on silica gel (eluent: Hexanes/EtOAc = 25/1) to provide the desired product **6a** as a white solid (38 mg, 53% yield).

Synthesis of 6b: To a solution of $LiAlH_4$ (61 mg, 1.6 mmol, 8.0 eq.) in dry THF (1.0 mL) was added a solution of **3a** (77 mg, 0.2 mmol, 1.0 eq.) in dry THF (1.0 mL) dropwisely under Ar at 0 °C. The mixture was warmed to room temperature and stirred for 12 hours. The reaction was quenched with sat. NH_4Cl aq. slowly at 0 °C, which was followed by the addition of water. The solution was then extracted with EtOAc (3x). The combined organic layer was washed with brine and dried over anhydrous Na_2SO_4. After concentrating under reduced pressure on a RotaVap, the crude product was purified by flash column chromatography (FC) on silica gel (eluent: Hexanes/EtOAc = 2/1 ~ 1/1) to provide the desired product **6b** as a white solid (58 mg, 78% yield). It is worth noting that **6b** is a single diastereomer, although its relative configuration could not be determined at this stage by NMR analysis.

Synthesis of 6c: To a solution of **6b** (45 mg, 0.12 mmol, 1.0 eq.) in dry DCM (2.0 mL) was added TEA (14 mg, 0.14 mmol, 1.2 eq.) and TsCl (27 mg, 0.14 mmol, 1.2 eq.) at 0 °C. The mixture was warmed to room temperature and stirred for 48 hours. The crude product was purified by flash column chromatography (FC) on silica gel (eluent: Hexanes/EtOAc = 8/1 ~ 1/1) to provide the desired product **6c** as a white solid (17 mg, 27% yield) as well as the recovered **6b** (20 mg, 44% yield).

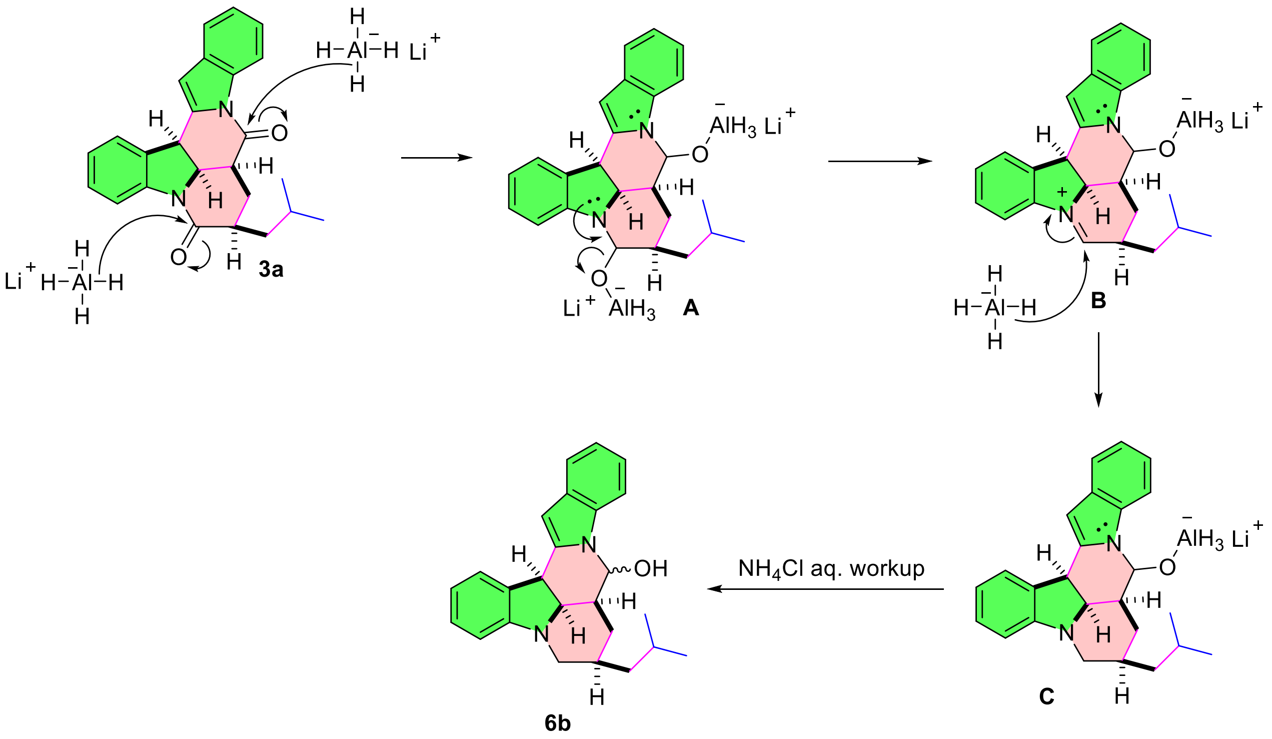

Scheme S5. Proposed mechanism for the formation of **6b**

To rationalize the formation of product **6b**, a simplified reaction mechanism was proposed for the selective reduction of **3a** (Scheme S5). First, nucleophilic addition of two hydrides to both carbonyl groups of **3a** generated tetrahedral intermediate **A**, converting the C=O oxygens into good leaving groups. Next, selective elimination of the oxygen derived from the indoline N1-amide in **A** produced the iminium ion intermediate **B**. No elimination occurs at the oxygen of the indole N1-amide due to the decreased nucleophilicity of the indole nitrogen, whose lone pair is delocalized within the 10π-aromatic system. Subsequently, hydride addition to the iminium ion reduced the indoline N1-amide C=O to CH_2, yielding intermediate **C**. Finally, quenching with saturated NH_4Cl aqueous solution protonated the intermediate **C** to afford the final product **6b**.

12. Mechanistic studies

Experimental procedure for the radical trapping experiments: In an Ar glove box, **2e** (0.1 mmol, 1.0 eq.) and *fac*-Ir(ppy)$_3$ (0.001 mmol, 1 mol% or 0.1 mmol, 1.0 eq.) were added to an oven-dried (overnight) Schlenk tube containing a stirring bar, followed by adding anhydrous DMSO (1.0 mL), **1a** (0.3 mmol, 3.0 eq.) and TEMPO (0.5 mmol, 5.0 eq.). The Schlenk tube was then sealed, removed from the glove box, and the mixture was stirred at room temperature under 23 W CFL irradiation. After 18 hours, the crude reaction mixture was monitored by both TLC and HRMS.

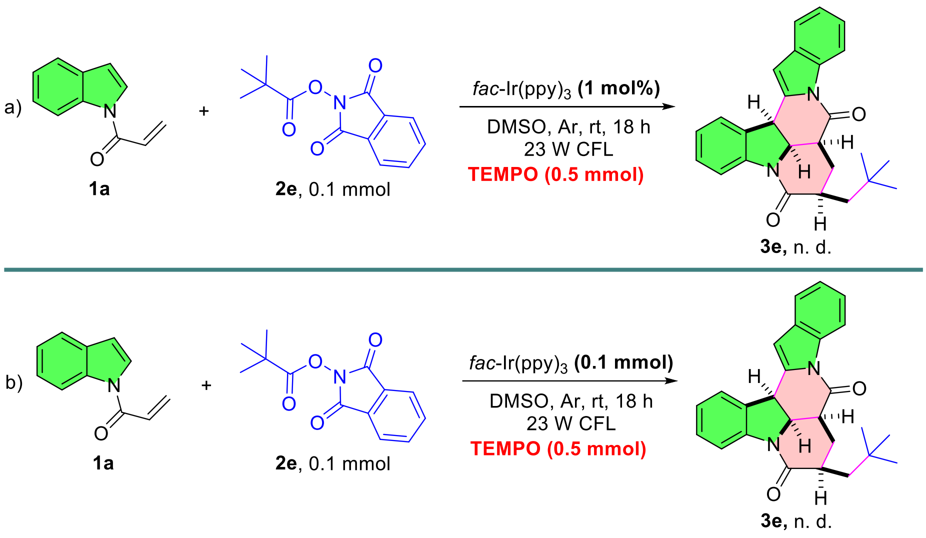

Scheme S6. Radical trapping experiments

As shown in Scheme S6, the formation of 3e was suppressed, supporting the involvement of radical intermediates. Unfortunately, no TEMPO-trapped adducts were detected by HRMS, which may be due to their formation in only trace amounts or their decomposition during analysis.

Experimental procedure for the light/dark interval experiment: In an Ar glove box, **2e** (0.05 mmol, 1.0 eq.) and *fac*-Ir(ppy)$_3$ (0.0005 mmol, 1 mol%) were added to an oven-dried (overnight) NMR tube, followed by adding degassed d_6-DMSO (0.5 mL), **1a** (0.15 mmol, 3.0 eq.) and trimethoxybenzene (0.05 mmol, 1.0 eq.). The NMR tube was then sealed, removed from the glove box, and the mixture was stirred at room temperature under 23 W CFL irradiation or in dark. After the indicated time, the reaction mixture was monitored by ^{1}H NMR to determine the yield of **3e** directly.

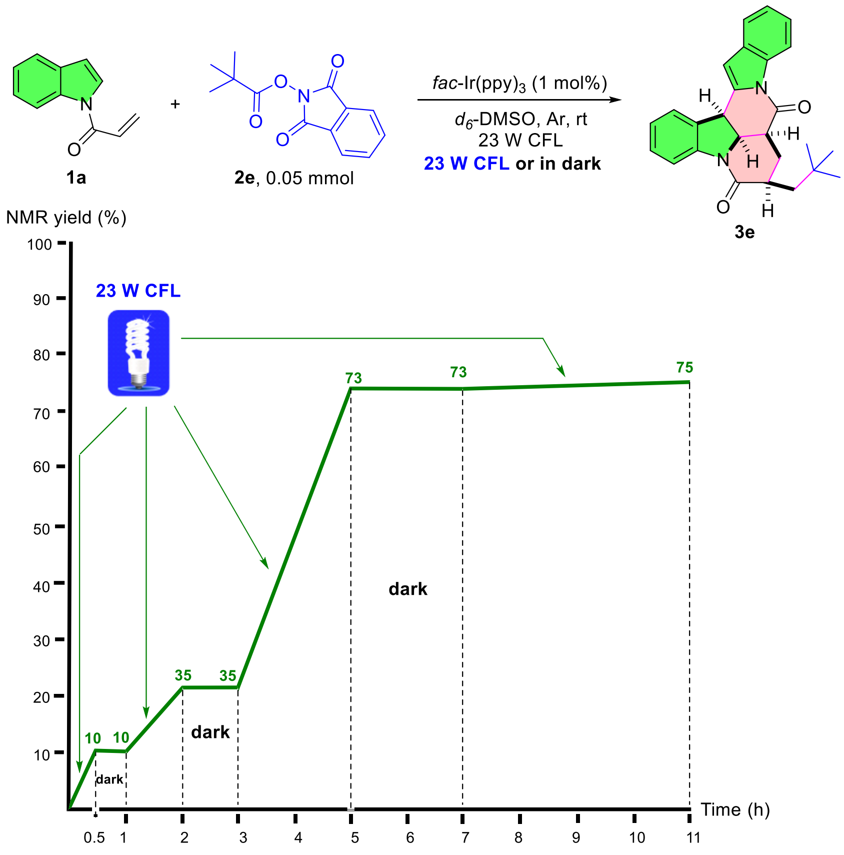

Scheme S7. Light/dark interval experiment

The requirement of constant irradiation of visible light suggests that the reaction is not likely to process though a radical chain propagation pathway.[14]

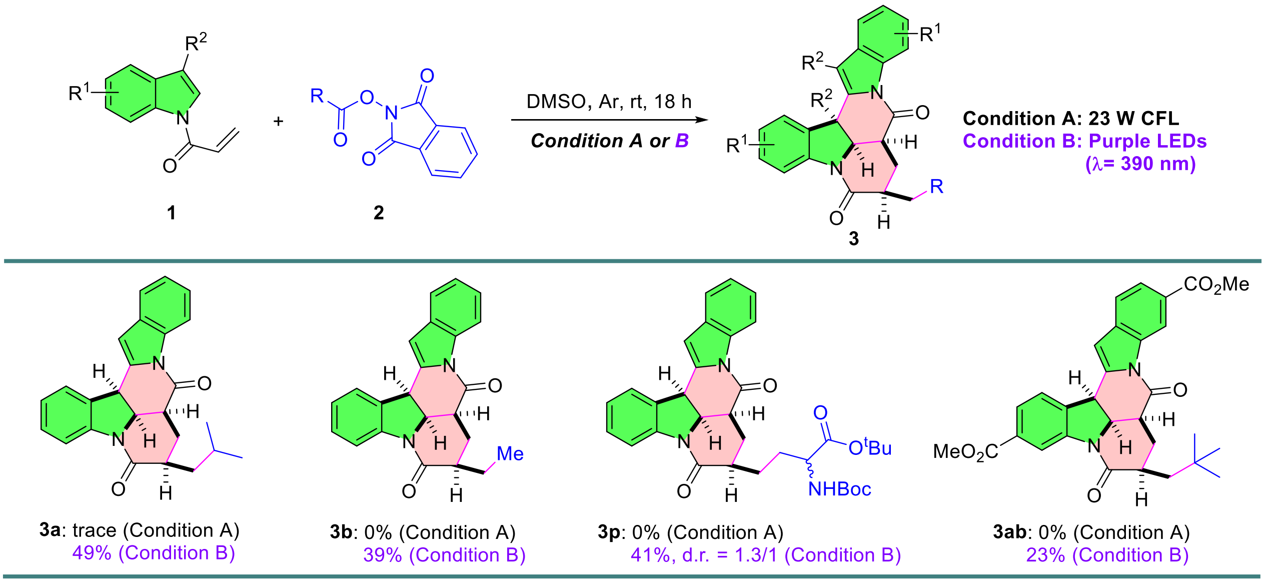

[a] The reaction was conducted with **1** (0.3 mmol) and **2** (0.1 mmol) in DMSO (1.0 mL). Isolated yield.

Table S5. Photo-mediated PC-free radical cascade [4 + 2]/[4 + 2] cycloaddition of simple N-acryloyl indoles and NHPI esters

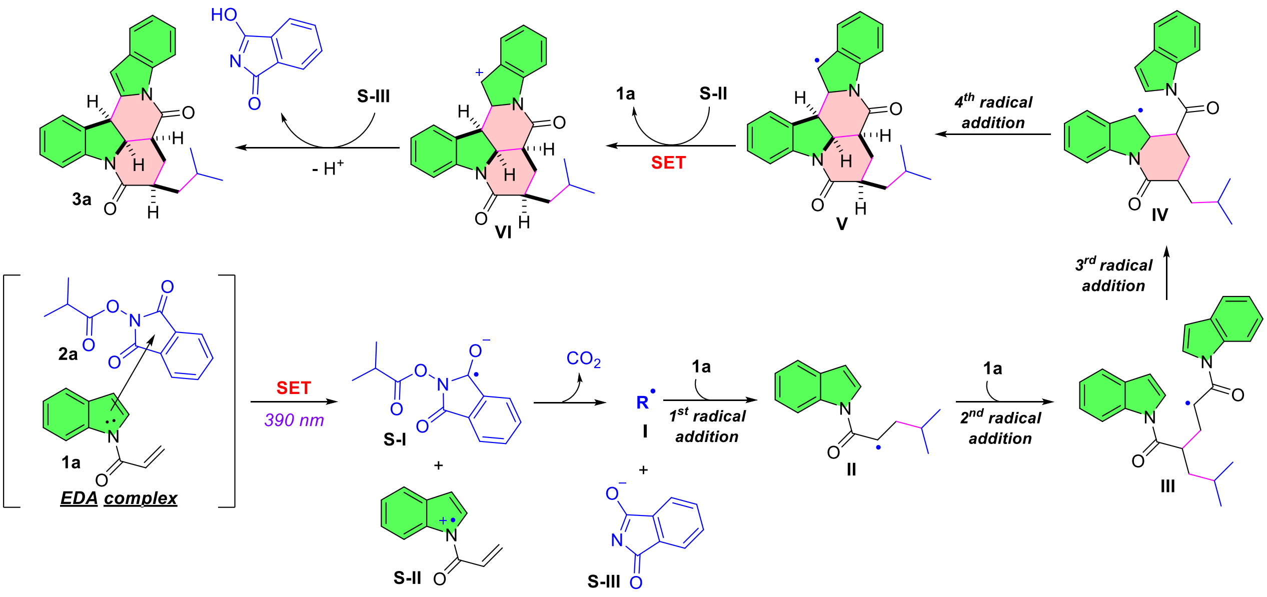

Scheme S8. Proposed visible-light-induced EDA-driven mechanism

The formation of **3a**, **3b**, **3p**, and **3ab** in the absence of PC under purple light suggests that an EDA-complex-driven pathway is also possible (Table S5). This alternative pathway could account for the increased yields observed for certain products (e.g., **3b**, **3p**, **3aa**, and **3ab**) under purple LEDs (λ_{max} = 390 nm) in Table 2. Based on previous reports,[15-19] we therefore tentatively propose a visible-light-induced EDA-driven mechanism (Scheme S8). Upon formation of a photoactive electron donor–acceptor (EDA) complex between **1a** and **2a**, a SET event occurs under purple light irradiation, generating intermediates **S-I** and **S-II**. Intermediate **S-I** subsequently decomposes to afford an alkyl radical **I** (R$^{\bullet}$) and anion **S-III**. Radical **I** then participates in a cascade of four consecutive radical additions to generate the key benzyl radical intermediate **V**. Intermediate **V** undergoes a second SET process with **S-II** to form the benzylic carbocation intermediate **VI**, which is subsequently deprotonated by **S-III** to furnish the desired product **3a**.

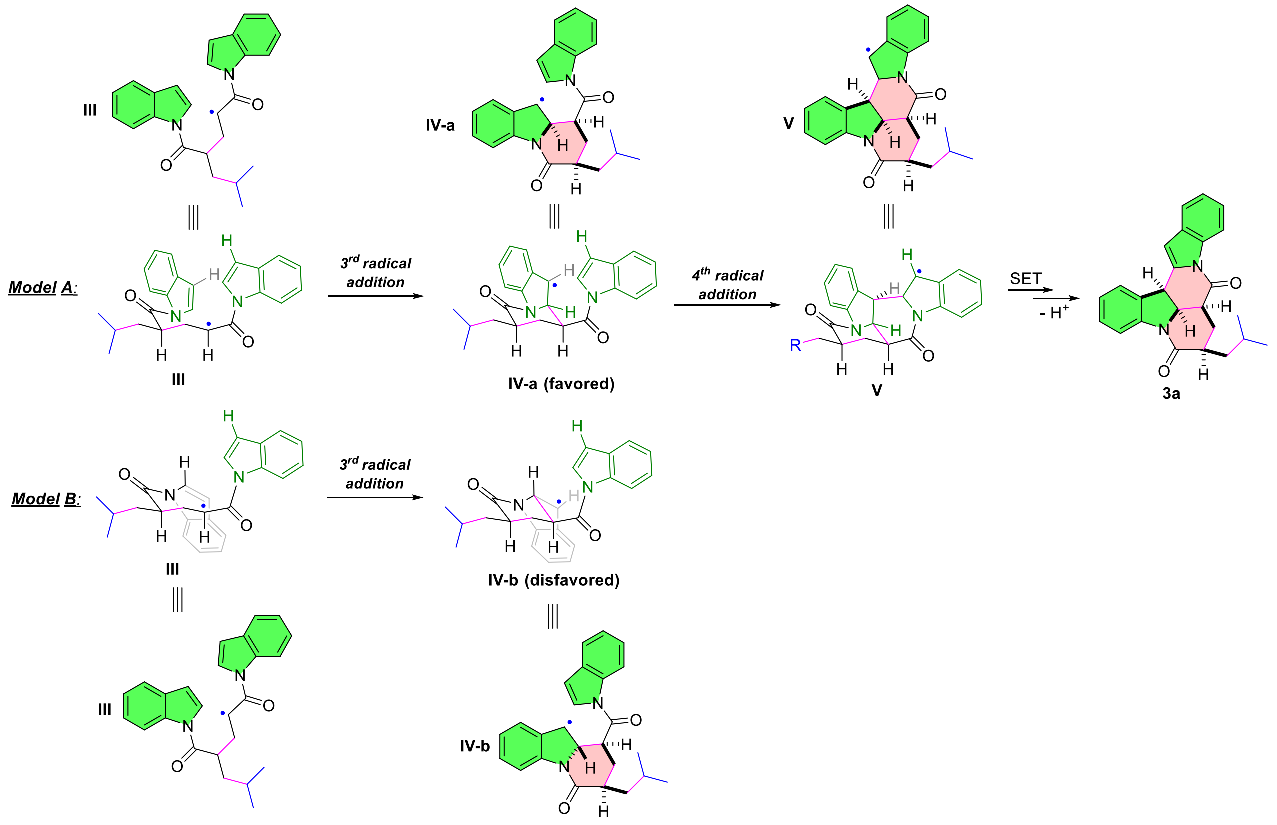

Scheme S9. Two proposed mechanistic models for the observed diastereoselectivity

To elucidate the origin of the exclusive diastereoselectivity observed in this reaction, two possible mechanistic models involving the key radical intermediate **IV** are proposed. As shown in Scheme S9, intermediate **IV-a** is more favorable than **IV-b** due to steric repulsion between the two axial hydrogens and the benzene ring (highlighted in grey) in **IV-b**. Consequently, a selective radical attack from the backside of the indole ring in **IV-a** leads to the formation of intermediate **V**, ultimately resulting in the exclusive formation of the observed diastereomer **3a**.

13. Characterization for simple N-acryloyl indoles 1 and NHPI esters 2

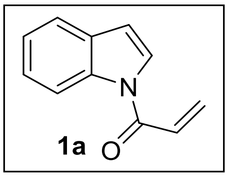

1-(1H-indol-1-yl)prop-2-en-1-one (**1a**):[1] Yield (30.0 mmol scale, 3.16 g, 62% yield after recrystallization in Et_2O/Hexanes at -20 °C). A white solid. R_f = 0.5 (Hexanes/EtOAc = 4/1). FC (Hexanes/EtOAc = 20/1). ^1H NMR (500 MHz, $CDCl_3$) δ 8.44 (dd, *J* = 8.4, 0.9 Hz, 1H), 7.51 (ddd, *J* = 7.7, 1.3, 1.1 Hz, 1H), 7.45 (d, *J* = 3.8 Hz, 1H), 7.30 (ddd, *J* = 8.4, 7.2, 1.3 Hz, 1H), 7.23 (ddd, *J* = 7.7, 7.2, 1.1 Hz, 1H), 6.90 (dd, *J* = 16.8, 10.5 Hz, 1H), 6.67 – 6.56 (m, 2H), 5.97 (dd, *J* = 10.5, 1.4 Hz, 1H). $^{13}C\{^1H\}$ NMR (126 MHz, $CDCl_3$) δ 163.9, 135.8, 132.1, 130.6, 128.0, 125.2, 124.6, 124.0, 120.9, 116.8, 109.4.

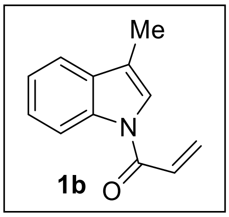

1-(3-methyl-1H-indol-1-yl)prop-2-en-1-one (**1b**): Yield (10.0 mmol scale, 328 mg, 18% yield after recrystallization in Et_2O/Hexanes at -20 °C). A white solid. M. P.: 33 – 35 °C. R_f = 0.6 (Hexanes/EtOAc = 4/1). FC (Hexanes/EtOAc = 20/1). ^1H NMR (500 MHz, $CDCl_3$) δ 8.49 (dd, *J* = 8.1, 1.1 Hz, 1H), 7.52 (ddd, *J* = 7.6, 1.4, 1.1 Hz, 1H), 7.38 (ddd, *J* = 8.4, 7.2, 1.4 Hz, 1H), 7.34 – 7.27 (m, 2H), 6.95 (dd, *J* = 16.8, 10.5 Hz, 1H), 6.65 (dd, *J* = 16.8, 1.5 Hz, 1H), 6.01 (dd, *J* = 10.5, 1.5 Hz, 1H), 2.30 (d, *J* = 1.4 Hz, 3H). $^{13}C\{^1H\}$ NMR (126 MHz, $CDCl_3$)δ 163.5, 136.1, 131.6, 131.5, 128.2, 125.2, 123.7, 121.5, 118.9, 118.7, 116.9, 9.7. HRMS (ESI) m/z: $[M + H]^+$ Calcd. for $C_{12}H_{12}NO$ 186.0913; Found 186.0915.

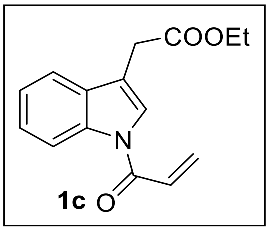

ethyl 2-(1-acryloyl-1H-indol-3-yl)acetate (**1c**): Yield (10.0 mmol scale, 1.32 g, 51% yield after recrystallization in Et_2O/Hexanes at -20 °C). A white solid. M. P.: 50 – 52 °C. R_f = 0.5 (Hexanes/EtOAc = 4/1). FC (Hexanes/EtOAc = 8/1). ^1H NMR (500 MHz, $CDCl_3$) δ 8.43 (ddd, *J* = 8.3, 1.1, 0.9 Hz, 1H), 7.53 – 7.43 (m, 2H), 7.32 (ddd, *J* = 8.4, 7.2, 1.3 Hz, 1H), 7.25 (ddd, *J* = 7.5, 7.2, 1.1 Hz, 1H), 6.90 (dd, *J* = 16.7, 10.5 Hz, 1H), 6.59 (dd, *J* = 16.7, 1.4 Hz, 1H), 5.96 (dd, *J* = 10.5, 1.5 Hz, 1H), 4.13 (q, *J* = 7.1 Hz, 2H), 3.66 (d, *J* = 1.1 Hz, 2H), 1.21 (t, *J* = 7.2 Hz, 3H). $^{13}C\{^1H\}$ NMR (126 MHz, $CDCl_3$) δ 170.8, 163.7, 136.0, 132.0, 130.3, 128.0, 125.5, 123.9, 123.2, 119.0, 117.0, 115.3, 61.1, 31.1, 14.2. HRMS (ESI) m/z: $[M + H]^+$ Calcd. for $C_{15}H_{16}NO_3$ 258.1125; Found 258.1128.

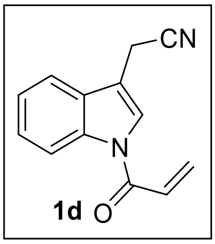

2-(1-acryloyl-1H-indol-3-yl)acetonitrile (**1d**): Yield (10.0 mmol scale, 1.05 g, 50% yield). A white solid. R_f = 0.3 (Hexanes/EtOAc = 3/1). FC (Hexanes/EtOAc = 5/1 ~ 4/1). ^{1}H NMR (500 MHz, $CDCl_3$) δ 8.43 (ddd, *J* = 8.4, 1.0, 0.9 Hz, 1H), 7.49 (d, *J* = 1.4 Hz, 1H), 7.43 (ddd, *J* = 7.7, 1.4, 1.0 Hz, 1H), 7.36 (ddd, *J* = 8.5, 7.2, 1.3 Hz, 1H), 7.28 (ddd, *J* = 7.7, 7.2, 1.0 Hz, 1H), 6.87 (dd, *J* = 16.7, 10.4 Hz, 1H), 6.61 (dd, *J* = 16.8, 1.3 Hz, 1H), 6.00 (dd, *J* = 10.5, 1.4 Hz, 1H), 3.71 (d, *J* = 1.3 Hz, 2H).^{13}C{^{1}H} NMR (126 MHz, $CDCl_3$) δ 163.6, 136.2, 132.8, 128.6, 127.5, 126.2, 124.3, 122.9, 118.2, 117.2, 116.9, 111.8, 14.5. HRMS (ESI) m/z: $[M + H]^+$ Calcd. for $C_{13}H_{11}N_2O$ 211.0866; Found 211.0868.

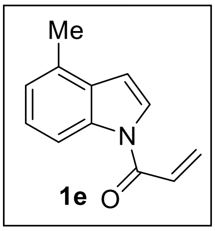

1-(4-methyl-1H-indol-1-yl)prop-2-en-1-one (**1e**): Yield (10.0 mmol scale, 574 mg, 31% yield after recrystallization in Et_2O/Hexanes at -20 ºC). A white solid. M. P.: 26 – 28 ºC. R_f = 0.6 (Hexanes/EtOAc = 4/1). FC (Hexanes/EtOAc = 25/1). ^{1}H NMR (500 MHz, $CDCl_3$) δ 8.33 (d, *J* = 8.3 Hz, 1H), 7.51 (d, *J* = 3.8 Hz, 1H), 7.27 (dd, *J* = 8.3, 7.3 Hz, 1H), 7.10 (ddd, *J* = 7.2, 0.9, 0.9 Hz, 1H), 6.97 (dd, *J* = 16.8, 10.5 Hz, 1H), 6.74 – 6.63 (m, 2H), 6.03 (dd, *J* = 10.5, 1.4 Hz, 1H), 2.53 (s, 3H).^{13}C{^{1}H} NMR (126 MHz, $CDCl_3$) δ δ 164.0, 135.6, 131.9, 130.3, 130.2, 128.1, 125.2, 124.4, 124.0, 114.3, 107.8, 18.5. HRMS (ESI) m/z: $[M + H]^+$ Calcd. for $C_{12}H_{12}NO$ 186.0913; Found 186.0914.

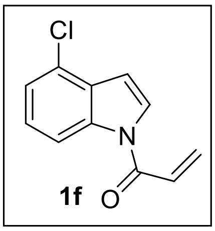

1-(4-chloro-1H-indol-1-yl)prop-2-en-1-one (**1f**): Yield (10.0 mmol scale, 1.45 g, 71% yield). A white solid. R_f = 0.5 (Hexanes/EtOAc = 4/1). FC (Hexanes/EtOAc = 10/1). ^{1}H NMR (500 MHz, $CDCl_3$) δ 8.37 – 8.28 (m, 1H), 7.45 (d, *J* = 3.9 Hz, 1H), 7.24 – 7.18 (m, 2H), 6.86 (dd, *J* = 16.7, 10.5 Hz, 1H), 6.71 (dd, *J* = 3.8, 0.8 Hz, 1H), 6.61 (dd, *J* = 16.8, 1.4 Hz, 1H), 5.99 (dd, *J* = 10.5, 1.4 Hz, 1H). ^{13}C{^{1}H} NMR (126 MHz, $CDCl_3$) δ 163.9, 136.5, 132.8, 129.4, 127.6, 126.1, 125.9, 125.1, 123.8, 115.3, 107.4. HRMS (ESI) m/z: $[M + H]^+$ Calcd. for $C_{11}H_9ClNO$ 206.0367; Found 206.0368.

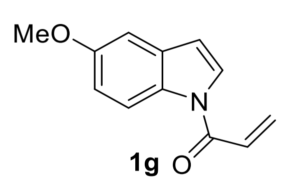

1g

1-(5-methoxy-1H-indol-1-yl)prop-2-en-1-one (**1g**): Yield (10.0 mmol scale, 932 mg, 46% yield). A white solid. R_f = 0.5 (Hexanes/EtOAc = 4/1). FC (Hexanes/EtOAc = 15/1 ~ 10/1). ^{1}H NMR (500 MHz, $CDCl_3$) δ 8.33 (d, *J* = 9.0 Hz, 1H), 7.41 (d, *J* = 3.8 Hz, 1H), 6.97 (d, *J* = 2.6 Hz, 1H), 6.93 – 6.84 (m, 2H), 6.59 (dd, *J* = 16.7, 1.5 Hz, 1H), 6.53 (dd, *J* = 3.8, 0.7 Hz, 1H), 5.95 (dd, *J* = 10.5, 1.5 Hz, 1H), 3.79 (s, 3H).^{13}C{^{1}H} NMR (126 MHz, $CDCl_3$) δ 163.5, 156.7, 131.8, 131.7, 130.5, 127.7, 125.2, 117.6, 113.5, 109.3, 103.7, 55.6. HRMS (ESI) m/z: $[M + H]^+$ Calcd. for $C_{12}H_{12}NO$ 202.0863; Found 202.0864.

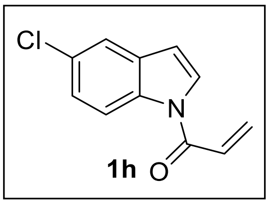

1h

1-(5-chloro-1H-indol-1-yl)prop-2-en-1-one (**1h**): Yield (10.0 mmol scale, 975 mg, 47% yield). A white solid. R_f = 0.5 (Hexanes/EtOAc = 4/1). FC (Hexanes/EtOAc = 15/1 ~ 10/1). ^{1}H NMR (500 MHz, $CDCl_3$) δ 8.36 (d, *J* = 8.9 Hz, 1H), 7.45 (dd, *J* = 7.4, 2.8 Hz, 2H), 7.24 (dd, *J* = 8.8, 2.1 Hz, 1H), 6.86 (dd, *J* = 16.7, 10.4 Hz, 1H), 6.61 (dd, *J* = 16.8, 1.4 Hz, 1H), 6.53 (dd, *J* = 3.8, 1.3 Hz, 1H), 5.98 (ddd, *J* = 10.5, 1.3, 1.1 Hz, 1H). ^{13}C{^{1}H} NMR (126 MHz, $CDCl_3$) δ 163.7, 134.2, 132.6, 131.8, 129.5, 127.5, 125.8, 125.3, 120.5, 117.8, 108.6. HRMS (ESI) m/z: $[M + H]^+$ Calcd. for $C_{11}H_9ClNO$ 206.0367; Found 206.0371.

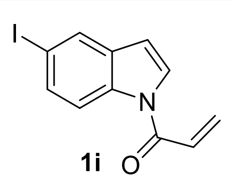

1i

1-(5-iodo-1H-indol-1-yl)prop-2-en-1-one (**1i**): Yield (10.0 mmol scale, 1.02 g, 34% yield). A white solid. R_f = 0.5 (Hexanes/EtOAc = 8/1). FC (Hexanes/EtOAc = 15/1 ~ 10/1). ^{1}H NMR (500 MHz, $CDCl_3$) δ 8.17 (dd, *J* = 8.8, 0.7 Hz, 1H), 7.80 (d, *J* = 1.7 Hz, 1H), 7.53 (dd, *J* = 8.7, 1.7 Hz, 1H), 7.35 (d, *J* = 3.8 Hz, 1H), 6.82 (dd, *J* = 16.7, 10.5 Hz, 1H), 6.58 (dd, *J* = 16.7, 1.4 Hz, 1H), 6.47 (dd, *J* = 3.8, 0.8 Hz, 1H), 5.96 (dd, *J* = 10.4, 1.4 Hz, 1H). ^{13}C{^{1}H} NMR (126 MHz, $CDCl_3$) 163.7, 135.0, 133.6, 132.8, 132.7, 129.8, 127.5, 125.3, 118.6, 108.3, 88.2. HRMS (ESI) m/z: $[M + H]^+$ Calcd. for $C_{11}H_9INO$ 297.9723; Found 297.9724.

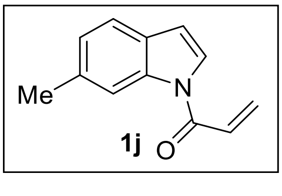

1j

1-(6-methyl-1H-indol-1-yl)prop-2-en-1-one (**1j**): Yield (10.0 mmol scale, 211 mg, 11% yield after recrystallization in Et_2O/Hexanes at -80 °C). A brown oil at room temperature. R_f = 0.6 (Hexanes/EtOAc = 4/1). FC (Hexanes/EtOAc = 25/1). ^1H NMR (500 MHz, $CDCl_3$) δ 8.35 (s, 1H), 7.51 – 7.39 (m, 2H), 7.13 (dd, J = 7.9, 1.5 Hz, 1H), 6.96 (dd, J = 16.8, 10.4 Hz, 1H), 6.71 – 6.57 (m, 2H), 6.03 (dd, J = 10.4, 1.5 Hz, 1H), 2.50 (s, 3H). $^{13}C\{^1H\}$ NMR (126 MHz, $CDCl_3$) δ 163.9, 136.2, 135.3, 131.8, 128.3, 128.0, 125.4, 124.0, 120.4, 117.1, 109.3, 21.9. HRMS (ESI) m/z: $[M + H]^+$ Calcd. for $C_{12}H_{12}NO$ 186.0913; Found 186.0915.

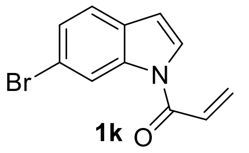

1k

1-(6-bromo-1H-indol-1-yl)prop-2-en-1-one (**1k**): Yield (10.0 mmol scale, 1.74 g, 70% yield). A white solid. R_f = 0.5 (Hexanes/EtOAc = 4/1). FC (Hexanes/EtOAc = 15/1). ^1H NMR (500 MHz, $CDCl_3$) δ 8.66 (dd, J = 1.6, 0.8 Hz, 1H), 7.42 (d, J = 3.8 Hz, 1H), 7.40 – 7.30 (m, 2H), 6.86 (dd, J = 16.7, 10.4 Hz, 1H), 6.67 – 6.54 (m, 2H), 6.00 (dd, J = 10.4, 1.4 Hz, 1H). $^{13}C\{^1H\}$ NMR (126 MHz, $CDCl_3$) δ 163.7, 136.4, 132.7, 129.4, 127.5, 127.2, 125.0, 121.9, 120.0, 118.9, 109.1. HRMS (ESI) m/z: $[M + H]^+$ Calcd. for $C_{11}H_9BrNO$ 249.9862; Found 249.9862.

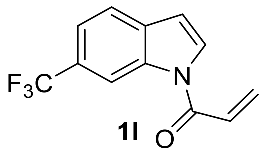

1l

1-(6-(trifluoromethyl)-1H-indol-1-yl)prop-2-en-1-one (**1l**): Yield (10.0 mmol scale, 1.02 g, 43% yield after recrystallization in Et_2O/Hexanes at -20 °C). A white solid. M. P.: 58 – 60 °C. R_f = 0.4 (Hexanes/EtOAc = 4/1). FC (Hexanes/EtOAc = 15/1 ~ 10/1). ^1H NMR (500 MHz, $CDCl_3$) δ 8.85 (s, 1H), 7.71 – 7.60 (m, 2H), 7.54 (dd, J = 8.1, 1.6 Hz, 1H), 6.96 (dd, J = 16.7, 10.4 Hz, 1H), 6.78 – 6.66 (m, 2H), 6.10 (dd, J = 10.5, 1.3 Hz, 1H). $^{13}C\{^1H\}$ NMR (126 MHz, $CDCl_3$) δ 163.8, 135.0, 133.1, 133.1, 127.3, 127.2 (q, J = 32.8 Hz), 126.9, 124.7 (q, J = 272.2 Hz), 121.2, 120.7 (q, J = 3.78 Hz), 114.4 (q, J = 3.78 Hz), 108.9. ^{19}F NMR (471 MHz, $CDCl_3$) δ -61.03. HRMS (ESI) m/z: $[M + H]^+$ Calcd. for $C_{12}H_9F_3NO$ 240.0631; Found 240.0634.

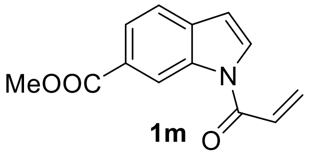

1m

methyl 1-acryloyl-1H-indole-6-carboxylate (**1m**): Yield (10.0 mmol scale, 1.53 g, 67% yield). A white solid. R_f = 0.3 (Hexanes/EtOAc = 4/1). FC (Hexanes/EtOAc = 10/1 ~ 5/1). ^1H NMR (500 MHz, $CDCl_3$) δ 9.17 (d, J = 1.4 Hz, 1H), 8.00 (dd, J = 8.2, 1.5 Hz, 1H), 7.66 (d, J = 3.8 Hz, 1H), 7.60 (d, J = 8.2 Hz, 1H), 6.97 (dd, J = 16.7, 10.4 Hz, 1H), 6.77 – 6.65 (m, 2H), 6.08 (dd, J = 10.4, 1.4 Hz, 1H), 3.95 (s, 3H). $^{13}C\{^1H\}$ NMR (126 MHz, $CDCl_3$) δ 167.51, 163.69, 135.21, 134.30,

132.85, 127.51, 127.46, 126.87, 125.20, 120.62, 118.46, 109.08, 52.13. HRMS (ESI) m/z: $[M + H]^+$ Calcd. for $C_{13}H_{12}NO_3$ 230.0812; Found 230.0794.

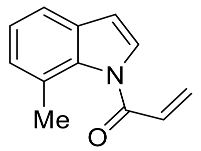

1n

1-(7-methyl-1H-indol-1-yl)prop-2-en-1-one (**1n**): Yield (10.0 mmol scale, 101 mg, 5% yield). A white solid. R_f = 0.6 (Hexanes/EtOAc = 4/1). FC (Hexanes/EtOAc = 25/1). ^{1}H NMR (500 MHz, $CDCl_3$) δ 7.42 (d, *J* = 7.7 Hz, 1H), 7.38 (d, *J* = 3.8 Hz, 1H), 7.21 (dd, *J* = 7.7, 7.3 Hz, 1H), 7.15 (d, *J* = 7.3 Hz, 1H), 6.85 (dd, *J* = 17.0, 10.4 Hz, 1H), 6.65 – 6.54 (m, 2H), 6.04 (dd, *J* = 10.4, 1.3 Hz, 1H), 2.53 (s, 3H). ^{13}C{^{1}H} NMR (126 MHz, $CDCl_3$) δ 163.7, 135.1, 132.2, 132.1, 130.0, 127.9, 126.6, 126.4, 124.2, 118.7, 108.7, 22.1. HRMS (ESI) m/z: $[M + H]^+$ Calcd. for $C_{12}H_{12}NO$ 186.0913; Found 186.0915.

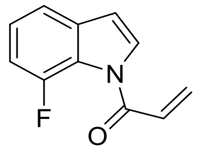

1o

1-(7-fluoro-1H-indol-1-yl)prop-2-en-1-one (**1o**): Yield (10.0 mmol scale, 693 mg, 37% yield). A white solid. R_f = 0.5 (Hexanes/EtOAc = 4/1). FC (Hexanes/EtOAc = 15/1). ^{1}H NMR (500 MHz, $CDCl_3$) δ 7.62 (d, *J* = 3.7 Hz, 1H), 7.37 (dd, *J* = 7.7, 1.0 Hz, 1H), 7.22 (ddd, *J* = 8.0, 7.7, 4.2 Hz, 1H), 7.06 (ddd, *J* = 12.2, 8.0, 1.0 Hz, 1H), 6.92 (ddd, *J* = 16.8, 10.4, 3.1 Hz, 1H), 6.73 – 6.62 (m, 2H), 6.10 – 6.01 (m, 1H). ^{13}C{^{1}H} NMR (126 MHz, $CDCl_3$) δ 163.1, 150.3 (d, *J* = 252.0 Hz), 135.0 (d, *J* = 3.8 Hz), 132.4 (d, *J* = 1.3 Hz), 129.1 (d, *J* = 7.6 Hz), 127.3, 124.5 (d, *J* = 7.6 Hz), 122.2 (d, *J* = 10.1 Hz), 117.1 (d, *J* = 3.8 Hz), 111.7 (d, *J* = 21.4 Hz), 108.7 (d, *J* = 2.5 Hz). ^{19}F NMR (471 MHz, $CDCl_3$) δ -114.72. HRMS (ESI) m/z: $[M + H]^+$ Calcd. for $C_{11}H_9FNO$ 190.0663; Found 190.0664.

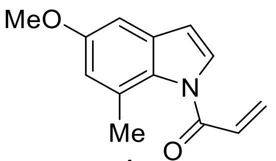

1p

1-(5-methoxy-7-methyl-1H-indol-1-yl)prop-2-en-1-one (**1p**): Yield (10.0 mmol scale, 154 mg, 7% yield). A white solid. R_f = 0.6 (Hexanes/EtOAc = 4/1). FC (Hexanes/EtOAc = 25/1). ^{1}H NMR (500 MHz, $CDCl_3$) δ 7.37 (d, *J* = 3.7 Hz, 1H), 6.92 – 6.81 (m, 2H), 6.78 (d, *J* = 2.5 Hz, 1H), 6.64 – 6.51 (m, 2H), 6.03 (dd, *J* = 10.3, 1.3 Hz, 1H), 3.84 (s, 3H), 2.51 (s, 3H). ^{13}C{^{1}H} NMR (126 MHz, $CDCl_3$) δ 163.4, 156.9, 133.2, 131.8, 129.9, 129.8, 127.7, 127.3, 116.4, 108.8, 101.0, 55.6, 22.2. HRMS (ESI) m/z: $[M + H]^+$ Calcd. for $C_{13}H_{14}NO_2$ 216.1019; Found 216.1018.

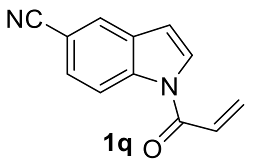

1q

1-acryloyl-1H-indole-5-carbonitrile (**1q**): Yield (10.0 mmol scale, 412 mg, 21% yield). A white solid. R_f = 0.5 (Hexanes/EtOAc = 2/1). FC (Hexanes/EtOAc/DCM = 10/2/1). ^{1}H NMR (500 MHz, $CDCl_3$) δ 8.60 (d, *J* = 8.6 Hz, 1H), 7.91 (d, *J* = 1.7 Hz, 1H), 7.65 (d, *J* = 3.9 Hz, 1H), 7.61 (dd, *J* = 8.7, 1.7 Hz, 1H), 6.96 (dd, *J* = 16.7, 10.4 Hz, 1H), 6.81 – 6.68 (m, 2H), 6.14 (dd, *J* = 10.4, 1.3 Hz, 1H). $^{13}C\{^1H\}$ NMR (126 MHz, $CDCl_3$) δ 163.9, 137.6, 133.6, 130.6, 128.2, 127.2, 126.7, 125.7, 119.5, 117.6, 108.8, 107.3. HRMS (ESI) m/z: $[M + H]^+$ Calcd. for $C_{12}H_9N_2O$ 197.0709; Found 197.0712.

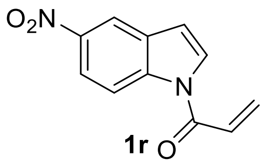

1r

1-(5-nitro-1H-indol-1-yl)prop-2-en-1-one (**1r**): Yield (10.0 mmol scale, 182 mg, 8% yield after recrystallization in Et_2O/Hexanes at -20 ºC). A light yellow solid. M. P.: 144 – 146 ºC. R_f = 0.5 (Hexanes/EtOAc = 2/1). FC (Hexanes/EtOAc = 5/1 ~ 4/1). ^{1}H NMR (500 MHz, *d*$_6$-DMSO) δ 8.63 – 8.50 (m, 2H), 8.32 (d, *J* = 3.9 Hz, 1H), 8.22 (dd, *J* = 9.1, 2.4 Hz, 1H), 7.39 (dd, *J* = 16.6, 10.4 Hz, 1H), 7.02 (dd, *J* = 3.8, 0.7 Hz, 1H), 6.64 (dd, *J* = 16.6, 1.5 Hz, 1H), 6.21 (dd, *J* = 10.4, 1.5 Hz, 1H). $^{13}C\{^1H\}$ NMR (126 MHz, *d*$_6$-DMSO) δ 164.6, 144.2, 138.7, 134.3, 131.1, 130.1, 128.3, 120.2, 117.6, 117.0, 109.8. HRMS (ESI) m/z: $[M + H]^+$ Calcd. for $C_{11}H_9N_2O_3$ 217.0608; Found 217.0609.

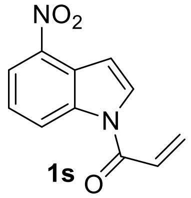

1s

1-(4-nitro-1H-indol-1-yl)prop-2-en-1-one (**1s**): Yield (10.0 mmol scale, 852 mg, 39% yield). A light yellow solid. R_f = 0.5 (Hexanes/EtOAc = 2/1). FC (Hexanes/EtOAc = 5/1 ~ 4/1). ^{1}H NMR (500 MHz, $CDCl_3$) δ 8.80 (ddd, *J* = 8.3, 0.9, 0.9 Hz, 1H), 8.16 (dd, *J* = 8.0, 0.9 Hz, 1H), 7.68 (d, *J* = 3.8 Hz, 1H), 7.45 – 7.31 (m, 2H), 6.91 (dd, *J* = 16.7, 10.4 Hz, 1H), 6.67 (dd, *J* = 16.8, 1.2 Hz, 1H), 6.08 (dd, *J* = 10.4, 1.3 Hz, 1H). $^{13}C\{^1H\}$ NMR (126 MHz, $CDCl_3$) δ 163.9, 140.4, 137.4, 133.8, 128.4, 127.1, 125.1, 124.6, 123.1, 120.6, 108.4. HRMS (ESI) m/z: $[M + H]^+$ Calcd. for $C_{11}H_9N_2O_3$ 217.0608; Found 217.0610.

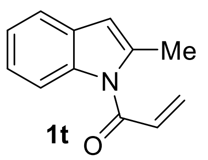

1t

1-(2-methyl-1H-indol-1-yl)prop-2-en-1-one (**1t**):[2] Yield (20.0 mmol scale, 282 mg, 8% yield; The low yield is likely due to product loss during rotary evaporation). A light yellow oil. R_f = 0.5 (Hexanes/EtOAc = 10/1). FC (Hexanes/EtOAc = 30/1). ^{1}H NMR (500 MHz, $CDCl_3$) δ 7.85 – 7.77 (m, 1H), 7.54 – 7.45 (m, 1H), 7.31 – 7.19 (m, 2H), 7.00 (dd, *J* = 17.0, 10.4 Hz, 1H), 6.64 (dd, *J* = 17.0, 1.4 Hz, 1H), 6.41 (s, 1H), 6.05 (dd, *J* = 10.4, 1.4 Hz, 1H), 2.64 (s, 3H). $^{13}C\{^1H\}$ NMR (126 MHz, $CDCl_3$) δ 166.0, 137.4, 136.1, 131.8, 131.2, 129.9, 123.2, 123.1, 120.1, 114.6, 109.0, 16.5.

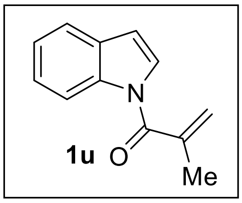

1u

1-(1H-indol-1-yl)-2-methylprop-2-en-1-one (**1u**):[3] Yield (10.0 mmol scale, 962 mg, 52% yield in 2 steps). A brown oil. R_f = 0.5 (Hexanes/EtOAc = 10/1). FC (Hexanes/EtOAc = 30/1). ^{1}H NMR (500 MHz, $CDCl_3$) δ 8.35 (ddd, *J* = 8.3, 1.3, 0.9 Hz, 1H), 7.51 (ddd, *J* = 7.7, 1.1, 0.9 Hz, 1H), 7.41 (d, *J* = 3.8 Hz, 1H), 7.29 (ddd, *J* = 8.4, 7.2, 1.3 Hz, 1H), 7.22 (ddd, *J* = 7.5, 7.2, 1.1 Hz, 1H), 6.54 (dd, *J* = 3.8, 0.8 Hz, 1H), 5.61 (qd, *J* = 1.6, 0.6 Hz, 1H), 5.40 – 4.39 (m, 1H), 2.10 (t, *J* = 1.4 Hz, 3H). $^{13}C\{^1H\}$ NMR (126 MHz, $CDCl_3$) δ 169.7, 139.9, 135.6, 131.0, 127.0, 124.9, 123.9, 121.9, 120.8, 116.5, 108.5, 20.0.

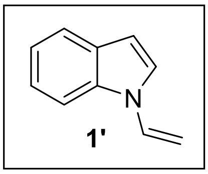

1'

N-vinylindole (**1'**):[12] Yield (10.0 mmol scale, 458 mg, 32% yield for 2 steps). A colorless solid. R_f = 0.5 (Hexanes/EtOAc = 10/1). FC (Hexanes/EtOAc = 30/1). ^{1}H NMR (500 MHz, $CDCl_3$) δ 7.65 (ddd, *J* = 7.8, 1.1 1.1 Hz, 1H), 7.55 – 7.40 (m, 2H), 7.36 – 7.23 (m, 2H), 7.23 – 7.15 (m, 1H), 6.66 (dd, *J* = 3.0, 3.0 Hz, 1H), 5.24 – 5.20 (m, 1H), 4.80 (ddd, *J* = 8.9, 1.4, 1.4 Hz, 1H). $^{13}C\{^1H\}$ NMR 135.48, 129.66, 129.13, 123.36, 122.69, 121.16, 120.77, 109.49, 104.92, 96.43.

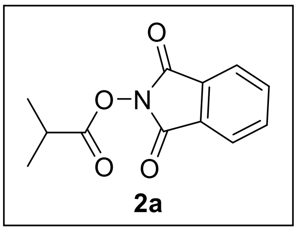

2a

1,3-dioxoisoindolin-2-yl isobutyrate (**2a**):[5] Yield (10.0 mmol scale, 1.72 g, 74% yield). A white solid. R_f = 0.6 (Hexanes/EtOAc = 2/1). FC (Hexanes/EtOAc = 6/1). ^{1}H NMR (500 MHz, $CDCl_3$) δ 7.80 (dd, *J* = 5.5, 3.1 Hz, 2H), 7.71 (dd, *J* = 5.5, 3.1 Hz, 2H), 2.88 (hept, *J* = 7.0 Hz, 1H), 1.30 (d, *J* = 7.0 Hz, 6H). $^{13}C\{^1H\}$ NMR (126 MHz, $CDCl_3$) δ 173.1, 162.0, 134.7, 129.0, 123.9, 31.8, 18.8.

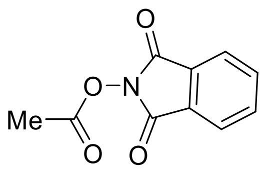

2b

1,3-dioxoisoindolin-2-yl acetate (**2b**):[5] Yield (10.0 mmol scale, 476 mg, 23% yield). A white solid. R_f = 0.6 (Hexanes/EtOAc = 2/1). FC (Hexanes/EtOAc = 6/1). ^{1}H NMR (500 MHz, $CDCl_3$) δ 7.89 (dd, *J* = 5.5, 3.1 Hz, 2H), 7.79 (dd, *J* = 5.5, 3.1 Hz, 2H), 2.40 (s, 3H). ^{13}C{^{1}H} NMR (126 MHz, $CDCl_3$) δ 166.6, 161.9, 134.8, 128.9, 124.0, 17.6.

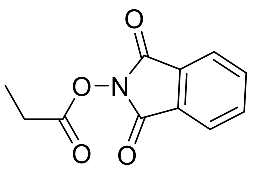

2c

1,3-dioxoisoindolin-2-yl propionate (**2c**):[6] Yield (10.0 mmol scale,1.75 g, 80% yield). A white solid. R_f = 0.6 (Hexanes/EtOAc = 2/1). FC (Hexanes/EtOAc = 6/1). ^{1}H NMR (500 MHz, $CDCl_3$) δ 7.80 (dd, *J* = 5.5, 3.1 Hz, 2H), 7.71 (dd, *J* = 5.5, 3.1 Hz, 2H), 2.63 (q, *J* = 7.5 Hz, 2H), 1.24 (t, *J* = 7.5 Hz, 3H). ^{13}C{^{1}H} NMR (126 MHz, $CDCl_3$) δ 170.4, 162.0, 134.8, 128.9, 123.9, 24.5, 8.7.

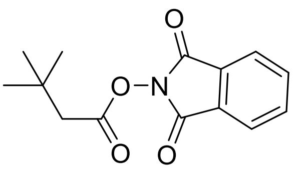

2d

1,3-dioxoisoindolin-2-yl 3,3-dimethylbutanoate (**2d**):[7] Yield (10.0 mmol scale, 2.00 g, 77% yield). A white solid. R_f = 0.6 (Hexanes/EtOAc = 2/1). FC (Hexanes/EtOAc = 6/1). ^{1}H NMR (500 MHz, $CDCl_3$) δ 7.88 (dd, *J* = 5.5, 3.1 Hz, 2H), 7.79 (dd, *J* = 5.5, 3.1 Hz, 2H), 2.53 (s, 2H), 1.17 (s, 9H). ^{13}C{^{1}H} NMR (126 MHz, $CDCl_3$) δ 167.9, 162.1, 134.7, 129.0, 123.9, 44.6, 31.3, 29.5.

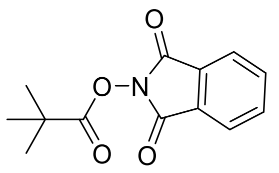

2e

1,3-dioxoisoindolin-2-yl pivalate (**2e**):[5] Yield (10.0 mmol scale, 2.08 g, 84% yield). A white solid. R_f = 0.6 (Hexanes/EtOAc = 2/1). FC (Hexanes/EtOAc = 6/1). ^{1}H NMR (500 MHz, $CDCl_3$) δ 7.80 (dd, *J* = 5.4, 3.2 Hz, 2H), 7.71 (dd, *J* = 5.6, 3.2 Hz, 2H), 1.36 (s, 9H). ^{13}C{^{1}H} NMR (126 MHz, $CDCl_3$) δ 174.4, 162.1, 134.7, 129.1, 123.9, 38.4, 27.0.

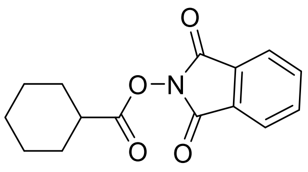

2f

1,3-dioxoisoindolin-2-yl cyclohexanecarboxylate (**2f**):[5] Yield (10.0 mmol scale, 1.95 g, 71% yield). A white solid. R_f = 0.6 (Hexanes/EtOAc = 2/1). FC (Hexanes/EtOAc = 6/1). ^{1}H NMR (500 MHz, $CDCl_3$) δ 7.81 (dd, *J* = 5.4, 3.1 Hz, 2H), 7.71 (dd, *J* = 5.5, 3.1 Hz, 2H), 2.67 (tt, *J* = 10.9, 3.7 Hz, 1H), 2.10 – 1.99 (m, 2H), 1.82 – 1.70 (m, 2H), 1.66 – 1.54 (m, 3H), 1.37 – 1.21 (m, 3H). ^{13}C{^{1}H} NMR (126 MHz, $CDCl_3$) δ 171.8, 162.1, 134.7, 129.0, 123.9, 40.5, 28.8, 25.5, 25.0.

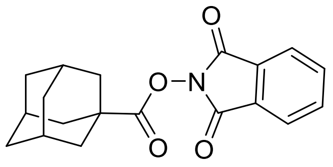

2g

1,3-dioxoisoindolin-2-yl (3r,5r,7r)-adamantane-1-carboxylate (**2g**):[5] Yield (10.0 mmol scale, 3.03 g, 93% yield). A white solid. R_f = 0.6 (Hexanes/EtOAc = 2/1). FC (Hexanes/EtOAc = 6/1). ^{1}H NMR (500 MHz, $CDCl_3$) δ 7.87 (dd, *J* = 5.4, 3.1 Hz, 2H), 7.78 (dd, *J* = 5.5, 3.1 Hz, 2H), 2.14 (d, *J* = 3.0 Hz, 6H), 2.13 – 2.06 (m, 3H), 1.84 – 1.73 (m, 6H). ^{13}C{^{1}H} NMR (126 MHz, $CDCl_3$) δ 173.3, 162.2, 134.6, 129.1, 123.8, 40.5, 38.5, 36.2, 27.7.

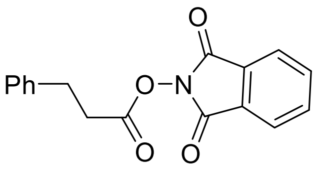

2h

1,3-dioxoisoindolin-2-yl 3-phenylpropanoate (**2h**):[8] Yield (10.0 mmol scale, 2.50 g, 85% yield). A white solid. R_f = 0.6 (Hexanes/EtOAc = 2/1). FC (Hexanes/EtOAc = 6/1). ^{1}H NMR (500 MHz, $CDCl_3$) δ 7.79 (dd, *J* = 5.5, 3.1 Hz, 2H), 7.69 (dd, *J* = 5.5, 3.1 Hz, 2H), 7.28 – 7.22 (m, 2H), 7.16 (ddd, *J* = 10.8, 6.5, 1.5 Hz, 3H), 3.01 (dd, *J* = 8.9, 7.1 Hz, 2H), 2.90 (ddd, *J* = 8.1, 7.2, 1.0 Hz, 2H). ^{13}C{^{1}H} NMR (126 MHz, $CDCl_3$) δ 168.9, 161.9, 139.2, 134.8, 128.9, 128.7, 128.3, 126.7, 124.0, 32.7, 30.6.

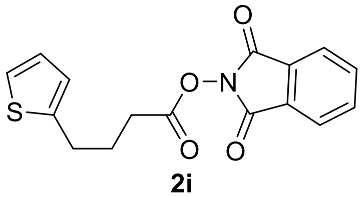

2i

1,3-dioxoisoindolin-2-yl 4-(thiophen-2-yl)butanoate (**2i**):[5] Yield (10.0 mmol scale, 2.65 g, 84% yield). A white solid. R_f = 0.5 (Hexanes/EtOAc = 3/1). FC (Hexanes/EtOAc = 4/1). ^{1}H NMR (500 MHz, $CDCl_3$) δ 7.89 (dd, *J* = 5.5, 3.1 Hz, 2H), 7.79 (dd, *J* = 5.5, 3.1 Hz, 2H), 7.15 (dd, *J* = 5.2, 1.2

Hz, 1H), 6.94 (dd, J = 5.1, 3.4 Hz, 1H), 6.87 (ddt, J = 3.4, 1.2, 1.0 Hz, 1H), 3.01 (td, J = 7.4, 1.0 Hz, 2H), 2.72 (t, J = 7.3 Hz, 2H), 2.16 (p, J = 7.4 Hz, 2H). $^{13}C\{^1H\}$ NMR (126 MHz, $CDCl_3$)δ 169.3, 162.0, 143.2, 134.8, 128.9, 127.0, 125.1, 124.0, 123.6, 30.0, 28.6, 26.6.

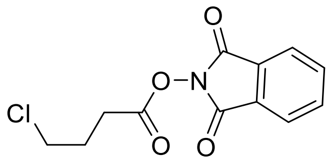

2j

1,3-dioxoisoindolin-2-yl 4-chlorobutanoate (**2j**):[5] Yield (10.0 mmol scale, 2.12 g, 79% yield). A white solid. R_f = 0.6 (Hexanes/EtOAc = 2/1). FC (Hexanes/EtOAc = 5/1). ^1H NMR (500 MHz, $CDCl_3$) δ 7.91 (dd, J = 5.5, 3.1 Hz, 2H), 7.82 (dd, J = 5.5, 3.1 Hz, 2H), 3.71 (t, J = 6.2 Hz, 2H), 2.91 (t, J = 7.3 Hz, 2H), 2.27 (tt, J = 7.2, 6.1 Hz, 2H). $^{13}C\{^1H\}$ NMR (126 MHz, $CDCl_3$) δ 168.9, 161.9, 134.9, 128.8, 124.0, 43.3, 28.2, 27.4.

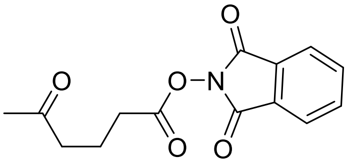

2k

1,3-dioxoisoindolin-2-yl 5-oxohexanoate (**2k**):[7] Yield (10.0 mmol scale, 1.98 g, 76% yield). A white solid. R_f = 0.3 (Hexanes/EtOAc = 2/1). FC (Hexanes/EtOAc = 4/1 ~ 2/1). ^1H NMR (500 MHz, $CDCl_3$) δ 7.89 (dd, J = 5.5, 3.1 Hz, 2H), 7.80 (dd, J = 5.5, 3.1 Hz, 2H), 2.73 (t, J = 7.1 Hz, 2H), 2.66 (t, J = 7.1 Hz, 2H), 2.19 (s, 3H), 2.05 (p, J = 7.0 Hz, 2H). $^{13}C\{^1H\}$ NMR (126 MHz, $CDCl_3$) δ 207.4, 169.3, 161.9, 134.8, 128.9, 124.0, 41.5, 30.1, 30.0, 18.6.

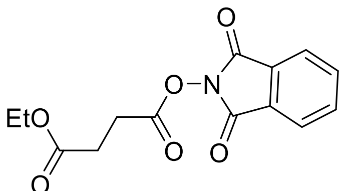

2l

1,3-dioxoisoindolin-2-yl ethyl succinate (**2l**):[9] Yield (10.0 mmol scale, 2.36 g, 81% yield). A white solid. R_f = 0.4 (Hexanes/EtOAc = 2/1). FC (Hexanes/EtOAc = 5/1 ~ 3/1). ^1H NMR (500 MHz, $CDCl_3$) δ 7.89 (dd, J = 5.5, 3.1 Hz, 2H), 7.79 (dd, J = 5.5, 3.1 Hz, 2H), 4.20 (q, J = 7.1 Hz, 2H), 3.02 (t, J = 7.1 Hz, 2H), 2.78 (t, J = 7.1 Hz, 2H), 1.28 (t, J = 7.1 Hz, 3H). $^{13}C\{^1H\}$ NMR (126 MHz, $CDCl_3$) δ 171.0, 168.7, 161.7, 134.8, 128.9, 124.0, 61.2, 28.9, 26.4, 14.1.

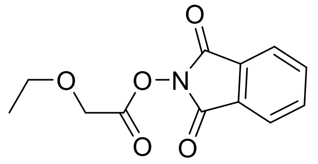

2m

1,3-dioxoisoindolin-2-yl 2-ethoxyacetate (**2m**):[10] Yield (10.0 mmol scale, 1.59 g, 64% yield). A white solid. R_f = 0.4 (Hexanes/EtOAc = 2/1). FC (Hexanes/EtOAc = 5/1 ~ 4/1). ^{1}H NMR (500 MHz, $CDCl_3$) δ 7.90 (dd, *J* = 5.5, 3.1 Hz, 2H), 7.81 (dd, *J* = 5.5, 3.1 Hz, 2H), 4.49 (s, 2H), 3.71 (q, *J* = 7.0 Hz, 2H), 1.29 (t, *J* = 7.0 Hz, 3H). ^{13}C{^{1}H} NMR (126 MHz, $CDCl_3$) δ 167.0, 161.6, 134.9, 128.8, 124.1, 67.9, 65.9, 14.9.

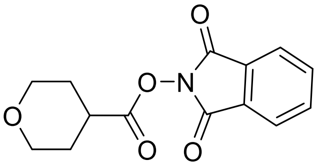

2n

1,3-dioxoisoindolin-2-yl tetrahydro-2H-pyran-4-carboxylate (**2n**):[5] Yield (10.0 mmol scale, 1.36 g, 49% yield). R_f = 0.4 (Hexanes/EtOAc = 2/1). FC (Hexanes/EtOAc = 5/1 ~ 4/1). ^{1}H NMR (500 MHz, $CDCl_3$) δ 7.89 (dd, *J* = 5.5, 3.1 Hz, 2H), 7.80 (dd, *J* = 5.5, 3.1 Hz, 2H), 4.02 (dt, *J* = 11.8, 3.8 Hz, 2H), 3.54 (ddd, *J* = 11.9, 10.2, 3.0 Hz, 2H), 3.01 (tt, *J* = 10.2, 4.5 Hz, 1H), 2.11 – 1.92 (m, 4H). ^{13}C{^{1}H} NMR (126 MHz, $CDCl_3$) δ 170.6, 161.9, 134.8, 128.9, 124.0, 66.6, 37.6, 28.3.

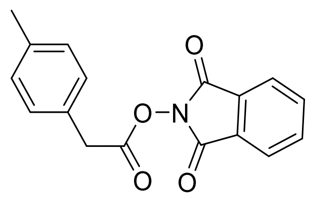

2o

1,3-dioxoisoindolin-2-yl 2-(p-tolyl)acetate (**2o**):[5] Yield (10.0 mmol scale, 1.91 g, 65% yield). A white solid. R_f = 0.4 (Hexanes/EtOAc = 2/1). FC (Hexanes/EtOAc = 5/1 ~ 4/1). ^{1}H NMR (500 MHz, $CDCl_3$) δ 7.85 (dd, *J* = 5.5, 3.1 Hz, 2H), 7.75 (dd, *J* = 5.5, 3.1 Hz, 2H), 7.26 (d, *J* = 7.9 Hz, 2H), 7.17 (d, *J* = 7.9 Hz, 2H), 3.95 (s, 2H), 2.34 (s, 3H). ^{13}C{^{1}H} NMR (126 MHz, $CDCl_3$) δ 167.9, 161.9, 137.5, 134.8, 129.6, 129.2, 128.9, 128.5, 124.0, 37.3, 21.2.

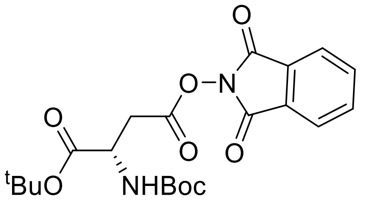

2p

1-(tert-butyl) 4-(1,3-dioxoisoindolin-2-yl) (tert-butoxycarbonyl)-L-aspartate (**2p**):[5] Yield (10.0

mmol scale, 3.01 g, 69% yield). A white solid. R_f = 0.4 (Hexanes/EtOAc = 2/1). FC (Hexanes/EtOAc = 5/1 ~ 4/1). ^{1}H NMR (500 MHz, $CDCl_3$) δ 7.89 (dd, *J* = 5.5, 3.1 Hz, 2H), 7.80 (dd, *J* = 5.5, 3.1 Hz, 2H), 5.52 (d, *J* = 7.9 Hz, 1H), 4.59 (dt, *J* = 8.5, 4.5 Hz, 1H), 3.43 – 3.12 (m, 2H), 1.49 (s, 9H), 1.47 (s, 9H). ^{13}C{^{1}H} NMR (126 MHz, $CDCl_3$) δ 168.7, 167.5, 161.6, 155.3, 134.8, 128.8, 124.0, 83.3, 80.2, 50.2, 34.2, 28.3, 27.7.

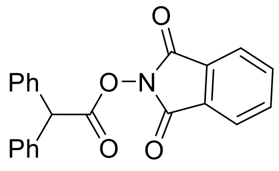

2q

1,3-dioxoisoindolin-2-yl 2,2-diphenylacetate (**2q**):[11] Yield (10.0 mmol scale, 428 mg, 12% yield). A white solid. R_f = 0.4 (Hexanes/EtOAc = 2/1). FC (Hexanes/EtOAc = 4/1). ^{1}H NMR (500 MHz, $CDCl_3$) δ 7.86 (dd, *J* = 5.5, 3.1 Hz, 2H), 7.76 (dd, *J* = 5.5, 3.1 Hz, 2H), 7.45 – 7.35 (m, 8H), 7.32 (ddd, *J* = 8.4, 5.9, 1.8 Hz, 2H), 5.42 (s, 1H). ^{13}C{^{1}H} NMR (126 MHz, $CDCl_3$) δ 169.1, 161.9, 136.8, 134.8, 128.9, 128.7, 127.9, 124.0, 54.0.

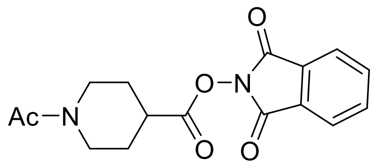

2r

1,3-dioxoisoindolin-2-yl 1-acetylpiperidine-4-carboxylate (**2r**):[5] Yield (10.0 mmol scale, 1.92 g, 61 % yield). A white solid. R_f = 0.3 (EtOAc). FC (Hexanes/EtOAc = 1/2 ~ 0/1). ^{1}H NMR (500 MHz, $CDCl_3$) δ 7.89 (dd, *J* = 5.5, 3.1 Hz, 2H), 7.81 (dd, *J* = 5.5, 3.1 Hz, 2H), 4.41 – 4.31 (m, 1H), 3.84 (dt, *J* = 13.6, 3.8 Hz, 1H), 3.29 (ddd, *J* = 13.7, 10.4, 3.1 Hz, 1H), 3.11 – 2.96 (m, 2H), 2.16 – 2.07 (m, 5H), 1.97 – 1.85 (m, 2H). ^{13}C{^{1}H} NMR (126 MHz, $CDCl_3$) δ 170.4, 168.9, 161.9, 134.9, 128.9, 124.0, 45.1, 40.2, 38.3, 28.3, 27.4, 21.4.

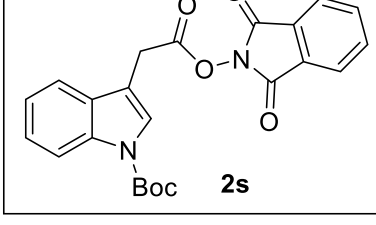

2s

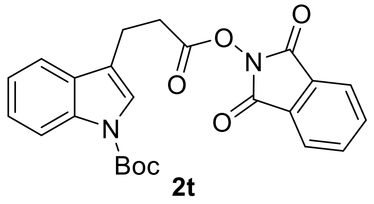

2t

2s and **2t** have been reported and characterized in our previous work.[4]

14. Characterization for DHPI products 3, 5 and compounds 6-7

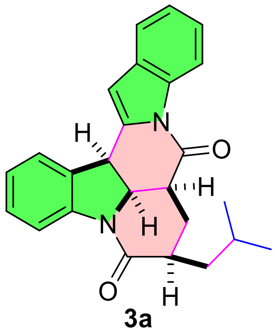

3a

7-isobutyl-51,8,8a,15b-tetrahydro-6H-diindolo[1,2-g:3',2',1'-ij][1,6]naphthyridine-6,9(7H)-dione (**3a**): Yield after the additional washing step (0.1 mmol scale; **23 W CFL**: 24 mg, 62% yield; **Purple**: 23 mg, 60% yield). A white solid. M. P.: 162 – 164 °C. R_f = 0.4 (Hexanes/EtOAc = 3/1). FC (Hexanes/EtOAc = 6/1 ~ 5/1). ^{1}H NMR (500 MHz, $CDCl_3$) δ 8.37 (d, *J* = 9.3 Hz, 1H), 8.11 (d, *J* = 8.1 Hz, 1H), 7.51 – 7.42 (m, 2H), 7.33 – 7.19 (m, 3H), 7.10 (ddd, *J* = 7.5, 1.1, 1.1 Hz, 1H), 6.50 (dd, J = 1.5, 0.8 Hz, 1H), 4.86 (dd, *J* = 8.6, 6.2 Hz, 1H), 4.78 (d, *J* = 8.7 Hz, 1H), 3.45 – 3.37 (m, 1H), 2.64 – 2.52 (m, 2H), 2.47 – 2.35 (m, 1H), 1.99 – 1.88 (m, 1H), 1.88 – 1.79 (m, 1H), 1.36 (ddd, *J* = 13.9, 8.8, 5.3 Hz, 1H), 0.97 (d, *J* = 6.5 Hz, 3H), 0.95 (d, *J* = 6.5 Hz, 3H). $^{13}C\{^1H\}$ NMR (126 MHz, $CDCl_3$) δ 172.2, 168.2, 141.3, 136.7, 135.0, 131.8, 129.9, 129.1, 124.8, 124.6, 124.5, 124.3, 120.3, 116.7, 116.2, 106.8, 58.9, 39.8, 38.5, 38.3, 25.9, 25.3, 23.4, 21.6. HRMS (ESI) m/z: $[M + H]^+$ Calcd. for $C_{25}H_{25}N_2O_2$ 385.1911; Found 385.1909.

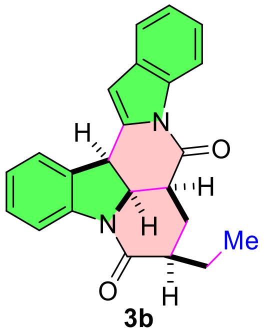

3b

7-ethyl-51,8,8a,15b-tetrahydro-6H-diindolo[1,2-g:3',2',1'-ij][1,6]naphthyridine-6,9(7H)-dione (**3b**): Yield after the additional washing step (0.1 mmol scale; **23 W CFL**: 5 mg, 14% yield; **Purple**: 17 mg, 48% yield). A white solid. R_f = 0.3 (Hexanes/EtOAc = 3/1). FC (Hexanes/EtOAc = 5/1 ~ 4/1). ^{1}H NMR (500 MHz, $CDCl_3$) δ 8.38 (dd, *J* = 8.1, 1.1 Hz, 1H), 8.12 (dd, *J* = 8.0, 1.4 Hz, 1H), 7.51 – 7.43 (m, 2H), 7.31 – 7.23 (m, 3H), 7.10 (ddd, *J* = 8.0, 7.6, 1.1 Hz, 1H), 6.51 (dd, *J* = 1.5, 0.8 Hz, 1H), 4.87 (dd, *J* = 8.7, 6.2 Hz, 1H), 4.81 (d, *J* = 8.5 Hz, 1H), 3.48 – 3.38 (m, 1H), 2.68 – 2.57 (m, 1H), 2.49 – 2.38 (m, 2H), 2.09 – 1.98 (m, 1H), 1.59 – 1.49 (m, 1H), 1.11 (t, *J* = 7.4 Hz, 3H). $^{13}C\{^1H\}$ NMR (126 MHz, $CDCl_3$) δ 171.8, 168.2, 141.3, 136.7, 135.1, 131.7, 129.9, 129.1, 124.8, 124.6, 124.5, 124.3, 120.3, 116.7, 116.2, 106.8, 58.9, 42.3, 38.4, 38.3, 25.5, 23.9, 12.1. HRMS (ESI) m/z: $[M + H]^+$ Calcd. for $C_{23}H_{21}N_2O_2$ 357.1598; Found 357.1601.

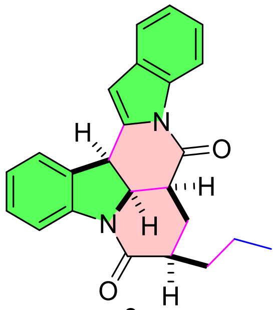

3c

7-propyl-51,8,8a,15b-tetrahydro-6H-diindolo[1,2-g:3',2',1'-ij][1,6]naphthyridine-6,9(7H)-dione (**3c**): Yield after the additional washing step (0.1 mmol scale; **23 W CFL**: 21 mg, 57% yield). A white solid. R_f = 0.4 (Hexanes/EtOAc = 3/1). FC (Hexanes/EtOAc = 6/1 ~ 5/1). ^{1}H NMR (500 MHz, $CDCl_3$) δ 8.37 (d, J = 8.1 Hz, 1H), 8.11 (d, J = 8.2 Hz, 1H), 7.48 – 7.44 (m, 2H), 7.32 – 7.19 (m, 3H), 7.09 (ddd, J = 7.5, 1.1, 1.0 Hz, 1H), 6.50 (dd, J = 1.0, 1.0 Hz, 1H), 4.85 (dd, J = 8.7, 6.2 Hz, 1H), 4.78 (d, J = 8.7 Hz, 1H), 3.45 – 3.36 (m, 1H), 2.61 (ddd, J = 13.9, 8.5, 5.1 Hz, 1H), 2.55 – 2.37 (m, 2H), 2.02 – 1.91 (m, 1H), 1.62 – 1.55 (m, 1H), 1.55 – 1.42 (m, 2H), 0.98 (t, J = 7.0 Hz, 3H). ^{13}C{^{1}H} NMR (126 MHz, $CDCl_3$) δ 172.0, 168.2, 141.3, 136.7, 135.0, 131.7, 129.9, 129.1, 124.8, 124.5, 124.4, 124.3, 120.3, 116.7, 116.2, 106.8, 58.9, 40.5, 38.4, 38.3, 32.9, 25.9, 20.6, 14.1. HRMS (ESI) m/z: $[M + H]^+$ Calcd. for $C_{24}H_{23}N_2O_2$ 371.1754; Found 371.1760.

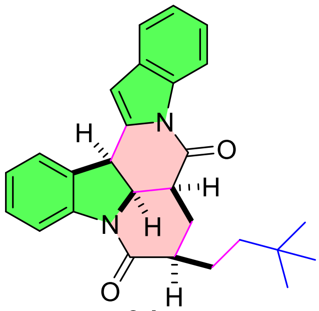

3d

7-(3,3-dimethylbutyl)-51,8,8a,15b-tetrahydro-6H-diindolo[1,2-g:3',2',1'-ij][1,6]naphthyridine-6,9(7H)-dione (**3d**): Yield (0.1 mmol scale; **23 W CFL**: 23 mg, 56% yield). A white solid. R_f = 0.5 (Hexanes/EtOAc = 3/1). FC (Hexanes/EtOAc = 6/1). ^{1}H NMR (500 MHz, $CDCl_3$) δ 8.38 (d, J = 8.1 Hz, 1H), 8.13 (d, J = 8.0 Hz, 1H), 7.48 – 7.44 (m, 2H), 7.30 – 7.21 (m, 2H), 7.10 (ddd, J = 8.0, 7.5, 1.1 Hz, 1H), 6.50 (d, J = 1.4 Hz, 1H), 4.84 (dd, J = 8.7, 6.2 Hz, 1H), 4.78 (d, J = 8.6 Hz, 1H), 3.46 – 3.36 (m, 1H), 2.69 – 2.56 (m, 1H), 2.50 – 2.35 (m, 2H), 2.06 – 1.94 (m, 1H), 1.55 – 1.38 (m, 2H), 1.35 – 1.27 (m, 1H), 0.93 (s, 9H). ^{13}C{^{1}H} NMR (126 MHz, $CDCl_3$) δ 172.0, 168.2, 141.3, 136.6, 135.1, 131.8, 129.9, 129.1, 124.8, 124.6, 124.5, 124.3, 120.3, 116.7, 116.2, 106.8, 58.9, 41.8, 41.6, 38.3, 38.3, 30.5, 29.4, 25.9, 25.8. HRMS (ESI) m/z: $[M + H]^+$ Calcd. for $C_{27}H_{29}N_2O_2$ 413.2224; Found 413.2220.

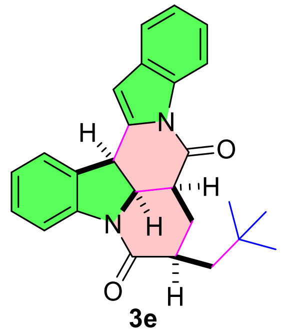

3e

7-neopentyl-51,8,8a,15b-tetrahydro-6H-diindolo[1,2-g:3',2',1'-ij][1,6]naphthyridine-6,9(7H)-dione (**3e**): Yield (0.1 mmol scale; **23 W CFL**: 27 mg, 68% yield; **Purple**: 25 mg, 63% yield). A white solid. R_f = 0.5 (Hexanes/EtOAc = 3/1). FC (Hexanes/EtOAc = 6/1). ^{1}H NMR (500 MHz, $CDCl_3$) δ 8.27 (dd, *J* = 8.1, 1.1 Hz, 1H), 8.02 (dd, *J* = 8.1, 1.0 Hz, 1H), 7.39 (dd, *J* = 6.9, 1.3 Hz, 1H), 7.34 (d, *J* = 7.5 Hz, 1H), 7.23 – 7.10 (m, 3H), 6.99 (ddd, *J* = 7.5, 6.8, 1.1 Hz, 1H), 6.45 (dd, *J* = 1.0, 1.0 Hz, 1H), 4.85 (dd, *J* = 9.2, 6.4 Hz, 1H), 4.76 (d, *J* = 9.1 Hz, 1H), 3.33 (ddd, *J* = 8.9,6.4, 6.4 Hz, 1H), 2.50 – 2.40 (m, 2H), 2.40 – 2.33 (m, 1H), 2.29 (dd, *J* = 14.1, 3.5 Hz, 1H), 1.20 (dd, *J* = 14.2, 5.3 Hz, 1H), 0.92 (s, 9H). ^{13}C{^{1}H} NMR (126 MHz, $CDCl_3$) δ 172.3, 168.3, 141.4, 136.8, 135.1, 131.6, 129.9, 129.1, 124.8, 124.6, 124.3, 124.3, 120.3, 116.4, 116.1, 106.4, 58.3, 42.6, 39.1, 38.2, 37.4, 30.8, 29.6, 29.5. HRMS (ESI) m/z: $[M + H]^+$ Calcd. for $C_{26}H_{27}N_2O_2$ 399.2067; Found 399.2070.

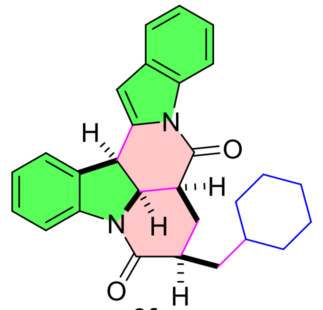

3f

7-(cyclohexylmethyl)-51,8,8a,15b-tetrahydro-6H-diindolo[1,2-g:3',2',1'-ij][1,6]naphthyridine-6,9(7H)-dione (**3f**): Yield after the additional washing step (0.1 mmol scale; **23 W CFL**: 30 mg, 71% yield; **Purple**: 27 mg, 64% yield). A white solid. R_f = 0.4 (Hexanes/EtOAc = 3/1). FC (Hexanes/EtOAc = 6/1 ~ 5/1). ^{1}H NMR (500 MHz, $CDCl_3$) δ 8.41 – 8.33 (m, 1H), 8.11 (dd, *J* = 8.2, 1.1 Hz, 1H), 7.47 – 7.44 (m, 2H), 7.32 – 7.20 (m, 3H), 7.09 (ddd, *J* = 8.1, 7.5, 1.1 Hz, 1H), 6.49 (dd, *J* = 1.5, 0.7 Hz, 1H), 4.84 (dd, *J* = 8.7, 6.2 Hz, 1H), 4.81 – 4.73 (m, 1H), 3.39 (ddd, *J* = 8.5, 6.2, 4.9 Hz, 1H), 2.66 – 2.51 (m, 2H), 2.46 – 2.36 (m, 1H), 1.87 (ddd, *J* = 13.8, 8.8, 4.9 Hz, 1H), 1.81 – 1.67 (m, 5H), 1.64 – 1.53 (m, 1H), 1.38 – 1.24 (m, 3H), 1.24 – 1.13 (m, 1H), 1.01 – 0.85 (m, 2H). ^{13}C{^{1}H} NMR (126 MHz, $CDCl_3$) δ 172.3, 168.2, 141.3, 136.7, 135.1, 131.7, 129.9, 129.1, 124.8, 124.6, 124.4, 124.3, 120.3, 116.7, 116.2, 106.8, 58.8, 38.4, 38.3, 38.3, 37.7, 34.8, 34.0, 32.4, 26.7, 26.4, 26.2, 26.2. HRMS (ESI) m/z: $[M + H]^+$ Calcd. for $C_{28}H_{29}N_2O_2$ 425.2224; Found 425.2225.

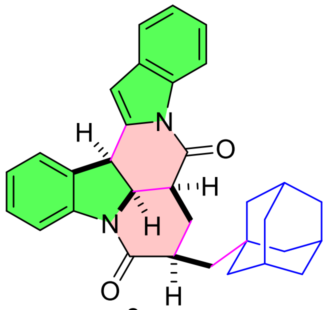

3g

7-(((3S,5S,7S)-adamantan-1-yl)methyl)-51,8,8a,15b-tetrahydro-6H-diindolo[1,2-g:3',2',1'-ij][1,6]naphthyridine-6,9(7H)-dione (**3g**): Yield (0.1 mmol scale; **23 W CFL**: 29 mg, 61% yield). A white solid. R_f = 0.5 (Hexanes/EtOAc = 3/1). FC (Hexanes/EtOAc = 6/1). ^{1}H NMR (500 MHz, $CDCl_3$) δ 8.36 (d, *J* = 8.1 Hz, 1H), 8.11 (d, *J* = 8.0 Hz, 1H), 7.47 (d, *J* = 7.5 Hz, 1H), 7.44 (d, *J* = 7.5 Hz, 1H), 7.33 – 7.17 (m, 3H), 7.07 (dd, *J* = 7.5, 7.5 Hz, 1H), 6.54 (s, 1H), 4.97 (dd, *J* = 9.1, 6.5 Hz, 1H), 4.87 (d, *J* = 9.1 Hz, 1H), 3.49 – 3.37 (m, 1H), 2.56 – 2.47 (m, 1H), 2.22 (dd, *J* = 14.3, 3.0 Hz, 1H), 2.05 – 1.92 (m, 3H), 1.75 – 1.57 (m, 12H), 1.15 (dd, *J* = 14.3, 4.2 Hz, 1H). $^{13}C\{^1H\}$ NMR (126 MHz, $CDCl_3$) δ 172.5, 168.3, 141.4, 136.8, 135.1, 131.5, 129.9, 129.1, 124.8, 124.6, 124.3, 124.3, 120.3, 116.4, 116.1, 106.4, 58.3, 43.5, 42.6, 39.2, 38.2, 37.1, 35.4, 32.6, 29.8, 28.7. HRMS (ESI) m/z: $[M + H]^+$ Calcd. for $C_{32}H_{33}N_2O_2$ 477.2537; Found 477.2532.

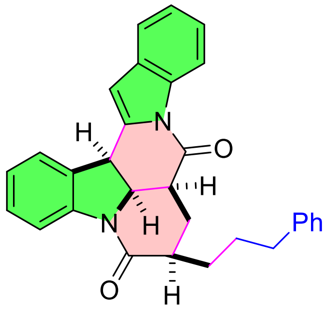

3h

7-(3-phenylpropyl)-51,8,8a,15b-tetrahydro-6H-diindolo[1,2-g:3',2',1'-ij][1,6]naphthyridine-6,9(7H)-dione (**3h**): Yield after the additional washing step (0.1 mmol scale; **23 W CFL**: 25 mg, 56% yield; **Purple**: 23 mg, 52% yield). A white solid. R_f = 0.3 (Hexanes/EtOAc = 3/1). FC (Hexanes/EtOAc = 5/1 ~ 4/1). ^{1}H NMR (500 MHz, $CDCl_3$) δ 8.35 (dd, *J* = 8.1, 1.0 Hz, 1H), 8.11 (dd, *J* = 8.1, 1.1 Hz, 1H), 7.50 – 7.43 (m, 2H), 7.31 – 7.21 (m, 7H), 7.21 – 7.15 (m, 1H), 7.09 (ddd, *J* = 7.5, 7.5, 1.1 Hz, 1H), 6.50 (dd, *J* = 1.0, 1.0 Hz, 1H), 4.82 (dd, *J* = 8.7, 6.0 Hz, 1H), 4.78 (d, *J* = 8.7 Hz, 1H), 3.44 – 3.33 (m, 1H), 2.69 – 2.63 (m, 2H), 2.61 (ddd, *J* = 14.0, 8.8, 5.2 Hz, 1H), 2.52 – 2.35 (m, 2H), 2.10 – 1.99 (m, 1H), 1.96 – 1.86 (m, 1H), 1.86 – 1.75 (m, 1H), 1.60 – 1.49 (m, 1H). $^{13}C\{^1H\}$ NMR (126 MHz, $CDCl_3$) δ 171.7, 168.2, 142.3, 141.2, 136.6, 135.0, 131.8, 129.9, 129.1, 128.5, 128.4, 125.8, 124.9, 124.6, 124.5, 124.3, 120.3, 116.7, 116.2, 106.8, 58.9, 40.8, 38.4, 38.3, 36.1, 30.6, 29.4, 25.9. HRMS (ESI) m/z: $[M + H]^+$ Calcd. for $C_{30}H_{27}N_2O_2$ 447.2067; Found 447.2073.

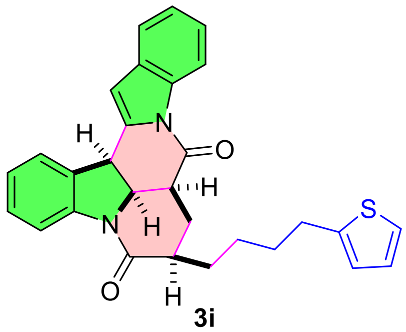

7-(4-(thiophen-2-yl)butyl)-51,8,8a,15b-tetrahydro-6H-diindolo[1,2-g:3',2',1'-ij][1,6]naphthyridine-6,9(7H)-dione (**3i**): Yield after the additional washing step (0.1 mmol scale; **23 W CFL**: 24 mg, 51% yield). A white solid. R_f = 0.4 (Hexanes/EtOAc = 3/1). FC (Hexanes/EtOAc = 5/1 ~ 4/1). ^{1}H NMR (500 MHz, $CDCl_3$) δ 8.29 (d, *J* = 8.2 Hz, 1H), 8.04 (d, *J* = 8.0 Hz, 1H), 7.40 – 6.37 (m, 2H), 7.24 – 7.12 (m, 3H), 7.06 – 6.98 (m, 2H), 6.84 (dd, *J* = 5.1, 3.4 Hz, 1H), 6.72 (dd, *J* = 3.3, 1.2 Hz, 1H), 6.42 (dd, *J* = 1.0, 0.5 Hz, 1H), 4.76 (dd, *J* = 8.7, 6.1 Hz, 1H), 4.71 (d, *J* = 8.7 Hz, 1H), 3.32 (ddd, *J* = 8.3, 5.5, 5.5 Hz, 1H), 2.80 (t, *J* = 7.6 Hz, 2H), 2.53 (ddd, *J* = 13.8, 8.5, 5.1 Hz, 1H), 2.44 – 2.27 (m, 2H), 2.01 – 1.89 (m, 1H), 1.71 – 1.64 (m, 2H), 1.62 – 1.53 (m, 1H), 1.53 – 1.39 (m, 2H). ^{13}C{^{1}H} NMR (126 MHz, $CDCl_3$) δ δ 171.8, 168.2, 145.5, 141.2, 136.6, 135.0, 131.7, 129.9, 129.1, 126.7, 124.9, 124.6, 124.5, 124.3, 124.1, 122.8, 120.3, 116.7, 116.2, 106.8, 58.9, 40.7, 38.4, 38.3, 31.8, 30.5, 29.8, 27.0, 25.9. HRMS (ESI) m/z: $[M + H]^+$ Calcd. for $C_{29}H_{27}N_2O_2S$ 467.1788; Found 467.1789.

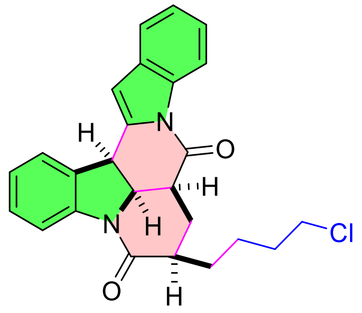

7-(4-chlorobutyl)-51,8,8a,15b-tetrahydro-6H-diindolo[1,2-g:3',2',1'-ij][1,6]naphthyridine-6,9(7H)-dione (**3j**): Yield (0.1 mmol scale; **23 W CFL**: 24 mg, 57% yield). A white solid. M. P.: 145 – 147 °C. R_f = 0.3 (Hexanes/EtOAc = 3/1). FC (Hexanes/EtOAc = 5/1 ~ 4/1). ^{1}H NMR (500 MHz, $CDCl_3$) δ 8.37 (dd, *J* = 8.1, 1.1 Hz, 1H), 8.11 (dd, *J* = 8.1, 1.0 Hz, 1H), 7.49 – 7.45 (m, 2H), 7.33 – 7.21 (m, 3H), 7.11 (ddd, *J* = 7.5, 7.5, 1.1 Hz, 1H), 6.51 (dd, *J* = 1.0, 1.0 Hz, 1H), 4.86 (dd, *J* = 8.7, 6.1 Hz, 1H), 4.81 (d, *J* = 8.6 Hz, 1H), 3.58 (t, *J* = 6.7 Hz, 2H), 3.43 (ddd, *J* = 8.5, 6.1, 5.0 Hz, 1H), 2.64 (ddd, *J* = 13.8, 8.4, 5.0 Hz, 1H), 2.55 – 2.38 (m, 2H), 2.07 – 1.95 (m, 1H), 1.90 – 1.80 (m, 2H), 1.77 – 1.69 (m, 1H), 1.66 – 1.60 (m, 1H), 1.56 – 1.46 (m, 1H). ^{13}C{^{1}H} NMR (126 MHz, $CDCl_3$) δ 171.5, 168.1, 141.2, 136.6, 135.0, 131.8, 129.9, 129.1, 124.9, 124.6, 124.4, 120.3, 116.7, 116.2, 106.9, 58.9, 44.9, 40.7, 38.3, 38.3, 32.7, 30.2, 25.8, 24.9. HRMS (ESI) m/z: $[M + H]^+$ Calcd. for $C_{25}H_{24}ClN_2O_2$ 419.1521; Found 419.1522.

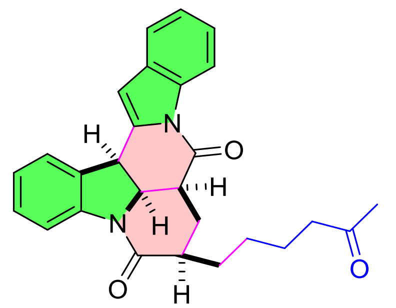

3k

7-(5-oxohexyl)-51,8,8a,15b-tetrahydro-6H-diindolo[1,2-g:3',2',1'-ij][1,6]naphthyridine-6,9(7H)-dione (**3k**): Yield (0.1 mmol scale; **23 W CFL**: 29 mg, 68% yield). A light yellow solid. R_f = 0.3 (Hexanes/EtOAc = 1/1). FC (Hexanes/EtOAc = 2/1 ~ 1/2). ^{1}H NMR (500 MHz, $CDCl_3$) δ 8.37 (d, J = 8.6 Hz, 1H), 8.10 (d, J = 8.4 Hz, 1H), 7.48 – 7.45 (m, 2H), 7.34 – 7.19 (m, 3H), 7.10 (ddd, J = 7.6, 7.6, 1.1 Hz, 1H), 6.50 (dd, J = 1.0, 1.0 Hz, 1H), 4.84 (dd, J = 8.7, 6.0 Hz, 1H), 4.78 (d, J = 8.6 Hz, 1H), 3.40 (ddd, J = 8.5, 5.4, 5.0 Hz, 1H), 2.61 (ddd, J = 13.9, 8.7, 5.0 Hz, 1H), 2.47 (t, J = 7.3 Hz, 3H), 2.44 – 2.35 (m, 1H), 2.14 (s, 3H), 2.03 – 1.91 (m, 1H), 1.68 – 1.59 (m, 2H), 1.58 – 1.53 (m, 1H), 1.52 – 1.47 (m, 2H). $^{13}C\{^1H\}$ NMR (126 MHz, $CDCl_3$) δ 209.1, 171.7, 168.2, 141.2, 136.7, 135.0, 131.8, 129.9, 129.1, 124.8, 124.6, 124.5, 124.3, 120.3, 116.7, 116.2, 106.8, 58.9, 43.4, 40.5, 38.3, 38.3, 30.6, 30.0, 26.9, 25.8, 23.6. HRMS (ESI) m/z: $[M + H]^+$ Calcd. for $C_{27}H_{27}N_2O_3$ 427.2016; Found 427.2019.

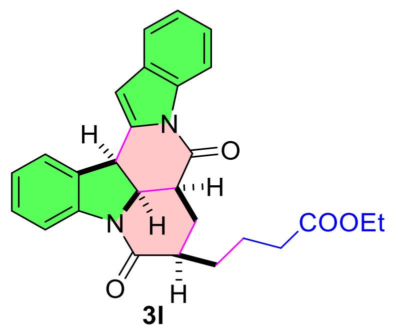

3l

ethyl-6,9-dioxo-51,7,8,8a,9,15b-hexahydro-6H-diindolo[1,2-g:3',2',1'-ij][1,6]naphthyridin-7-yl)butanoate (**3l**): Yield (0.1 mmol scale; **23 W CFL**: 19 mg, 43% yield; **Purple**: 24 mg, 54% yield). A light yellow solid. R_f = 0.5 (Hexanes/EtOAc = 1/1). FC (Hexanes/EtOAc = 3/1 ~ 2/1). ^{1}H NMR (500 MHz, $CDCl_3$) δ 8.36 (dd, J = 8.0, 1.1 Hz, 1H), 8.10 (d, J = 8.4 Hz, 1H), 7.48 – 7.45 (m, 2H), 7.31 – 7.22 (m, 3H), 7.10 (ddd, J =7.5, 7.5, 1.1 Hz, 1H), 6.52 (dd, J = 1.0, 1.0 Hz, 1H), 4.87 (dd, J = 8.8, 6.0 Hz, 1H), 4.82 (d, J = 8.8 Hz, 1H), 4.14 (q, J = 7.1 Hz, 2H), 3.48 – 3.36 (m, 1H), 2.62 (ddd, J = 13.7, 8.4, 5.1 Hz, 1H), 2.55 – 2.42 (m, 2H), 2.38 (t, J = 7.5 Hz, 2H), 2.08 – 1.97 (m, 1H), 1.97 – 1.76 (m, 2H), 1.59 – 1.47 (m, 1H), 1.27 (t, J = 7.2 Hz, 3H). $^{13}C\{^1H\}$ NMR (126 MHz, $CDCl_3$) δ 173.5, 171.4, 168.1, 141.2, 136.6, 135.0, 131.7, 129.9, 129.1, 124.8, 124.6, 124.5, 124.3, 120.3, 116.6, 116.2, 106.7, 60.3, 58.8, 40.6, 38.5, 38.3, 34.3, 30.2, 25.9, 23.0, 14.3. HRMS (ESI) m/z: $[M + H]^+$ Calcd. for $C_{27}H_{27}N_2O_4$ 443.1965; Found 443.1965.

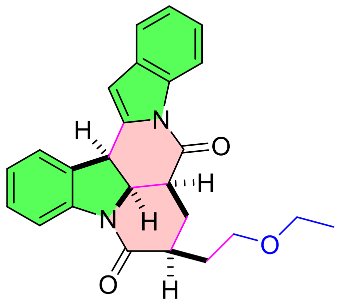

3m

7-(2-ethoxyethyl)-51,8,8a,15b-tetrahydro-6H-diindolo[1,2-g:3',2',1'-ij][1,6]naphthyridine-6,9(7H)-dione (**3m**): Yield (0.1 mmol scale; **23 W CFL**: 26 mg, 65% yield). A white solid. R_f = 0.5 (Hexanes/EtOAc = 2/1). FC (Hexanes/EtOAc = 4/1 ~ 2/1). ^{1}H NMR (500 MHz, $CDCl_3$) δ 8.35 (d, *J* = 8.1 Hz, 1H), 8.09 (d, *J* = 8.0 Hz, 1H), 7.47 (dd, *J* = 7.4, 1.5 Hz, 1H), 7.44 (d, *J* = 7.5 Hz, 1H), 7.33 – 7.19 (m, 3H), 7.08 (ddd, *J* = 7.5, 7.4, 1.1 Hz, 1H), 6.54 (s, 1H), 4.91 (dd, *J* = 9.1, 6.2 Hz, 1H), 4.85 (d, *J* = 9.1 Hz, 1H), 3.73 – 3.62 (m, 2H), 3.59 – 3.47 (m, 2H), 3.44 (ddd, *J* = 9.1, 6.0, 6.0 Hz, 1H), 2.80 – 2.73 (m, 1H), 2.60 – 2.46 (m, 2H), 2.39 – 2.29 (m, 1H), 1.78 – 1.65 (m, 1H), 1.22 (t, *J* = 7.0 Hz, 3H). ^{13}C{^{1}H} NMR (126 MHz, $CDCl_3$) 171.9, 168.3, 141.1, 136.7, 135.1, 131.7, 129.9, 129.1, 124.8, 124.7, 124.4, 124.3, 120.3, 116.4, 116.1, 106.5, 68.0, 66.1, 58.4, 38.8, 38.2, 37.3, 30.6, 26.4, 15.3. HRMS (ESI) m/z: $[M + H]^+$ Calcd. for $C_{25}H_{25}N_2O_3$ 401.1860; Found 401.1864.

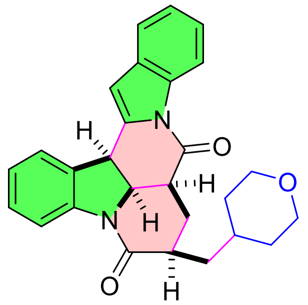

3n

7-((tetrahydro-2H-pyran-4-yl)methyl)-51,8,8a,15b-tetrahydro-6H-diindolo[1,2-g:3',2',1'-ij][1,6]naphthyridine-6,9(7H)-dione (**3n**): Yield (0.1 mmol scale; **23 W CFL**: 27 mg, 63% yield). A light yellow solid. R_f = 0.5 (Hexanes/EtOAc = 1/1). FC (Hexanes/EtOAc = 2/1 ~ 1/2). ^{1}H NMR (500 MHz, $CDCl_3$) δ 8.37 (d, *J* = 8.0 Hz, 1H), 8.11 (d, *J* = 8.0 Hz, 1H), 7.54 – 7.42 (m, 2H), 7.34 – 7.20 (m, 3H), 7.11 (dd, *J* = 7.4, 7.4 Hz, 1H), 6.50 (s, 1H), 4.86 (dd, *J* = 7.5, 6.5 Hz, 1H), 4.78 (d, *J* = 7.9 Hz, 1H), 4.00 – 3.96 (m, 2H), 3.48 – 3.40 (m, 3H), 2.68 – 2.60 (m, 2H), 2.44 – 2.36 (m, 1H), 1.96 – 1.88 (m, 2H), 1.70 – 1.63 (m, 2H), 1.48 – 1.27 (m, 3H). ^{13}C{^{1}H} NMR (126 MHz, $CDCl_3$) δ 171.8, 168.1, 141.2, 136.6, 135.0, 131.8, 129.9, 129.1, 124.9, 124.6, 124.6, 124.4, 120.3, 116.8, 116.1, 107.0, 68.1, 67.9, 59.1, 38.3, 38.2, 37.2, 33.5, 32.5, 32.3, 26.2. HRMS (ESI) m/z: $[M + H]^+$ Calcd. for $C_{27}H_{27}N_2O_3$ 427.2016; Found 427.2019.

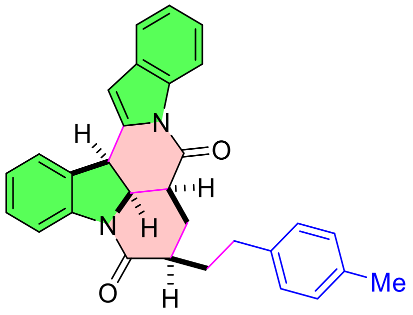

3o

7-(4-methylphenethyl)-51,8,8a,15b-tetrahydro-6H-diindolo[1,2-g:3',2',1'-ij][1,6]naphthyridine-6,9(7H)-dione (**3o**): Yield after the additional washing step (0.1 mmol scale; **23 W CFL**: 22 mg, 49% yield; **Purple**: 6 mg, 13% yield). A white solid. R_f = 0.3 (Hexanes/EtOAc = 3/1). FC (Hexanes/EtOAc = 4/1 ~ 3/1). ^{1}H NMR (500 MHz, $CDCl_3$) δ 8.31 (d, J = 8.1 Hz, 1H), 8.04 (d, J = 8.0 Hz, 1H), 7.39 – 7.37 (m, 2H), 7.24 – 7.15 (m, 3H), 7.09 – 7.07 (m, 2H), 7.03 – 7.00 (m, 3H), 6.43 (s, 1H), 4.78 – 4.65 (m, 2H), 3.33 (ddd, J = 8.3, 5.3, 5.3 Hz, 1H), 2.88 – 2.78 (m, 1H), 2.75 – 2.66 (m, 1H), 2.64 – 2.53 (m, 1H), 2.44 – 2.32 (m, 2H), 2.29 – 2.22 (m, 4H), 1.75 – 1.67 (m, 1H). $^{13}C\{^1H\}$ NMR (126 MHz, $CDCl_3$) δ 171.7, 168.2, 141.2, 138.4, 136.7, 135.4, 135.0, 131.7, 129.9, 129.1, 129.1, 128.5, 124.8, 124.6, 124.5, 124.4, 120.3, 116.7, 116.2, 106.8, 58.8, 39.6, 38.4, 38.3, 32.9, 32.5, 25.8, 21.1. HRMS (ESI) m/z: $[M + H]^+$ Calcd. for $C_{30}H_{27}N_2O_2$ 447.2067; Found 447.2070.

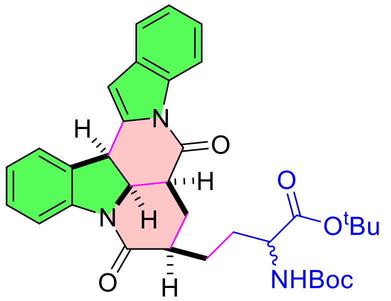

3p

tert-butyl (S)-2-((tert-butoxycarbonyl)amino)-4-6,9-dioxo-51,7,8,8a,9,15b-hexahydro-6H-diindolo[1,2-g:3',2',1'-ij][1,6]naphthyridin-7-yl)butanoate (**3p**): Yield (0.1 mmol scale; **23 W CFL**: 15 mg, 26% yield, d.r. = 1.3/1; **Purple**: 30 mg, 51% yield, d.r. = 1.3/1). A light yellow solid. R_f = 0.4 (Hexanes/EtOAc = 2/1). FC (Hexanes/EtOAc = 3/1 ~ 2/1). ^{1}H NMR (500 MHz, $CDCl_3$) δ 8.37 (d, J = 8.1 Hz, 1H), 8.09 (ddd, J = 8.1, 1.3, 1.3 Hz, 1H), 7.48 – 7.44 (m, 2H), 7.33 – 7.20 (m, 3H), 7.09 (ddd, J = 7.6, 7.6, 4.1 Hz, 1H), 6.54 (s, 1H), 5.23 (dd, J = 8.1, 5.0 Hz, 1H), 4.94 – 4.79 (m, 2H), 4.27 – 4.14 (m, 1H), 3.43 (ddd, J = 9.0, 5.3, 5.3 Hz, 1H), 2.66 – 2.49 (m, 2H), 2.46 – 2.36 (m, 1H), 2.15 – 1.96 (m, 2H), 1.96 – 1.85 (m, 1H), 1.61 – 1.55 (m, 1H), 1.50 (s, 5H), 1.47 (s, 5H), 1.46 (s, 4H), 1.45 (s, 4H). $^{13}C\{^1H\}$ NMR (126 MHz, $CDCl_3$) δ 171.8, 171.2, 168.1, 155.5, 141.0, 136.6, 135.0, 131.7, 129.8, 129.1, 124.8, 124.6, 124.6, 124.3, 120.3, 116.4, 116.1, 106.7, 81.9, 79.6, 58.5, 53.6, 40.0, 38.4, 38.2, 30.8, 28.3, 28.0, 26.1, 25.3. HRMS (ESI) m/z: $[M + H]^+$ Calcd. for $C_{34}H_{40}N_3O_6$ 586.2912; Found 586.2908.

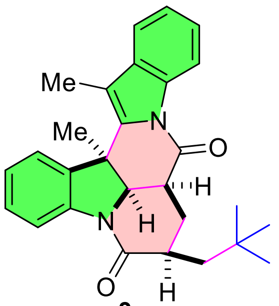

15,15b-dimethyl-7-neopentyl-51,8,8a,15b-tetrahydro-6H-diindolo[1,2-g:3',2',1'-ij][1,6]naphthyridine-6,9(7H)-dione (**3q**): Yield (0.1 mmol scale; **23 W CFL**: 29 mg, 68% yield; **Purple**: 19 mg, 45% yield). A white solid. R_f = 0.5 (Hexanes/EtOAc = 3/1). FC (Hexanes/EtOAc = 8/1). ^{1}H NMR (500 MHz, $CDCl_3$) δ 8.35 (d, *J* = 8.1 Hz, 1H), 8.06 (dd, *J* = 8.1, 1.1 Hz, 1H), 7.49 (dd, *J* = 7.6, 1.3 Hz, 1H), 7.31 (dd, *J* = 7.6, 1.6 Hz, 1H), 7.21 (ddd, *J* = 8.3, 7.2, 1.4 Hz, 1H), 7.19 – 7.11 (m, 2H), 7.03 (ddd, *J* = 7.5, 7.2, 1.1 Hz, 1H), 4.27 (d, *J* = 4.7 Hz, 1H), 3.28 (ddd, *J* = 7.7, 4.7, 3.2 Hz, 1H), 2.82 (ddd, *J* = 14.3, 5.7, 3.2 Hz, 1H), 2.51 – 2.42 (m, 1H), 2.32 – 2.25 (m, 4H), 2.08 (dd, *J* = 14.0, 2.8 Hz, 1H), 1.89 (s, 3H), 1.50 (dd, *J* = 14.1, 6.8 Hz, 1H), 0.93 (s, 9H). $^{13}C\{^1H\}$ NMR (126 MHz, $CDCl_3$) δ 172.0, 167.7, 141.6, 135.4, 134.1, 134.1, 131.8, 128.8, 125.2, 123.9, 123.7, 123.6, 118.0, 116.8, 116.4, 114.8, 69.2, 45.3, 44.4, 37.0, 31.2, 29.7, 28.5, 23.1, 10.2. HRMS (ESI) m/z: $[M + H]^+$ Calcd. for $C_{28}H_{31}N_2O_2$ 427.2380; Found 427.2377.

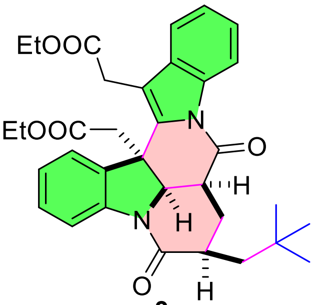

diethyl 2,2'-7-neopentyl-6,9-dioxo-7,8,8a,9-tetrahydro-6H-diindolo[1,2-g:3',2',1'-ij][1,6]naphthyridine-15,15b(51H)-diyl)diacetate (**3r**): Yield (0.1 mmol scale; **23 W CFL**: 44 mg, 77% yield; **Purple**: 31 mg, 54% yield). A white solid. R_f = 0.3 (Hexanes/EtOAc = 4/1). FC (Hexanes/EtOAc = 6/1 ~ 5/1). ^{1}H NMR (500 MHz, $CDCl_3$) δ 8.35 (ddd, *J* = 8.3, 0.9, 0.9 Hz, 1H), 8.09 (dd, *J* = 8.1, 1.2 Hz, 1H), 7.45 (dd, *J* = 7.8, 1.4 Hz, 1H), 7.38 (ddd, *J* = 7.8, 1.0, 0.9 Hz, 1H), 7.25 (ddd, *J* = 8.3, 7.2, 1.3 Hz, 1H), 7.21 – 7.14 (m, 2H), 6.99 (ddd, *J* = 7.6, 1.2, 0.9 Hz, 1H), 4.72 (d, *J* = 5.5 Hz, 1H), 4.12 – 4.04 (m, 2H), 4.04 – 3.94 (m, 2H), 3.88 (d, *J* = 16.2 Hz, 1H), 3.81 – 3.67 (m, 3H), 3.18 (d, *J* = 16.6 Hz, 1H), 2.65 (ddd, *J* = 13.9, 7.8, 4.4 Hz, 1H), 2.48 – 2.31 (m, 2H), 2.15 (dd, *J* = 14.1, 3.3 Hz, 1H), 1.32 (dd, *J* = 14.2, 5.9 Hz, 1H), 1.16 (t, *J* = 7.2 Hz, 3H), 1.05 (t, *J* = 7.2 Hz, 3H), 0.92 (s, 9H). $^{13}C\{^1H\}$ NMR (126 MHz, $CDCl_3$) δ 172.2, 170.3, 170.1, 168.8, 141.4, 134.7, 134.3, 133.1, 130.8, 129.6, 125.7, 124.1, 123.8, 123.6, 118.5, 116.9, 116.3, 112.1, 67.2, 61.3, 61.3, 46.5, 43.8, 43.1, 38.4, 37.1, 31.1, 31.0, 29.6, 28.7, 14.2, 14.0. HRMS (ESI) m/z: $[M + H]^+$ Calcd. for $C_{34}H_{39}N_2O_6$ 571.2803; Found 571.2779.

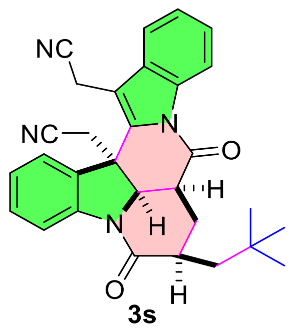

2,2'-7-neopentyl-6,9-dioxo-7,8,8a,9-tetrahydro-6H-diindolo[1,2-g:3',2',1'-ij][1,6]naphthyridine-15,15b(51H)-diyl)diacetonitrile (**3s**): Yield (0.1 mmol scale; **23 W CFL**: 34 mg, 71% yield; **Purple**: 30 mg, 63% yield). A white solid. R_f = 0.5 (Hexanes/EtOAc = 1/1). FC (Hexanes/EtOAc = 3/1 ~ 2/1). ^{1}H NMR (500 MHz, $CDCl_3$) δ 8.56 (ddd, *J* = 8.3, 1.1, 0.9 Hz, 1H), 8.24 (dd, *J* = 8.1, 1.1 Hz, 1H), 7.76 (dd, *J* = 7.7, 1.3 Hz, 1H), 7.52 (ddd, *J* = 7.8, 1.0, 0.9 Hz, 1H), 7.48 (ddd, *J* = 8.4, 7.2, 1.2 Hz, 1H), 7.45 – 7.37 (m, 2H), 7.30 (ddd, *J* = 7.8, 7.6, 1.1 Hz, 1H), 4.72 (d, *J* = 5.6 Hz, 1H), 3.76 (d, *J* = 18.1 Hz, 1H), 3.69 (ddd, *J* = 7.1, 5.6, 4.2 Hz, 1H), 3.65 (d, *J* = 18.0 Hz, 1H), 3.53 (d, *J* = 17.5 Hz, 1H), 3.46 (d, *J* = 17.5 Hz, 1H), 2.87 (ddd, *J* = 14.4, 5.3, 4.2 Hz, 1H), 2.69 – 2.59 (m, 1H), 2.51 (ddd, *J* = 14.6, 7.3, 7.3 Hz, 1H), 1.97 (dd, *J* = 14.0, 2.8 Hz, 1H), 1.48 (dd, *J* = 14.0, 6.7 Hz, 1H), 1.01 (s, 9H). ^{13}C{^{1}H} NMR (126 MHz, $CDCl_3$) δ 172.2, 167.1, 142.1, 134.3, 132.0, 130.9, 129.5, 128.6, 127.2, 125.1, 124.4, 122.7, 118.2, 117.7, 117.0, 116.9, 115.5, 110.5, 67.3, 45.9, 45.0, 37.1, 36.6, 31.3, 29.5, 29.1, 24.5, 13.2. HRMS (ESI) m/z: $[M + H]^+$ Calcd. for $C_{30}H_{29}N_4O_2$ 477.2285; Found 477.2274.

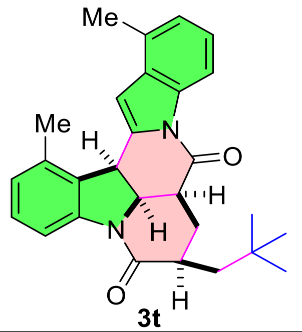

1,14-dimethyl-7-neopentyl-51,8,8a,15b-tetrahydro-6H-diindolo[1,2-g:3',2',1'-ij][1,6]naphthyridine-6,9(7H)-dione (**3t**): Yield (0.1 mmol scale; **23 W CFL**: 35 mg, 82% yield; **Purple**: 32 mg, 75% yield). A white solid. R_f = 0.5 (Hexanes/EtOAc = 3/1). FC (Hexanes/EtOAc = 8/1). ^{1}H NMR (500 MHz, CD_2Cl_2) δ 8.11 (d, *J* = 8.2 Hz, 1H), 7.84 (d, *J* = 8.0 Hz, 1H), 7.09 (ddd, *J* = 7.7, 7.4, 2.6 Hz, 2H), 6.95 (d, *J* = 7.4 Hz, 1H), 6.91 (d, *J* = 7.7 Hz, 1H), 6.33 (d, *J* = 1.7 Hz, 1H), 4.72 (dd, *J* = 7.6, 6.1 Hz, 1H), 4.66 (dd, *J* = 7.6, 1.7 Hz, 1H), 3.40 (ddd, *J* = 7.9, 6.2, 6.2 Hz, 1H), 2.54 (s, 3H), 2.47 – 2.34 (m, 6H), 2.10 (dd, *J* = 14.1, 3.1 Hz, 1H), 1.25 – 1.19 (m, 1H), 0.89 (s, 9H). ^{13}C{^{1}H} NMR (126 MHz, CD_2Cl_2) δ 172.4, 168.6, 142.0, 135.8, 134.8, 133.8, 130.7, 129.7, 129.3, 128.7, 125.9, 124.7, 124.6, 113.8, 113.5, 105.7, 60.0, 43.4, 38.6, 37.4, 37.4, 30.8, 30.6, 29.3, 19.4, 18.1. HRMS (ESI) m/z: $[M + H]^+$ Calcd. for $C_{28}H_{31}N_2O_2$ 427.2380; Found 427.2377.

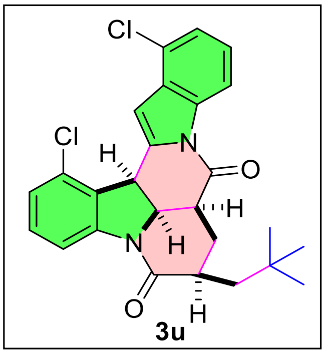

1,14-dichloro-7-neopentyl-51,8,8a,15b-tetrahydro-6H-diindolo[1,2-g:3',2',1'-ij][1,6]naphthyridine-6,9(7H)-dione (**3u**): Yield (0.1 mmol scale; **23 W CFL**: 37 mg, 79% yield; **Purple**: 30 mg, 64% yield). A white solid. R_f = 0.5 (Hexanes/EtOAc = 3/1). FC (Hexanes/EtOAc = 8/1). ^{1}H NMR (500 MHz, CD_2Cl_2) δ 8.22 (dd, *J* = 7.5, 1.5 Hz, 1H), 7.93 (dd, *J* = 8.0, 0.5 Hz, 1H), 7.21 – 7.10 (m, 3H), 7.06 (d, *J* = 8.0 Hz, 1H), 6.92 (s, 1H), 4.92 (dd, *J* = 7.5, 1.5 Hz, 1H), 4.84 (dd, *J* = 7.7, 5.3 Hz, 1H), 3.49 – 3.37 (m, 1H), 2.62 – 2.52 (m, 1H), 2.52 – 2.38 (m, 2H), 2.15 (dd, *J* = 13.7, 3.0 Hz, 1H), 1.27 (dd, *J* = 14.1, 5.6 Hz, 1H), 0.91 (s, 9H). ^{13}C{^{1}H} NMR (126 MHz, CD_2Cl_2) δ 172.4, 168.2, 143.4, 136.0, 135.5, 130.5, 130.0, 129.9, 128.7, 125.6, 125.5, 124.8, 123.9, 114.7, 114.7, 105.6, 59.8, 43.7, 38.5, 37.9, 37.2, 30.8, 29.6, 29.3. HRMS (ESI) m/z: $[M + H]^+$ Calcd. for $C_{26}H_{25}Cl_2N_2O_2$ 467.1288; Found 467.1286.

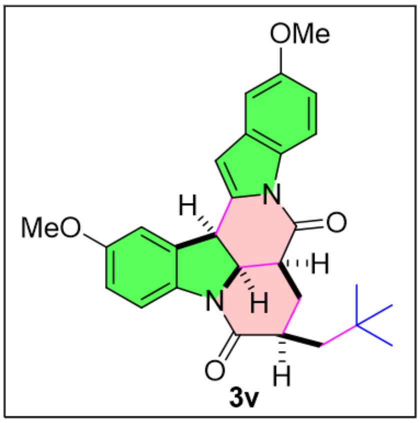

2,13-dimethoxy-7-neopentyl-51,8,8a,15b-tetrahydro-6H-diindolo[1,2-g:3',2',1'-ij][1,6]naphthyridine-6,9(7H)-dione (**3v**): Yield after the additional washing step (0.1 mmol scale; **23 W CFL**: 27 mg, 59% yield; **Purple**: 28 mg, 61% yield). A white solid. R_f = 0.2 (Hexanes/EtOAc = 3/1). FC (Hexanes/EtOAc = 4/1 ~ 2/1). ^{1}H NMR (500 MHz, $CDCl_3$) δ 8.17 (d, *J* = 9.0 Hz, 1H), 7.95 (d, *J* = 8.9 Hz, 1H), 6.92 (d, *J* = 2.6 Hz, 1H), 6.85 (d, *J* = 2.5 Hz, 1H), 6.80 (dd, *J* = 9.0, 2.6 Hz, 1H), 6.67 (dd, *J* = 8.8, 2.6 Hz, 1H), 6.37 (dd, *J* = 1.0, 0.5 Hz, 1H), 4.83 (dd, *J* = 8.8, 6.4 Hz, 1H), 4.67 (d, *J* = 8.7 Hz, 1H), 3.73 (s, 3H), 3.72 (s, 3H), 3.32 (ddd, *J* = 8.9, 6.0, 6.0 Hz, 1H), 2.53 – 2.32 (m, 3H), 2.24 (dd, *J* = 14.1, 3.2 Hz, 1H), 1.23 (dd, *J* = 14.2, 5.6 Hz, 1H), 0.92 (s, 9H). ^{13}C{^{1}H} NMR (126 MHz, $CDCl_3$) δ 171.7, 167.8, 156.9, 156.8, 137.1, 135.2, 133.1, 131.0, 129.7, 117.1, 117.0, 113.0, 112.9, 111.4, 106.5, 103.4, 58.8, 55.8, 55.7, 43.0, 38.7, 38.3, 37.1, 30.9, 29.6, 29.6. HRMS (ESI) m/z: $[M + H]^+$ Calcd. for $C_{28}H_{31}N_2O_4$ 459.2278; Found 459.2276.

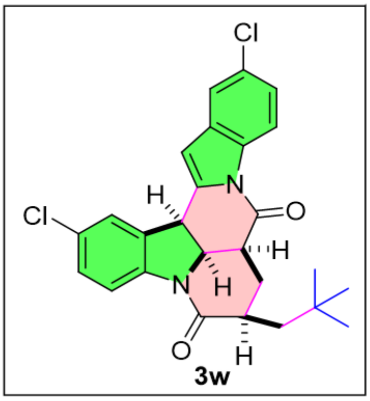

2,13-dichloro-7-neopentyl-51,8,8a,15b-tetrahydro-6H-diindolo[1,2-g:3',2',1'-ij][1,6]naphthyridine-6,9(7H)-dione (**3w**): Yield after the additional washing step (0.1 mmol scale; **23 W CFL**: 26 mg, 56% yield; **Purple**: 24 mg, 51% yield). A white solid. R_f = 0.4 (Hexanes/EtOAc = 3/1). FC (Hexanes/EtOAc = 6/1 ~ 5/1). ^{1}H NMR (500 MHz, $CDCl_3$) δ 8.27 (d, J = 8.8 Hz, 1H), 8.04 (d, J = 8.6 Hz, 1H), 7.46 (d, J = 2.1 Hz, 1H), 7.38 (d, J = 2.1 Hz, 1H), 7.25 (dd, J = 8.7, 2.1 Hz, 1H), 7.21 (dd, J = 8.6, 2.2 Hz, 1H), 6.50 (dd, J = 1.0, 0.5 Hz, 1H), 5.00 (dd, J = 9.1, 6.4 Hz, 1H), 4.85 (d, J = 9.1 Hz, 1H), 3.45 (ddd, J = 8.8, 6.3, 6.3 Hz, 1H), 2.61 – 2.50 (m, 2H), 2.50 – 2.43 (m, 1H), 2.35 (dd, J = 14.2, 3.5 Hz, 1H), 1.34 – 1.19 (m, 2H), 0.99 (s, 9H). ^{13}C{^{1}H} NMR (126 MHz, $CDCl_3$) δ 172.2, 167.9, 140.0, 137.3, 133.4, 133.0, 131.0, 130.0, 129.3, 129.3, 125.1, 124.8, 120.1, 117.4, 117.1, 105.9, 58.5, 42.7, 39.0, 38.0, 37.3, 30.8, 29.6, 29.3. HRMS (ESI) m/z: $[M + H]^+$ Calcd. for $C_{26}H_{25}Cl_2N_2O_2$ 467.1288; Found 467.1283.

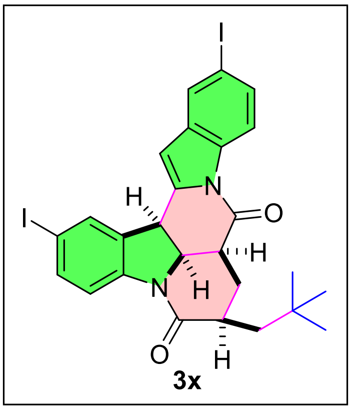

2,13-diiodo-7-neopentyl-51,8,8a,15b-tetrahydro-6H-diindolo[1,2-g:3',2',1'-ij][1,6]naphthyridine-6,9(7H)-dione (**3x**): Yield (0.1 mmol scale; **23 W CFL**: 46 mg, 71% yield; **Purple**: 35 mg, 54% yield). A white solid. R_f = 0.4 (Hexanes/EtOAc = 5/1). FC (Hexanes/EtOAc = 8/1). ^{1}H NMR (500 MHz, $CDCl_3$) δ 7.98 (d, J = 8.7 Hz, 1H), 7.78 (d, J = 8.5 Hz, 1H), 7.73 (d, J = 1.8 Hz, 1H), 7.60 (d, J = 1.8 Hz, 1H), 7.47 – 7.43 (m, 2H), 6.37 (s, 1H), 4.89 (dd, J = 9.0, 6.1 Hz, 1H), 4.75 (d, J = 8.9 Hz, 1H), 3.34 (ddd, J = 8.6, 6.0, 6.0 Hz, 1H), 2.53 – 2.40 (m, 2H), 2.40 – 2.33 (m, 1H), 2.27 (dd, J = 14.2, 3.4 Hz, 1H), 1.21 – 1.17 (m, 1H), 0.91 (s, 9H). ^{13}C{^{1}H} NMR (126 MHz, $CDCl_3$) δ 172.2, 168.0, 141.1, 138.1, 136.9, 134.3, 133.6, 133.5, 133.4, 132.0, 129.3, 118.3, 117.8, 105.5, 88.7, 86.9, 58.3, 42.6, 39.1, 37.8, 37.4, 30.8, 29.6, 29.2. HRMS (ESI) m/z: $[M + H]^+$ Calcd. for $C_{26}H_{25}I_2N_2O_2$ 651.0000; Found 650.9998.

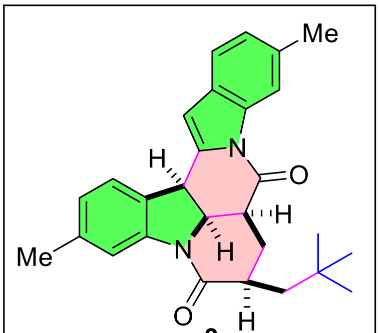

3,12-dimethyl-7-neopentyl-51,8,8a,15b-tetrahydro-6H-diindolo[1,2-g:3',2',1'-ij][1,6]naphthyridine-6,9(7H)-dione (**3y**): Yield (0.1 mmol scale; **23 W CFL**: 30 mg, 70% yield). A white solid. R_f = 0.5 (Hexanes/EtOAc = 3/1). FC (Hexanes/EtOAc = 8/1). ^{1}H NMR (500 MHz, $CDCl_3$) δ 8.10 (dd, J = 1.7, 0.9 Hz, 1H), 7.92 – 7.86 (m, 1H), 7.25 (d, J = 7.9 Hz, 1H), 7.21 (d, J = 7.7 Hz, 1H), 6.98 (dd, J = 7.9, 1.5 Hz, 1H), 6.84 – 6.77 (m, 1H), 6.38 (dd, J = 1.0, 1.0 Hz, 1H), 4.83 (dd, J = 9.1, 6.5 Hz, 1H), 4.70 (d, J = 9.0 Hz, 1H), 3.31 (ddd, J = 8.9, 6.2, 6.2 Hz, 1H), 2.50 – 2.33 (m, 6H), 2.29 (dd, J = 14.1, 3.5 Hz, 1H), 2.22 (s, 3H), 1.21 (dd, J = 14.1, 5.1 Hz, 1H), 0.92 (s, 9H). ^{13}C{^{1}H} NMR (126 MHz, $CDCl_3$) δ 172.3, 168.3, 141.4, 139.3, 136.3, 135.5, 134.9, 128.9, 127.6, 125.6, 125.0, 124.2, 119.8, 117.1, 116.3, 106.3, 58.6, 42.8, 39.2, 38.0, 37.4, 30.8, 29.6, 29.6, 21.9, 21.6. HRMS (ESI) m/z: $[M + H]^+$ Calcd. for $C_{28}H_{31}N_2O_2$ 427.2380; Found 427.2360.

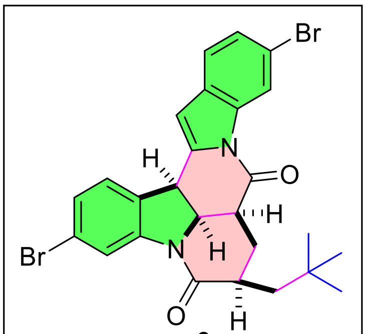

3,12-dibromo-7-neopentyl-51,8,8a,15b-tetrahydro-6H-diindolo[1,2-g:3',2',1'-ij][1,6]naphthyridine-6,9(7H)-dione (**3z**): Yield after the additional washing step (0.1 mmol scale; **23 W CFL**: 35 mg, 63% yield; **Purple**: 31 mg, 56% yield). A white solid. R_f = 0.4 (Hexanes/EtOAc = 3/1). FC (Hexanes/EtOAc = 6/1 ~ 5/1). ^{1}H NMR (500 MHz, $CDCl_3$) δ 8.54 (d, J = 1.7 Hz, 1H), 8.31 (d, J = 1.8 Hz, 1H), 7.36 (dd, J = 8.3, 1.7 Hz, 1H), 7.31 (d, J = 8.4 Hz, 1H), 7.27 (d, J = 8.1 Hz, 1H), 7.19 (dd, J = 8.0, 1.8 Hz, 1H), 6.47 (dd, J = 1.0, 1.0 Hz, 1H), 4.99 (dd, J = 9.1, 6.4 Hz, 1H), 4.81 (d, J = 9.0 Hz, 1H), 3.51 – 3.40 (m, 1H), 2.57 – 2.50 (m, 2H), 2.50 – 2.43 (m, 1H), 2.34 (dd, J = 14.1, 3.5 Hz, 1H), 1.28 (dd, J = 14.2, 5.2 Hz, 1H), 1.00 (s, 9H). ^{13}C{^{1}H} NMR (126 MHz, $CDCl_3$) δ 172.4, 168.0, 142.4, 136.7, 135.5, 130.3, 128.6, 127.6, 127.4, 125.7, 122.9, 121.4, 119.6, 119.2, 118.4, 106.2, 58.7, 42.8, 39.0, 37.8, 37.3, 30.9, 29.6, 29.3. HRMS (ESI) m/z: $[M + H]^+$ Calcd. for $C_{26}H_{25}Br_2N_2O_2$ 555.0277; Found 555.0280.

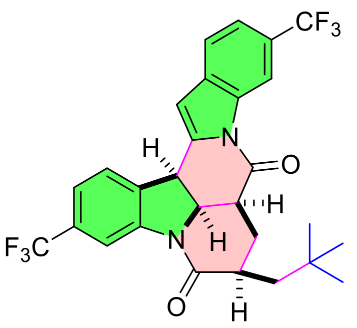

3aa

7-neopentyl-3,12-bis(trifluoromethyl)-51,8,8a,15b-tetrahydro-6H-diindolo[1,2-g:3',2',1'-ij][1,6]naphthyridine-6,9(7H)-dione (**3aa**): Yield after the additional washing step (0.1 mmol scale; **23 W CFL**: 20 mg, 37% yield; **Purple**: 23 mg, 43% yield). A white solid. R_f = 0.2 (Hexanes/EtOAc = 3/1). FC (Hexanes/EtOAc = 5/1 ~ 4/1). ^{1}H NMR (500 MHz, $CDCl_3$) δ 8.60 (s, 1H), 8.35 (d, J = 1.7 Hz, 1H), 7.49 (dd, J = 10.5, 8.1 Hz, 2H), 7.43 (dd, J = 8.2, 1.6 Hz, 1H), 7.29 (dd, J = 7.9, 1.7 Hz, 1H), 6.54 (s, 1H), 5.00 (dd, J = 9.1, 6.2 Hz, 1H), 4.91 (d, J = 8.9 Hz, 1H), 3.45 (ddd, J = 8.4, 6.2, 6.2 Hz, 1H), 2.58 – 2.48 (m, 2H), 2.48 – 2.39 (m, 1H), 2.30 (dd, J = 14.2, 3.5 Hz, 1H), 1.24 (dd, J = 14.2, 5.3 Hz, 1H), 0.93 (s, 9H). ^{13}C{^{1}H} NMR (126 MHz, $CDCl_3$) δ 172.5, 167.9, 141.8, 138.4, 134.7, 134.2, 132.3, 131.8 (q, J = 32.6 Hz), 127.2 (q, J = 32.3 Hz), 125.6, 124.9, 123.5, 121.5 (q, J = 3.9 Hz), 121.2 (q, J = 3.7 Hz), 120.7, 113.7 (q, J = 4.3 Hz), 113.6 (q, J = 4.4 Hz), 106.3, 58.6, 42.8, 39.1, 38.2, 37.4, 30.8, 29.6, 29.3. ^{19}F NMR (471 MHz, $CDCl_3$) δ -61.09, -62.46. HRMS (ESI) m/z: $[M + H]^+$ Calcd. for $C_{28}H_{25}F_6N_2O_2$ 535.1815; Found 535.1816.

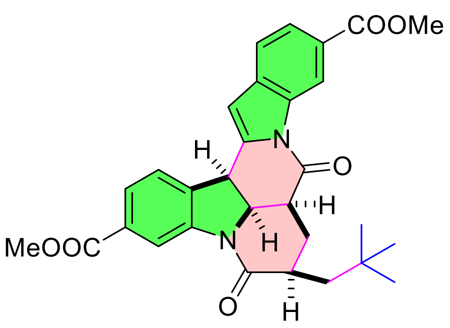

3ab

dimethyl 7-neopentyl-6,9-dioxo-51,7,8,8a,9,15b-hexahydro-6H-diindolo[1,2-g:3',2',1'-ij][1,6]naphthyridine-3,12-dicarboxylate (**3ab**): Yield (0.1 mmol scale; **23 W CFL**: 12 mg, 23% yield; **Purple**: 25 mg, 49% yield). A white solid. R_f = 0.3 (Hexanes/EtOAc = 2/1). FC (Hexanes/EtOAc = 3/1 ~ 2/1). ^{1}H NMR (500 MHz, $CDCl_3$) δ 8.91 (d, J = 1.5 Hz, 1H), 8.63 (d, J = 1.6 Hz, 1H), 7.85 (dd, J = 8.2, 1.5 Hz, 1H), 7.70 (dd, J = 7.9, 1.6 Hz, 1H), 7.39 (dd, J = 8.1, 3.9 Hz, 2H), 6.46 (s, 1H), 4.98 (dd, J = 9.0, 6.3 Hz, 1H), 4.84 (d, J = 8.9 Hz, 1H), 3.83 (s, 3H), 3.77 (s, 3H), 3.51 – 3.41 (m, 1H), 2.51 – 2.47 (m, 2H), 2.46 – 2.38 (m, 1H), 2.30 (dd, J = 14.2, 3.4 Hz, 1H), 1.22 (dd, J = 14.2, 5.2 Hz, 1H), 0.92 (s, 9H). ^{13}C{^{1}H} NMR (126 MHz, $CDCl_3$) δ 172.4, 168.0, 167.3, 166.4, 141.6, 139.0, 135.8, 134.4, 133.4, 131.4, 126.6, 126.3, 125.6, 124.4, 120.0, 117.7, 117.2, 106.4, 58.6, 52.2, 52.2, 42.9, 39.1, 38.3, 37.3, 30.8, 29.6, 29.4. HRMS (ESI) m/z: $[M + H]^+$ Calcd. for $C_{30}H_{31}N_2O_6$ 515.2177; Found 515.2150.

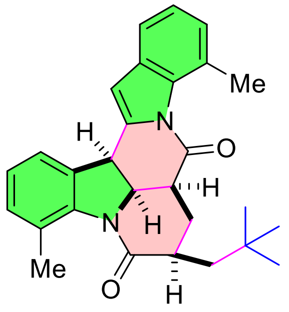

3ac

4,11-dimethyl-7-neopentyl-51,8,8a,15b-tetrahydro-6H-diindolo[1,2-g:3',2',1'-ij][1,6]naphthyridine-6,9(7H)-dione (**3ac**): Yield (0.1 mmol scale; **23 W CFL**: 15 mg, 35% yield; **Purple**: 14 mg, 33% yield). A white solid. $R_f = 0.5$ (Hexanes/EtOAc = 3/1). FC (Hexanes/EtOAc = 8/1). ^{1}H NMR (500 MHz, $CDCl_3$) δ 7.28 (dd, J = 6.4, 2.3 Hz, 1H), 7.16 (d, J = 7.6 Hz, 1H), 7.10 – 7.01 (m, 3H), 6.99 (dd, J = 7.3, 1.2 Hz, 1H), 6.36 (d, J = 1.6 Hz, 1H), 4.77 (dd, J = 7.4, 6.2 Hz, 1H), 4.40 (dd, J = 7.4, 1.5 Hz, 1H), 3.35 (ddd, J = 5.8, 5.8, 3.6 Hz, 1H), 2.79 (ddd, J = 14.1, 3.6, 3.4 Hz, 1H), 2.63 – 2.57 (m, 1H), 2.54 (s, 3H), 2.44 (ddd, J = 14.1, 8.5, 5.6 Hz, 1H), 2.20 (s, 3H), 2.00 (dd, J = 13.8, 2.8 Hz, 1H), 1.50 (dd, J = 13.8, 6.9 Hz, 1H), 0.92 (s, 9H). ^{13}C{^{1}H} NMR (126 MHz, $CDCl_3$) δ 171.4, 167.6, 140.8, 138.1, 135.2, 134.4, 131.8, 131.5, 129.4, 128.1, 127.1, 125.8, 124.8, 121.6, 117.9, 108.5, 62.7, 45.1, 40.6, 39.4, 36.8, 31.3, 30.8, 29.5, 23.0, 21.1. HRMS (ESI) m/z: $[M + H]^+$ Calcd. for $C_{28}H_{31}N_2O_2$ 427.2380; Found 427.2380.

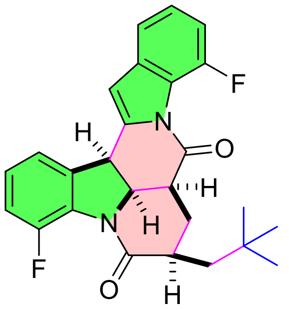

3ad

4,11-difluoro-7-neopentyl-51,8,8a,15b-tetrahydro-6H-diindolo[1,2-g:3',2',1'-ij][1,6]naphthyridine-6,9(7H)-dione (**3ad**): Yield (0.1 mmol scale; **23 W CFL**: 19 mg, 44% yield; **Purple**: 21 mg, 48% yield). A white solid. $R_f = 0.3$ (Hexanes/EtOAc = 2/1). FC (Hexanes/EtOAc = 3/1 ~ 1.5/1). ^{1}H NMR (500 MHz, $CDCl_3$) δ 7.24 (d, J = 7.3 Hz, 1H), 7.16 – 7.03 (m, 3H), 7.02 – 6.87 (m, 2H), 6.37 (s, 1H), 4.86 (dd, J = 6.9, 6.9 Hz, 1H), 4.56 (d, J = 7.3 Hz, 1H), 3.42 (ddd, J = 6.3, 6.3, 3.8 Hz, 1H), 2.77 (ddd, J = 14.3, 4.2, 4.2 Hz, 1H), 2.62 – 2.57 (m, 1H), 2.43 (ddd, J = 14.2, 7.1, 7.1 Hz, 1H), 1.94 (dd, J = 14.1, 2.8 Hz, 1H), 1.47 (dd, J = 14.1, 6.7 Hz, 1H), 0.90 (s, 9H). ^{13}C{^{1}H} NMR (126 MHz, $CDCl_3$) δ 170.7, 166.1, 151.5 (d, J = 256.5 Hz), 150.4 (d, J = 255.5 Hz), 137.8, 136.3 (d, J = 3.3 Hz), 133.9 (d, J = 4.2 Hz), 128.3 (d, J = 12.3 Hz), 126.7 (d, J = 6.9 Hz), 125.6 (d, J = 7.1 Hz), 121.7 (d, J = 11.7 Hz), 120.1 (d, J = 3.4 Hz), 117.8 (d, J = 21.9 Hz), 116.2 (d, J = 3.8 Hz), 112.2 (d, J = 22.1 Hz), 108.3, 62.1, 44.5, 40.1, 38.8 (d, J = 2.3 Hz), 36.7 (d, J = 2.0 Hz), 31.2, 30.0, 29.5. ^{19}F NMR (471 MHz, $CDCl_3$) δ -109.39, -110.89. HRMS (ESI) m/z: $[M + H]^+$ Calcd. for $C_{26}H_{25}F_2N_2O_2$ 435.1879; Found 435.1863.

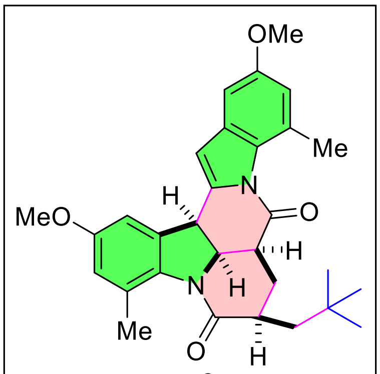

2,13-dimethoxy-4,11-dimethyl-7-neopentyl-51,8,8a,15b-tetrahydro-6H-diindolo[1,2-g:3',2',1'-ij][1,6]naphthyridine-6,9(7H)-dione (**3ae**): Yield after the additional washing step (0.1 mmol scale; **23 W CFL**: 21 mg, 43% yield; **Purple**: 21 mg, 43% yield). A white solid. R_f = 0.3 (Hexanes/EtOAc = 3/1). FC (Hexanes/EtOAc = 6/1 ~ 3/1). ^{1}H NMR (500 MHz, $CDCl_3$) δ 6.92 (d, J = 2.6 Hz, 1H), 6.70 (d, J = 2.6 Hz, 1H), 6.66 (d, J = 2.5 Hz, 1H), 6.63 (d, J = 2.5 Hz, 1H), 6.37 (d, J = 1.5 Hz, 1H), 4.77 (dd, J = 6.7, 6.7 Hz, 1H), 4.35 (dd, J = 7.2, 1.6 Hz, 1H), 3.82 (s, 3H), 3.78 (s, 3H), 3.36 (ddd, J = 5.7, 5.7, 3.5 Hz, 1H), 2.86 (ddd, J = 14.2, 3.6, 2.4 Hz, 1H), 2.68 – 2.62 (m, 1H), 2.59 (s, 3H), 2.47 (ddd, J = 13.9, 8.4, 5.4 Hz, 1H), 2.27 (s, 3H), 1.99 (dd, J = 13.7, 2.6 Hz, 1H), 1.57 (dd, J = 13.8, 7.0 Hz, 1H), 0.98 (s, 9H). ^{13}C{^{1}H} NMR (126 MHz, $CDCl_3$) δ 171.3, 167.3, 157.9, 157.1, 138.4, 135.8, 134.4, 133.0, 130.6, 129.8, 128.3, 116.0, 115.5, 108.6, 108.2, 100.7, 62.9, 55.7, 55.5, 45.2, 40.8, 38.9, 36.5, 31.4, 30.6, 29.5, 23.1, 21.3. HRMS (ESI) m/z: $[M + H]^+$ Calcd. for $C_{30}H_{35}N_2O_4$ 487.2591; Found 487.2588.

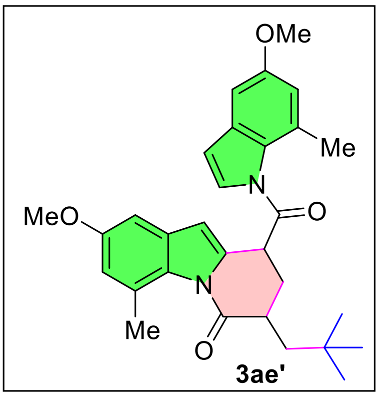

2-methoxy-9-(5-methoxy-7-methyl-1H-indole-1-carbonyl)-4-methyl-7-neopentyl-8,9-dihydropyrido[1,2-a]indol-6(7H)-one (**3ae'**): Yield (0.1 mmol scale; **23 W CFL**: 5 mg, 10% yield; **Purple**: 12 mg, 25% yield). A white solid. R_f = 0.5 (Hexanes/EtOAc = 3/1). FC (Hexanes/EtOAc = 8/1 ~ 6/1). ^{1}H NMR (500 MHz, $CDCl_3$) δ 7.46 (d, J = 3.8 Hz, 1H), 6.83 (d, J = 2.6 Hz, 1H), 6.72 (d, J = 2.5 Hz, 1H), 6.67 – 6.64 (m, 2H), 6.59 (d, J = 3.8 Hz, 1H), 6.21 (d, J = 1.3 Hz, 1H), 4.80 – 4.71 (m, 1H), 3.78 (s, 3H), 3.72 (s, 3H), 3.16 (ddd, J = 7.6, 7.6, 5.0 Hz, 1H), 2.69 (ddd, J = 13.5, 7.5, 4.9 Hz, 1H), 2.49 (s, 3H), 2.38 (s, 3H), 2.25 (ddd, J = 13.0, 7.6, 5.1 Hz, 1H), 2.13 (dd, J = 14.1, 4.8 Hz, 1H), 1.33 (dd, J = 14.1, 5.4 Hz, 1H), 0.95 (s, 9H). ^{13}C{^{1}H} NMR (126 MHz, $CDCl_3$) δ 171.6, 168.8, 157.1, 157.0, 136.7, 133.2, 131.9, 130.2, 130.1, 128.2, 128.0, 126.3, 116.8, 116.3, 110.2, 107.7, 101.2, 100.6, 55.6, 55.5, 44.8, 39.6, 38.7, 33.6, 31.4, 29.6, 22.8, 22.4. HRMS (ESI) m/z: $[M + H]^+$ Calcd. for $C_{30}H_{35}N_2O_4$ 487.2591; Found 487.2587.

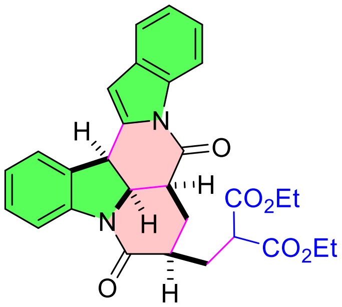

5a

diethyl 2-((6,9-dioxo-51,7,8,8a,9,15b-hexahydro-6H-diindolo[1,2-g:3',2',1'-ij][1,6]naphthyridin-7-yl)methyl)malonate (**5a**): Yield (0.1 mmol scale; **23 W CFL**: 22 mg, 44% yield; **Blue**: 25 mg, 50% yield). A white solid. R_f = 0.3 (Hexanes/EtOAc = 2/1). FC (Hexanes/EtOAc = 3/1 ~ 2/1). ^{1}H NMR (500 MHz, $CDCl_3$) δ 8.26 (d, J = 8.0 Hz, 1H), 8.01 (d, J = 7.9 Hz, 1H), 7.40 (dd, J = 7.6, 2.0 Hz, 1H), 7.36 (d, J = 7.6 Hz, 1H), 7.22 – 7.14 (m, 3H), 7.01 (ddd, J = 7.6, 7.6, 1.1 Hz, 1H), 6.48 (s, 1H), 4.84 – 4.79 (m, 2H), 4.22 – 4.06 (m, 4H), 3.89 (dd, J = 8.8, 6.3 Hz, 1H), 3.36 (ddd, J = 9.4, 4.8, 4.8 Hz, 1H), 2.62 – 2.49 (m, 2H), 2.49 – 2.36 (m, 2H), 2.03 (ddd, J = 14.0, 8.8, 4.9 Hz, 1H), 1.22 – 1.18 (m, 6H). ^{13}C{^{1}H} NMR (126 MHz, $CDCl_3$) δ 170.8, 169.6, 169.3, 168.0, 140.9, 136.6, 135.1, 131.7, 129.9, 129.1, 124.9, 124.7, 124.6, 124.3, 120.3, 116.4, 116.1, 106.5, 61.5, 61.5, 58.3, 50.0, 38.7, 38.2, 38.1, 29.9, 26.7, 14.1. HRMS (ESI) m/z: $[M + H]^+$ Calcd. for $C_{29}H_{29}N_2O_6$ 501.2020; Found 501.2020.

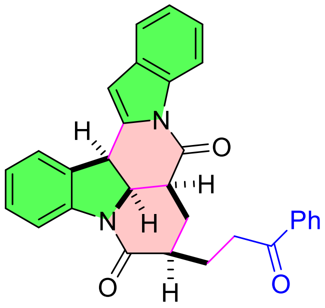

5b

7-(3-oxo-3-phenylpropyl)-51,8,8a,15b-tetrahydro-6H-diindolo[1,2-g:3',2',1'-ij][1,6]naphthyridine-6,9(7H)-dione (**5b**): Yield (0.1 mmol scale; **23 W CFL**: 11 mg, 24% yield; **Blue**: 19 mg, 41% yield). A white solid. R_f = 0.3 (Hexanes/EtOAc = 2/1). FC (Hexanes/EtOAc = 3/1 ~ 2/1). ^{1}H NMR (500 MHz, $CDCl_3$) δ 8.38 (d, J = 8.0 Hz, 1H), 8.12 (d, J = 8.0 Hz, 1H), 8.08 – 8.00 (m, 2H), 7.58 (dd, J = 7.4, 7.4 Hz, 1H), 7.53 – 7.44 (m, 4H), 7.33 – 7.25 (m, 3H), 7.11 (ddd, J = 7.5, 7.5, 1.1 Hz, 1H), 6.57 (s, 1H), 4.96 – 4.85 (m, 2H), 3.50 – 3.38 (m, 1H), 3.45 – 3.37 (m, 1H), 3.32 (ddd, J = 17.4, 7.3, 6.0 Hz, 1H), 2.78 – 2.64 (m, 2H), 2.59 – 2.51 (m, 1H), 2.35 – 2.28 (m, 1H), 2.14 – 2.06 (m, 1H). ^{13}C{^{1}H} NMR (126 MHz, $CDCl_3$) δ 200.2, 171.6, 168.2, 141.0, 136.9, 136.7, 135.1, 133.1, 131.7, 129.9, 129.1, 128.6, 128.2, 124.9, 124.7, 124.5, 124.3, 120.3, 116.4, 116.1, 106.6, 58.5, 39.7, 38.8, 38.3, 36.3, 26.7, 25.4. HRMS (ESI) m/z: $[M + H]^+$ Calcd. for $C_{30}H_{25}N_2O_3$ 461.1860; Found 461.1860.

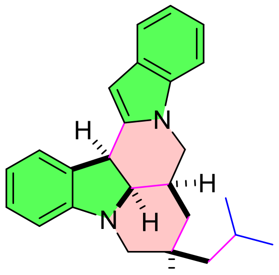

6a

7-isobutyl-5[1],7,8,8a,9,15b-hexahydro-6H-diindolo[1,2-g:3',2',1'-ij][1,6]naphthyridine (**6a**): Yield (0.2 mmol scale, 38 mg, 53% yield). A white solid. R_f = 0.7 (Hexanes/EtOAc = 6/1). FC (Hexanes/EtOAc = 25/1). ^{1}H NMR (500 MHz, $CDCl_3$) δ 7.62 (ddd, *J* = 7.8, 1.0, 1.0 Hz, 1H), 7.32 (dd, *J* = 8.1, 1.0 Hz, 1H), 7.26 – 7.21 (m, 1H), 7.18 (ddd, *J* = 8.2, 7.0, 1.2 Hz, 1H), 7.15 – 7.04 (m, 2H), 6.65 (ddd, *J* = 7.4, 7.4, 1.0 Hz, 1H), 6.54 (s, 1H), 6.43 (d, *J* = 7.8 Hz, 1H), 4.87 (d, *J* = 9.6 Hz, 1H), 4.19 (dd, *J* = 11.7, 4.9 Hz, 1H), 4.07 (dd, *J* = 9.7, 4.1 Hz, 1H), 3.88 (dd, *J* = 12.0, 12.0 Hz, 1H), 3.32 (dd, *J* = 11.5, 5.1 Hz, 1H), 3.21 (dd, *J* = 11.4, 4.4 Hz, 1H), 2.44 – 2.35 (m, 1H), 2.20 – 2.13 (m, 1H), 2.12 – 2.04 (m, 1H), 1.81 – 1.72 (m, 1H), 1.59 – 1.47 (m, 2H), 1.36 – 1.25 (m, 1H), 1.00 (d, *J* = 6.4 Hz, 3H), 0.99 (d, *J* = 6.4 Hz, 3H). ^{13}C{^{1}H} NMR (126 MHz, $CDCl_3$) δ 151.4, 136.9, 135.7, 130.6, 128.5, 128.3, 124.9, 120.4, 120.1, 119.5, 117.4, 108.7, 105.3, 97.6, 61.4, 49.7, 44.9, 43.2, 39.5, 33.6, 32.0, 30.1, 26.2, 23.0, 22.7. HRMS (ESI) m/z: $[M + H]^+$ Calcd. for $C_{25}H_{29}N_2$ 357.2325; Found 357.2328.

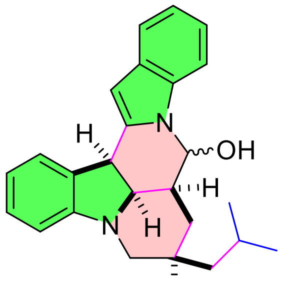

6b

7-isobutyl-5[1],7,8,8a,9,15b-hexahydro-6H-diindolo[1,2-g:3',2',1'-ij][1,6]naphthyridin-9-ol (**6b**): Yield (0.2 mmol scale, 58 mg, 78% yield). A white solid. R_f = 0.3 (Hexanes/EtOAc = 2/1). FC (Hexanes/EtOAc = 2/1 ~ 1/1). ^{1}H NMR (500 MHz, $CDCl_3$) δ 7.43 (d, *J* = 7.8 Hz, 1H), 7.41 – 7.33 (m, 2H), 7.08 (ddd, *J* = 8.2, 6.9, 1.2 Hz, 1H), 7.03 – 6.94 (m, 2H), 6.82 (ddd, *J* = 7.5, 1.2, 0.9 Hz, 1H), 6.62 (d, *J* = 7.8 Hz, 1H), 6.33 (s, 1H), 5.80 (d, *J* = 1.8 Hz, 1H), 5.26 (bs, 1H), 4.73 (d, *J* = 8.7 Hz, 1H), 4.32 (ddd, *J* = 8.7, 1.9, 1.9 Hz, 1H), 3.64 (dd, *J* = 10.4, 3.9 Hz, 1H), 3.42 (dd, *J* = 10.4, 6.9 Hz, 1H), 2.32 – 2.21 (m, 1H), 1.95 (dd, *J* = 13.5, 13.5 Hz, 1H), 1.89 – 1.75 (m, 2H), 1.68 – 1.59 (m, 1H), 1.23 – 1.07 (m, 2H), 0.86 (d, *J* = 3.0 Hz, 3H), 0.85 (d, *J* = 3.0 Hz, 3H). ^{13}C{^{1}H} NMR (126 MHz, $CDCl_3$) δ 147.9, 135.4, 134.6, 133.0, 128.9, 128.3, 125.2, 121.9, 121.3, 120.5, 120.0, 112.1, 109.5, 98.9, 65.9, 59.6, 41.7, 41.0, 37.5, 34.9, 32.1, 25.5, 23.1, 22.9. HRMS (ESI) m/z: $[M + H]^+$ Calcd. for $C_{25}H_{29}N_2O$ 373.2274; Found 373.2270.

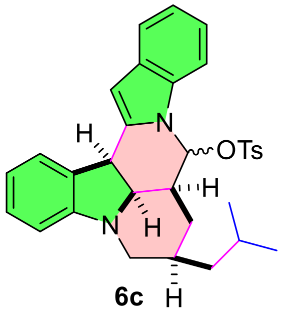

6c

7-isobutyl-5[1],7,8,8a,9,15b-hexahydro-6H-diindolo[1,2-g:3',2',1'-ij][1,6]naphthyridin-9-yl 4-methylbenzenesulfonate (**6c**): Yield (0.12 mmol scale, 17 mg, 27% yield; 20 mg (44% yield) of SM-**6b** recovered; 48% yield BRSM). A white solid. R_f = 0.4 (Hexanes/EtOAc = 2/1). FC (Hexanes/EtOAc = 8/1 ~ 5/1). ^{1}H NMR (500 MHz, $CDCl_3$) δ 7.77 (d, J = 8.3 Hz, 2H), 7.54 (ddd, J = 8.0, 1.0, 1.0 Hz, 1H), 7.49 (d, J = 7.5 Hz, 1H), 7.44 (dd, J = 8.2, 0.9 Hz, 1H), 7.29 (d, J = 7.6 Hz, 2H), 7.18 (ddd, J = 8.2, 7.0, 1.2 Hz, 1H), 7.15 – 7.06 (m, 2H), 6.93 (ddd, J = 7.5, 7.5, 1.0 Hz, 1H), 6.73 (d, J = 7.9 Hz, 1H), 6.44 (dd, J = 1.0, 1.0 Hz, 1H), 5.80 (ddd, J = 11.7, 3.2, 1.5 Hz, 1H), 5.63 (d, J = 11.7 Hz, 1H), 4.81 (d, J = 8.6 Hz, 1H), 4.38 (d, J = 5.0 Hz, 1H), 4.36 – 4.29 (m, 1H), 4.20 (dd, J = 10.0, 3.8 Hz, 1H), 3.97 (dd, J = 10.0, 6.6 Hz, 1H), 2.43 (s, 3H), 2.26 – 2.19 (m, 1H), 2.17 – 2.09 (m, 1H), 1.98 – 1.94 (m, 2H), 1.73 – 1.62 (m, 1H), 0.93 (d, J = 6.5 Hz, 6H). $^{13}C\{^1H\}$ NMR (126 MHz, $CDCl_3$) δ 148.2, 145.1, 135.4, 134.4, 132.8, 132.6, 130.0, 128.9, 128.3, 127.8, 125.1, 121.7, 121.3, 120.5, 120.0, 111.9, 109.5, 98.9, 72.7, 59.7, 41.3, 41.1, 37.2, 32.7, 31.9, 25.2, 23.0, 22.5, 21.6. HRMS (ESI) m/z: $[M + H]^+$ Calcd. for $C_{32}H_{35}N_2O_3S$ 527.2363; Found 527.2361.

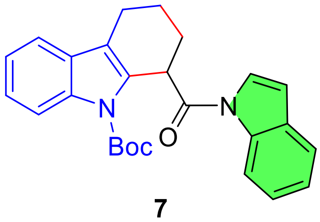

7

tert-butyl 1-(1H-indole-1-carbonyl)-1,2,3,4-tetrahydro-9H-carbazole-9-carboxylate (**7**): Yield (0.1 mmol scale, 21 mg, 51% yield). A white solid. R_f = 0.5 (Hexanes/EtOAc = 5/1). FC (Hexanes/EtOAc = 15/1). ^{1}H NMR (500 MHz, $CDCl_3$) δ 8.46 (d, J = 8.2 Hz, 1H), 8.03 (d, J = 8.2 Hz, 1H), 7.67 (d, J = 3.8 Hz, 1H), 7.59 (ddd, J = 7.6, 1.0, 1.0 Hz, 1H), 7.51 – 7.44 (m, 1H), 7.37 – 7.18 (m, 4H), 6.70 (dd, J = 3.8, 0.7 Hz, 1H), 5.12 – 5.03 (m, 1H), 2.85 (ddd, J = 16.3, 4.5, 4.5 Hz, 1H), 2.72 – 2.65 (m, 1H), 2.35 – 2.19 (m, 2H), 2.03 – 1.82 (m, 2H), 1.46 (s, 9H). $^{13}C\{^1H\}$ NMR (126 MHz, $CDCl_3$) δ 172.1, 150.7, 136.1, 135.8, 131.3, 130.3, 129.3, 125.1, 125.1, 124.3, 123.5, 122.5, 120.7, 119.8, 118.3, 116.9, 115.8, 108.9, 84.0, 42.3, 28.3, 28.1, 21.0, 19.7. HRMS (ESI) m/z: $[M + H]^+$ Calcd. for $C_{26}H_{27}N_2O_3$ 415.2016; Found 415.2016.

15. X-ray crystallographic information of compounds 3a and 3j

X-ray data were collected from a shock-cooled single crystal at 150.00(10) K on a XtaLAB Synergy, Dualflex, HyPix four-circle diffractometer with a micro-focus sealed X-ray tube using a mirror as monochromator and a HyPix detector. The diffractometer was equipped with an Oxford Cryostream 800 low temperature device and used Cu K_{α} radiation (λ = 1.54184 Å).

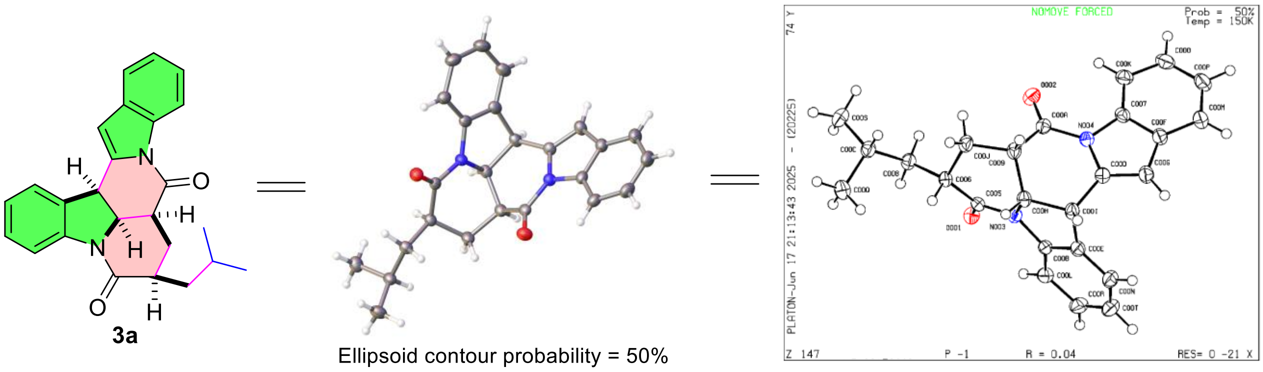

3a

Ellipsoid contour probability = 50%

X-ray crystals were obtained by slow diffusion of **3a** in $CHCl_3$/DCM/Hexanes.

CCDC number	2465195
Empirical formula	$C_{25}H_{24}N_2O_2$
Formula weight	384.46
Temperature [K]	150.00(10)
Crystal system	triclinic
Space group (number)	$P\bar{1}$ (2)
a [Å]	4.9409(2)
b [Å]	13.2499(4)
c [Å]	14.7976(3)
α [°]	86.392(2)
β [°]	80.733(2)
γ [°]	85.329(3)
Volume [Å^3]	951.70(5)
Z	2
ρ_{calc} [gcm^{-3}]	1.342
μ [mm^{-1}]	0.676
F(000)	408
Crystal size [mm^3]	0.124×0.067×0.03
Crystal colour	clear light colourless
Crystal shape	irregular
Radiation	Cu K_{α} (λ=1.54184 Å)
2θ range [°]	9.27 to 160.69 (0.78 Å)
Index ranges	−6 ≤ h ≤ 6

	$-16 \leq k \leq 16$
	$-15 \leq l \leq 18$
Reflections collected	19870
Independent reflections	4069<br>$R_{int} = 0.0407$<br>$R_{sigma} = 0.0296$
Completeness to θ = 67.684°	99.9 %
Data / Restraints / Parameters	4069/0/264
Goodness-of-fit on F^2	1.080
Final R indexes [$I \geq 2\sigma(I)$]	$R_1 = 0.0449$<br>$wR_2 = 0.1168$
Final R indexes [all data]	$R_1 = 0.0489$<br>$wR_2 = 0.1214$
Largest peak/hole [eÅ^{-3}]	0.22/-0.28

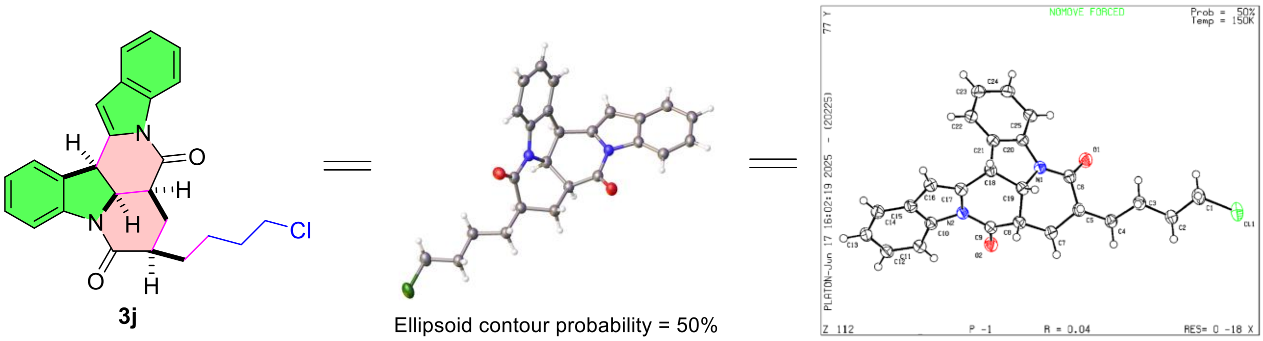

3j

Ellipsoid contour probability = 50%

X-ray crystals were obtained by slow diffusion of **3j** in DCM/Hexanes.

CCDC number	2465294
Empirical formula	$C_{25}H_{23}ClN_2O_2$
Formula weight	418.90
Temperature [K]	150.00(10)
Crystal system	triclinic
Space group (number)	$P\bar{1}$ (2)
a [Å]	8.3590(2)
b [Å]	9.6705(2)
c [Å]	13.3536(2)
α [°]	88.2122(16)
β [°]	73.347(2)
γ [°]	72.327(2)
Volume [Å^3]	983.52(4)
Z	2
ρ_{calc} [gcm^{-3}]	1.415

μ [mm^{-1}]	1.923
F(000)	440
Crystal size [mm^3]	0.336×0.23×0.152
Crystal colour	clear light colourless
Crystal shape	irregular
Radiation	Cu K_α (λ=1.54184 Å)
2θ range [°]	6.92 to 160.73 (0.78 Å)
Index ranges	−10 ≤ h ≤ 10<br>−12 ≤ k ≤ 10<br>−17 ≤ l ≤ 16
Reflections collected	26868
Independent reflections	4206<br>R_{int} = 0.0565<br>R_{sigma} = 0.0262
Completeness to θ = 67.684°	99.9 %
Data / Restraints / Parameters	4206/0/272
Goodness-of-fit on F^2	1.155
Final R indexes [I≥2σ(I)]	R_1 = 0.0443<br>wR_2 = 0.1284
Final R indexes [all data]	R_1 = 0.0464<br>wR_2 = 0.1305
Largest peak/hole [eÅ^{-3}]	0.27/-0.47
Extinction coefficient	0.0038(7)

16. NMR spectra charts for compounds 1-3 and 5-7

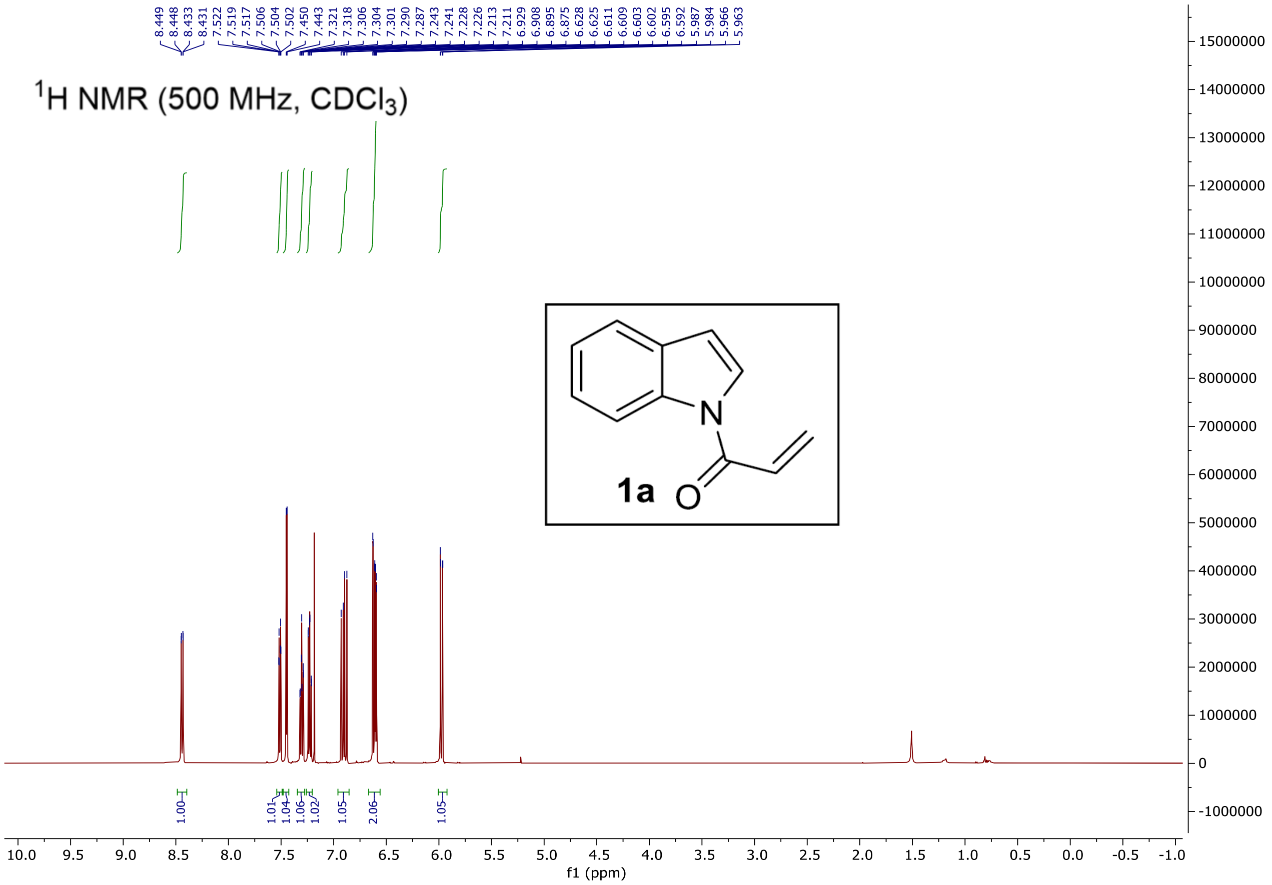

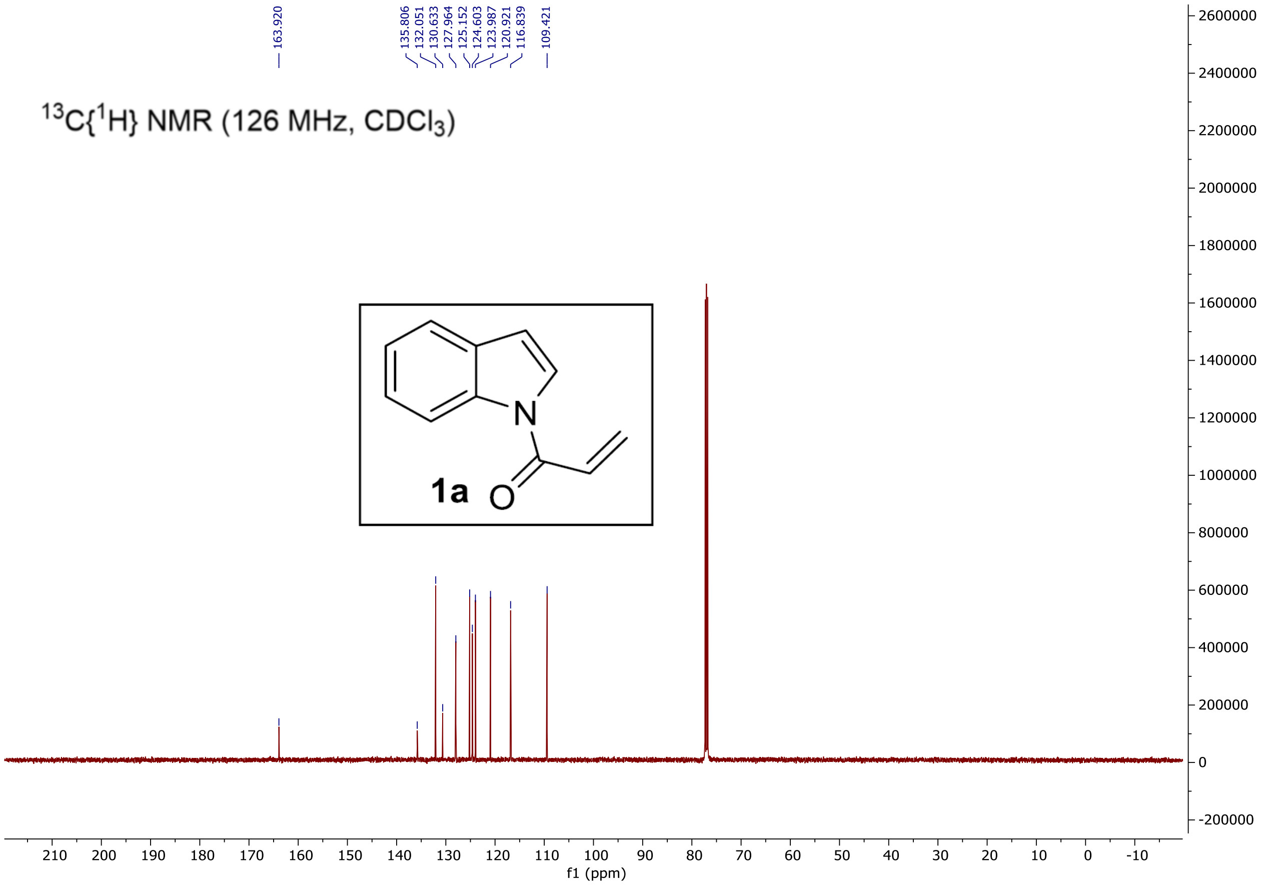

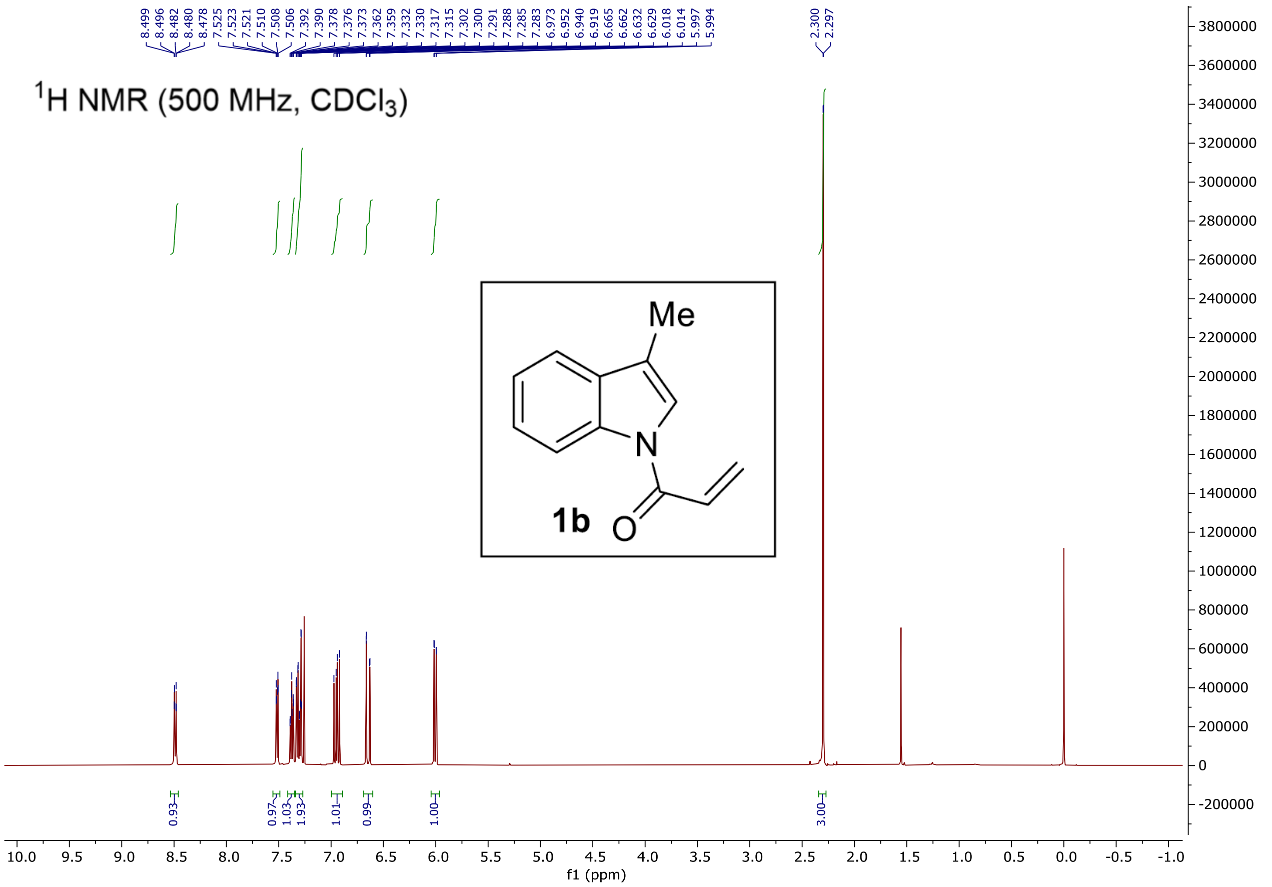

8.499 8.496 8.482 8.480 8.478 7.525 7.523 7.521 7.510 7.508 7.506 7.392 7.390 7.378 7.376 7.373 7.362 7.359 7.332 7.330 7.317 7.315 7.302 7.300 7.291 7.288 7.285 7.283 6.973 6.952 6.940 6.919 6.665 6.662 6.632 6.629 6.018 6.014 5.997 5.994
2.300 2.297
1H NMR (500 MHz, CDCl3)
Me
N
O
1b
0.93 0.97 1.03 1.93 1.01 0.99 1.00 3.00
f1 (ppm)

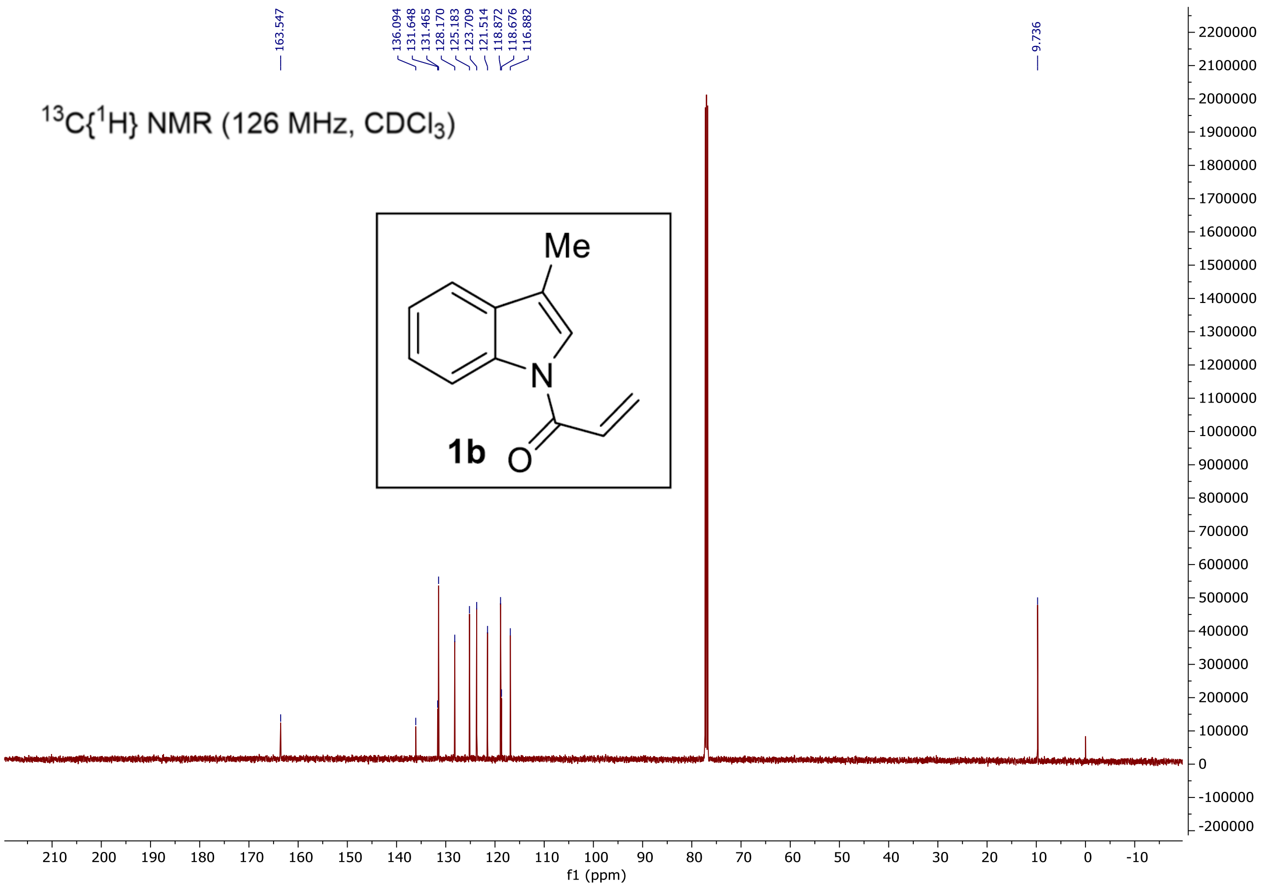

163.547
136.094 131.648 131.465 128.170 125.183 123.709 121.514 118.872 118.676 116.882
9.736
13C{1H} NMR (126 MHz, CDCl3)
Me
N
O
1b
f1 (ppm)

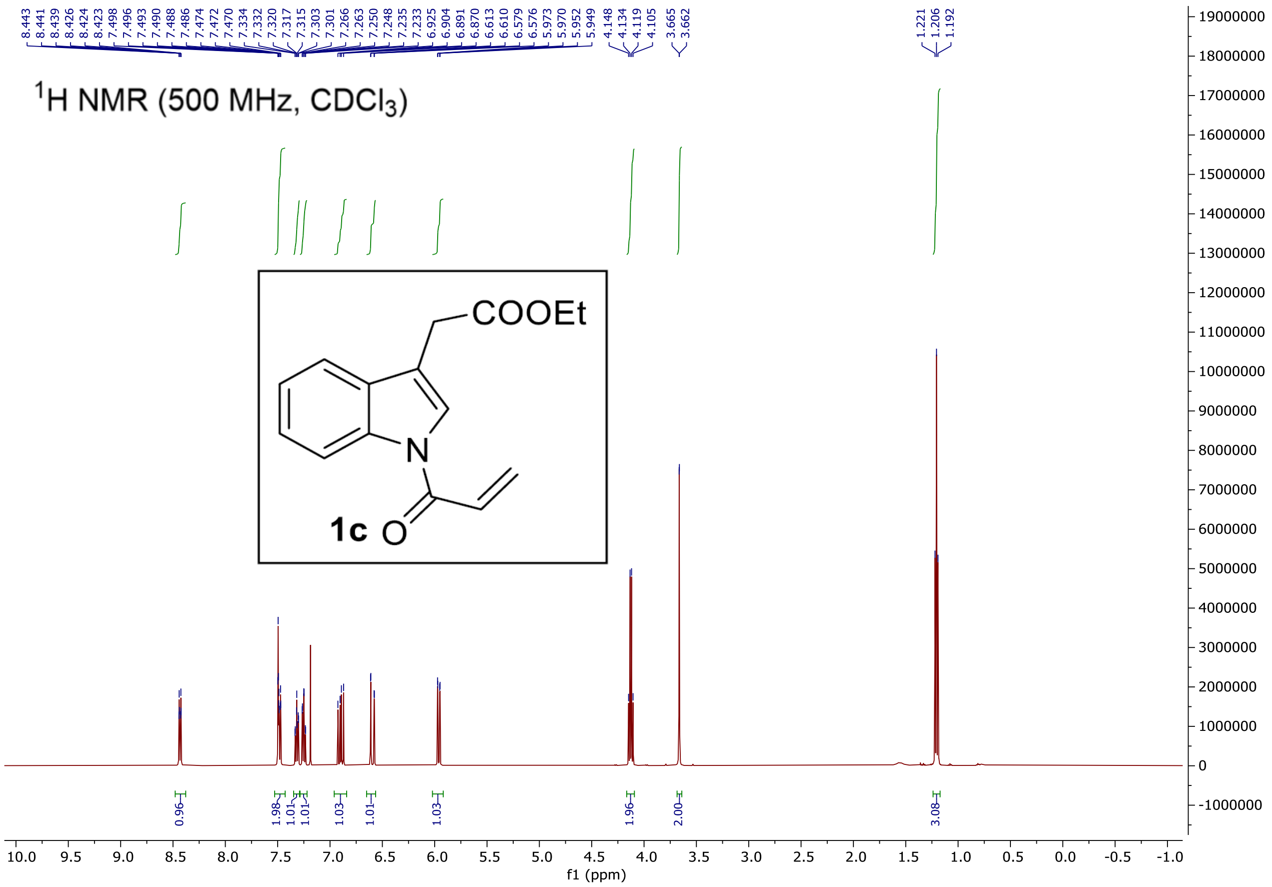

1H NMR (500 MHz, CDCl3)
1c
COOEt
N
O
f1 (ppm)

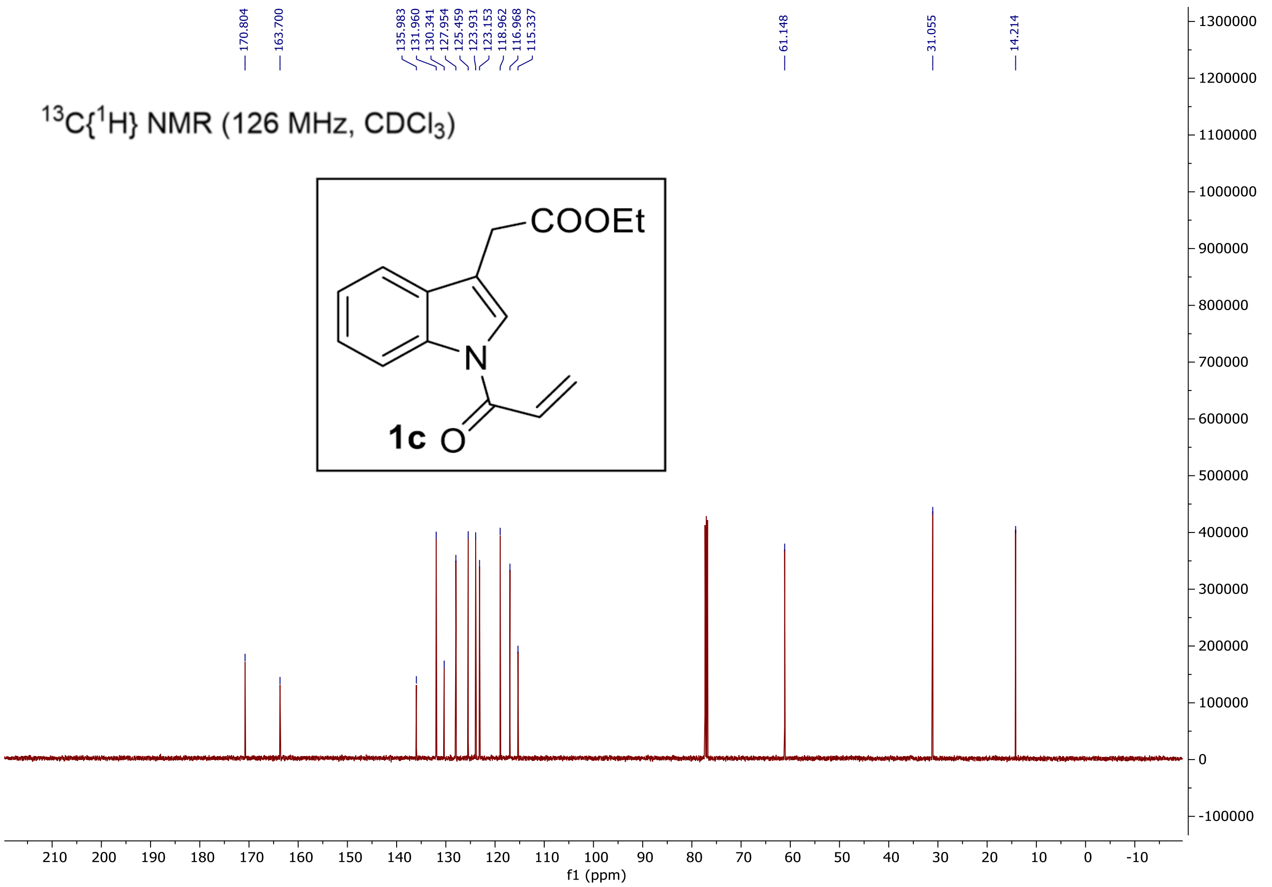

13C{1H} NMR (126 MHz, CDCl3)
1c
COOEt
N
O
f1 (ppm)

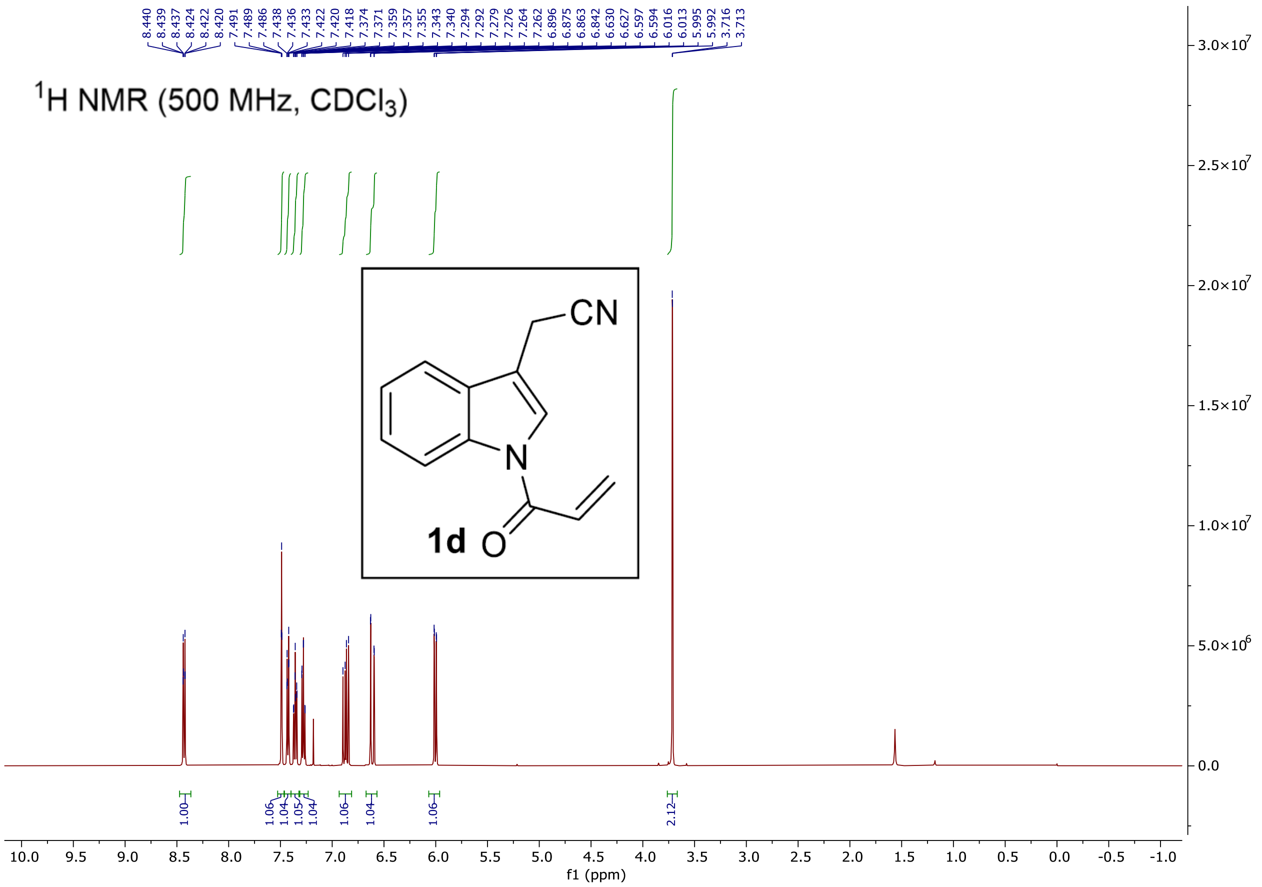

^{1}H NMR (500 MHz, CDCl$_3$)
1d
CN
N
O
f1 (ppm)

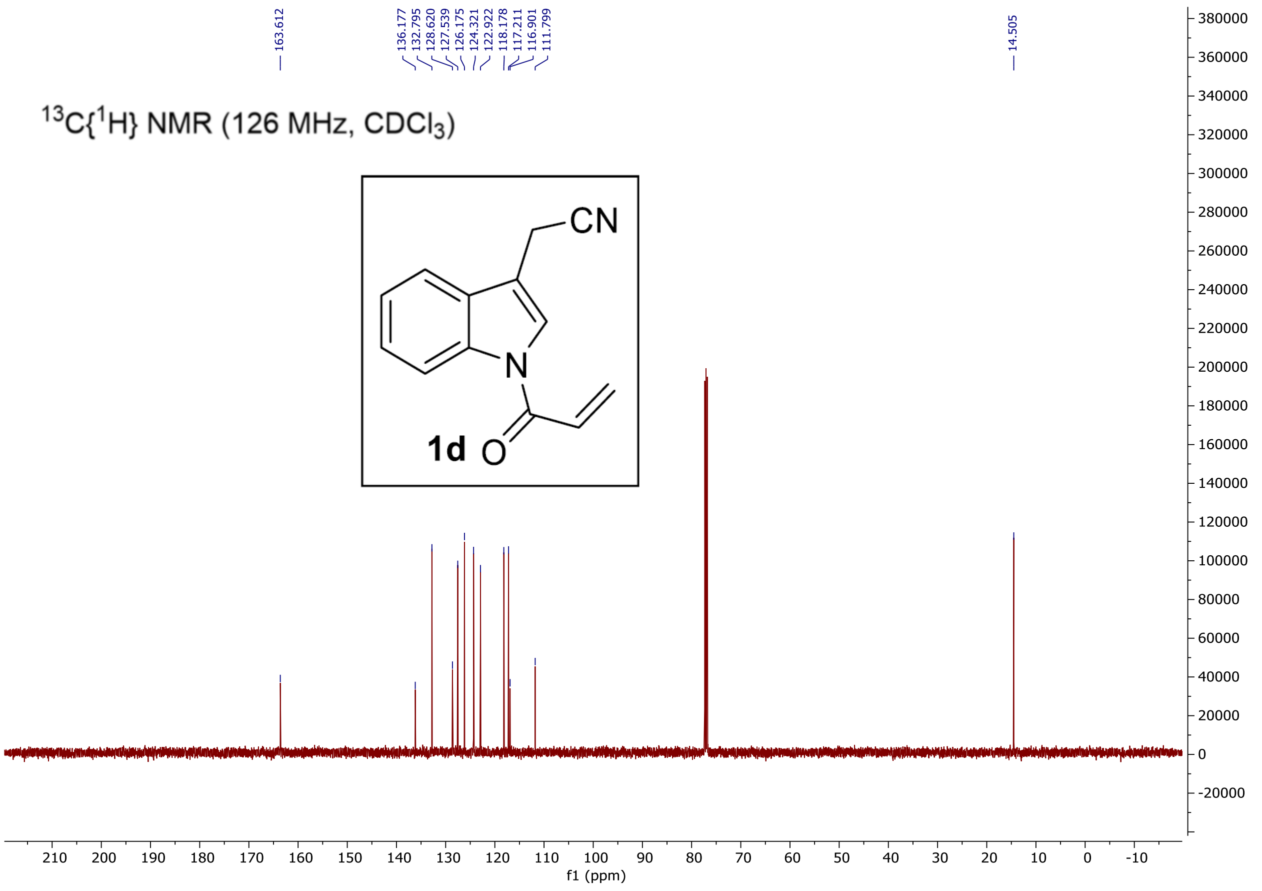

^{13}C{^{1}H} NMR (126 MHz, CDCl$_3$)
1d
CN
N
O
f1 (ppm)

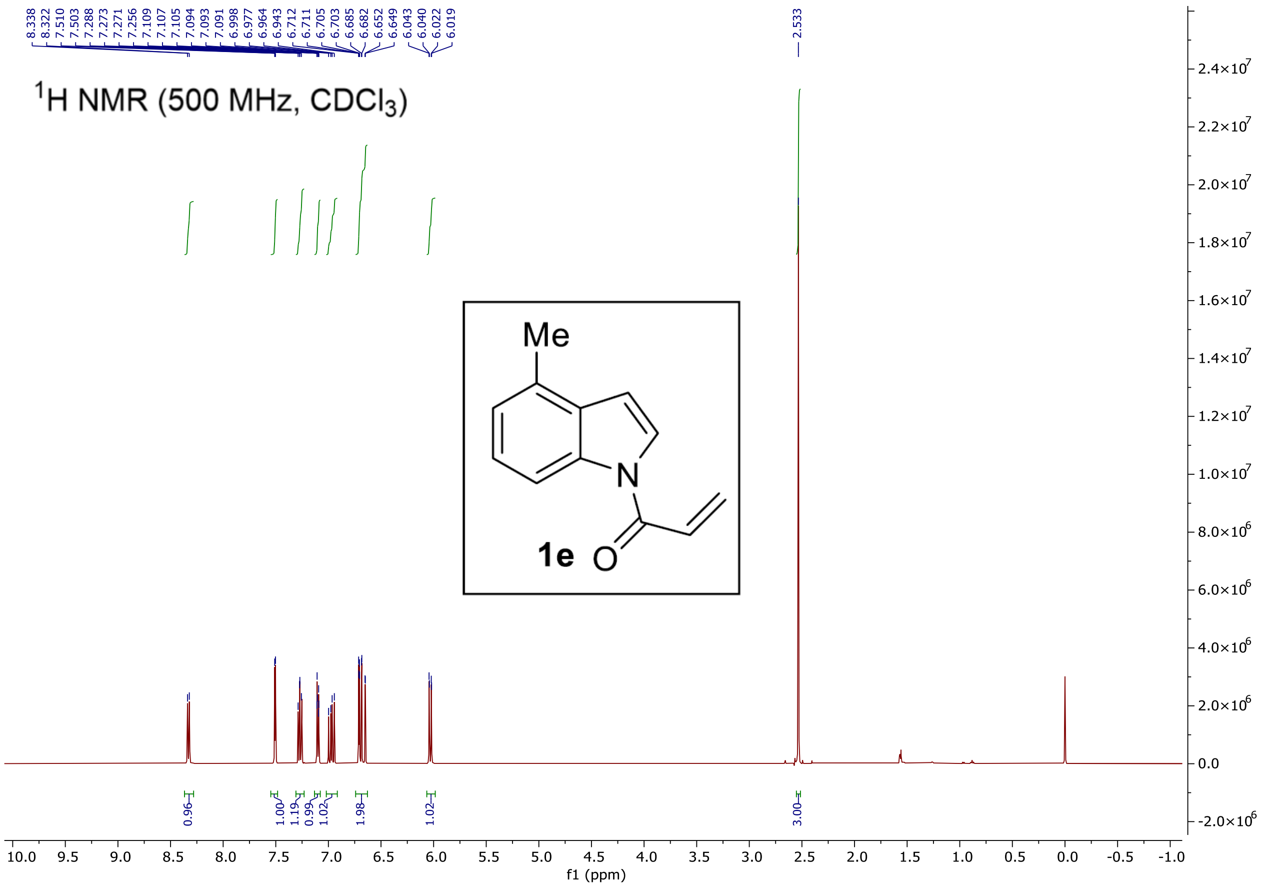

8.338
8.322
7.510
7.503
7.288
7.273
7.271
7.256
7.109
7.107
7.105
7.094
7.093
7.091
6.998
6.977
6.964
6.943
6.712
6.711
6.705
6.703
6.685
6.682
6.652
6.649
6.043
6.040
6.022
6.019
2.533
^{1}H NMR (500 MHz, CDCl$_3$)
Me
N
O
1e
0.96
1.00
1.19
0.99
1.02
1.98
1.02
3.00
f1 (ppm)

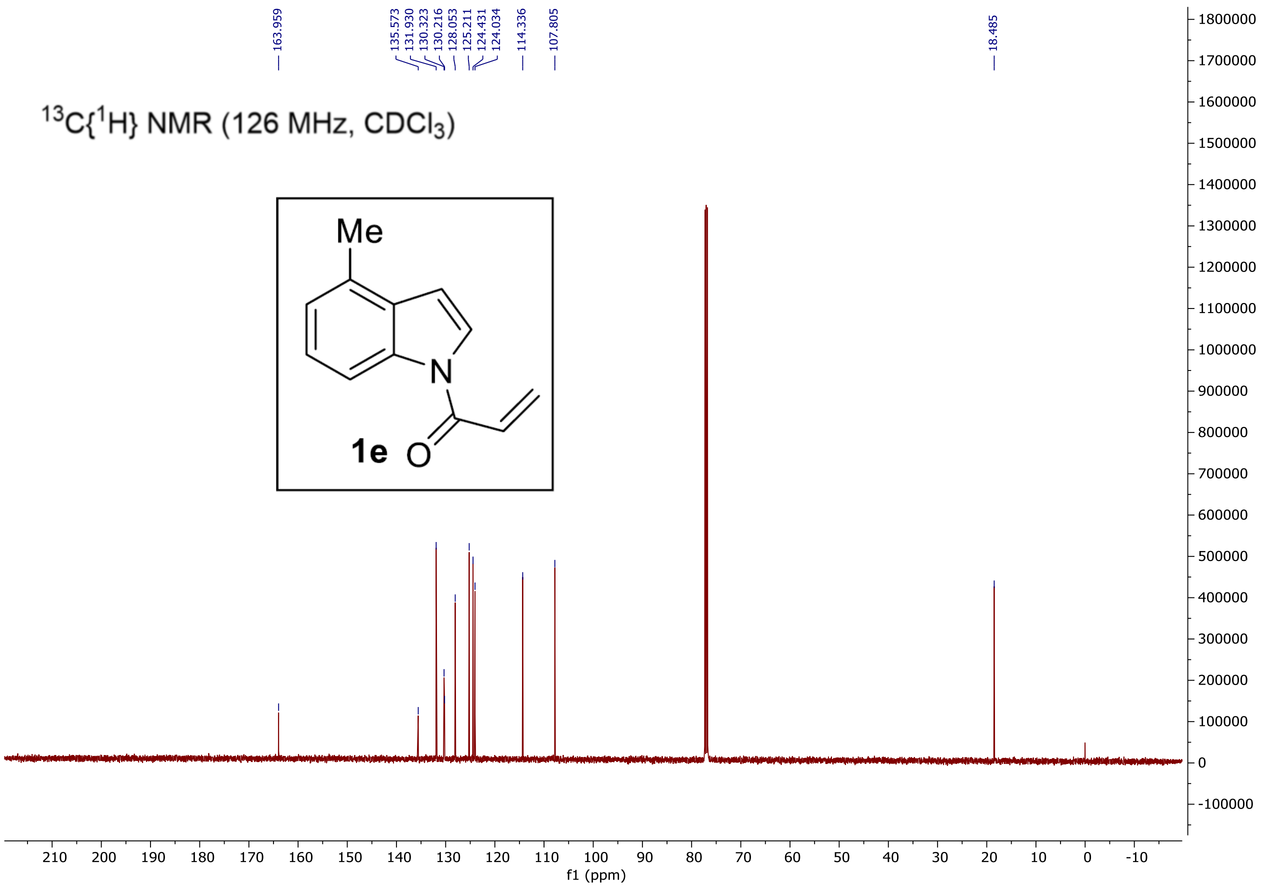

163.959
135.573
131.930
130.323
130.216
128.053
125.211
124.431
124.034
114.336
107.805
18.485
^{13}C{^{1}H} NMR (126 MHz, CDCl$_3$)
Me
N
O
1e
f1 (ppm)

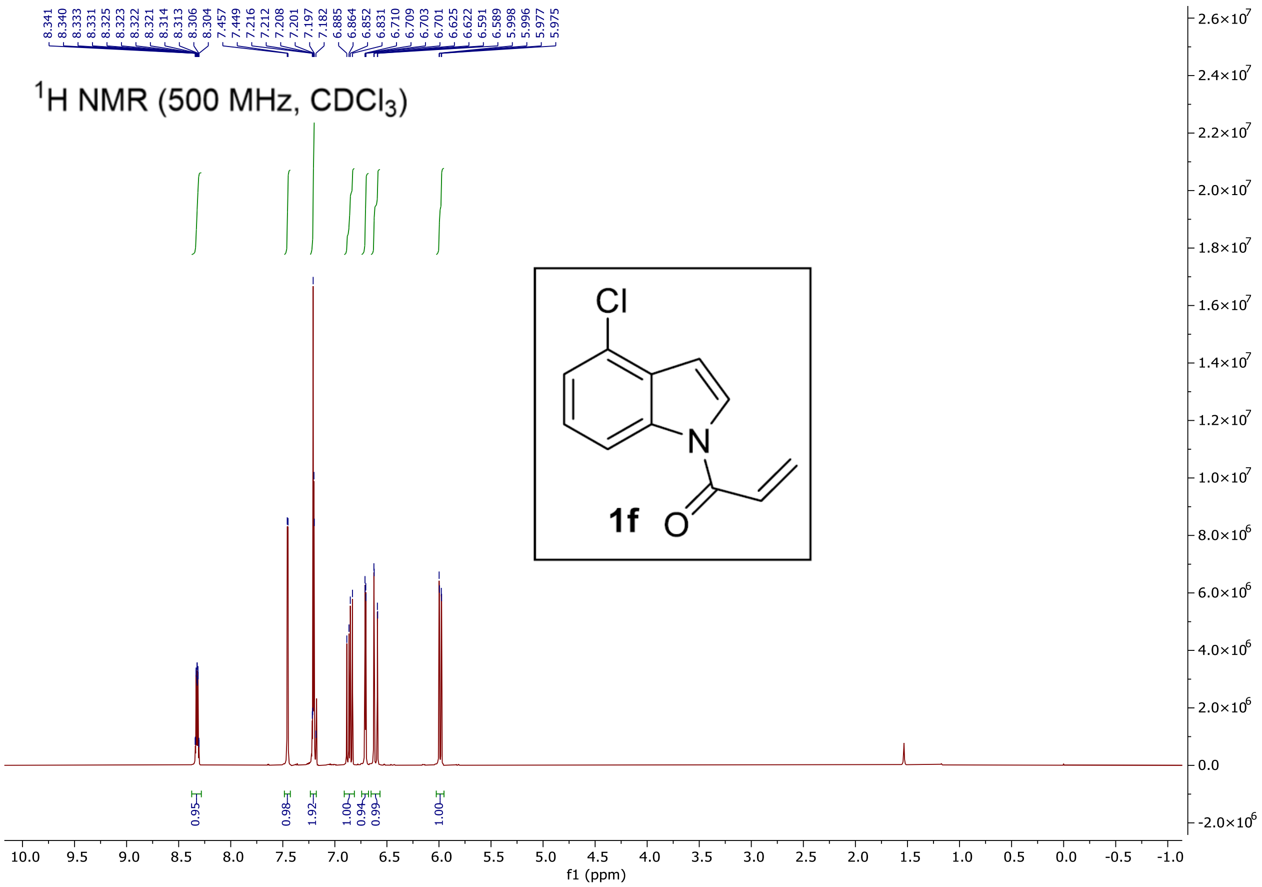

^{1}H NMR (500 MHz, CDCl$_3$)
Cl
N
O
1f
f1 (ppm)

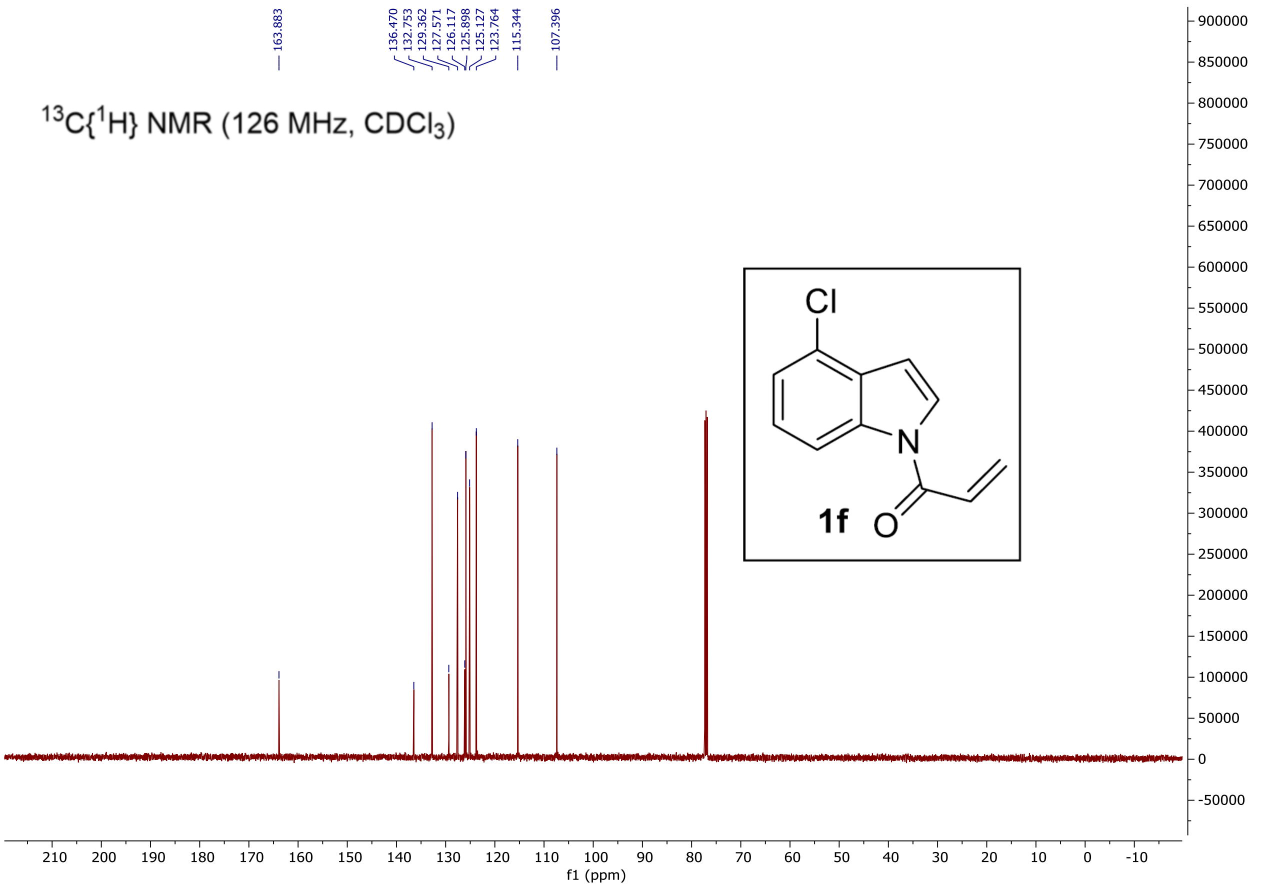

^{13}C{^{1}H} NMR (126 MHz, CDCl$_3$)
163.883
136.470
132.753
129.362
127.571
126.117
125.898
125.127
123.764
115.344
107.396
Cl
N
O
1f
f1 (ppm)

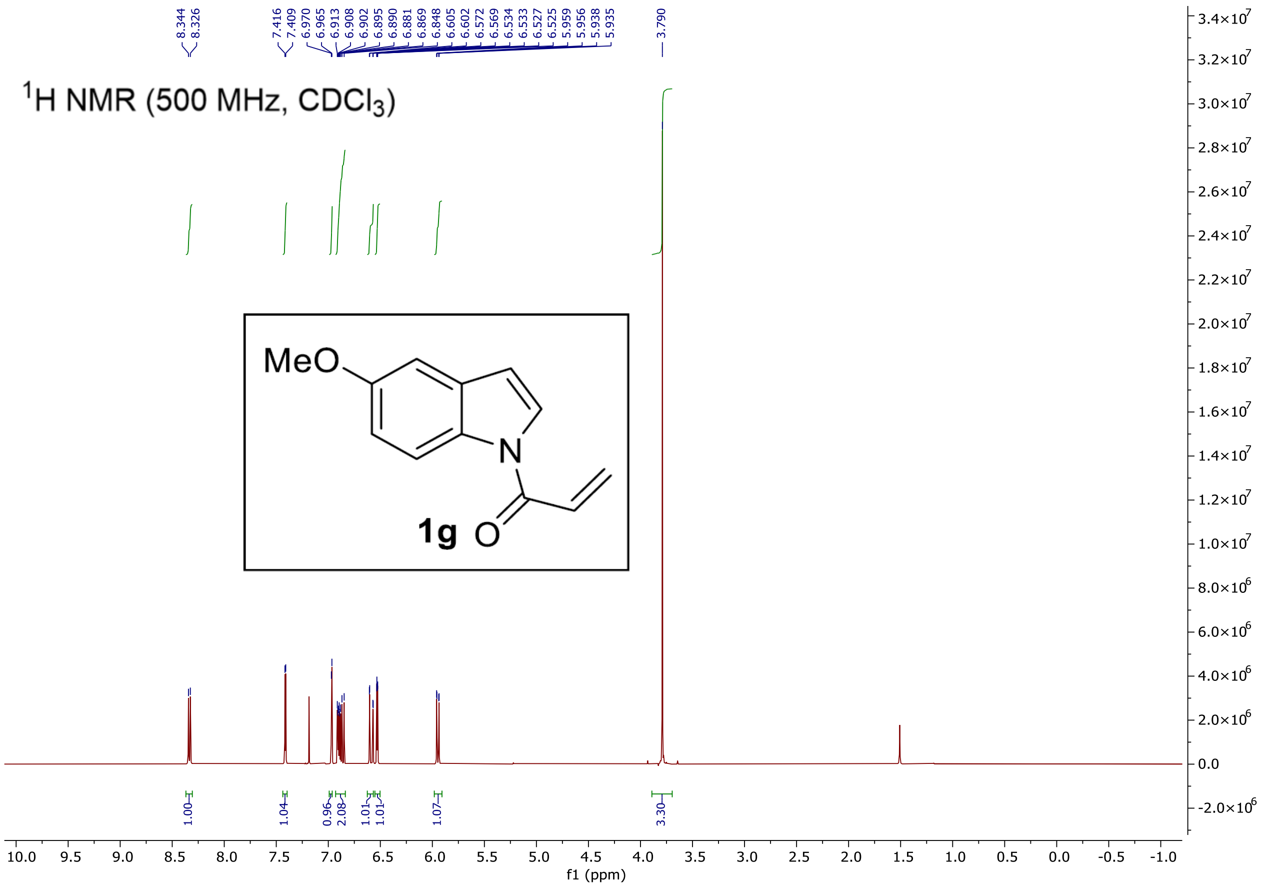

8.344
8.326
7.416
7.409
6.970
6.965
6.913
6.908
6.902
6.895
6.890
6.881
6.869
6.848
6.605
6.602
6.572
6.569
6.534
6.533
6.527
6.525
5.959
5.956
5.938
5.935
3.790
^{1}H NMR (500 MHz, $CDCl_3$)
MeO
N
O
1g
1.00
1.04
0.96
2.08
1.01
1.01
1.07
3.30
f1 (ppm)

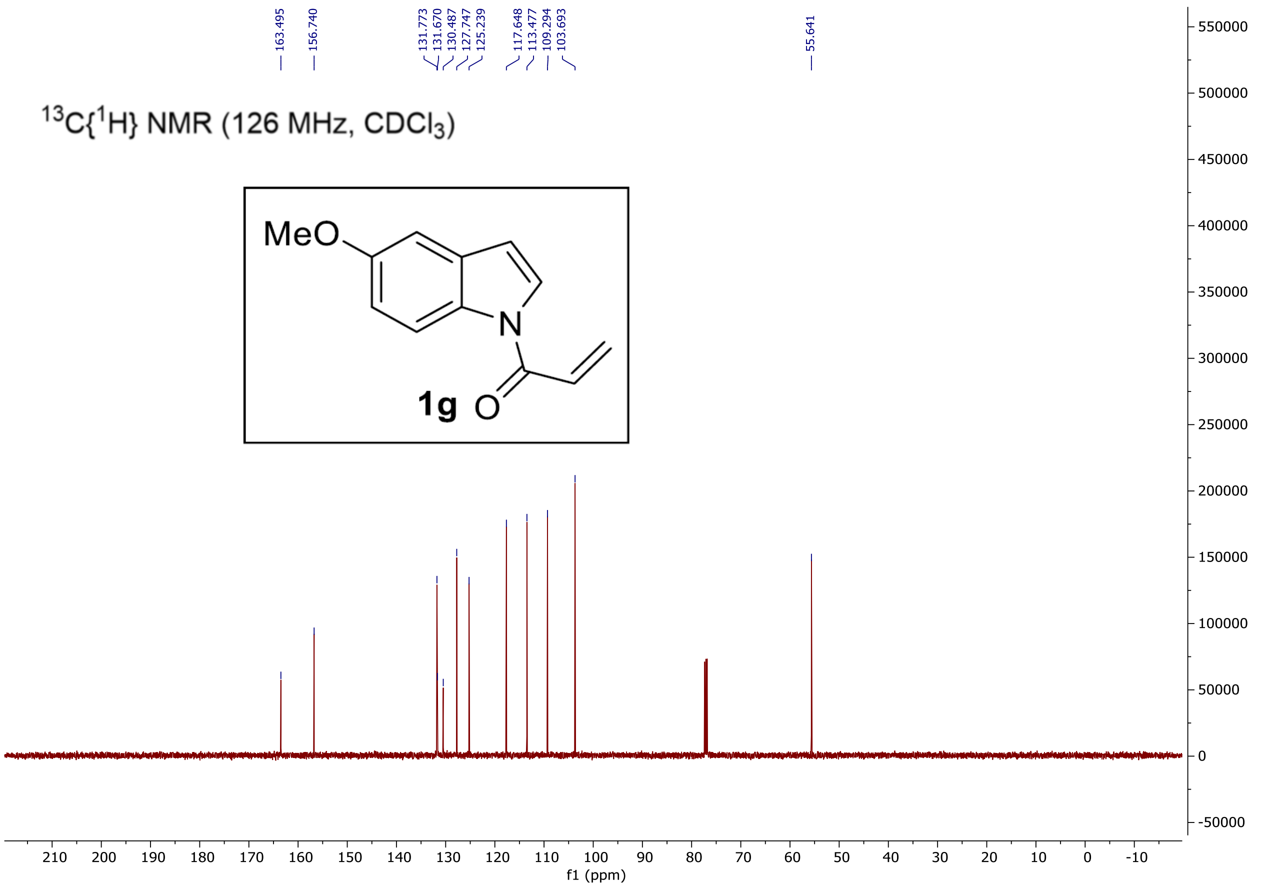

163.495
156.740
131.773
131.670
130.487
127.747
125.239
117.648
113.477
109.294
103.693
55.641
^{13}C{^{1}H} NMR (126 MHz, $CDCl_3$)
MeO
N
O
1g
f1 (ppm)

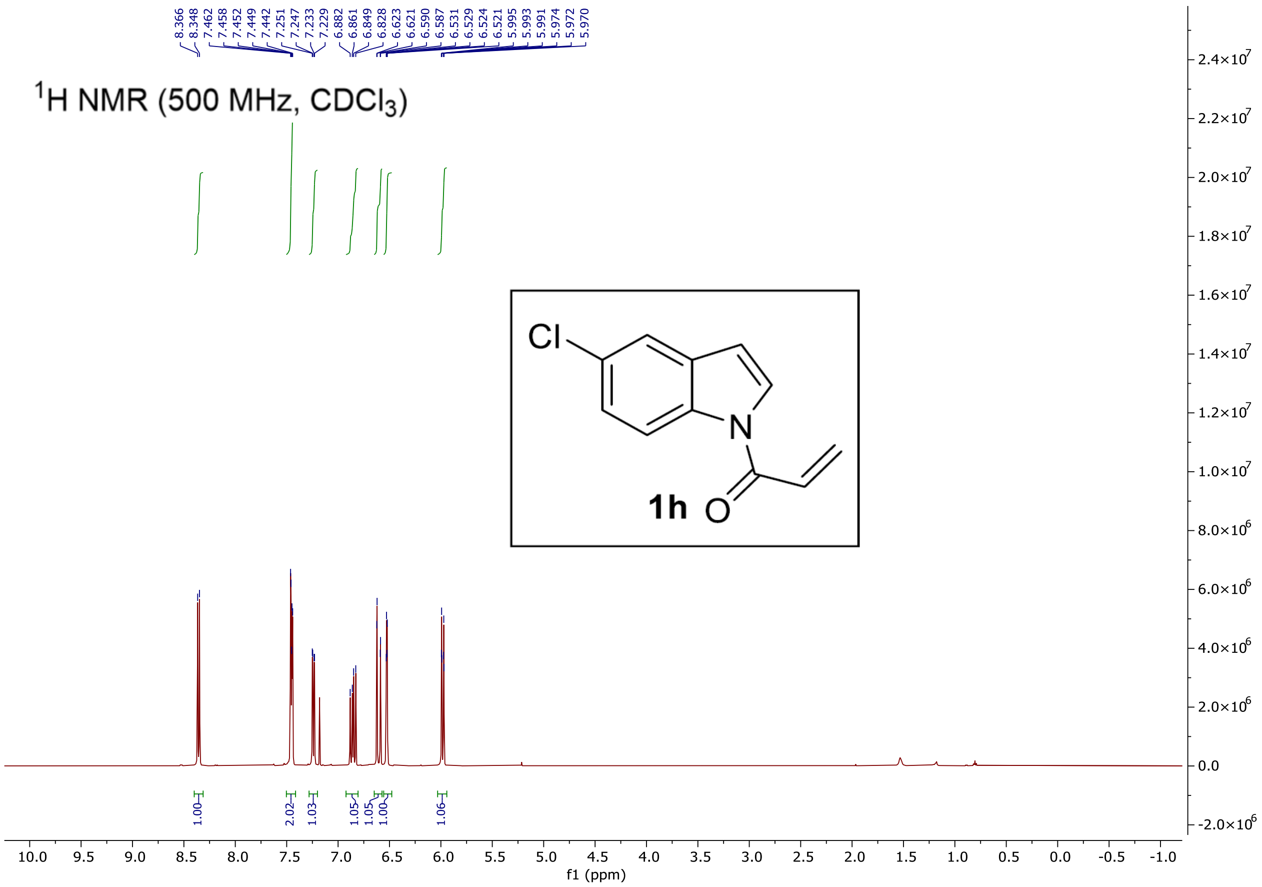

8.366
8.348
7.462
7.458
7.452
7.449
7.442
7.251
7.247
7.233
7.229
6.882
6.861
6.849
6.828
6.623
6.621
6.590
6.587
6.531
6.529
6.524
6.521
5.995
5.993
5.991
5.974
5.972
5.970
^{1}H NMR (500 MHz, CDCl$_3$)
Cl
N
O
1h
1.00
2.02
1.03
1.05
1.05
1.00
1.06
f1 (ppm)

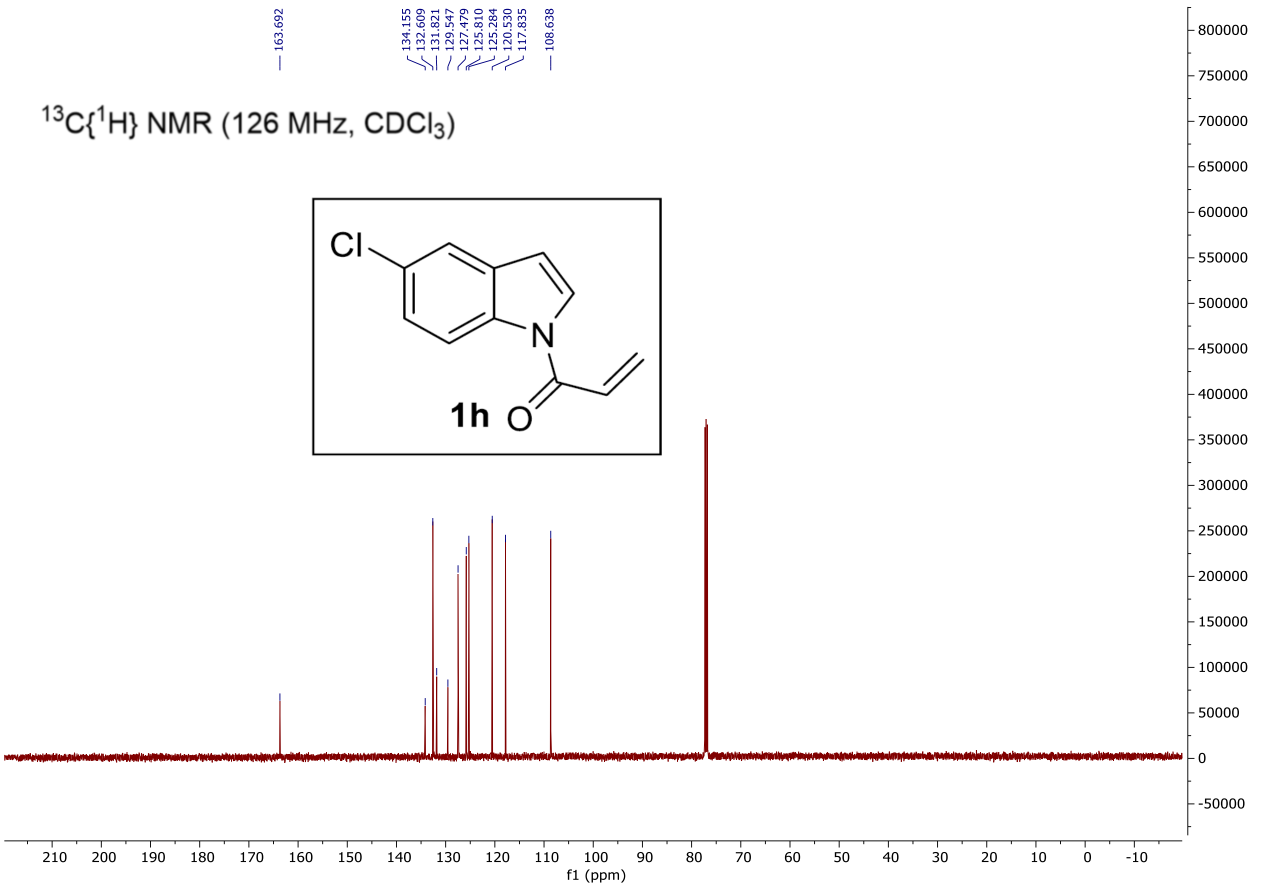

163.692
134.155
132.609
131.821
129.547
127.479
125.810
125.284
120.530
117.835
108.638
^{13}C{^{1}H} NMR (126 MHz, CDCl$_3$)
Cl
N
O
1h
f1 (ppm)

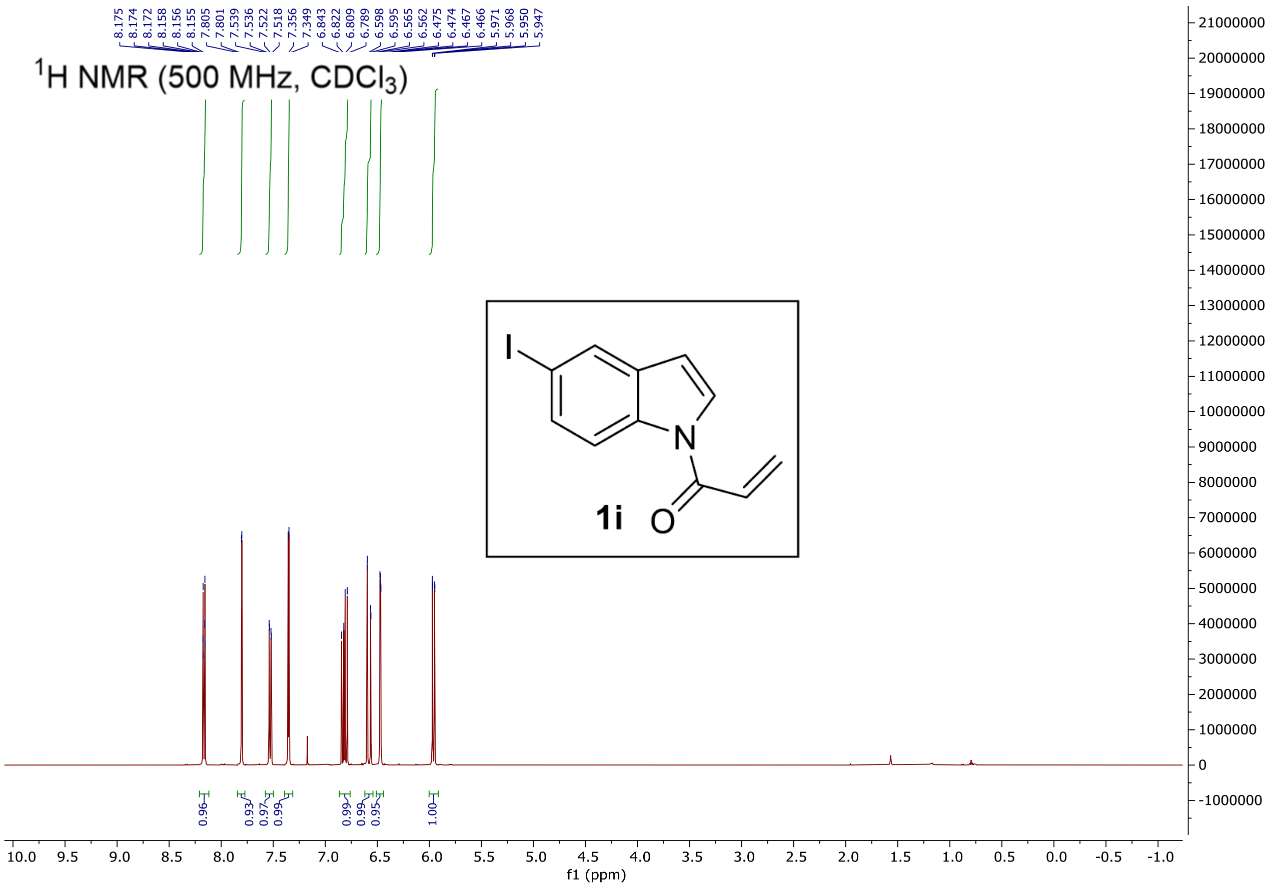
8.175
8.174
8.172
8.158
8.156
8.155
7.805
7.801
7.539
7.536
7.522
7.518
7.356
7.349
6.843
6.822
6.809
6.789
6.598
6.595
6.565
6.562
6.475
6.474
6.467
6.466
5.971
5.968
5.950
5.947
1H NMR (500 MHz, CDCl3)
I
N
O
1i
0.96
0.93
0.97
0.99
0.99
0.99
0.95
1.00
f1 (ppm)

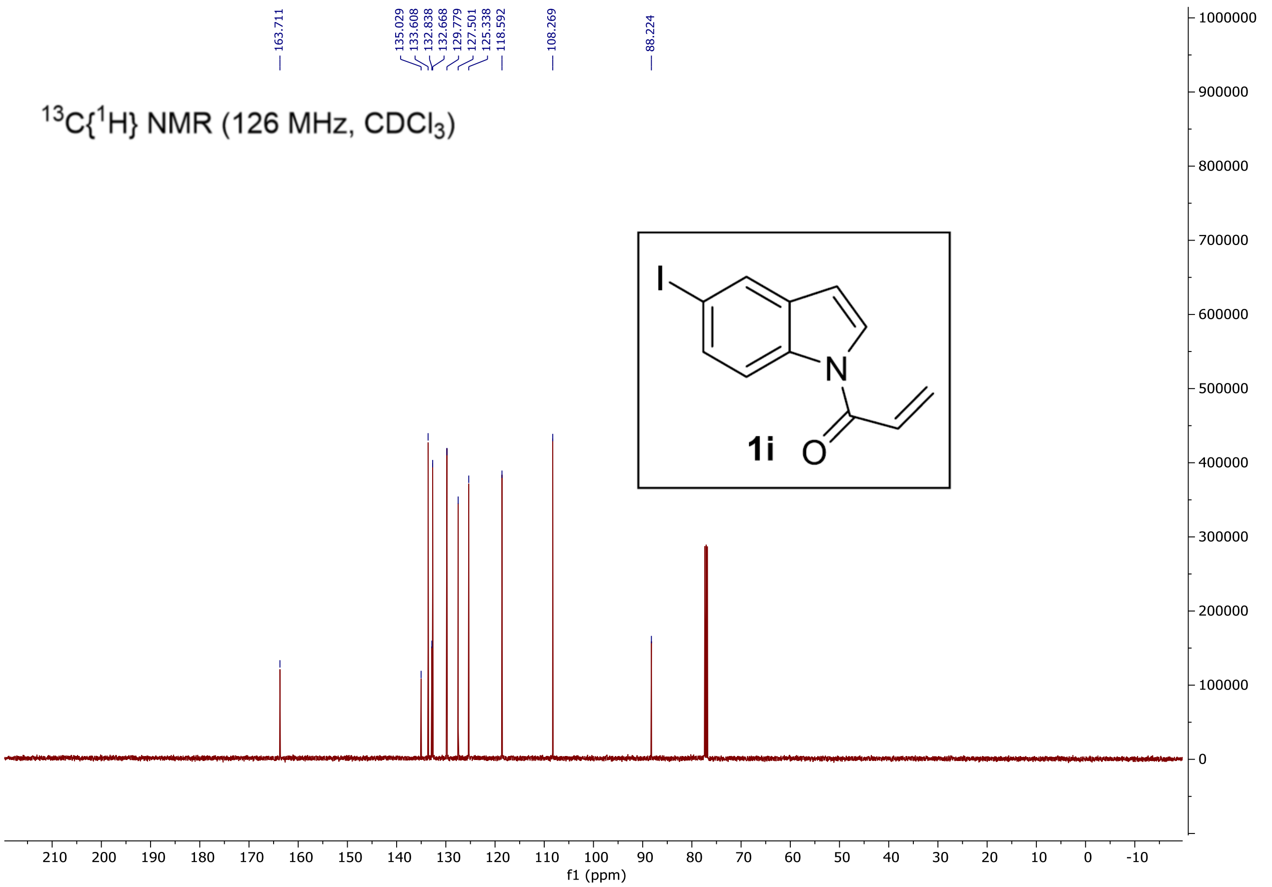
163.711
135.029
133.608
132.838
132.668
129.779
127.501
125.338
118.592
108.269
88.224
13C{1H} NMR (126 MHz, CDCl3)
I
N
O
1i
f1 (ppm)

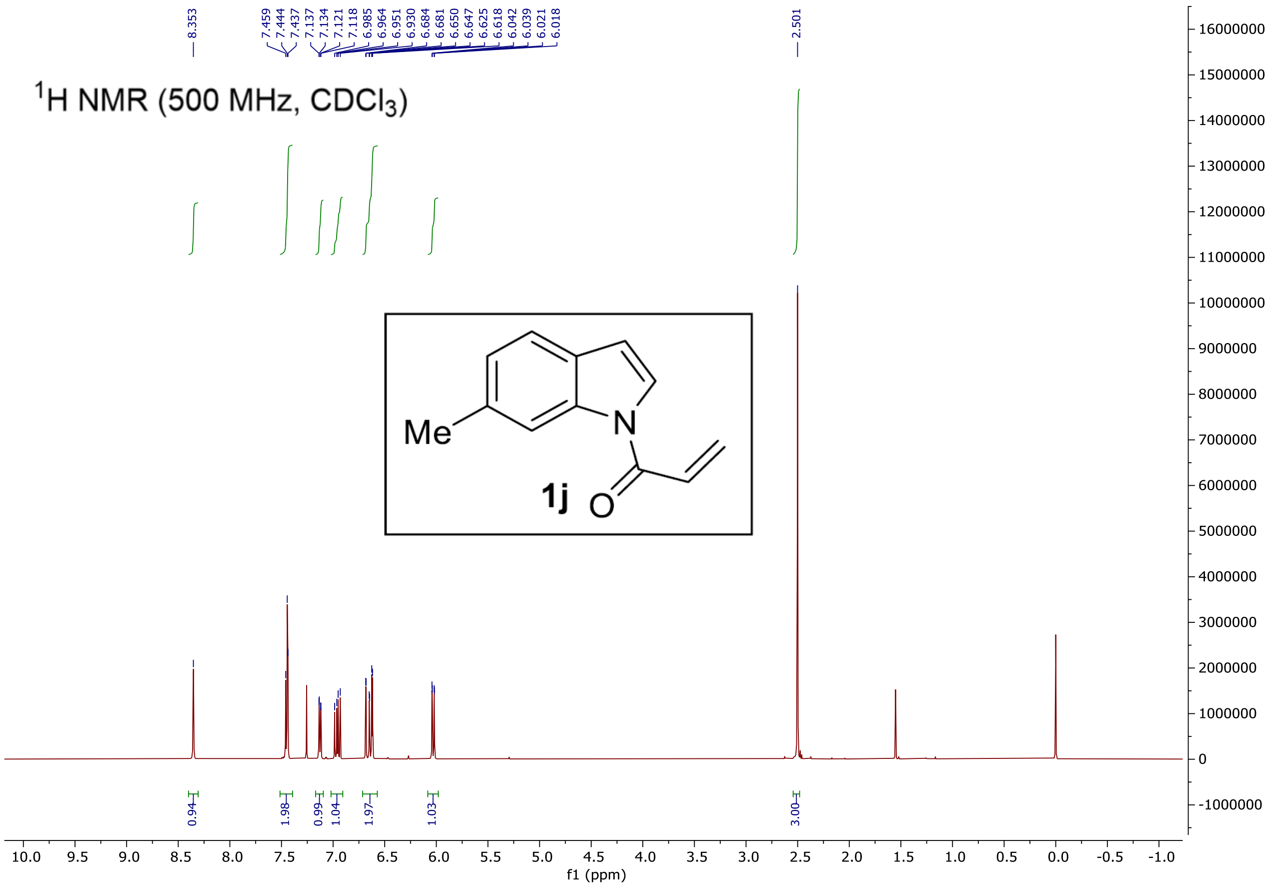

8.353
7.459
7.444
7.437
7.137
7.134
7.121
7.118
6.985
6.964
6.951
6.930
6.684
6.681
6.650
6.647
6.625
6.618
6.042
6.039
6.021
6.018
2.501
^{1}H NMR (500 MHz, $CDCl_3$)
Me
N
1j
O
0.94
1.98
0.99
1.04
1.97
1.03
3.00
f1 (ppm)

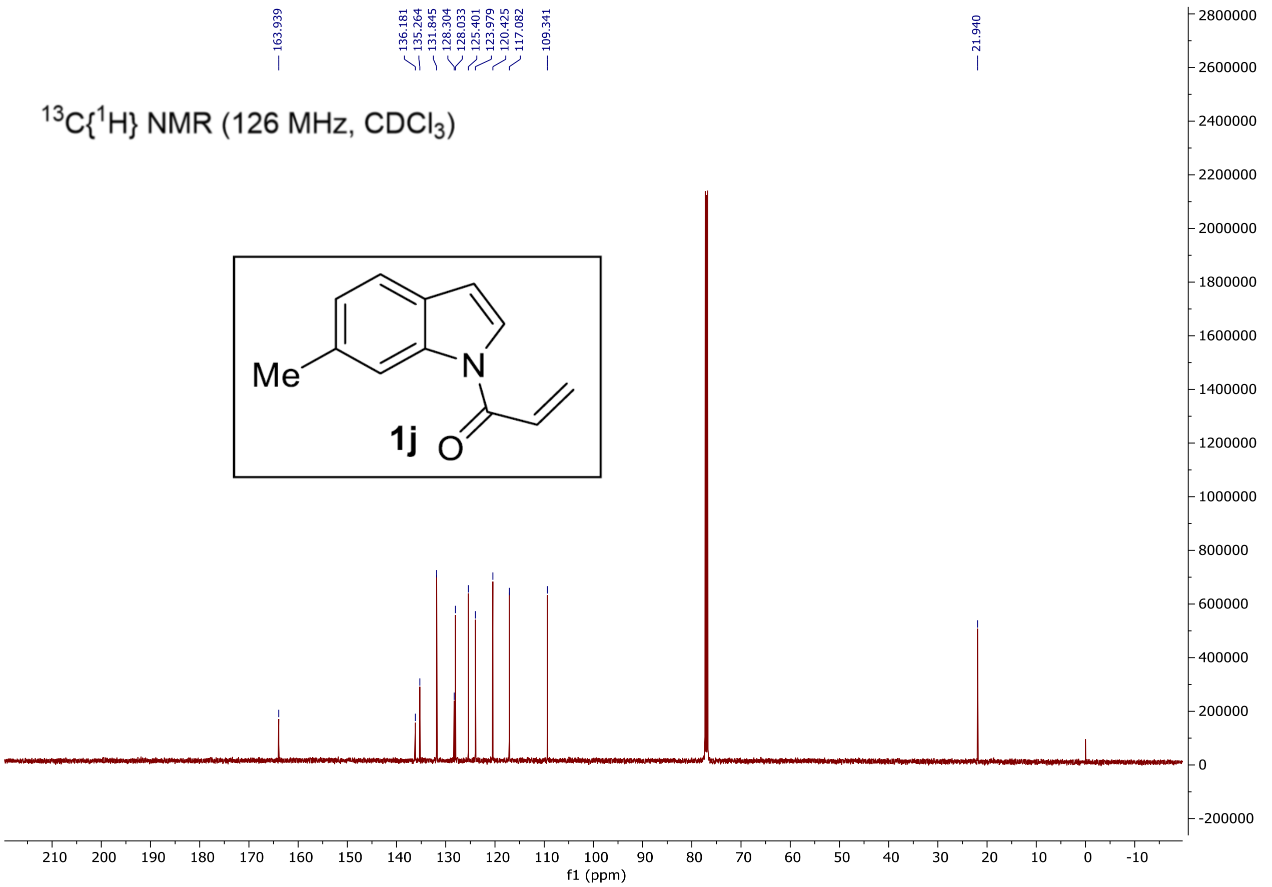

163.939
136.181
135.264
131.845
128.304
128.033
125.401
123.979
120.425
117.082
109.341
21.940
^{13}C{^{1}H} NMR (126 MHz, $CDCl_3$)
Me
N
1j
O
f1 (ppm)

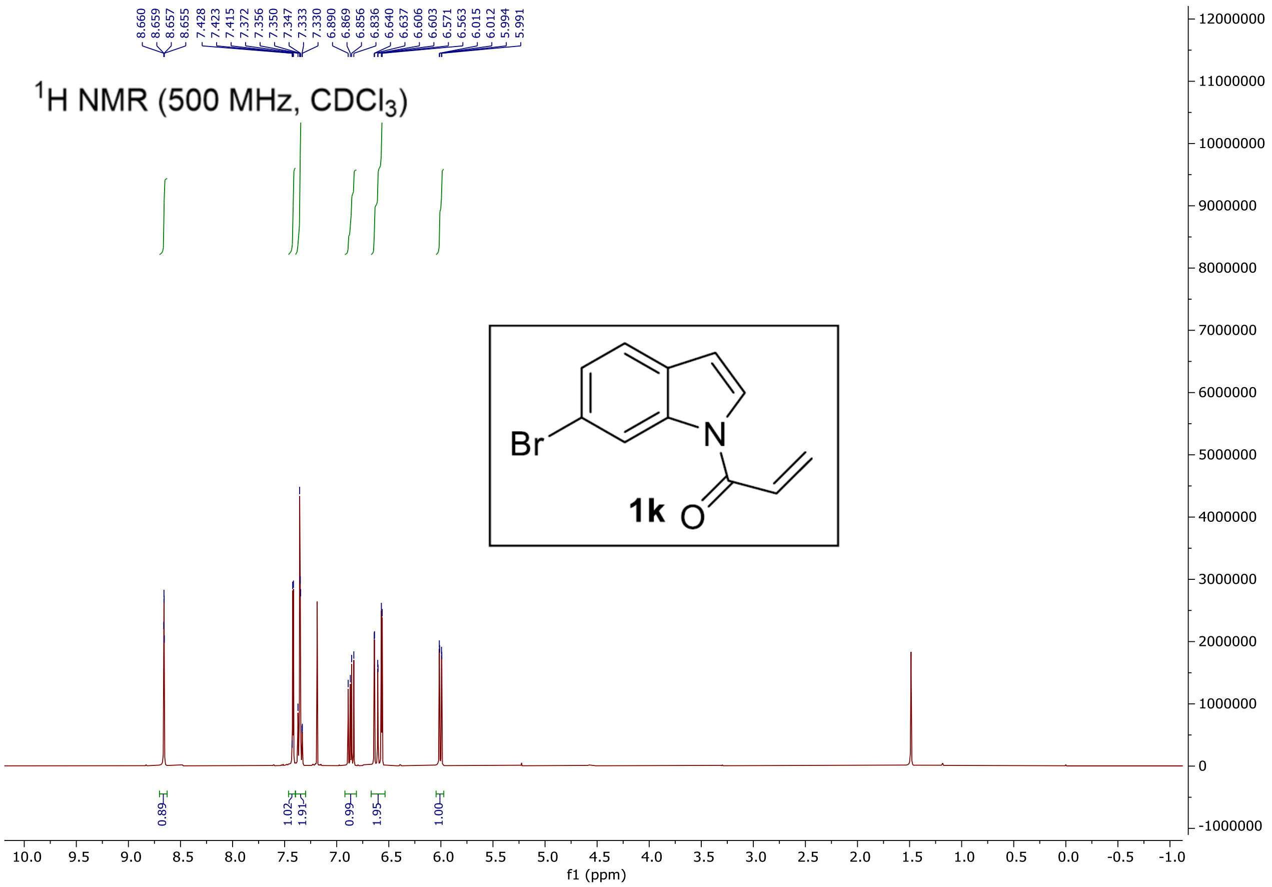
8.660
8.659
8.657
8.655
7.428
7.423
7.415
7.372
7.356
7.350
7.347
7.333
7.330
6.890
6.869
6.856
6.836
6.640
6.637
6.606
6.603
6.571
6.563
6.015
6.012
5.994
5.991
1H NMR (500 MHz, CDCl3)
Br
N
O
1k
0.89
1.02
1.91
0.99
1.95
1.00
f1 (ppm)

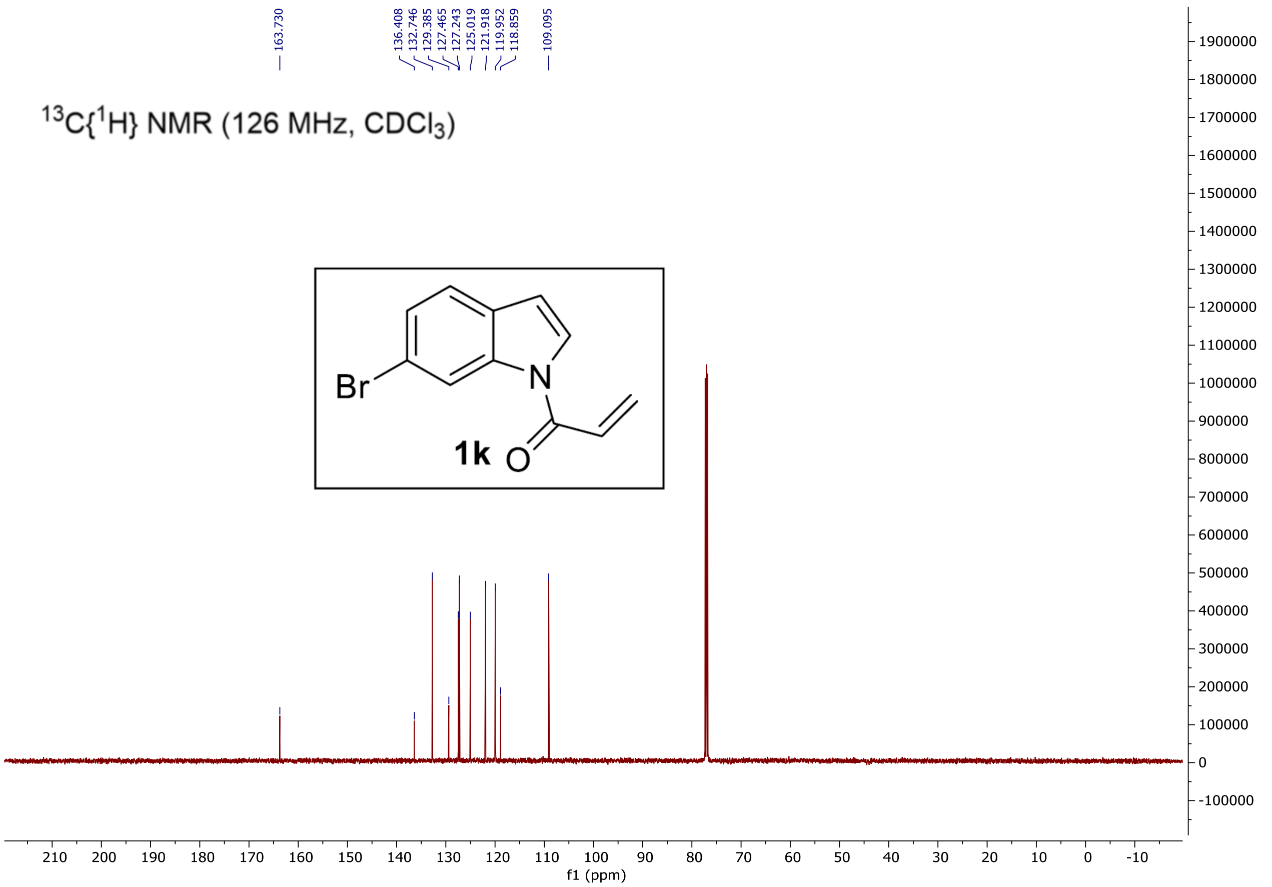
163.730
136.408
132.746
129.385
127.465
127.243
125.019
121.918
119.952
118.859
109.095
13C{1H} NMR (126 MHz, CDCl3)
Br
N
O
1k
f1 (ppm)

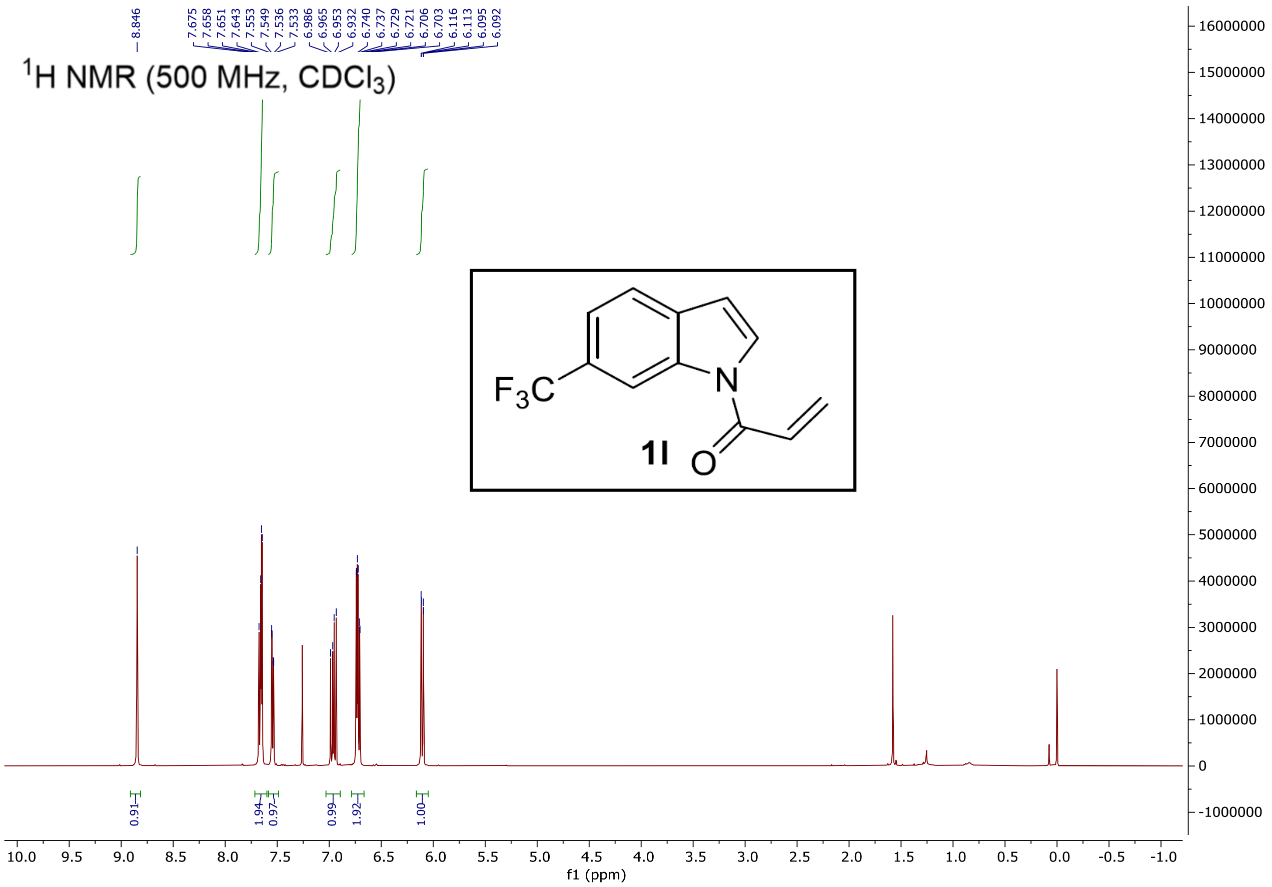
^{1}H NMR (500 MHz, CDCl$_3$)
8.846
7.675 7.658 7.651 7.643 7.553 7.549 7.536 7.533 6.986 6.965 6.953 6.932 6.740 6.737 6.729 6.721 6.706 6.703 6.116 6.113 6.095 6.092
0.91 1.94 0.97 0.99 1.92 1.00
F$_3$C
N
O
1l
f1 (ppm)

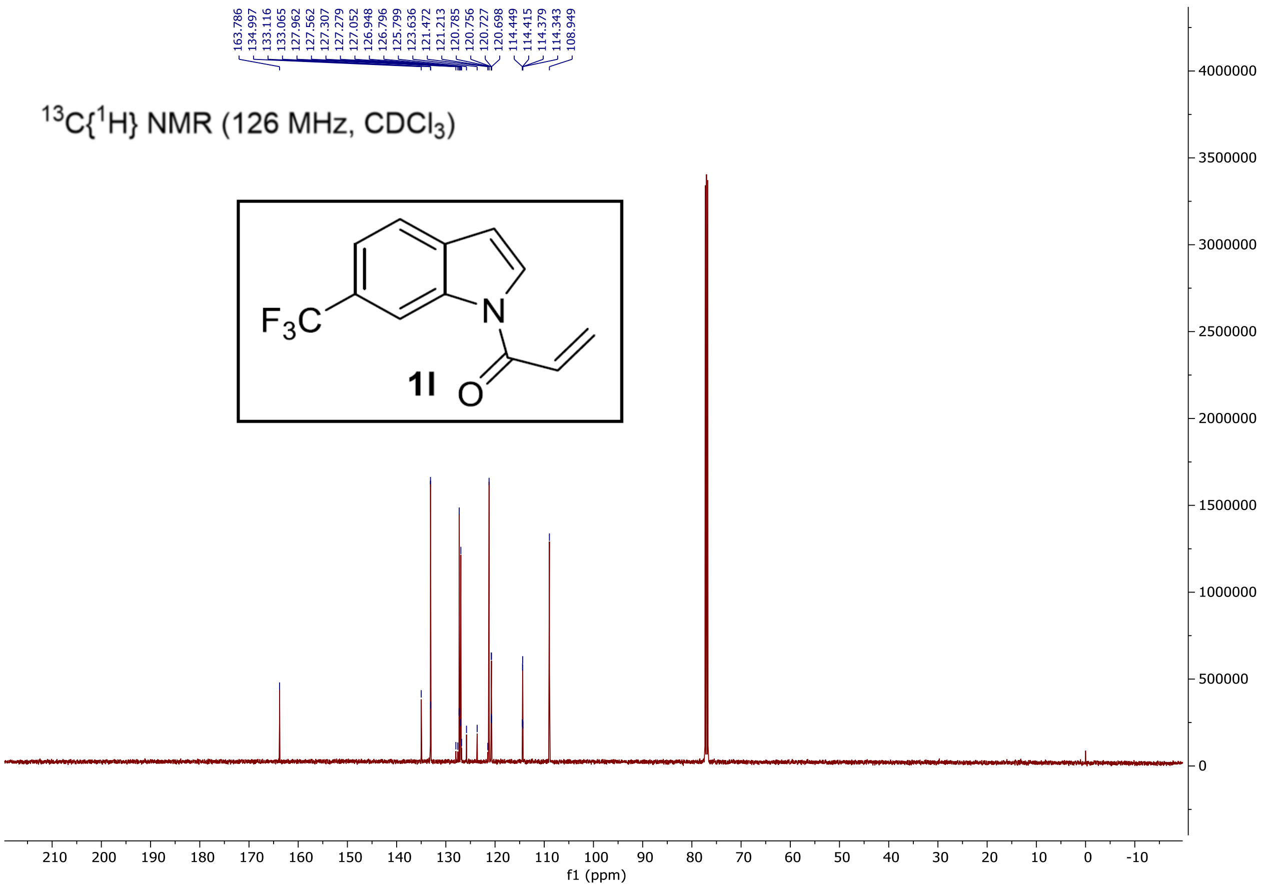
^{13}C{^{1}H} NMR (126 MHz, CDCl$_3$)
163.786 134.997 133.116 133.065 127.962 127.562 127.307 127.279 127.052 126.948 126.796 125.799 123.636 121.472 121.213 120.785 120.756 120.727 120.698 114.449 114.415 114.379 114.343 108.949
F$_3$C
N
O
1l
f1 (ppm)

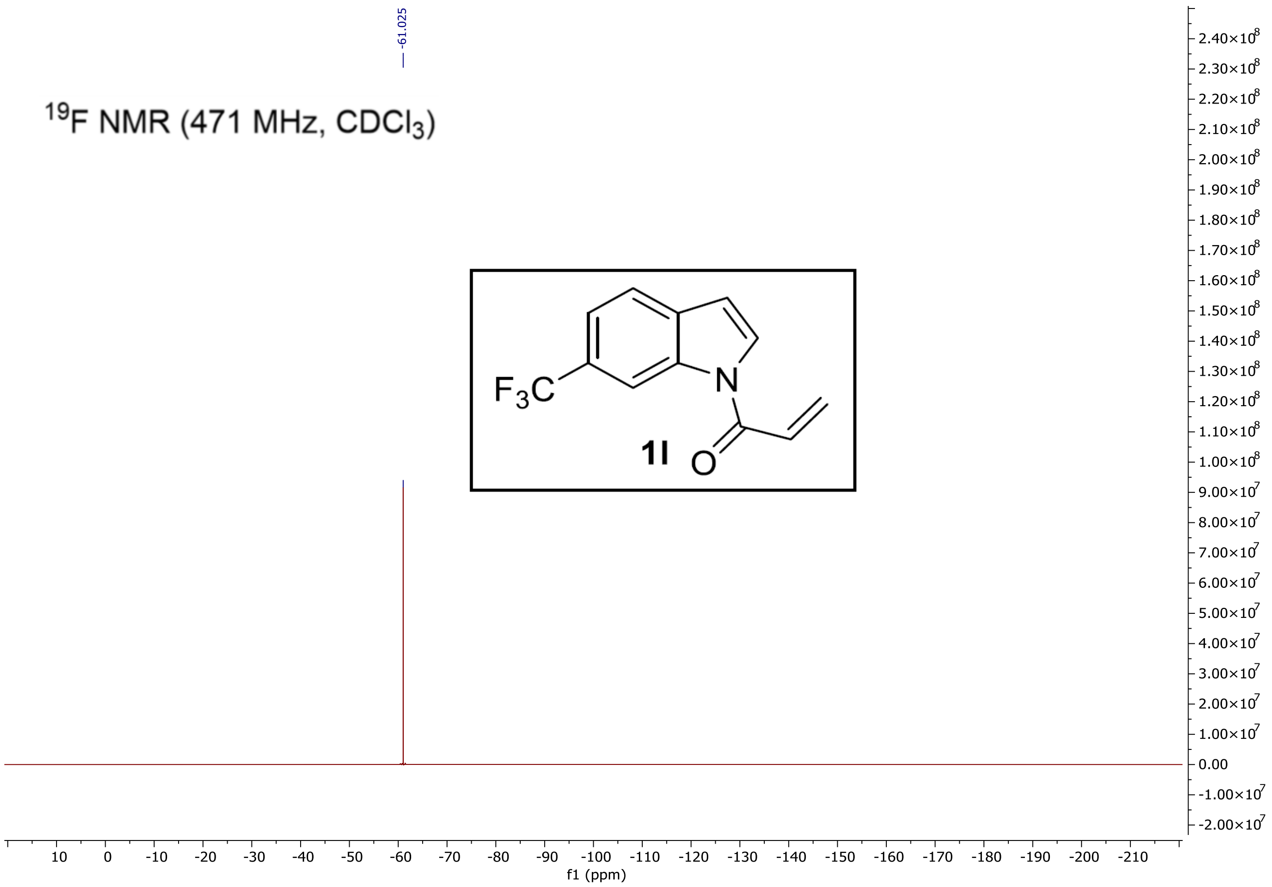
-61.025
19F NMR (471 MHz, CDCl3)
F3C
N
O
1l
f1 (ppm)

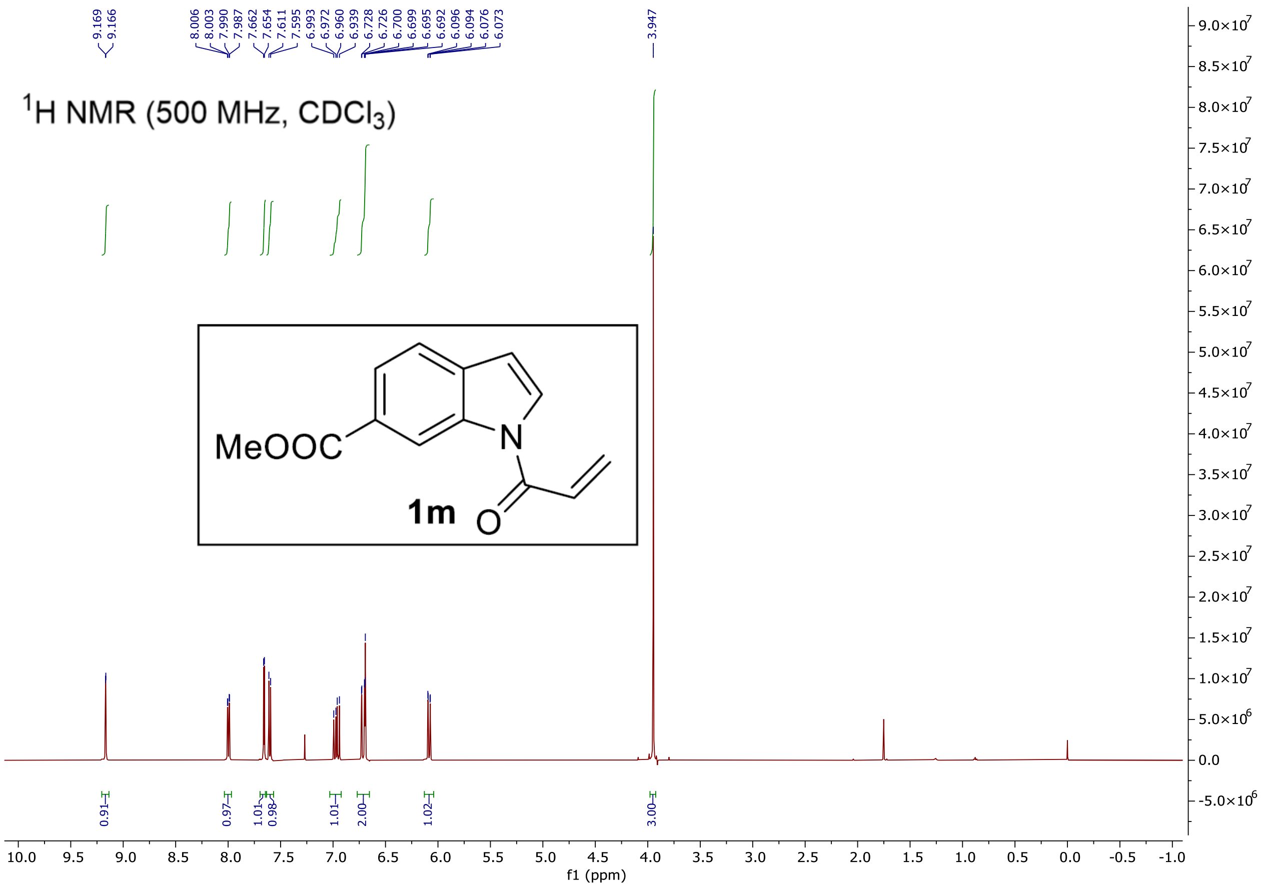
9.169
9.166
8.006
8.003
7.990
7.987
7.662
7.654
7.611
7.595
6.993
6.972
6.960
6.939
6.728
6.726
6.700
6.699
6.695
6.692
6.096
6.094
6.076
6.073
3.947
1H NMR (500 MHz, CDCl3)
MeOOC
N
O
1m
0.91
0.97
1.01
0.98
1.01
2.00
1.02
3.00
f1 (ppm)

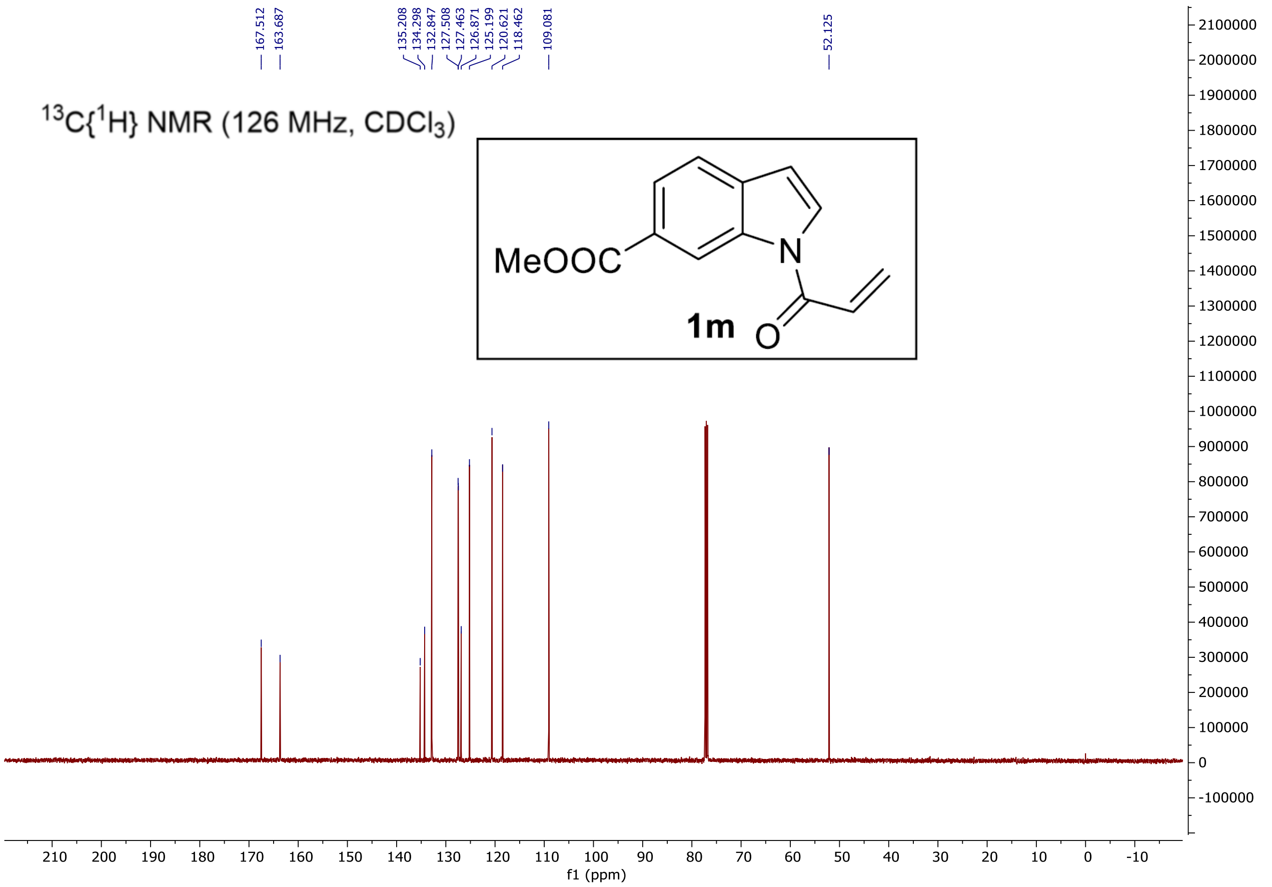

167.512
163.687
135.208
134.298
132.847
127.508
127.463
126.871
125.199
120.621
118.462
109.081
52.125
13C{1H} NMR (126 MHz, CDCl3)
MeOOC
N
O
1m
f1 (ppm)

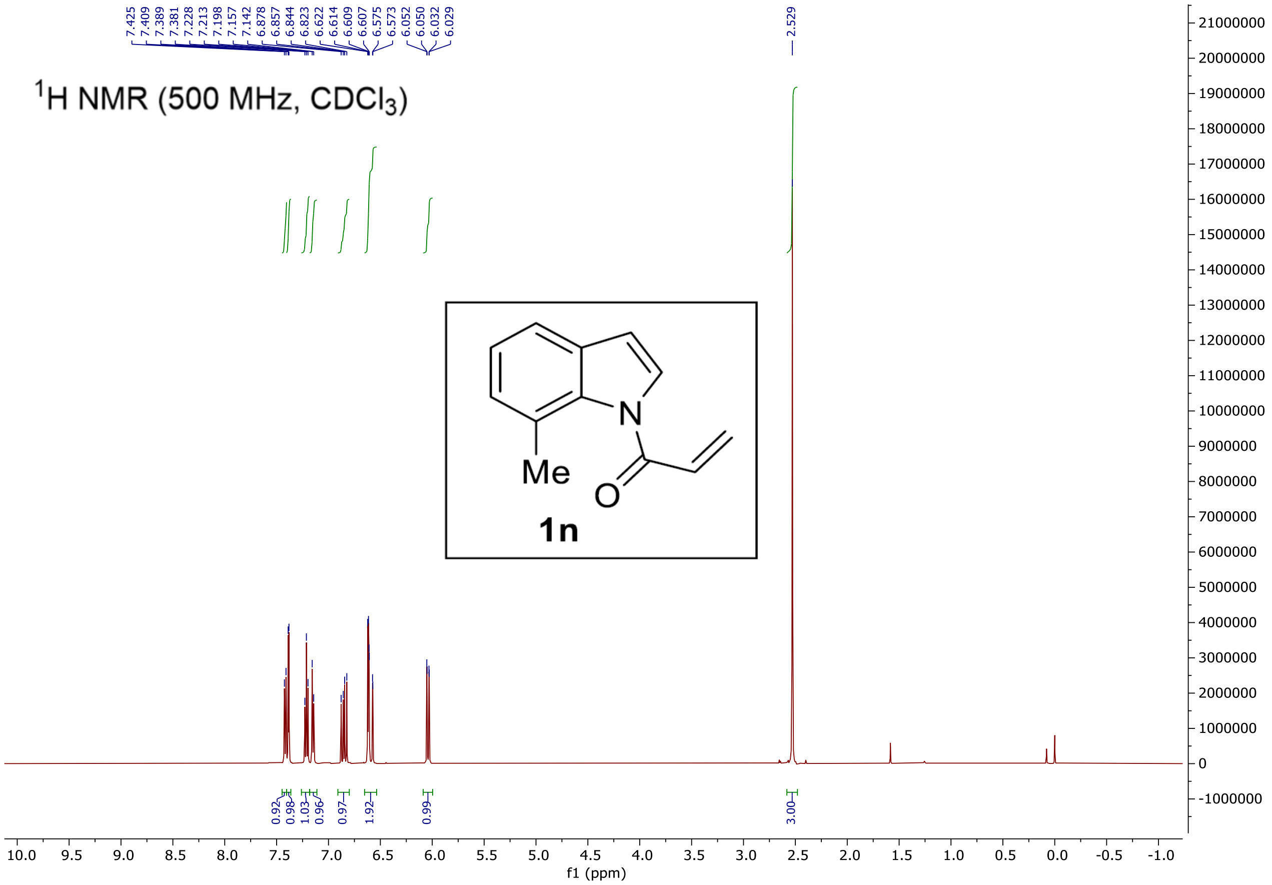

7.425
7.409
7.389
7.381
7.228
7.213
7.198
7.157
7.142
6.878
6.857
6.844
6.823
6.622
6.614
6.609
6.607
6.575
6.573
6.052
6.050
6.032
6.029
2.529
1H NMR (500 MHz, CDCl3)
N
Me
O
1n
0.92
0.98
1.03
0.96
0.97
1.92
0.99
3.00
f1 (ppm)

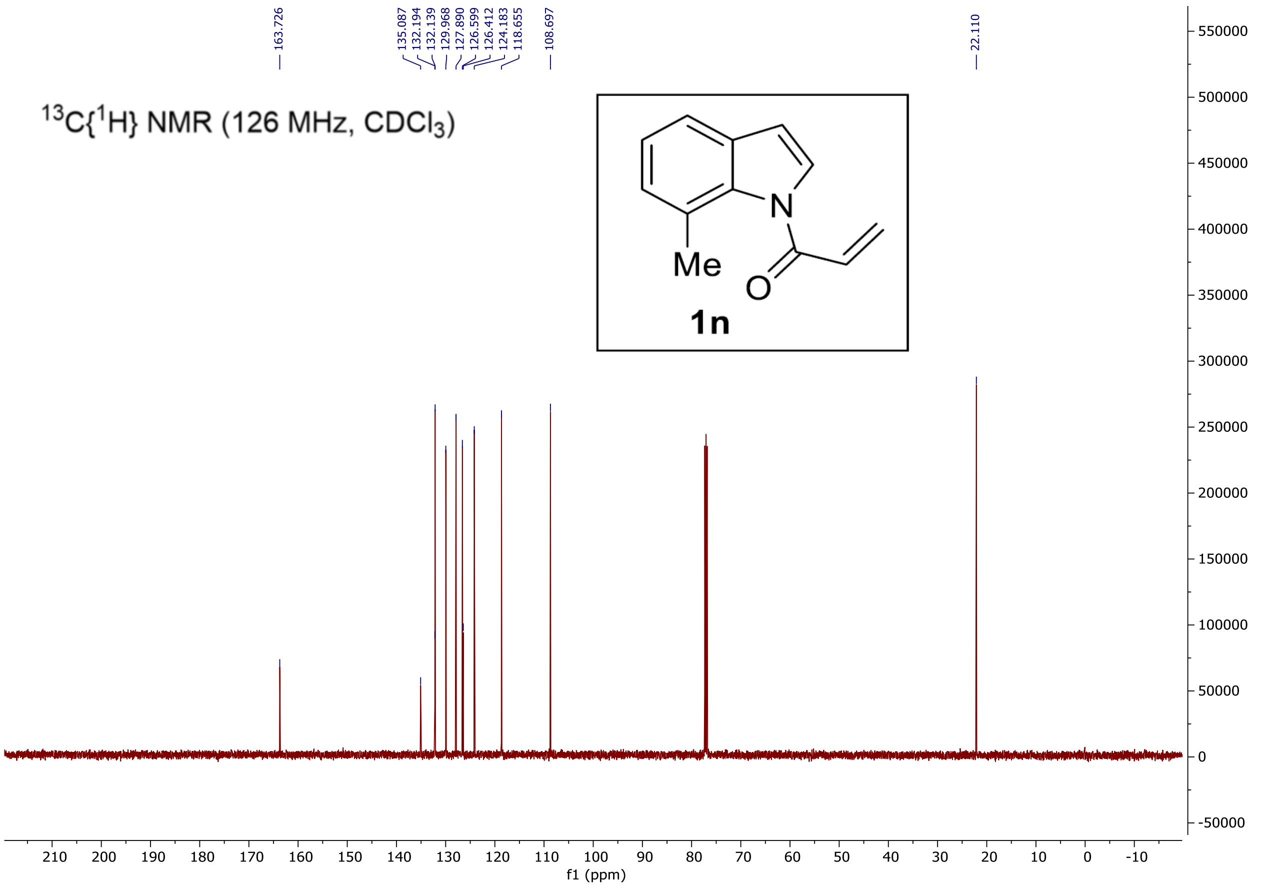

163.726
135.087
132.194
132.139
129.968
127.890
126.599
126.412
124.183
118.655
108.697
22.110
13C{1H} NMR (126 MHz, CDCl3)
Me
N
O
1n
f1 (ppm)

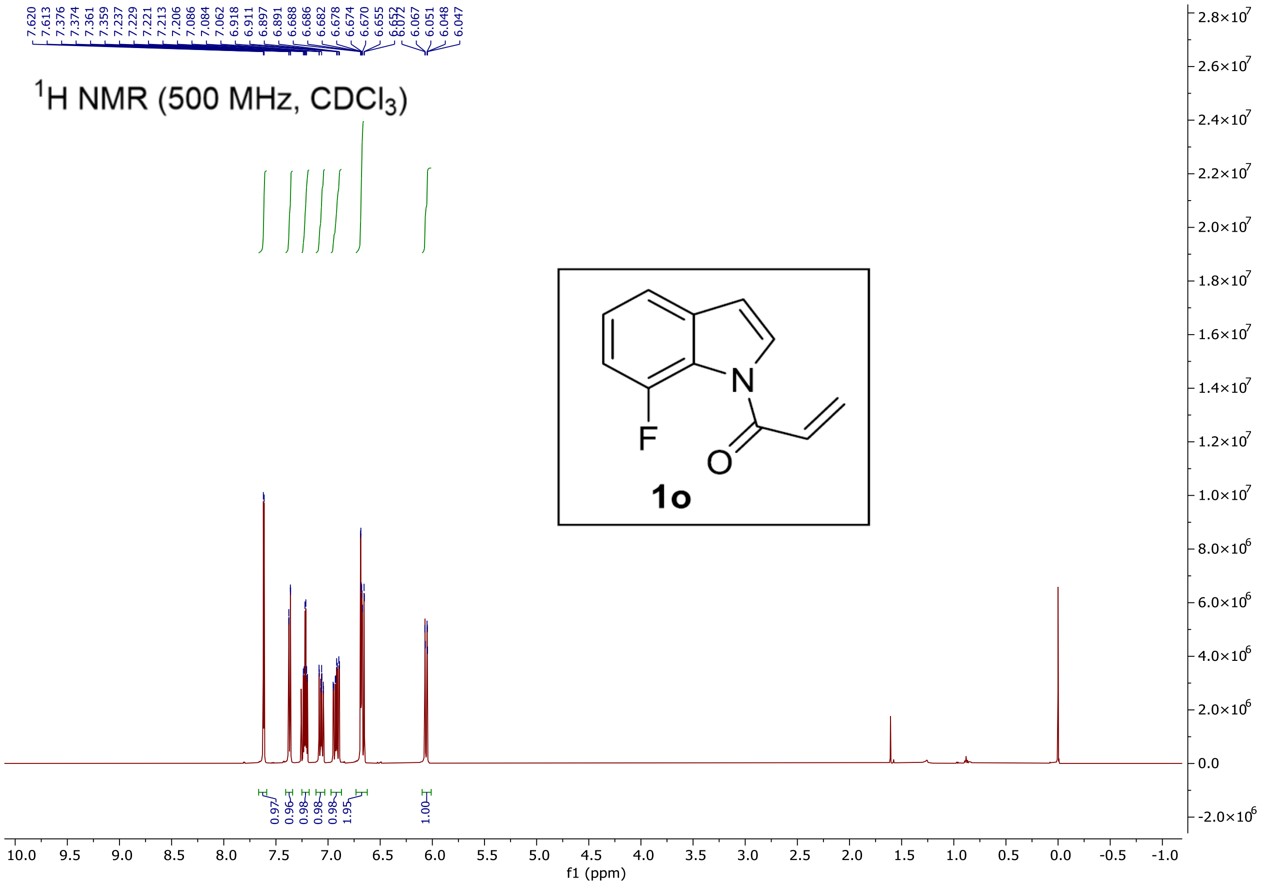

1H NMR (500 MHz, CDCl3)
F
N
O
1o
f1 (ppm)

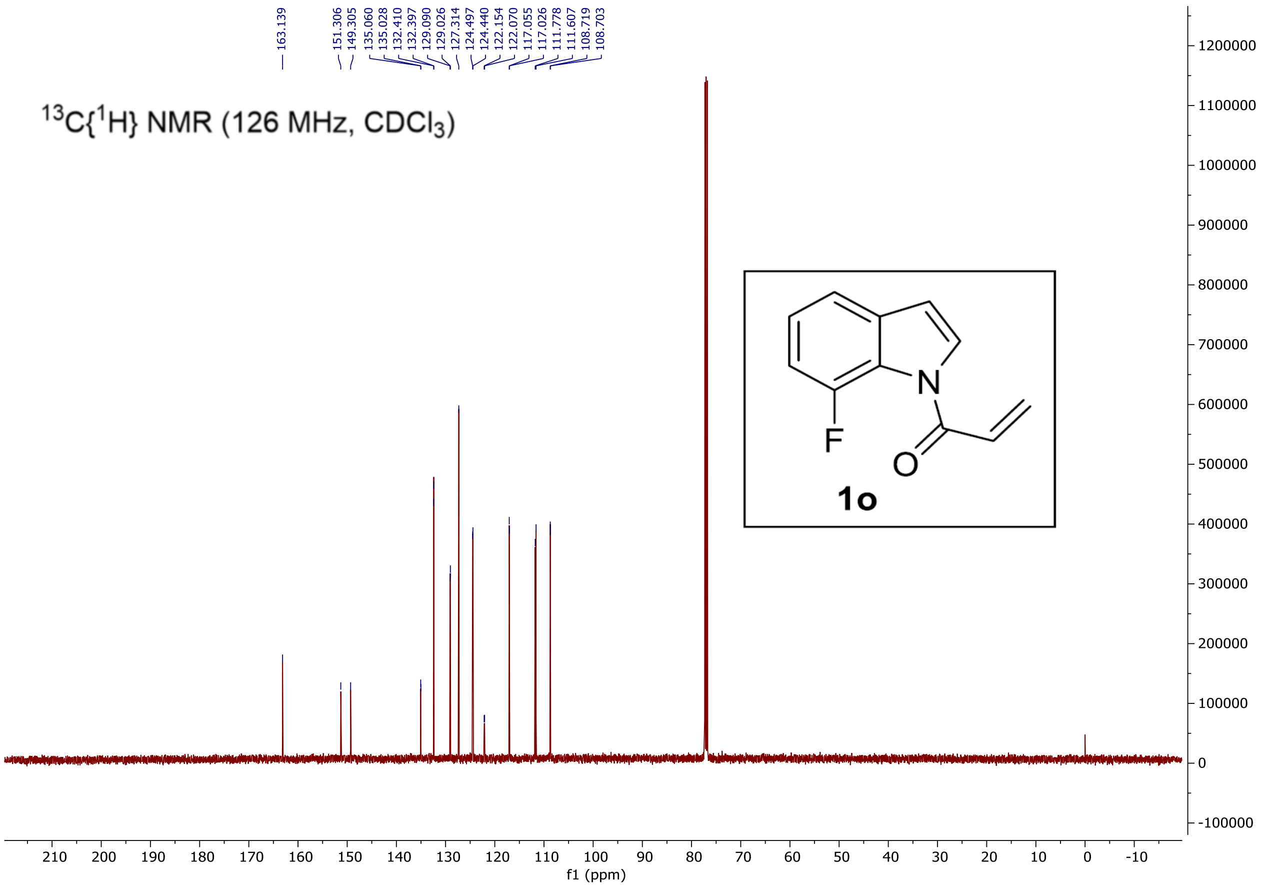

163.139
151.306
149.305
135.060
135.028
132.410
132.397
129.090
129.026
127.314
124.497
124.440
122.154
122.070
117.055
117.026
111.778
111.607
108.719
108.703
$^{13}C\{^1H\}$ NMR (126 MHz, $CDCl_3$)
N
F
O
1o
f1 (ppm)

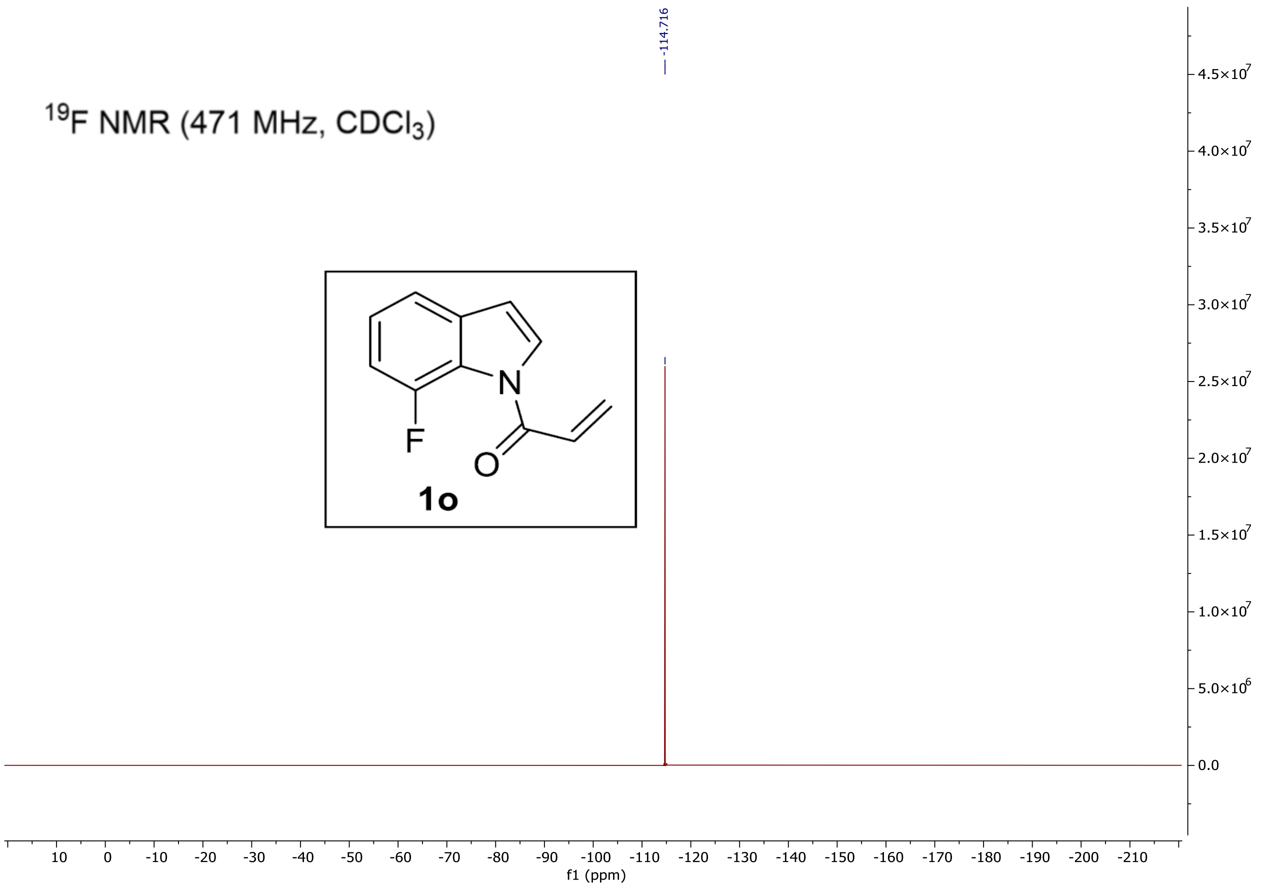

-114.716
^{19}F NMR (471 MHz, $CDCl_3$)
N
F
O
1o
f1 (ppm)

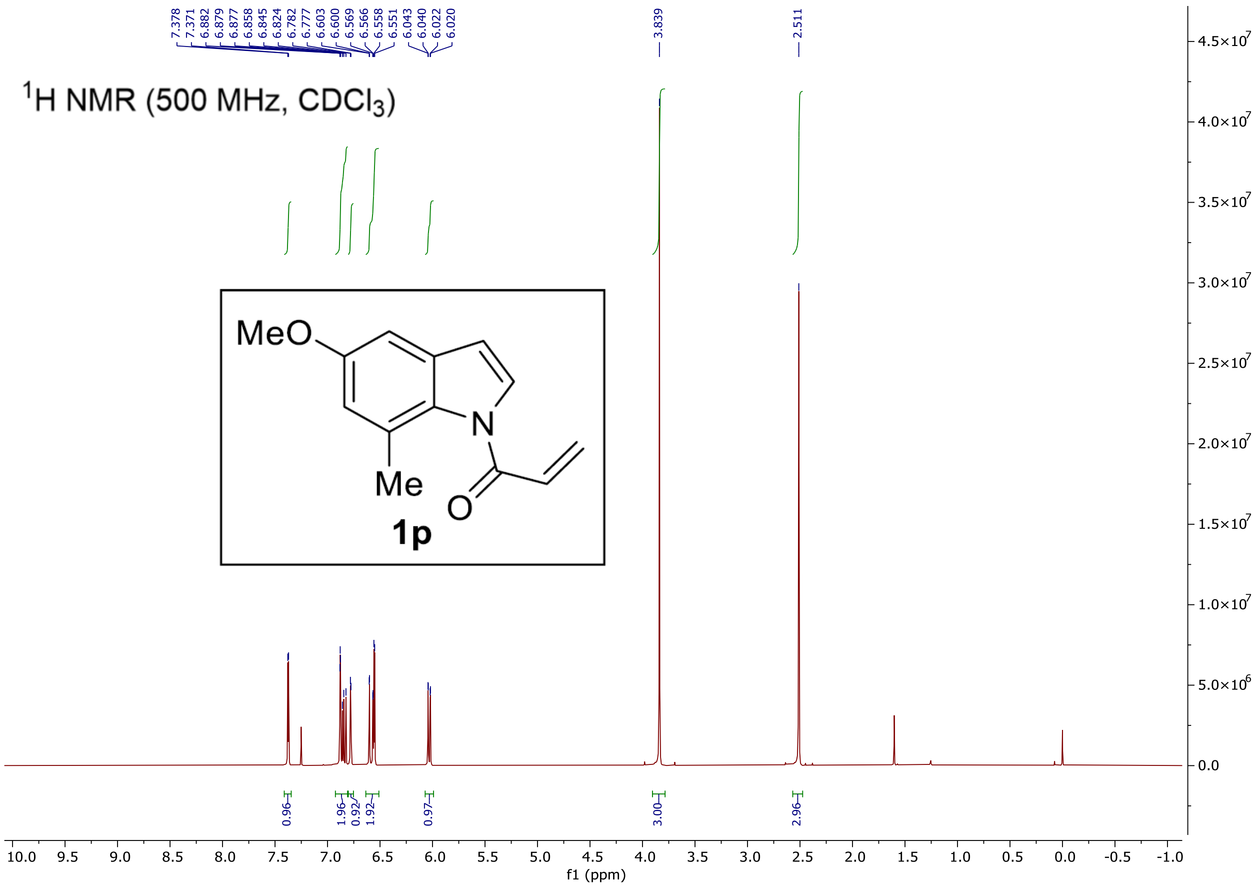

7.378
7.371
6.882
6.879
6.877
6.858
6.845
6.824
6.782
6.777
6.603
6.600
6.569
6.566
6.558
6.551
6.043
6.040
6.022
6.020
3.839
2.511
1H NMR (500 MHz, CDCl3)
MeO
N
Me
O
1p
0.96
1.96
0.92
1.92
0.97
3.00
2.96
f1 (ppm)

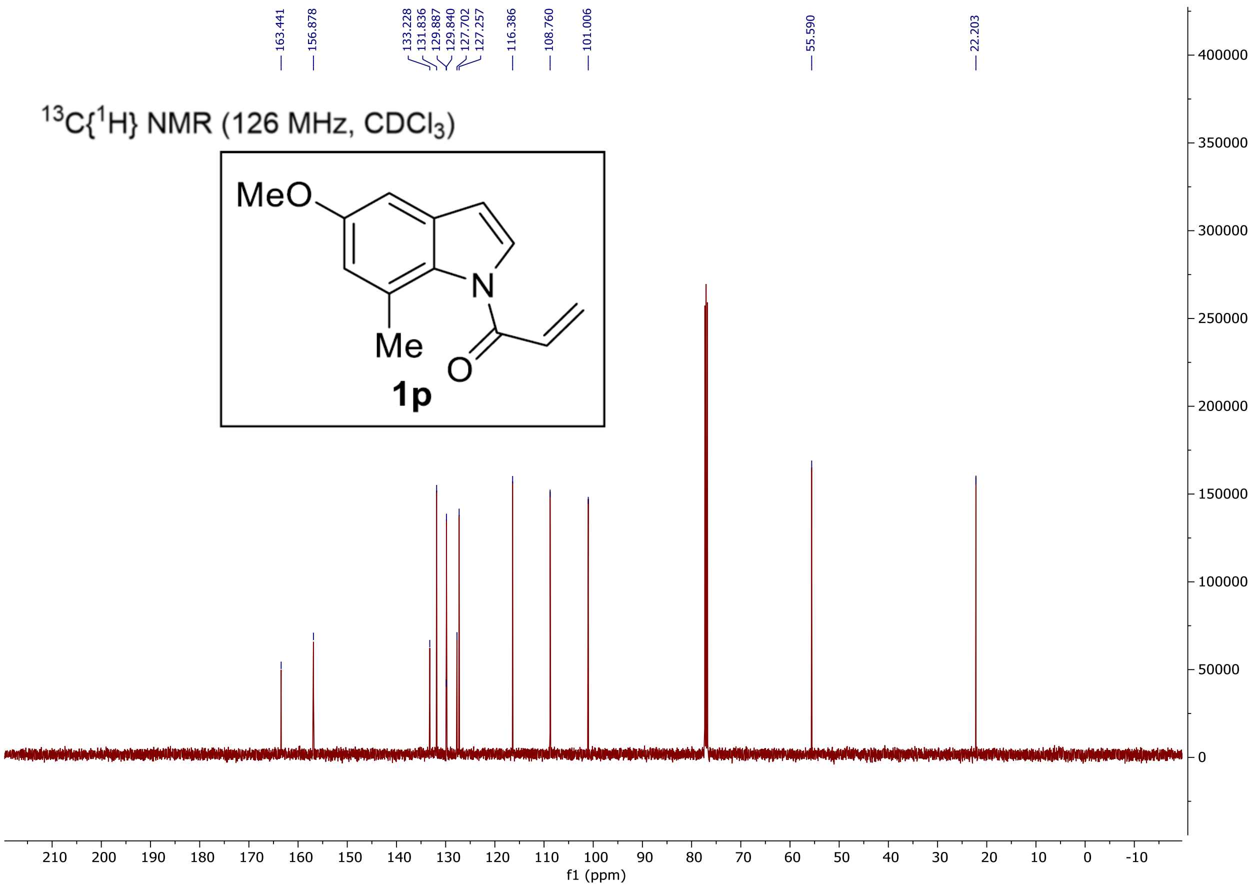

163.441
156.878
133.228
131.836
129.887
129.840
127.702
127.257
116.386
108.760
101.006
55.590
22.203
13C{1H} NMR (126 MHz, CDCl3)
MeO
N
Me
O
1p
f1 (ppm)

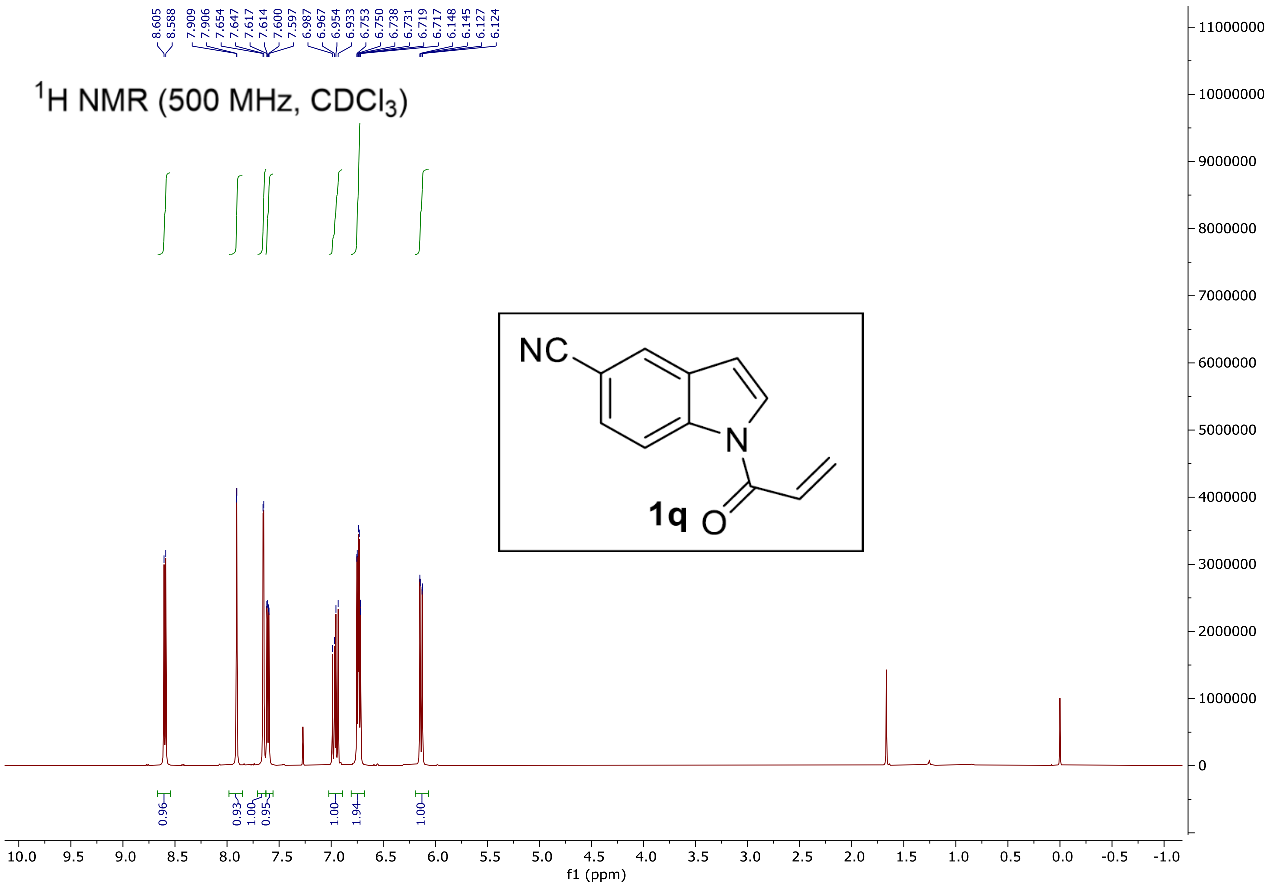

8.605
8.588
7.909
7.906
7.654
7.647
7.617
7.614
7.600
7.597
6.987
6.967
6.954
6.933
6.753
6.750
6.738
6.731
6.719
6.717
6.148
6.145
6.127
6.124
¹H NMR (500 MHz, CDCl₃)
NC
N
1q
O
0.96
0.93
1.00
0.95
1.00
1.94
1.00
f1 (ppm)

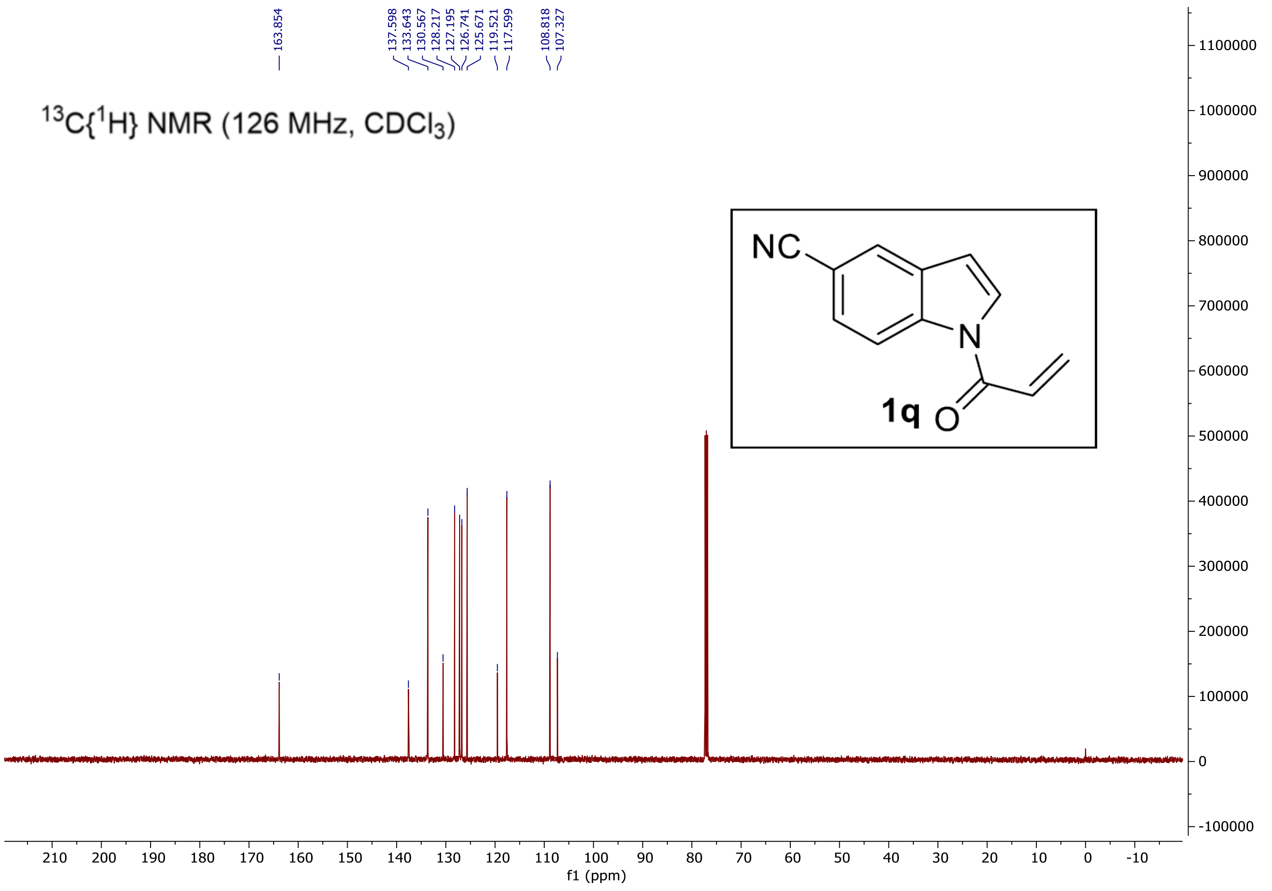

163.854
137.598
133.643
130.567
128.217
127.195
126.741
125.671
119.521
117.599
108.818
107.327
¹³C{¹H} NMR (126 MHz, CDCl₃)
NC
N
1q
O
f1 (ppm)

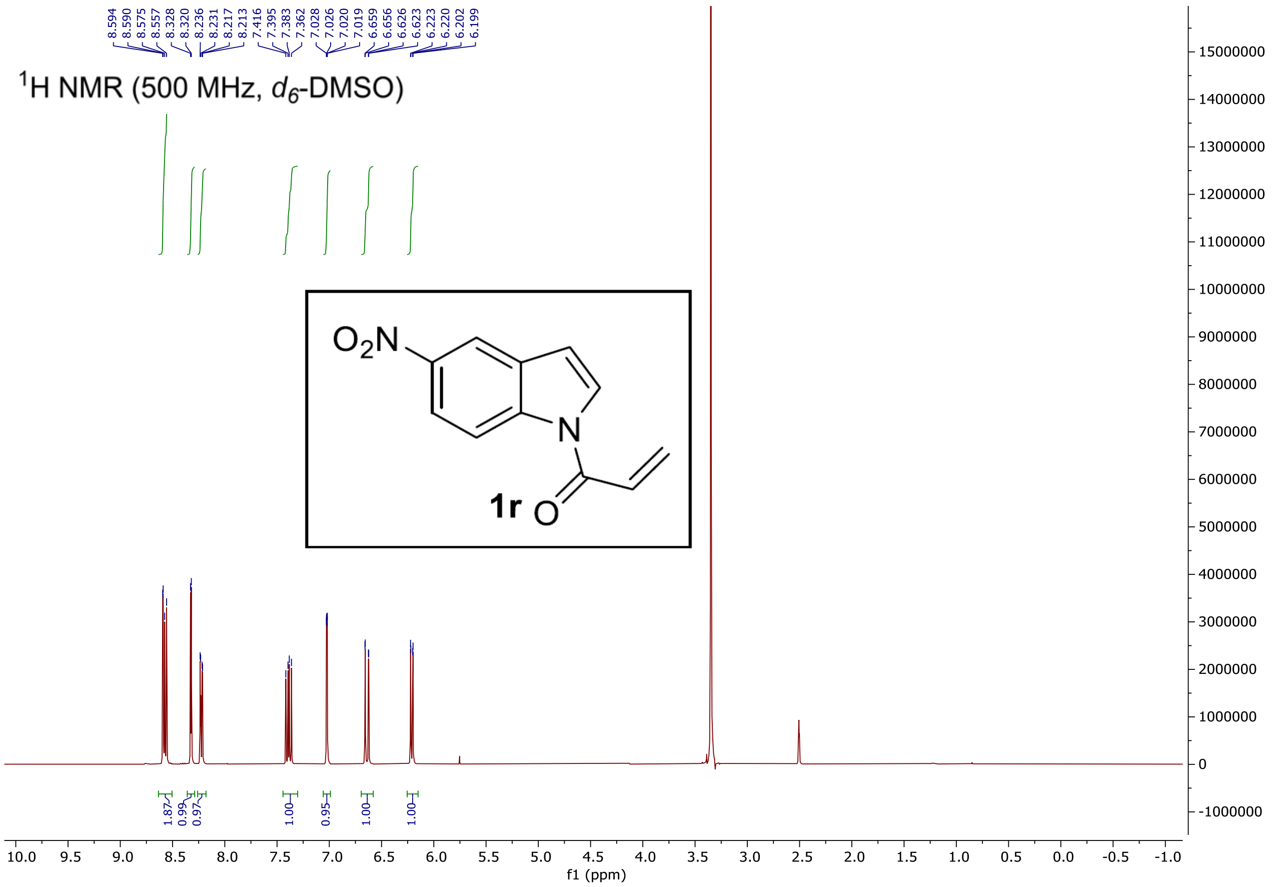
8.594 8.590 8.575 8.557 8.328 8.320 8.236 8.231 8.217 8.213 7.416 7.395 7.383 7.362 7.028 7.026 7.020 7.019 6.659 6.656 6.626 6.623 6.223 6.220 6.202 6.199
1H NMR (500 MHz, d6-DMSO)
O2N
N
O
1r
1.87 0.99 0.97 1.00 0.95 1.00 1.00
f1 (ppm)

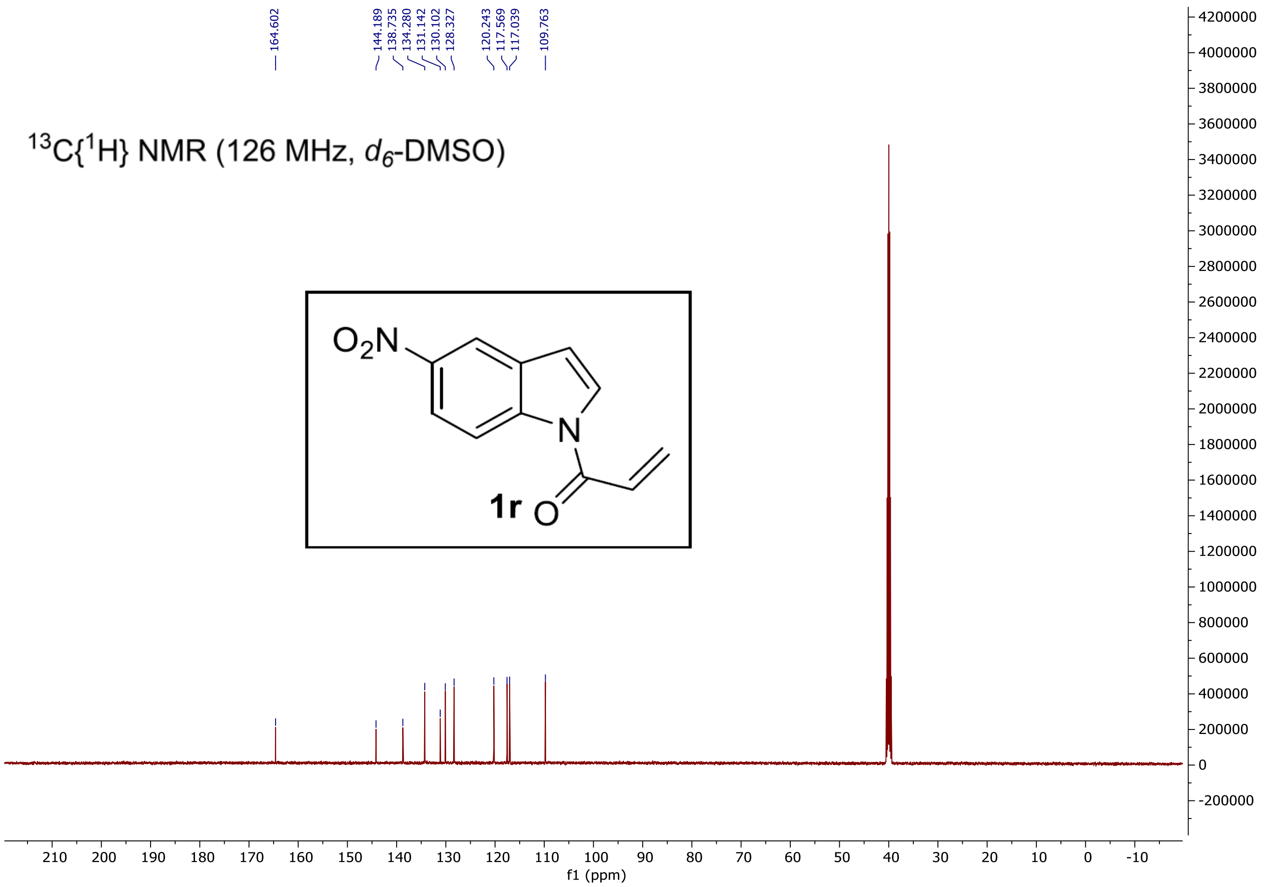
164.602
144.189 138.735 134.280 131.142 130.102 128.327
120.243 117.569 117.039
109.763
13C{1H} NMR (126 MHz, d6-DMSO)
O2N
N
O
1r
f1 (ppm)

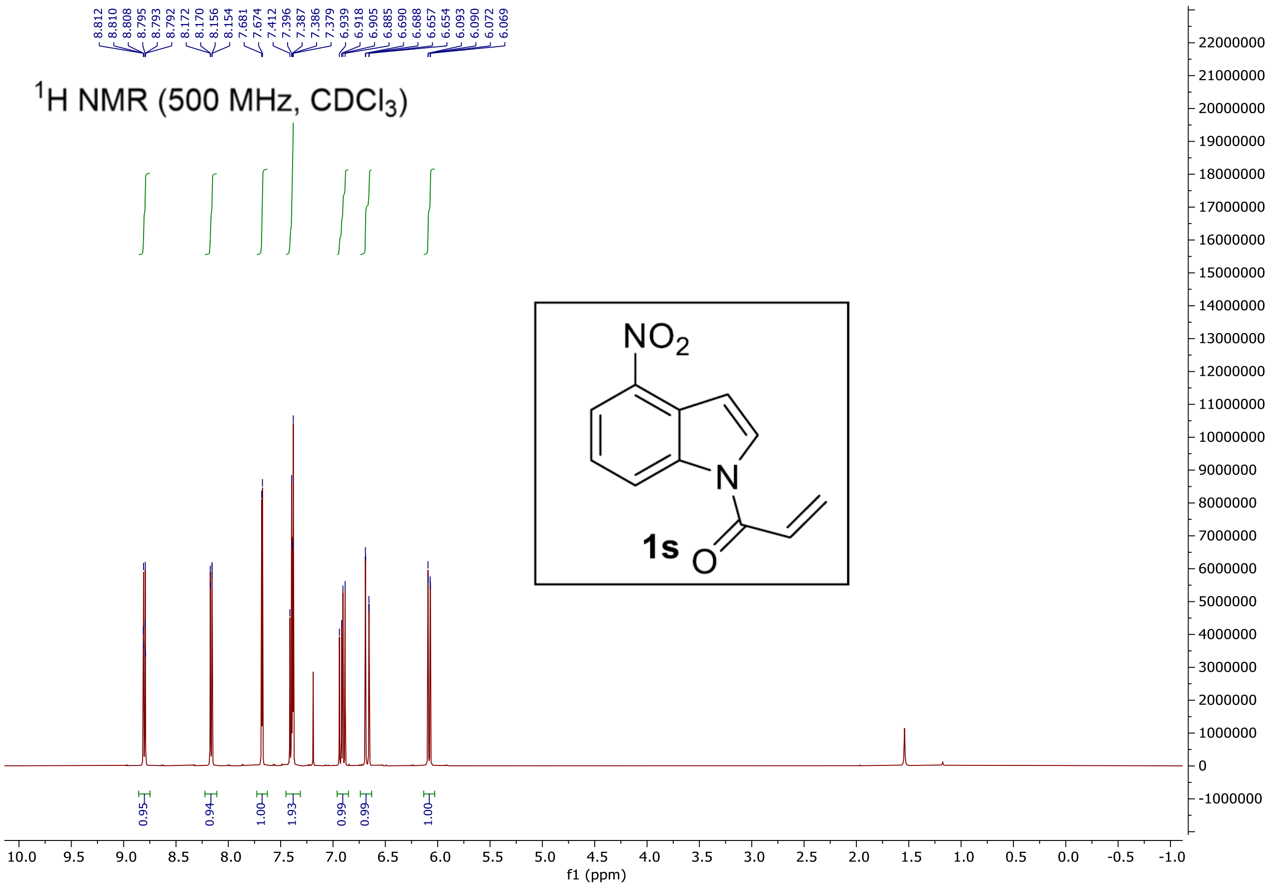

8.812
8.810
8.808
8.795
8.793
8.792
8.172
8.170
8.156
8.154
7.681
7.674
7.412
7.396
7.387
7.386
7.379
6.939
6.918
6.905
6.885
6.690
6.688
6.657
6.654
6.093
6.090
6.072
6.069
^{1}H NMR (500 MHz, CDCl$_3$)
NO_2
N
O
1s
0.95
0.94
1.00
1.93
0.99
0.99
1.00
f1 (ppm)

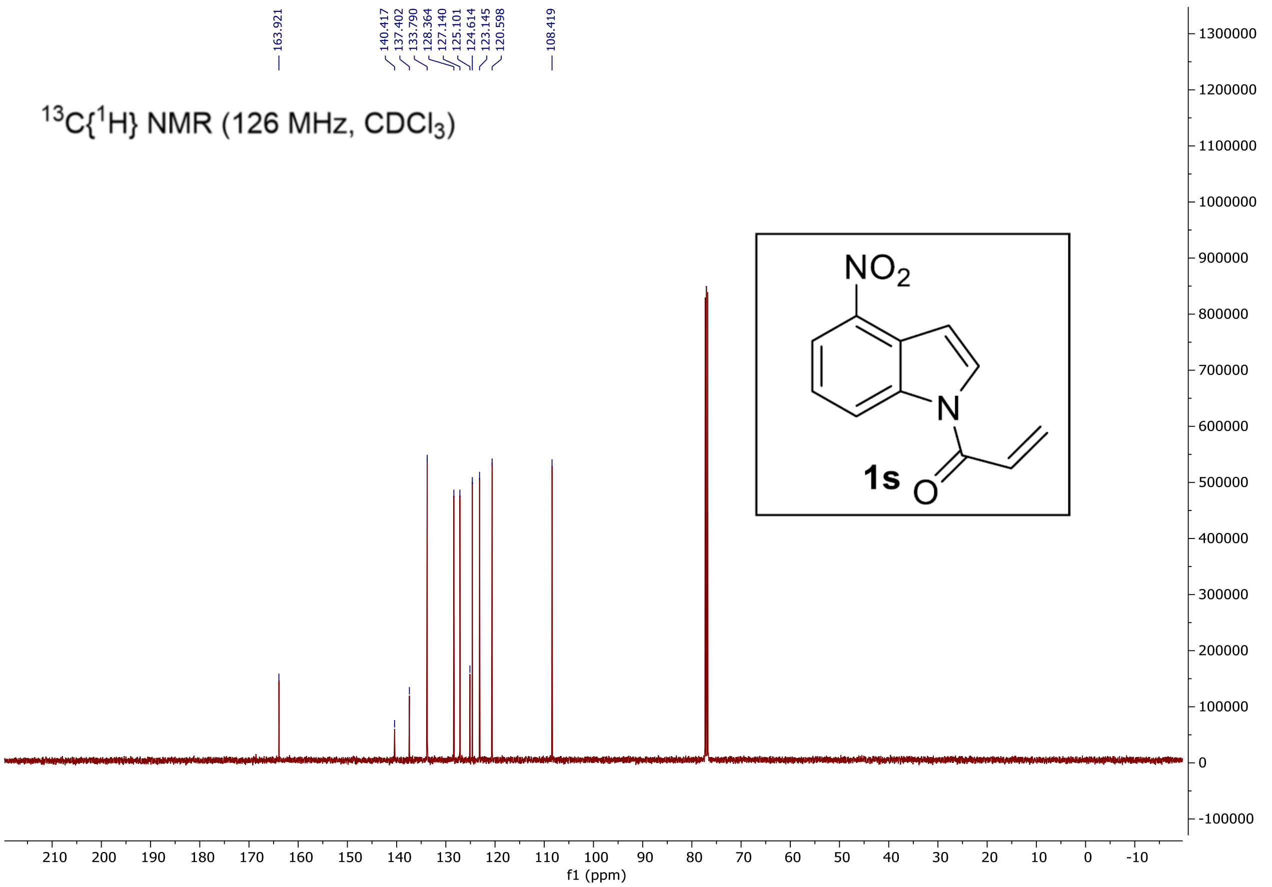

163.921
140.417
137.402
133.790
128.364
127.140
125.101
124.614
123.145
120.598
108.419
^{13}C{^{1}H} NMR (126 MHz, CDCl$_3$)
NO_2
N
O
1s
f1 (ppm)

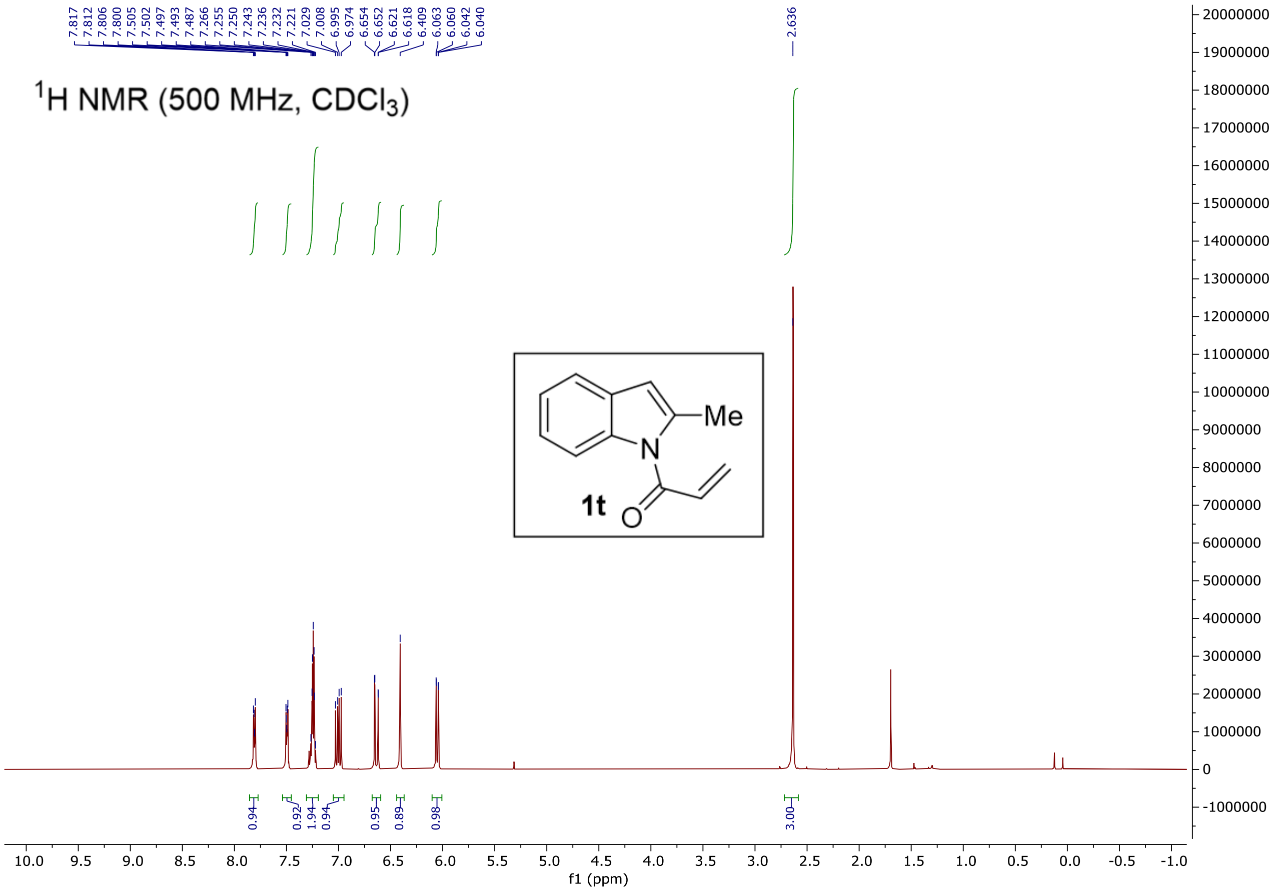

7.817
7.812
7.806
7.800
7.505
7.502
7.497
7.493
7.487
7.266
7.255
7.250
7.243
7.236
7.232
7.221
7.029
7.008
6.995
6.974
6.654
6.652
6.621
6.618
6.409
6.063
6.060
6.042
6.040
2.636
1H NMR (500 MHz, CDCl3)
Me
N
1t
O
0.94
0.92
1.94
0.94
0.95
0.89
0.98
3.00
f1 (ppm)

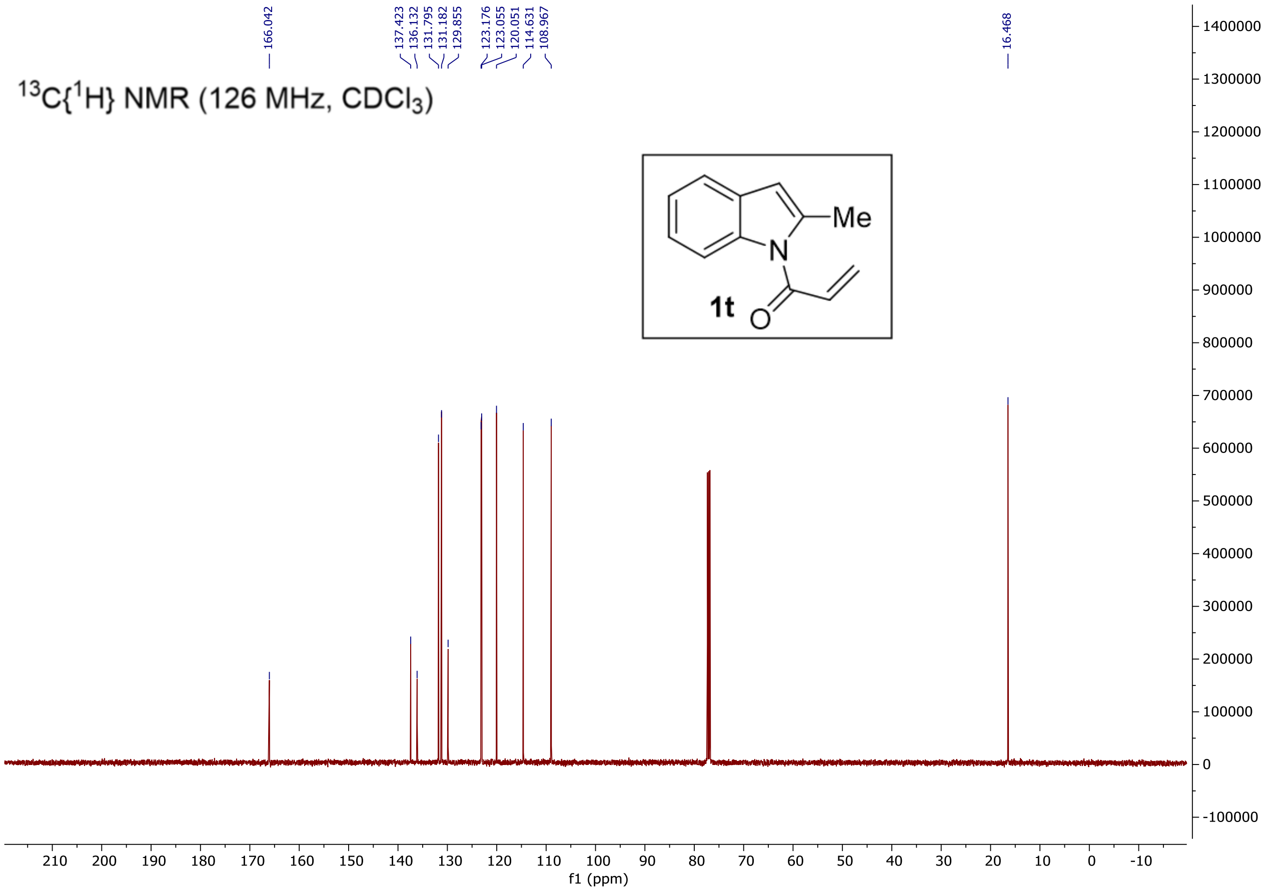

166.042
137.423
136.132
131.795
131.182
129.855
123.176
123.055
120.051
114.631
108.967
16.468
13C{1H} NMR (126 MHz, CDCl3)
Me
N
1t
O
f1 (ppm)

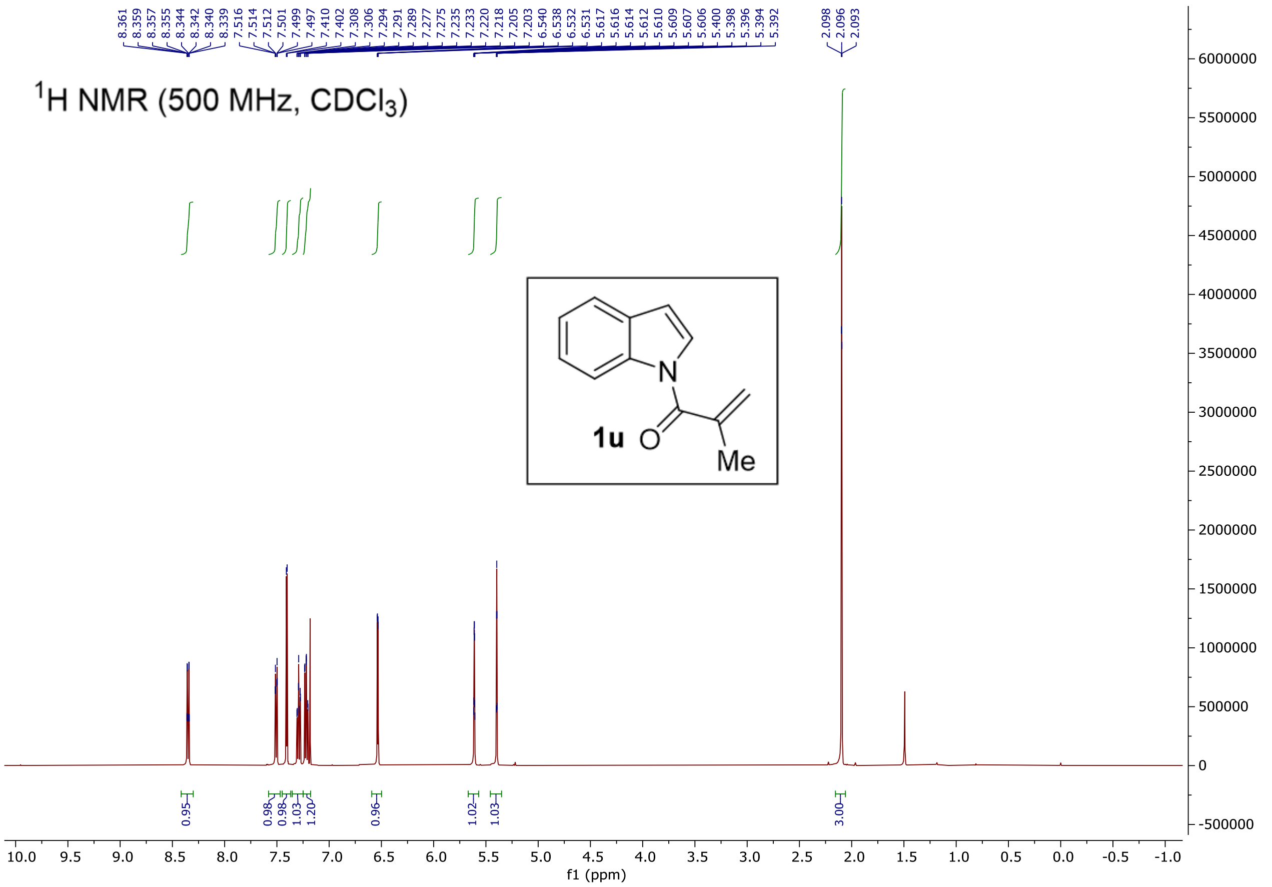

8.361
8.359
8.357
8.355
8.344
8.342
8.340
8.339
7.516
7.514
7.512
7.501
7.499
7.497
7.410
7.402
7.308
7.306
7.294
7.291
7.289
7.277
7.275
7.235
7.233
7.220
7.218
7.205
7.203
6.540
6.538
6.532
6.531
5.617
5.616
5.614
5.612
5.610
5.609
5.607
5.606
5.400
5.398
5.396
5.394
5.392
2.098
2.096
2.093
^{1}H NMR (500 MHz, CDCl$_3$)
N
1u
O
Me
0.95
0.98
0.98
1.03
1.20
0.96
1.02
1.03
3.00
f1 (ppm)

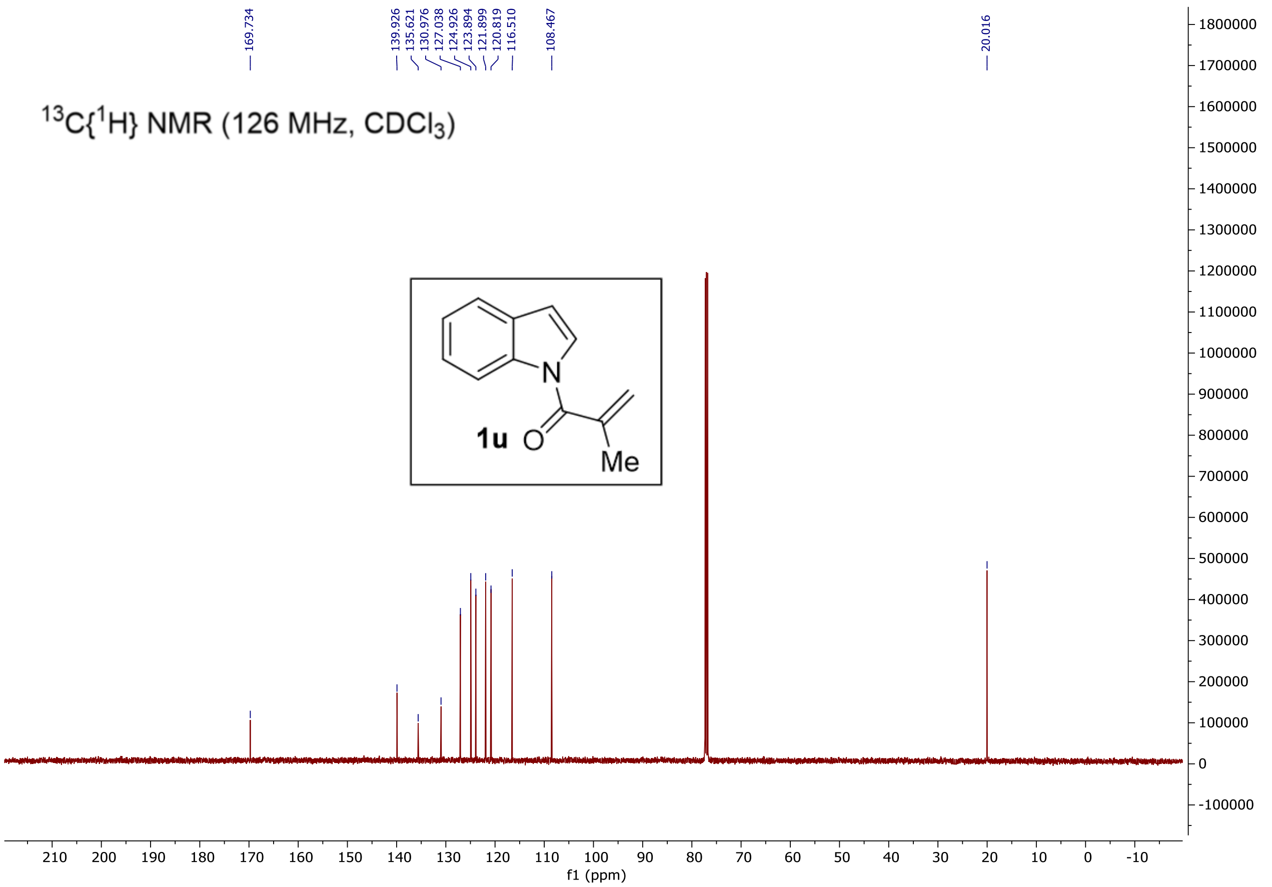

169.734
139.926
135.621
130.976
127.038
124.926
123.894
121.899
120.819
116.510
108.467
20.016
^{13}C{^{1}H} NMR (126 MHz, CDCl$_3$)
N
1u
O
Me
f1 (ppm)

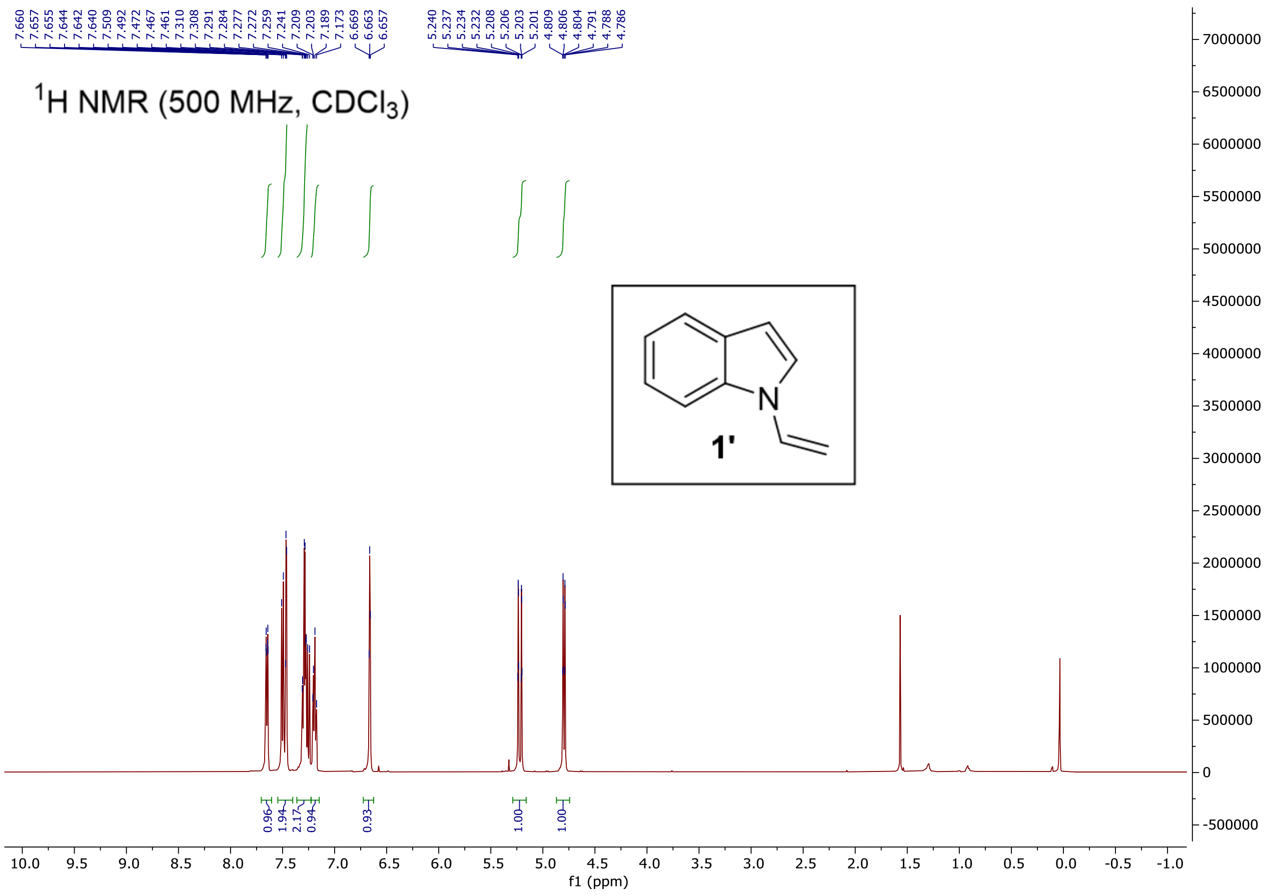

7.660
7.657
7.655
7.644
7.642
7.640
7.509
7.492
7.472
7.467
7.461
7.310
7.308
7.291
7.284
7.277
7.272
7.259
7.241
7.209
7.203
7.189
7.173
6.669
6.663
6.657
5.240
5.237
5.234
5.232
5.208
5.206
5.203
5.201
4.809
4.806
4.804
4.791
4.788
4.786
^{1}H NMR (500 MHz, CDCl$_3$)
1'
0.96
1.94
2.17
0.94
0.93
1.00
1.00
f1 (ppm)

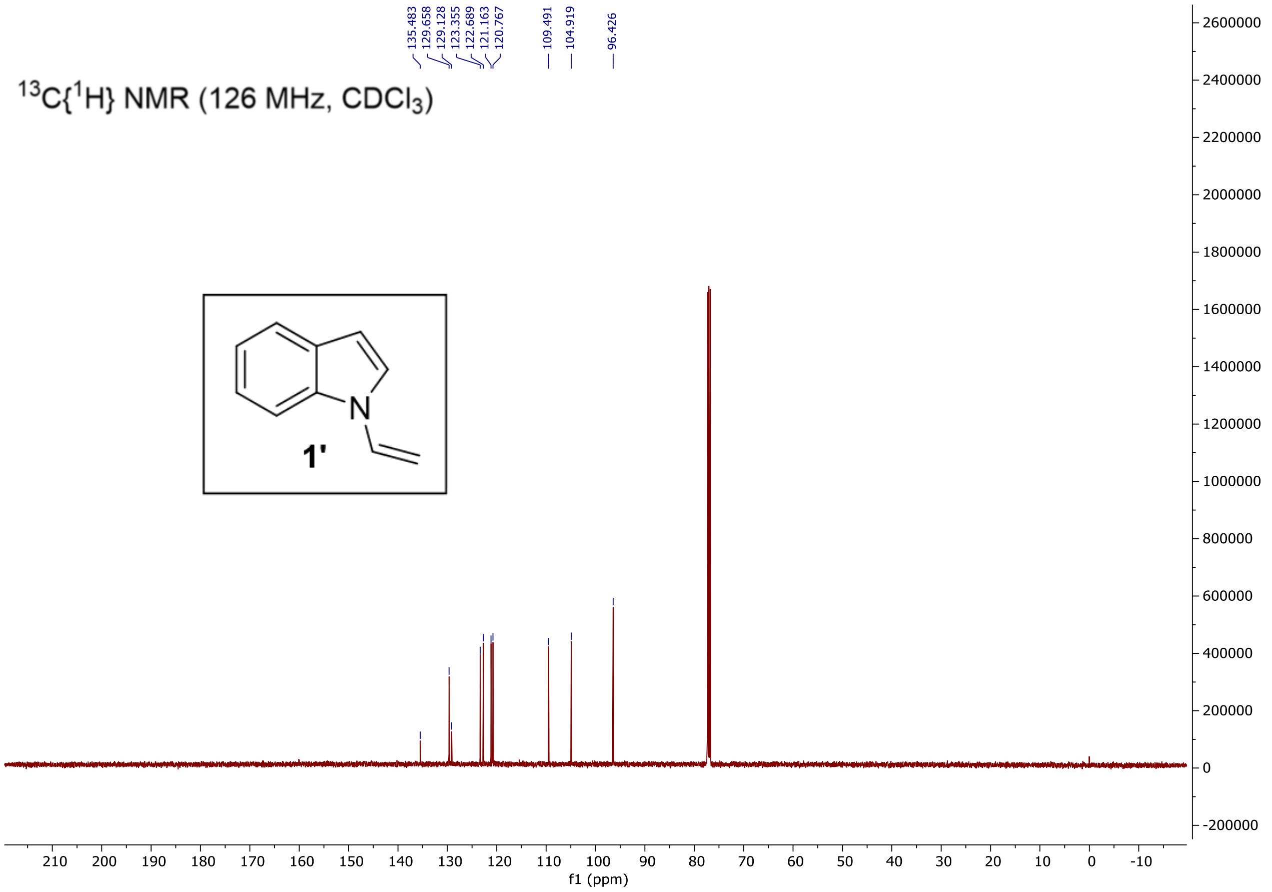

135.483
129.658
129.128
123.355
122.689
121.163
120.767
109.491
104.919
96.426
^{13}C{^{1}H} NMR (126 MHz, CDCl$_3$)
1'
f1 (ppm)

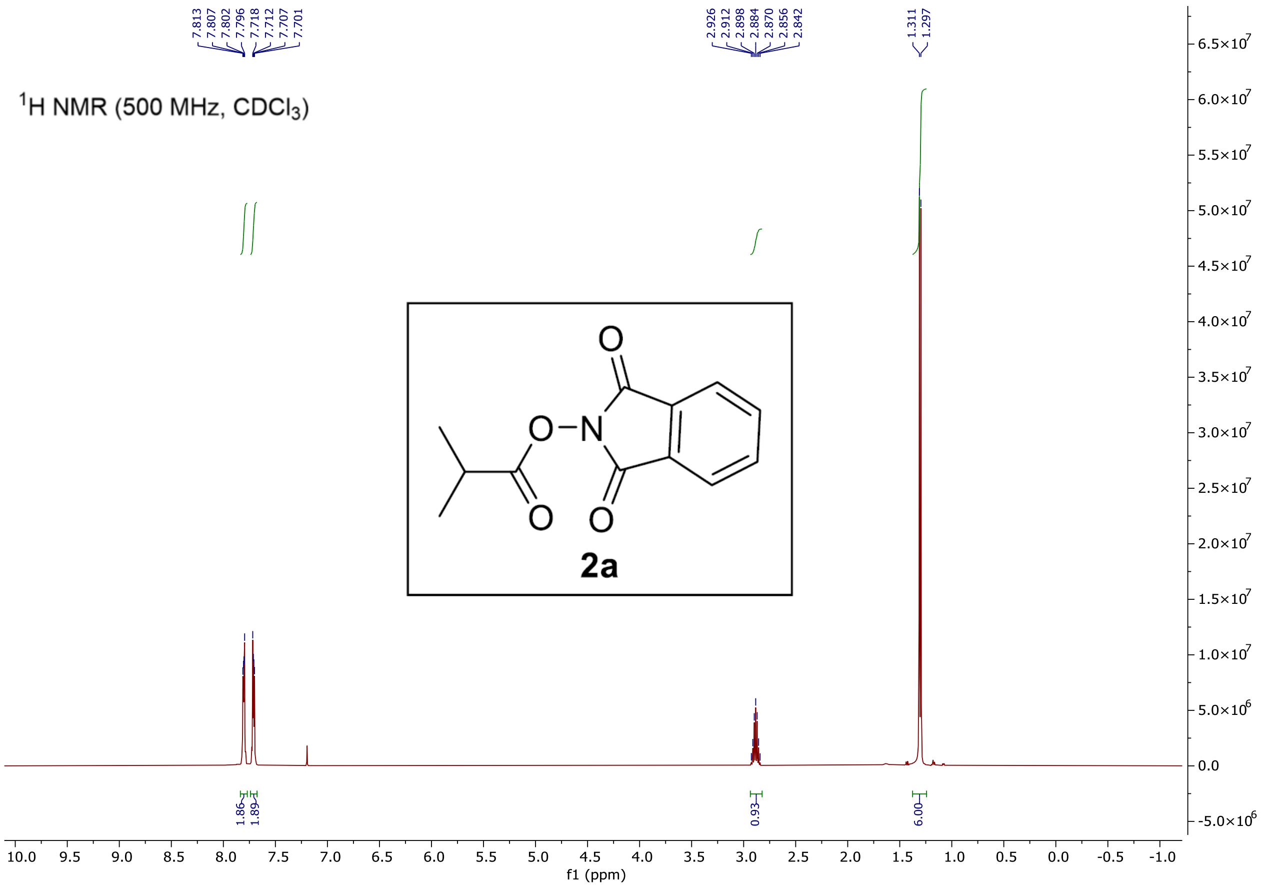
7.813 7.807 7.802 7.796 7.718 7.712 7.707 7.701
2.926 2.912 2.898 2.884 2.870 2.856 2.842
1.311 1.297
1H NMR (500 MHz, CDCl3)
2a
1.86 1.89 0.93 6.00
f1 (ppm)

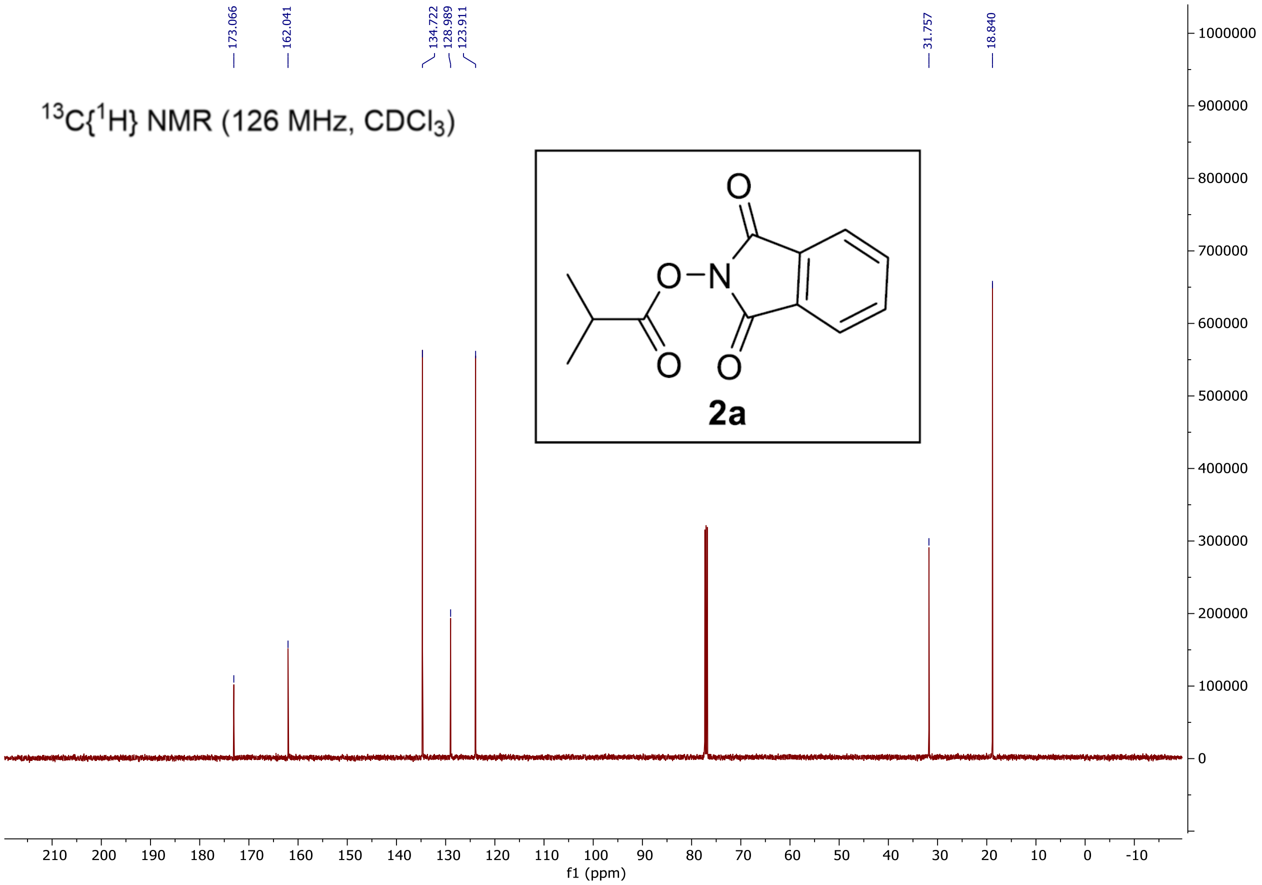
173.066 162.041 134.722 128.989 123.911 31.757 18.840
13C{1H} NMR (126 MHz, CDCl3)
2a
f1 (ppm)

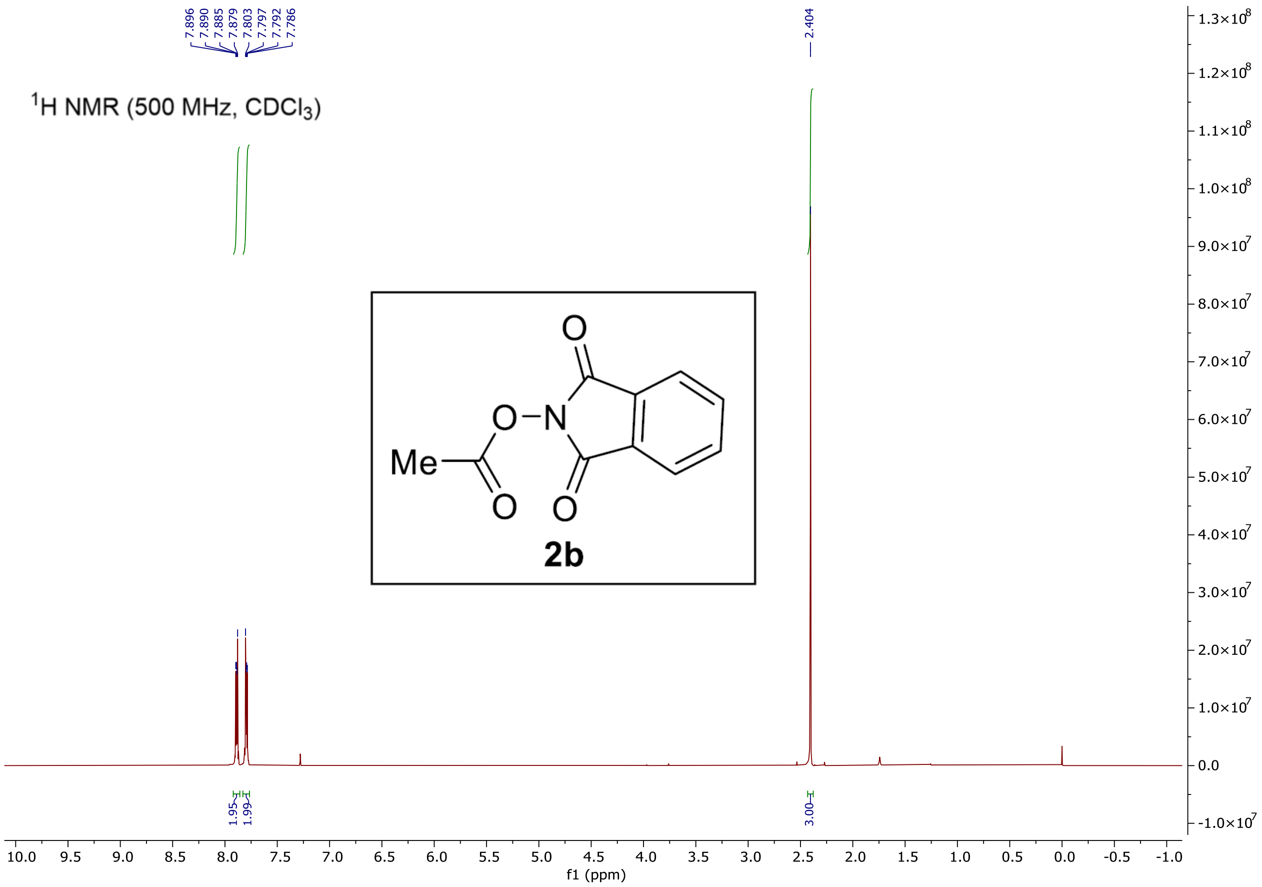

7.896
7.890
7.885
7.879
7.803
7.797
7.792
7.786
2.404
1H NMR (500 MHz, CDCl3)
O
O–N
Me
O
O
2b
1.95
1.99
3.00
f1 (ppm)

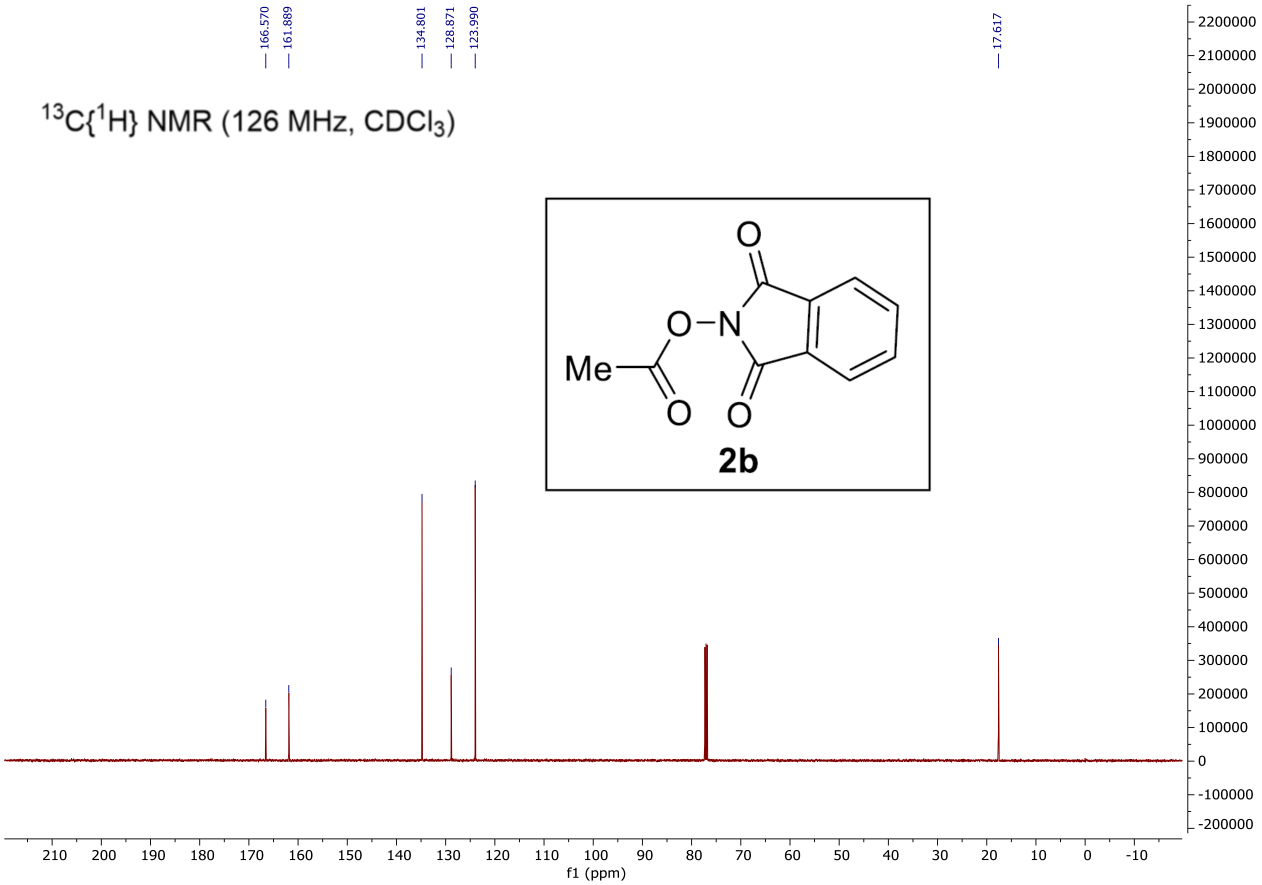

166.570
161.889
134.801
128.871
123.990
17.617
13C{1H} NMR (126 MHz, CDCl3)
O
O–N
Me
O
O
2b
f1 (ppm)

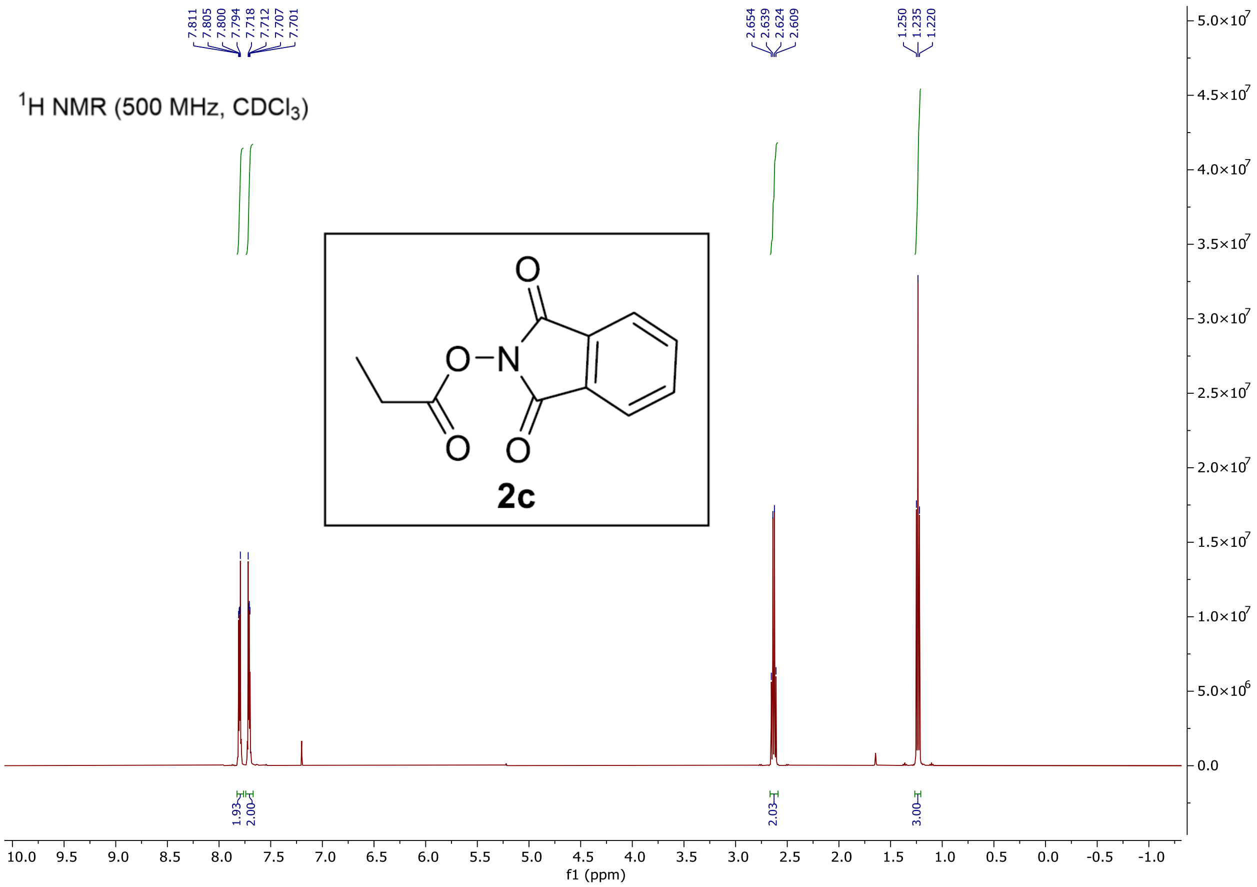

7.811
7.805
7.800
7.794
7.718
7.712
7.707
7.701
2.654
2.639
2.624
2.609
1.250
1.235
1.220
1H NMR (500 MHz, CDCl3)
2c
1.93
2.00
2.03
3.00
f1 (ppm)

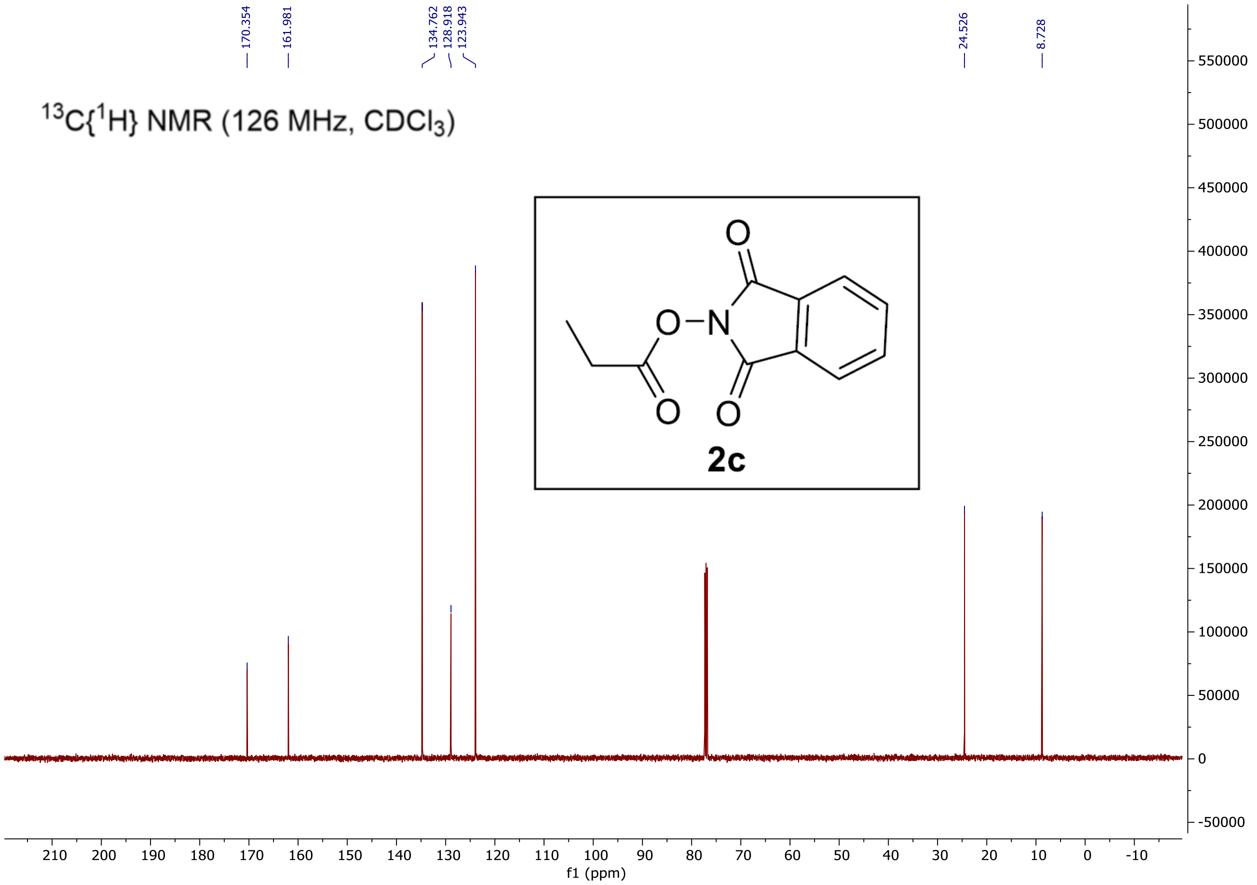

170.354
161.981
134.762
128.918
123.943
24.526
8.728
13C{1H} NMR (126 MHz, CDCl3)
2c
f1 (ppm)

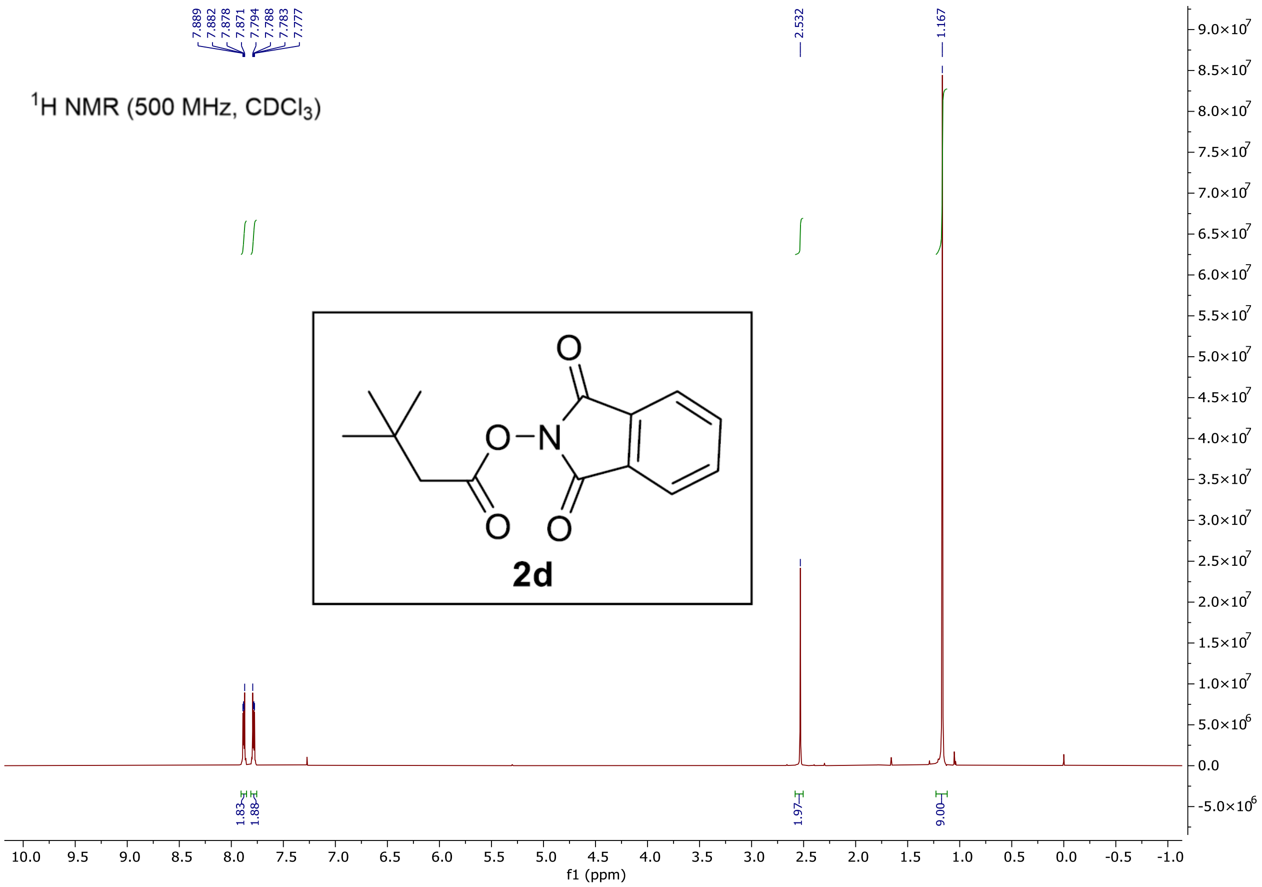
^{1}H NMR (500 MHz, CDCl$_3$)
7.889 7.882 7.878 7.871 7.794 7.788 7.783 7.777
2.532
1.167
1.83
1.88
1.97
9.00
2d
f1 (ppm)

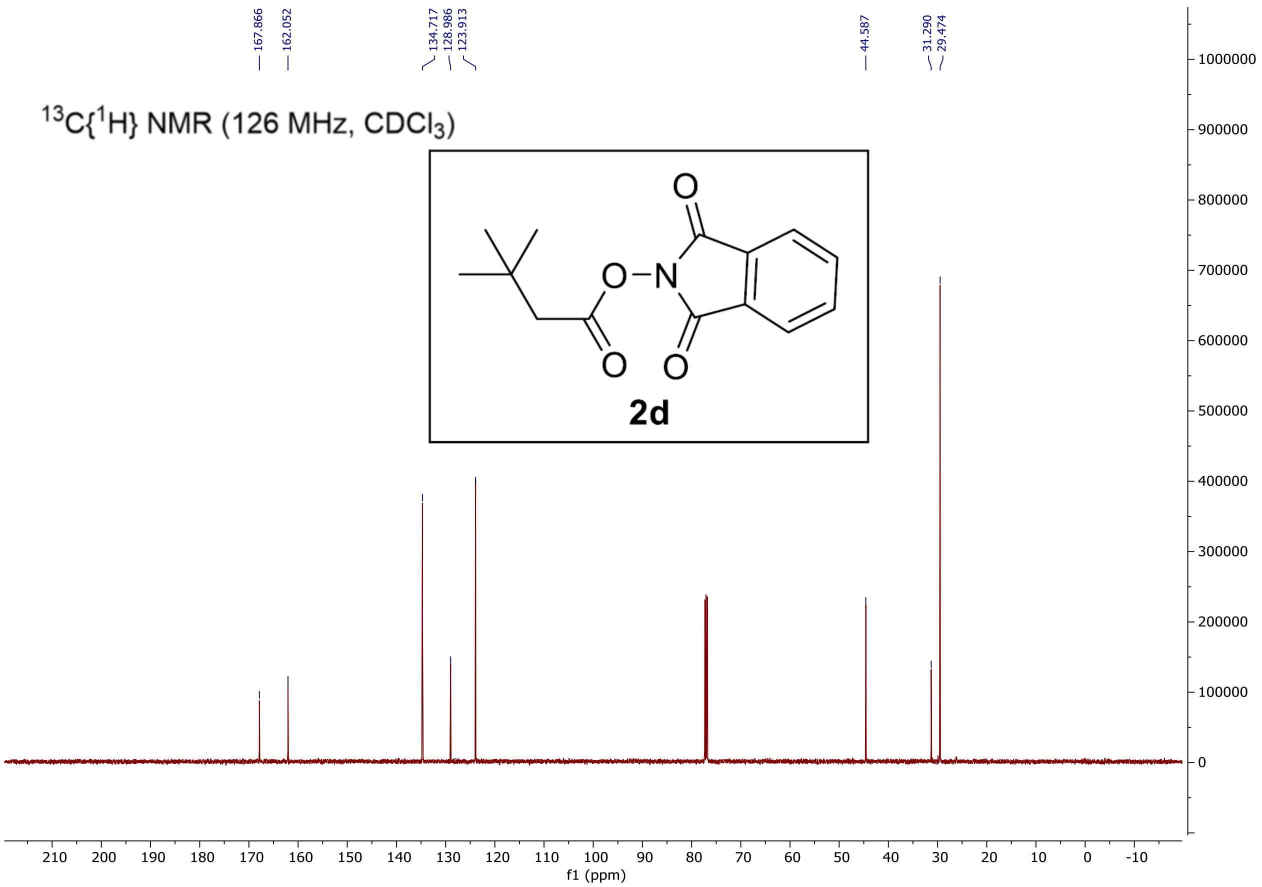
^{13}C{^{1}H} NMR (126 MHz, CDCl$_3$)
167.866
162.052
134.717 128.986 123.913
44.587
31.290 29.474
2d
f1 (ppm)

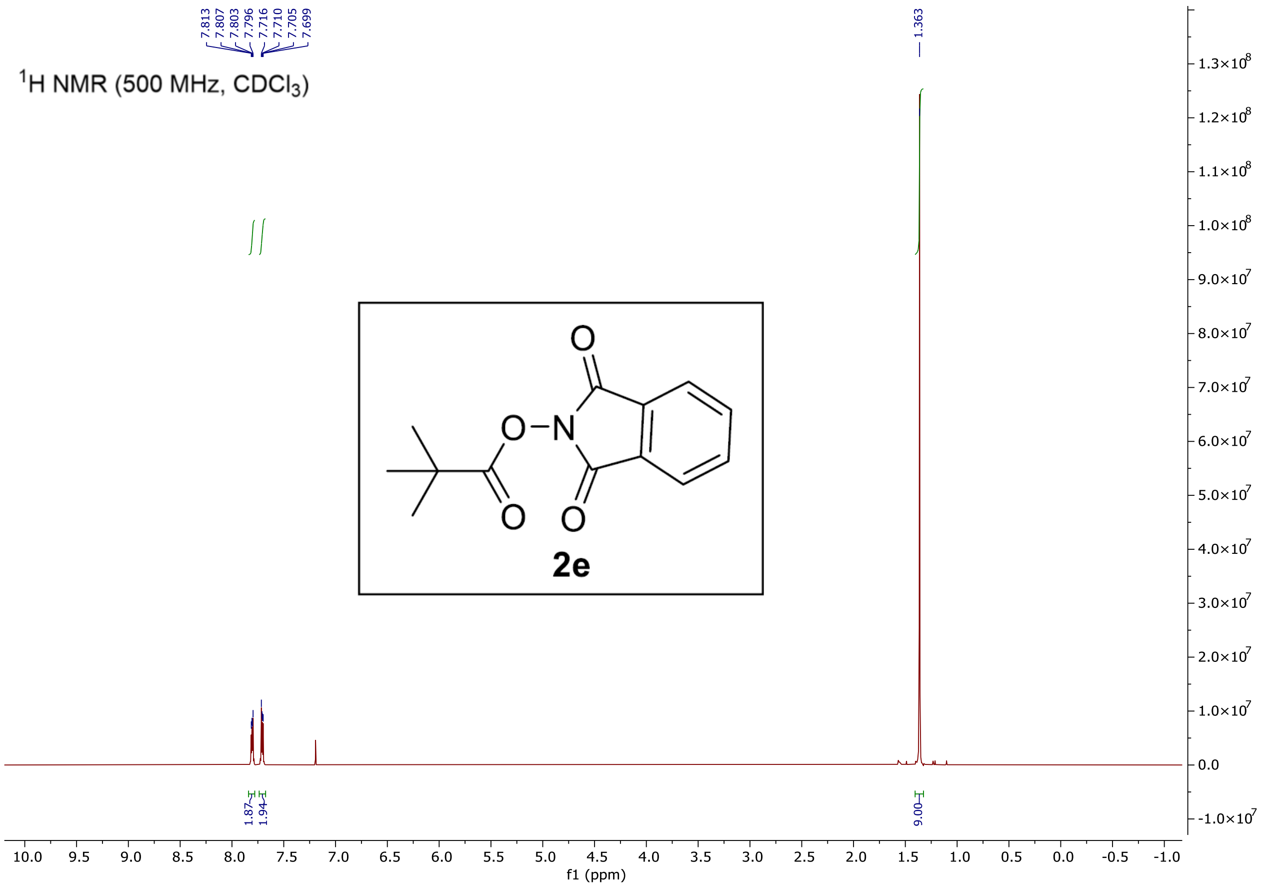

7.813
7.807
7.803
7.796
7.716
7.710
7.705
7.699
1.363
^{1}H NMR (500 MHz, CDCl$_3$)
O
O-N
O
O
2e
1.87
1.94
9.00
f1 (ppm)

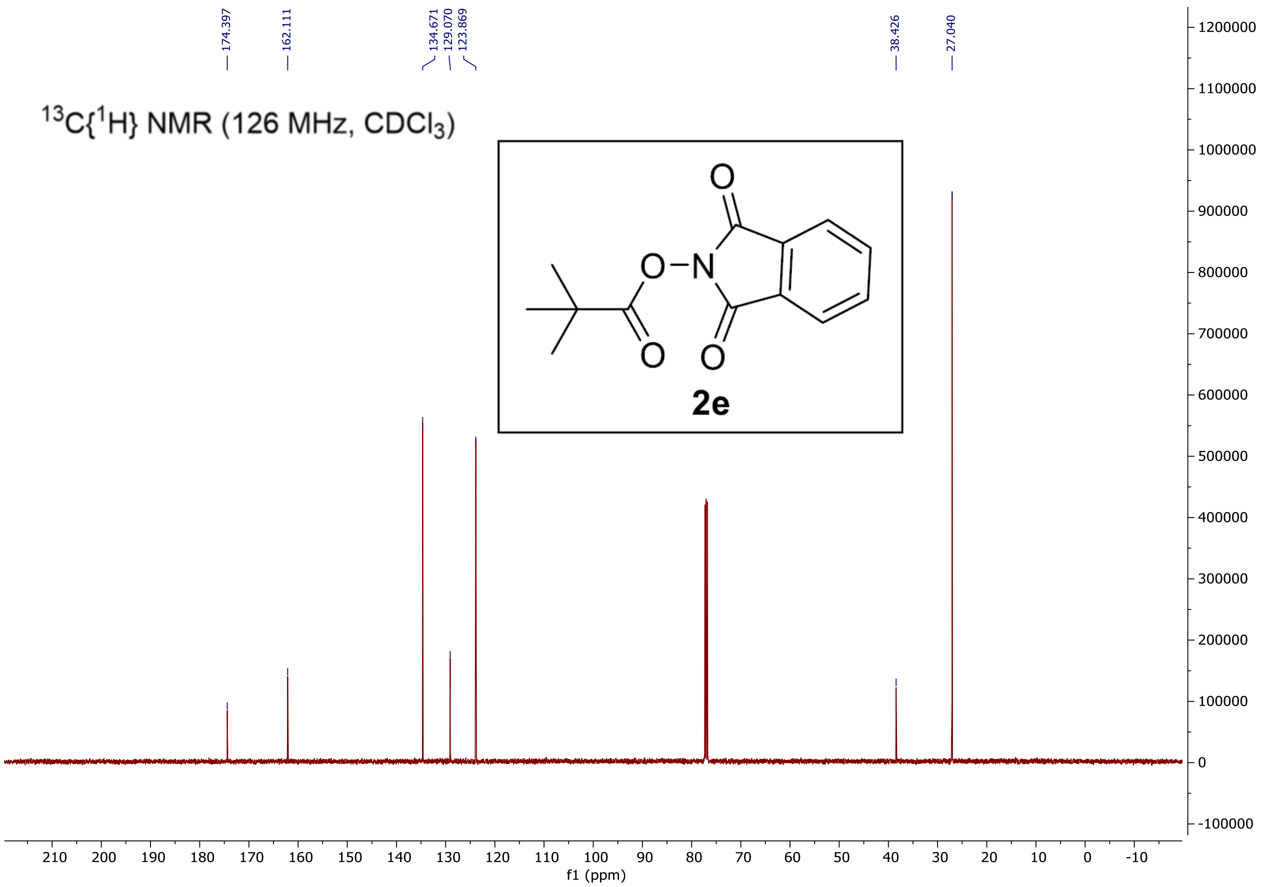

174.397
162.111
134.671
129.070
123.869
38.426
27.040
^{13}C{^{1}H} NMR (126 MHz, CDCl$_3$)
O
O-N
O
O
2e
f1 (ppm)

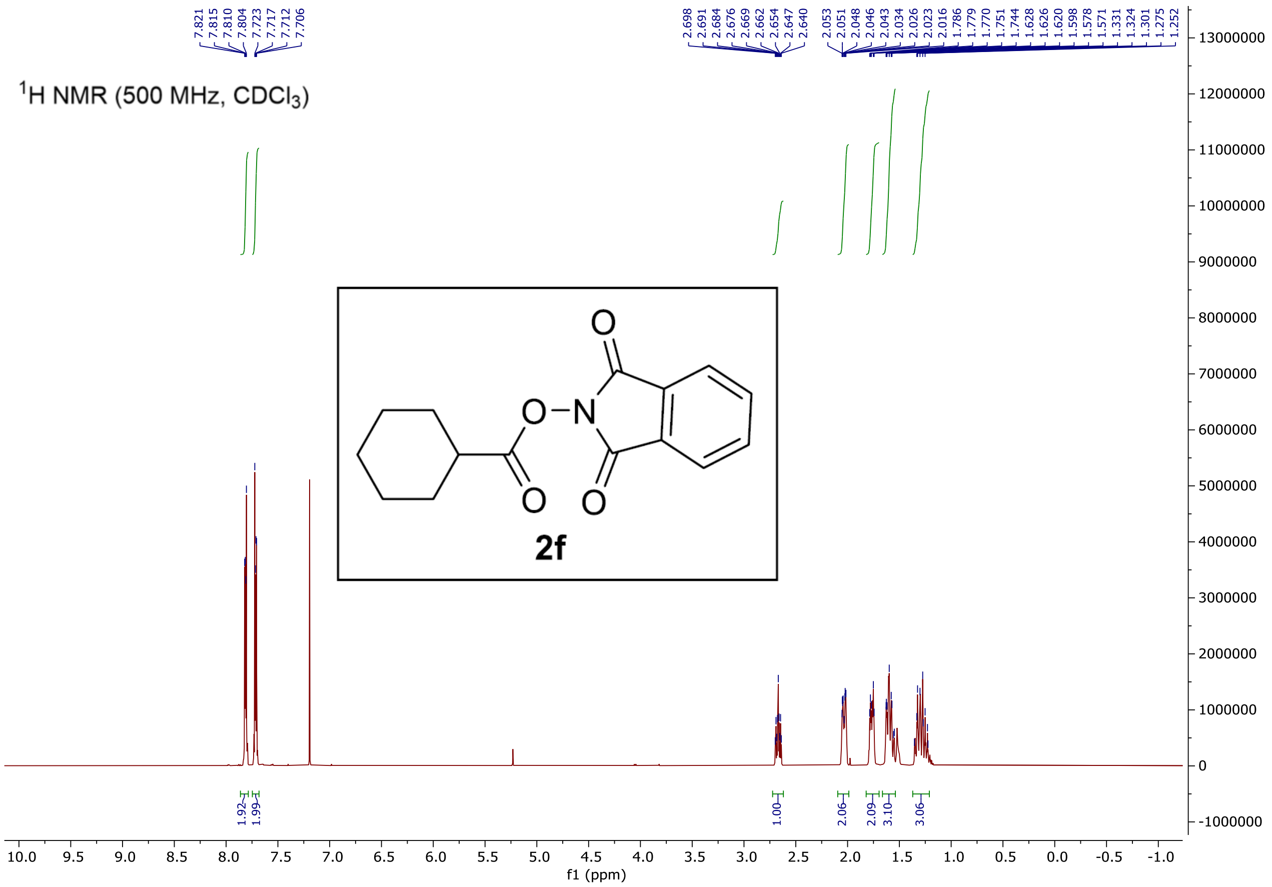
1H NMR (500 MHz, CDCl3)
2f
7.821
7.815
7.810
7.804
7.723
7.717
7.712
7.706
2.698
2.691
2.684
2.676
2.669
2.662
2.654
2.647
2.640
2.053
2.051
2.048
2.046
2.043
2.034
2.026
2.023
2.016
1.786
1.779
1.770
1.751
1.744
1.628
1.626
1.620
1.598
1.578
1.571
1.331
1.324
1.301
1.275
1.252
1.92
1.99
1.00
2.06
2.09
3.10
3.06
f1 (ppm)

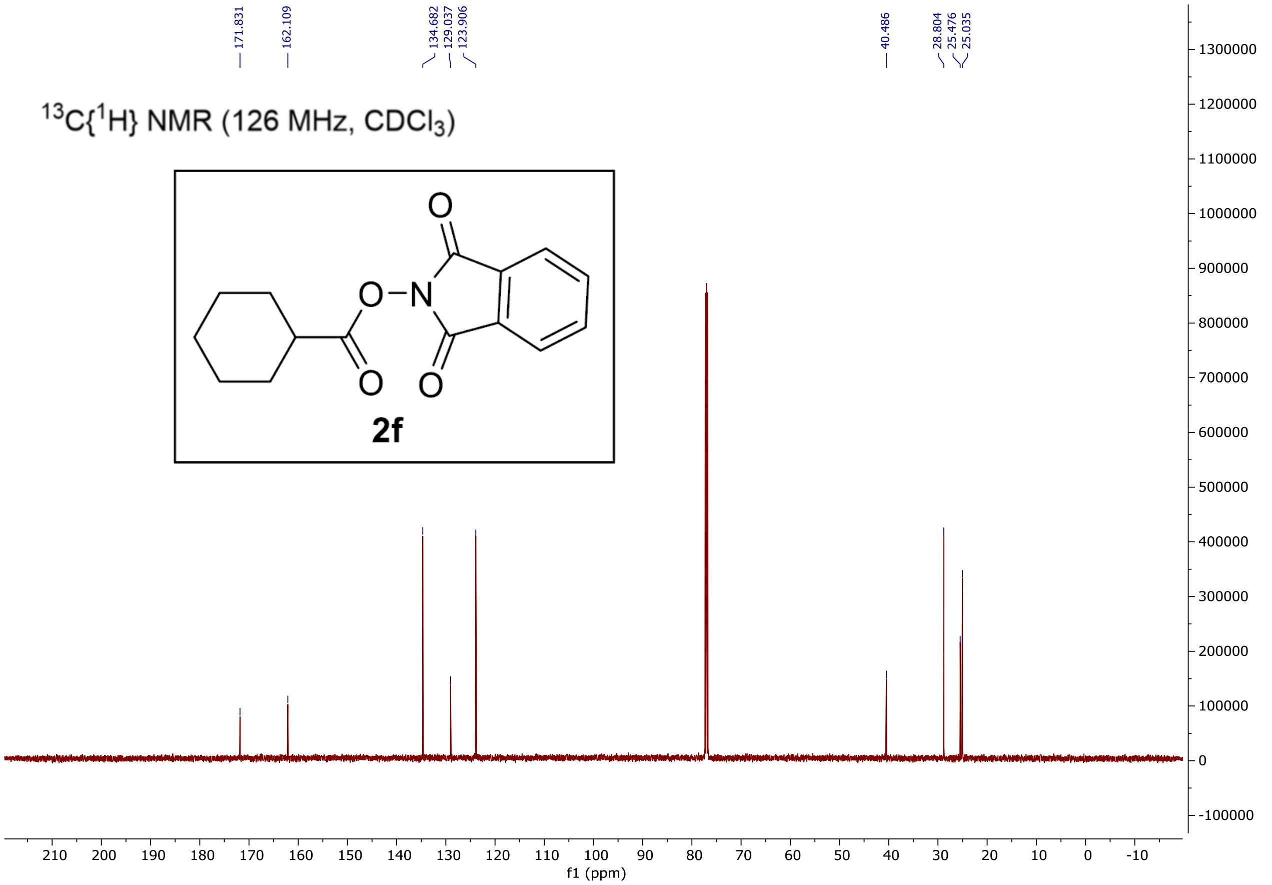
13C{1H} NMR (126 MHz, CDCl3)
2f
171.831
162.109
134.682
129.037
123.906
40.486
28.804
25.476
25.035
f1 (ppm)

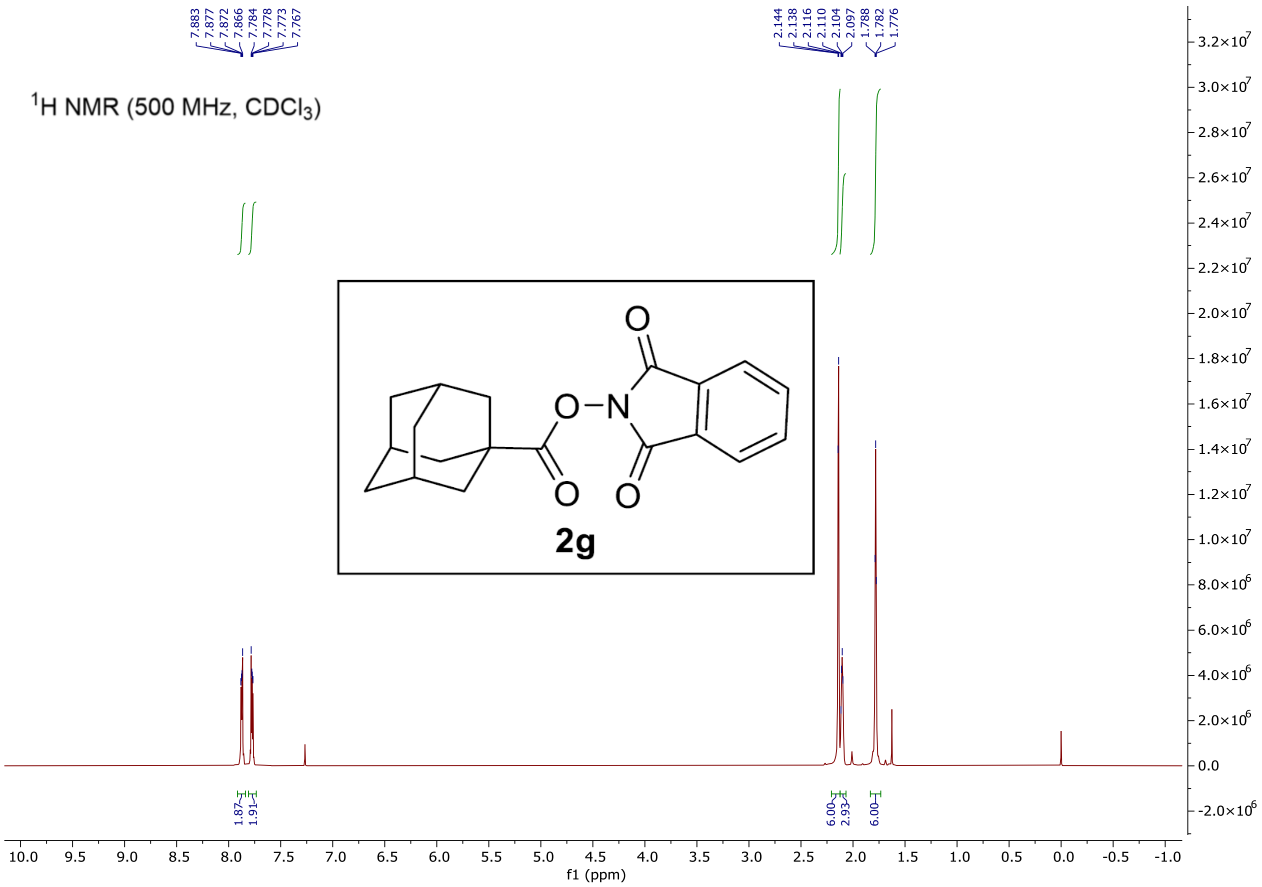
^{1}H NMR (500 MHz, CDCl$_3$)
7.883 7.877 7.872 7.866 7.784 7.778 7.773 7.767
2.144 2.138 2.116 2.110 2.104 2.097 1.788 1.782 1.776
1.87 1.91 6.00 2.93 6.00
O N O O O
2g
f1 (ppm)

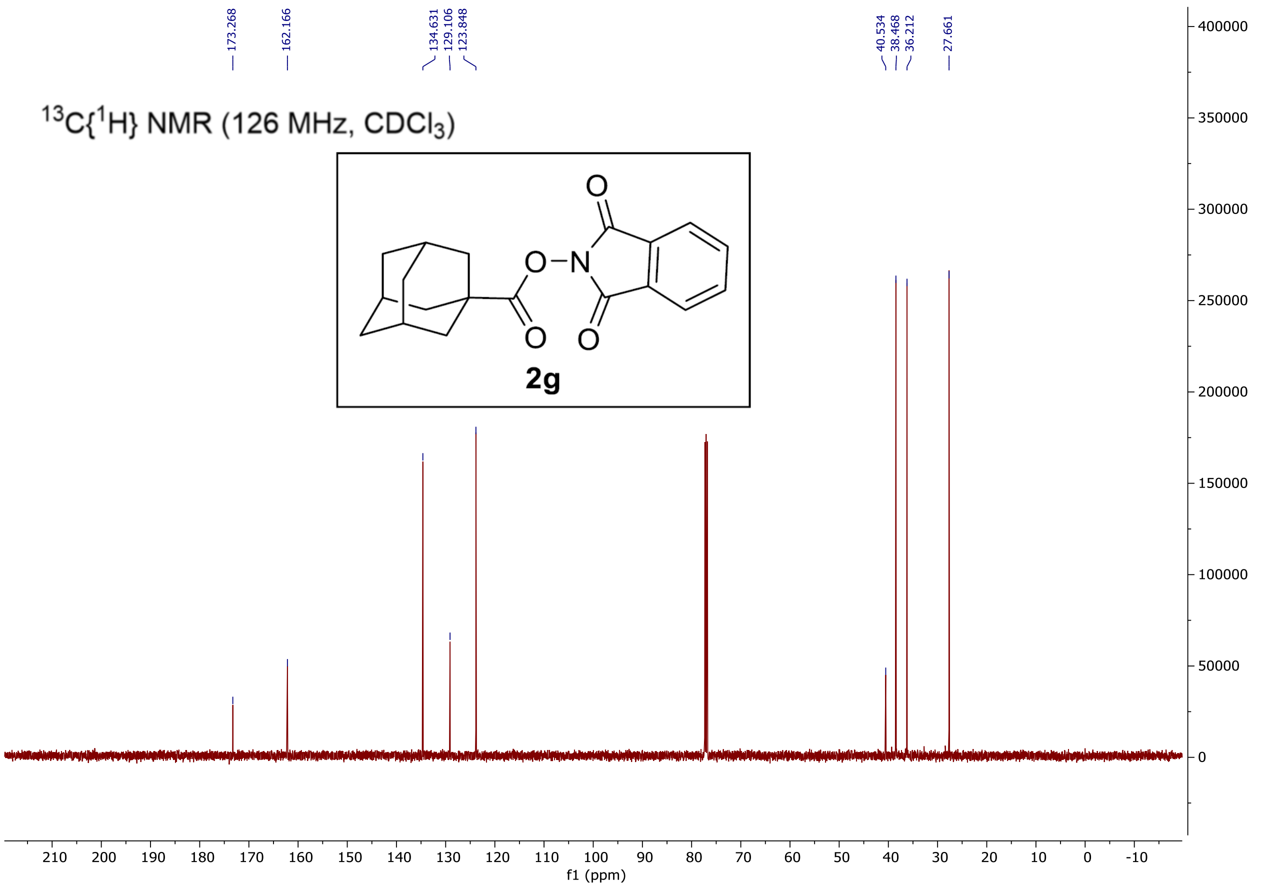
^{13}C{^{1}H} NMR (126 MHz, CDCl$_3$)
173.268 162.166 134.631 129.106 123.848 40.534 38.468 36.212 27.661
O N O O O
2g
f1 (ppm)

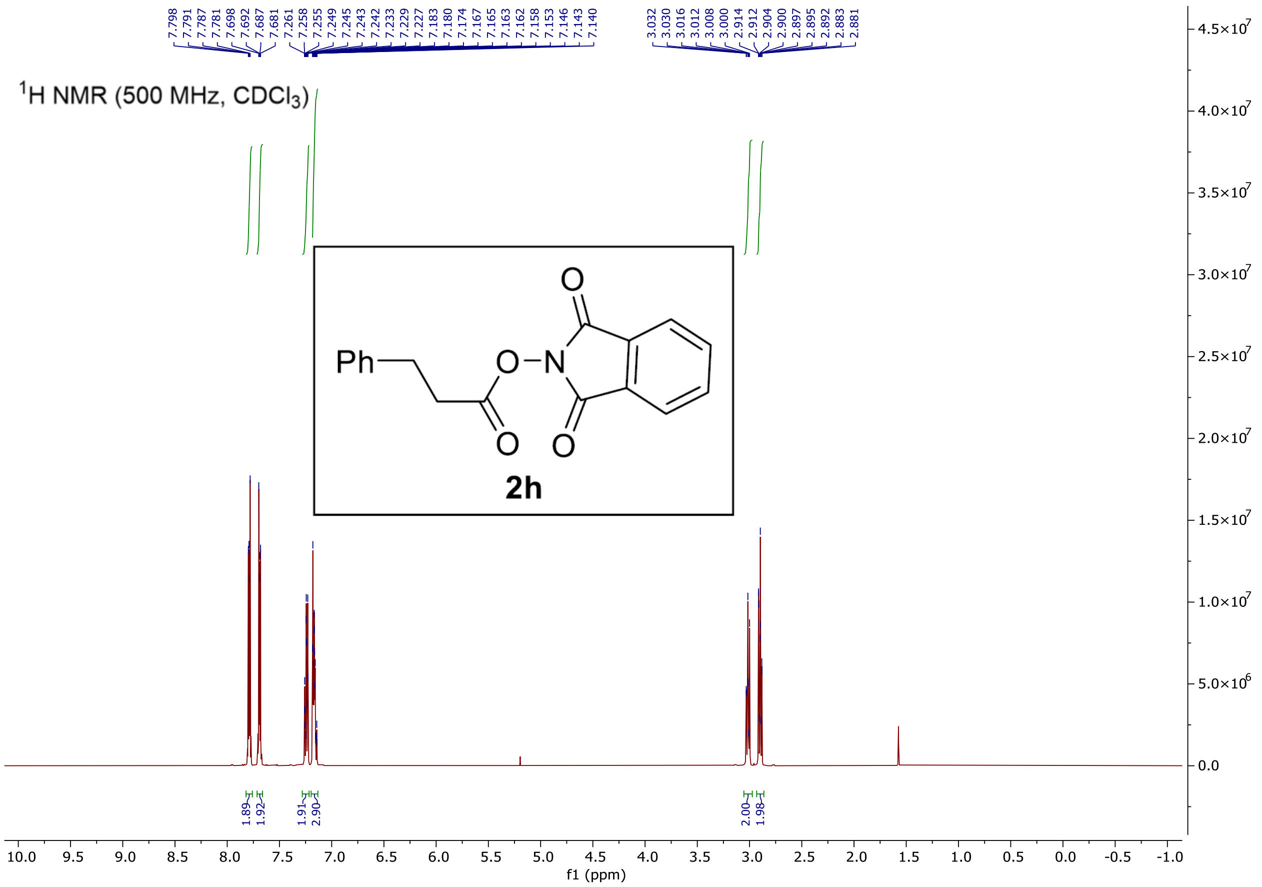

^{1}H NMR (500 MHz, CDCl$_3$)
2h
f1 (ppm)

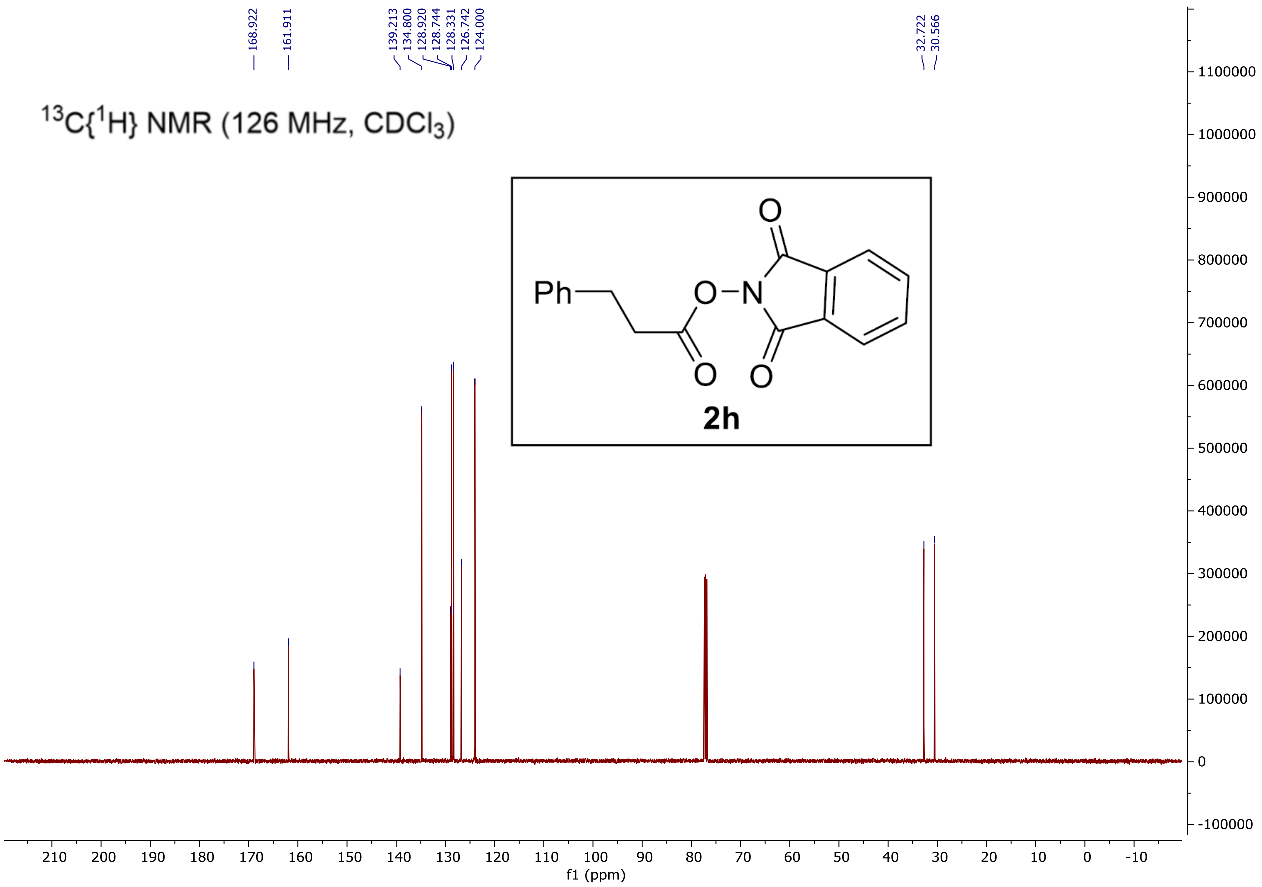

^{13}C{^{1}H} NMR (126 MHz, CDCl$_3$)
2h
f1 (ppm)

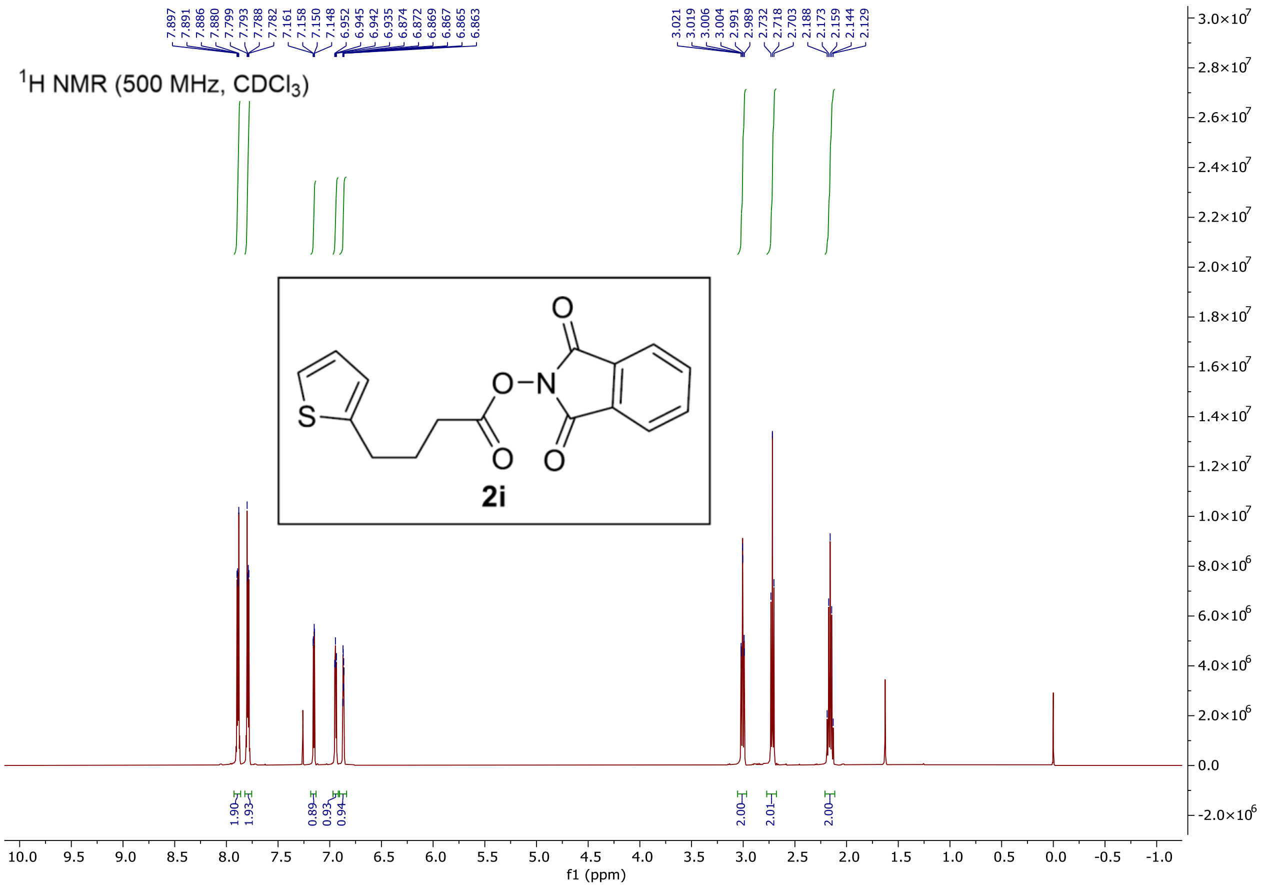

7.897
7.891
7.886
7.880
7.799
7.793
7.788
7.782
7.161
7.158
7.150
7.148
6.952
6.945
6.942
6.935
6.874
6.872
6.869
6.867
6.865
6.863
3.021
3.019
3.006
3.004
2.991
2.989
2.732
2.718
2.703
2.188
2.173
2.159
2.144
2.129
^{1}H NMR (500 MHz, $CDCl_3$)
O
O-N
S
O
O
2i
1.90
1.93
0.89
0.93
0.94
2.00
2.01
2.00
f1 (ppm)

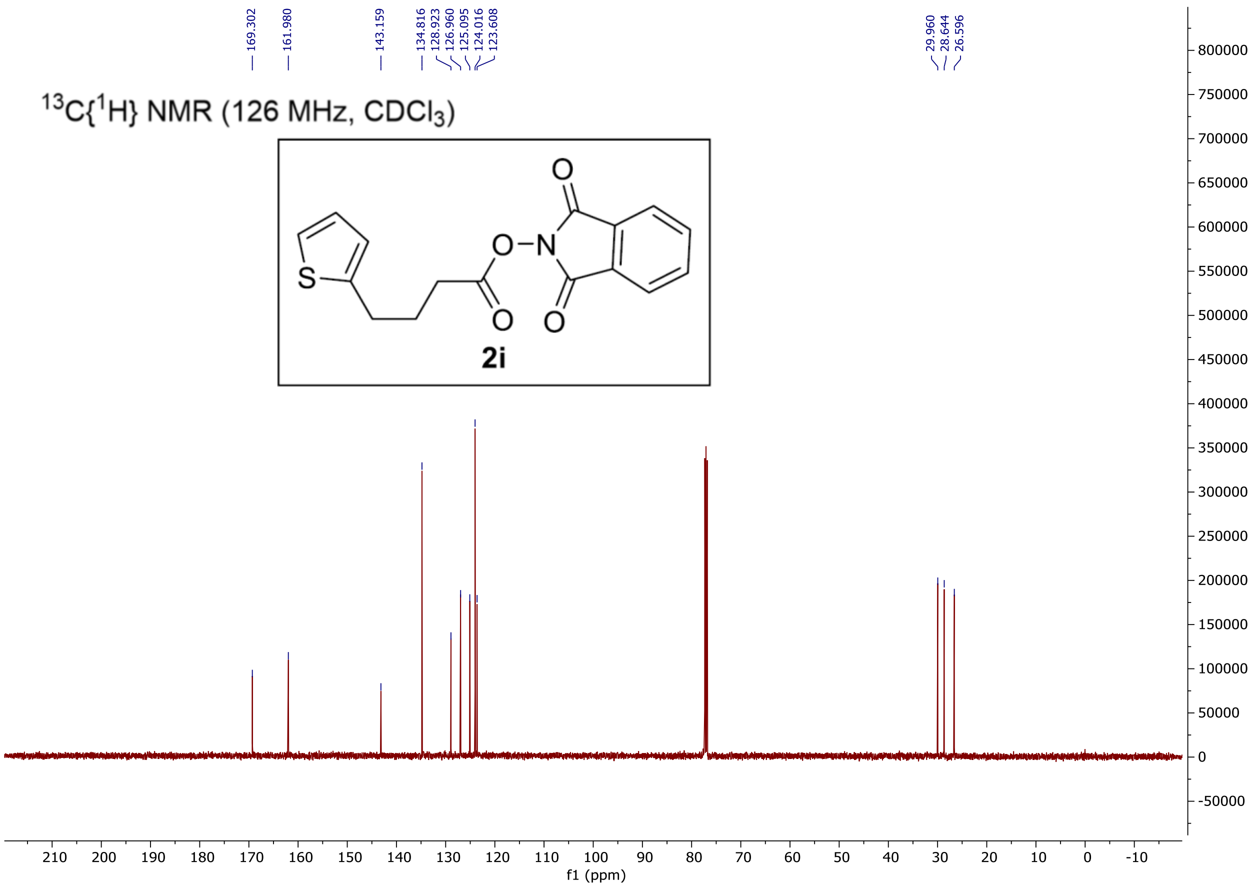

169.302
161.980
143.159
134.816
128.923
126.960
125.095
124.016
123.608
29.960
28.644
26.596
$^{13}C\{^{1}H\}$ NMR (126 MHz, $CDCl_3$)
O
O-N
S
O
O
2i
f1 (ppm)

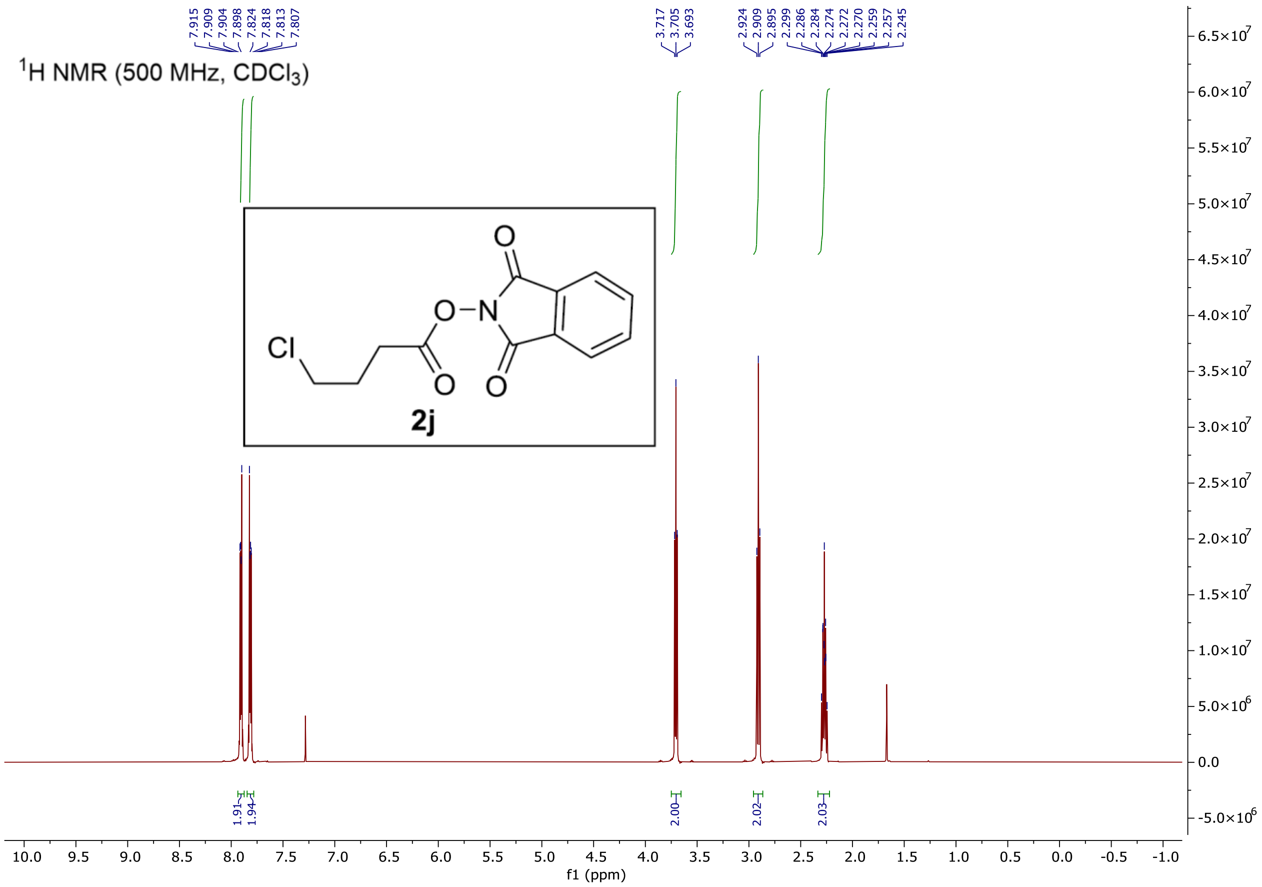

^{1}H NMR (500 MHz, CDCl$_3$)
2j
f1 (ppm)

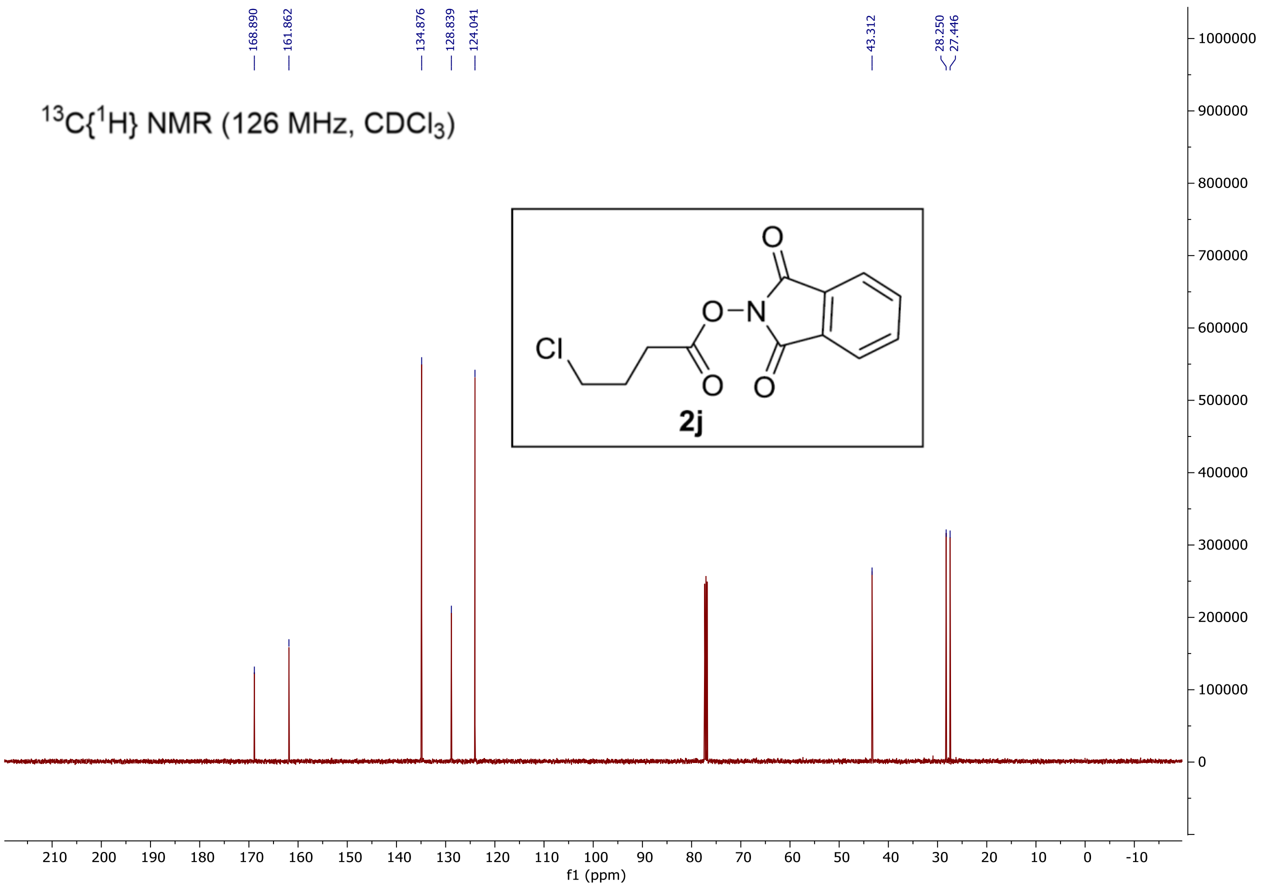

^{13}C{^{1}H} NMR (126 MHz, CDCl$_3$)
2j
f1 (ppm)

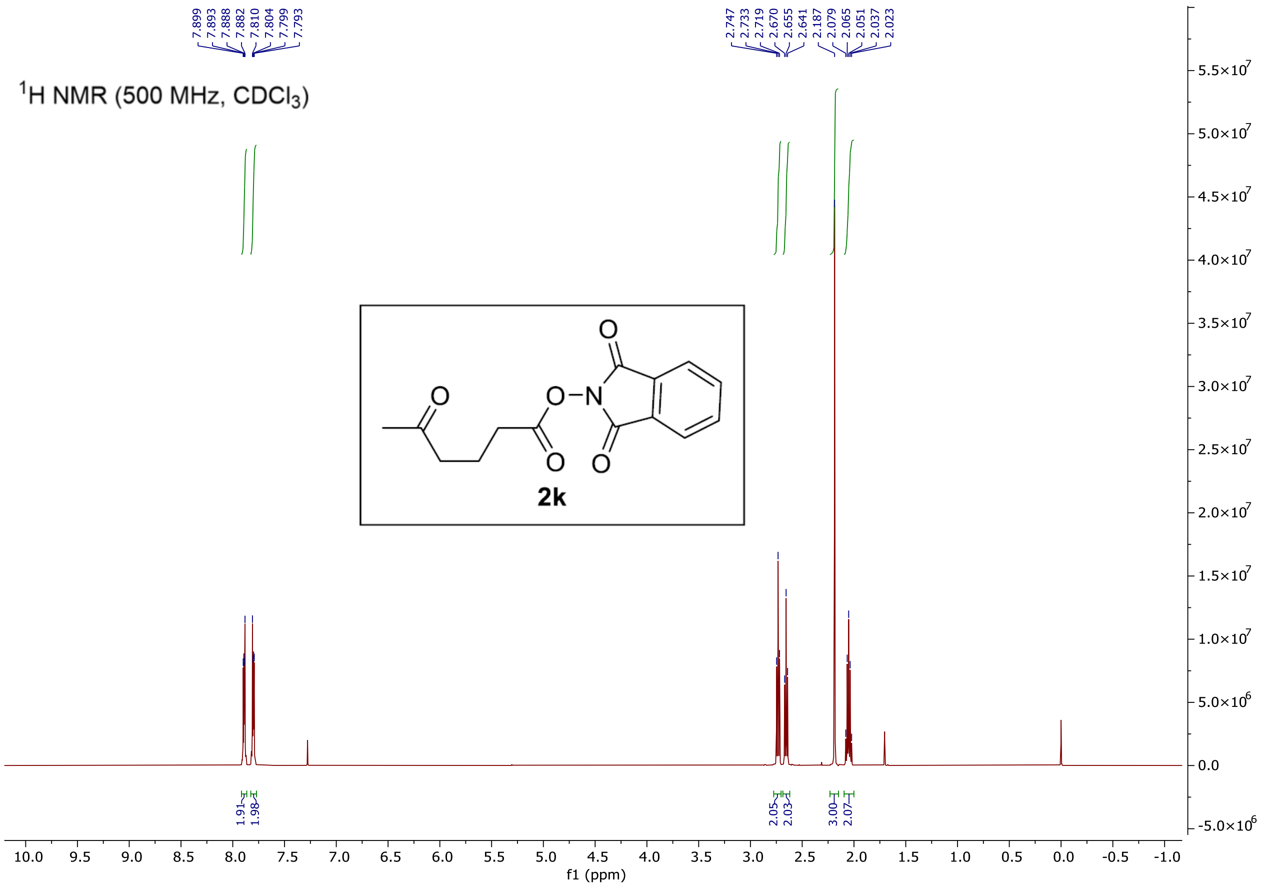

7.899
7.893
7.888
7.882
7.810
7.804
7.799
7.793
2.747
2.733
2.719
2.670
2.655
2.641
2.187
2.079
2.065
2.051
2.037
2.023
^{1}H NMR (500 MHz, $CDCl_3$)
O
O–N
O
O
O
2k
1.91
1.98
2.05
2.03
3.00
2.07
f1 (ppm)

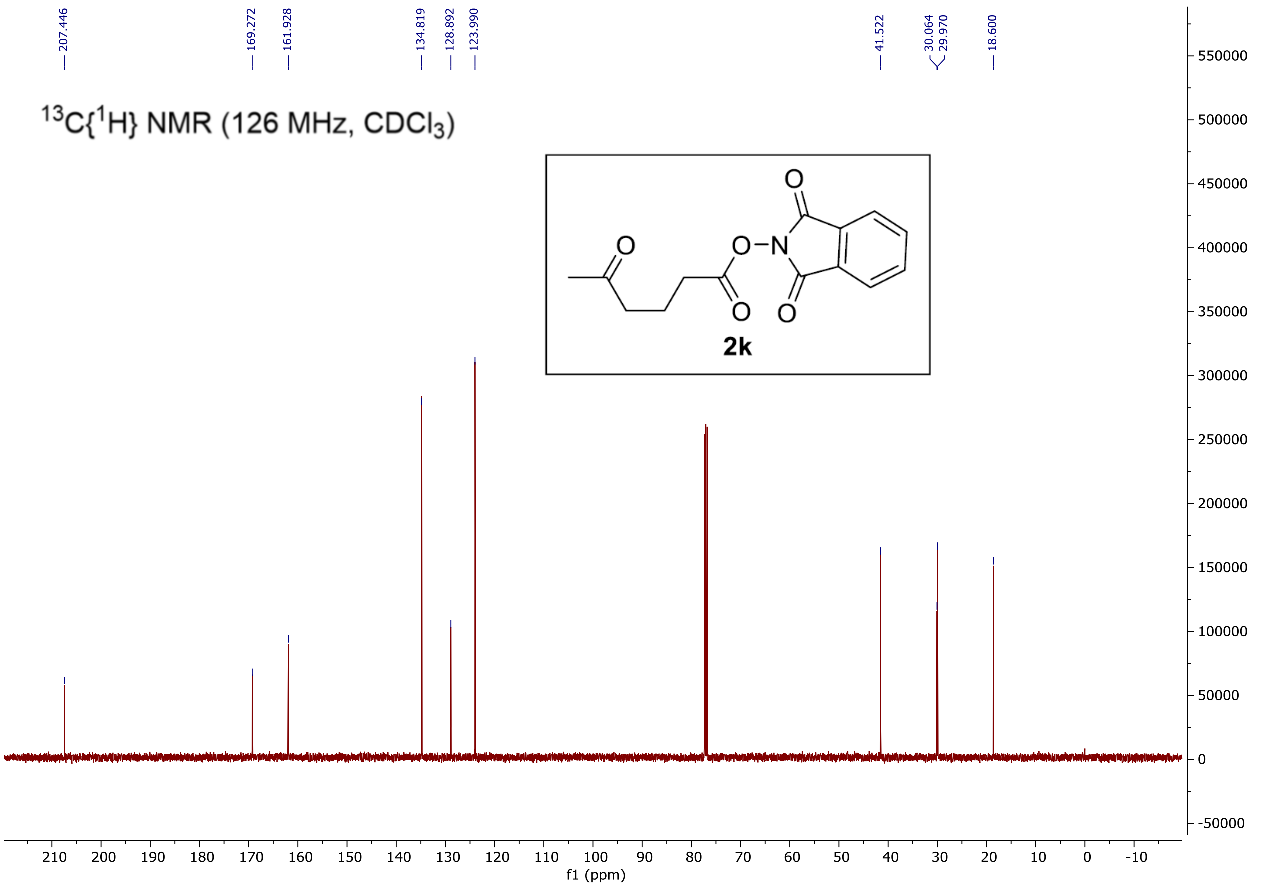

207.446
169.272
161.928
134.819
128.892
123.990
41.522
30.064
29.970
18.600
$^{13}C\{^{1}H\}$ NMR (126 MHz, $CDCl_3$)
O
O–N
O
O
O
2k
f1 (ppm)

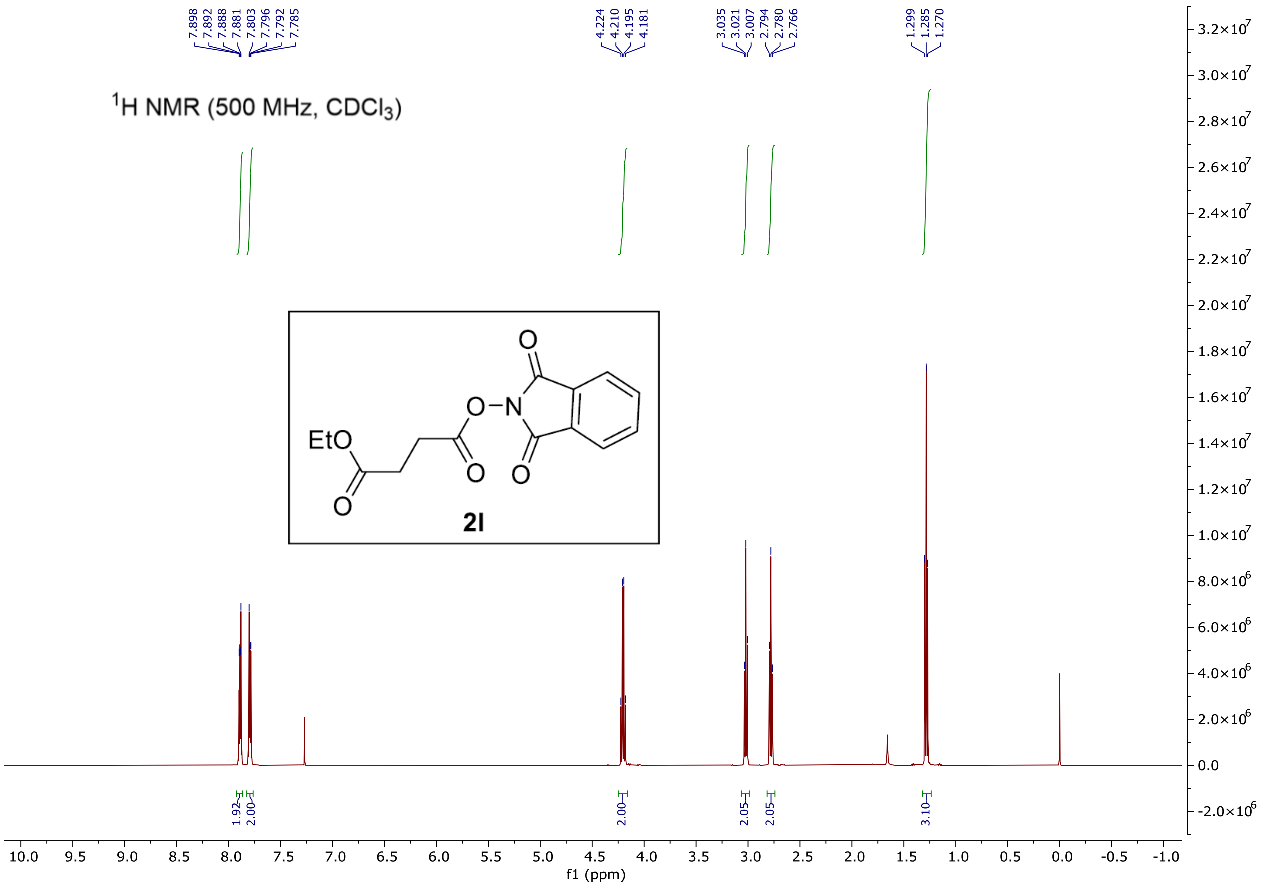

7.898
7.892
7.888
7.881
7.803
7.796
7.792
7.785
4.224
4.210
4.195
4.181
3.035
3.021
3.007
2.794
2.780
2.766
1.299
1.285
1.270
^{1}H NMR (500 MHz, CDCl$_3$)
EtO
O
O
N
O
O
O
2l
1.92
2.00
2.00
2.05
2.05
3.10
f1 (ppm)

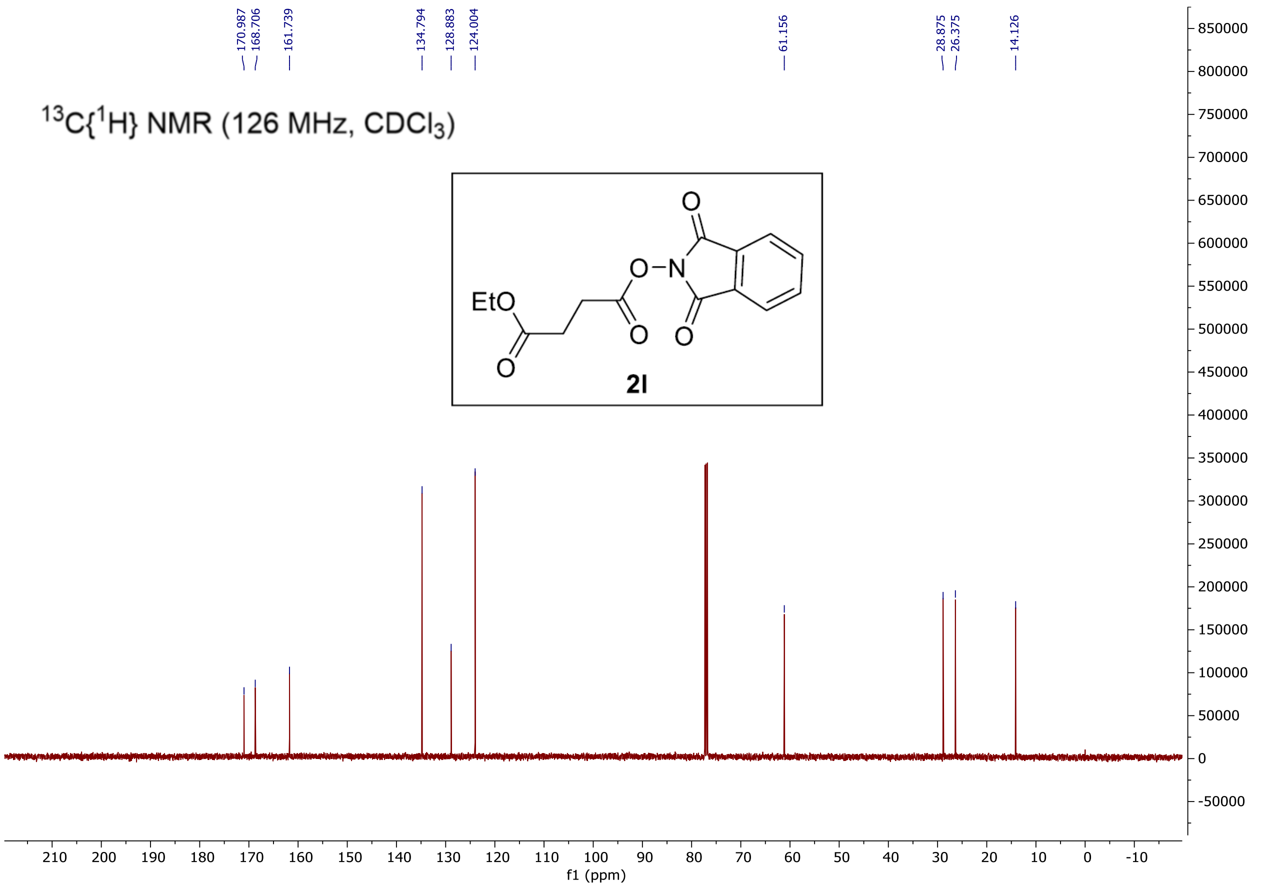

170.987
168.706
161.739
134.794
128.883
124.004
61.156
28.875
26.375
14.126
^{13}C{^{1}H} NMR (126 MHz, CDCl$_3$)
EtO
O
O
N
O
O
O
2l
f1 (ppm)

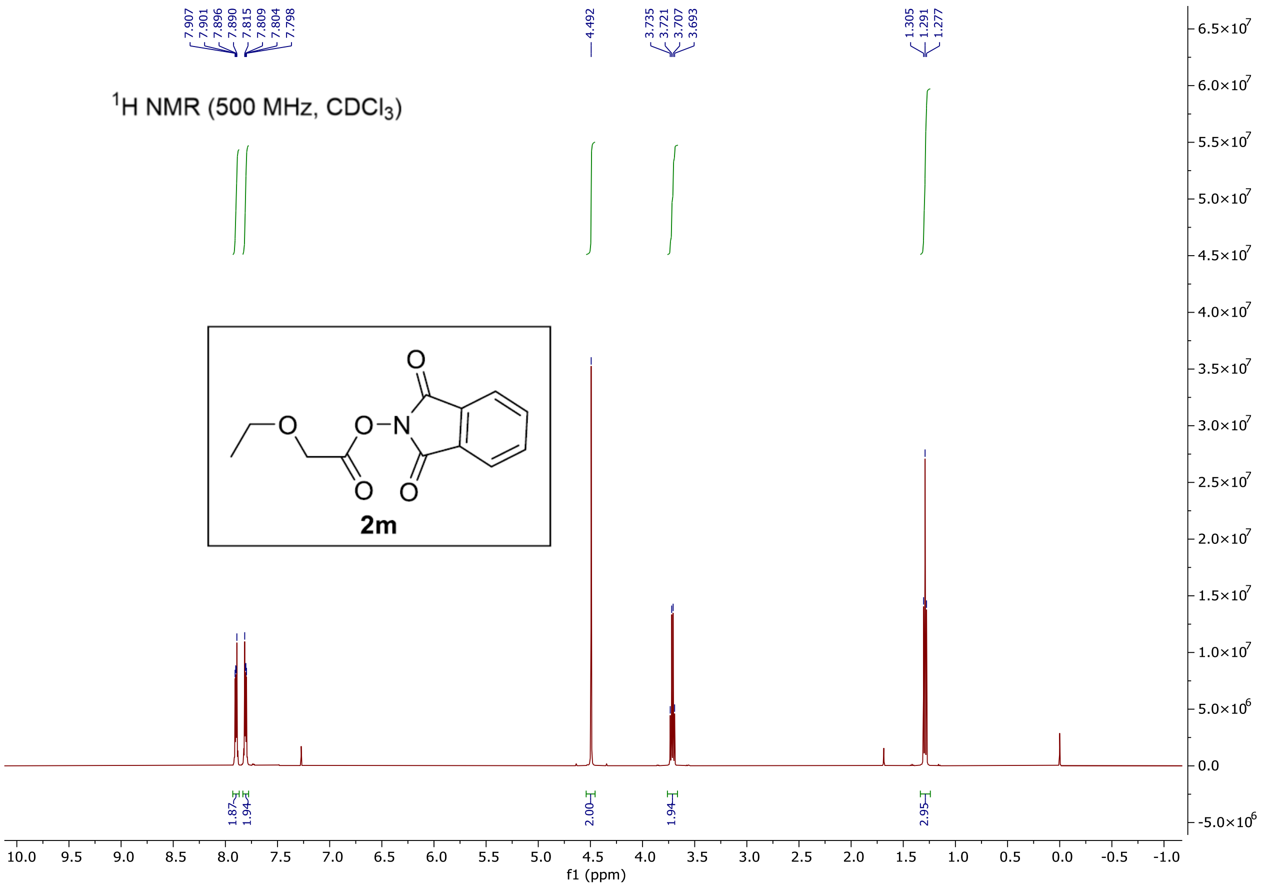

7.907
7.901
7.896
7.890
7.815
7.809
7.804
7.798
4.492
3.735
3.721
3.707
3.693
1.305
1.291
1.277
1H NMR (500 MHz, CDCl3)
O
O
O
N
O
O
2m
1.87
1.94
2.00
1.94
2.95
f1 (ppm)

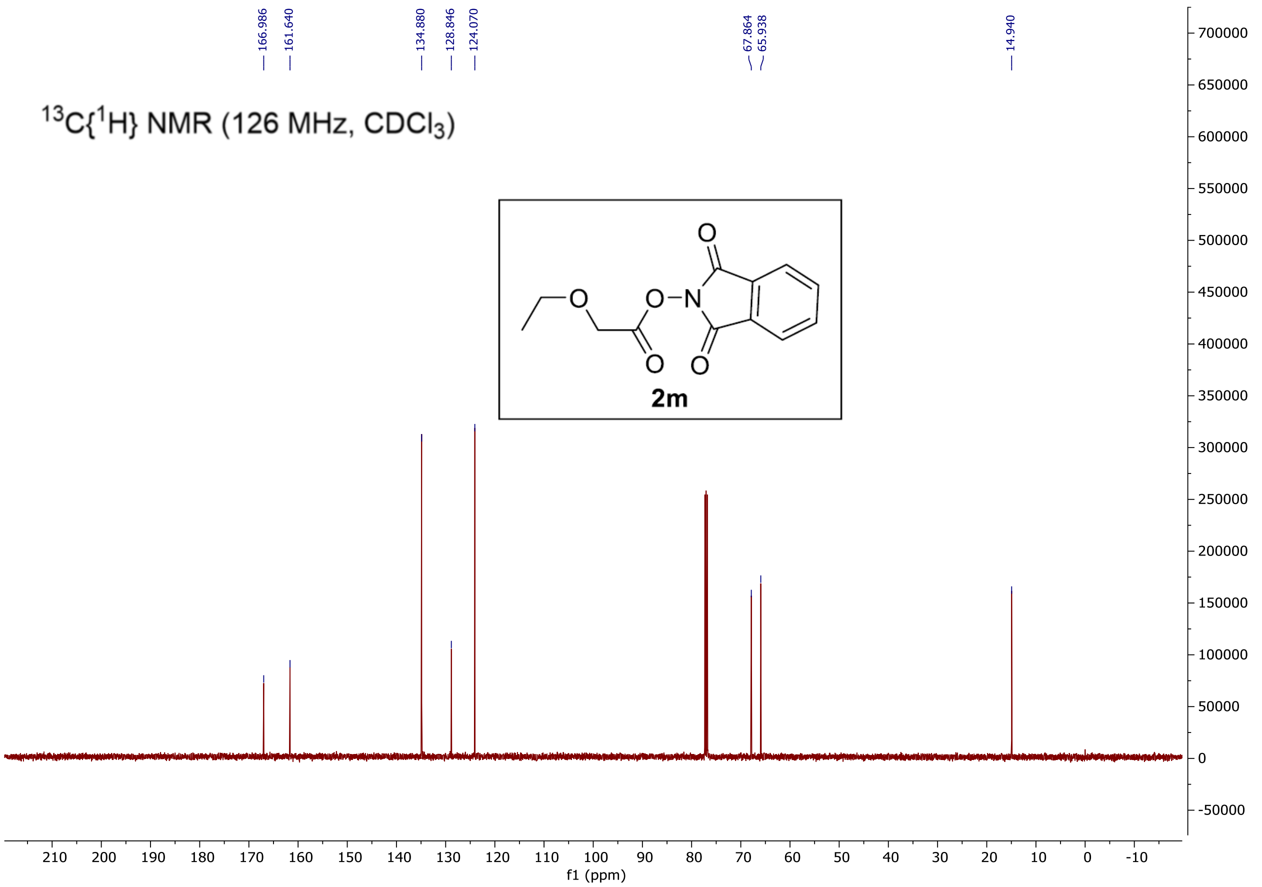

166.986
161.640
134.880
128.846
124.070
67.864
65.938
14.940
13C{1H} NMR (126 MHz, CDCl3)
O
O
O
N
O
O
2m
f1 (ppm)

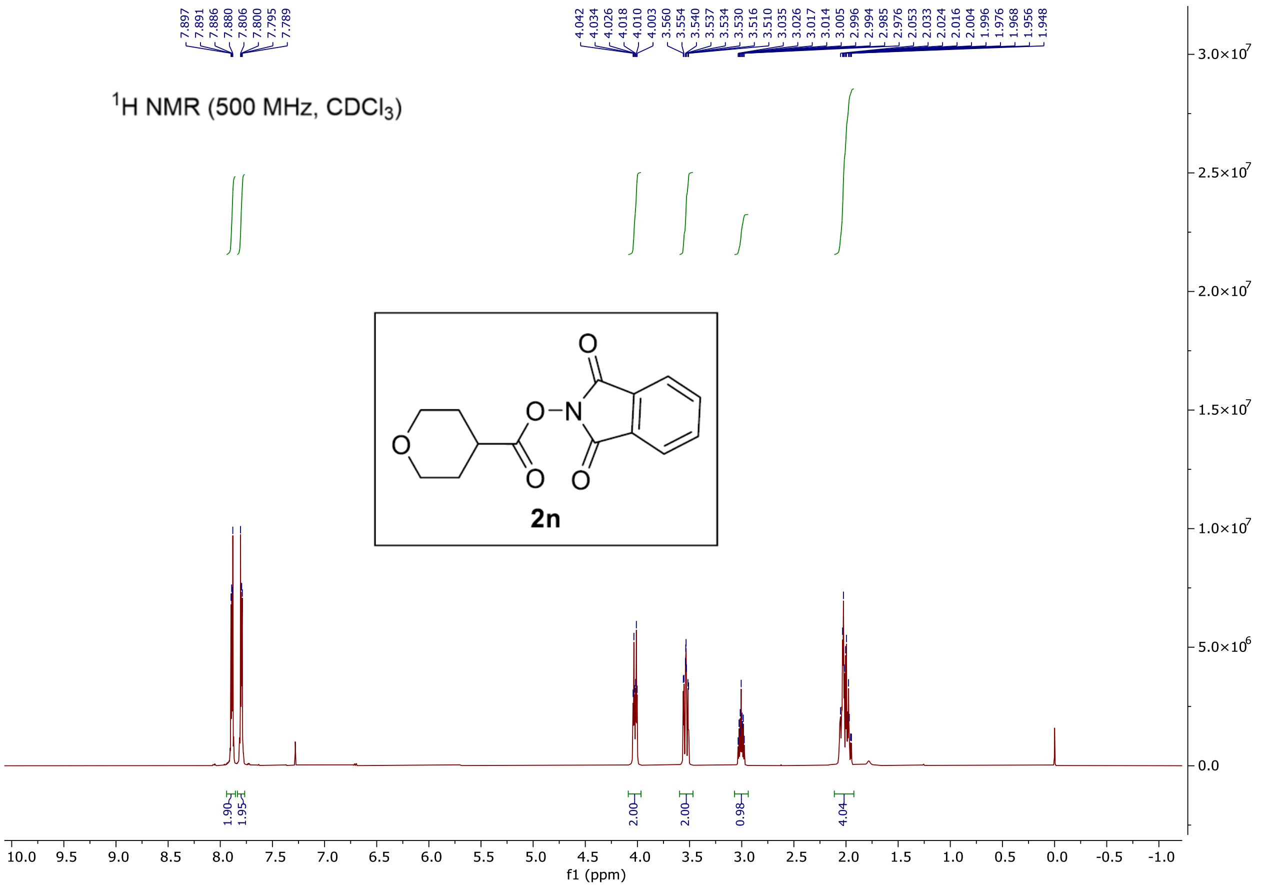
7.897
7.891
7.886
7.880
7.806
7.800
7.795
7.789
4.042
4.034
4.026
4.018
4.010
4.003
3.560
3.554
3.540
3.537
3.534
3.530
3.516
3.510
3.035
3.026
3.017
3.014
3.005
2.996
2.994
2.985
2.976
2.053
2.033
2.024
2.016
2.004
1.996
1.976
1.968
1.956
1.948
1H NMR (500 MHz, CDCl3)
O
O–N
O
O
O
2n
1.90
1.95
2.00
2.00
0.98
4.04
f1 (ppm)

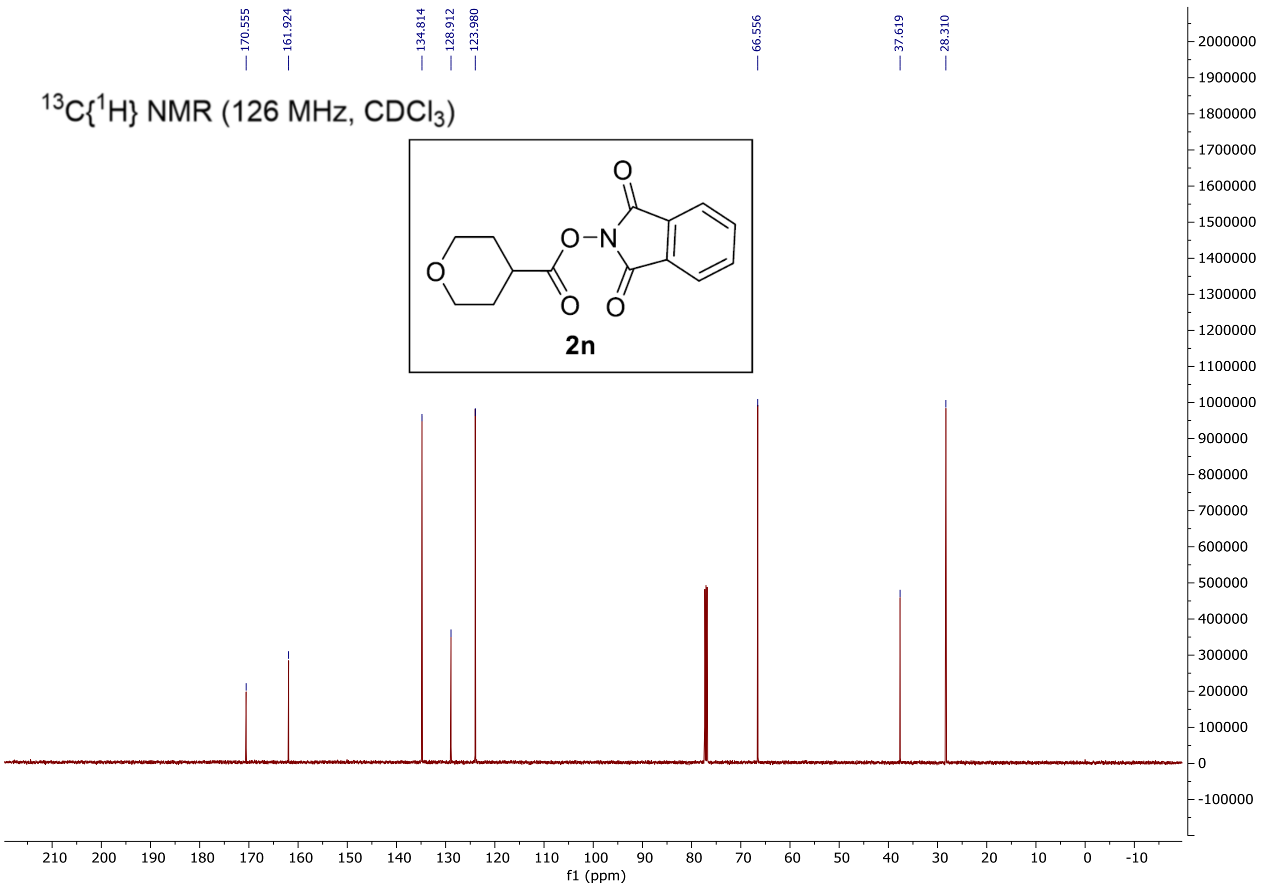
170.555
161.924
134.814
128.912
123.980
66.556
37.619
28.310
13C{1H} NMR (126 MHz, CDCl3)
O
O–N
O
O
O
2n
f1 (ppm)

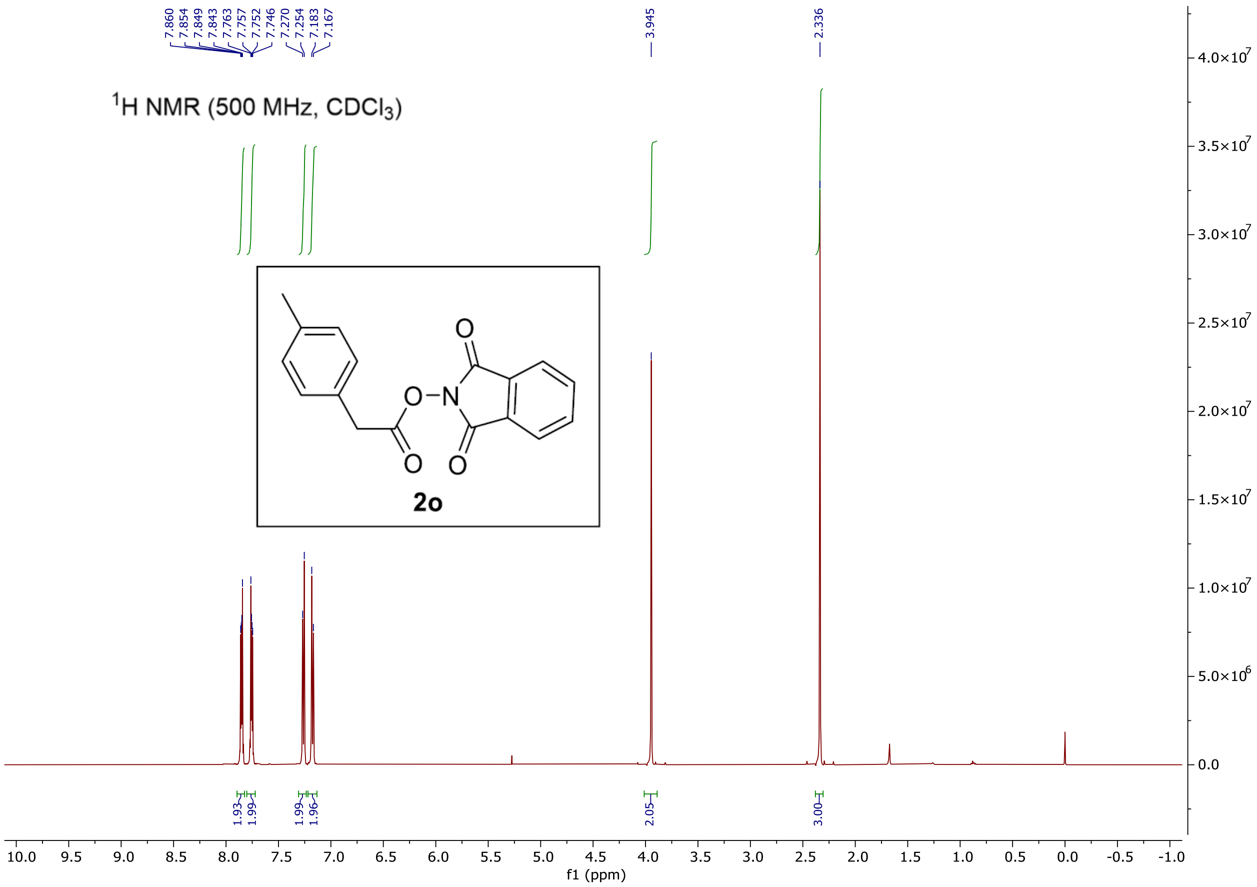

7.860
7.854
7.849
7.843
7.763
7.757
7.752
7.746
7.270
7.254
7.183
7.167
3.945
2.336
^{1}H NMR (500 MHz, $CDCl_3$)
O
O-N
O
O
2o
1.93
1.99
1.99
1.96
2.05
3.00
f1 (ppm)

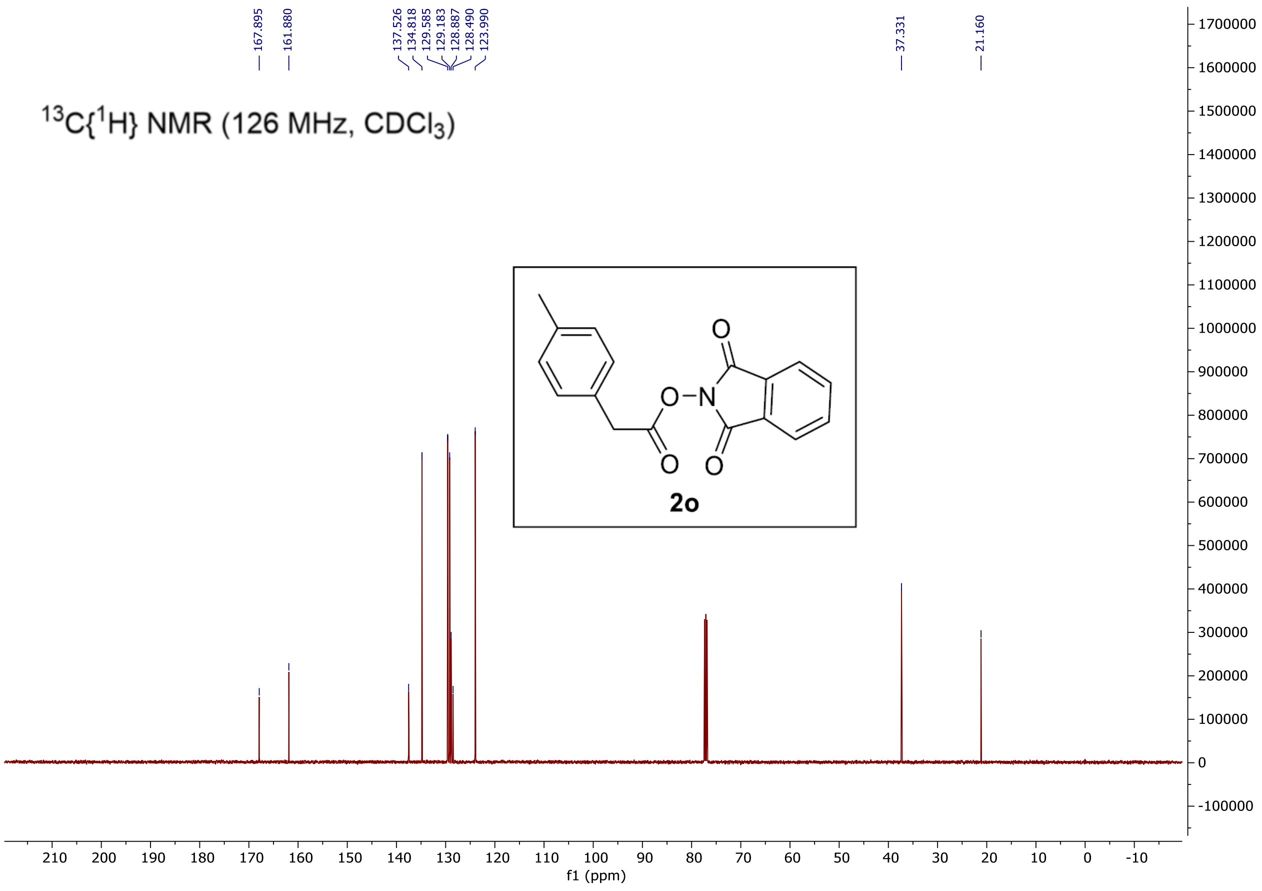

167.895
161.880
137.526
134.818
129.585
129.183
128.887
128.490
123.990
37.331
21.160
^{13}C{^{1}H} NMR (126 MHz, $CDCl_3$)
O
O-N
O
O
2o
f1 (ppm)

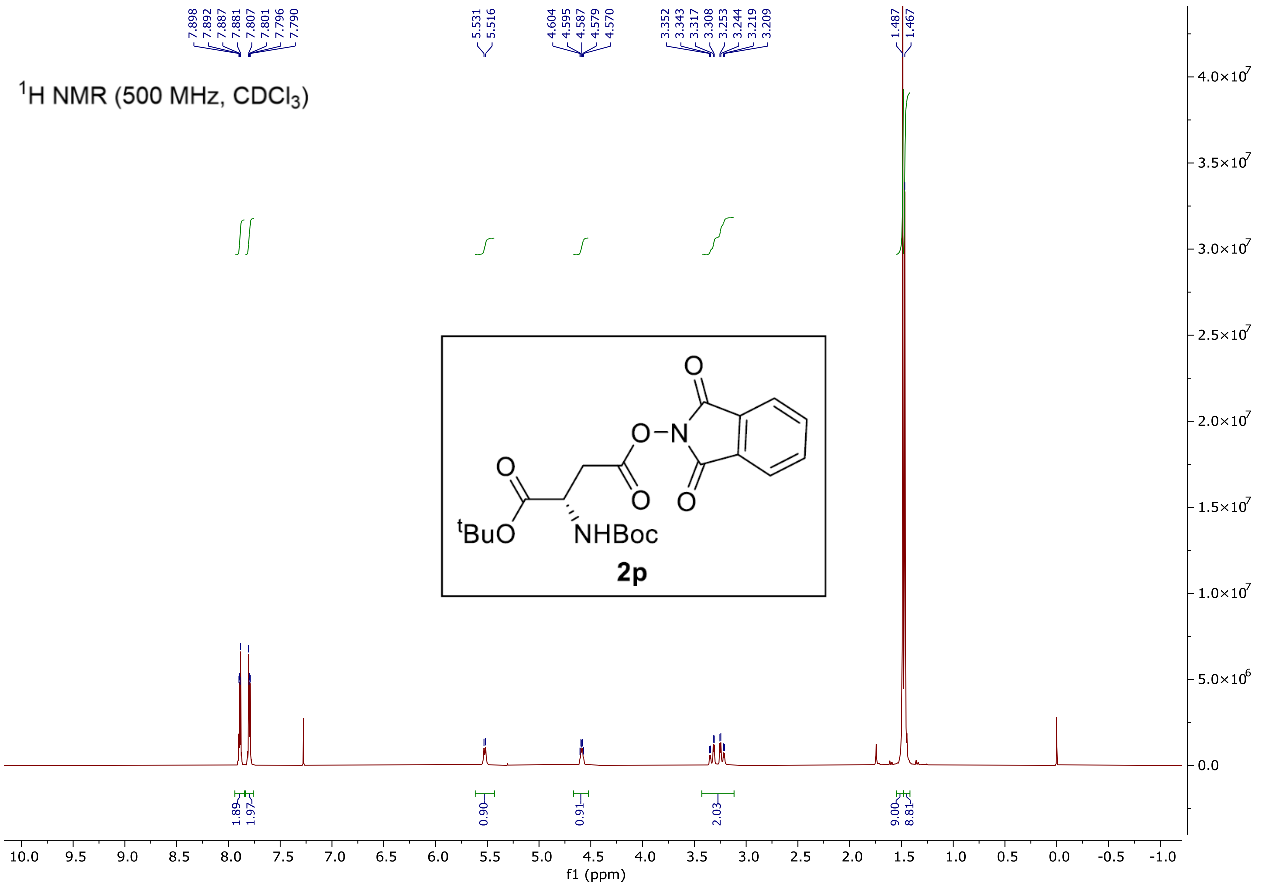

7.898
7.892
7.887
7.881
7.807
7.801
7.796
7.790
5.531
5.516
4.604
4.595
4.587
4.579
4.570
3.352
3.343
3.317
3.308
3.253
3.244
3.219
3.209
1.487
1.467
1H NMR (500 MHz, CDCl3)
tBuO
NHBoc
2p
1.89
1.97
0.90
0.91
2.03
9.00
8.81
f1 (ppm)

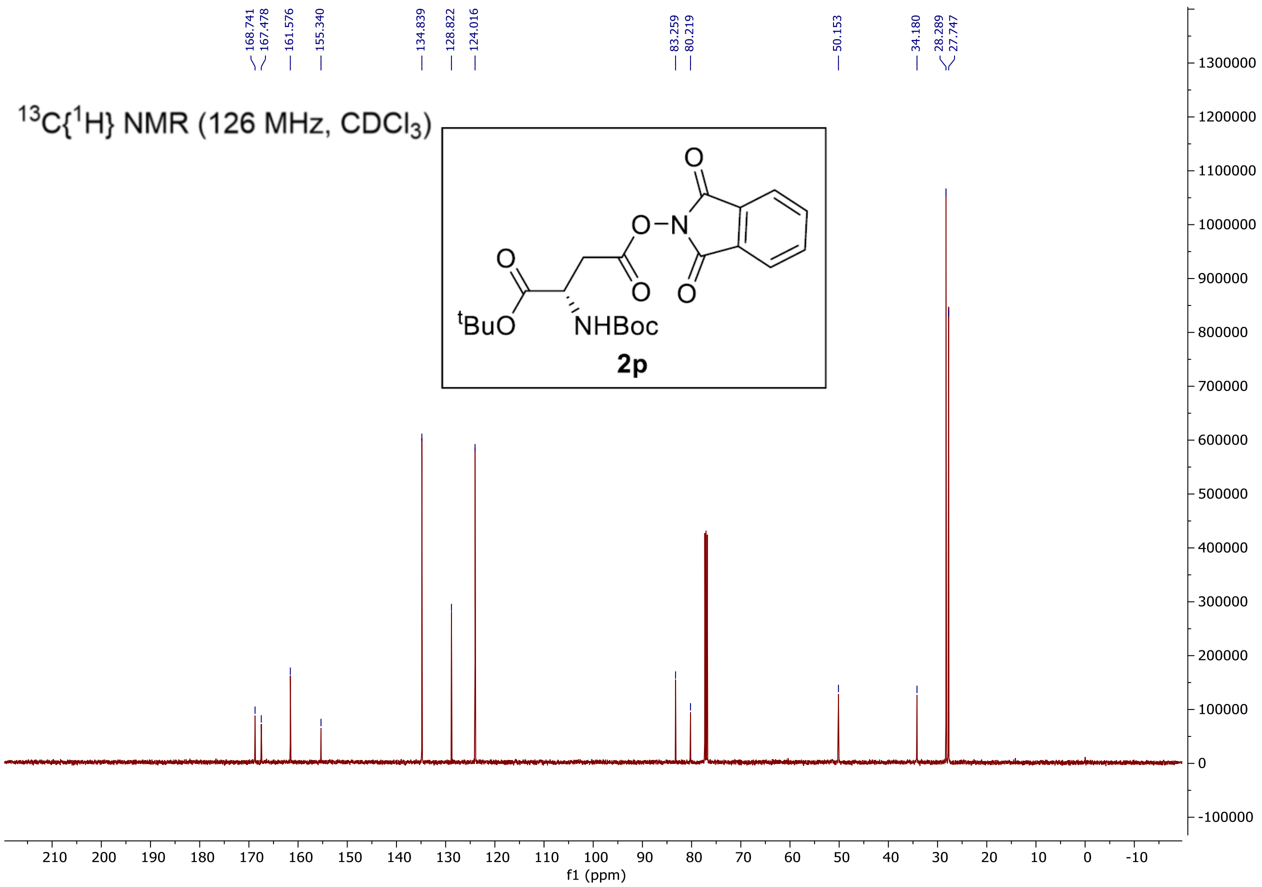

168.741
167.478
161.576
155.340
134.839
128.822
124.016
83.259
80.219
50.153
34.180
28.289
27.747
13C{1H} NMR (126 MHz, CDCl3)
tBuO
NHBoc
2p
f1 (ppm)

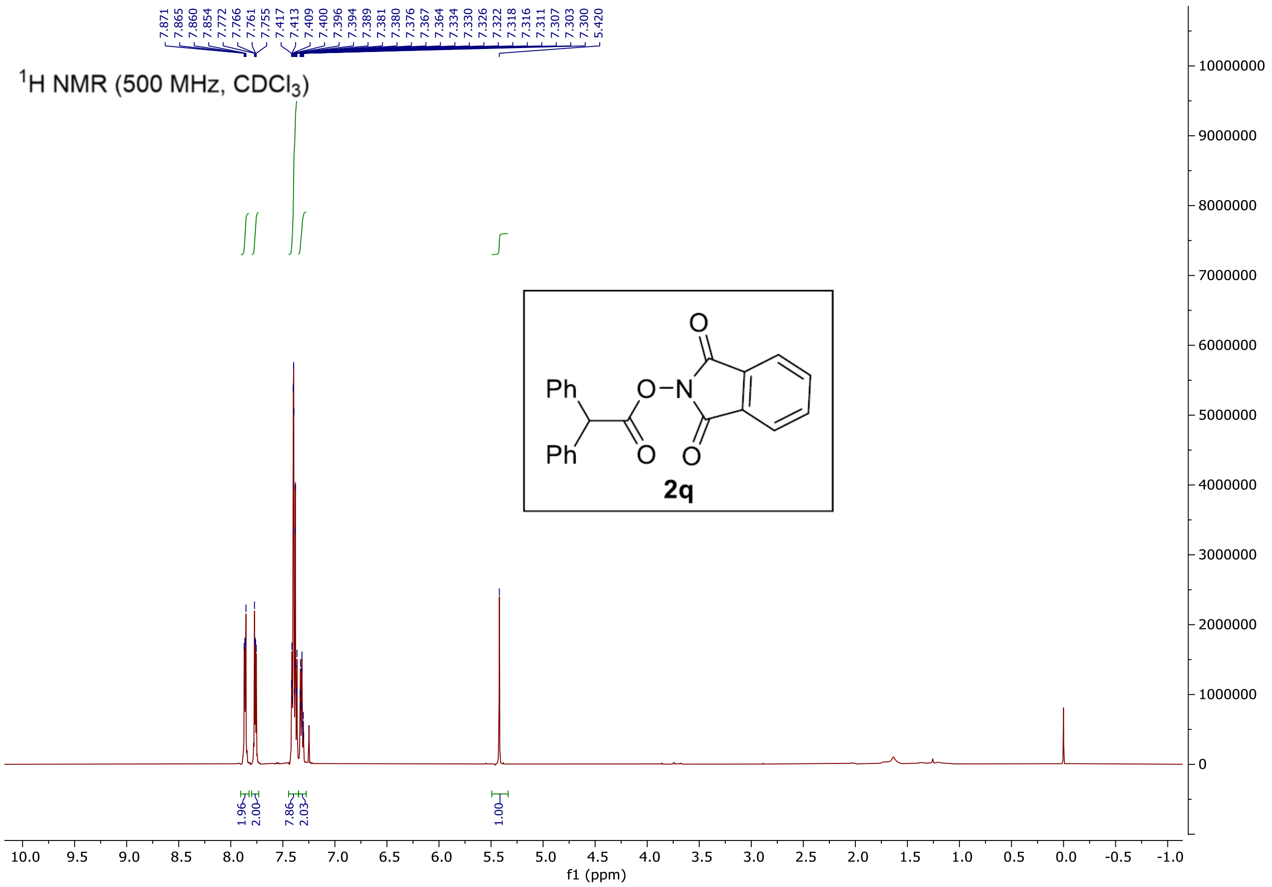
7.871
7.865
7.860
7.854
7.772
7.766
7.761
7.755
7.417
7.413
7.409
7.400
7.396
7.394
7.389
7.381
7.380
7.376
7.367
7.364
7.334
7.330
7.326
7.322
7.318
7.316
7.311
7.307
7.303
7.300
5.420
^{1}H NMR (500 MHz, CDCl$_3$)
Ph
Ph
O
O
O
O–N
2q
1.96
2.00
7.86
2.03
1.00
f1 (ppm)

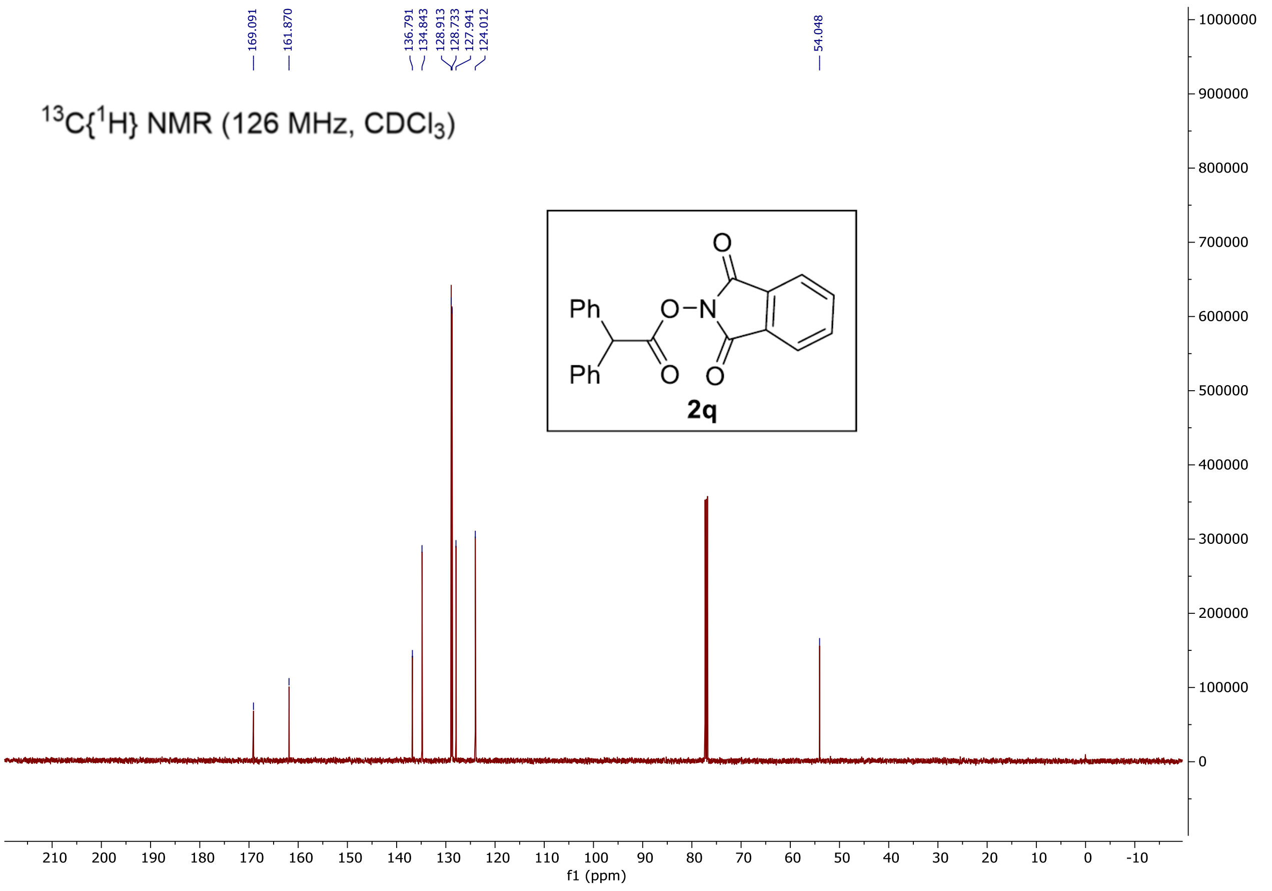
169.091
161.870
136.791
134.843
128.913
128.733
127.941
124.012
54.048
^{13}C{^{1}H} NMR (126 MHz, CDCl$_3$)
Ph
Ph
O
O
O
O–N
2q
f1 (ppm)

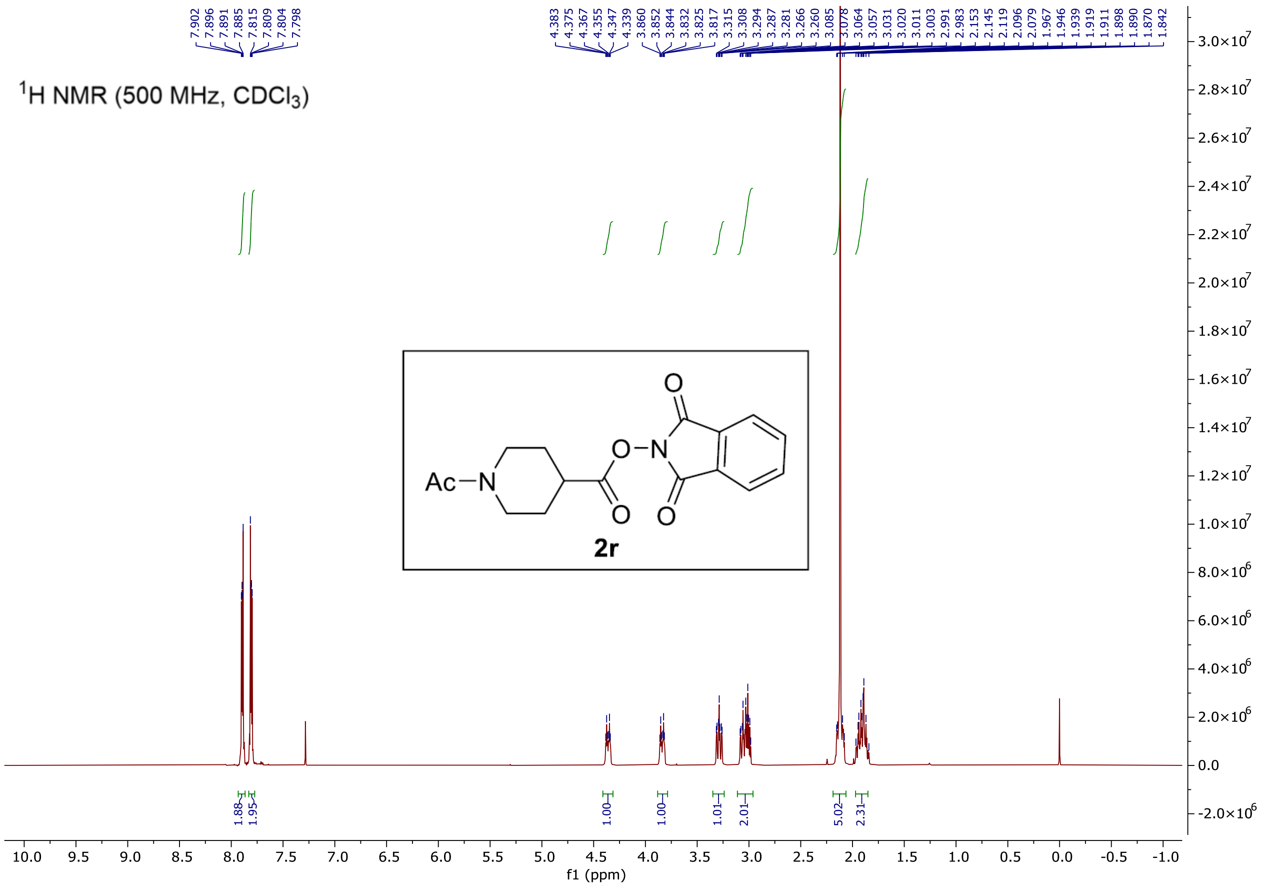

^{1}H NMR (500 MHz, CDCl$_3$)
Ac−N
O−N
O
2r
f1 (ppm)

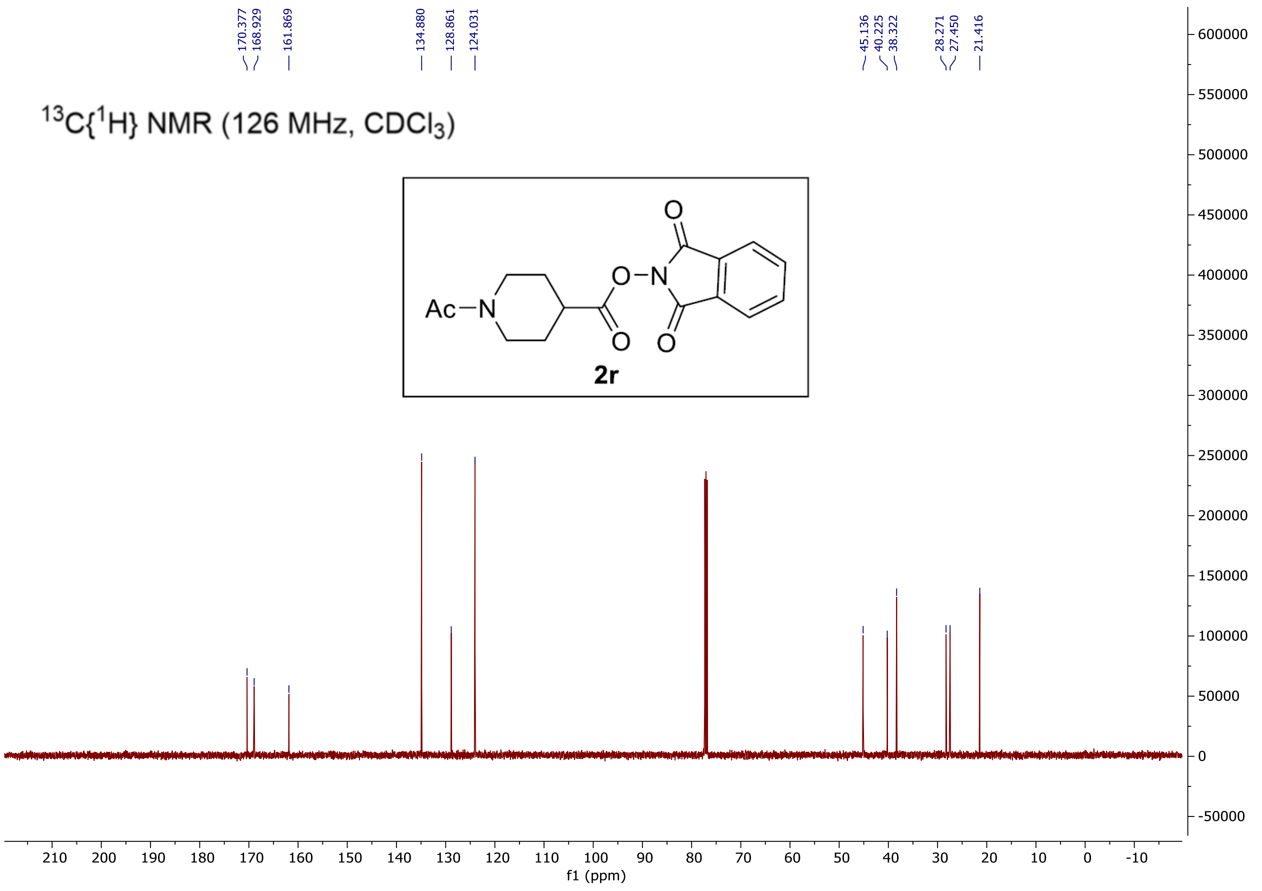

^{13}C{^{1}H} NMR (126 MHz, CDCl$_3$)
170.377
168.929
161.869
134.880
128.861
124.031
45.136
40.225
38.322
28.271
27.450
21.416
Ac−N
O−N
O
2r
f1 (ppm)

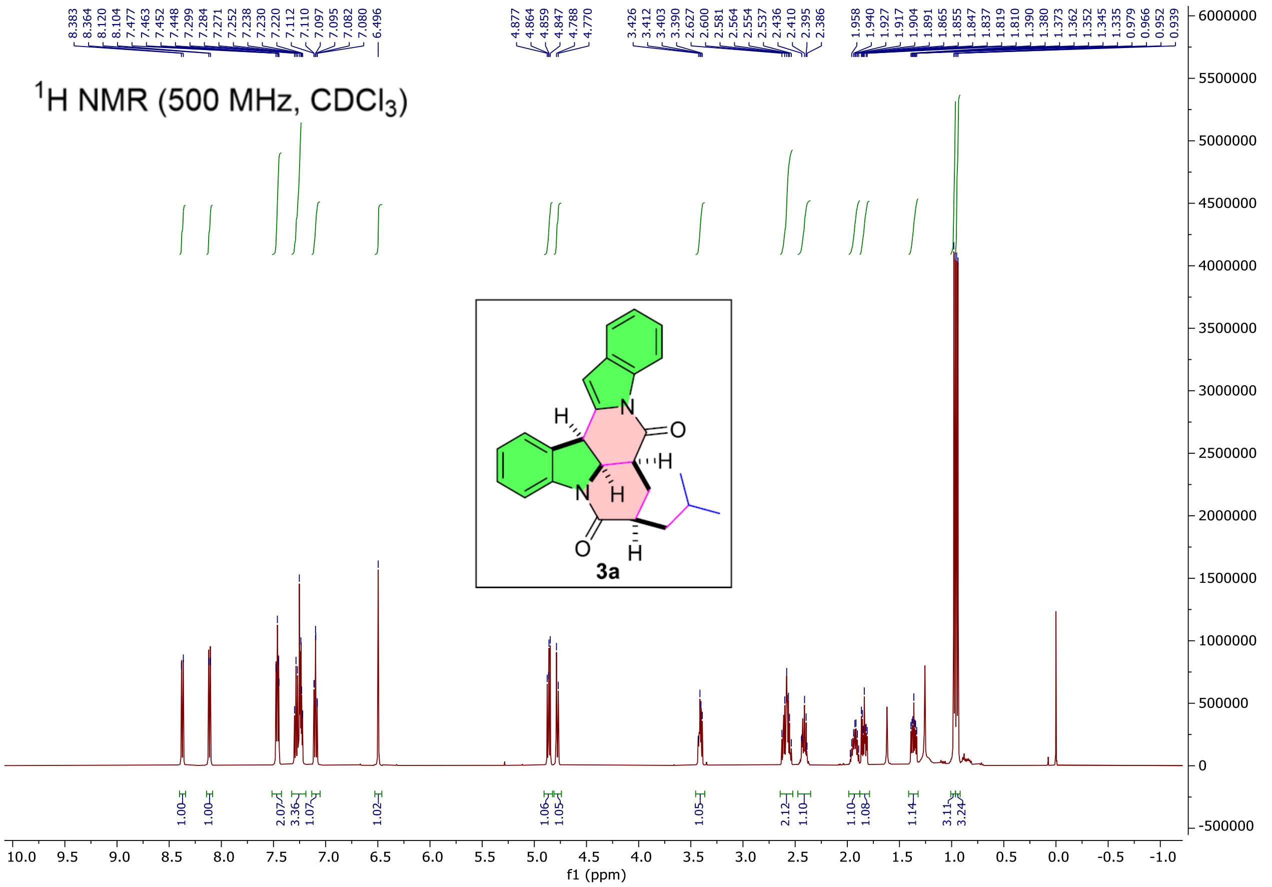
1H NMR (500 MHz, CDCl3)
3a
f1 (ppm)

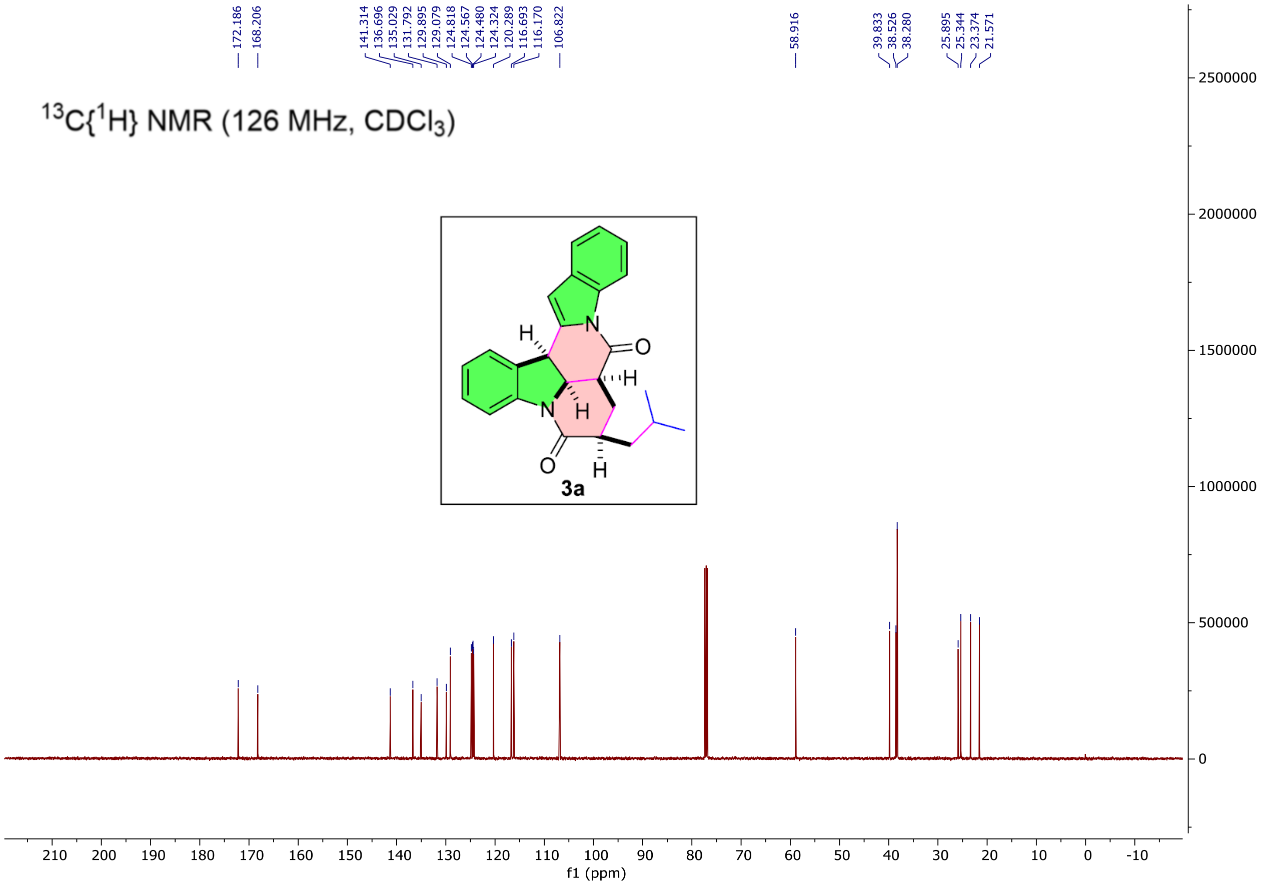
13C{1H} NMR (126 MHz, CDCl3)
3a
f1 (ppm)

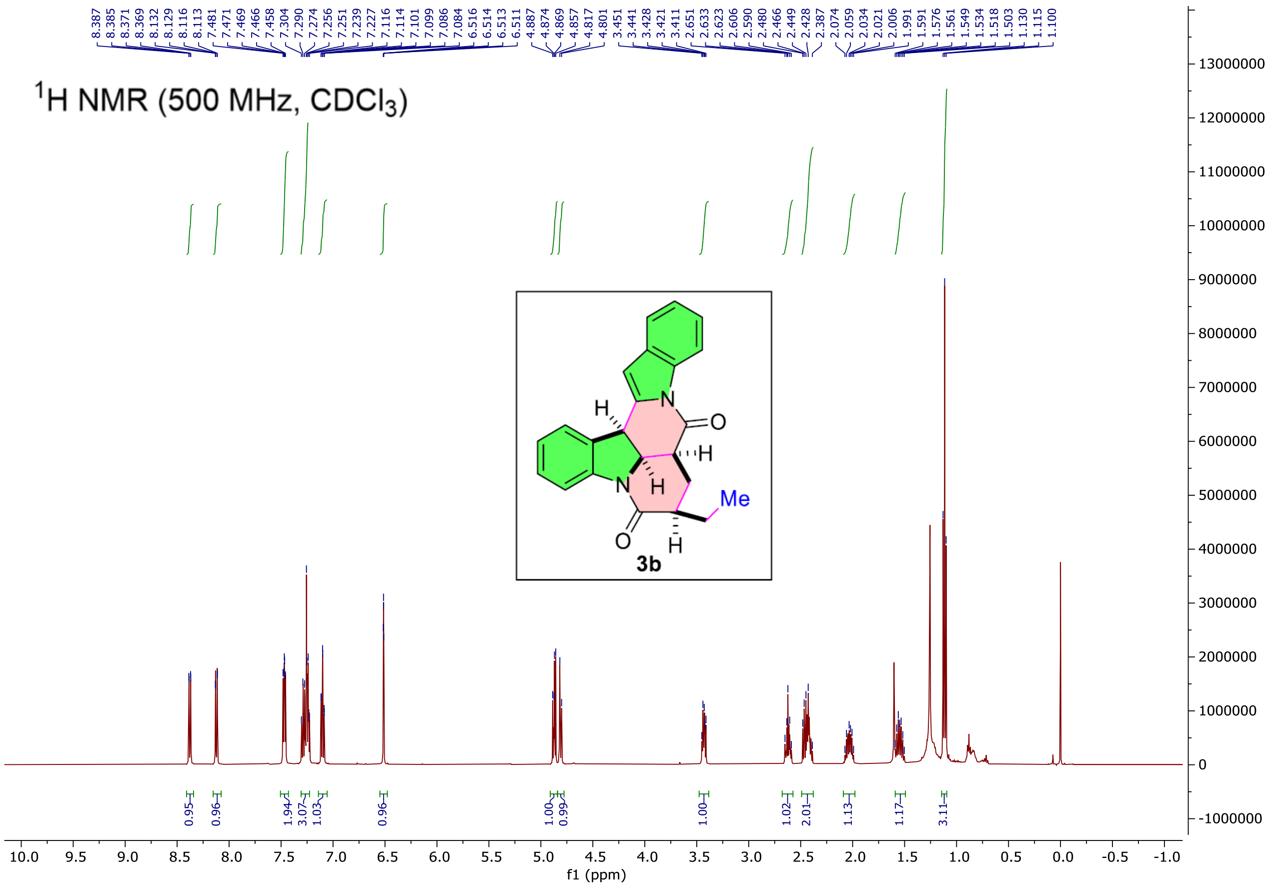

8.387 8.385 8.371 8.369 8.132 8.129 8.116 8.113 7.481 7.471 7.469 7.466 7.458 7.304 7.290 7.274 7.256 7.251 7.239 7.227 7.116 7.114 7.101 7.099 7.086 7.084 6.516 6.514 6.513 6.511 4.887 4.874 4.869 4.857 4.817 4.801 3.451 3.441 3.428 3.421 3.411 2.651 2.633 2.623 2.606 2.590 2.480 2.466 2.449 2.428 2.387 2.074 2.059 2.034 2.021 2.006 1.991 1.591 1.576 1.561 1.549 1.534 1.518 1.503 1.130 1.115 1.100
^{1}H NMR (500 MHz, CDCl$_3$)
H
N
O
H
N
H
Me
O
H
3b
0.95 0.96 1.94 3.07 1.03 0.96 1.00 0.99 1.00 1.02 2.01 1.13 1.17 3.11
f1 (ppm)

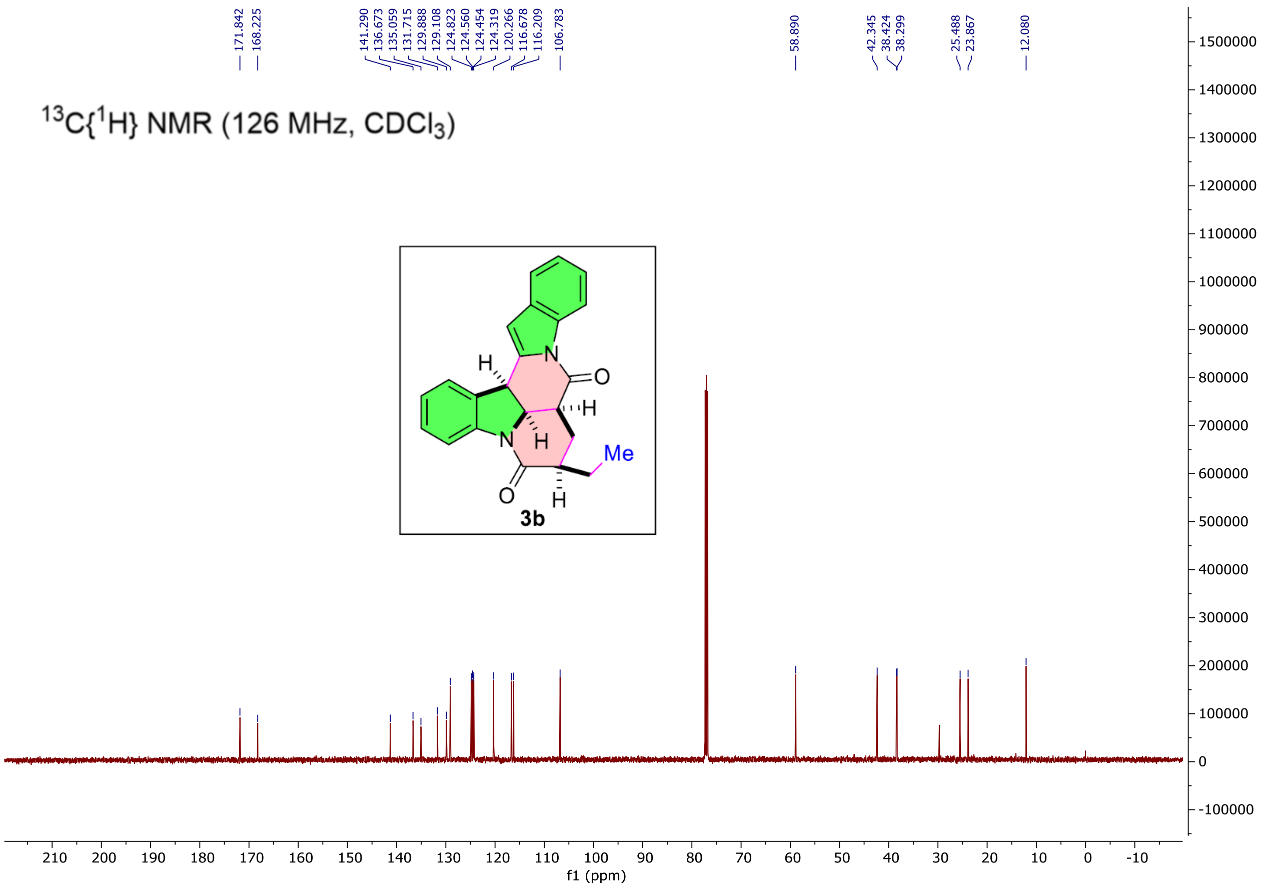

171.842 168.225 141.290 136.673 135.059 131.715 129.888 129.108 124.823 124.560 124.454 124.319 120.266 116.678 116.209 106.783 58.890 42.345 38.424 38.299 25.488 23.867 12.080
^{13}C{^{1}H} NMR (126 MHz, CDCl$_3$)
H
N
O
H
N
H
Me
O
H
3b
f1 (ppm)

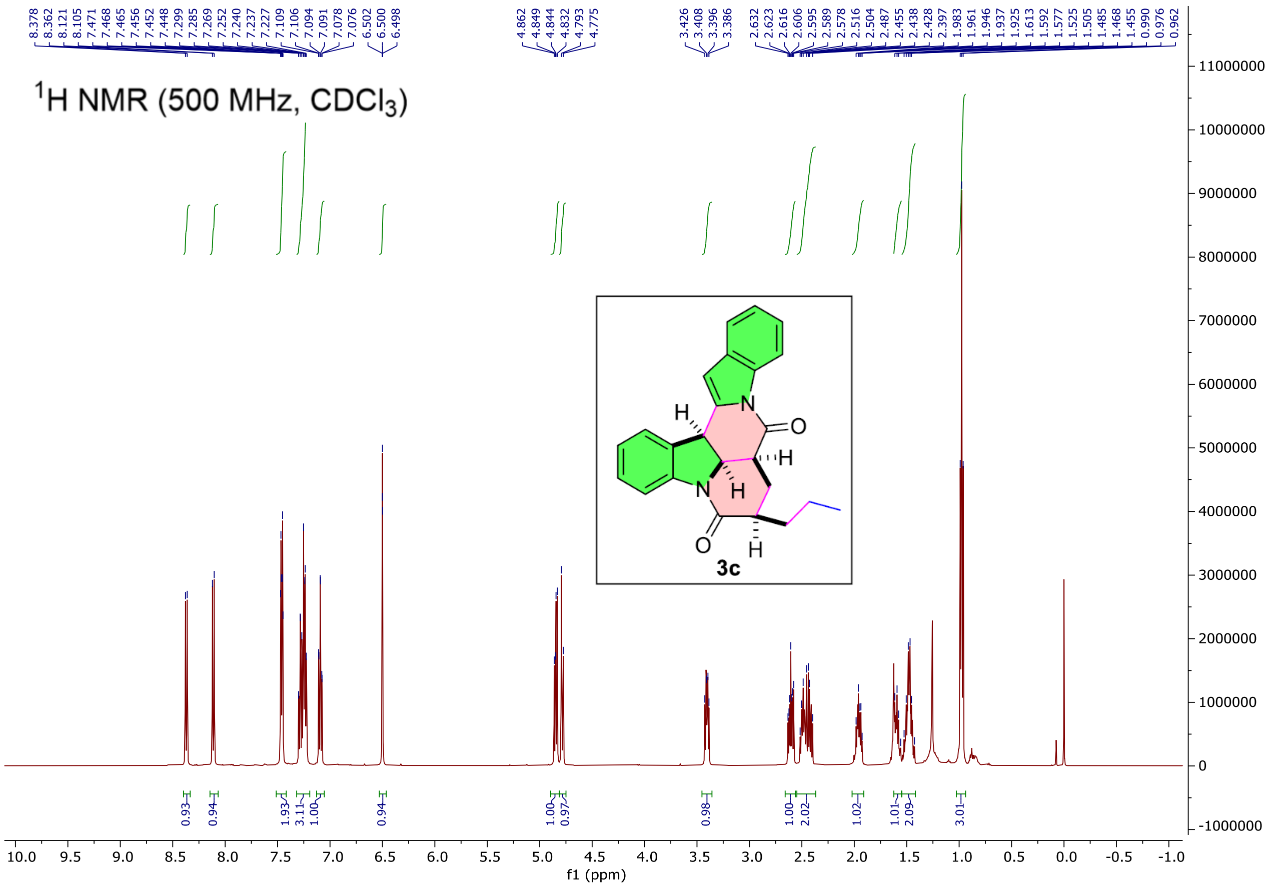
1H NMR (500 MHz, CDCl3)
3c
f1 (ppm)

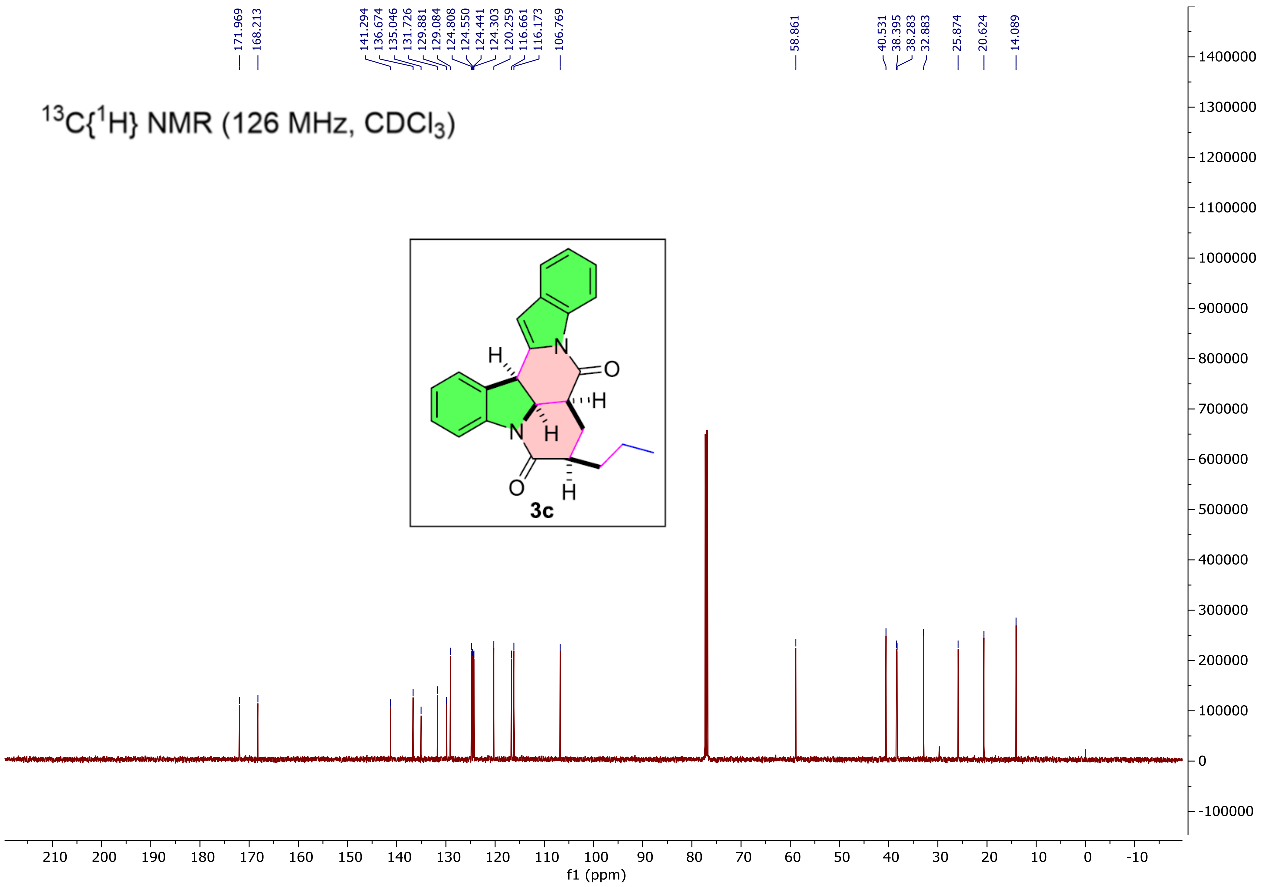
13C{1H} NMR (126 MHz, CDCl3)
3c
f1 (ppm)

^1H NMR (500 MHz, $CDCl_3$)

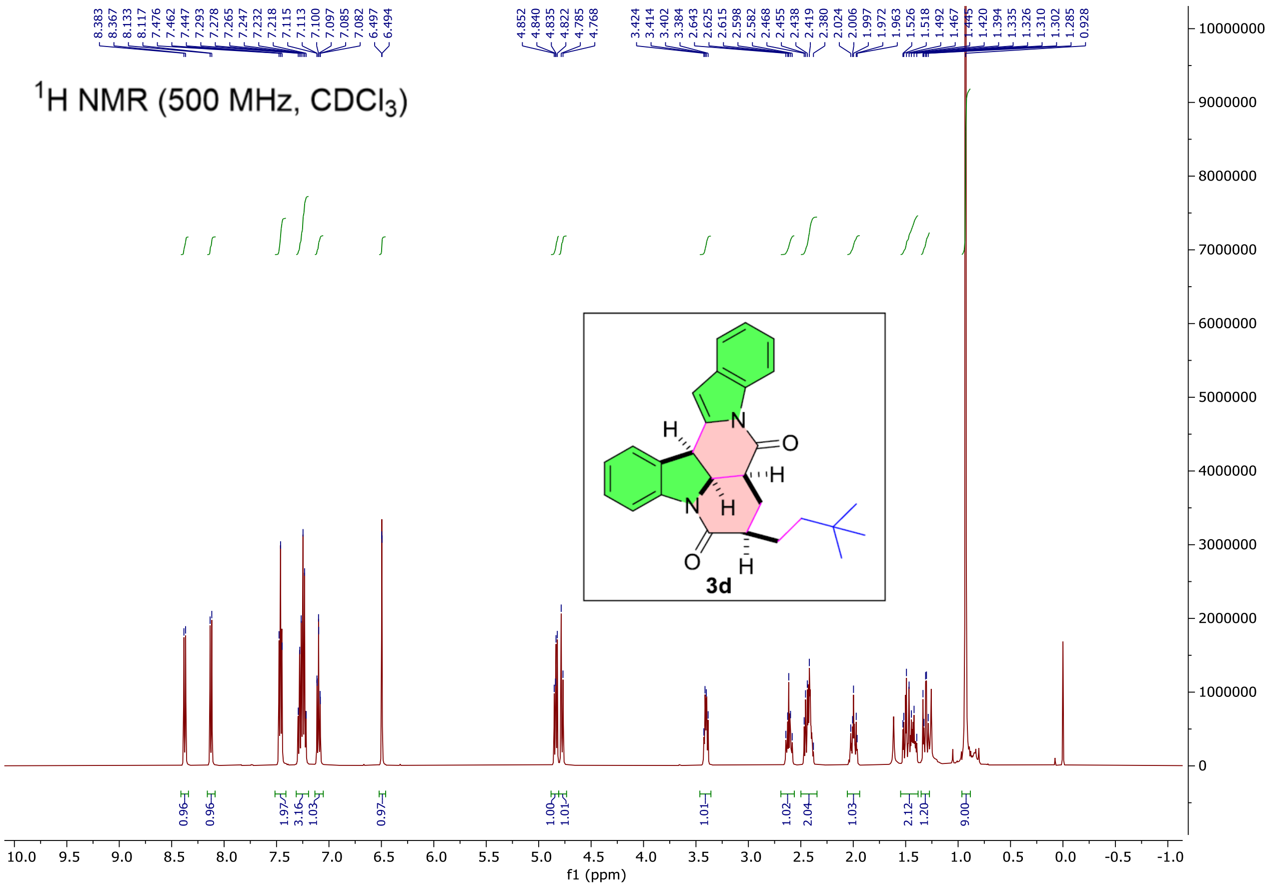

$^{13}C\{^1H\}$ NMR (126 MHz, $CDCl_3$)

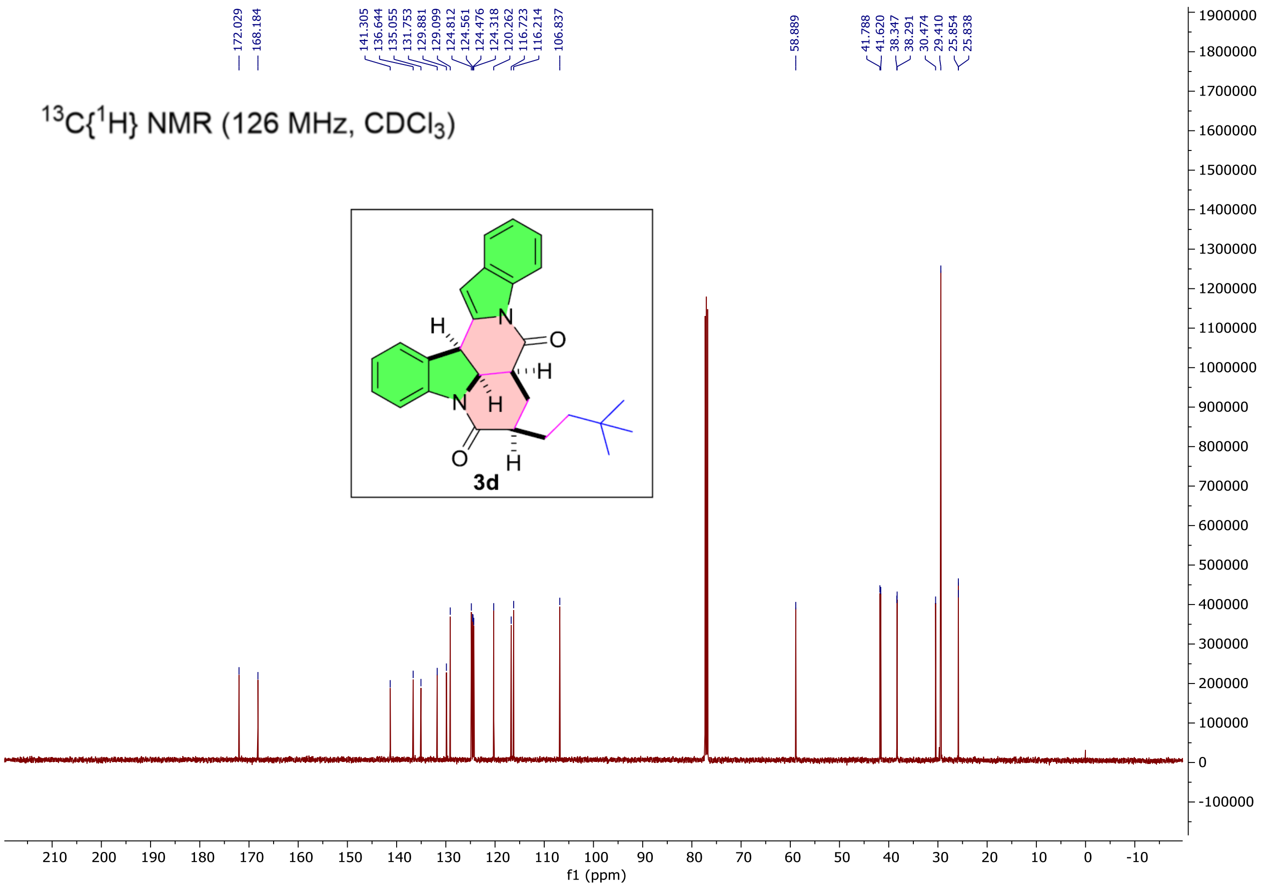

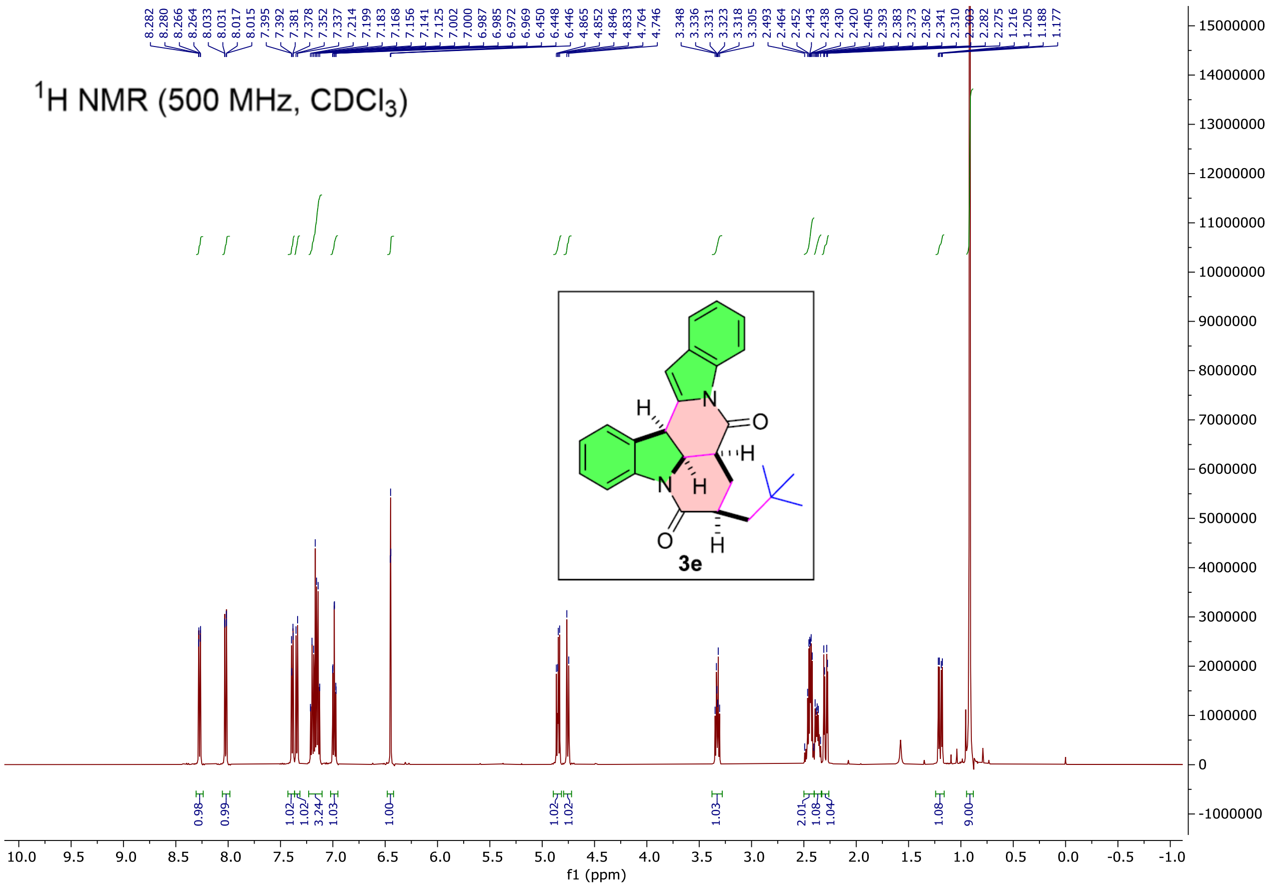
1H NMR (500 MHz, CDCl3)
3e
f1 (ppm)

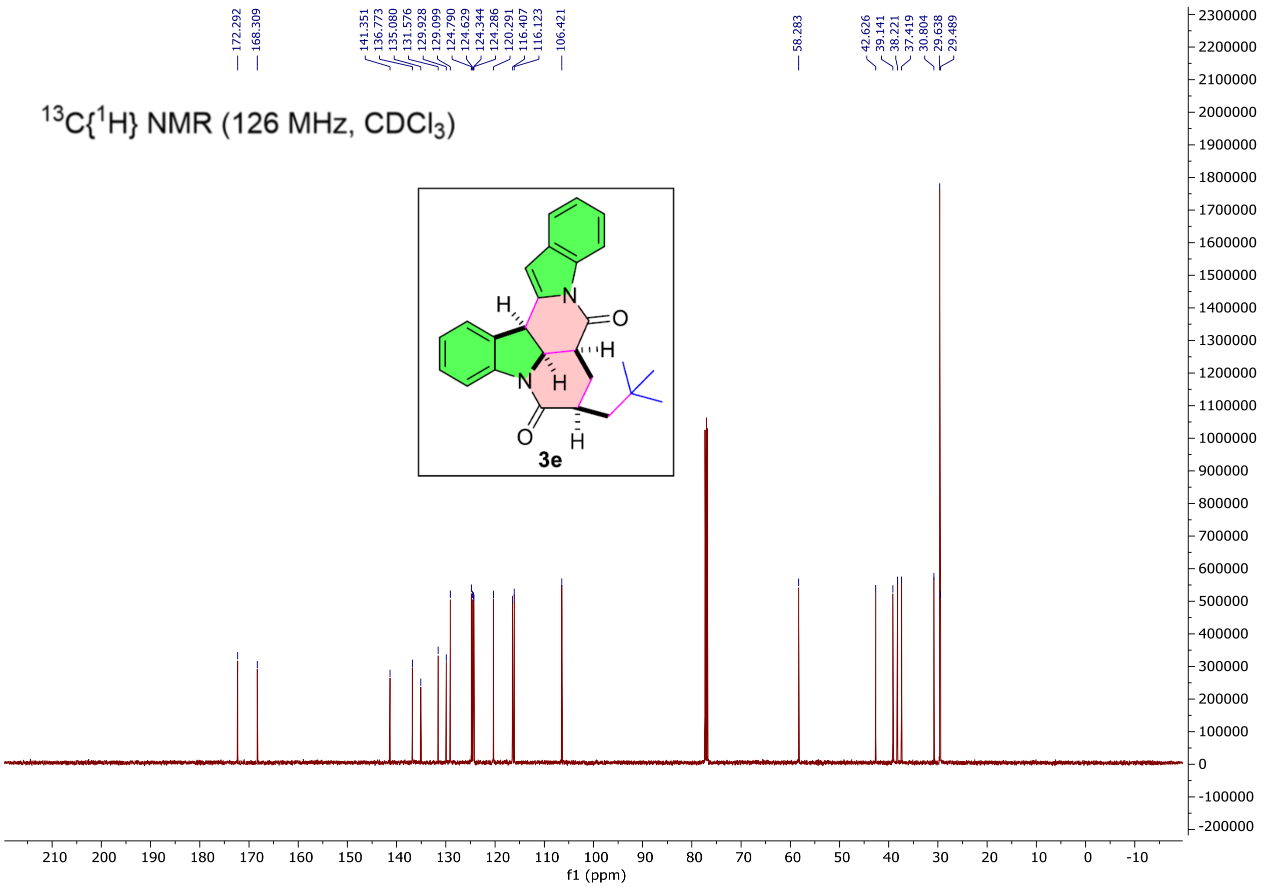
13C{1H} NMR (126 MHz, CDCl3)
3e
f1 (ppm)

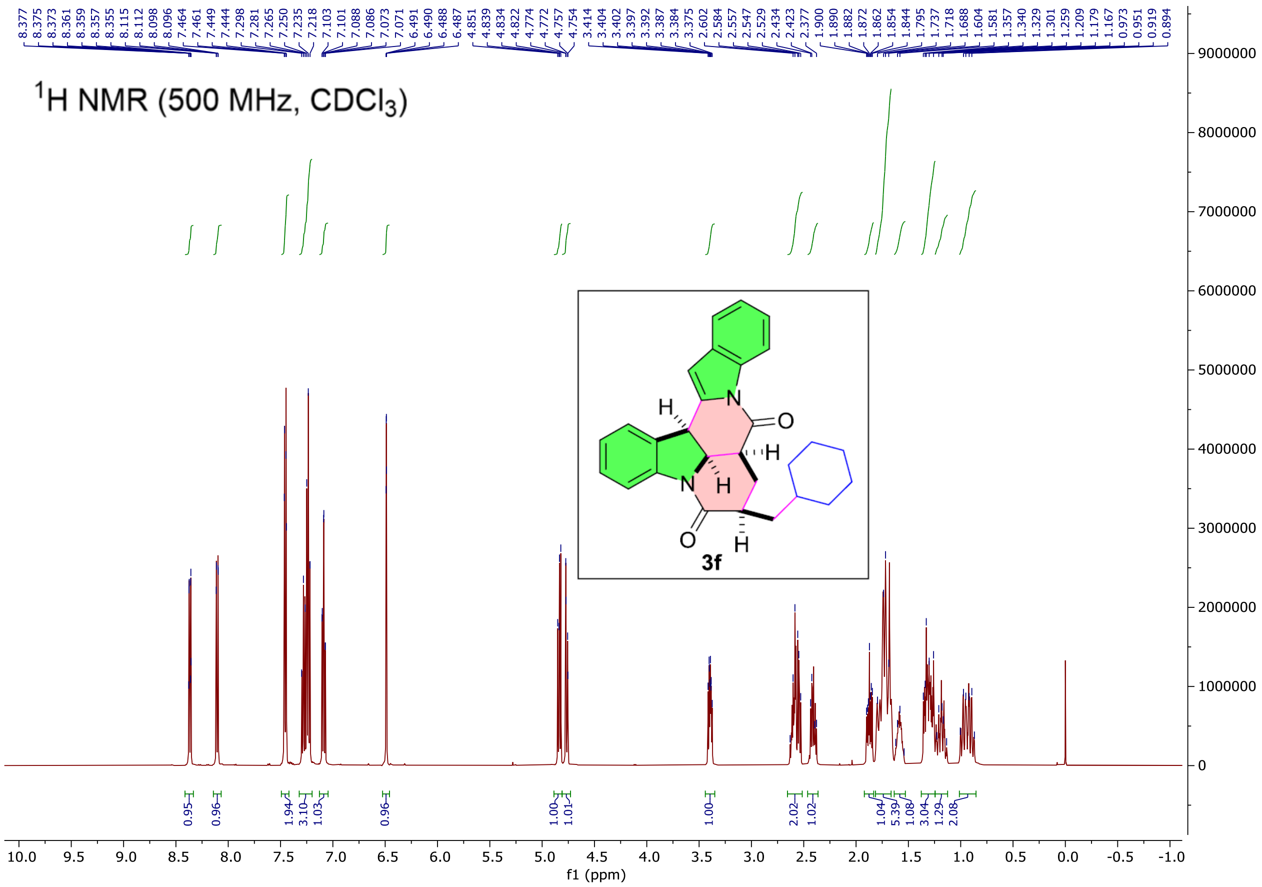
8.377
8.375
8.373
8.361
8.359
8.357
8.355
8.115
8.112
8.098
8.096
7.464
7.461
7.449
7.444
7.298
7.281
7.265
7.250
7.235
7.218
7.103
7.101
7.088
7.086
7.073
7.071
6.491
6.490
6.488
6.487
4.851
4.839
4.834
4.822
4.774
4.772
4.757
4.754
3.414
3.404
3.402
3.397
3.392
3.387
3.384
3.375
2.602
2.584
2.557
2.547
2.529
2.434
2.423
2.377
1.900
1.890
1.882
1.872
1.862
1.854
1.844
1.795
1.737
1.718
1.688
1.604
1.581
1.357
1.340
1.329
1.301
1.259
1.209
1.179
1.167
0.973
0.951
0.919
0.894
1H NMR (500 MHz, CDCl3)
H
N
O
H
N
H
O
H
3f
0.95
0.96
1.94
3.10
1.03
0.96
1.00
1.01
1.00
2.02
1.02
1.04
5.39
1.08
3.04
1.29
2.08
f1 (ppm)

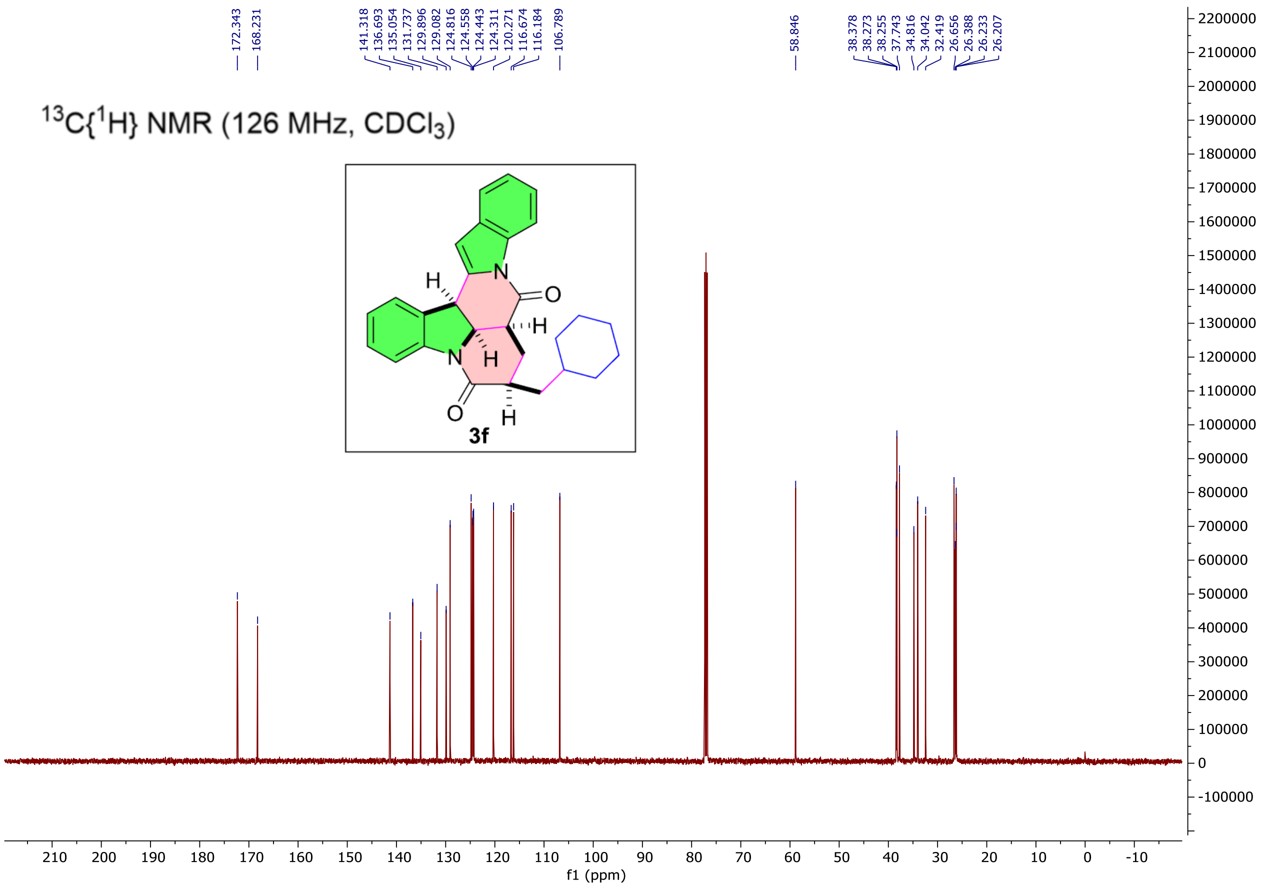
172.343
168.231
141.318
136.693
135.054
131.737
129.896
129.082
124.816
124.558
124.443
124.311
120.271
116.674
116.184
106.789
58.846
38.378
38.273
38.255
37.743
34.816
34.042
32.419
26.656
26.388
26.233
26.207
13C{1H} NMR (126 MHz, CDCl3)
H
N
O
H
N
H
O
H
3f
f1 (ppm)

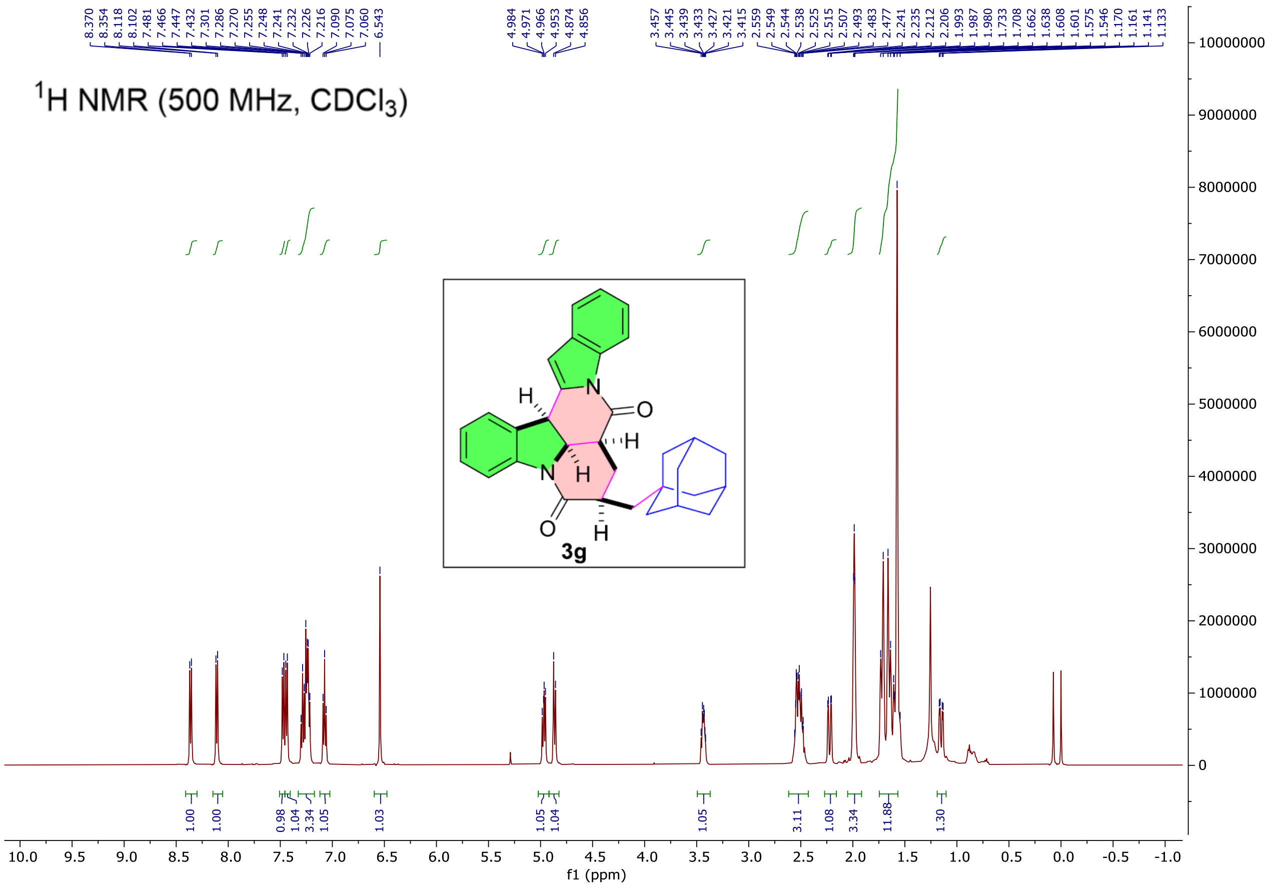
1H NMR (500 MHz, CDCl3)
8.370 8.354 8.118 8.102 7.481 7.466 7.447 7.432 7.301 7.286 7.270 7.255 7.248 7.241 7.232 7.226 7.216 7.090 7.075 7.060 6.543
4.984 4.971 4.966 4.953 4.874 4.856
3.457 3.445 3.439 3.433 3.427 3.421 3.415 2.559 2.549 2.544 2.538 2.525 2.515 2.507 2.493 2.483 2.477 2.241 2.235 2.212 2.206 1.993 1.987 1.980 1.733 1.708 1.662 1.638 1.608 1.601 1.575 1.546 1.170 1.161 1.141 1.133
1.00 1.00 0.98 1.04 3.34 1.05 1.03 1.05 1.04 1.05 3.11 1.08 3.34 11.88 1.30
N
O
H
3g
f1 (ppm)

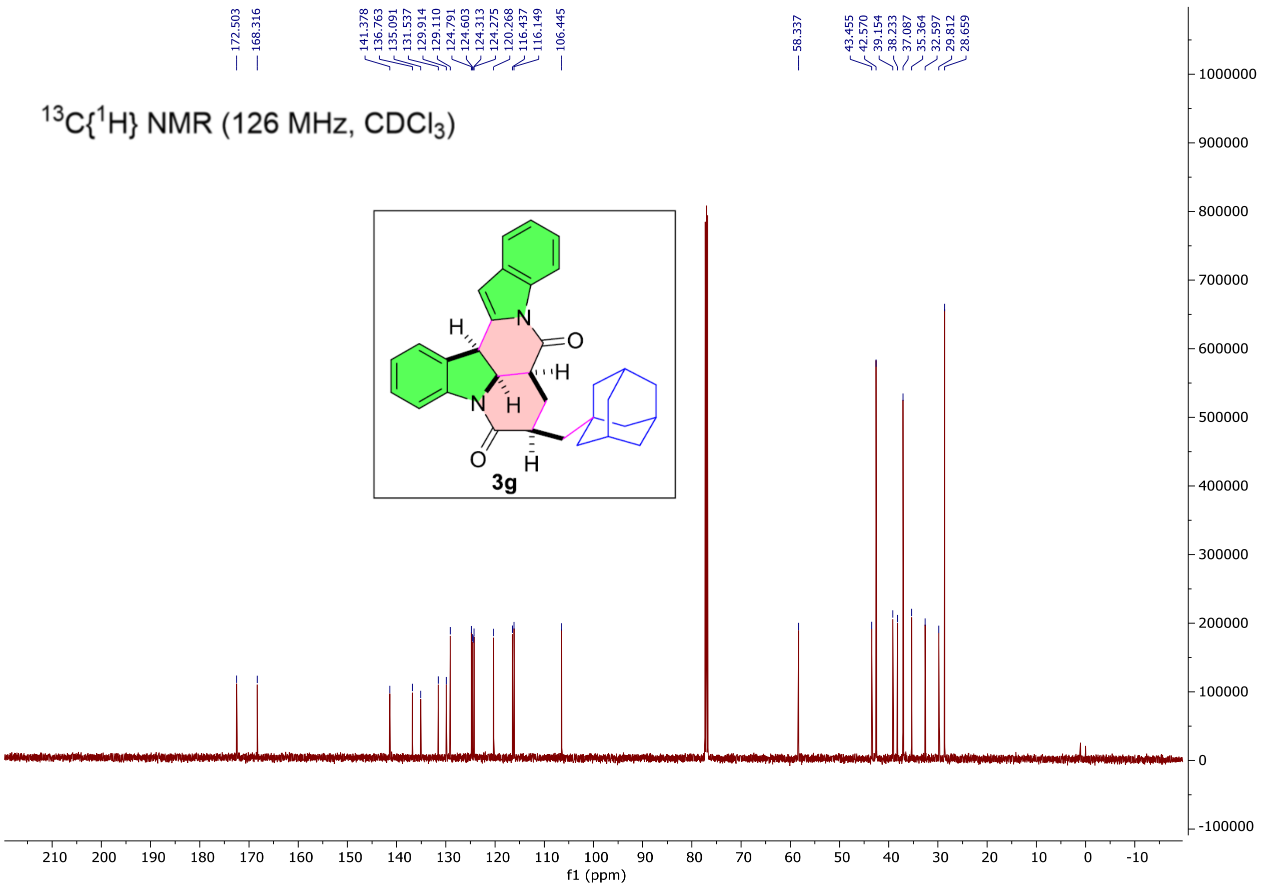
13C{1H} NMR (126 MHz, CDCl3)
172.503 168.316
141.378 136.763 135.091 131.537 129.914 129.110 124.791 124.603 124.313 124.275 120.268 116.437 116.149 106.445
58.337
43.455 42.570 39.154 38.233 37.087 35.364 32.597 29.812 28.659
N
O
H
3g
f1 (ppm)

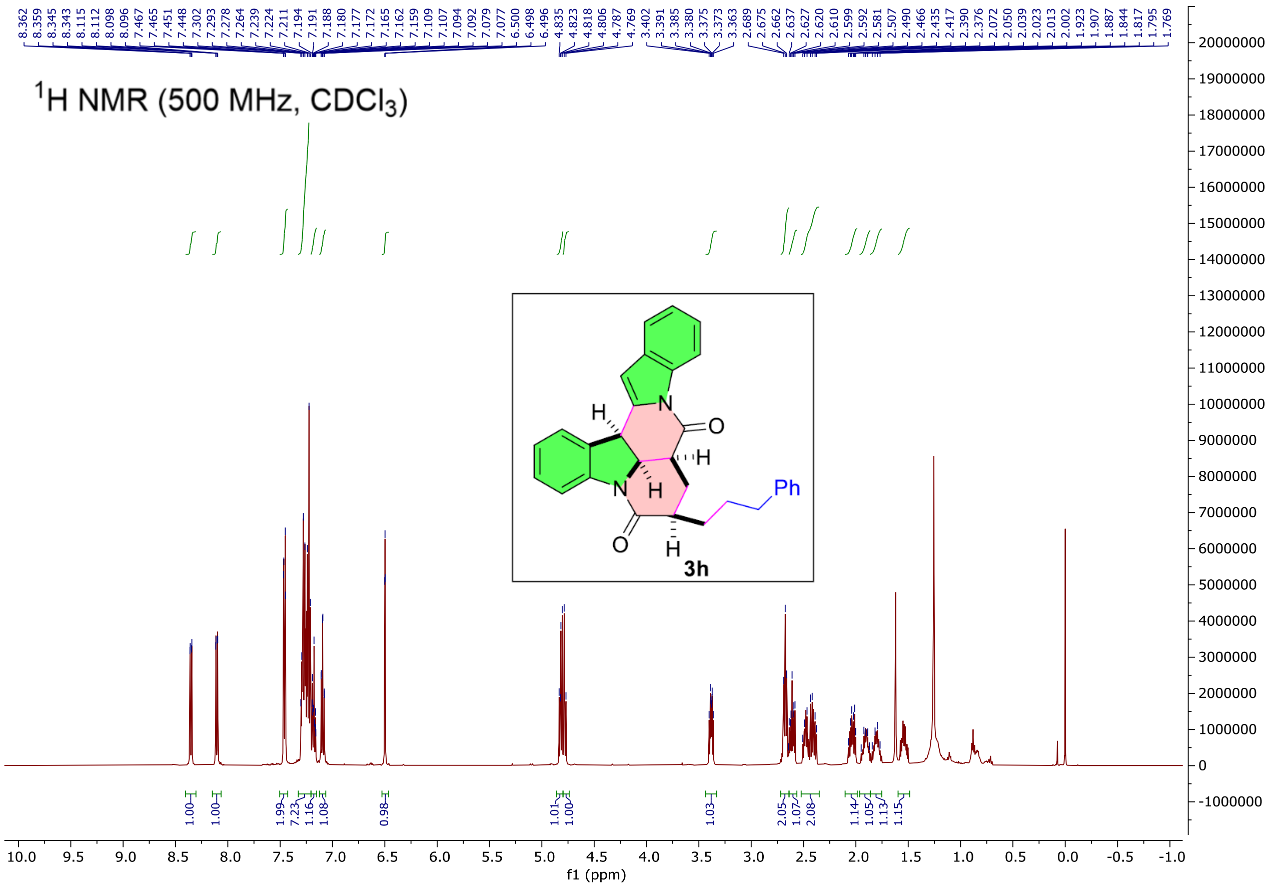

^{1}H NMR (500 MHz, CDCl$_3$)
3h
Ph

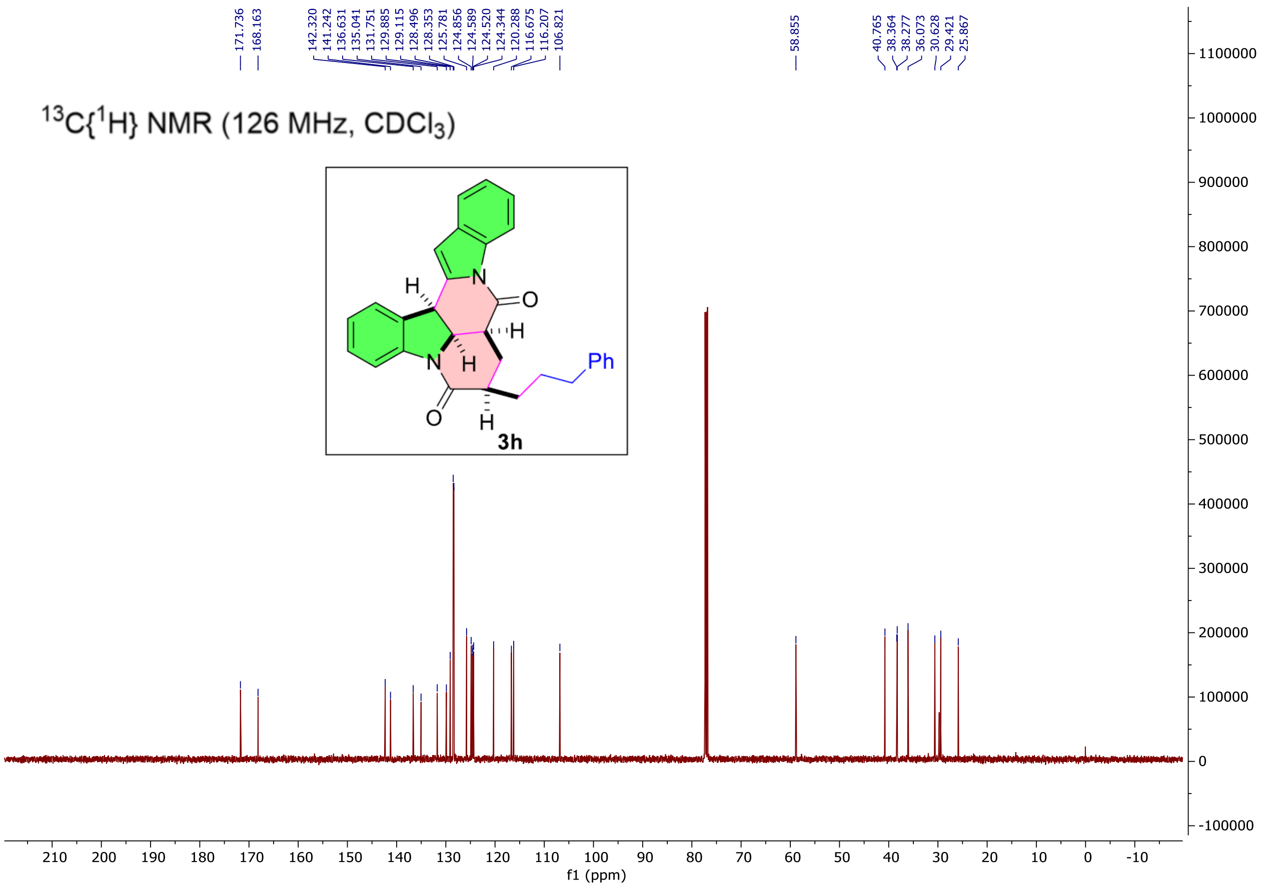

^{13}C{^{1}H} NMR (126 MHz, CDCl$_3$)
3h
Ph

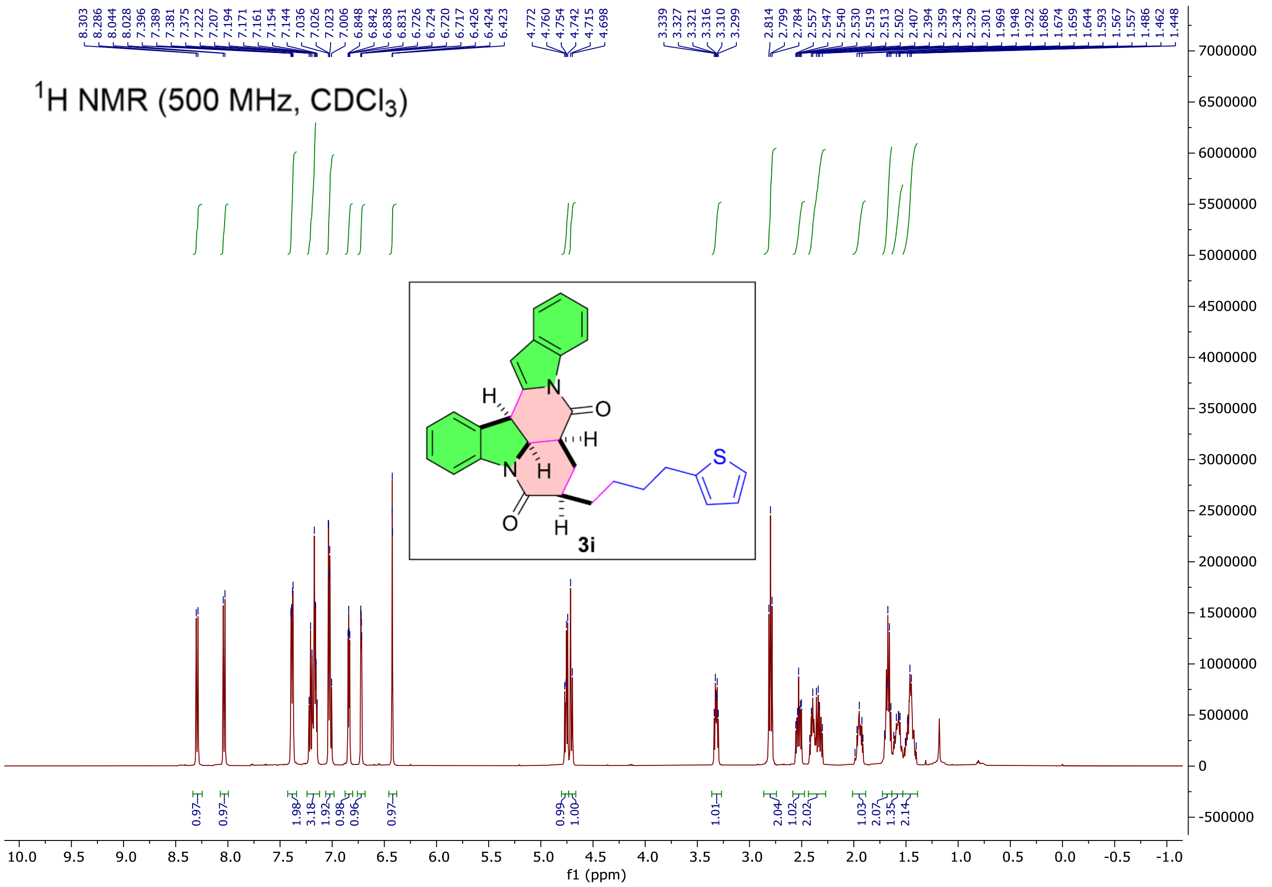

8.303 8.286 8.044 8.028 7.396 7.389 7.381 7.375 7.222 7.207 7.194 7.171 7.161 7.154 7.144 7.036 7.026 7.023 7.006 6.848 6.842 6.838 6.831 6.726 6.724 6.720 6.717 6.426 6.424 6.423 4.772 4.760 4.754 4.742 4.715 4.698 3.339 3.327 3.321 3.316 3.310 3.299 2.814 2.799 2.784 2.557 2.547 2.540 2.530 2.519 2.513 2.502 2.407 2.394 2.359 2.342 2.329 2.301 1.969 1.948 1.922 1.686 1.674 1.659 1.644 1.593 1.567 1.557 1.486 1.462 1.448
^{1}H NMR (500 MHz, CDCl$_3$)
3i
f1 (ppm)

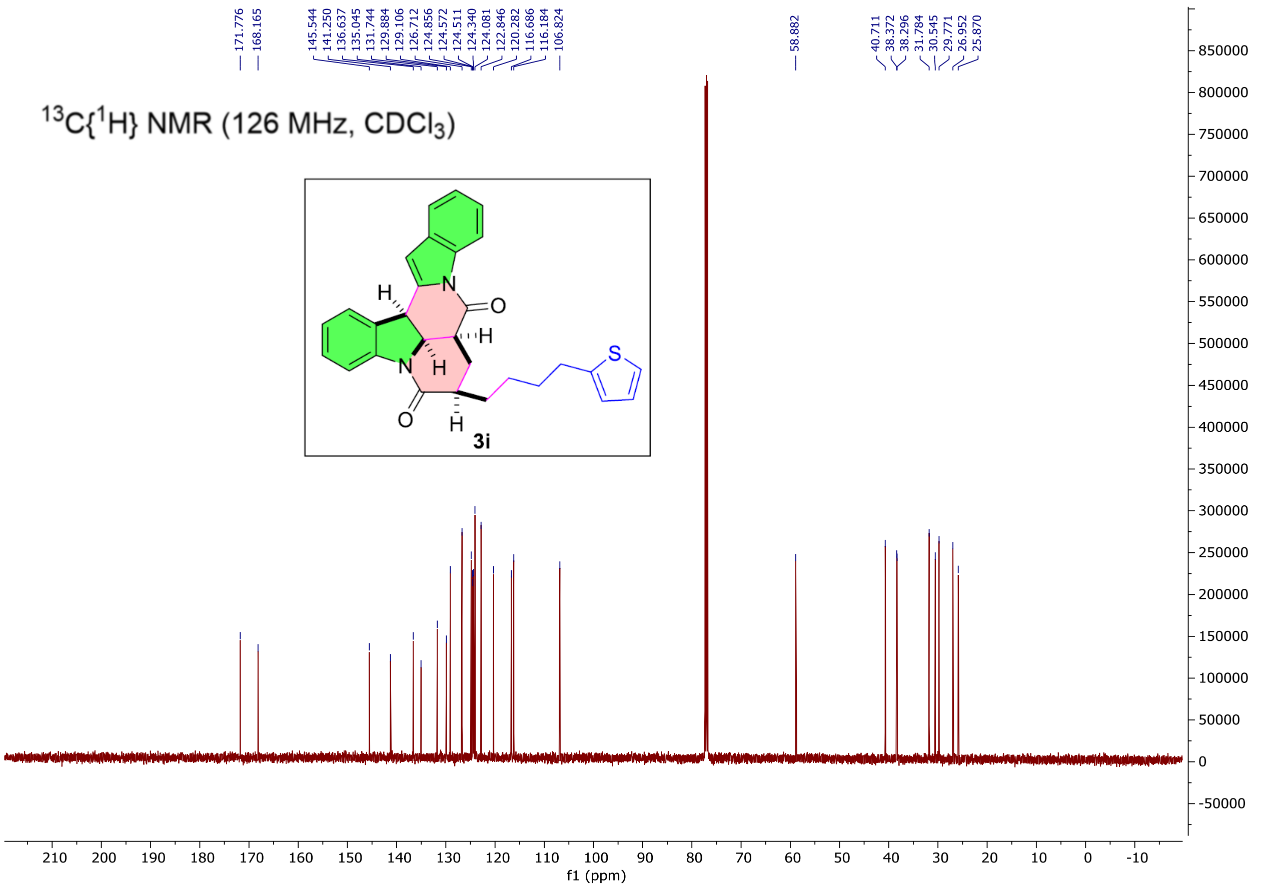

171.776 168.165 145.544 141.250 136.637 135.045 131.744 129.884 129.106 126.712 124.856 124.572 124.511 124.340 124.081 122.846 120.282 116.686 116.184 106.824 58.882 40.711 38.372 38.296 31.784 30.545 29.771 26.952 25.870
^{13}C{^{1}H} NMR (126 MHz, CDCl$_3$)
3i
f1 (ppm)

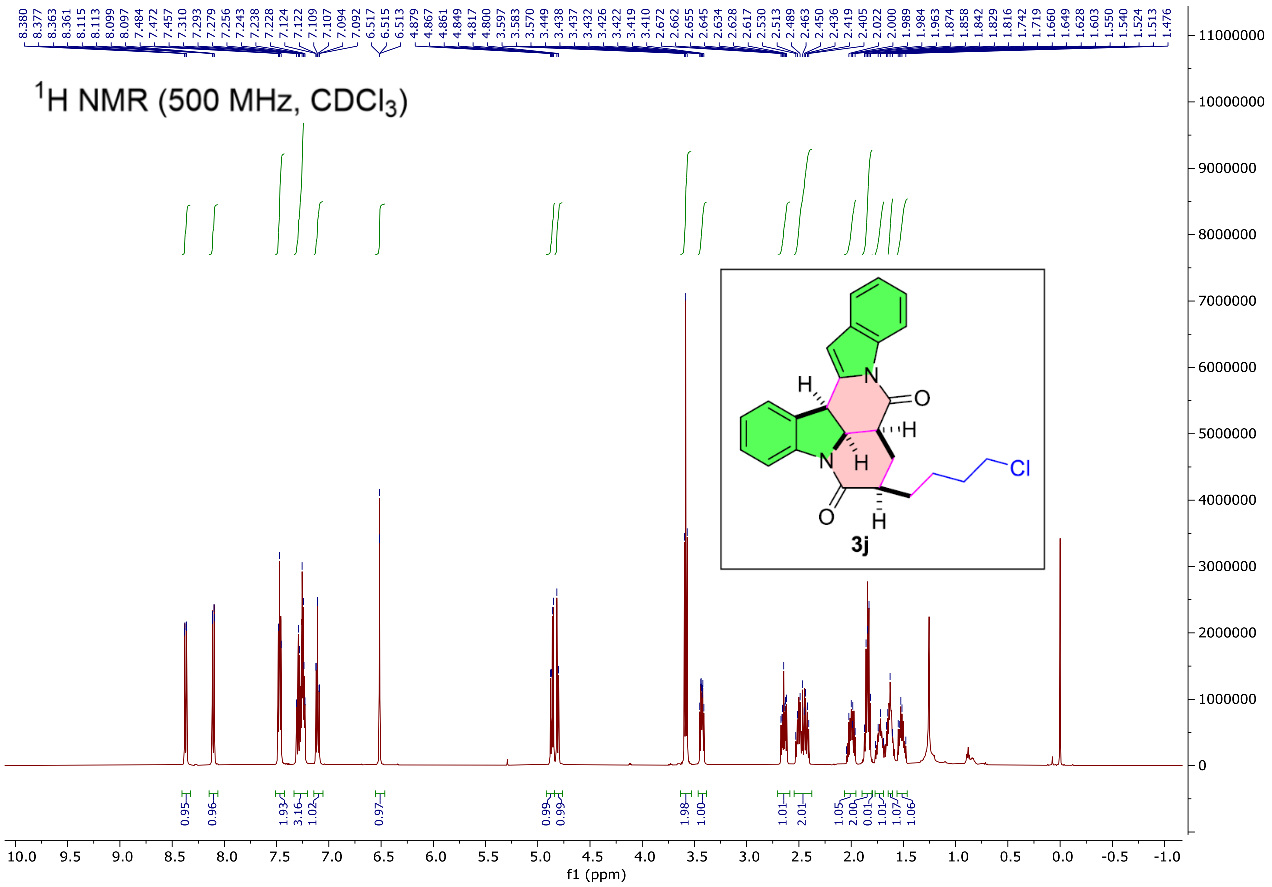

1H NMR (500 MHz, CDCl3)
3j
f1 (ppm)

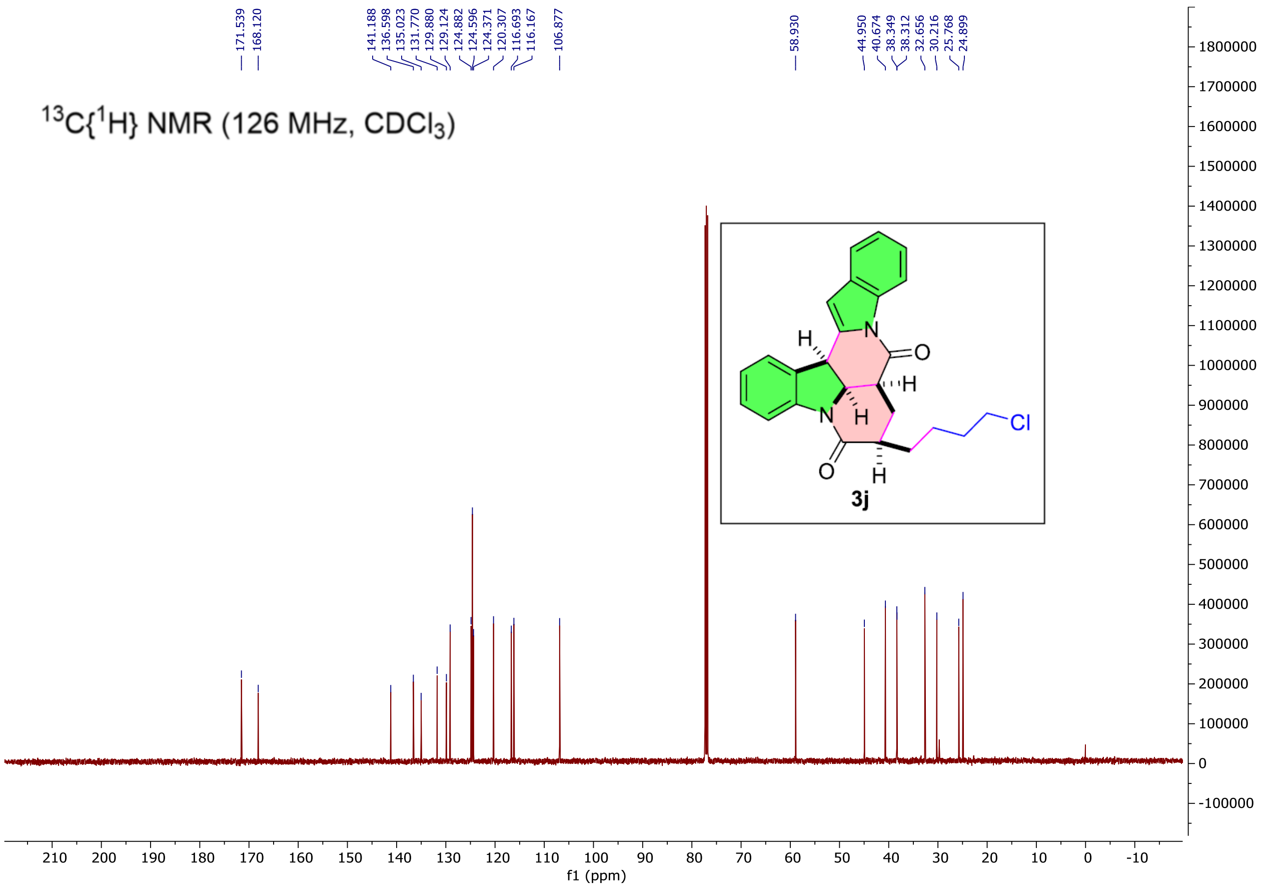

13C{1H} NMR (126 MHz, CDCl3)
3j
f1 (ppm)

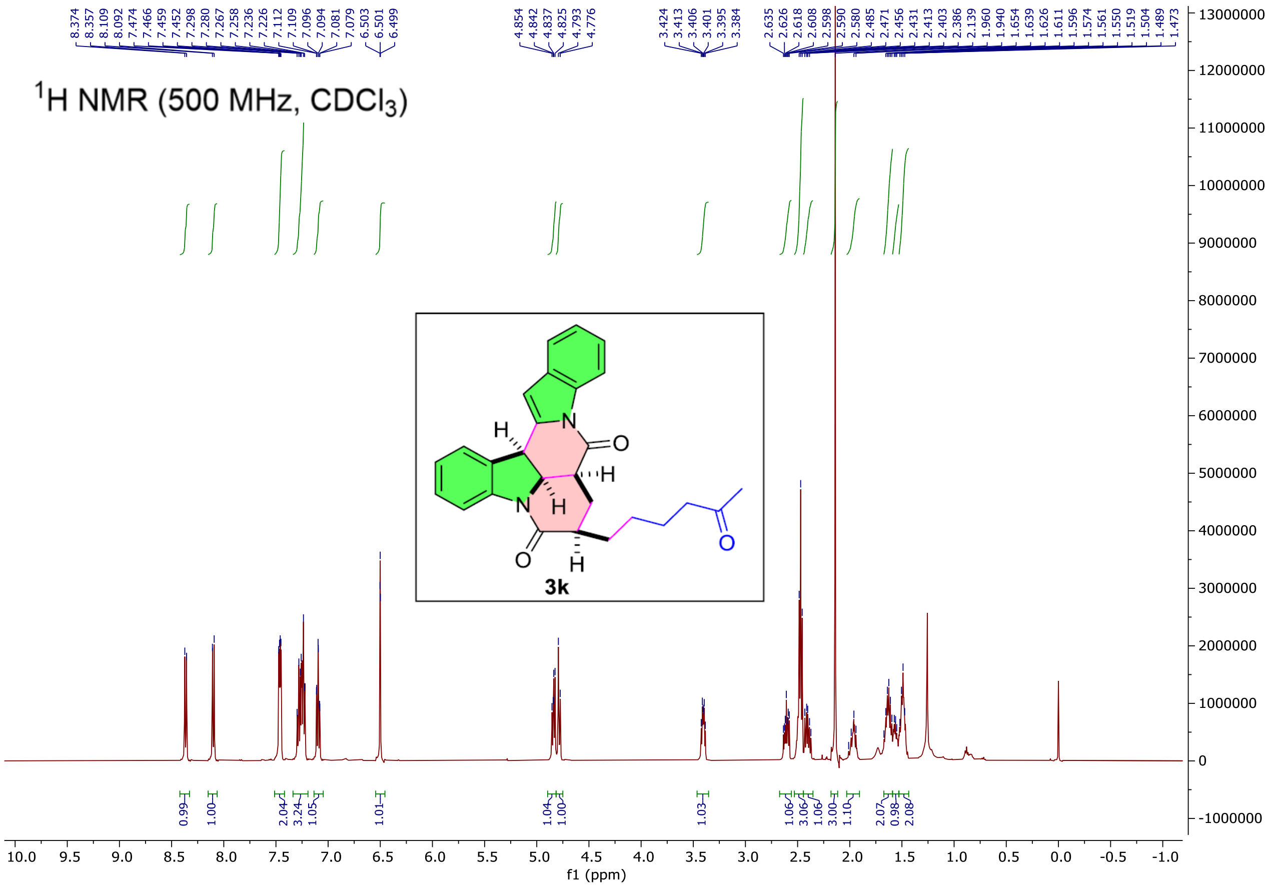

1H NMR (500 MHz, CDCl3)
8.374 8.357 8.109 8.092 7.474 7.466 7.459 7.452 7.298 7.280 7.267 7.258 7.236 7.226 7.112 7.109 7.096 7.094 7.081 7.079 6.503 6.501 6.499
4.854 4.842 4.837 4.825 4.793 4.776
3.424 3.413 3.406 3.401 3.395 3.384
2.635 2.626 2.618 2.608 2.598 2.590 2.580 2.485 2.471 2.456 2.431 2.413 2.403 2.386 2.139 1.960 1.940 1.654 1.639 1.626 1.611 1.596 1.574 1.561 1.550 1.519 1.504 1.489 1.473
3k
f1 (ppm)

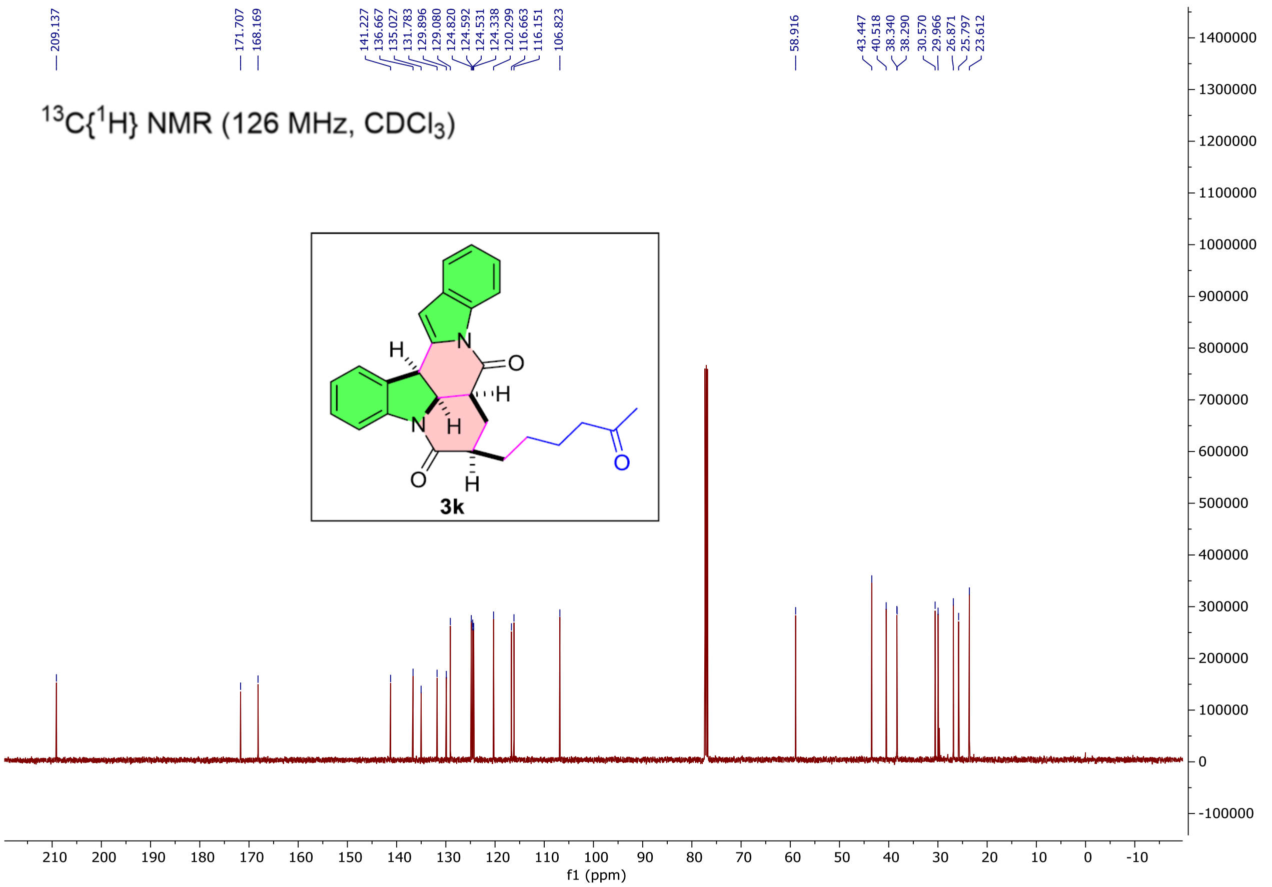

13C{1H} NMR (126 MHz, CDCl3)
209.137 171.707 168.169 141.227 136.667 135.027 131.783 129.896 129.080 124.820 124.592 124.531 124.338 120.299 116.663 116.151 106.823 58.916 43.447 40.518 38.340 38.290 30.570 29.966 26.871 25.797 23.612
3k
f1 (ppm)

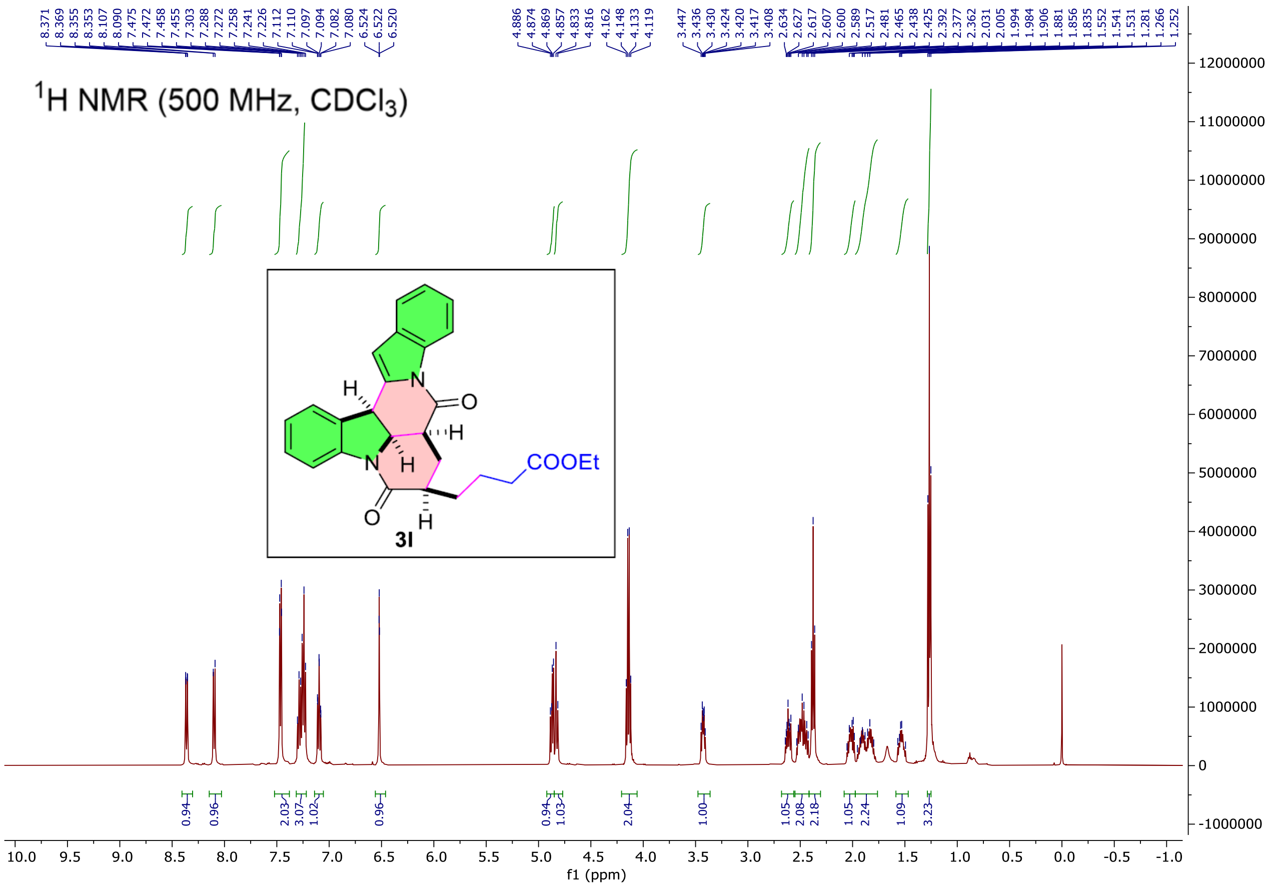

^{1}H NMR (500 MHz, $CDCl_3$)
COOEt
3l
f1 (ppm)

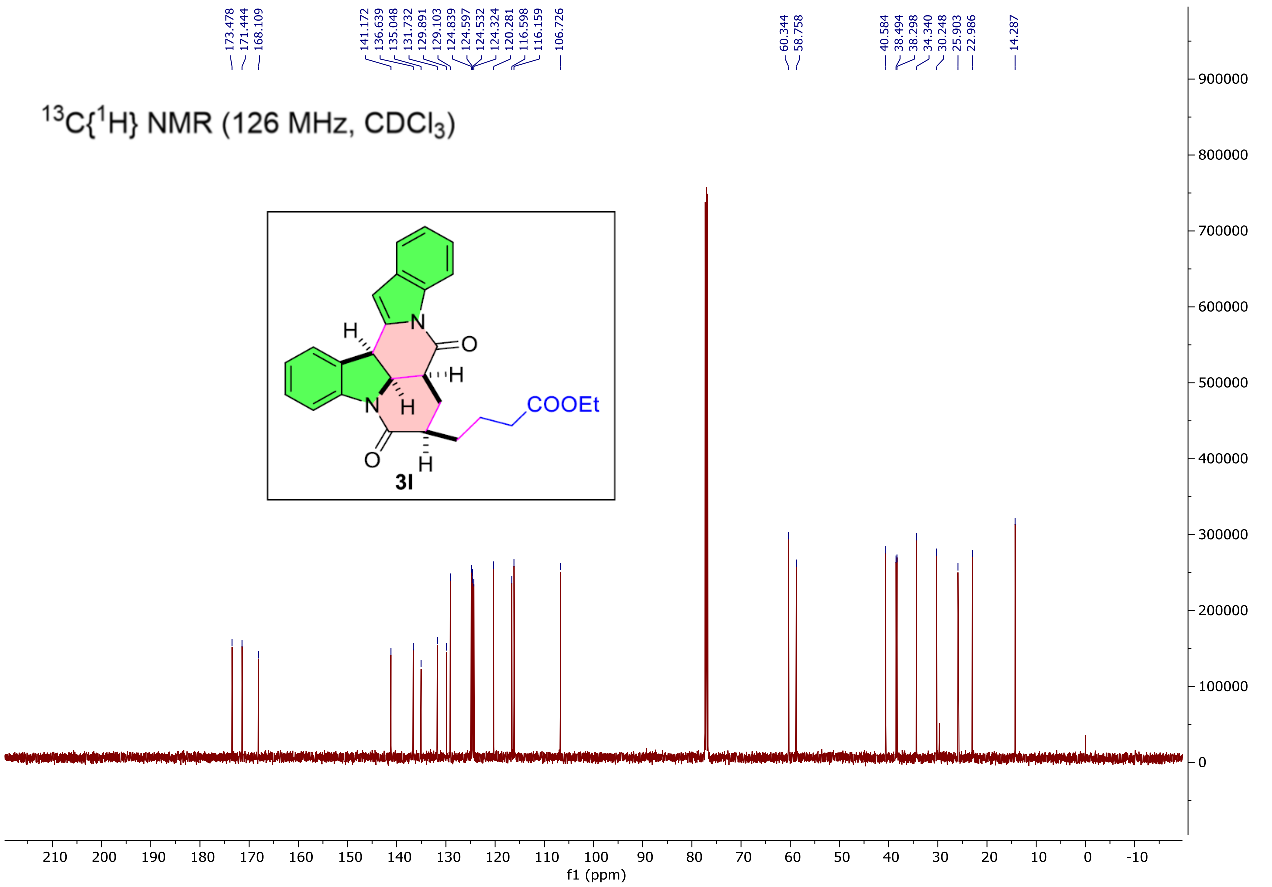

$^{13}C\{^{1}H\}$ NMR (126 MHz, $CDCl_3$)
COOEt
3l
f1 (ppm)

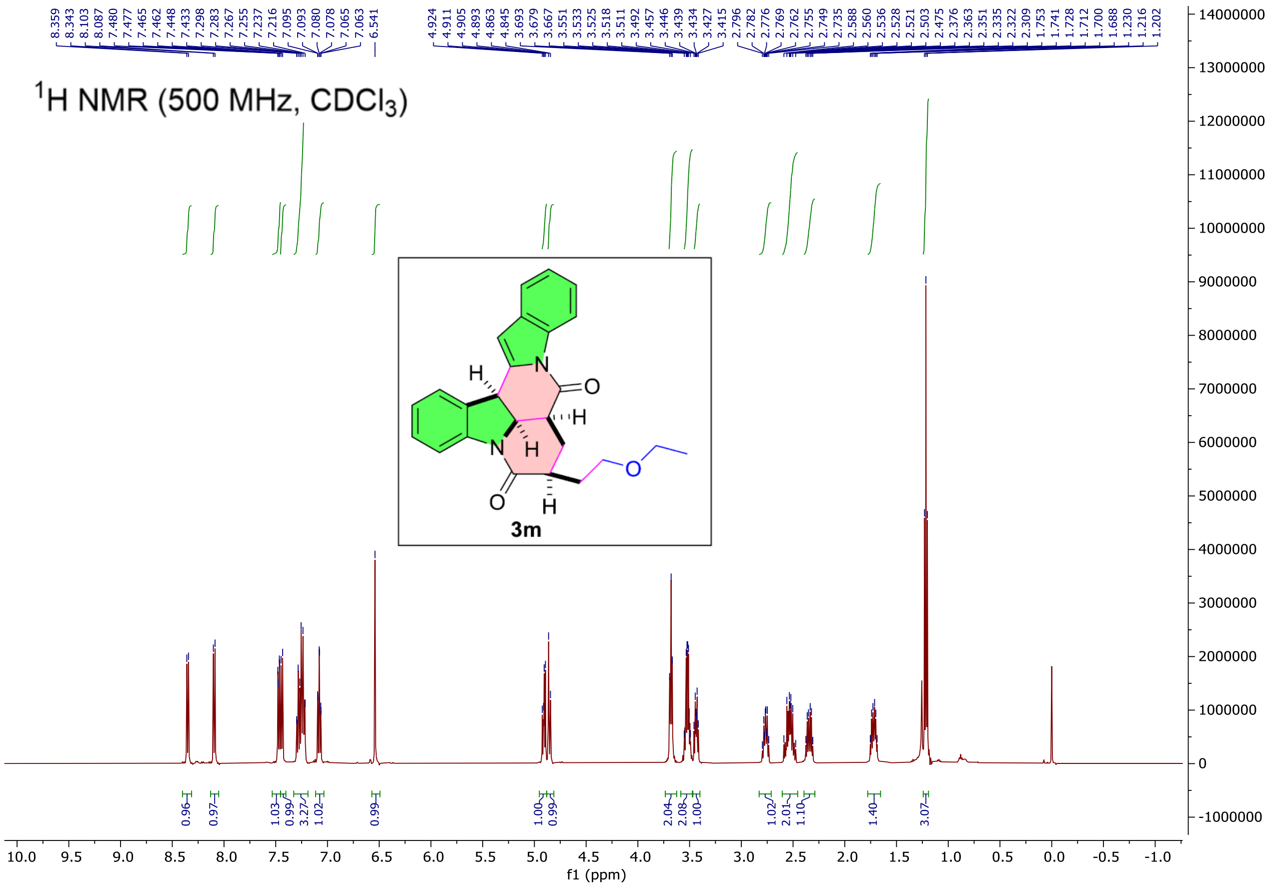

1H NMR (500 MHz, CDCl3)
8.359 8.343 8.103 8.087 7.480 7.477 7.465 7.462 7.448 7.433 7.298 7.283 7.267 7.255 7.237 7.216 7.095 7.093 7.080 7.078 7.065 7.063 6.541
4.924 4.911 4.905 4.893 4.863 4.845 3.693 3.679 3.667 3.551 3.533 3.525 3.518 3.511 3.492 3.457 3.446 3.439 3.434 3.427 3.415 2.796 2.782 2.776 2.769 2.762 2.755 2.749 2.735 2.588 2.560 2.536 2.528 2.521 2.503 2.475 2.376 2.363 2.351 2.335 2.322 2.309 1.753 1.741 1.728 1.712 1.700 1.688 1.230 1.216 1.202
3m
0.96 0.97 1.03 0.99 3.27 1.02 0.99 1.00 0.99 2.04 2.08 1.00 1.02 2.01 1.10 1.40 3.07
f1 (ppm)

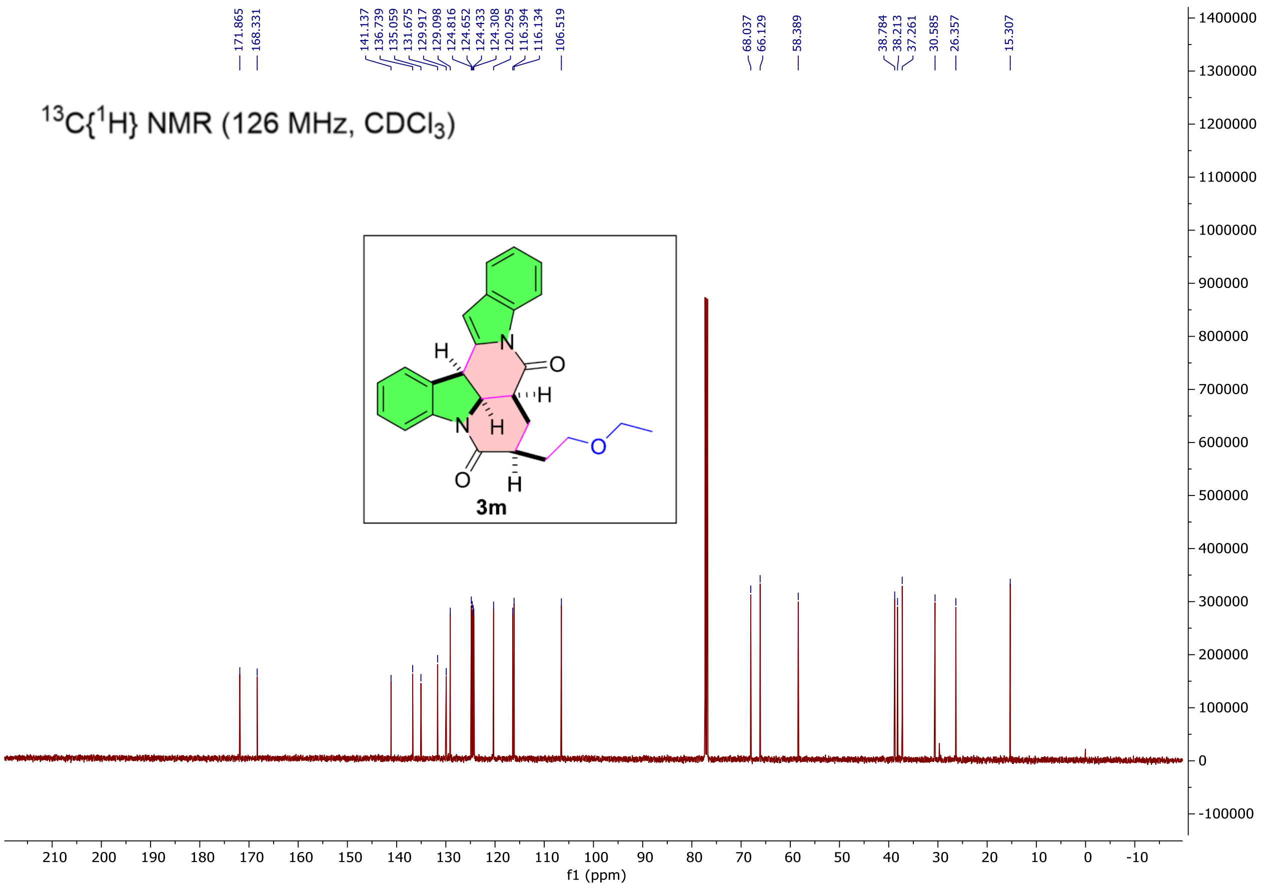

13C{1H} NMR (126 MHz, CDCl3)
171.865 168.331 141.137 136.739 135.059 131.675 129.917 129.098 124.816 124.652 124.433 124.308 120.295 116.394 116.134 106.519
68.037 66.129 58.389 38.784 38.213 37.261 30.585 26.357 15.307
3m
f1 (ppm)

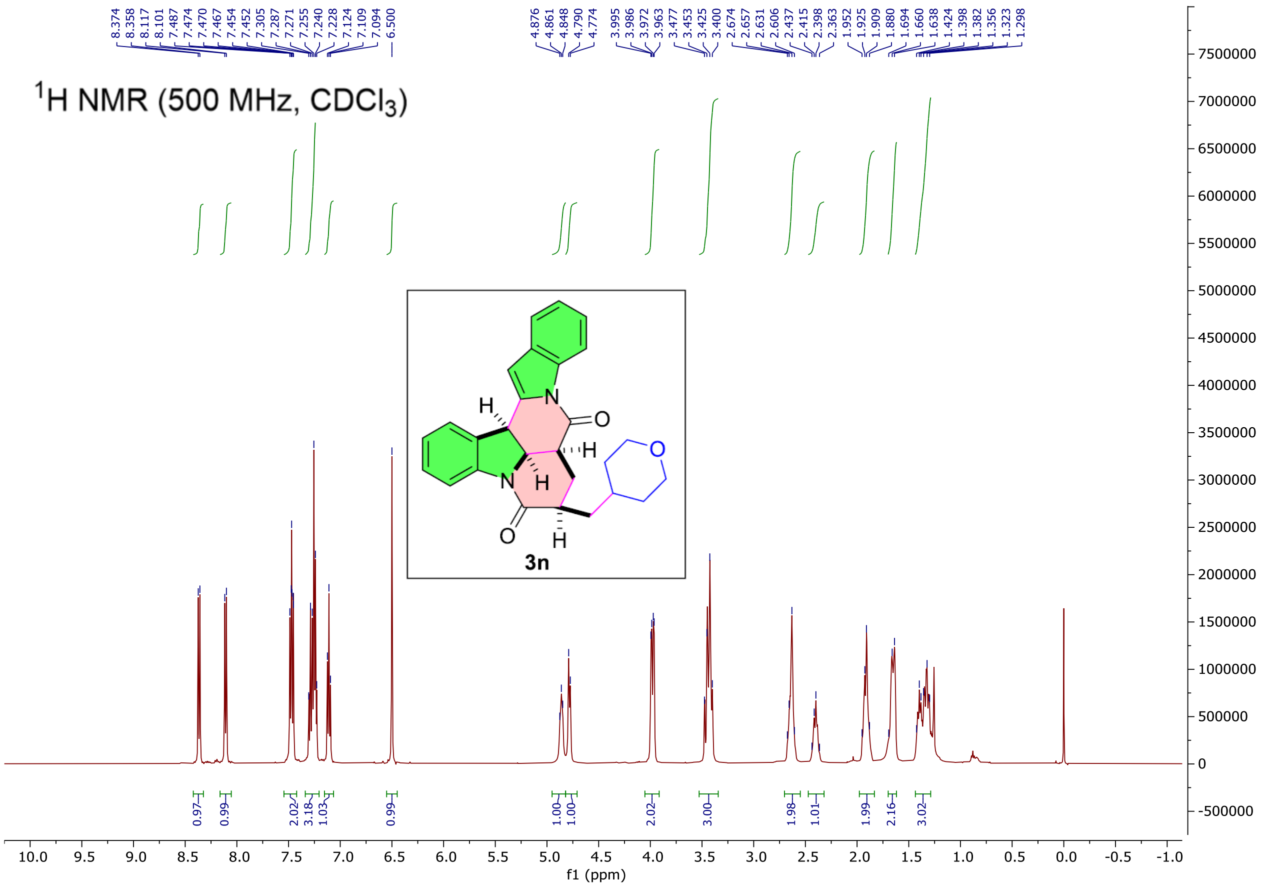

1H NMR (500 MHz, CDCl3)
8.374 8.358 8.117 8.101 7.487 7.474 7.470 7.467 7.454 7.452 7.305 7.287 7.271 7.255 7.240 7.228 7.124 7.109 7.094 6.500
4.876 4.861 4.848 4.790 4.774
3.995 3.986 3.972 3.963 3.477 3.453 3.425 3.400 2.674 2.657 2.631 2.606 2.437 2.415 2.398 2.363 1.952 1.925 1.909 1.880 1.694 1.660 1.638 1.424 1.398 1.382 1.356 1.323 1.298
0.97 0.99 2.02 3.18 1.03 0.99 1.00 1.00 2.02 3.00 1.98 1.01 1.99 2.16 3.02
N
O
H
3n
f1 (ppm)

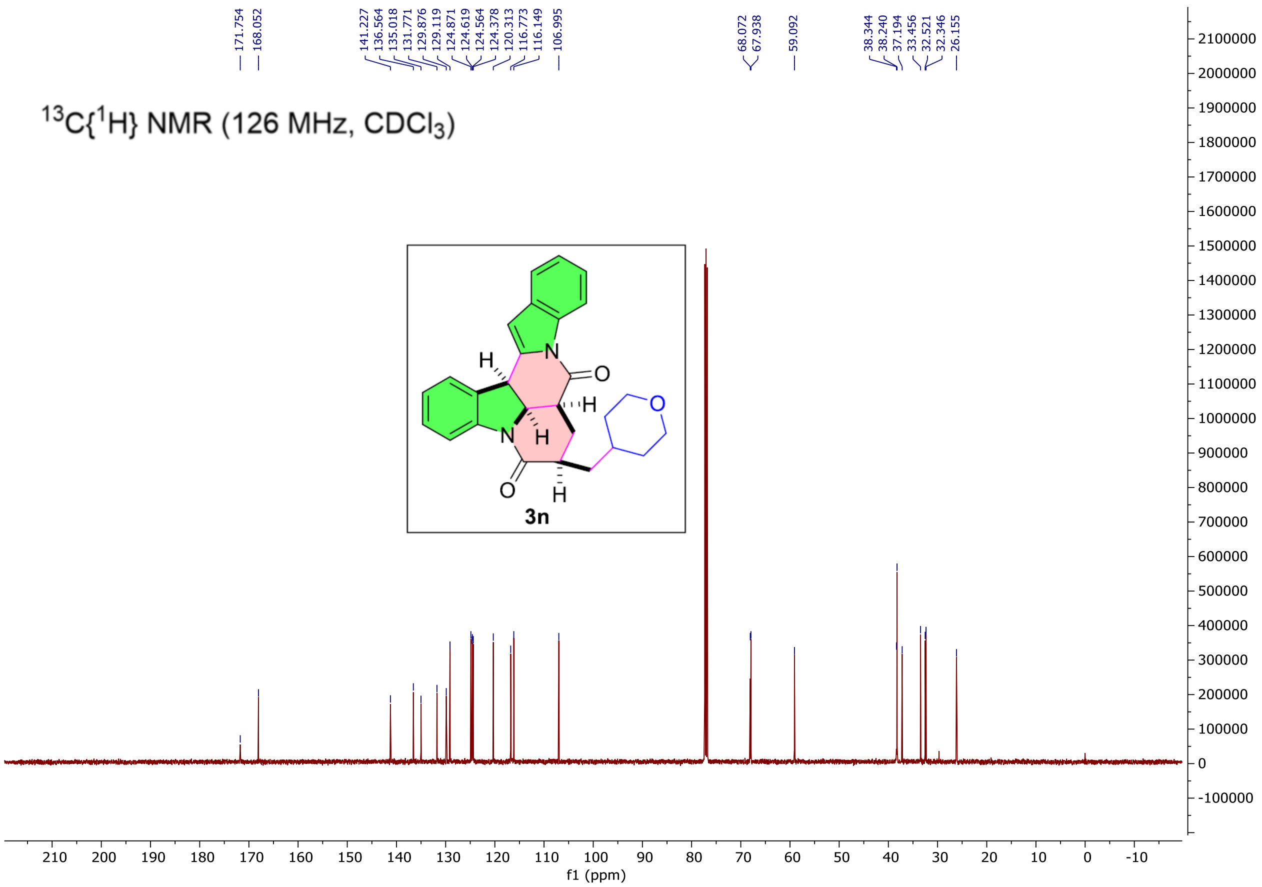

13C{1H} NMR (126 MHz, CDCl3)
171.754 168.052
141.227 136.564 135.018 131.771 129.876 129.119 124.871 124.619 124.564 124.378 120.313 116.773 116.149 106.995
68.072 67.938 59.092
38.344 38.240 37.194 33.456 32.521 32.346 26.155
N
O
H
3n
f1 (ppm)

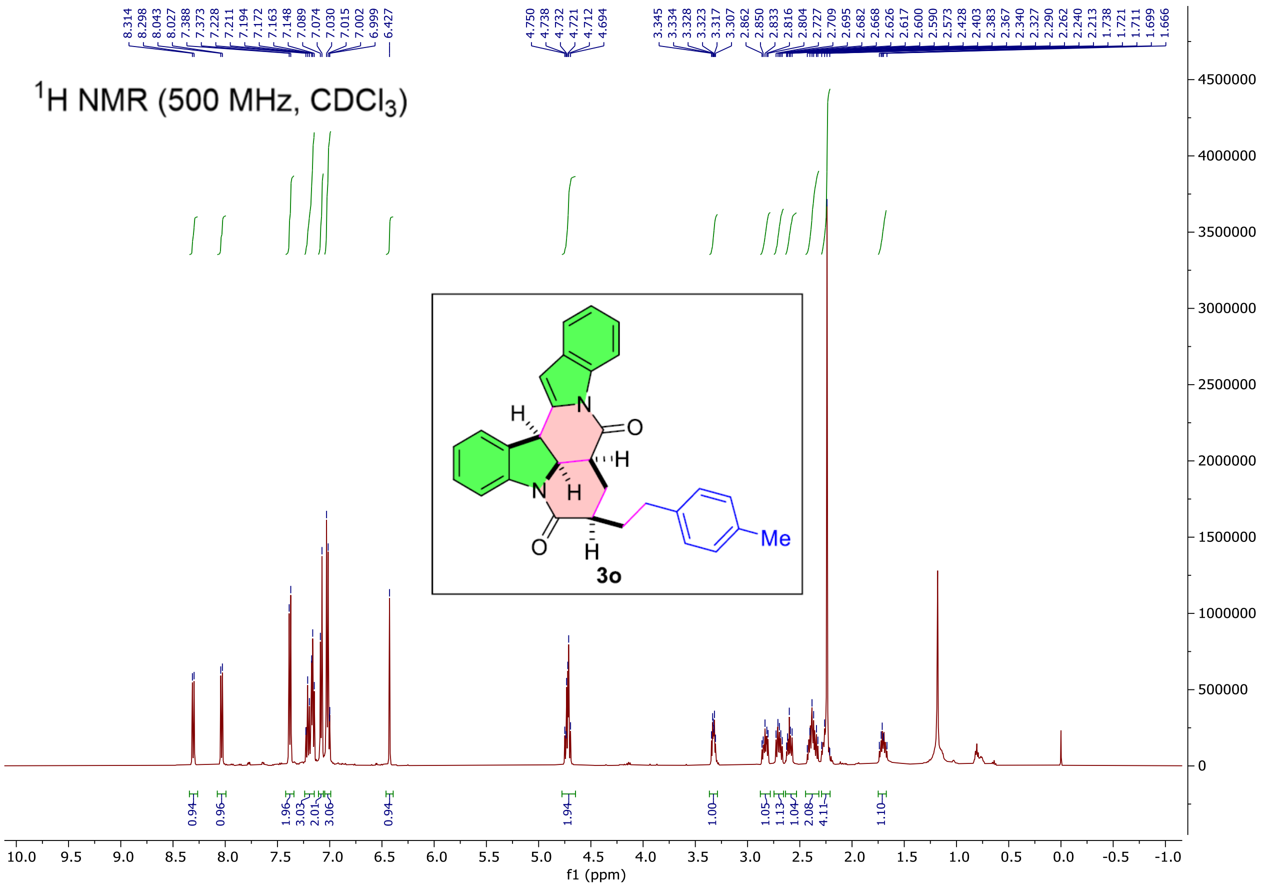

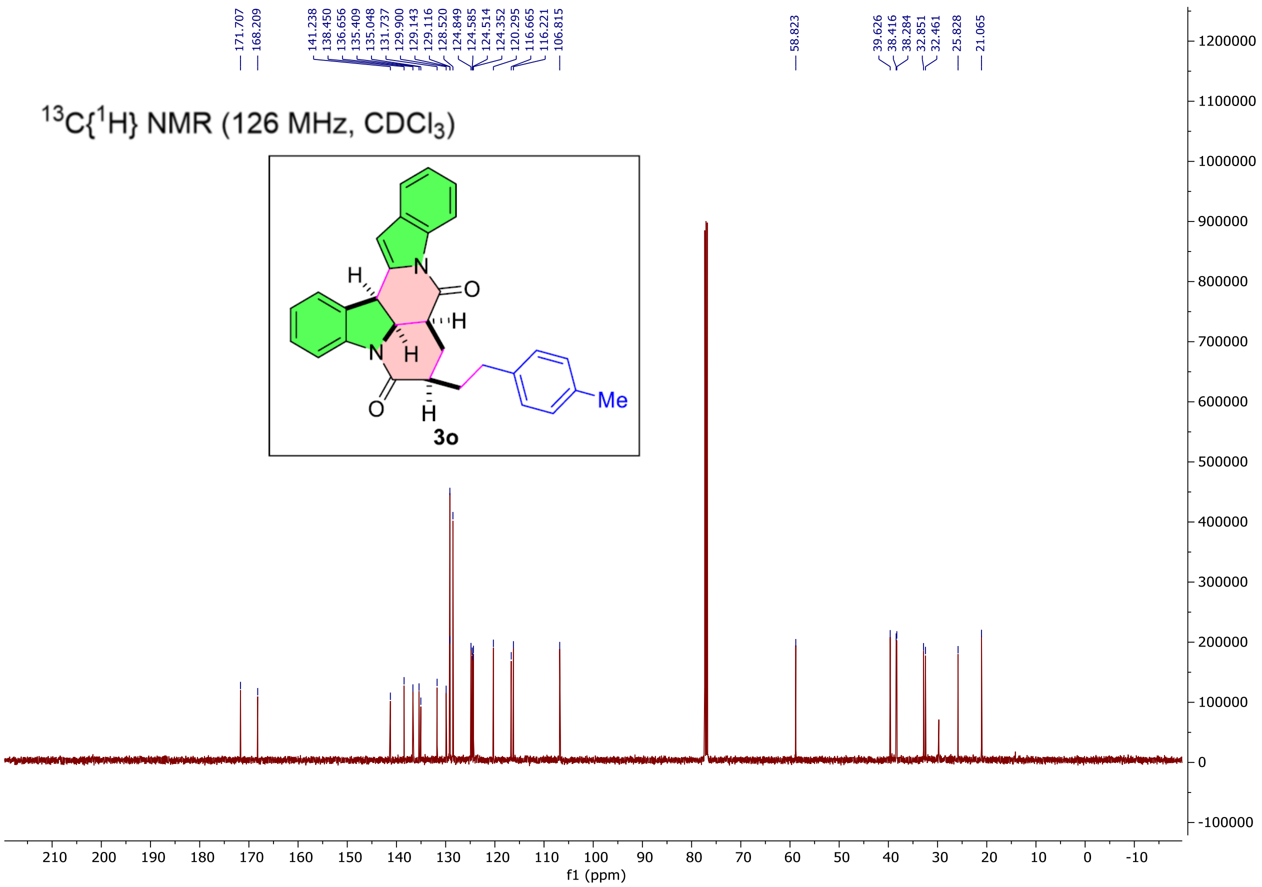

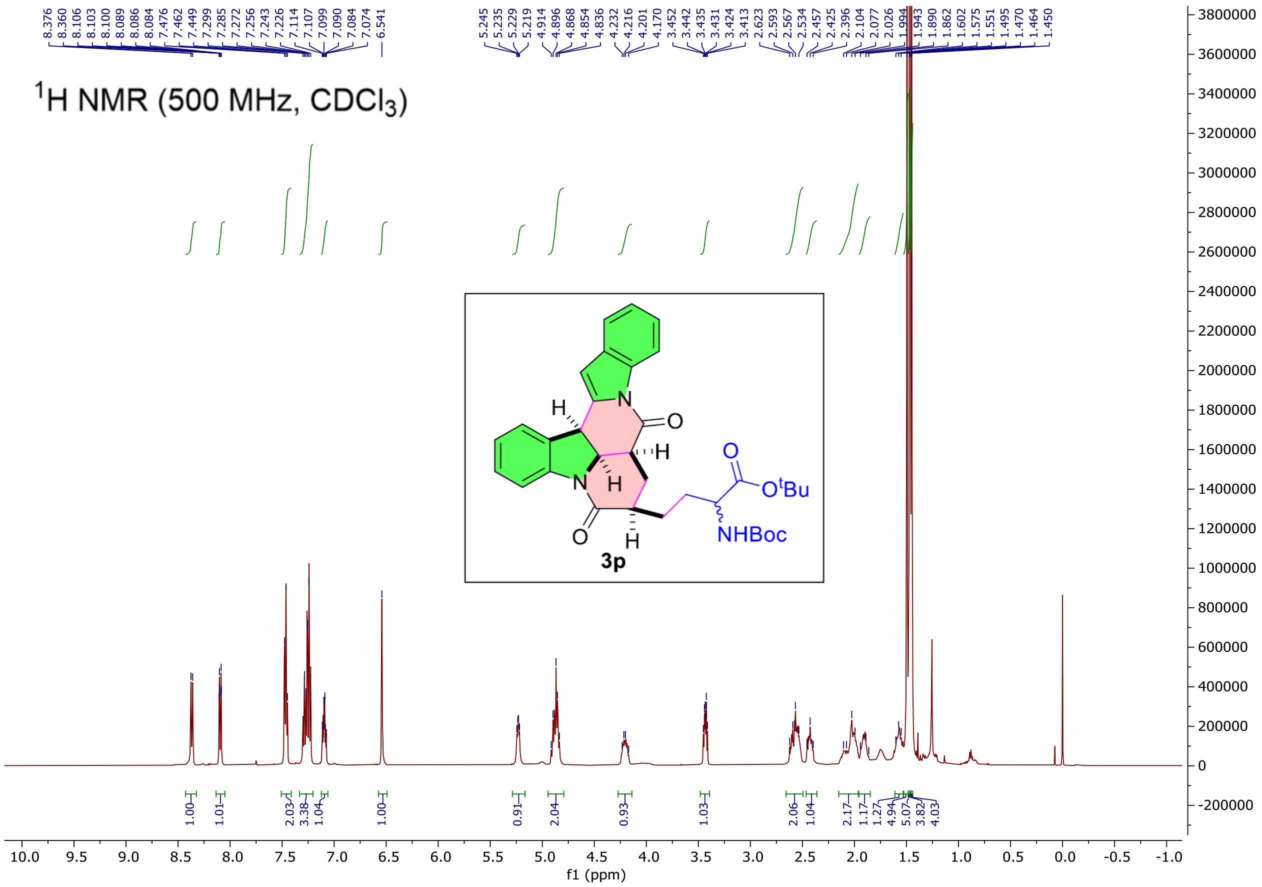

¹H NMR (500 MHz, CDCl₃)
3p
f1 (ppm)

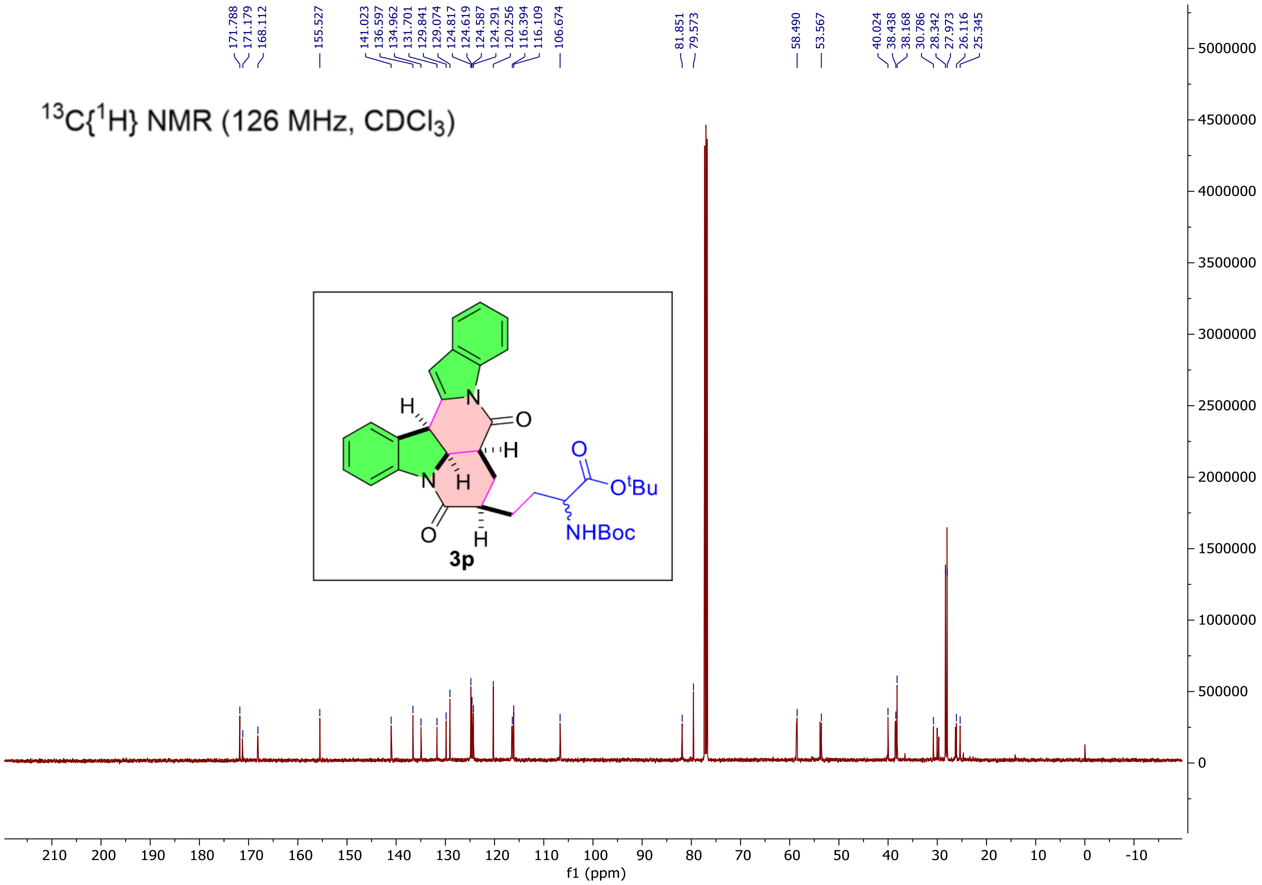

¹³C{¹H} NMR (126 MHz, CDCl₃)
3p
f1 (ppm)

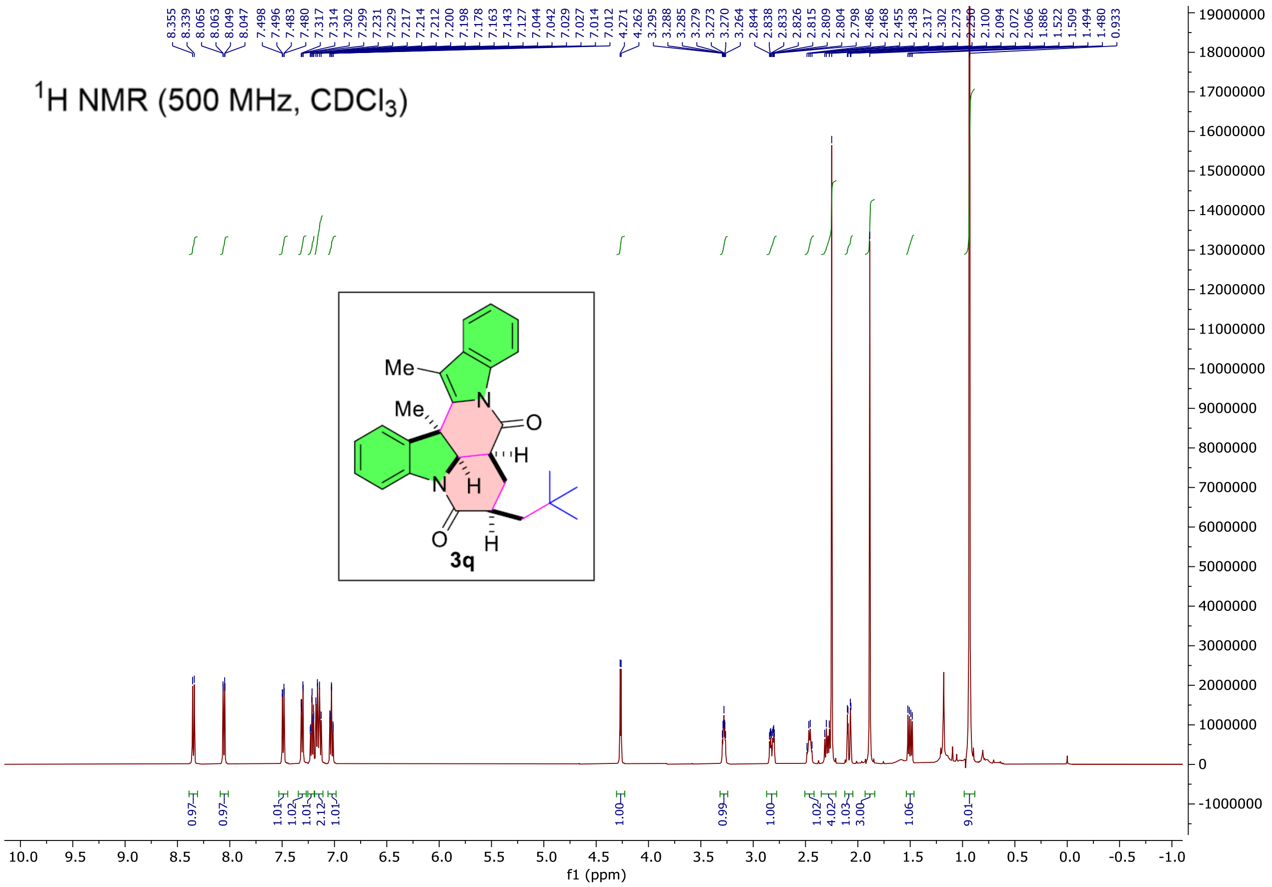

^{1}H NMR (500 MHz, CDCl$_3$)
Me
Me
N
O
H
N
H
O
H
3q
f1 (ppm)

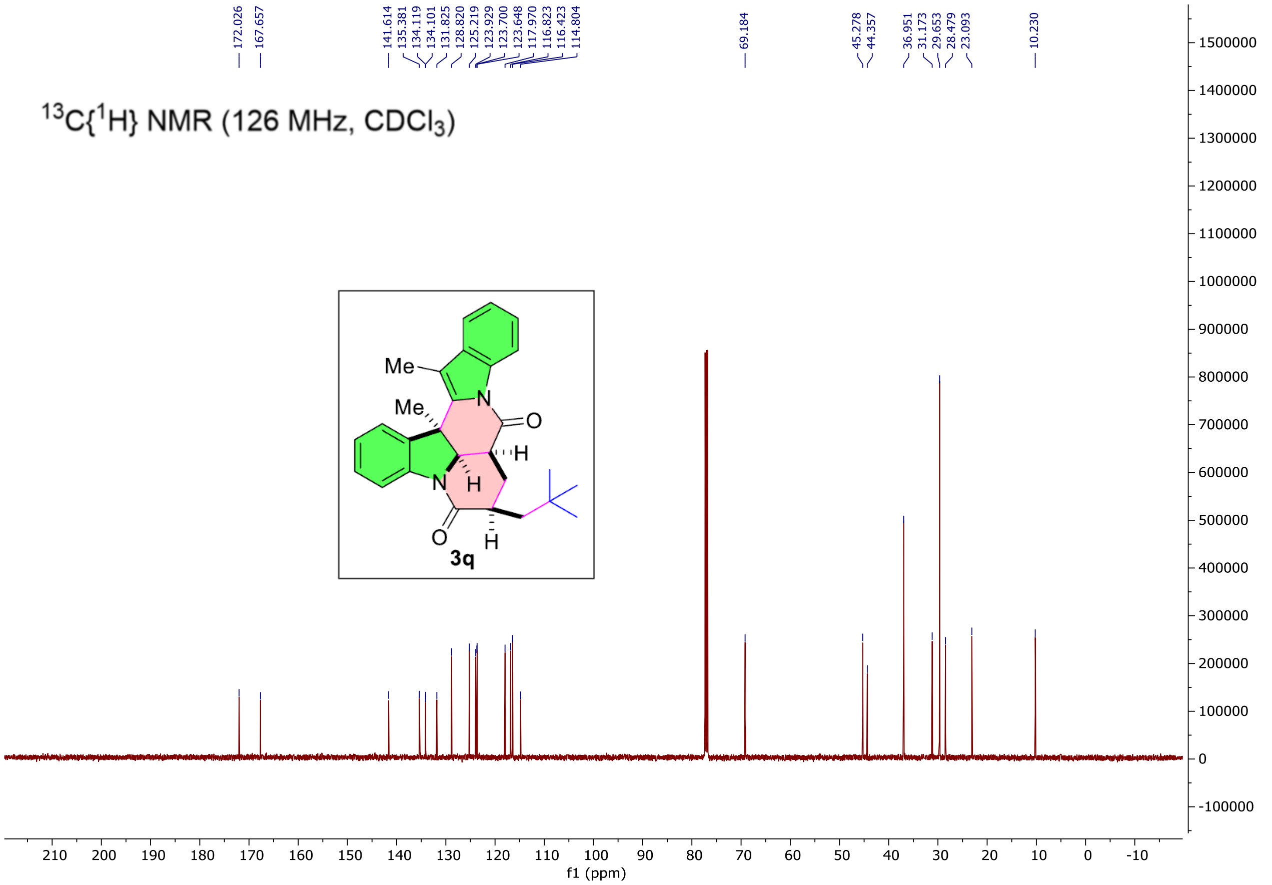

^{13}C{^{1}H} NMR (126 MHz, CDCl$_3$)
172.026
167.657
141.614
135.381
134.119
134.101
131.825
128.820
125.219
123.929
123.700
123.648
117.970
116.823
116.423
114.804
69.184
45.278
44.357
36.951
31.173
29.653
28.479
23.093
10.230
Me
Me
N
O
H
N
H
O
H
3q
f1 (ppm)

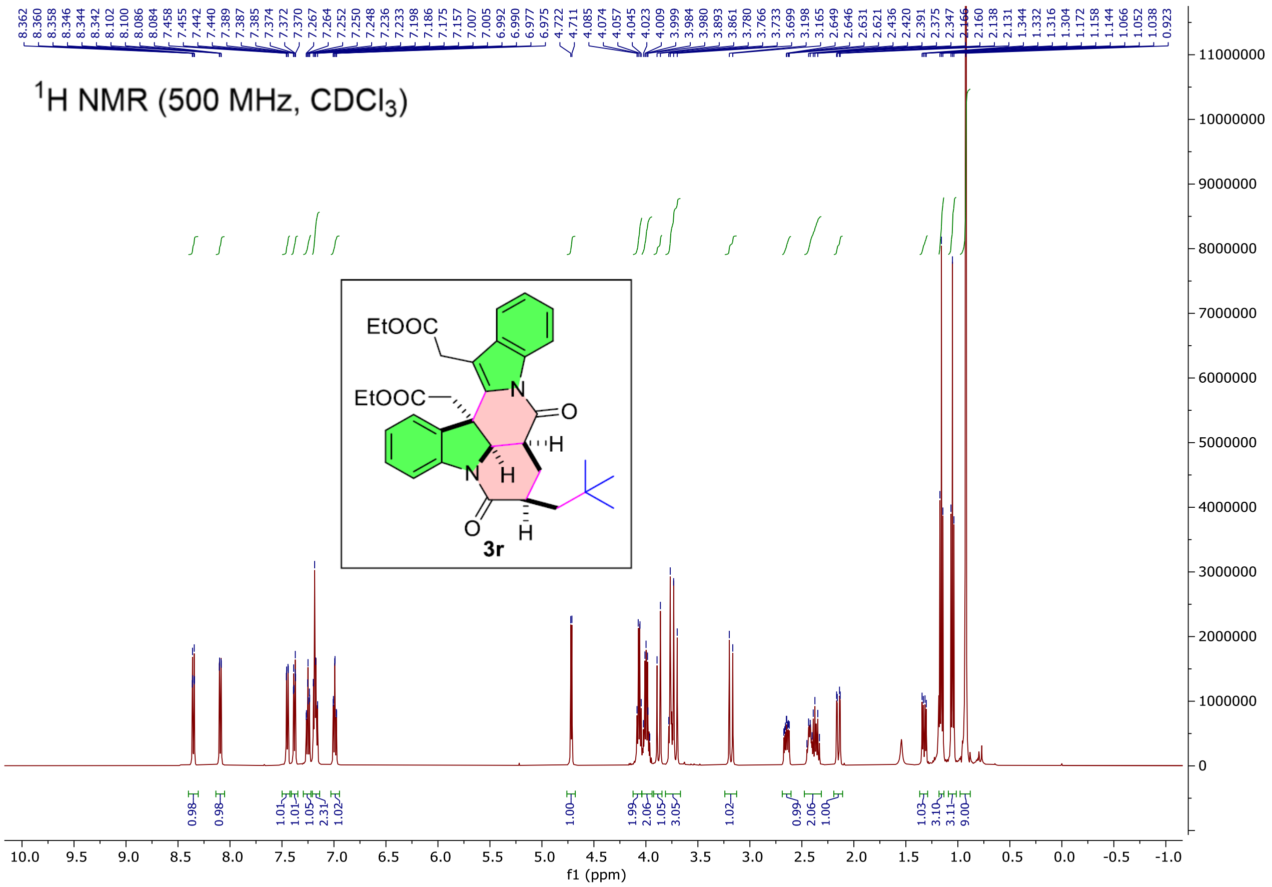
1H NMR (500 MHz, CDCl3)
EtOOC
EtOOC
N
O
H
N
H
O
H
3r
f1 (ppm)

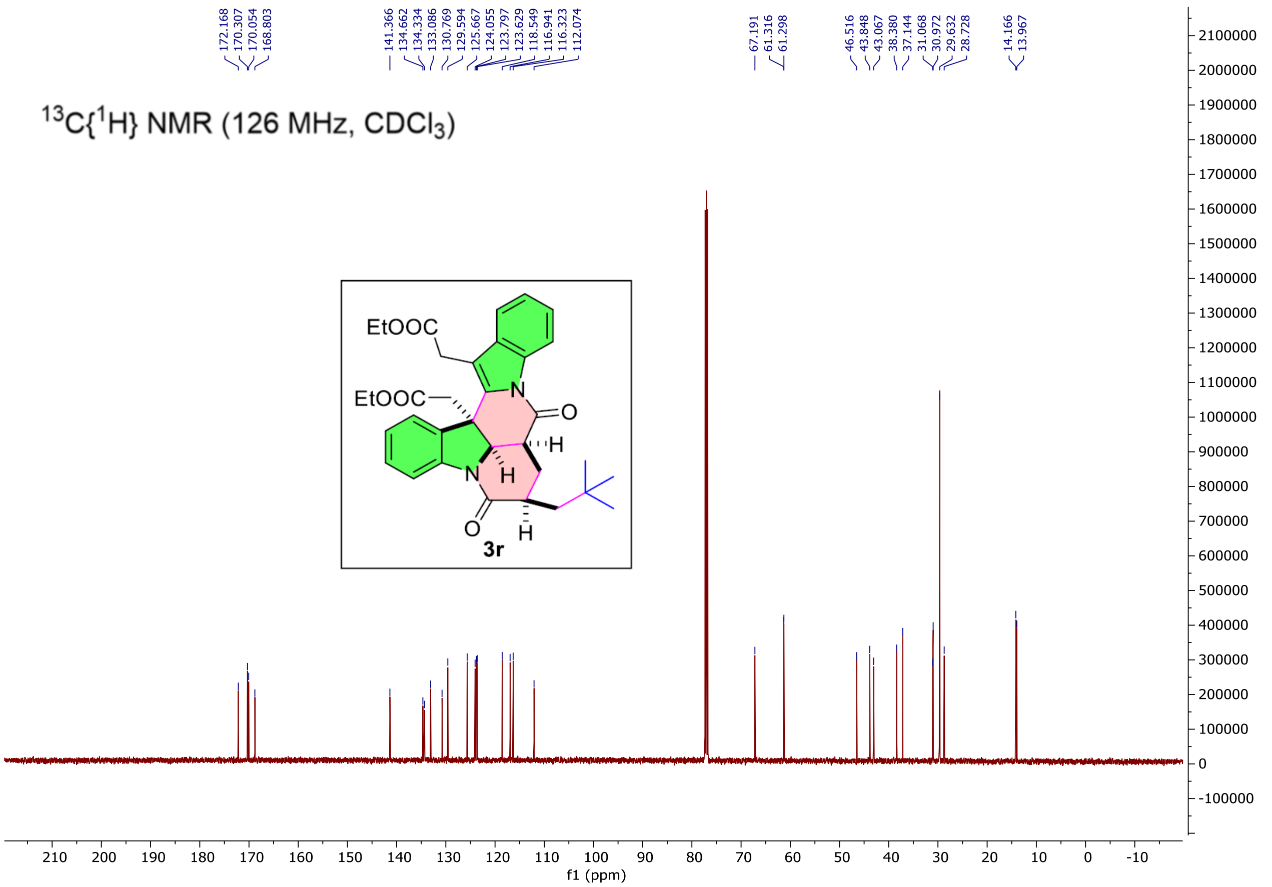
13C{1H} NMR (126 MHz, CDCl3)
172.168
170.307
170.054
168.803
141.366
134.662
134.334
133.086
130.769
129.594
125.667
124.055
123.797
123.629
118.549
116.941
116.323
112.074
67.191
61.316
61.298
46.516
43.848
43.067
38.380
37.144
31.068
30.972
29.632
28.728
14.166
13.967
EtOOC
EtOOC
N
O
H
N
H
O
H
3r
f1 (ppm)

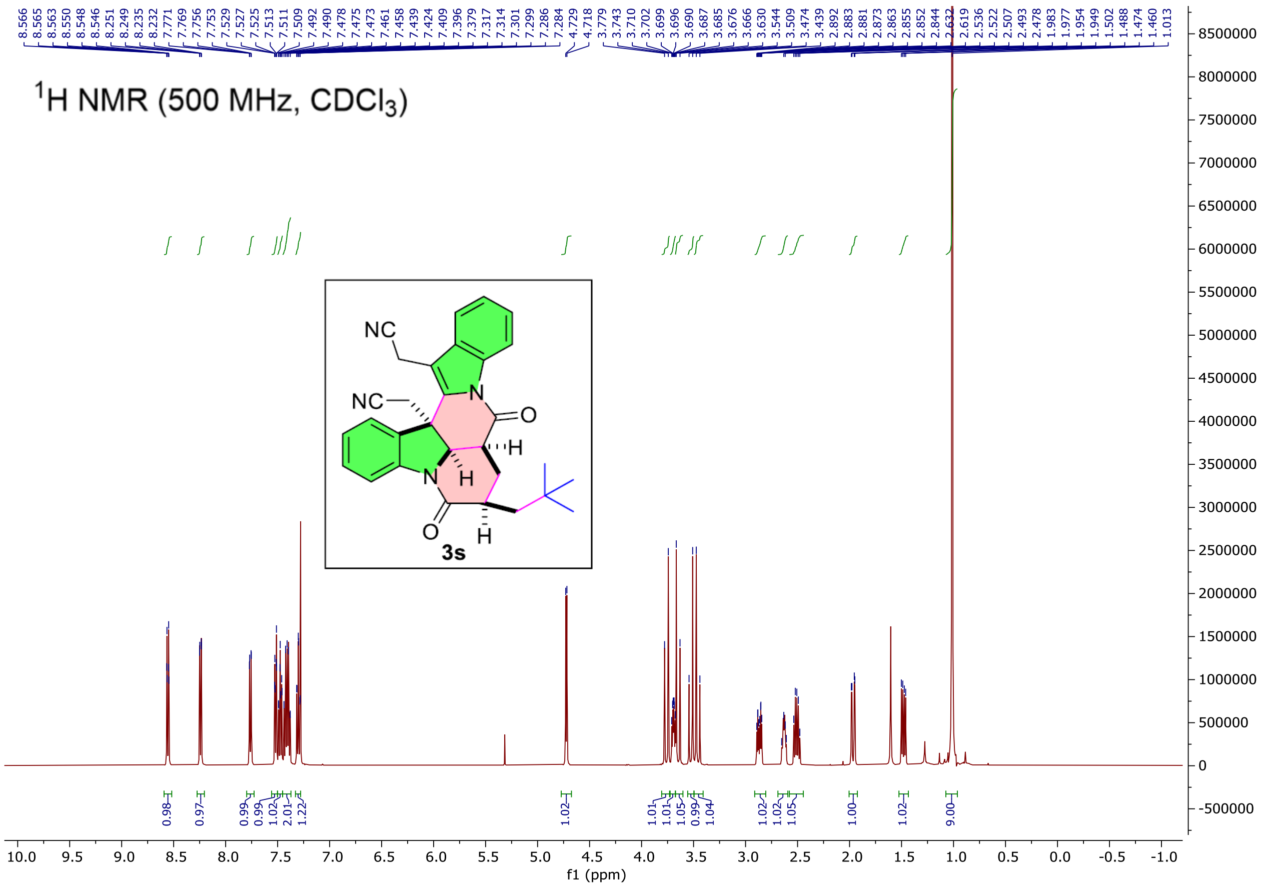

1H NMR (500 MHz, CDCl3)
NC
NC
N
O
H
N
H
O
H
3s
f1 (ppm)

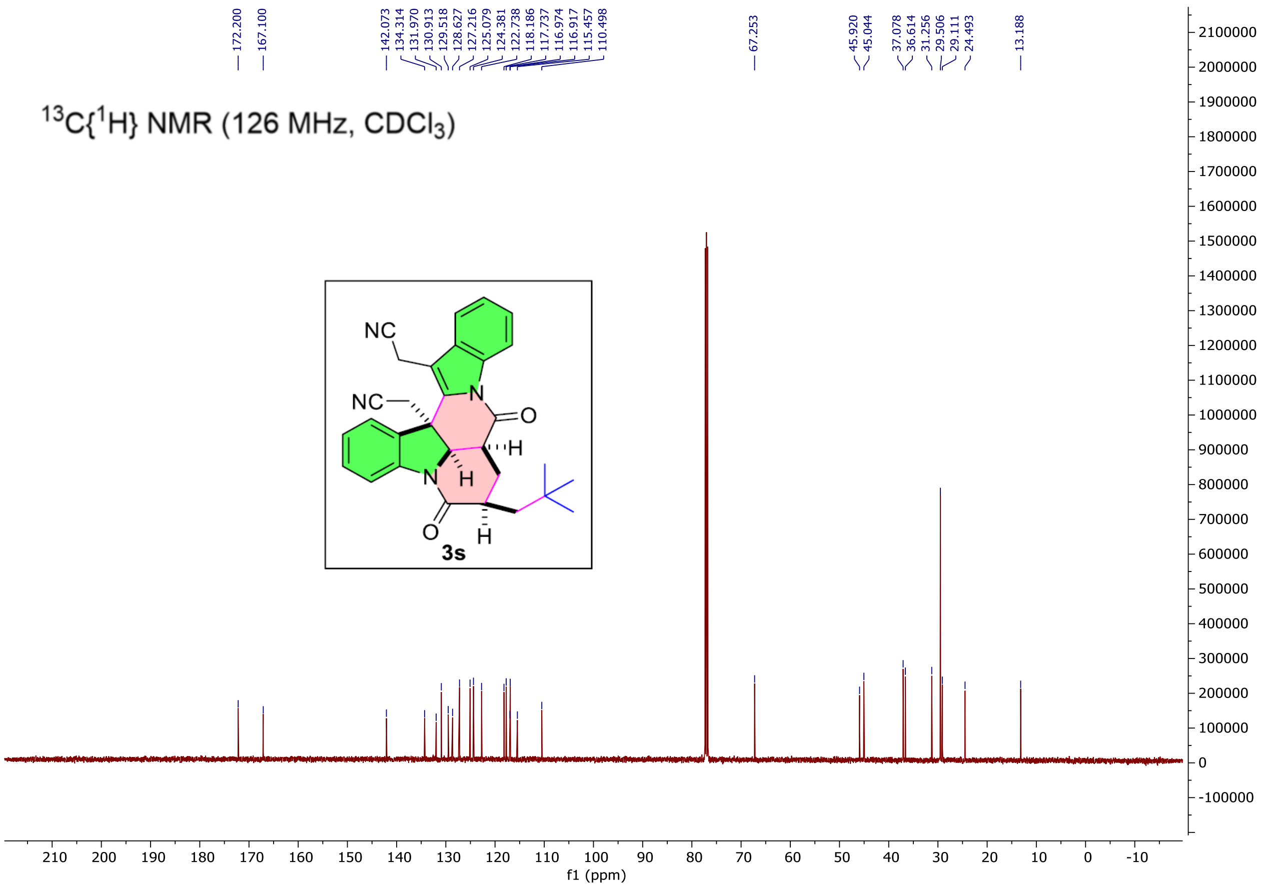

13C{1H} NMR (126 MHz, CDCl3)
NC
NC
N
O
H
N
H
O
H
3s
f1 (ppm)

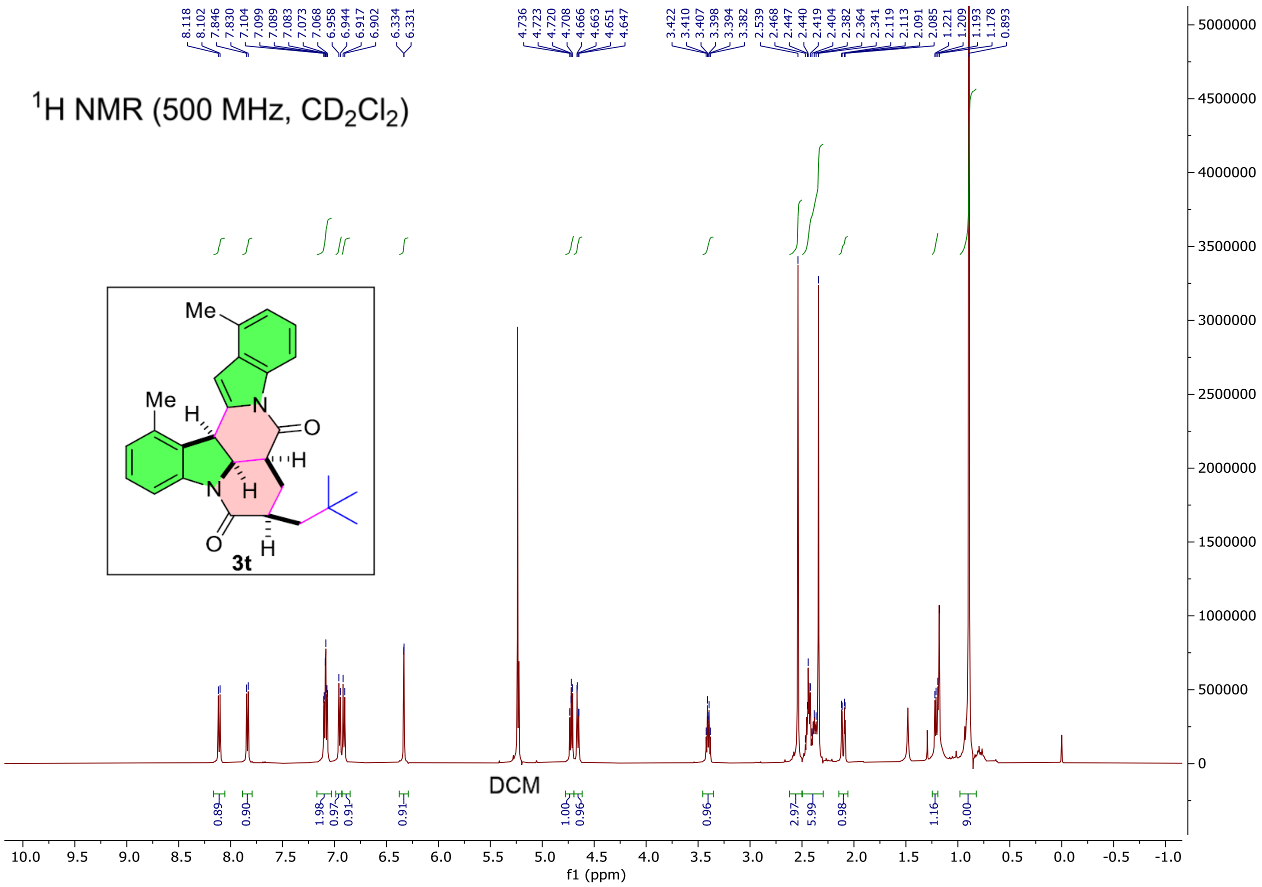
^{1}H NMR (500 MHz, CD_2Cl_2)
DCM
3t
f1 (ppm)

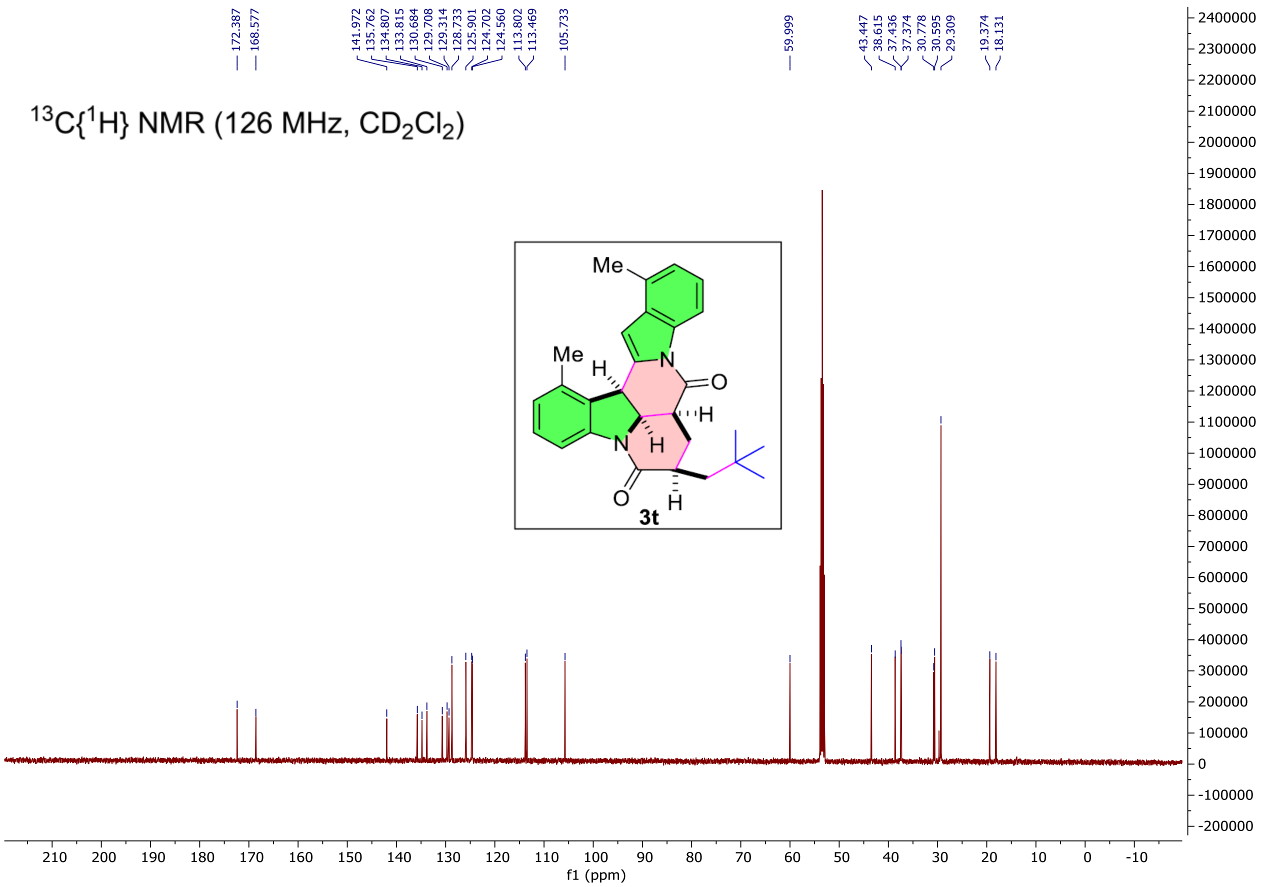
^{13}C{^{1}H} NMR (126 MHz, CD_2Cl_2)
3t
f1 (ppm)

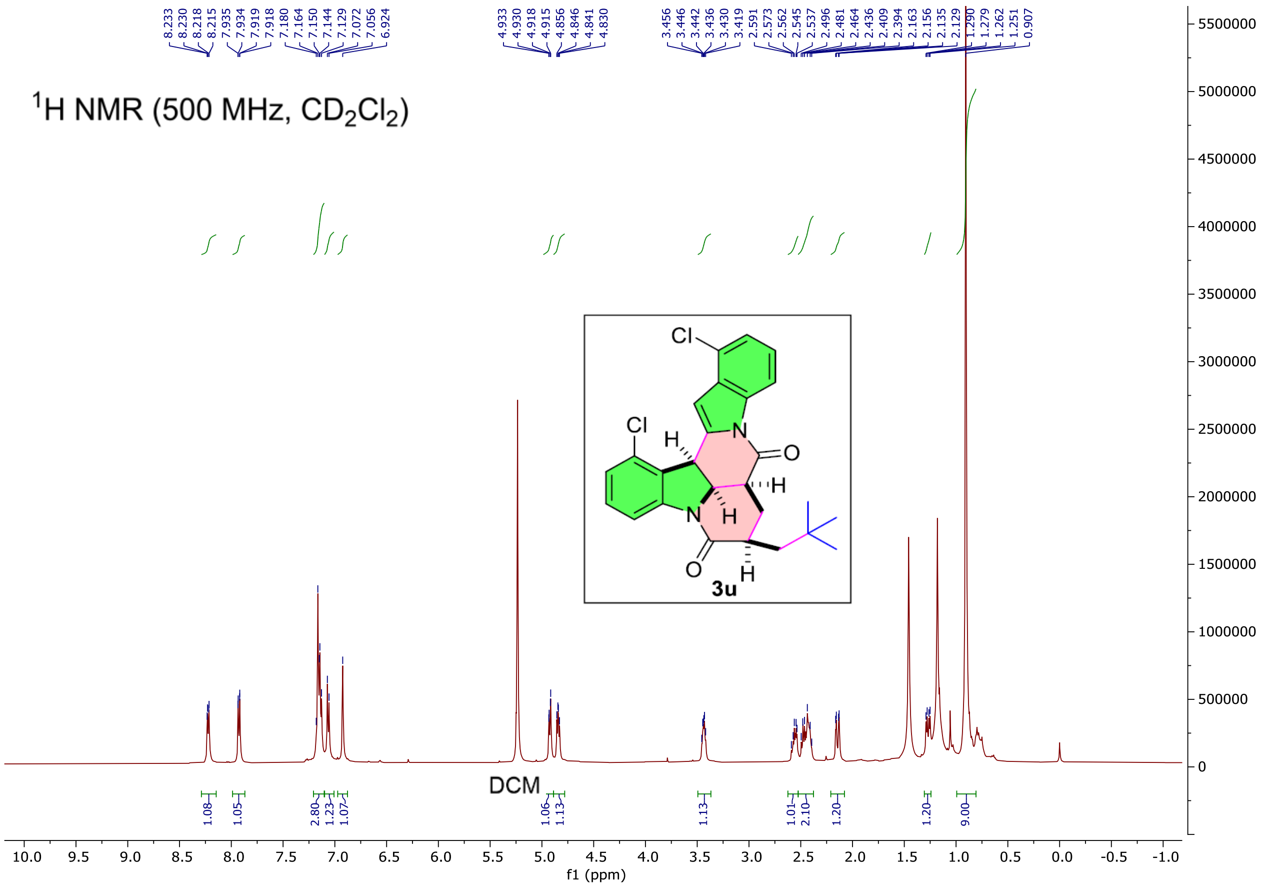

8.233 8.230 8.218 8.215 7.935 7.934 7.919 7.918 7.180 7.164 7.150 7.144 7.129 7.072 7.056 6.924
4.933 4.930 4.918 4.915 4.856 4.846 4.841 4.830
3.456 3.446 3.442 3.436 3.430 3.419 2.591 2.573 2.562 2.545 2.537 2.496 2.481 2.464 2.436 2.409 2.394 2.163 2.156 2.135 2.129 1.290 1.279 1.262 1.251 0.907
^{1}H NMR (500 MHz, CD_2Cl_2)
Cl
Cl
H
N
O
H
N
H
O
H
3u
DCM
1.08 1.05 2.80 1.23 1.07 1.06 1.13 1.13 1.01 2.10 1.20 1.20 9.00
f1 (ppm)

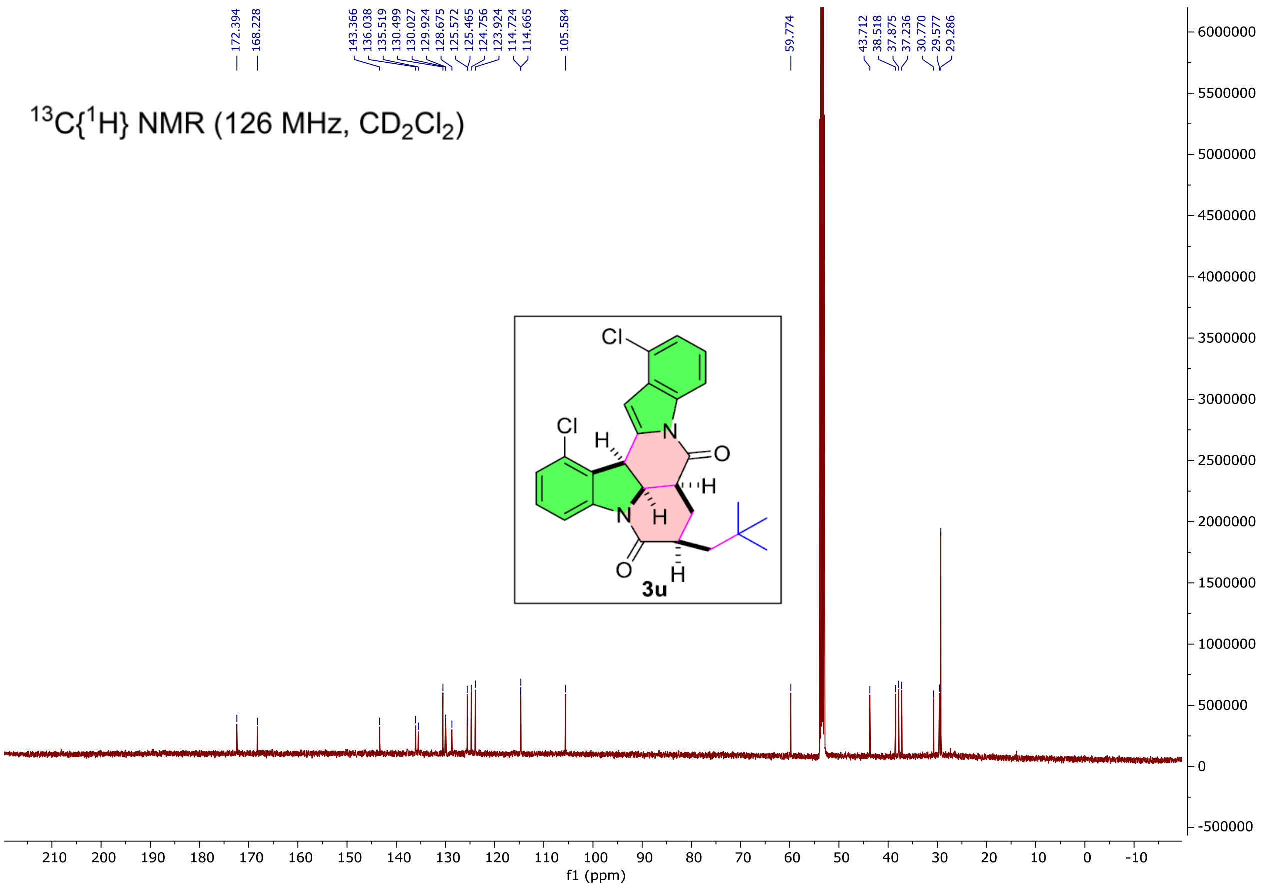

172.394 168.228
143.366 136.038 135.519 130.499 130.027 129.924 128.675 125.572 125.465 124.756 123.924 114.724 114.665 105.584
59.774
43.712 38.518 37.875 37.236 30.770 29.577 29.286
^{13}C{^{1}H} NMR (126 MHz, CD_2Cl_2)
Cl
Cl
H
N
O
H
N
H
O
H
3u
f1 (ppm)

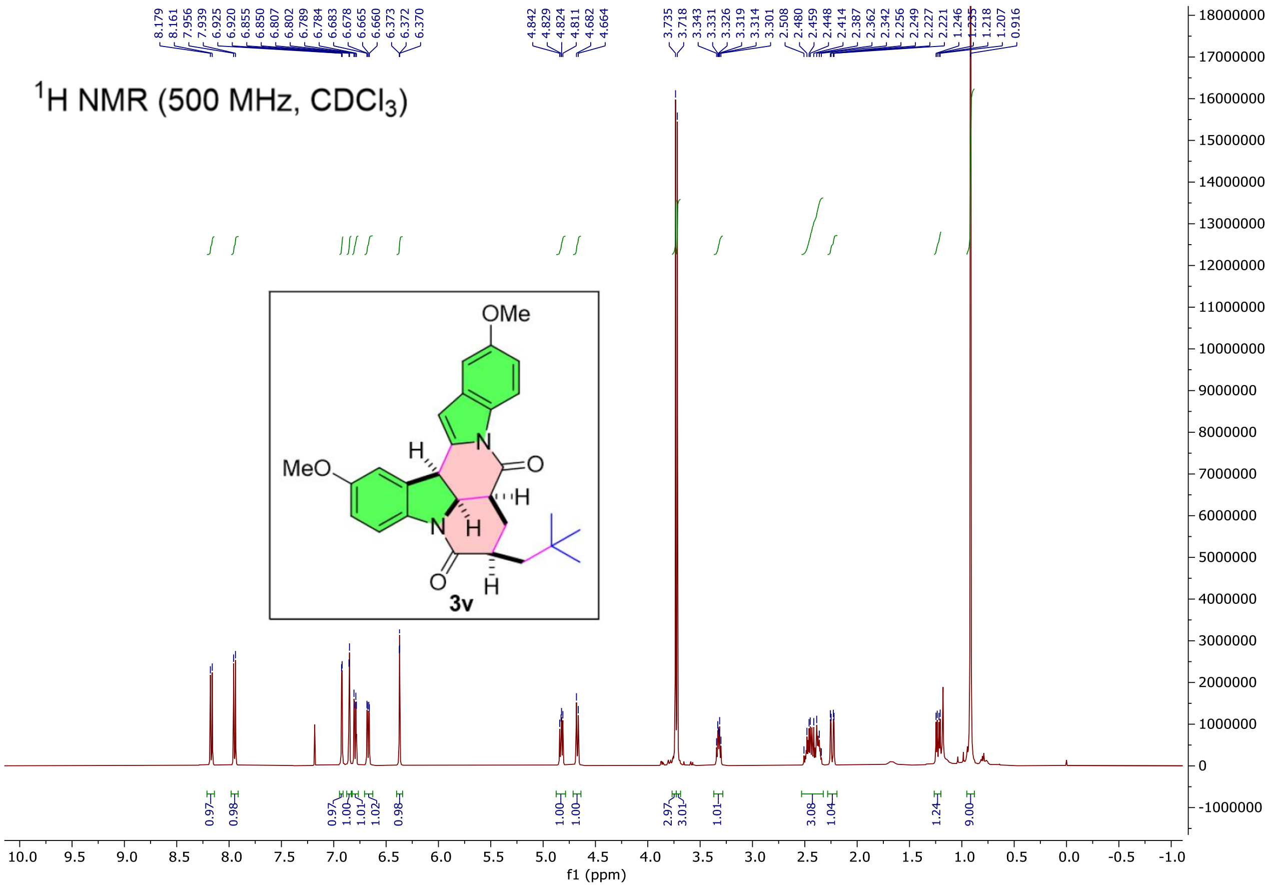

8.179
8.161
7.956
7.939
6.925
6.920
6.855
6.850
6.807
6.802
6.789
6.784
6.683
6.678
6.665
6.660
6.373
6.372
6.370
4.842
4.829
4.824
4.811
4.682
4.664
3.735
3.718
3.343
3.331
3.326
3.319
3.314
3.301
2.508
2.480
2.459
2.448
2.414
2.387
2.362
2.342
2.256
2.249
2.227
2.221
1.246
1.235
1.218
1.207
0.916
^{1}H NMR (500 MHz, CDCl$_3$)
OMe
MeO
H
N
O
H
N
H
O
H
3v
f1 (ppm)

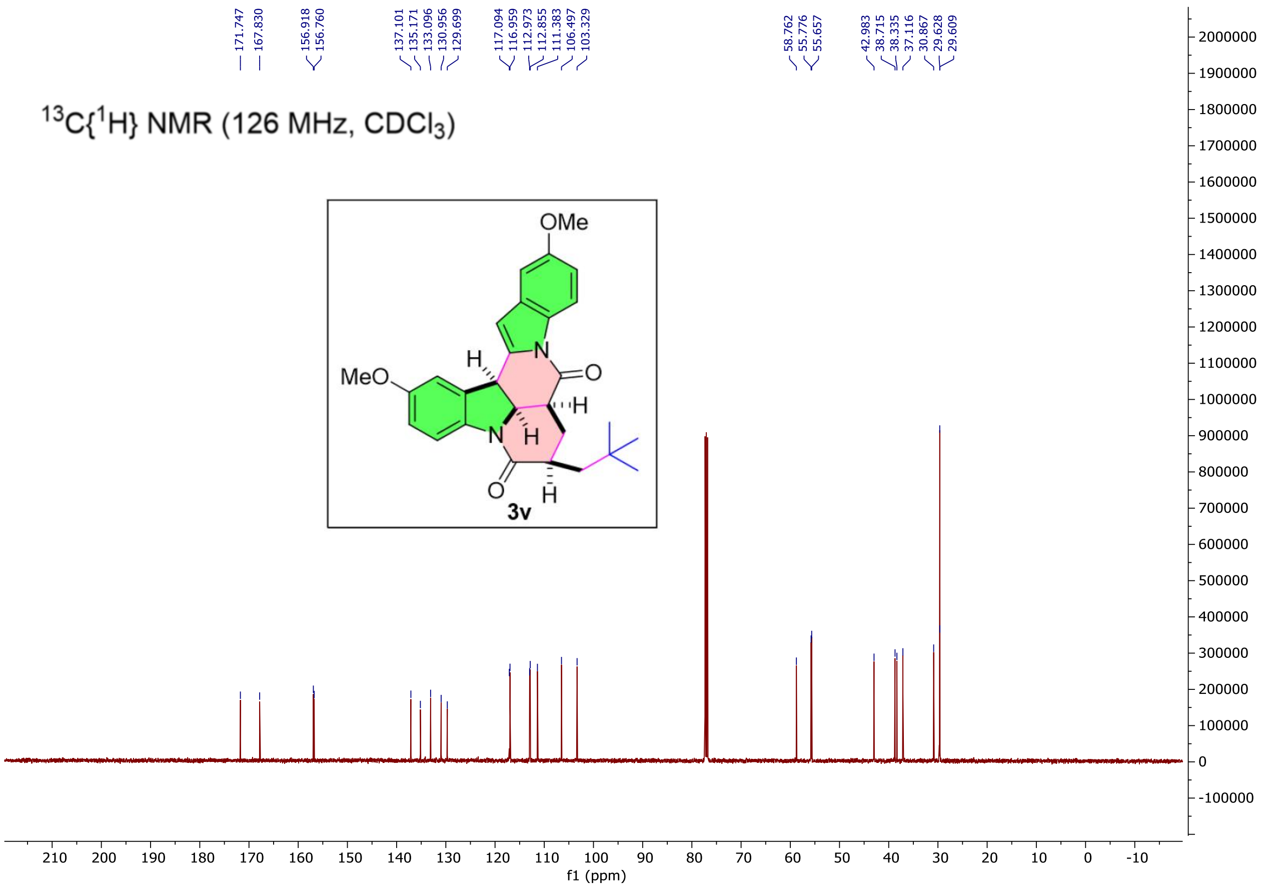

171.747
167.830
156.918
156.760
137.101
135.171
133.096
130.956
129.699
117.094
116.959
112.973
112.855
111.383
106.497
103.329
58.762
55.776
55.657
42.983
38.715
38.335
37.116
30.867
29.628
29.609
^{13}C{^{1}H} NMR (126 MHz, CDCl$_3$)
OMe
MeO
H
N
O
H
N
H
O
H
3v
f1 (ppm)

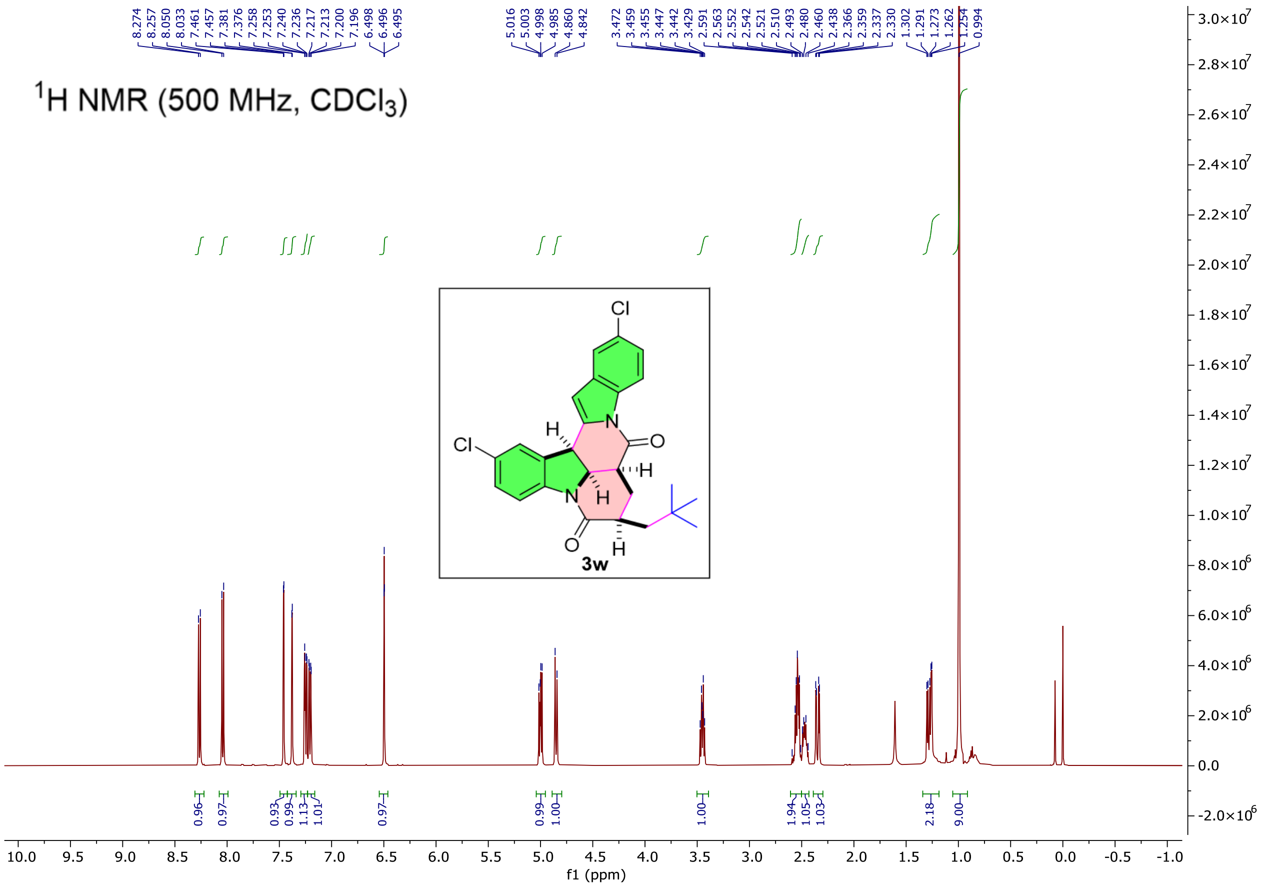
1H NMR (500 MHz, CDCl3)
8.274 8.257 8.050 8.033 7.461 7.457 7.381 7.376 7.258 7.253 7.240 7.236 7.217 7.213 7.200 7.196 6.498 6.496 6.495
5.016 5.003 4.998 4.985 4.860 4.842
3.472 3.459 3.455 3.447 3.442 3.429 2.591 2.563 2.552 2.542 2.521 2.510 2.493 2.480 2.460 2.438 2.366 2.359 2.337 2.330 1.302 1.291 1.273 1.262 1.254 0.994
0.96 0.97 0.93 0.99 1.13 1.01 0.97 0.99 1.00 1.00 1.94 1.05 1.03 2.18 9.00
Cl
Cl
N
N
H
H
H
H
O
O
3w
f1 (ppm)

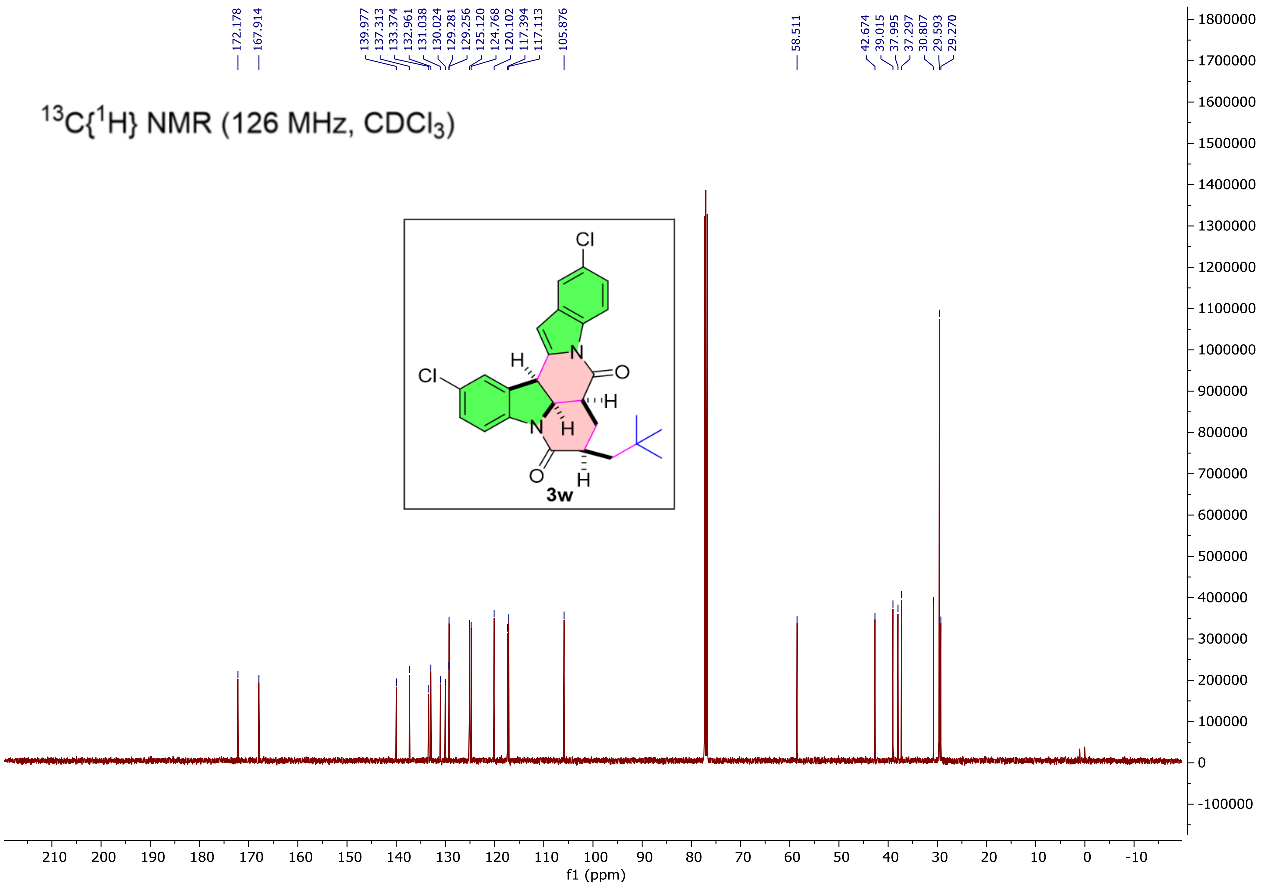
13C{1H} NMR (126 MHz, CDCl3)
172.178 167.914
139.977 137.313 133.374 132.961 131.038 130.024 129.281 129.256 125.120 124.768 120.102 117.394 117.113 105.876
58.511
42.674 39.015 37.995 37.297 30.807 29.593 29.270
Cl
Cl
N
N
H
H
H
H
O
O
3w
f1 (ppm)

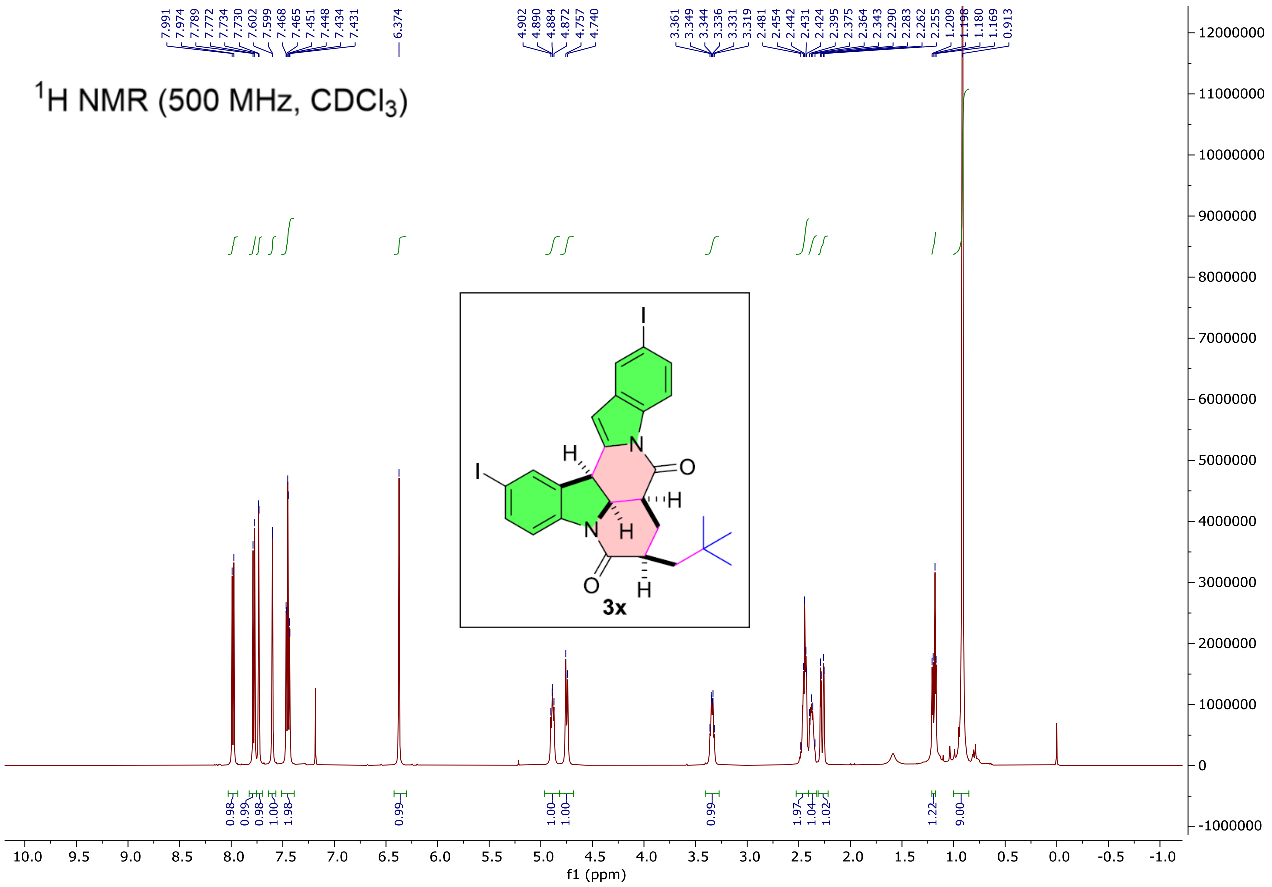

^{1}H NMR (500 MHz, $CDCl_3$)
3x
f1 (ppm)

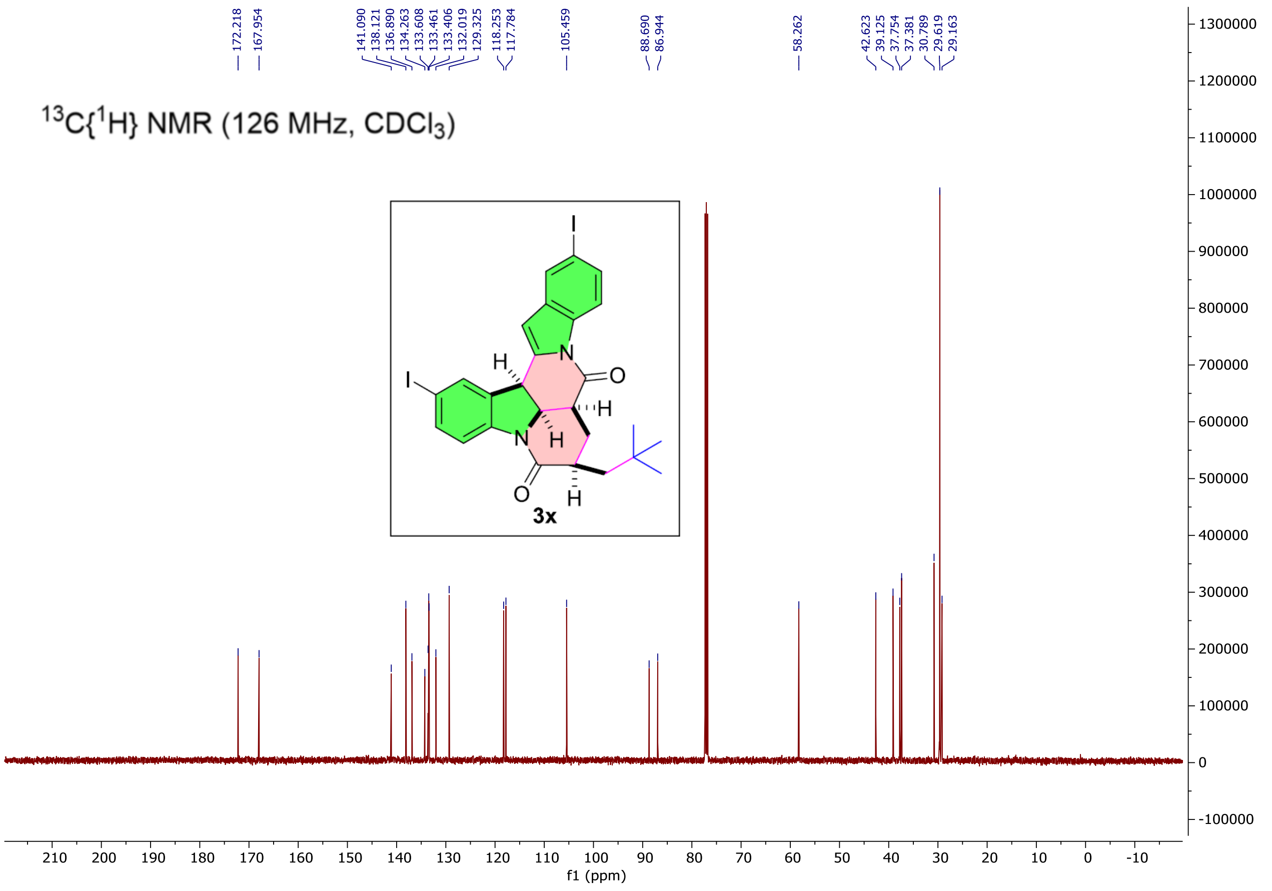

^{13}C{^{1}H} NMR (126 MHz, $CDCl_3$)
3x
f1 (ppm)

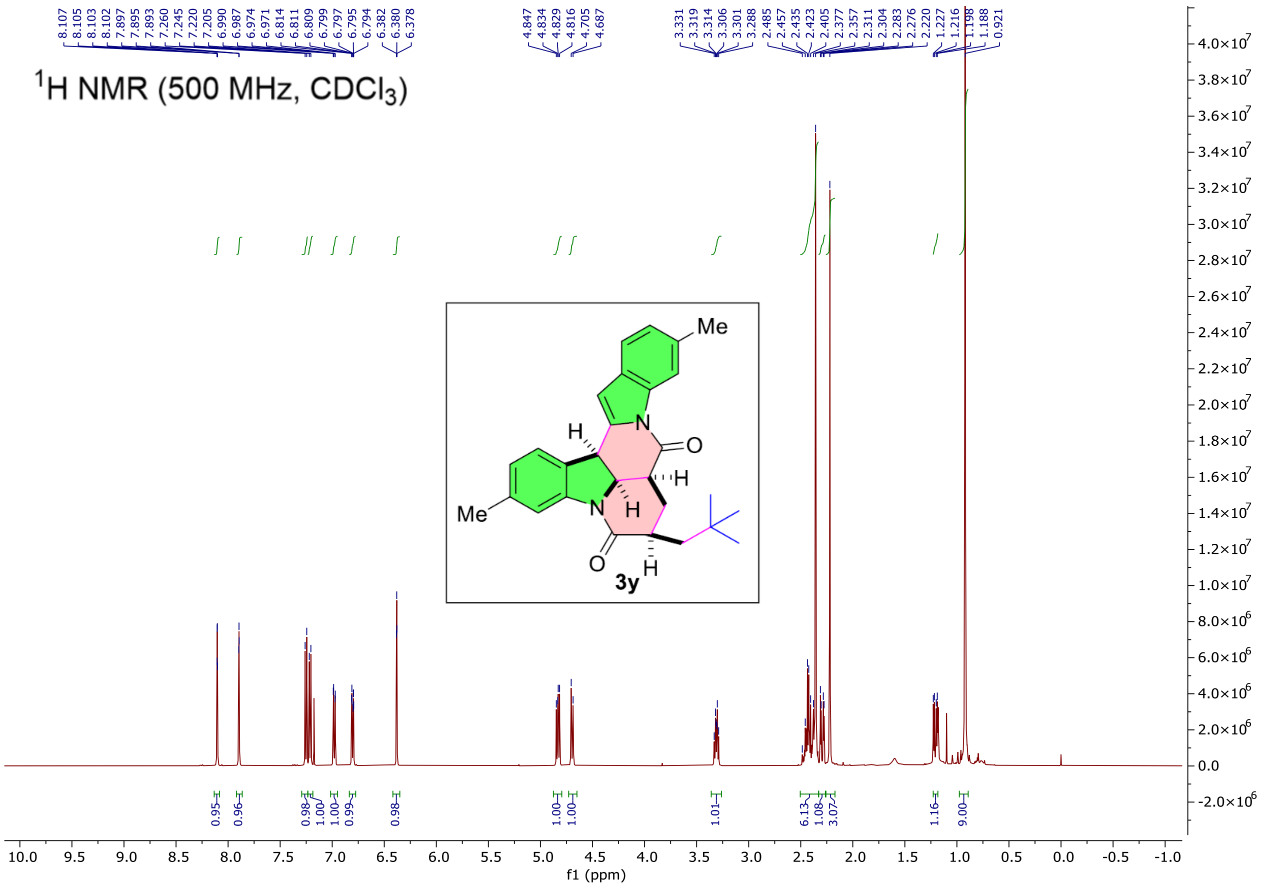

^{1}H NMR (500 MHz, $CDCl_3$)
Me
H
N
O
H
Me
N
H
O
H
3y
f1 (ppm)

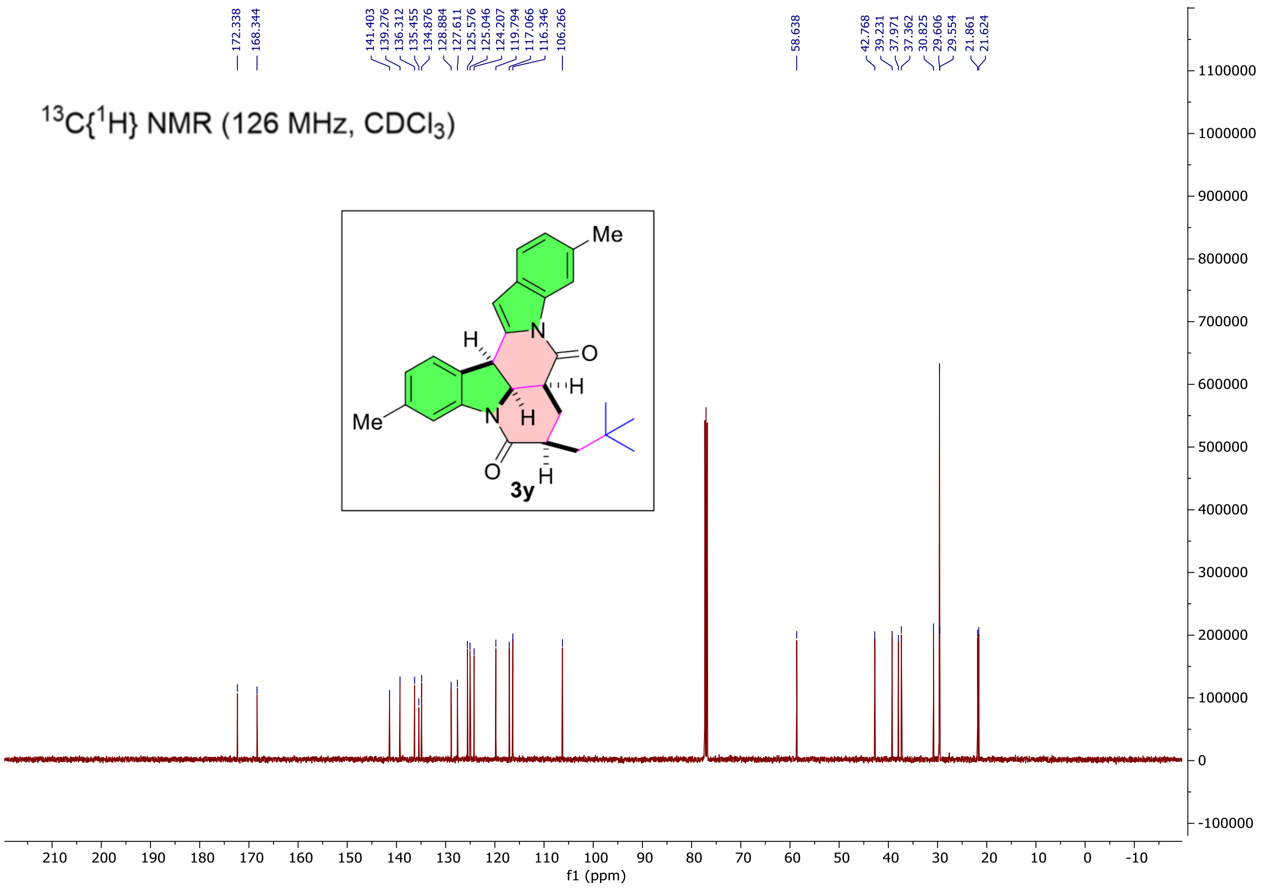

^{13}C{^{1}H} NMR (126 MHz, $CDCl_3$)
172.338
168.344
141.403
139.276
136.312
135.455
134.876
128.884
127.611
125.576
125.046
124.207
119.794
117.066
116.346
106.266
58.638
42.768
39.231
37.971
37.362
30.825
29.606
29.554
21.861
21.624
Me
H
N
O
H
Me
N
H
O
H
3y
f1 (ppm)

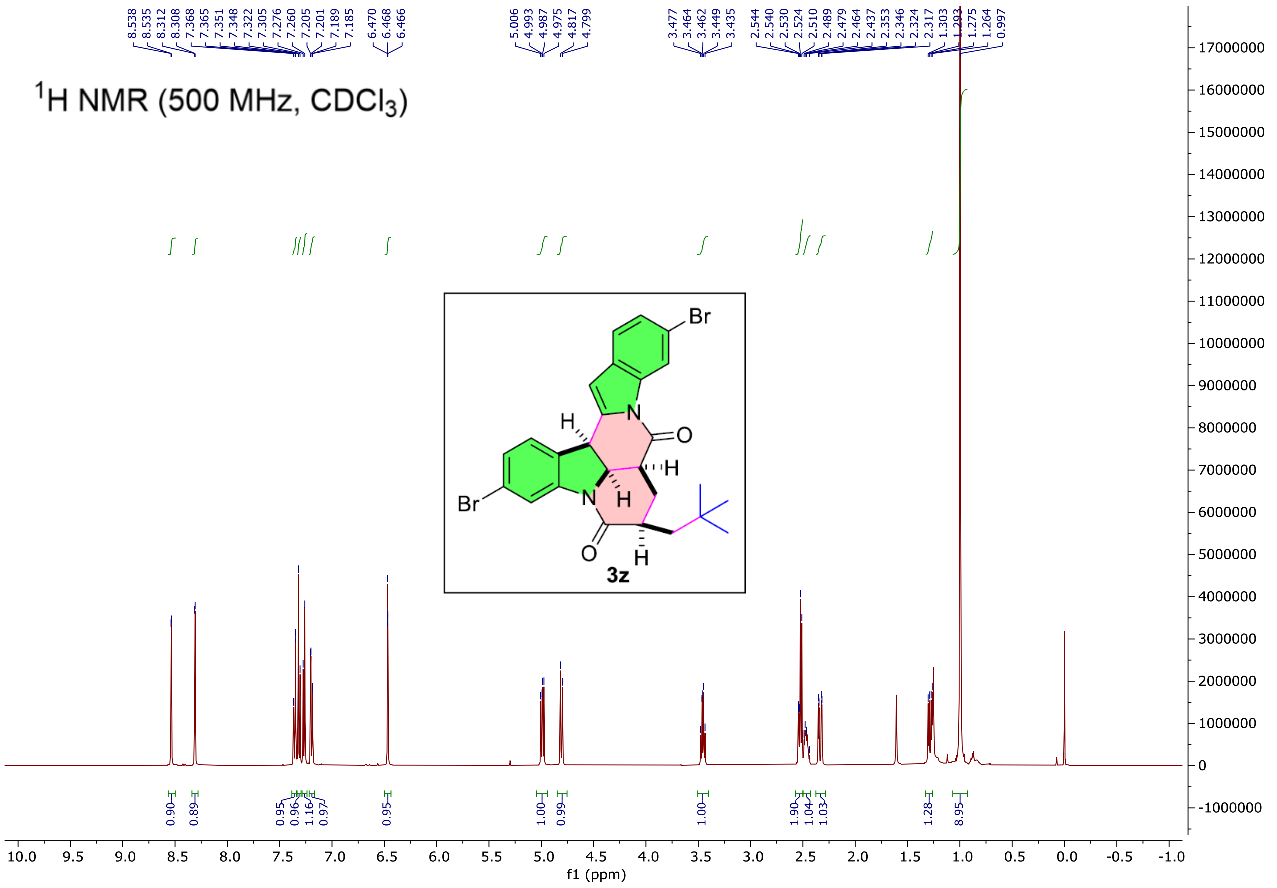

1H NMR (500 MHz, CDCl3)
8.538 8.535 8.312 8.308 7.368 7.365 7.351 7.348 7.322 7.305 7.276 7.260 7.205 7.201 7.189 7.185 6.470 6.468 6.466
5.006 4.993 4.987 4.975 4.817 4.799
3.477 3.464 3.462 3.449 3.435 2.544 2.540 2.530 2.524 2.510 2.489 2.479 2.464 2.437 2.353 2.346 2.324 2.317 1.303 1.293 1.275 1.264 0.997
0.90 0.89 0.95 0.96 1.16 0.97 0.95 1.00 0.99 1.00 1.90 1.04 1.03 1.28 8.95
Br
N
O
H
Br
N
H
O
H
H
3z
f1 (ppm)

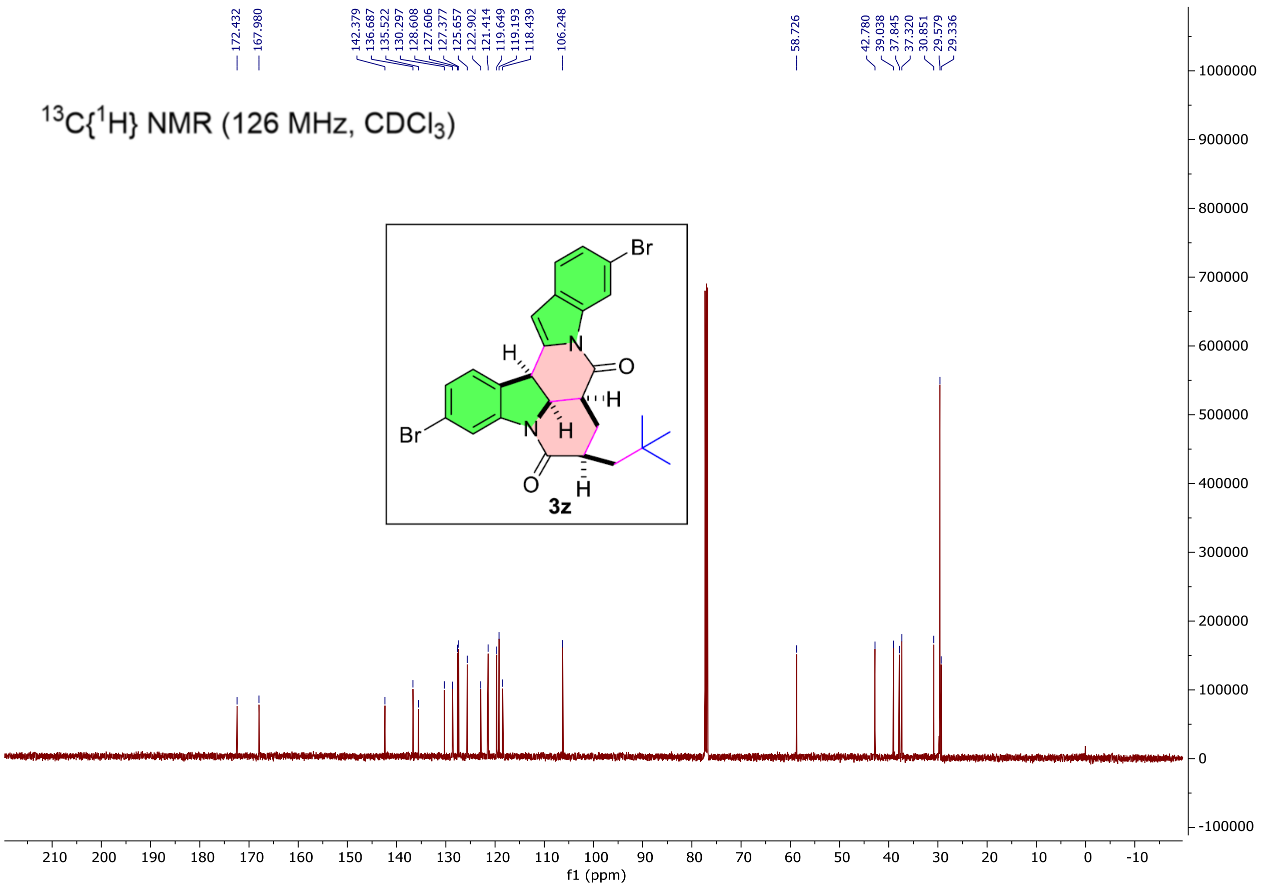

13C{1H} NMR (126 MHz, CDCl3)
172.432 167.980
142.379 136.687 135.522 130.297 128.608 127.606 127.377 125.657 122.902 121.414 119.649 119.193 118.439
106.248
58.726
42.780 39.038 37.845 37.320 30.851 29.579 29.336
Br
N
O
H
Br
N
H
O
H
H
3z
f1 (ppm)

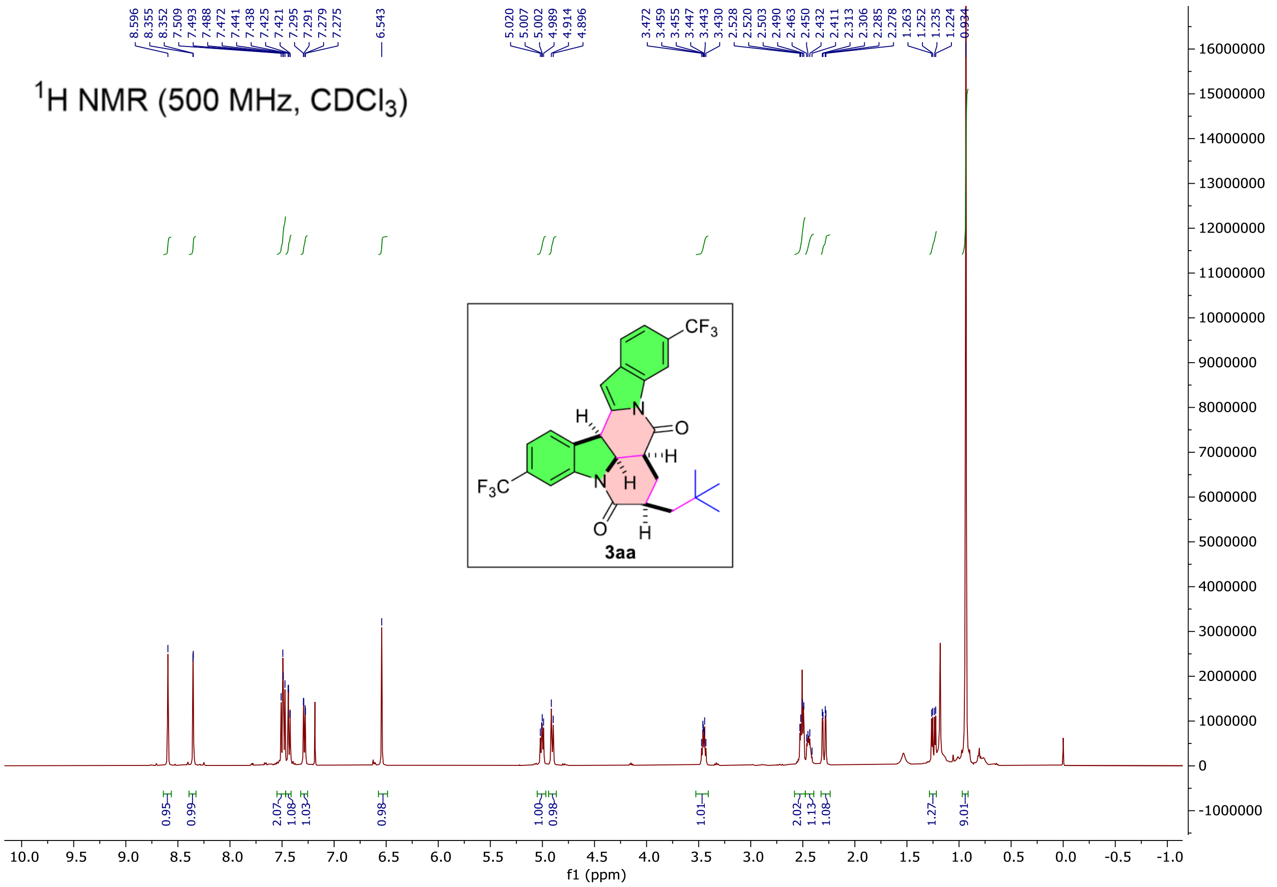
^{1}H NMR (500 MHz, CDCl$_3$)
3aa
f1 (ppm)

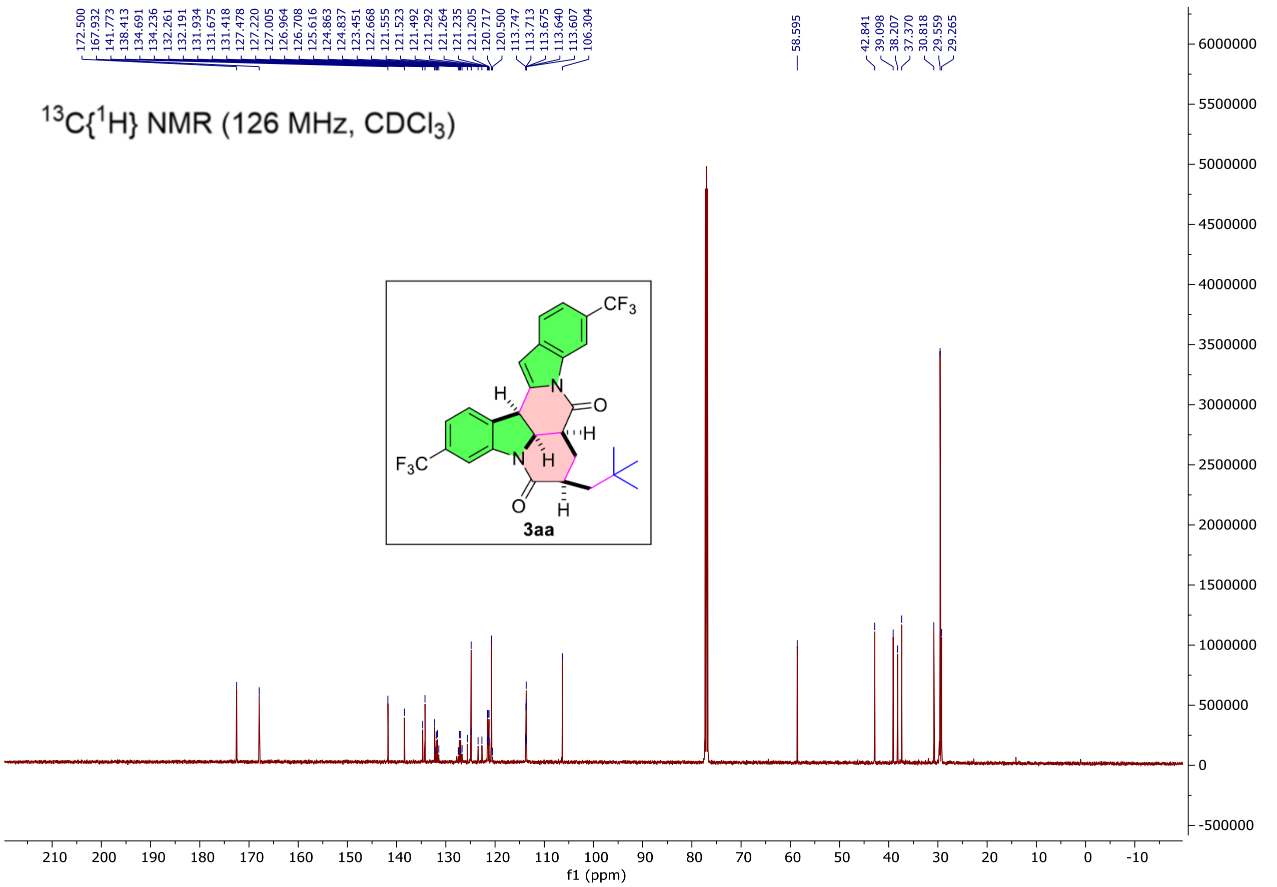
^{13}C{^{1}H} NMR (126 MHz, CDCl$_3$)
3aa
f1 (ppm)

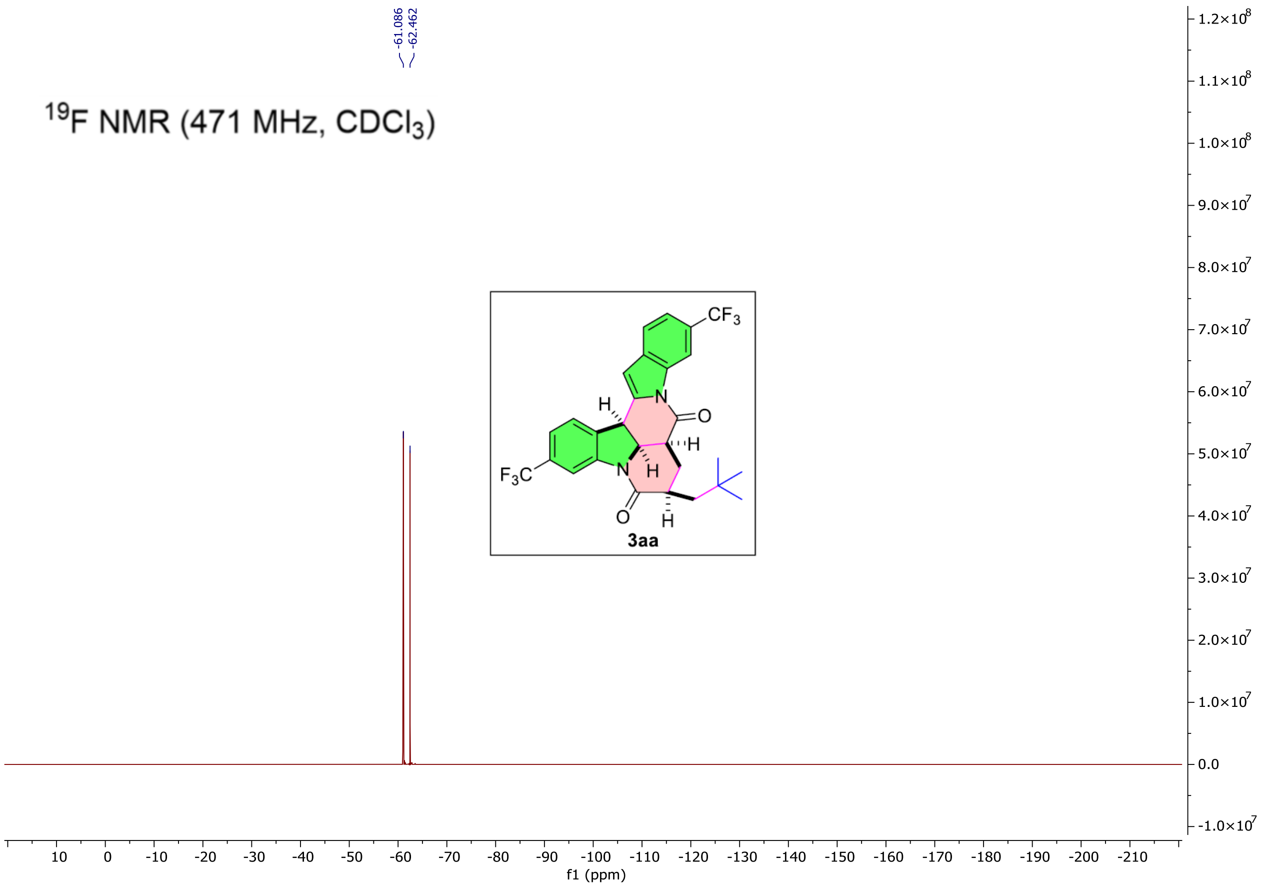

-61.086
-62.462
^{19}F NMR (471 MHz, CDCl$_3$)
CF$_3$
F$_3$C
N
H
O
3aa
f1 (ppm)

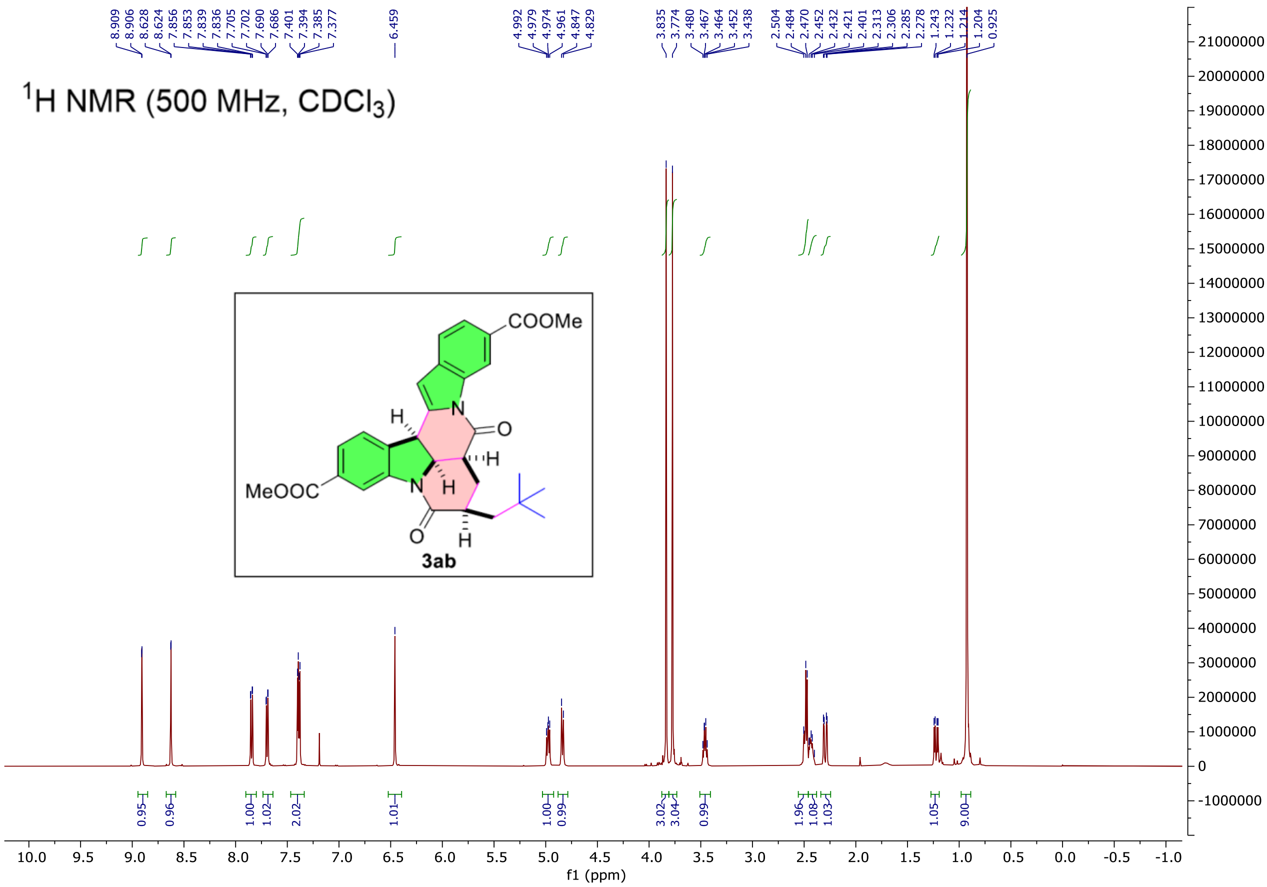

8.909
8.906
8.628
8.624
7.856
7.853
7.839
7.836
7.705
7.702
7.690
7.686
7.401
7.394
7.385
7.377
6.459
4.992
4.979
4.974
4.961
4.847
4.829
3.835
3.774
3.480
3.467
3.464
3.452
3.438
2.504
2.484
2.470
2.452
2.432
2.421
2.401
2.313
2.306
2.285
2.278
1.243
1.232
1.214
1.204
0.925
^{1}H NMR (500 MHz, CDCl$_3$)
COOMe
MeOOC
N
H
O
3ab
0.95
0.96
1.00
1.02
2.02
1.01
1.00
0.99
3.02
3.04
0.99
1.96
1.08
1.03
1.05
9.00
f1 (ppm)

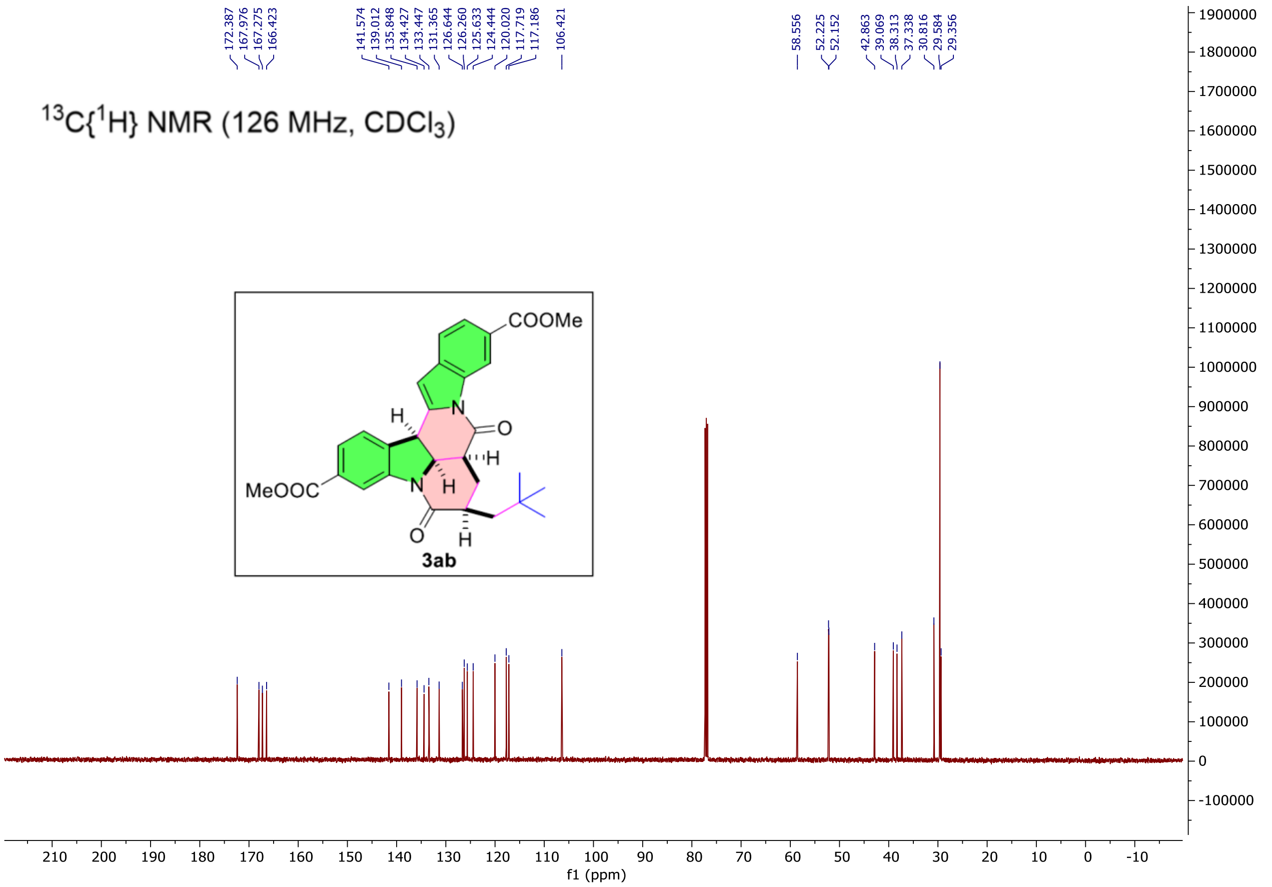
172.387
167.976
167.275
166.423
141.574
139.012
135.848
134.427
133.447
131.365
126.644
126.260
125.633
124.444
120.020
117.719
117.186
106.421
58.556
52.225
52.152
42.863
39.069
38.313
37.338
30.816
29.584
29.356
13C{1H} NMR (126 MHz, CDCl3)
COOMe
MeOOC
3ab
f1 (ppm)

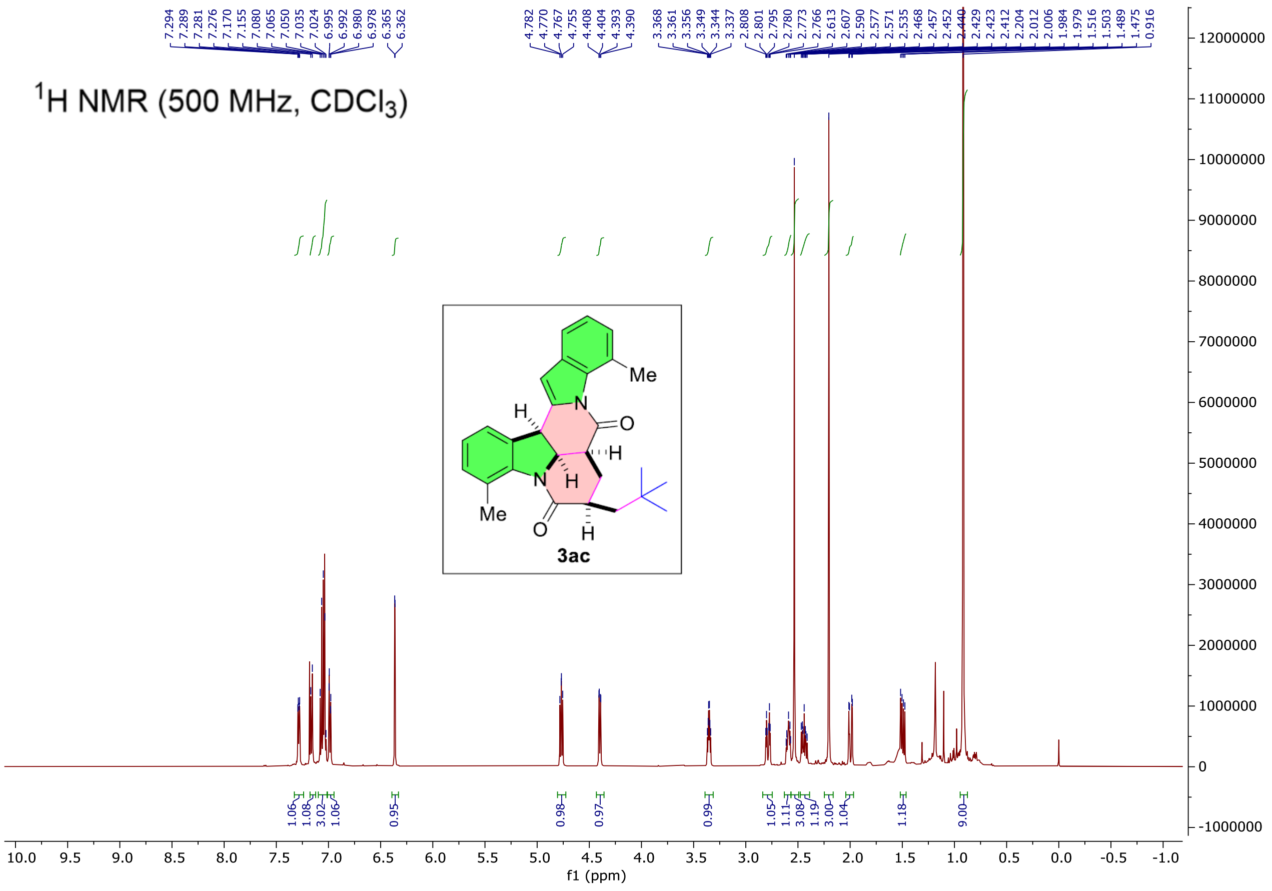
1H NMR (500 MHz, CDCl3)
Me
3ac
f1 (ppm)

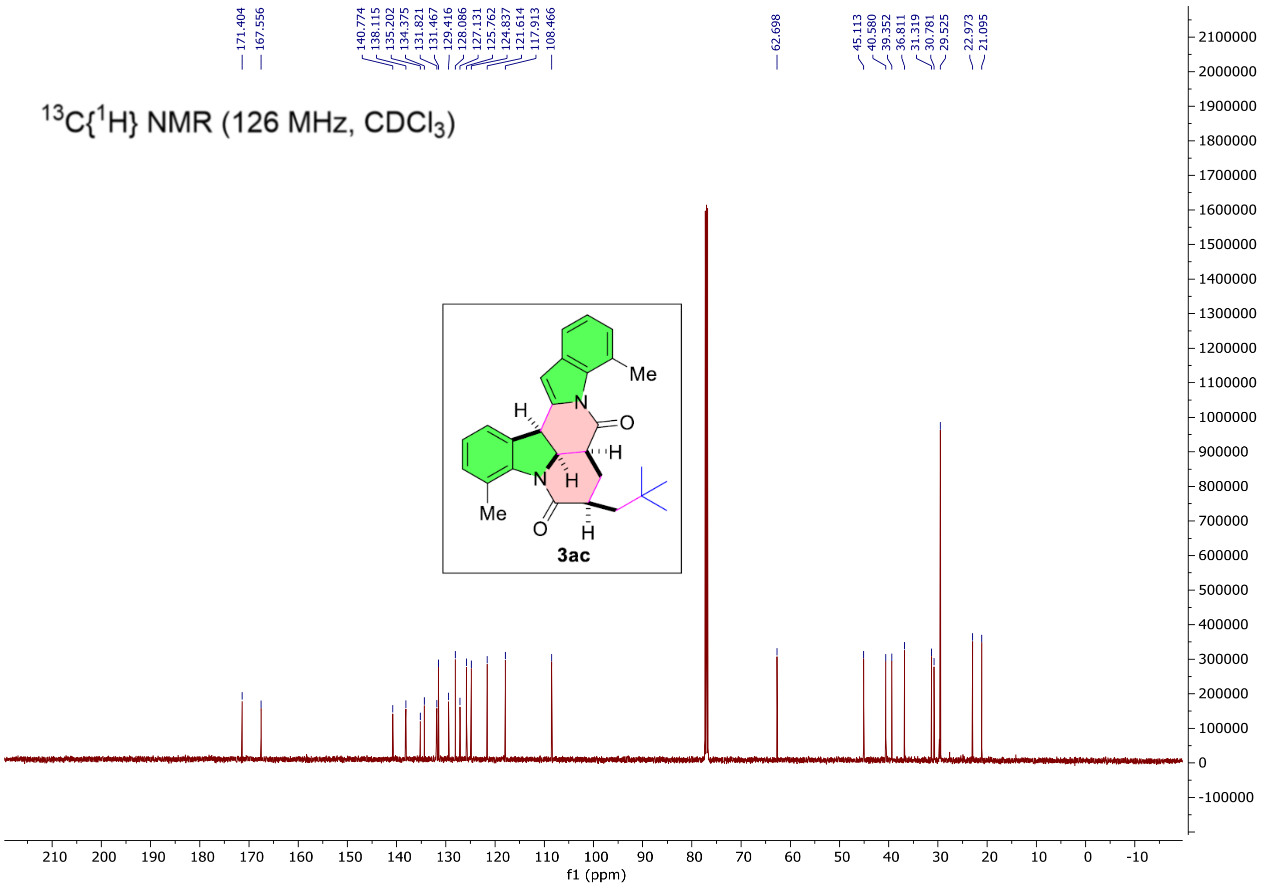
$^{13}C\{^1H\}$ NMR (126 MHz, $CDCl_3$)
Me
N
H
O
Me
O
H
H
H
3ac
f1 (ppm)

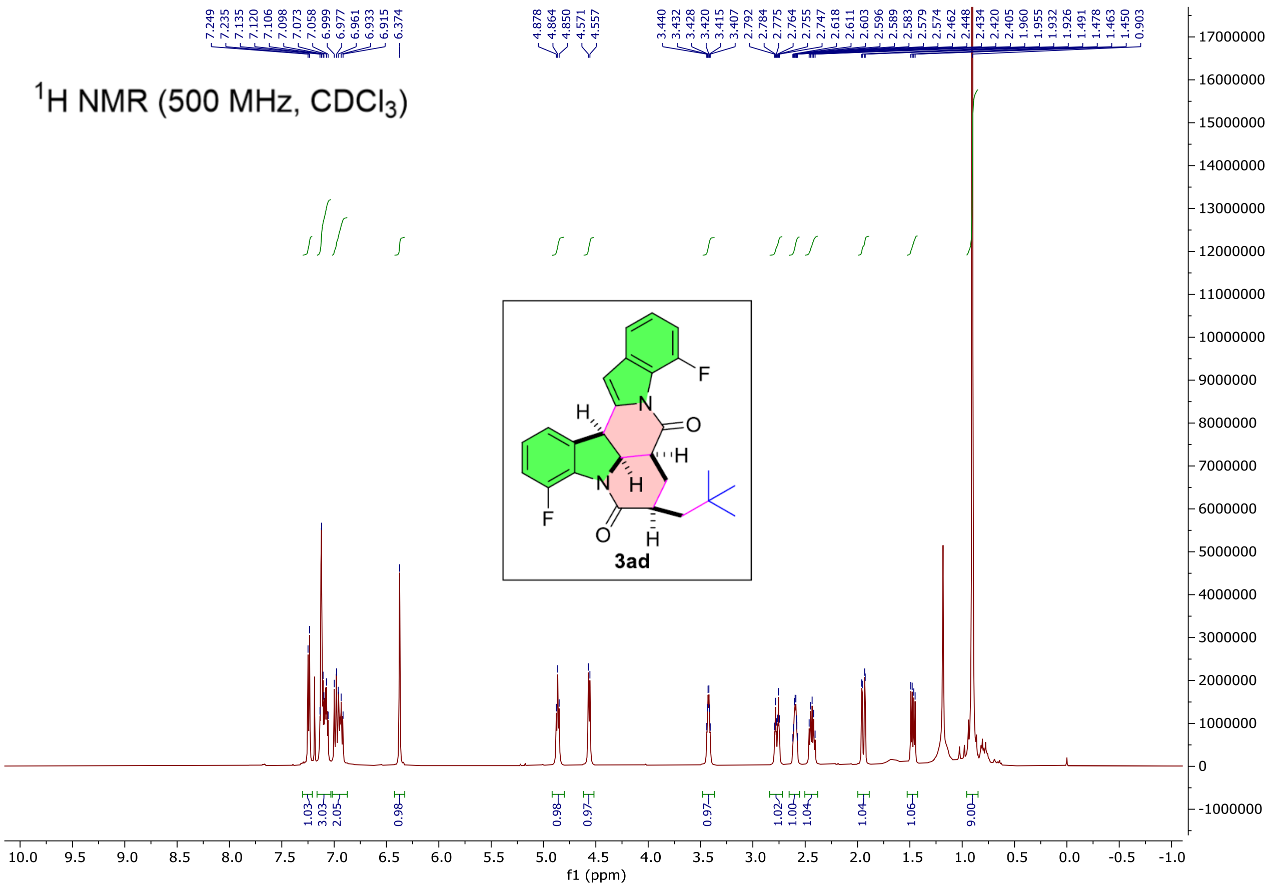
^1H NMR (500 MHz, $CDCl_3$)
F
N
H
O
F
O
H
H
H
3ad
f1 (ppm)

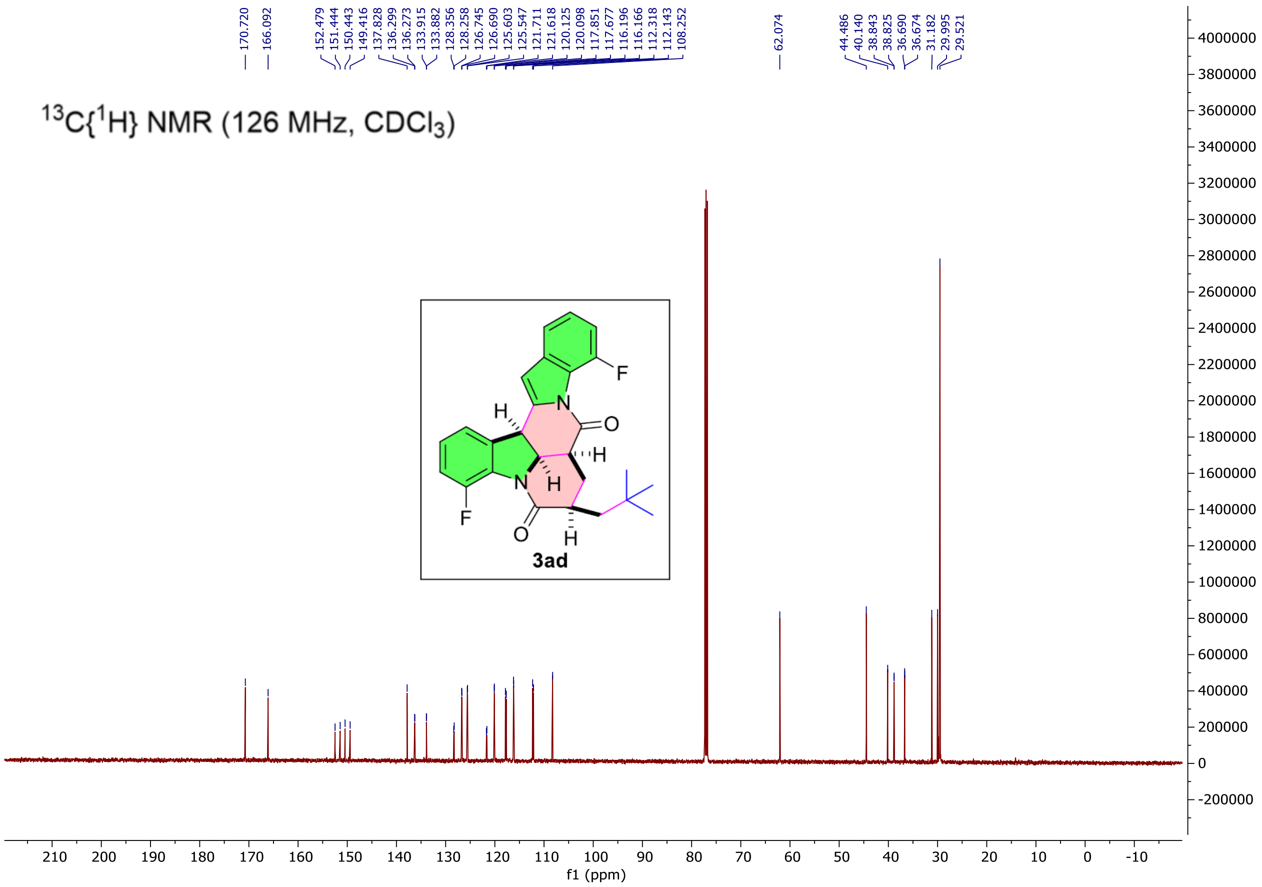
170.720
166.092
152.479
151.444
150.443
149.416
137.828
136.299
136.273
133.915
133.882
128.356
128.258
126.745
126.690
125.603
125.547
121.711
121.618
120.125
120.098
117.851
117.677
116.196
116.166
112.318
112.143
108.252
62.074
44.486
40.140
38.843
38.825
36.690
36.674
31.182
29.995
29.521
$^{13}C\{^1H\}$ NMR (126 MHz, $CDCl_3$)
F
H
N
O
H
N
H
F
O
H
3ad
f1 (ppm)

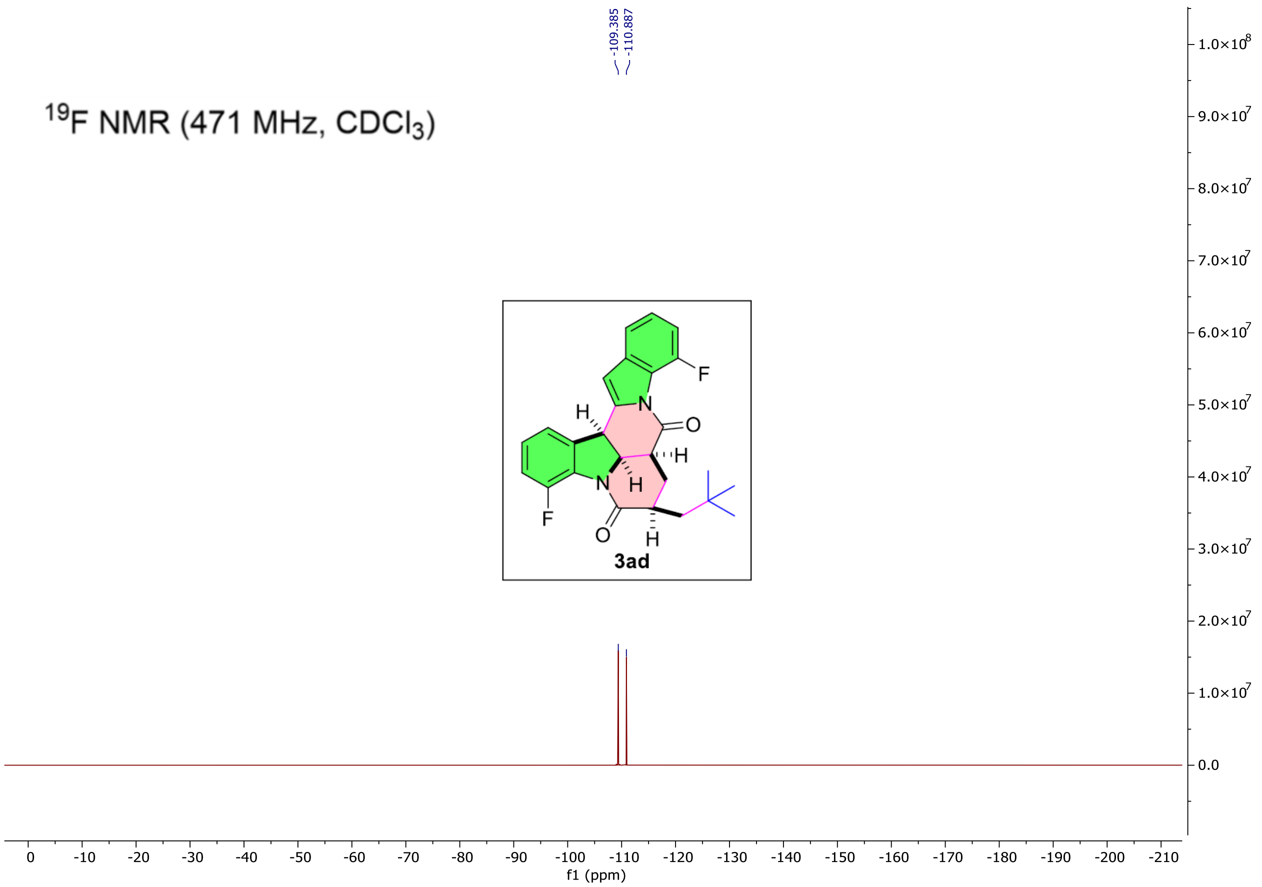
-109.385
-110.887
^{19}F NMR (471 MHz, $CDCl_3$)
F
H
N
O
H
N
H
F
O
H
3ad
f1 (ppm)

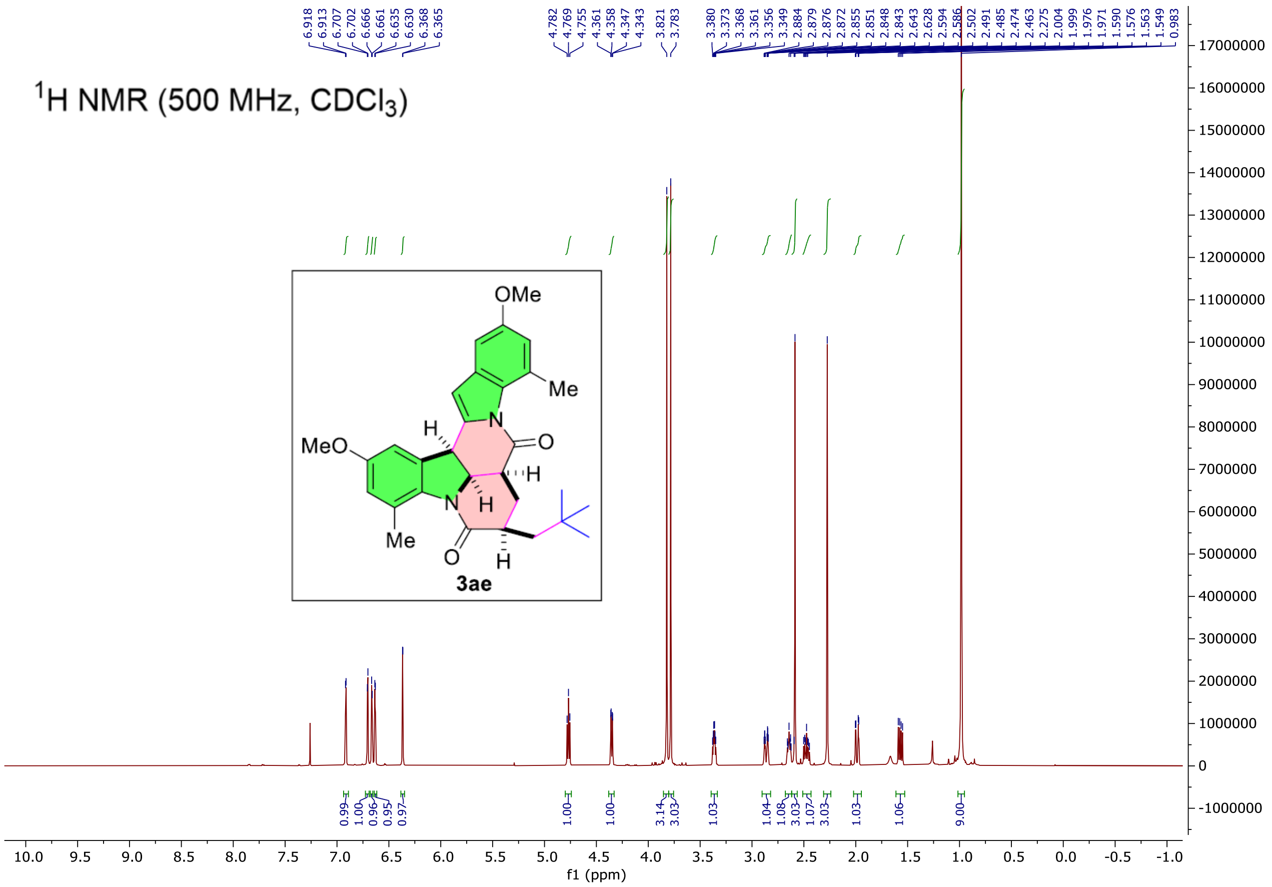

1H NMR (500 MHz, CDCl3)
OMe
Me
N
O
H
MeO
H
N
H
Me
O
H
3ae
f1 (ppm)

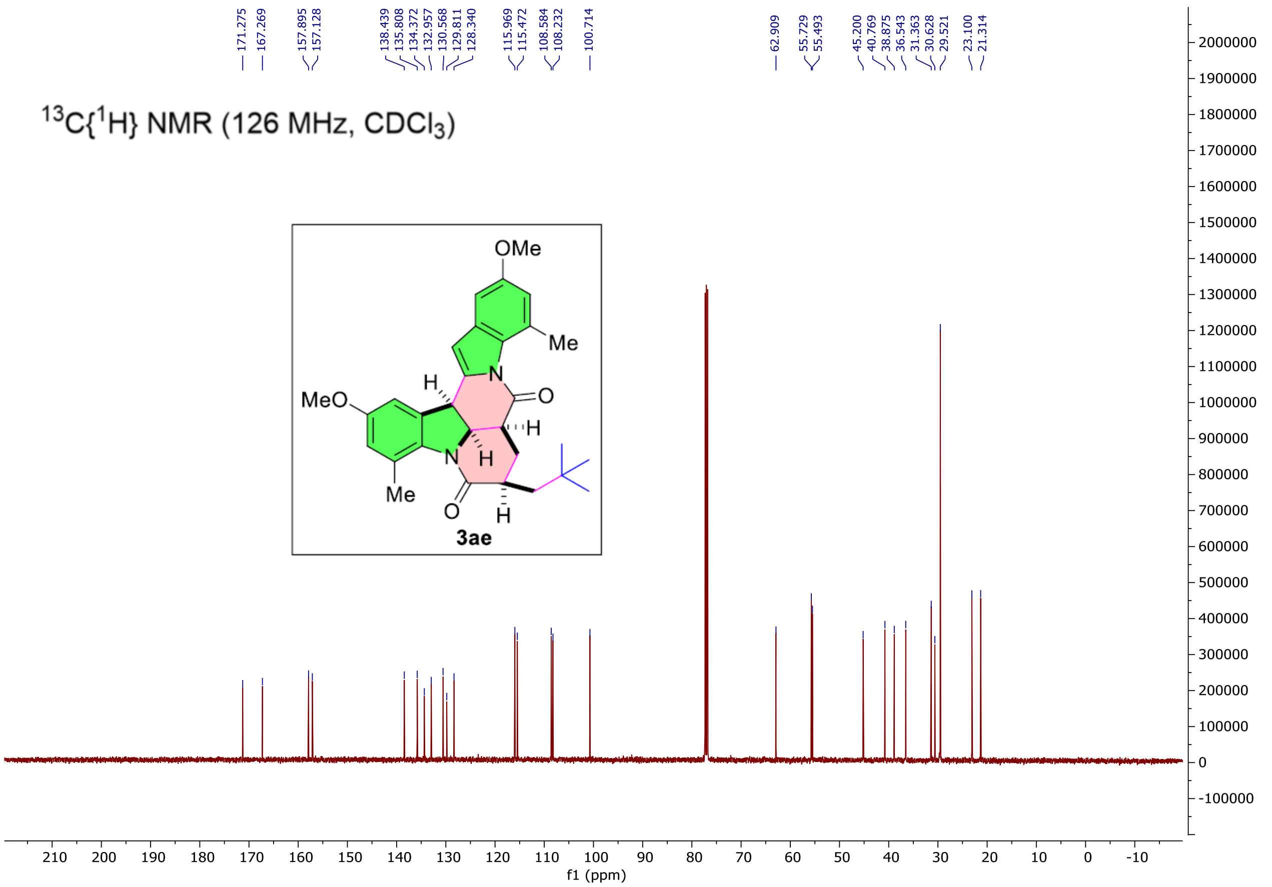

13C{1H} NMR (126 MHz, CDCl3)
OMe
Me
N
O
H
MeO
H
N
H
Me
O
H
3ae
f1 (ppm)

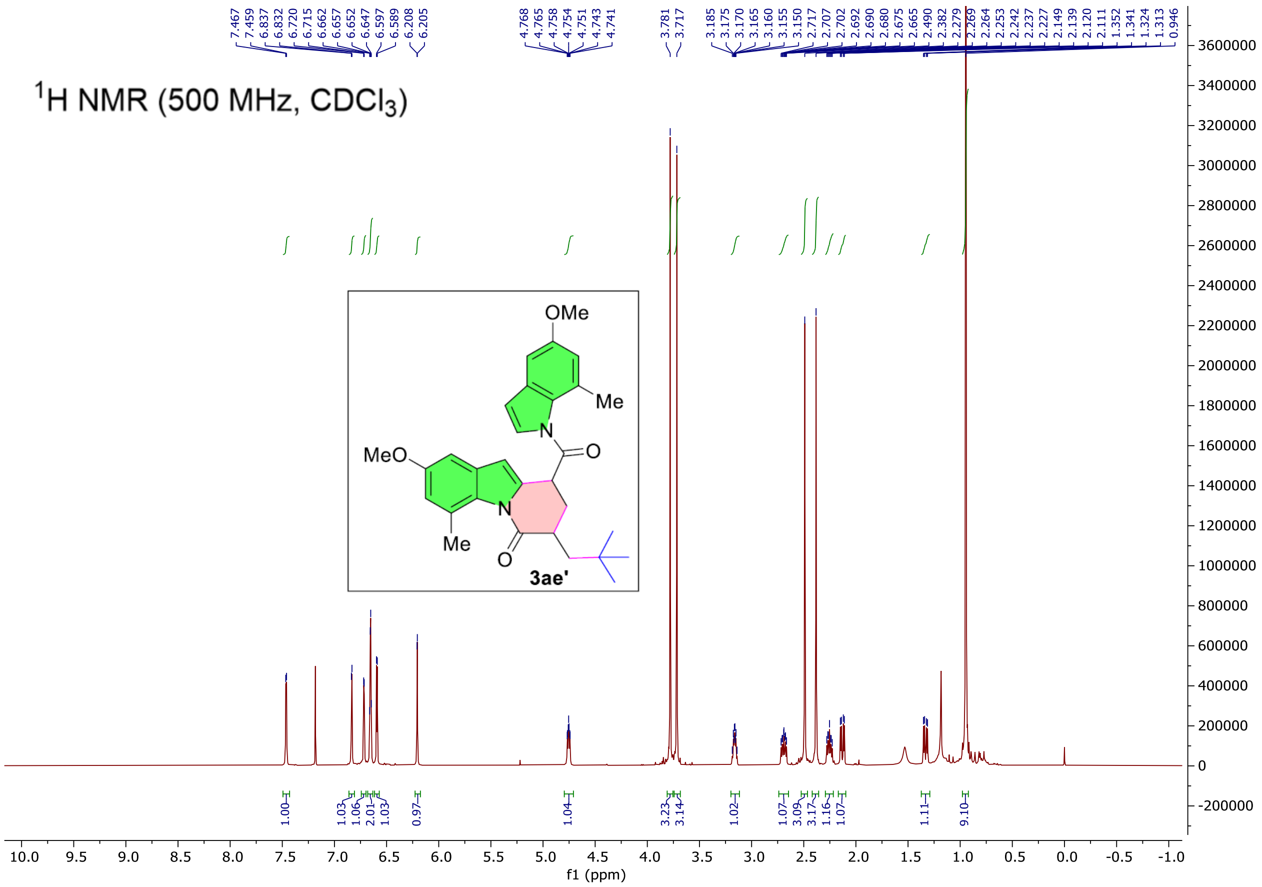

7.467 7.459 6.837 6.832 6.720 6.715 6.662 6.657 6.652 6.647 6.597 6.589 6.208 6.205
4.768 4.765 4.758 4.754 4.751 4.743 4.741
3.781 3.717
3.185 3.175 3.170 3.165 3.160 3.155 3.150 2.717 2.707 2.702 2.692 2.690 2.680 2.675 2.665 2.490 2.382 2.279 2.269 2.264 2.253 2.242 2.237 2.227 2.149 2.139 2.120 2.111 1.352 1.341 1.324 1.313 0.946
^{1}H NMR (500 MHz, CDCl$_3$)
OMe
Me
N
O
MeO
N
Me
O
3ae'
1.00 1.03 1.06 2.01 1.03 0.97 1.04 3.23 3.14 1.02 1.07 3.09 3.17 1.16 1.07 1.11 9.10
f1 (ppm)

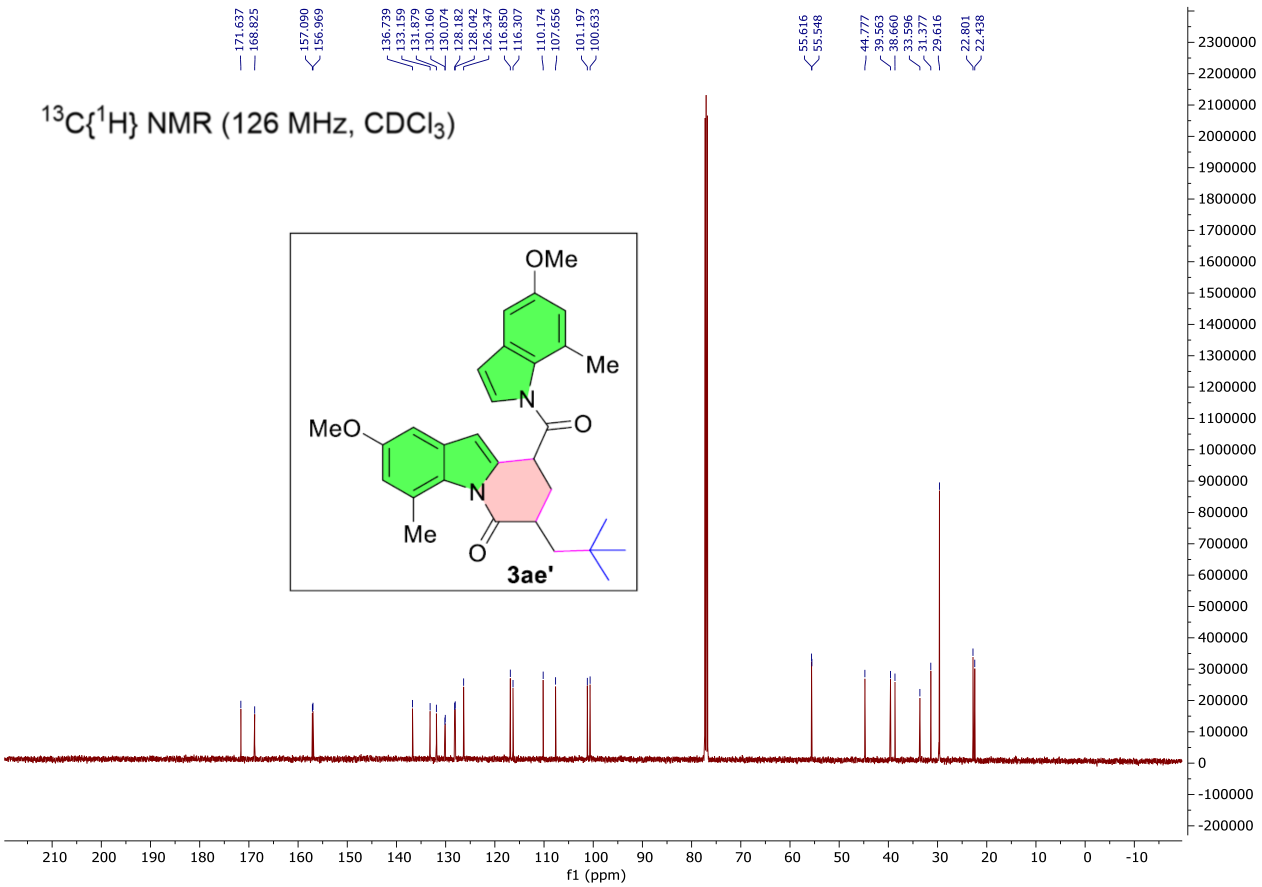

171.637 168.825 157.090 156.969 136.739 133.159 131.879 130.160 130.074 128.182 128.042 126.347 116.850 116.307 110.174 107.656 101.197 100.633 55.616 55.548 44.777 39.563 38.660 33.596 31.377 29.616 22.801 22.438
^{13}C{^{1}H} NMR (126 MHz, CDCl$_3$)
OMe
Me
N
O
MeO
N
Me
O
3ae'
f1 (ppm)

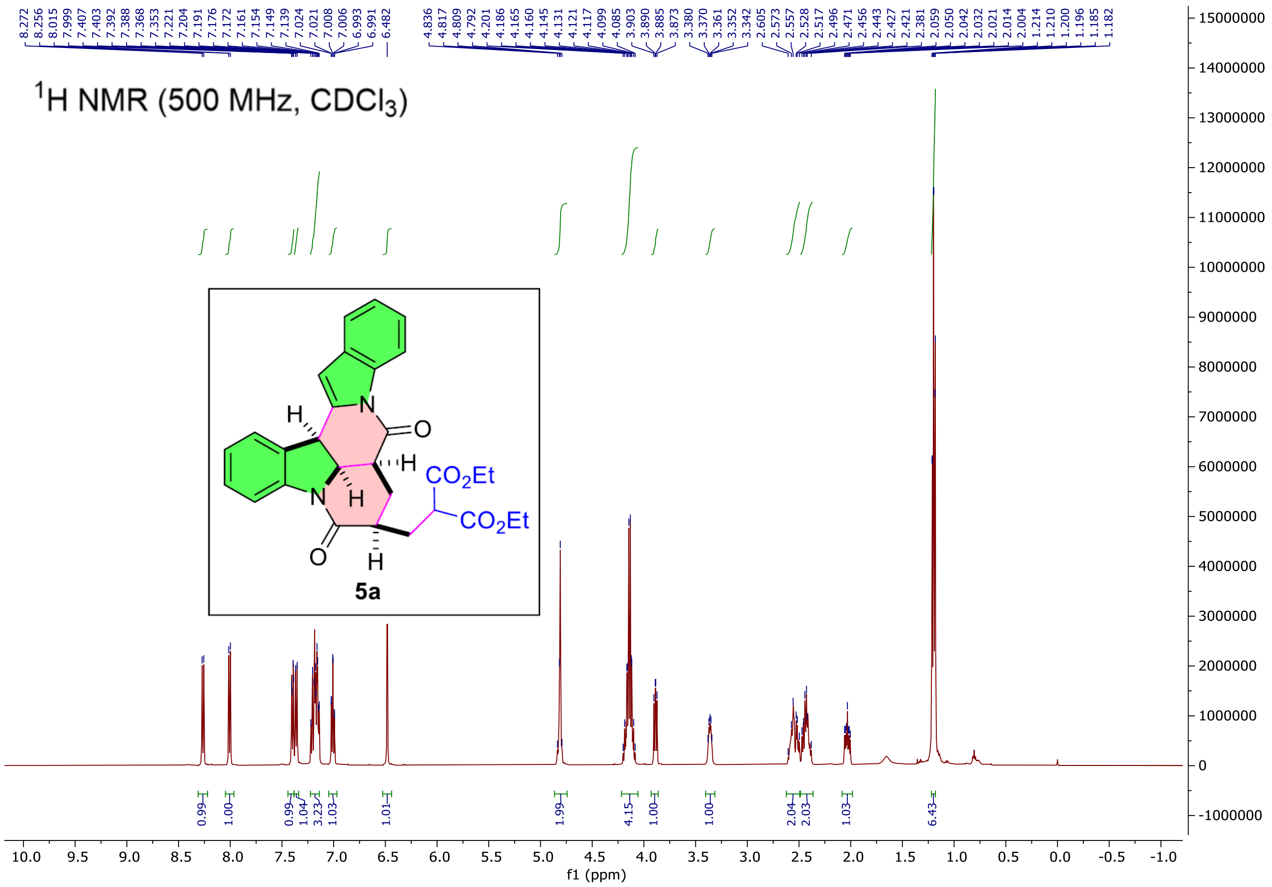
8.272 8.256 8.015 7.999 7.407 7.403 7.392 7.388 7.368 7.353 7.221 7.204 7.191 7.176 7.172 7.161 7.154 7.149 7.139 7.024 7.021 7.008 7.006 6.993 6.991 6.482
4.836 4.817 4.809 4.792 4.201 4.186 4.165 4.160 4.145 4.131 4.121 4.117 4.099 4.085 3.903 3.890 3.885 3.873 3.380 3.370 3.361 3.352 3.342 2.605 2.573 2.557 2.528 2.517 2.496 2.471 2.456 2.443 2.427 2.421 2.381 2.059 2.050 2.042 2.032 2.021 2.014 2.004 1.214 1.210 1.200 1.196 1.185 1.182
^{1}H NMR (500 MHz, CDCl$_3$)
H
N
O
H
CO$_2$Et
N
H
CO$_2$Et
O
H
5a
0.99 1.00 0.99 1.04 3.23 1.03 1.01 1.99 4.15 1.00 1.00 2.04 2.03 1.03 6.43
f1 (ppm)

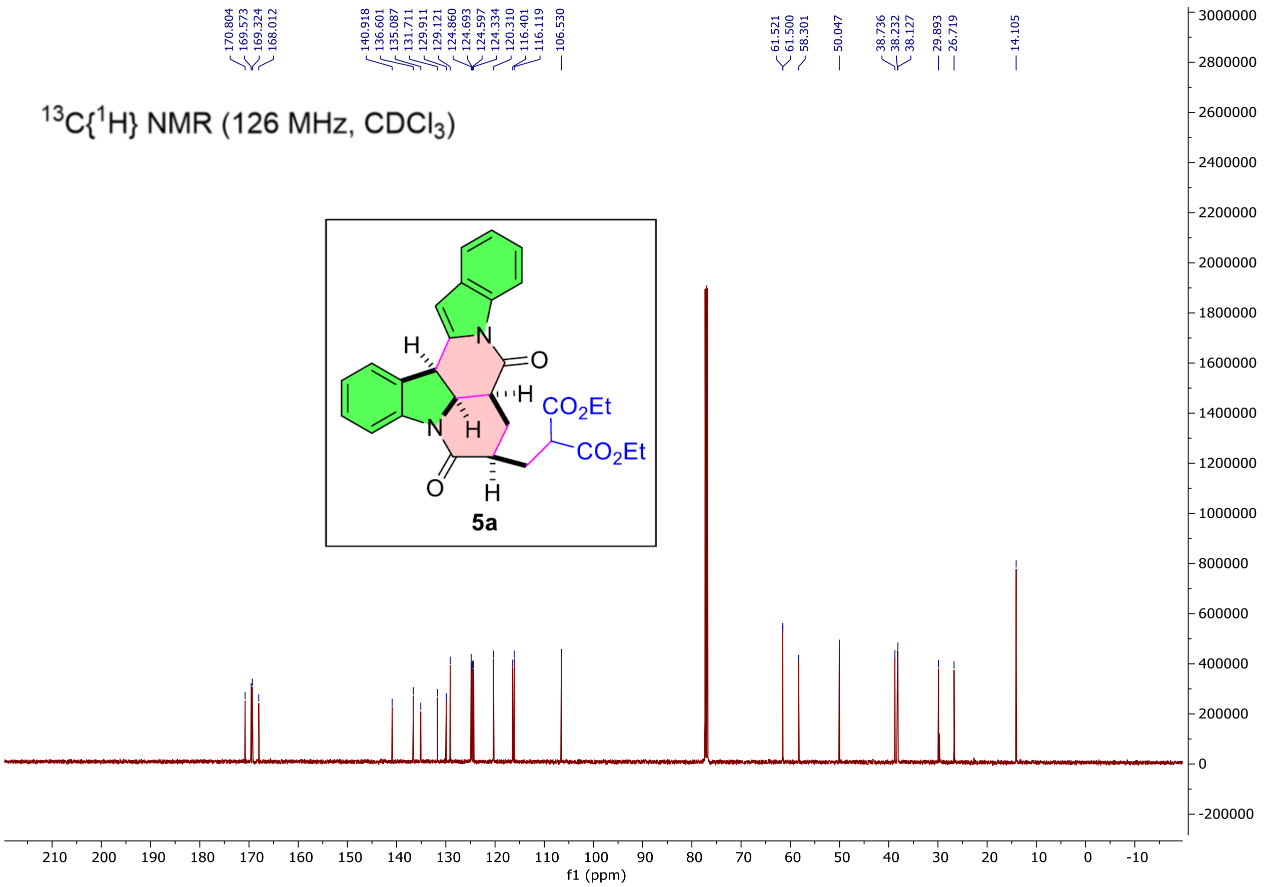
170.804 169.573 169.324 168.012
140.918 136.601 135.087 131.711 129.911 129.121 124.860 124.693 124.597 124.334 120.310 116.401 116.119 106.530
61.521 61.500 58.301 50.047 38.736 38.232 38.127 29.893 26.719 14.105
^{13}C{^{1}H} NMR (126 MHz, CDCl$_3$)
H
N
O
H
CO$_2$Et
N
H
CO$_2$Et
O
H
5a
f1 (ppm)

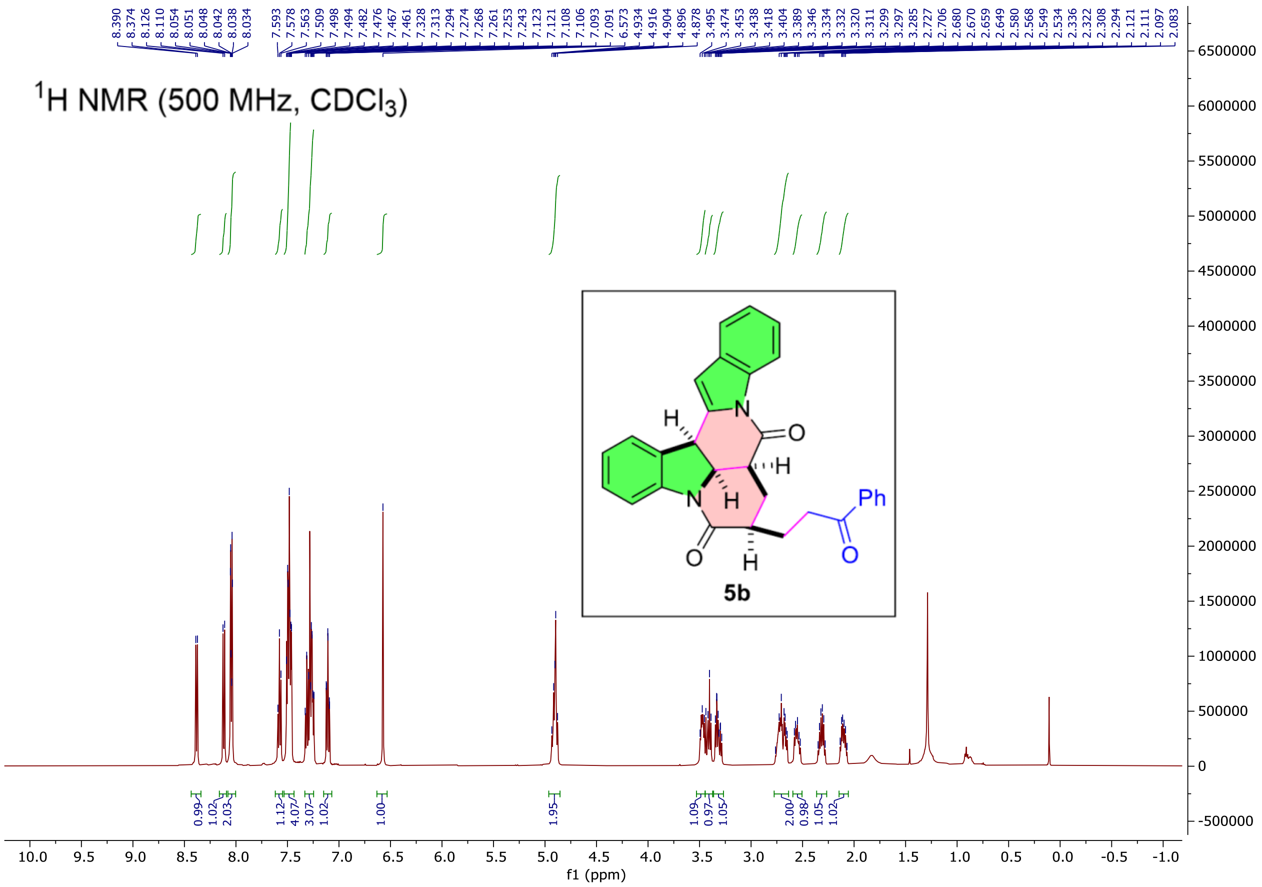

^{1}H NMR (500 MHz, CDCl$_3$)
5b
Ph
f1 (ppm)

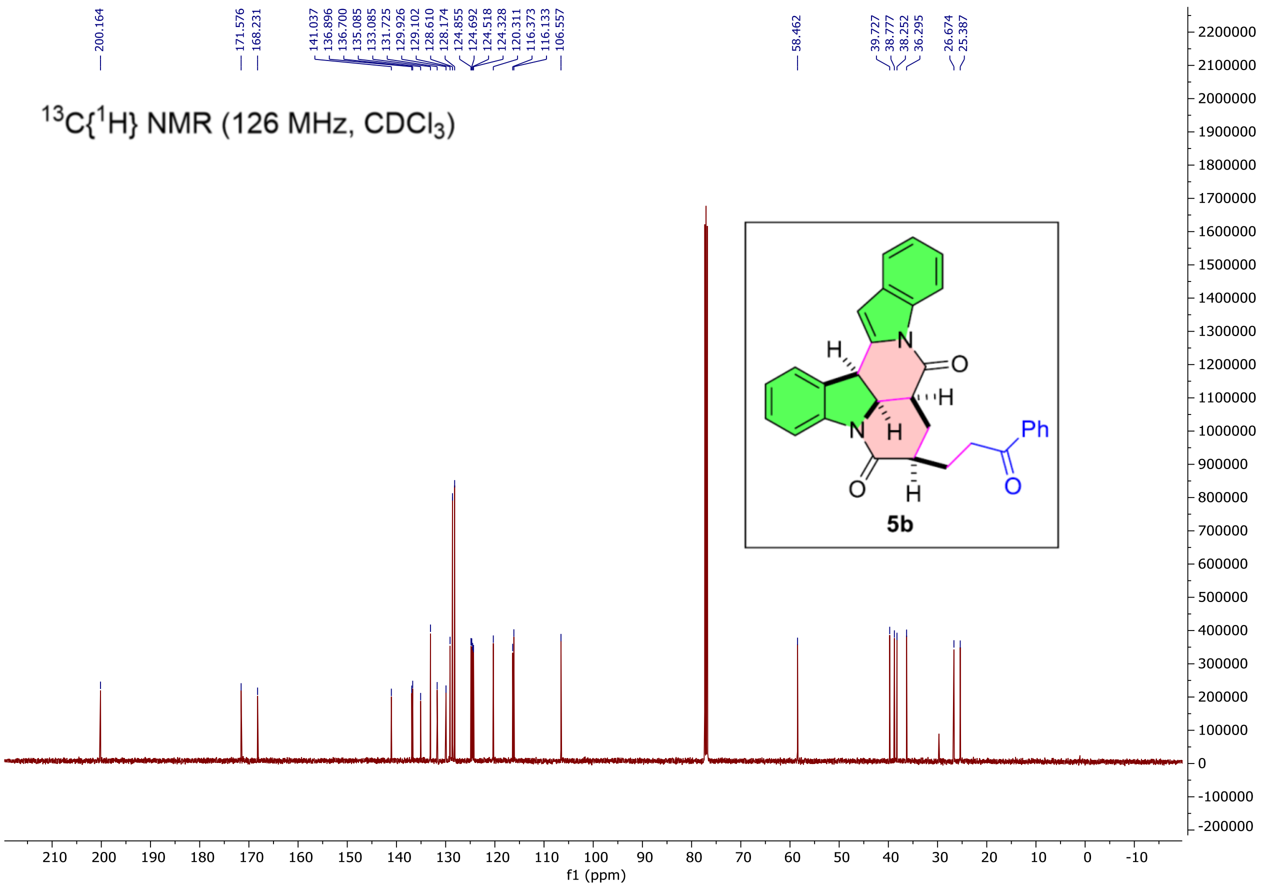

^{13}C{^{1}H} NMR (126 MHz, CDCl$_3$)
5b
Ph
f1 (ppm)

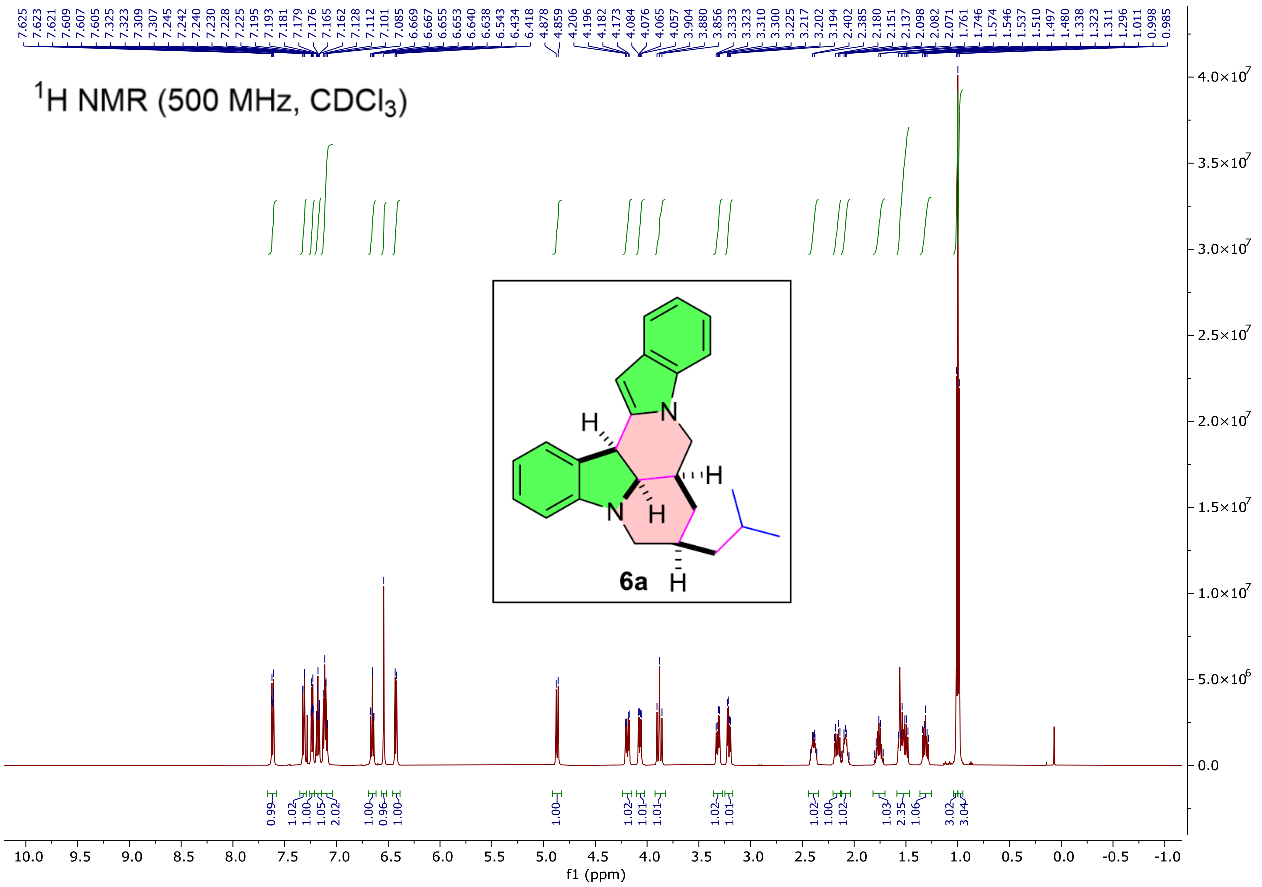

1H NMR (500 MHz, CDCl3)
6a
f1 (ppm)

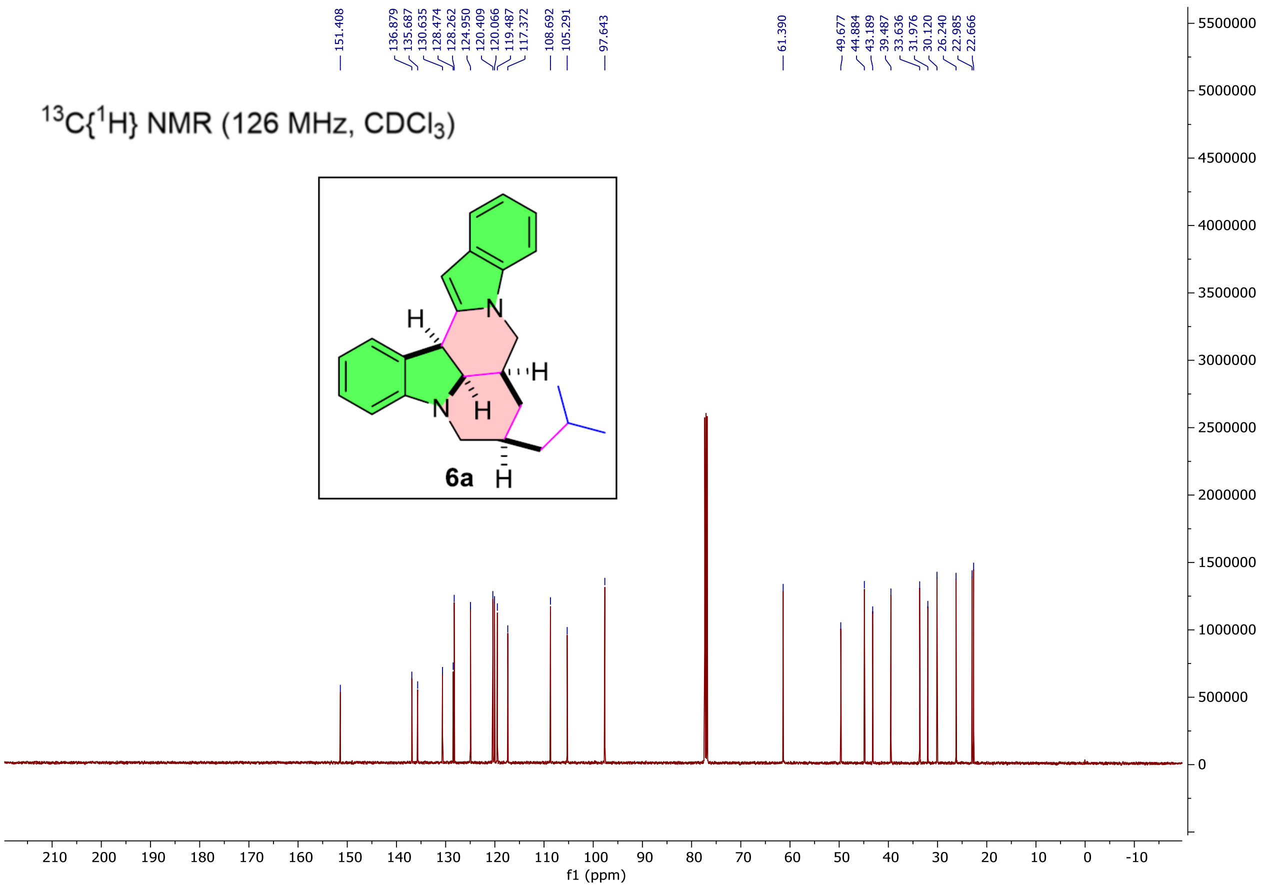

13C{1H} NMR (126 MHz, CDCl3)
6a
f1 (ppm)

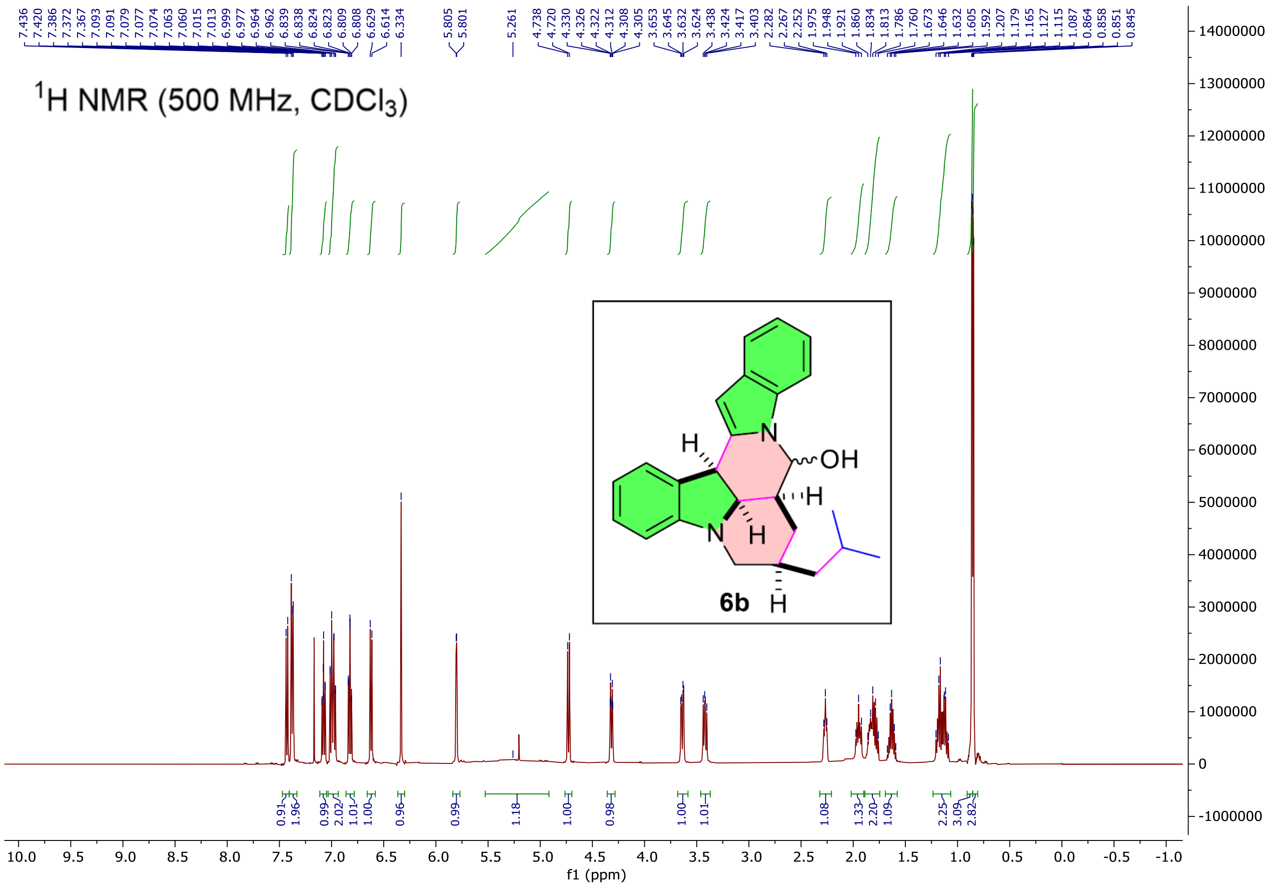

^{1}H NMR (500 MHz, CDCl$_3$)
6b
f1 (ppm)

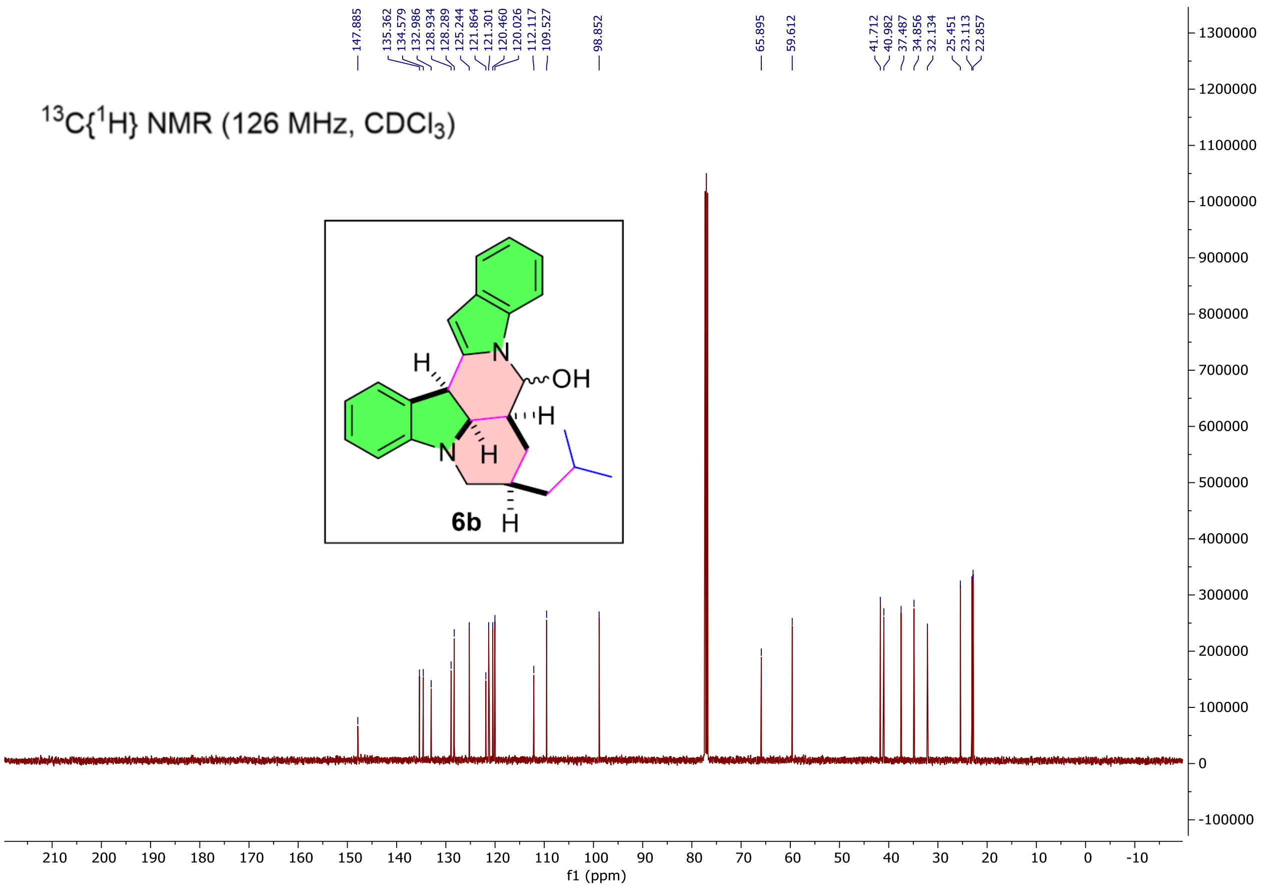

^{13}C{^{1}H} NMR (126 MHz, CDCl$_3$)
147.885
135.362
134.579
132.986
128.934
128.289
125.244
121.864
121.301
120.460
120.026
112.117
109.527
98.852
65.895
59.612
41.712
40.982
37.487
34.856
32.134
25.451
23.113
22.857
6b
f1 (ppm)

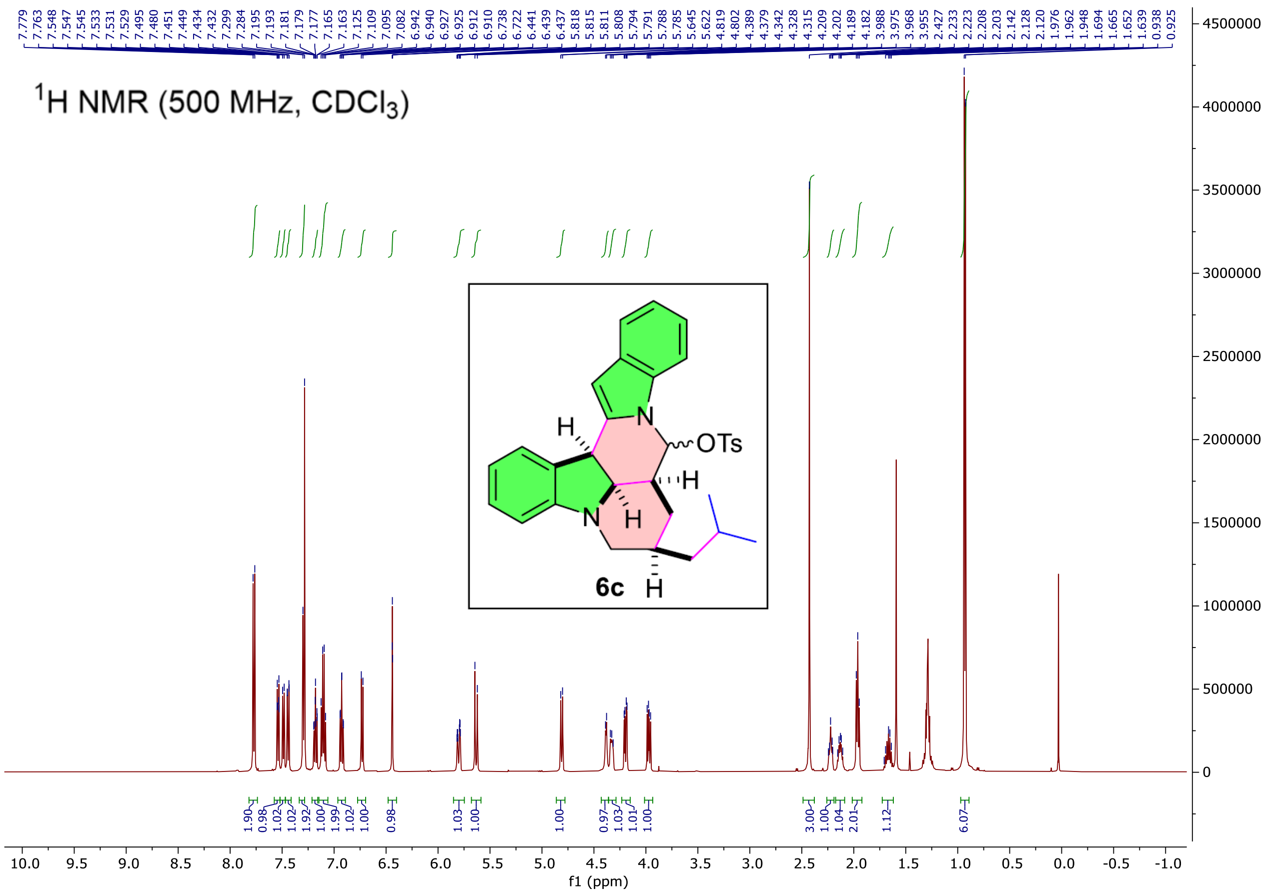
1H NMR (500 MHz, CDCl3)
7.779 7.763 7.548 7.547 7.545 7.533 7.531 7.529 7.495 7.480 7.451 7.449 7.434 7.432 7.299 7.284 7.195 7.193 7.181 7.179 7.177 7.165 7.163 7.125 7.109 7.095 7.082 6.942 6.940 6.927 6.925 6.912 6.910 6.738 6.722 6.441 6.439 6.437 5.818 5.815 5.811 5.808 5.794 5.791 5.788 5.785 5.645 5.622 4.819 4.802 4.389 4.379 4.342 4.328 4.315 4.209 4.202 4.189 4.182 3.988 3.975 3.968 3.955 2.427 2.233 2.223 2.208 2.203 2.142 2.128 2.120 1.976 1.962 1.948 1.694 1.665 1.652 1.639 0.938 0.925
1.90 0.98 1.02 1.02 1.92 1.00 1.99 1.02 1.00 0.98 1.03 1.00 1.00 0.97 1.03 1.01 1.00 3.00 1.00 1.04 2.01 1.12 6.07
H
N
OTs
H
N
H
6c
H
f1 (ppm)

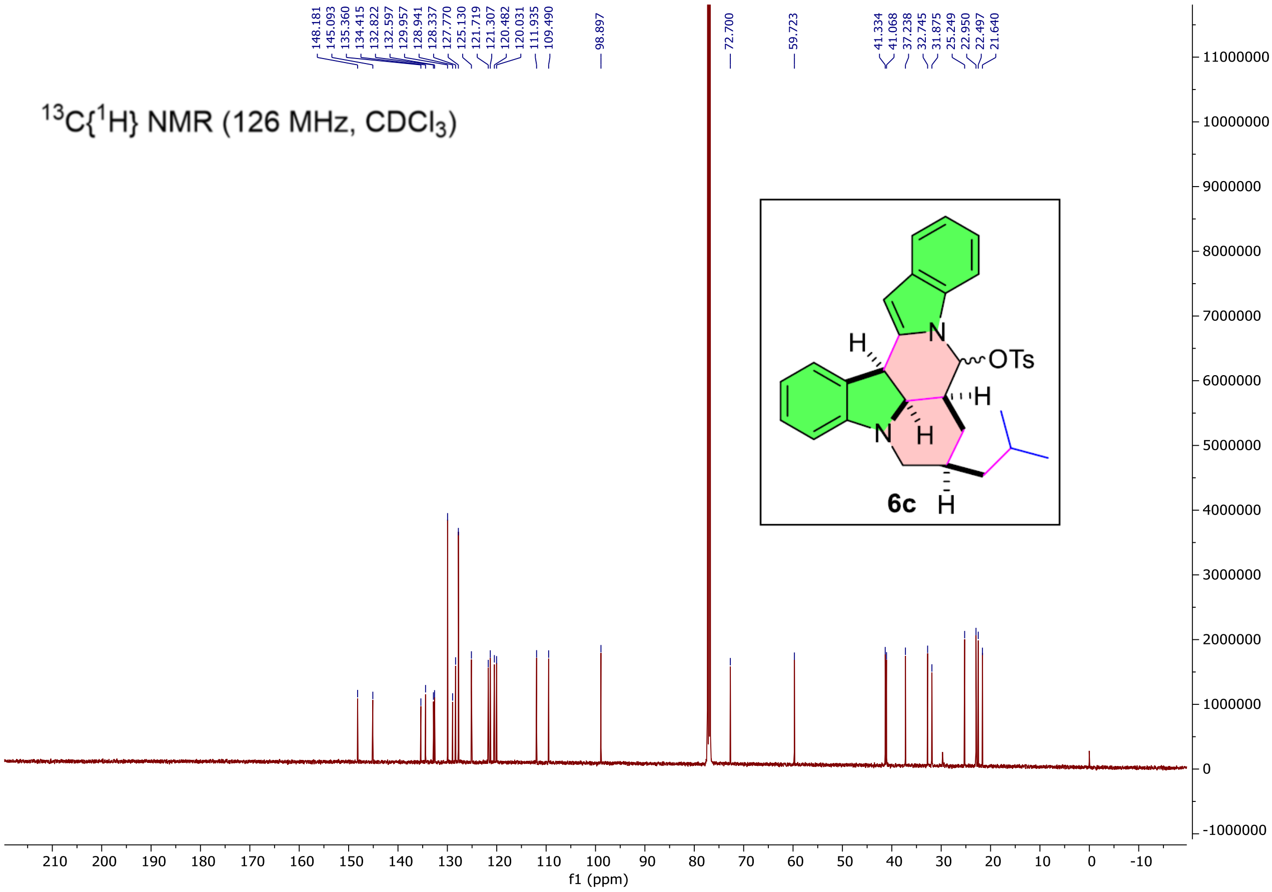
13C{1H} NMR (126 MHz, CDCl3)
148.181 145.093 135.360 134.415 132.822 132.597 129.957 128.941 128.337 127.770 125.130 121.719 121.307 120.482 120.031 111.935 109.490 98.897 72.700 59.723 41.334 41.068 37.238 32.745 31.875 25.249 22.950 22.497 21.640
H
N
OTs
H
N
H
6c
H
f1 (ppm)

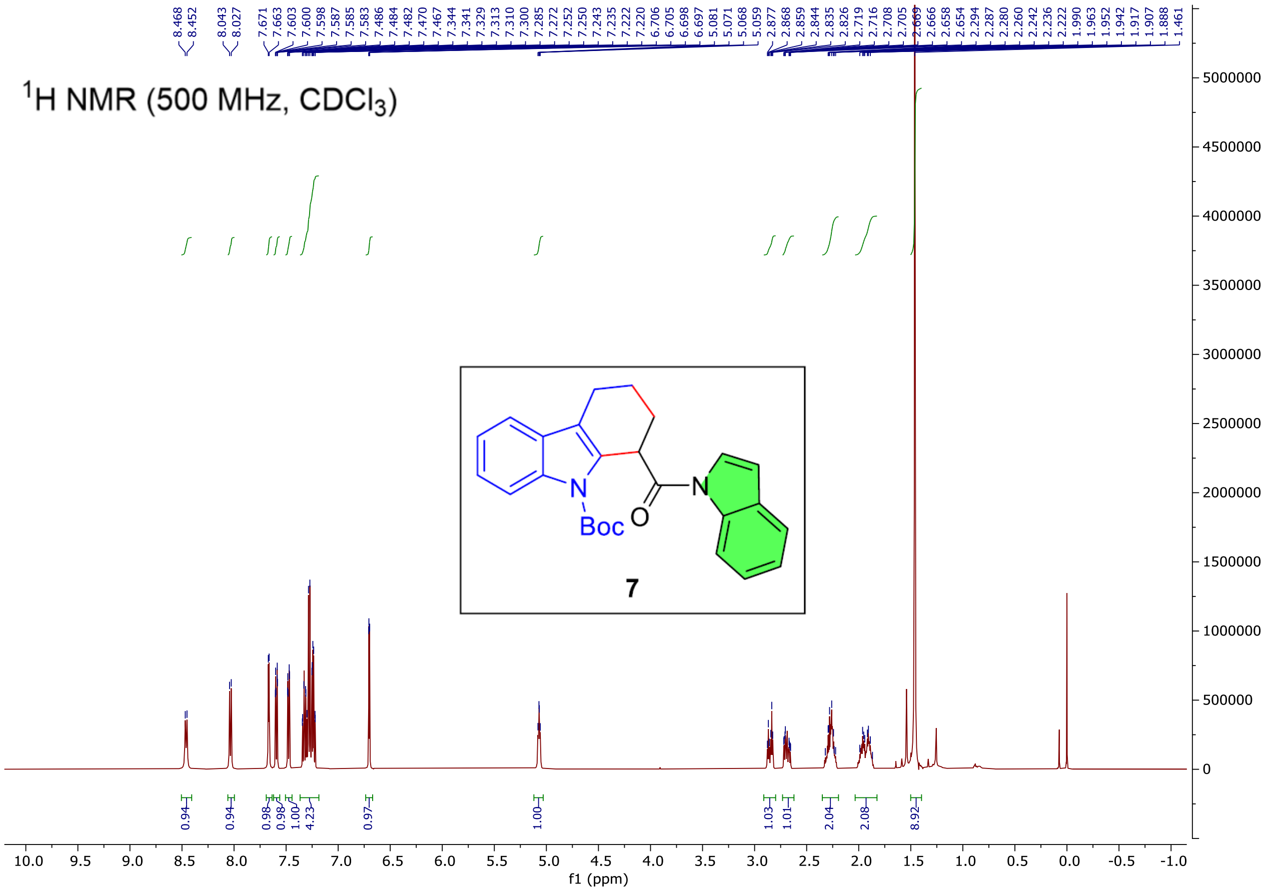

1H NMR (500 MHz, CDCl3)
N
Boc
O
N
7
f1 (ppm)

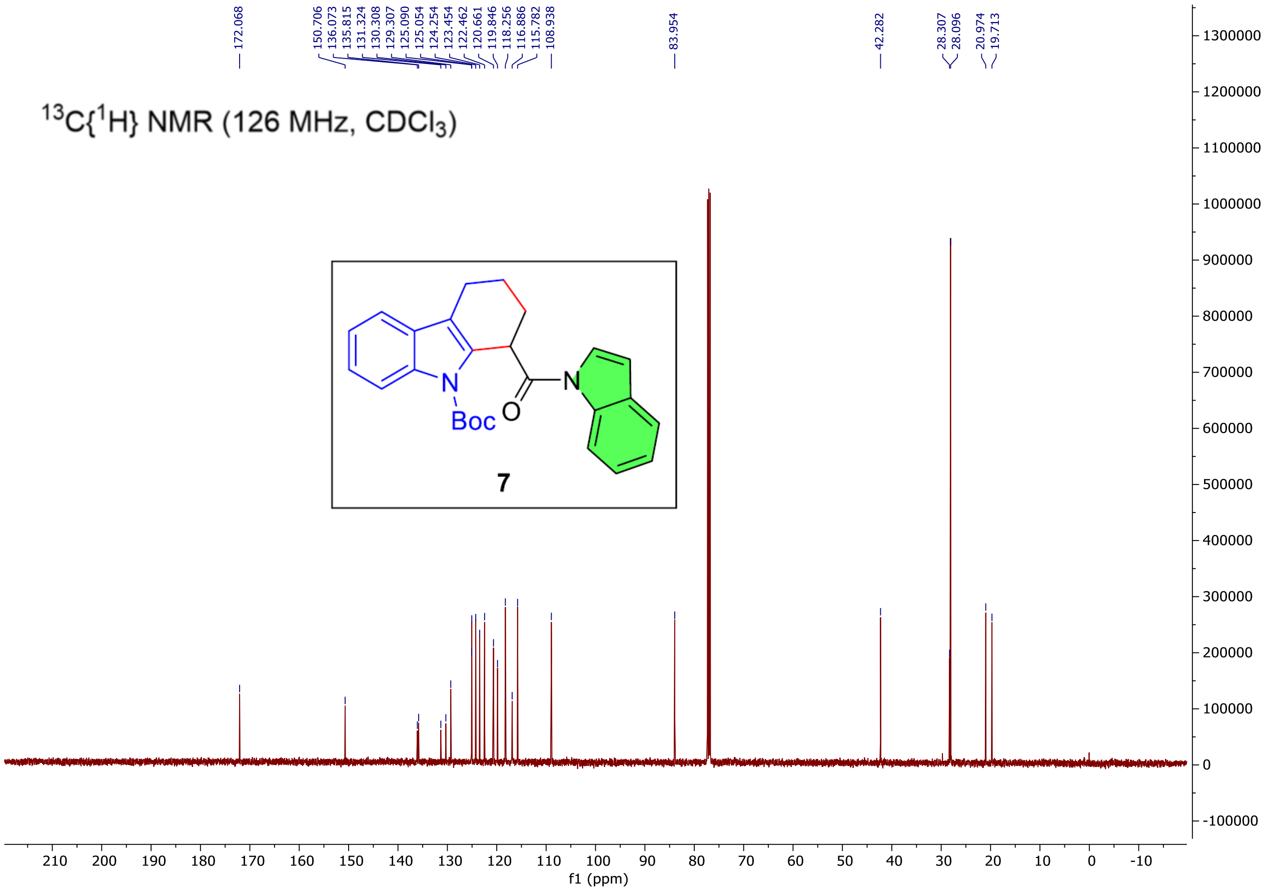

13C{1H} NMR (126 MHz, CDCl3)
172.068
150.706
136.073
135.815
131.324
130.308
129.307
125.090
125.054
124.254
123.454
122.462
120.661
119.846
118.256
116.886
115.782
108.938
83.954
42.282
28.307
28.096
20.974
19.713
N
Boc
O
N
7
f1 (ppm)

17. References

(1) Magolan, J.; Carson, C. A.; Kerr, M. A. Total Synthesis of (±)-Mersicarpine. *Org. Lett.* **2008**, *10*, 1437–1440.

(2) Xia, H. D.; Zhang, Y. D.; Wang, Y. H.; Zhang, C. Water-Soluble Hypervalent Iodine(III) Having an I−N Bond. A Reagent for the Synthesis of Indoles. *Org. Lett.* **2018**, *20*, 4052−4056.

(3) Irwin, L. C.; Kerr, M. A. One-Pot Michael Addition/Radical Cyclization Reaction of N-Acryloyl Indoles. *Synlett* **2017**, *28*, 2859–2864.

(4) Bishir, C.; Hubbard, A.; Mei, L. Visible-Light-Mediated Rose Bengal- or $[Ru(bpy)_3]^{2+}$-Catalyzed Radical [4 + 2] Cycloaddition: An Efficient Route to Tetrahydrocarbazoles. *ACS Omega* **2025**, *10*, 10713–10723.

(5) Yin, H.; Jian, S.; Feng, X.; Bao, M.; Zhang, X. Metal-free photoinduced denitrogenative alkylation of vinyl azides with alkyl radicals toward ketones. *Org. Chem. Front.* **2024**, *11*, 3124–3130.

(6) Song, H.; Cheng, R.; Min, Q. Q.; Zhang, X. Decarboxylative and Deaminative Alkylation of Difluoroenoxysilanes via Photoredox Catalysis: A General Method for Site-Selective Synthesis of Difluoroalkylated Alkanes. *Org. Lett.* **2020**, *22*, 7747–7751.

(7) Huihui, K. M. M.; Caputo, J. A.; Melchor, Z.; Olivares, A. M.; Spiewak, A. M.; Johnson, K. A.; Dibenedetto, T. A.; Kim, S.; Ackerman, L. K. G.; Weix, D. J. Decarboxylative Cross-Electrophile Coupling of N-Hydroxyphthalimide Esters with Aryl Iodides. *J. Am. Chem. Soc.* **2016**, *138*, 5016–5019.

(8) Brauer, J.; Quraishi, E.; Kammer, L.; Opatz, T. Nickel-Mediated Photoreductive Cross Coupling of Carboxylic Acid Derivatives for Ketone Synthesis. *Chem. Eur. J.* **2021**, *27*, 18168–18174.

(9) Xi, X.; Luo, Y.; Li, W.; Xu, M.; Zhao, H.; Chen, Y.; Zheng, S.; Qi, X.; Yuan, W. From Esters to Ketones via a Photoredox-Assisted Reductive Acyl Cross-Coupling Strategy. *Angew. Chem. Int. Ed.* **2022**, *61*, e202114731.

(10) Zhu, K.; Ma, Y.; Wu, Z.; Wu, J.; Lu, Y. Energy-Transfer-Enabled Regioconvergent Alkylation of Azlactones via Photocatalytic Radical-Radical Coupling. *ACS Catal.* **2023**, *13*, 4894–4902.

(11) Gao, L.; Wang, G.; Cao, J.; Chen, H.; Gu, Y.; Liu, X.; Cheng, X.; Ma, J.; Li, S. Lewis Acid-Catalyzed Selective Reductive Decarboxylative Pyridylation of N-Hydroxyphthalimide Esters: Synthesis of Congested Pyridine-Substituted Quaternary Carbons. *ACS Catal.* **2019**, *9*, 10142–10151.

(12) Jiang, B.; Shi, M. Rhodium(II)-catalyzed intermolecular [3+2] annulation of N-vinyl indoles with N-tosyl-1,2,3-triazoles *via* aza-vinyl Rh carbene. *Org. Chem. Front.* **2017**, *4*, 2459–2464.

(13) Zuo, Z.; Cong, H.; Li, W.; Choi, J.; Fu, G. C.; MacMillan, D. W. C. Enantioselective Decarboxylative Arylation of α-Amino Acids via the Merger of Photoredox and Nickel Catalysis. *J. Am. Chem. Soc.* **2016**, *138*, 1832–1835.

(14) Cismesia, M. A.; Yoon, T. P. Characterizing Chain Processes in Visible Light Photoredox Catalysis. *Chem. Sci.* **2015**, *6*, 5426–5434.

(15) Yang, Z.; Liu, Y.; Cao, K.; Zhang, X.; Jiang, H.; Li, J. Synthetic Reactions Driven by Electron-

donor–Acceptor (EDA) Complexes. *Beilstein J. Org. Chem.* **2021**, *17*, 771–799.

(16) James, M. J.; Strieth-Kalthoff, F.; Sandfort, F.; Klauck, F. J. R.; Wagener, F.; Glorius, F. Visible-Light-Mediated Charge Transfer Enables C−C Bond Formation with Traceless Acceptor Groups. *Chem. – Eur. J.* **2019,** *25,* 8240–8244.

(17) Zhu, M.; Zhou, K.; Zhang, X.; You, S.-L. Visible-Light-Promoted Cascade Alkene Trifluoromethylation and Dearomatization of Indole Derivatives via Intermolecular Charge Transfer. *Org. Lett.* **2018,** *20,* 4379–4383.

(18) Li, Y.; Zhang, J.; Li, D.; Chen, Y. Metal-Free C(sp^3)–H Allylation via Aryl Carboxyl Radicals Enabled by Donor–Acceptor Complex. *Org. Lett.* **2018,** *20,* 3296–3299.

(19) Kandukuri, S. R.; Bahamonde, A.; Chatterjee, I.; Jurberg, I. D.; Escudero-Adán, E. C.; Melchiorre, P. X-Ray Characterization of an Electron Donor–Acceptor Complex that Drives the Photochemical Alkylation of Indoles. *Angew. Chem., Int. Ed.* **2015,** *54,* 1485–1489.
